# Detection of Embryonic Trisomy 21 in the First Trimester Using Maternal Plasma Cell-Free RNA

**DOI:** 10.3390/diagnostics12061410

**Published:** 2022-06-07

**Authors:** Carl P. Weiner, Mark L. Weiss, Helen Zhou, Argyro Syngelaki, Kypros H. Nicolaides, Yafeng Dong

**Affiliations:** 1 Departments of Obstetrics and Gynecology and Molecular and Integrative Physiology, University of Kansas School of Medicine, Kansas City, KS 66160, USA; huizhoulp@gmail.com; 2 Rosetta Signaling Laboratory, Phoenix, AZ 85018, USA; yafengdong0228@163.com; 3 Departments of Anatomy and Physiology & Midwest Institute of Comparative Stem Cell Biology, Kansas State University, Manhattan, KS 66506, USA; weiss@vet.k-state.edu; 4 Fetal Medicine Research Institute, King’s College Hospital, London SE5 9RS, UK; argyrosyngelaki@yahoo.co.uk (A.S.); kypros@fetalmedicine.com (K.H.N.)

**Keywords:** pregnancy, trisomy 21, aneuploidy, antenatal screening, antenatal diagnosis, plasma transcriptome, RNA, machine learning

## Abstract

Prenatal trisomy 21 (T21) screening commonly involves testing a maternal blood sample for fetal DNA aneuploidy. It is reliable but poses a cost barrier to universal screening. We hypothesized maternal plasma RNA screening might provide similar reliability but at a lower cost. Discovery experiments used plasma cell-free RNA from 20 women 11–13 weeks tested by RNA and miRNA microarrays followed by qRT-PCR. Thirty-six mRNAs and 18 small RNAs of the discovery cDNA were identified by qPCR as potential markers of embryonic T21. The second objective was validation of the RNA predictors in 998 independent pregnancies at 11–13 weeks including 50 T21. Initial analyses identified 9–15 differentially expressed RNA with modest predictive power (AUC < 0.70). The 54 RNAs were then subjected to machine learning. Eleven algorithms were trained on one partition and tested on an independent partition. The three best algorithms were identified by Kappa score and the effects of training/testing partition size and dataset class imbalance on prediction were evaluated. Six to ten RNAs predicted T21 with AUCs up to 1.00. The findings suggest that maternal plasma collected at 11–13 weeks, tested by qRT-PCR, and classified by machine learning, may accurately predict T21 for a lower cost than plasma DNA, thus opening the door to universal screening.

## 1. Introduction

Trisomy 21 (T21) is the most common aneuploidy among liveborn infants. Maternal age, and to a lesser extent paternal age, directly impacts T21 prevalence [1,2]. Moreover, while the prevalence of most major birth defects has remained relatively stable over the past 15 years, the prevalence of T21 has increased in some European countries and the USA. The US Centers for Disease Control estimates the maternal age-adjusted prevalence of T21 increased from 1:691 births in 2010 to 1:635 births in 2014 [3,4]. The proportion of women ≥ 35 years giving birth and the prevalence of T21 birth both have increased [5].

A range of structural abnormalities, diseases (e.g., leukemia), and learning disabilities are well-described in T21 individuals. Though the pregnancy loss rate exceeds 50%, surviving T21 individuals typically die in middle age, often from a disorder resembling Alzheimer’s dementia [6]. About 40% of US families reported their T21 child’s medical condition led to financial hardship [7].

There has been steady growth in the use of non-invasive, early pregnancy testing for T21 [8] and for pregnancy complications in general [9]. Lo et al. first described tests that use maternal plasma cell-free (PCF) fetal DNA for non-invasive prenatal screening using massive parallel sequencing technology, and subsequently, others used microarrays [10,11,12], with invasive testing being reserved for diagnosis confirmation. PCF DNA testing for T21 is accurate but considered too costly to be practical as a universal screening test [13]. A Belgium Health Care Knowledge Center report estimated that the cost of a PCF DNA test might be 150 euros (USD 172.50) when performed in a national laboratory [14]. Private laboratory T21 testing costs far more, and universal PCF DNA testing for T21 is currently not cost-effective [15]. We hypothesized that a PCF RNA test for T21 based on qRT PCR, if similar in predictive accuracy, could be operationally cost-effective compared to PCF DNA.

The PCF transcriptome includes both coding and noncoding RNAs capable of modulating angiogenesis, cell proliferation/death, tumor cell invasion, and cell-to-cell communication, to list a few examples [16,17,18]. That the PCF transcriptome is altered by numerous diseases suggests its potential for prognostication [19,20,21]. Poon et al. first described PCF fetal RNA in the maternal circulation two decades ago [22]. Others have since described pregnancy-derived RNAs in maternal blood and suggested the possibility of screening for pregnancy complications [23,24,25,26,27,28,29,30]. MicroRNAs (miRNA) are a class of small non-coding RNAs present in the transcriptome. Multiple studies suggest miRNA and other noncoding RNAs might serve as disease biomarkers [31,32,33,34,35], or predict pregnancy complications including T21 [8,36,37]. Unfortunately, none of those efforts have survived a validation study [36,37,38,39,40,41].

We described a panel of five PCF RNAs that effectively identified by 16 weeks, women destined for extreme preterm birth (e.g., birth ≤ 32 weeks) whether due to labor or preterm premature rupture of membranes [42]. Our in vitro work suggested the prognostic RNAs originate from the placenta [42]. Here, we hypothesize that transcriptional abnormalities in maternal plasma will reflect T21 and can be predicted reproducibly by qRT-PCR. Other reports provide support for the hypothesis such as T21 studies on the retina [43], peripheral mononuclear cells [44,45], astrocytes [46], fibroblasts [47], placenta [40,48,49,50], and blastocysts [51]. We can identify only one prior attempt to identify plasma RNAs for T21 screening. Zednikova et al. attempted in 2020 to identify differentially expressed maternal plasma miRs in pregnancies with a first-trimester embryo using microarrays and placental studies [41]. Unfortunately, the validation effort failed.

Our first objective here was to seek differentially expressed PCF RNAs (both mRNAs and miRNAs) in first trimester pregnancies with T21 embryos compared to healthy embryos. Microarray-based analysis failed to identify a single differentially expressed RNA after multiple comparisons correction, perhaps due to the conservative nature of such correction. In response, we used the uncorrected data to generate a list of potentially differentially expressed RNAs based on the effect size, consistency of response, and a lack of a race impact and tested those by qPCR using the same aliquot of cDNA that was used for the microarray studies. This effort derived a list of 54 plasma mRNA and miRNA for further study. Our second objective was to validate the 54 RNA predictors by using a qRT-PCR test in an independent cohort of 998 women sampled in the first trimester (50 T21, 948 controls). Previously, Yang et al. had applied support vector machine learning (ML) classification to PCF DNA screening for T21 diagnosis with success [52], and we wondered whether we could improve upon our prior unpublished efforts by using ML. Eleven (11) ML algorithms were surveyed. The four top-performing algorithms were decision tree-based methods. Next, the effects of training/testing partition size and dataset class imbalance were evaluated. Addressing imbalance, both oversampling and SMOTE were effective at improving ML predictive outcomes. Our findings suggest the utility of PCF RNA testing via qRT-PCR coupled with ML for noninvasive T21 prediction in the first trimester.

## 2. Materials and Methods

### 2.1. Cohort

The plasma samples were prospectively obtained by Fetal Medicine Research Institute, King’s College Hospital, London, United Kingdom, after signed informed consent was obtained between 11 and 13 weeks gestation and stored at −80 °C until used. All T21 diagnoses were confirmed either by invasive testing or at delivery. Two sets were generated from the stored samples. The first set was used for discovery and confirmation and consisted of 20 samples selected at random by one of the authors (AS) such that there were 10 T21 “cases” and 10 with a normal pregnancy outcome “controls”. In this set, half of the mothers self-identified as “White” and half as “Black”. The second sample set was used for the initial validation study. It consisted of 50 T21 cases and 968 normal controls selected at random from the biobank without regard to race. In contrast to the cohort used for discovery, the initial validation cohort had five races represented in normal controls and three races represented in the T21 cases. De-identified samples were transferred to the KUMC laboratory (CPW), and all laboratory processing was performed by masked investigators (i.e., blinded to case/control status of the sample). Gestational age was based on crown-rump length (CRL).

### 2.2. Laboratory Methods

#### 2.2.1. RNA Extraction

Total PCF RNA was extracted by Rosetta Signaling Laboratory using a proprietary method (Rosetta Signaling Laboratory, Phoenix, AZ, USA). The EDTA sample volume extracted was typically 500 µL. The average total RNA (±SD) extracted was 25.02 ± 14.03 µg in approximately 40 µL in the discovery/confirmation phase and 15.74 ± 15.81 µg in 20 µL in the initial validation phase (see Supplemental Table S1). RNA yield was assessed by a nano spectrometer (NanoDrop Technologies, Wilmington, DE, USA) and RNA integrity was confirmed by an Agilent bio-analyzer (Agilent, Santa Clara, CA, USA).

#### 2.2.2. Discovery Study

##### Microarrays

Affymetrix Human Exon 1.0 ST Array (Santa Clara, CA, USA) and the Affymetrix GeneChip miRNA array (847 human miRNAs) were used. All microarrays were processed and read in 2011 by the KUMC Genomics Core according to the manufacturer’s instructions using a GeneChip Microfluidics 450 Center and GeneChip 3000 scanner with 7G upgrade (Applied Biosystems) with Affymetrix GeneChip Command Console Software. Microarray RNA quality control evaluation was performed before each microarray (see Supporting Information). Primary data analysis was conducted by the KUMC Bioinformatics Core following minimum information about a microarray experiment guidelines [53], and they conducted gene expression quality controls (see Supporting Information—qc on microarray data).

##### qPCR

Potential mRNA markers were initially sought based on being up/down-regulated (*p*-value ≤ 0.05 prior to FDR) vs. normal control and with a fold change exceeding ±1.50 after PARTEK RMA data analysis. Potential miRNA markers were selected similarly. Unfortunately, no RNAs were identified as differentially expressed using this approach. Next, the pre-FDR correction expression data were ranked by *p*-value and reordered by narrowness of distribution for each variable in the T21 group using the MetaCore Bioinformatics Suite. As a result, potential RNA markers of T21 were rejected if the change in expression shown in the microarray for that RNA was due to its change in 3 or less of the T21 women.

Some 100 of the highest-ranked RNAs were then subject to qPCR using the same cDNA aliquot used for the microarray studies. RNAs identified by qPCR as differentially expressed were considered for analysis in the initial validation study. Two microliters of the pre-amplified cDNA samples was diluted into a 10 µL PCR reaction mix, followed by qPCR. Multiplex qPCR reactions were performed using the ViiA 7 Real-Time PCR System. The primers for the qPCR studies were custom designed and synthesized by Integrated DNA Technologies (IDT, Coralville, IA, USA). Information about the primer sequences is available from the authors. The probe sets in each well included a spiked-in cDNA and the primers for the RNA under study plus normalization and spike-in genes so that all three were run in the same well to minimize assay variation. Threshold cycles (Ct values) of qPCR reactions were derived using QuantStudio™ Software V1.3 (Applied Biosystems, Foster City, CA, USA). Marker RNAs were normalized to the housekeeping control and a spiked-in cDNA. The ΔΔCts were determined and the relative fold value was calculated using the 2^−ΔΔCt^ method (or 2^ΔΔCt^ for down-regulated RNAs).

#### 2.2.3. Validation Study

##### qRT-PCR Assays

*mRNA RT:* The RNA samples were diluted, and a master mix was prepared including dNTP mix, Omniscript Reverse Transcriptase, and Random Primer (Invitrogen, Carlsbad, CA, USA). The mRNA of each sample was converted into cDNA at 37 °C for 60 min per the manufacturer’s instructions.

*miRNA RT:* The miRs were polyadenylated using reagents from the Invitrogen NCode miRNA First-Strand cDNA Synthesis Kit (ThermoFisher, Waltham, MA, USA). The polyadenylated microRNA was reverse transcribed to generate the first strand of cDNA according to the manufacturer’s protocol.

*Preamplification:* One microliter of RT samples was prepared with the preamplification Mix Reaction and underwent 12 cycles of amplification.

Two customized probe-based microfluidic PCR Cards with 384 wells were developed for the selected mRNA and small noncoding RNA markers using a proprietary method (Rosetta Signaling Laboratory, Phoenix, AZ, USA). The probe sets in each well included a spiked-in cDNA and the primers for the RNA under study plus normalization and spike-in genes so that all three were run in the same reaction well to minimize assay variation. Threshold cycles (Ct values) of qPCR reactions were derived using QuantStudio™ Software V1.3 (Applied Biosystems, Foster City, CA, USA). Marker RNAs were normalized to the housekeeping control and a spiked-in cDNA. The ΔΔCts were determined and the relative fold value was calculated using the 2^−ΔΔCt^ method (or 2^ΔΔCt^ for down-regulated RNAs).

### 2.3. Statistical Analysis

#### 2.3.1. Validation Analysis

An independent cohort of 1018 patient samples with maternal and pregnancy variables available including maternal age, weight, height, race (self-identified), and gestational age at sampling was employed. Following RNA extraction and RNA quality assessment, 998 RNAs were subjected to card-based PCR. The results were first subjected to quality control assessment using on-card-positive PCR controls, negative controls, and then normalized using two proprietary housekeeping genes. The normalized results were input into eleven classification algorithms within the CARET package using the freely available R Project for Statistical Computing downloaded from CRAN. R scripts used for analysis are available from the authors. The ML dataset is provided in Supplemental Table S5.

#### 2.3.2. Data Preprocessing

ΔΔCt values were not normally distributed by either the Shapiro–Wilk test or scatterplot inspection, and attempts were made to normalize the dataset using common procedures, e.g., centering (subtraction by the average value) and scaling (dividing by the standard deviation), or transformation via lognormal, log10, log2, square, square root, and simple combination of mathematical transformations. Ct values failed to normalize (not shown), and fold-change values were used without transformation (see Supplemental Table S5).

In addition to normalization, a correlation matrix of the expression results was generated (see Supplemental Table S4). These two characteristics suggested this dataset was unlikely to be tractable to linear methods. Since tree-based learning methods are notably insensitive to the characteristics of the predictor, those methods were part of the survey.

#### 2.3.3. Differential Expression

The expression levels between cases/controls were compared using the Mann–Whitney–Wilcoxon test in R. The raw *p*-value was adjusted for multiple comparisons using false discovery rate correction methods: Q-values, Benjamini–Hochberg, Benjamini–Yekutieli, Holm, Hochberg, Hommel, and Bonferroni family-wise correction for potential RNA markers with *p* < 0.05, two-tailed labeled as significant. Boxplots and ROC curves for differentially expressed genes were generated using the R statistical package, saved as enhanced metafiles, and edited for publication using Canvas x19 (build 333).

#### 2.3.4. Machine Learning

Complete datasets were used for ML, and cases/controls with incomplete data were omitted. The dataset was split randomly into training and testing partitions using R’s “createDataPartition” function and a fixed seed. The models were trained using repeatedcv, which applies repeated k-fold cross-validation (CV) with options: number = 10, repeats = 5. This means the training dataset was randomly divided into 10 parts and then using each of the ten parts as a testing dataset for the model trained on the other nine. The average of the 10 error terms is thus obtained. In 5 repeats of 10-fold CV means that the average of error terms obtained by performing 10-fold CV five times was obtained. Each model was tuned using the metric Kappa or accuracy to optimize and use the algorithm defaults for tuning variables. Due to the trial-and-error nature of ML, we scanned model performance across eleven ML algorithms in the CARET package using a fixed allocation of the dataset (70% to the training set) and then tested using the holdout (30% of the whole dataset for testing) set. The eleven ML algorithms used were: generalized boosted regression models (GBM), decision trees and rule-based model that extended the work by Quinlan (C50, Quinlan, 1993, ISBN:1-55860-238-0), random forest (RF), the boosted adabag model described by Freund and Schapire (adaboost, [54]), Naïve Bayes (NB), multivariate regression splines (MARS) model by Friedman (Earth package, see Friedman’s papers “Fast MARS” and “multivariate adaptive regression splines” <doi:10.1214/aos/1176347963>), mixture and flexible discriminant analysis (MDA), linear discriminant analysis (LDA), neural networks (NNET), support vector machine (spherical, SVM), and classification and regression trees (CART) in Comprehensive R Archive Network (CRAN) for R (R build 4.0.3 through 4.1.2). The four best-performing algorithms defined by Kappa or accuracy were evaluated using tuning and ensemble techniques. In each case, model performance was evaluated by applying the final model to the test dataset and the generation of a confusion matrix. The accuracy and Kappa of each final model were recorded and graphed vs. training partition size and ROC curves were prepared using the ROCR package.

To evaluate the impact of training partition size and class imbalance on ML performance, model performance data (Kappa and accuracy) was plotted against training partition size (45–90%) and with or without the application of four methods that address class imbalance: Oversampling, downsampling, or the synthetic minority oversampling technique (SMOTE) [55] or the random oversampling technique (ROSE) [56]. Graphs were generated in R and saved as EMF files. The EMF files were imported into Canvas x19 (build 333) and assembled into final figures and saved in TIFF format.

## 3. Results

### 3.1. Discovery

#### 3.1.1. Study Subjects

A cohort of 40 women (½ with a T21 fetus; ½ Black, ½ White) sampled in the first trimester had PCF RNA isolated. The biographical information is shown in Table 1. Note that the maternal age of the T21 group compared to the normal group differed significantly using one-tailed testing based upon the hypothesis that older MA would increase the risk of T21 (37.3 ± 4.1 y T21 vs. 33.7 ± 5.0 normal, *p* < 0.05 one-tailed). Race and ethnicity did not differ significantly between the two groups. PCF RNA was extracted from the entire plasma sample using a proprietary method (Rosetta Signaling Laboratory LLC, Phoenix, AZ, USA). The average yield was 25.02 µg ± 14.03 µg (±1 SD, range 9.60–72.63 µg, n = 40; see Supplemental Table S1).

RNA from 20 subjects (½ with a T21 fetus; ½ Black, ½ White) was selected at random, reverse transcribed, and applied to Affymetrix Exon 1.0 ST microarrays for mRNA expression and Affymetrix GeneChip miRNA microarray for noncoding RNAs. After background subtraction, normalization, and differential expression analysis using the RMA protocol, 232,119 exons were read of which 2686 (1.2%) were located on chromosome #21. In total, 10,280 exons differed in expression by >1.5-fold between T21 and normal control after accounting for the effect of race and ethnicity and before application of *p*-value correction for false discovery rate (FDR). However, none of these RNAs were differentially expressed following correction for the FDR (original dataset, Supplemental Tables S2 and S3).

#### 3.1.2. Focused Search Following Effect Size Stratification and Removal of RNAs Affected by Race

The microarray data was ranked by effect size, *p*-value, and narrowness of distribution, and the top 100 RNAs were subjected to qPCR (not shown). After expression was normalized to internal controls, fifty-four (54) RNAs were differentially expressed by qPCR (results not shown). Thirty-six mRNAs were differentially expressed by qPCR and the origins of these RNAs were distributed on seven different chromosomes. One-third of the exons were up-regulated in T21 (12 out of 36) and two-thirds were down-regulated in T21 (Table 2a). Two of the RNAs originated on chromosome #4, 1 on chromosome #11, 2 on chromosome #14, 1 on chromosome #16, 2 on chromosome #17, 1 on chromosome #19, and 19 on chromosome #21 (see Table 2a). There were 18 noncoding RNAs differentially expressed based on qPCR (Table 2b). The RNA’s genes of origin involved 13 chromosomes; about half of the noncoding RNAs were up-regulated in T21, and the other half were down-regulated. Three noncoding RNAs originated on the X chromosome, two noncoding RNAs originated on chromosomes #2, 3, and 5, and one noncoding RNA originated on chromosome #8, 9, 11, 13, 14, 15, 18, 19, and 21 (Table 2b). Thus, 54 RNAs were identified by qPCR as being differentially expressed in the cDNA from the same samples run on the microarrays (see Table 2a,b). These RNAs were used to design a custom-made 384 microfluidic-well card manufactured by Applied Biosciences for use in validation testing described below.

### 3.2. Validation Study

#### 3.2.1. Study Subjects

PCF RNA was extracted from an independent cohort of 1018 women sampled in the first trimester. An average of 15.74 ± 15.81 µg of total RNA in 20 µL (range of 3.34–88.02 µg) was isolated from the 500 µL plasma samples provided to Rosetta Signaling Technologies, LLC (see Supplemental Table S1; note Rosetta Signaling Technology’s historical data indicates an average yield of 15.78 ± 7.26 µg in 20 µL from 500 µL of plasma, n = 3000, data not shown). Twenty samples, all from the control group, were rejected due to either sample ID mismatch, hemolysis, or low RNA quality, leaving 998 samples for analysis, including 50 T21 and 948 normal controls. The biographical data and sampling time of the cohort are summarized in Table 3 (and the data used for ML in Supplemental Table S5). The normal controls, e.g., birth of euploid baby at term, included five self-identified racial and ethnic groups: White (698, 73%), Black (144, 15%), South Asian (48, 5%), East Asian (24, 2.5%), and mixed (37, 3.9%). The “cases”, e.g., birth of a T21 baby, included 3 self-identified racial and ethnic groups: White (42, 86%), Black (6, 12%), and East Asian (2, 4%). Due to the imbalanced dataset, “race” was excluded as a predictor variable for ML. Note, the gestational age at sampling of the T21 group was higher by an average of 0.3 weeks compared to control, but the range was the same: 11.2–14.1 weeks (Table 3). The average maternal age was significantly higher in the T21 group (T21 37.6 ± 4.4 years, n = 50 vs. normal 31.7 ± 5.64 years, n = 948). The receiver operator characteristic (ROC) curve demonstrates that maternal age was a predictor of T21 risk, as indicated by the area under the curve (AUC) of 0.796, 95% confidence interval [CI] 0.734–0.850. Both the maternal height and weight varied significantly among racial and ethnic groups (not shown) but did not differ between T21 cases and normal controls.

#### 3.2.2. Validation Results

**Figure 1:**
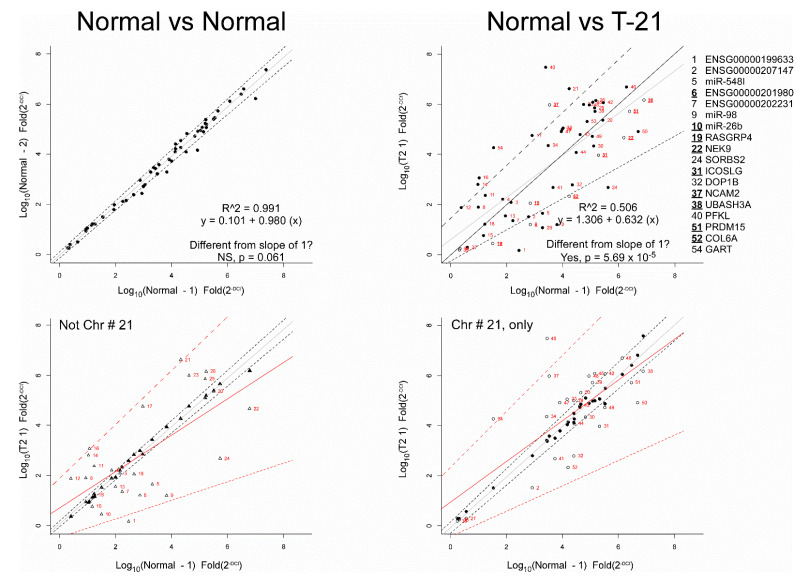
**RT-PCR results from Validation dataset.** (**Top left**) Data from the 948 controls were randomly allocated into two groups and then average expression of the 54 RNAs was plotted. The regression line (R^2^ > 0.99) falls along the slope of 1 (indicated by the grey line) and within 95% confidence interval (indicated by the broken lines). (**Top right**) Averaged expression data from the 50 T21 cases were plotted against averaged expression data from the 948 controls. Linear regression (R^2^ < 0.51) of the data is shown in grey (95% confidence interval indicated by broken lines) and is significantly different from the slope of 1 (line shown in black). Note that the RNAs evaluated are indicated by plate ID in red. The open circles represent RNAs found to be differentially expressed between T21 and control using Mann–Whitney–Wilcoxon test followed by the Bonferroni correction for false discovery rate. The numbers of selected RNAs and their gene name are provided to the right, with the nine differentially expressed genes shown in bold and underline. (**Bottom left**) RNAs found on chromosomes other than #21. In filled triangles, the average expression of the controls is plotted after being randomly allocated into two groups. In open triangles, the T21 case expression is plotted against average expression of controls. (**Bottom right**) RNAs found on chromosome #21 are shown. In filled circles, data from the controls were randomly allocated into two groups, then averaged and plotted. In the open circles, the average expression of T21 cases is plotted against the average expression of normal. Bottom two panels: The line fitting this data and the 95% confidence interval is shown. The solid black lines show the regression fit for control vs. control (the broken lines indicate the 95% confidence interval). The solid red lines show the regression fit for T21 vs. control (the broken lines indicate the 95% confidence interval). The numbers next to the data points of T21 vs. control indicate the RNA identification found in the plate.

To visually assess the differential expression of the fold values of the 54 RNAs, the normal control group was divided randomly in half and the log-fold value of each RNA was plotted (Figure 1, left). Following simple linear regression (gray line), the R^2^ of the predicted model was >0.99 and the predicted slope was not significantly different from a slope of 1 for the normal control RNA log-fold values in partition 1 vs. normal control RNA log-fold values in partition 2 (the dotted lines indicate the 95% confidence interval). This shows the reproducibility of the qRT-PCR data.

As shown in Figure 1, right, the log-fold value for RNA markers in the normal control group was plotted against the log-fold value for the RNAs in the T21 group, and a simple linear regression line was fitted (gray line). The R^22^ of the predicted line was < 0.51, and the slope of the regression line (0.632) was significantly different from a slope of 1 (the black line, *p* < 0.001). This result supports the differential expression of RNAs between T21 and normal control. It was noted that differentially expressed RNAs were found both on chromosome #21 (Figure 1, bottom right), and not on chromosome #21 (Figure 1, bottom left).

To further investigate differential expression between case/control, Mann–Whitney–Wilcoxon testing was used to compare T21 to normal control, followed by *p*-value correction for false discovery rate via Q-values [57], or the Benjamini–Hochberg method [58]. Fifteen (15) differentially expressed RNAs were identified by this approach (Table 4). In contrast, 13 RNAs were identified as differentially expressed in T21 using Benjamini–Yekutieli correction [59], nine RNAs using the Holm [60], Hochberg [61], Hommel [62], or Bonferroni [63] methods. The nine differentially expressed RNAs identified by Bonferroni are represented as open circles in Figure 1, right.

**Figure 2:**
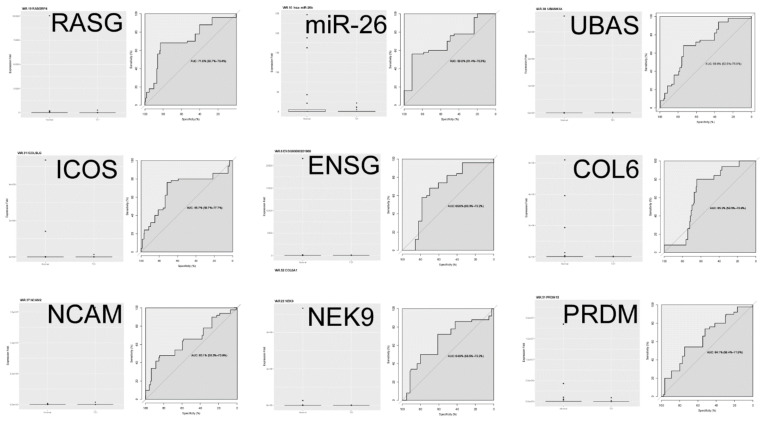
Boxplots and receiver operator characteristic (ROC) curves for the nine differentially expressed RNAs following Bonferroni correction for false discovery rate.

In Figure 2, nine differentially expressed RNAs are plotted individually to compare differential expression and a ROC curve. Summarizing the qRT-PCR findings presented so far: (1) PCR RNA from an independent and more diverse patient cohort than used in the discovery phase indicates validation of 9–15 RNAs originally suggested by microarray/qPCR as being differentially expressed between T21 case and normal control; (2) the AUC indicates that the predictive power of each of the 9 differentially expressed RNAs falls into a “fair” 0.6–0.7 range of accuracy, similar to what was found modeling maternal age, alone. This level of accuracy would not likely be clinically useful.

#### 3.2.3. Application of ML Classification Algorithms to qRT-PCR Data

**Figure 3:**
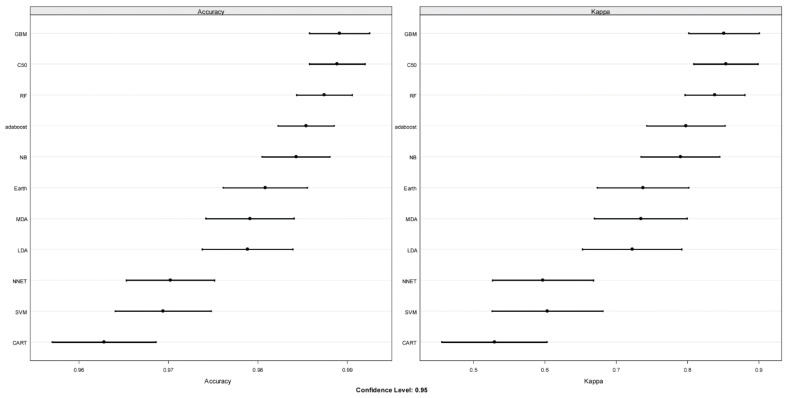
Using the CARET package in R, eleven machine learning (ML) algorithms were surveyed to predict trisomy 21 (T21). Left panel displays performance based upon accuracy, and the right panel displays the Kappa value. The bars represent the 95% confidence interval. Algorithms were all trained on the randomly allocated 75% partition using 10-fold cross-validation, e.g., the training dataset is randomly allocated into 10 parts and trained on 9 and tested on the one holdout, and this was repeated 5 times. Abbreviations: GBM: gradient boosting machine; C50, classification of data and decision tree algorithm C5.0; RF: random forest; adaboost, a decision tree model that uses a boosting method to improve learning rate; NB: naïve Bayes, a classification method that is based on Bayes’ theorem; Earth: multivariate adaptive regression splines model; MDA: flexible discriminant analysis, LDA: linear discriminant analysis; NNET: neural network; SVM: support vector machine; CART: classification and regression trees.

Eleven ML classification algorithms found in the CARET package of R were used for surveying. For training and performance evaluation, the dataset was parsed randomly into 70% training and 30% testing partitions. The ML survey results are shown in Figure 3. The top four algorithms, GBM, C50, RF, and adaboost, had an average accuracy of >98% and an average Kappa of >80%. GBM had the highest accuracy, and C50 had the highest Kappa. Patient-specific variables such as gestational age at sampling and maternal age were included or excluded during this modeling with no significant impact on Kappa (data not shown).

**Figure 4:**
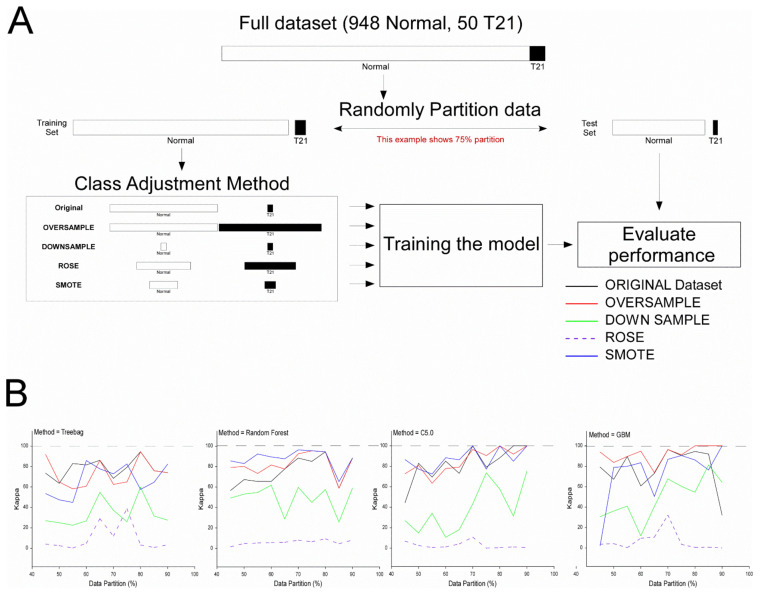
Effect of training partition size and class imbalance on three machine learning algorithms: Random forest, C5.0, and GBM. (**Panel A**) (top) shows the workflow. First, the dataset was randomly partitioned into training and testing (evaluation) sets from 45% of the data allocated to training, up to 90% of the data. To evaluate the impact of class imbalance, four different methods were applied that rebalance the class size. Specifically, oversampling, which randomly adds to the minority group with repetition to parity; downsampling, which randomly eliminates from the majority group to parity; or using ROSE or SMOTE, which are synthetic methods that created equal size groups using different approaches. Next, three models, random forest, C5.0, or GMB, were trained using 10-fold cross-validation with 5 repeats, then the performance of each model was evaluated using the holdout dataset. The performance was evaluated using Kappa, and the results were plotted in (**Panel B**). Note that generally, ROSE was ineffective at improving the algorithms, followed by downsampling. In contrast, Smote and oversampling produced gains in performance for random forest, and less consistently for C5.0 and GBM, compared to the original dataset.

We were concerned either the imbalanced dataset (50 T21 cases vs. 948 controls) or the training partition size might affect ML modeling outcomes. Thus, 4 statistical methods to address class imbalance were employed: Oversampling, downsampling, ROSE, and SMOTE, and the predictive accuracy and Kappa were measured across training partitions ranging from 45–90% (Figure 4). In general, applying ROSE (broken line) and downsampling (green line) tended to decrease the performance of the ML algorithms compared to the ORIGINAL (black line) dataset, while applying oversampling (red line), and SMOTE (blue line) tended to produce a modest increase in model performance. Predictive performance (Kappa) using the original dataset tended to rise by increasing the size of the training partition until it was 70–80%. Oversampling and SMOTE also tended to improve performance over the original dataset when the training partition size was 70%.

Table 5 shows the top seven “important” variables used for the best GBM, C5.0, and RF ML predictive modeling algorithms. The ML algorithms identified from 5 to 20 “important” variables but for simplicity only seven are shown in Table 5. The full dataset is provided in Supplemental Table S6. ML algorithms identified “important” variables mathematically, and it is interesting that in some ML models, differentially expressed RNAs were “important” (highlighted by yellow or blue fill). For example, GBM used only differentially expressed RNAs. In contrast, C5.0 and RF “important” variables were not differentially expressed. Notably, GART was identified as “important” by C5.0 and RF (highlighted by green fill) but was not differentially expressed (see Table 4). Note that many of the variables were positively correlated (see Supplemental Table S4). Note that some of the best predicting models included maternal age. The most parsimonious model was generated by the C5.0 method using only 5 miRNAs; that model had 99% accuracy and a Kappa of 88%. Two models were performed without error and yielded AUCs of 1.00 (Table 5).

The 5 top-performing ML algorithms containing 9 differentially expressed variables plus MA yielded an average accuracy of >98.3% and kappa of >79.3% (see Supplemental Table S6). In contrast, a selection of 9 variables of the highest average weight across the top five models yielded an average accuracy of >98.8% and kappa of >89.4%. This shows that ML-based predictive modeling generated models using not only RNAs found to be differentially expressed by qRT PCR but also other RNAs not differentially expressed by qRT PCR.

## 4. Discussion

### 4.1. Main Findings

These results demonstrate for the first time that the maternal first trimester PCF transcriptome is predictably altered by embryonic T21 and suggests that ML-based modeling using a subset of differentially expressed RNAs and biographical variables might identify T21 pregnancies with a prognostic accuracy similar to the current gold standard, PCF DNA. The discovery mRNA and miRNA microarray study was conducted in 2011 and the qRT PCR RNAs were identified in 2013. The 36 mRNAs combined with maternal age, weight, and race yielded a prior unpublished model (83% DR, 0% FPR; CPW, YD, unpublished observations) which was superior to the biochemical testing in clinical use at the time [64], but not as accurate as PCF DNA [13].

While the maternal plasma transcriptome has not changed over the last decade, the analytic tools have improved. We posited that the PCR RNA isolation and normalization protocol we developed for the prediction of preterm birth [42] would be useful for predicting other pregnancy complications such as T21. Other researchers have suggested the identification of a standardized RNA extraction and normalization protocol that provides both high yield and high-quality RNA is essential for downstream analysis to become reproducible and validated across RNA analysis methods and laboratories (e.g., see discussion in [41]). We believe the proprietary PCR RNA isolation protocol used in the current study is an important component for reproducibility. Follow-on studies using RNA isolated from placenta-derived extracellular vesicles should provide additional insight into the source of these RNA T21 biomarkers [26,65] and support our thesis [42] and that of others [26,40,66] that dysregulation of placental physiology is an antecedent to many pregnancy complications.

It was a concern that our microarray “Discovery” failed, e.g., the microarray was insufficiently sensitive to detect differentially expressed RNAs. To address this concern, we stratified the microarray data by effect size and performed qPCR analysis on candidate RNAs. We posited that since qPCR has greater sensitivity than microarray, and since the smaller cohort reduces the FDR correction factor by reducing the number of comparisons per trial, differentially expressed RNAs would be found. Moreover, it was with this approach that differentially expressed RNAs were identified for testing in the next stage of the workflow. Using qRT-PCR in the initial validation, the method applied to correct FDR impacts the number of significant differentially expressed RNAs, and the use of nested RT-PCR was sufficient to detect 9–15 differentially expressed RNAs. Both Q-values and Benjamini–Hochberg discovered the same set of 15 differentially expressed RNAs. The most conservative FDR correction methods, Holm, Hochberg, Hommel, and Bonferroni, found the same set of nine differentially expressed RNAs. Of these nine RNAs, five originate on chromosome #21. Interestingly, ML independently identified “important predicting variables” that overlapped with the differentially expressed RNAs (highlighted yellow or blue in Table 4 and Table 5). Also of interest, ML through its mathematical process identified other “important predictive variables”, such as GART, highlighted in green in Table 4 and Table 5. GART would not have been considered as a predictor of T21 if one only considered RNAs found to be “significantly different”. On the other hand, the ERG fusion gene, found to be differentially expressed following Q-values and Benjamini–Hochberg FDR correction, was not an “important predictor for ML” for ML algorithms tested here.

ML classification allowed for the first time the prediction of embryonic T21 using a minimally invasive maternal sample collected at 11–13 weeks [67]. The improvement in accuracy over our earlier effort was dramatic, yielding algorithms with predicted AUCs up to 1.00. Just as important, the approach permitted test simplification, reducing the number of RNA markers down from the original 54 to a more manageable number. In retrospect, we found that many of the prospective biomarker RNAs were highly correlated (Supplemental Table S4). It is likely that this reduces the efficiency of ML-based variable selection, and a refinement of the biomarker list to include variables with low correlation might further improve ML classification. The heteroscedastic nature of qPCR and qRT-PCR data is a concern for regression analysis, analysis of variance, and ML methods that assume a linear relationship between independent and dependent variables. Decision tree methods, support vector machine, naïve Bayes, and regression machine learning methods were employed here because they are less sensitive to these features.

It is unclear whether the chromosome location of the gene of origin is a good predictor of RNA importance. For example, 5 of 9 or 9 of 15 differentially expressed RNAs originated from chromosome #21 (Table 4). However, as shown in Table 5, most ML algorithms found RNAs originating from genes located on other than the #21 chromosome to be “important predictors”. The number of RNAs from chromosome #21 ranged from more than half for GBM methods to one algorithm that consisted of five noncoding RNA predictors with only one of which originated from genes on the #21 chromosome (Group 2, up.c50, 70% training).

### 4.2. Strengths and Limitations

One strength of the current investigation is its novelty. This is only the second published study of plasma transcriptome changes linked to a chromosomal abnormality, whether from an affected individual or a pregnant woman with an affected embryo/fetus. It is also the first to be successfully validated. Further, this appears to be just the second application of ML to a plasma transcriptome dataset, and the result was a dramatic improvement in predictive accuracy and a reduction in the number of RNAs required. A second strength of the study was the proprietary plasma RNA extraction that increases total RNA yield per milliliter to microgram quantities compared to the nanogram amounts with commercial kits [68,69]. The high-quality RNA resulted in consistency of expression across the normal control group, as indicated by Figure 1, left, and subsequent initial validation in an independent cohort using qRT-PCR. No PCF RNA test can be reproducible if the RNA extraction is not.

One limitation of the study is its case-control design with a relatively high T21 prevalence (50% in discovery/confirmation and 5.0% in the initial validation). While the results should be applicable to the general population because the differentially expressed markers are likely due to the presence of an extra #21 chromosome, there are important unknowns to resolve. First, we do not know the origin of the RNA markers (embryo/placenta, mother, or some combination). While our experience with preterm birth [42] suggests they are likely from the placenta, it is possible unrecognized maternal mosaicism might lead to a false-positive test. Second, we narrowed our outcomes to normal “controls” and T21 “cases”. We do not know whether embryonic mosaicism (which represents 2–3% of all T21s) or confined placental mosaicism will alter the maternal plasma transcriptome in the same fashion. It has been suggested that the impact of mosaicism is inversely related to the percentage of cells affected [70] and that the long-term prognosis of children from a pregnancy with confined placental mosaicism is reassuring [71], though follow-up studies of both conditions are sparse. However, if true, and the plasma transcriptome reflects the phenotype of the disorder, an inability to detect a mosaic T21 may provide valuable ancillary information regarding the postnatal phenotype of the child. Third, blood was sampled at 11–13 weeks of pregnancy. It is possible that samples obtained later in gestation might lead to different results. Similarly, additional work is needed to evaluate RNA isolation efficiency, normalization RNAs, and RNA biomarkers across gestation to expand understanding of the T21 predictive performance over the pregnancy. Fourth, several RNAs used for ML had high degrees of correlation. Ideally, one would remove correlated variables prior to ML to increase the efficiency to identify important predictors. Finally, all pregnancies studied were singletons. We have no experience with dizygotic multiple gestations and worry that this would compromise test accuracy by marker dilution since 8 of the 11 RNA markers used by the two best-performing prognostic algorithms display reduced expression in T21 compared to normal controls. Dilution would increase the false-positive rate.

### 4.3. ML Results

Classification by ML employs mathematical tools to predict class, e.g., case or control, and, as such, is a branch of artificial intelligence. One advantage of ML is it lacks underlying predispositions or user biases. It uses numerical methods to identify salient features, or, in this instance, RNAs predictive of T21. Importantly, large data sets can be rendered tractable through the application of ML. Generally, those datasets number in the tens or hundreds of thousand samples. Here, the use of one thousand samples is still on the “low end” of ML’s powerband and a larger dataset could improve ML modeling. ML methods may be affected by imbalanced datasets. We found improved performance by applying two methods that specifically address class imbalance. In addition to the impact of dataset size and class imbalance, ML is subject to overfitting, which means our predictive accuracy and Kappa values may be overly optimistic.

ML has proven robust and efficient at “mining”, e.g., extracting salient features from large datasets. Importantly, tree-based ML algorithms are not strongly affected by the lack of normality or constant variance as is characteristic of qPCR and other genomic datasets, in contrast to linear regression or ANOVA methods statistical inference based upon homoscedastic, normality, and unimodal data assumptions. While we posited that tree-based methods might be most useful here, there are no a priori rules to prospectively identify optimal ML algorithms. The CARET package in R contains more than 130 ML algorithms to evaluate, some are regression based, and must be modified for classification. Here, we employed a simplified workflow and sampled 11 of these 130 algorithms.

Because class imbalance can affect the efficiency of ML modeling [72,73,74], we investigated this possibility by using four methods: Oversampling, downsampling, ROSE, and SMOTE. These methods employ different tactics to balance class in the training dataset (see [75] for a detailed discussion). Differences in the efficiency of the four methods for model training were revealed by their predictive performance on the independent test data. Oversampling and SMOTE generally improved performance over the original dataset, while downsampling and ROSE generally decreased performance. Models trained using partition sizes >66% and <85% performed better at predicting T21. While our results are encouraging and show improvement over previous modeling efforts, the testing of a new, independent, and more diverse patient population is necessary to validate/refine the predictive models and determine whether they hold up across race and ethnic groups and across gestational epochs. Furthermore, it may be possible to improve the predictive power with little to no added cost by the inclusion of both maternal and paternal age and other biographical variables. Fortunately, the implementation of “simple” qRT-PCR technology coupled with the minimally invasive sampling early in pregnancy lowers the barriers to follow-on this work.

One interesting finding was that ML used some, but not all the RNAs found to be differentially expressed. For example, the ERG fusion gene was found to be differentially expressed after FDR correction via Q-values and Benjamini–Hochberg method. This variable was not found as an important variable in any of the ML models shown (see Table 5). In contrast, ML identified some important predictor variables that were not differentially expressed as important ones, e.g., GART. Since ML uses mathematical rather than statistical methods to learn and predict class, it is interesting ML independently identified many chromosome #21 and differentially expressed RNAs as important predictors. In the future, it might be valuable to prioritize markers by clustering via gene ontology, pathway, or Bayesian-like convergent functional genomics approach [76,77].

### 4.4. Interpretation

Our a priori assumption was that RNAs originating from genes on the #21 chromosome genes would be overrepresented as markers and overexpressed. That assumption proved incorrect, and the impact of the extra #21 chromosome on transcription is broader, impacting the transcription of genes on numerous chromosomes including the X chromosome. It is likely that most if not all the differentially expressed RNAs are related to the presence of the extra #21 chromosome in the conceptus regardless of the location of the chromosome of origin. If true, it is equally likely the maternal transcriptome will be altered by other significant aneuploidies, such as T13, T18, and XO. If these chromosome disorders are like T21, e.g., amenable to the identification of predictive maternal PCF RNA markers, a high-throughput PCR ‘aneuploidy’ card might be generated, assembled, and tested for USD 100 or less when run at scale (exclusive of sample transportation). Further, a single 500 µL PCR RNA extraction yields enough RNA to run both an aneuploidy panel and a panel for the prediction of PTB ≤ 32 weeks [42], potentially providing a universal screening tool at low overall costs.

## 5. Conclusions

Maternal PCF RNA and biographical predictors in ML-based classification algorithms may provide very good to excellent predictive accuracy for embryonic T21. These results support the further validation of PCF RNA as a low-cost T21 screening tool at 11–13 weeks of pregnancy.

## 6. Patents

The information presented is part of patent filings in the United States (U.S. Application No. 17/203,534) and in the European Community (PCT/US22/19680).

## Figures and Tables

**Table 1:** Biographical information about discovery cohort.

	T21 (n = 10) Mean (SD)	Range	Normal (n = 10) Mean (SD)	Range	T21 *p*-Value *	Race and Ethnicity *p*-Value *
GA (week)	12.9 (0.6)	(11.9–13.9)	12.7 (0.5)	(12.1–13.4)	NS	NS
MA (years)	37.3 (4.1)	(27.2–42.0)	33.7 (5.0)	(24.6–40.0)	*p* = 0.098 **	NS ***
Height (cm)	163.6 (4.9)	(157.5–170.2)	165.1 (4.4)	(157.5–171.0)	NS	NS
Weight (kg)	70.7 (9.9)	(60.0–83.5)	69.8 (18.7)	(50.00–115.0)	NS ***	NS ***

* Two-tailed *p*-value from Student’s *t*-test, unless indicated otherwise. ** Ho: MA-T21 > than MA-normal, one-tailed testing would indicate *p* < 0.05. *** Mann–Whitney rank-sum test.

**Table 2:** RNAs used in confirmation testing. (**a**) Coding mRNA; (**b**) Noncoding small RNA.

(a)				
Group 1. Coding mRNA Gene Name	*p*-Value	Ref Sequence	Chromosome Origin	Up or Down Regulation
SORBS2-Hs00243432_m1	<0.01	NM_001145671	4	Down
SORBS2-Hs01125202_m1	<0.01	NM_001145671	4	Up
DSCAM-Hs00242097_m1	<0.01	NM_020693	11	Down
NEK9-Hs00929602_m1	<0.01	NM_033116	14	Down
NEK9-Hs00929594_m1	<0.01	NM_033116	14	Up
ABCC1-Hs01561504_m1	<0.01	NM_004996	16	Up
FAM20A-Hs01034071_m1	<0.01	NR_027751	17	Down
FAM20A-Hs01034070_m1	<0.01	NR_027751	17	Down
RASGRP4-Hs01073179_m1	<0.01	NM_170604	19	Down
TMPRSS2-ERG fusion gene	<0.01	NM_002772	21	Down
ATP5O-Hs04272738_m1	<0.01	NM_001697.3	21	Down
ICOSLG-Hs00391287_m1	<0.01	NM_015259	21	Down
DOP1B-Hs01123288_m1	<0.01	NM_005128	21	Down
DOP1B-Hs01123267_g1	<0.01	NM_005128	21	Down
C21orf33-Hs01105802_g1	<0.01	NM_004649	21	Down
ADAMTS5-Hs04272736_s1	<0.01	NM_007038	21	Down
CXADR-Hs04194411_s1	<0.01	NM_001338	21	Down
UBASH3A-Hs00955168_m1	<0.01	NM_001001895.3	21	Down
CHODL-Hs01070471_m1	<0.01	NM_024944.3	21	Down
PKNOX1-Hs01007098_m1	<0.01	NM_004571	21	Down
PKNOX1-Hs01007097_m1	<0.01	NM_001286258	21	Down
PKNOX1-Hs00231814_m1	<0.01		21	Down
SLC19A1-Hs00953341_m1	<0.01	NM_194255	21	Down
PRDM15-Hs00411318_m1	<0.01	NM_022115	21	Down
COL6A1-Hs01095585_m1	<0.01	NM_001848	21	Down
ABCG1-Hs01555191_m1	<0.01	NM_016818	21	Down
GART-Hs00531926_m1	<0.01	NM_000819	21	Down
ERG-Hs01573964_m1	<0.01	NM_004449	21	Up
NCAM2-Hs01562292_m1	<0.01	NM_004540.5	21	Up
UBASH3A-Hs00955169_m1	<0.01	NM_018961.4	21	Up
PFKL-Hs01040525_m1	<0.01	NR_024108	21	Up
PKNOX1-Hs01007094_m1	<0.01	NM_001320694	21	Up
PKNOX1-Hs01007093_m1	<0.01		21	Up
PKNOX1-Hs01007092_m1	<0.01	NM_004571	21	Up
CYYR1-Hs00951849_m1	<0.01	NR_135472	21	Up
SLC19A1-Hs00953342_m1	<0.01	NM_194255	21	Up
**(b**)				
**Group 2. Noncoding Small RNA** **Gene Name**	***p*-Value**	**Type**	**Chromosome Origin**	**Up or Down Regulation**
hsa-mir-26b	<0.01	miRNA	2	Down
hsa-mir-216b	<0.01	miRNA	2	Up
hsa-mir-569 F1	<0.01	miRNA	3	Down
hsa-mir-548I	<0.01	miRNA	3	Down
ENSG00000212363	<0.01	snoRNA	5	Down
hsa-mir-581 F1	<0.01	miRNA	5	Up
HBII-276 F2	<0.01	CDBox	8	Up
hsa-let-7d F1	<0.01	miRNA	9	Up
ENSG00000201980	<0.01	snoRNA	11	Up
ENSG00000199282	<0.01	snoRNA	13	Down
hsa-mir-376a-2/1 F2	<0.01	miRNA	14	Down
ENSG00000199633 F2	<0.01	snoRNA	15	Up
ENSG00000199856 F1	<0.01	snoRNA	18	Down
hsa-mir-523	<0.01	miRNA	19	Down
ENSG00000207147 F2	<0.01	snoRNA	21	Up
ENSG00000202231	<0.01	snoRNA	X	Down
hsa-mir-98	<0.01	miRNA	X	Down
hsa-mir-450b	<0.01	miRNA	X	Down

**Table 3:** Biographical information about validation cohort.

	T21 (n = 50) Mean (SD)	Range	Normal (n = 948) Mean (SD)	Range	T21 *p*-Value *	Race/Ethnicity *p*-Value *
GA (week)	13.0 (0.7)	(11.3–14.1)	12.7 (0.6)	(11.2–14.1)	<0.001	NS
MA (years)	37.6 (4.4)	(26.4–46)	31.7 (5.6)	(18.1–45.1)	<0.001	<0.001
Height (cm)	164.6 (7.1)	(149.9–182.9)	164.5 (6.9)	(138.0–195.6)	NS	<0.001
Weight (kg)	68.1 (11.1)	(44.5–99.2)	66.6 (11.9)	(40.0–29.0)	NS	<0.001

* Two-tailed *p*-value from Mann–Whitney–Wilcoxon test.

**Table 4:** Differentially expressed RNAs and biographical variables used in machine learning.

Chromosome	Plate Position	Variables	NCBI Names	Mann–Whitney–Wilcoxon	Q-Values	Benjamini–Hochberg	Benjamini–Yekutieli	Holm	Hochberg	Hommel	Bonferroni
		MA		1.64 × 10^−12^	8.34 × 10^−11^	9.51 × 10^−11^	4.42 × 10^−10^	9.51 × 10^−11^	9.51 × 10^−11^	9.51 × 10^−11^	9.51 × 10^−11^
19	19	RASGRP4	NM_170604	2.49 × 10^−7^	6.33 × 10^−6^	7.21 × 10^−6^	3.35 × 10^−5^	1.42 × 10^−5^	1.42 × 10^−5^	1.42 × 10^−5^	1.44 × 10^−5^
2	10	hsa-mir-26b	miRNA miR-26b	2.16 × 10^−6^	3.52 × 10^−5^	4.01 × 10^−5^	1.86 × 10^−4^	1.21 × 10^−4^	1.21 × 10^−4^	1.19 × 10^−4^	1.25 × 10^−4^
21	38	UBASH3A	NM_018961.4	2.77 × 10^−6^	3.52 × 10^−5^	4.01 × 10^−5^	1.86 × 10^−4^	1.52 × 10^−4^	1.52 × 10^−4^	1.52 × 10^−4^	1.60 × 10^−4^
21	31	ICOSLG	NM_015259	7.77 × 10^−6^	7.91 × 10^−5^	9.01 × 10^−5^	4.19 × 10^−4^	4.20 × 10^−4^	4.20 × 10^−4^	4.20 × 10^−4^	4.51 × 10^−4^
11	9	ENSG00000201980	snoRNA SNORA62L4	6.24 × 10^−5^	5.29 × 10^−4^	6.03 × 10^−4^	2.80 × 10^−3^	3.31 × 10^−3^	3.31 × 10^−3^	3.31 × 10^−3^	3.62 × 10^−3^
		GA.w		2.51 × 10^−4^	1.68 × 10^−3^	1.92 × 10^−3^	8.92 × 10^−3^	1.30 × 10^−2^	1.30 × 10^−2^	1.23 × 10^−2^	1.45 × 10^−2^
21	52	COL6A1	NM_001848	2.85 × 10^−4^	1.68 × 10^−3^	1.92 × 10^−3^	8.92 × 10^−3^	1.45 × 10^−2^	1.45 × 10^−2^	1.40 × 10^−2^	1.65 × 10^−2^
21	37	NCAM2	NM_004540.5	2.98 × 10^−4^	1.68 × 10^−3^	1.92 × 10^−3^	8.92 × 10^−3^	1.49 × 10^−2^	1.49 × 10^−2^	1.46 × 10^−2^	1.73 × 10^−2^
14	22	NEK9	NM_033116	3.78 × 10^−4^	1.92 × 10^−3^	2.19 × 10^−3^	1.02 × 10^−2^	1.85 × 10^−2^	1.85 × 10^−2^	1.81 × 10^−2^	2.19 × 10^−2^
21	51	PRDM15	NM_022115	7.18 × 10^−4^	3.32 × 10^−3^	3.78 × 10^−3^	1.76 × 10^−2^	3.44 × 10^−2^	3.44 × 10^−2^	3.30 × 10^−2^	4.16 × 10^−2^
21	2	ENSG00000207147 F2	snoRNA SNORA51L12	1.41 × 10^−3^	5.96 × 10^−3^	6.80 × 10^−3^	3.16 × 10^−2^	6.64 × 10^−2^	6.64 × 10^−2^	6.32 × 10^−2^	8.19 × 10^−2^
21	27	TMPRSS2-ERG fusion gene	NM_002772	1.52 × 10^−3^	5.96 × 10^−3^	6.80 × 10^−3^	3.16 × 10^−2^	7.01 × 10^−2^	7.01 × 10^−2^	6.70 × 10^−2^	8.84 × 10^−2^
18	17	ENSG00000199856 F1	snoRNA SNODB852	2.03 × 10^−3^	7.39 × 10^−3^	8.42 × 10^−3^	3.91 × 10^−2^	9.15 × 10^−2^	9.15 × 10^−2^	8.95 × 10^−2^	1.18 × 10^−1^
21	33	DOP1B	NM_005128	3.08 × 10^−3^	1.04 × 10^−2^	1.19 × 10^−2^	5.53 × 10^−2^	1.36 × 10^−1^	1.36 × 10^−1^	1.32 × 10^−1^	1.79 × 10^−1^
21	26	SORBS2	NM_001145671	4.21 × 10^−3^	1.34 × 10^−2^	1.53 × 10^−2^	7.10 × 10^−2^	1.81 × 10^−1^	1.81 × 10^−1^	1.81 × 10^−1^	2.44 × 10^−1^
9	3	hsa-let-7d F1	miRNA let-7d	1.08 × 10^−2^	3.22 × 10^−2^	3.67 × 10^−2^	1.70 × 10^−1^	4.52 × 10^−1^	4.52 × 10^−1^	4.19 × 10^−1^	6.24 × 10^−1^
17	20	FAM20A	NR_027751	1.92 × 10^−2^	5.20 × 10^−2^	5.93 × 10^−2^	2.76 × 10^−1^	7.88 × 10^−1^	7.77 × 10^−1^	6.15 × 10^−1^	1.00 × 10^0^
21	41	CHODL	NM_024944.3	1.94 × 10^−2^	5.20 × 10^−2^	5.93 × 10^−2^	2.76 × 10^−1^	7.88 × 10^−1^	7.77 × 10^−1^	6.22 × 10^−1^	1.00 × 10^0^
X	12	hsa-mir-450b	miRNA miR-450b	2.79 × 10^−2^	7.10 × 10^−2^	8.10 × 10^−2^	3.76 × 10^−1^	1.00 × 10^0^	9.98 × 10^−1^	7.71 × 10^−1^	1.00 × 10^0^
17	21	FAM20A	NR_027751	3.41 × 10^−2^	8.25 × 10^−2^	9.41 × 10^−2^	4.37 × 10^−1^	1.00 × 10^0^	9.98 × 10^−1^	8.51 × 10^−1^	1.00 × 10^0^
21	34	C21orf33	NM_004649	5.11 × 10^−2^	1.18 × 10^−1^	1.35 × 10^−1^	6.26 × 10^−1^	1.00 × 10^0^	9.98 × 10^−1^	9.98 × 10^−1^	1.00 × 10^0^
11	29	DSCAM	NM_020693	5.46 × 10^−2^	1.21 × 10^−1^	1.38 × 10^−1^	6.40 × 10^−1^	1.00 × 10^0^	9.98 × 10^−1^	9.98 × 10^−1^	1.00 × 10^0^
21	36	CXADR	NM_001338	6.04 × 10^−2^	1.28 × 10^−1^	1.46 × 10^−1^	6.79 × 10^−1^	1.00 × 10^0^	9.98 × 10^−1^	9.98 × 10^−1^	1.00 × 10^0^
13	14	ENSG00000199282	snoRNA SNOFA9	6.63 × 10^−2^	1.35 × 10^−1^	1.54 × 10^−1^	7.14 × 10^−1^	1.00 × 10^0^	9.98 × 10^−1^	9.98 × 10^−1^	1.00 × 10^0^
21	30	ERG	NM_004449	8.44 × 10^−2^	1.65 × 10^−1^	1.88 × 10^−1^	8.75 × 10^−1^	1.00 × 10^0^	9.98 × 10^−1^	9.98 × 10^−1^	1.00 × 10^0^
3	4	hsa-mir-569 F1	miRNA miR-569	9.42 × 10^−2^	1.77 × 10^−1^	2.02 × 10^−1^	9.40 × 10^−1^	1.00 × 10^0^	9.98 × 10^−1^	9.98 × 10^−1^	1.00 × 10^0^
5	13	SORBS2	NM_001145671	1.09 × 10^−1^	1.91 × 10^−1^	2.18 × 10^−1^	1.00 × 10^0^	1.00 × 10^0^	9.98 × 10^−1^	9.98 × 10^−1^	1.00 × 10^0^
4	25	ENSG00000212363	snoRNA SNOFA40L2	1.09 × 10^−1^	1.91 × 10^−1^	2.18 × 10^−1^	1.00 × 10^0^	1.00 × 10^0^	9.98 × 10^−1^	9.98 × 10^−1^	1.00 × 10^0^
21	47	PKNOX1		1.20 × 10^−1^	2.04 × 10^−1^	2.33 × 10^−1^	1.00 × 10^0^	1.00 × 10^0^	9.98 × 10^−1^	9.98 × 10^−1^	1.00 × 10^0^
2	8	hsa-mir-216b	miRNA miR-216b	1.52 × 10^−1^	2.50 × 10^−1^	2.85 × 10^−1^	1.00 × 10^0^	1.00 × 10^0^	9.98 × 10^−1^	9.98 × 10^−1^	1.00 × 10^0^
21	32	DOP1B	NM_005128	1.63 × 10^−1^	2.59 × 10^−1^	2.95 × 10^−1^	1.00 × 10^0^	1.00 × 10^0^	9.98 × 10^−1^	9.98 × 10^−1^	1.00 × 10^0^
14	16	hsa-mir-376a-2/1 F2	miRNA miR-376a	1.86 × 10^−1^	2.86 × 10^−1^	3.26 × 10^−1^	1.00 × 10^0^	1.00 × 10^0^	9.98 × 10^−1^	9.98 × 10^−1^	1.00 × 10^0^
21	35	ADAMTS5	NM_007038	1.91 × 10^−1^	2.86 × 10^−1^	3.26 × 10^−1^	1.00 × 10^0^	1.00 × 10^0^	9.98 × 10^−1^	9.98 × 10^−1^	1.00 × 10^0^
		Weight		2.08 × 10^−1^	3.02 × 10^−1^	3.44 × 10^−1^	1.00 × 10^0^	1.00 × 10^0^	9.98 × 10^−1^	9.98 × 10^−1^	1.00 × 10^0^
5	11	hsa-mir-581 F1	miRNA miR-581	2.20 × 10^−1^	3.11 × 10^−1^	3.55 × 10^−1^	1.00 × 10^0^	1.00 × 10^0^	9.98 × 10^−1^	9.98 × 10^−1^	1.00 × 10^0^
21	46	PKNOX1	NM_004571	2.37 × 10^−1^	3.20 × 10^−1^	3.64 × 10^−1^	1.00 × 10^0^	1.00 × 10^0^	9.98 × 10^−1^	9.98 × 10^−1^	1.00 × 10^0^
21	44	PKNOX1	NM_001320694	2.39 × 10^−1^	3.20 × 10^−1^	3.64 × 10^−1^	1.00 × 10^0^	1.00 × 10^0^	9.98 × 10^−1^	9.98 × 10^−1^	1.00 × 10^0^
21	50	SLC19A1	NM_194255	2.97 × 10^−1^	3.81 × 10^−1^	4.34 × 10^−1^	1.00 × 10^0^	1.00 × 10^0^	9.98 × 10^−1^	9.98 × 10^−1^	1.00 × 10^0^
21	40	PFKL	NR_024108	2.99 × 10^−1^	3.81 × 10^−1^	4.34 × 10^−1^	1.00 × 10^0^	1.00 × 10^0^	9.98 × 10^−1^	9.98 × 10^−1^	1.00 × 10^0^
8	18	HBII-276 F2	SnoRNA HBII-276 CDBox	3.33 × 10^−1^	4.13 × 10^−1^	4.71 × 10^−1^	1.00 × 10^0^	1.00 × 10^0^	9.98 × 10^−1^	9.98 × 10^−1^	1.00 × 10^0^
15	1	ENSG00000199633 F2	snoRNA SNODB1383	3.62 × 10^−1^	4.38 × 10^−1^	5.00 × 10^−1^	1.00 × 10^0^	1.00 × 10^0^	9.98 × 10^−1^	9.98 × 10^−1^	1.00 × 10^0^
X	7	ENSG00000202231	snoRNA SNOFA9	3.74 × 10^−1^	4.43 × 10^−1^	5.05 × 10^−1^	1.00 × 10^0^	1.00 × 10^0^	9.98 × 10^−1^	9.98 × 10^−1^	1.00 × 10^0^
21	28	ATP5O	NM_001697.3	3.85 × 10^−1^	4.46 × 10^−1^	5.08 × 10^−1^	1.00 × 10^0^	1.00 × 10^0^	9.98 × 10^−1^	9.98 × 10^−1^	1.00 × 10^0^
21	49	SLC19A1	NM_194255	4.72 × 10^−1^	5.33 × 10^−1^	6.08 × 10^−1^	1.00 × 10^0^	1.00 × 10^0^	9.98 × 10^−1^	9.98 × 10^−1^	1.00 × 10^0^
4	24	ABCC1	NM_004996	5.60 × 10^−1^	6.20 × 10^−1^	7.06 × 10^−1^	1.00 × 10^0^	1.00 × 10^0^	9.98 × 10^−1^	9.98 × 10^−1^	1.00 × 10^0^
3	5	hsa-mir-548I	miRNA miR-548I	6.62 × 10^−1^	7.12 × 10^−1^	8.12 × 10^−1^	1.00 × 10^0^	1.00 × 10^0^	9.98 × 10^−1^	9.98 × 10^−1^	1.00 × 10^0^
21	45	PKNOX1		6.93 × 10^−1^	7.12 × 10^−1^	8.12 × 10^−1^	1.00 × 10^0^	1.00 × 10^0^	9.98 × 10^−1^	9.98 × 10^−1^	1.00 × 10^0^
21	48	CYYR1	NR_135472	6.99 × 10^−1^	7.12 × 10^−1^	8.12 × 10^−1^	1.00 × 10^0^	1.00 × 10^0^	9.98 × 10^−1^	9.98 × 10^−1^	1.00 × 10^0^
X	9	hsa-mir-98	miRNA miR-98	7.00 × 10^−1^	7.12 × 10^−1^	8.12 × 10^−1^	1.00 × 10^0^	1.00 × 10^0^	9.98 × 10^−1^	9.98 × 10^−1^	1.00 × 10^0^
21	42	PKNOX1	NM_004571	7.30 × 10^−1^	7.28 × 10^−1^	8.30 × 10^−1^	1.00 × 10^0^	1.00 × 10^0^	9.98 × 10^−1^	9.98 × 10^−1^	1.00 × 10^0^
21	54	GART	NM_000819	7.53 × 10^−1^	7.37 × 10^−1^	8.40 × 10^−1^	1.00 × 10^0^	1.00 × 10^0^	9.98 × 10^−1^	9.98 × 10^−1^	1.00 × 10^0^
14	23	NEK9	NM_033116	8.48 × 10^−1^	8.14 × 10^−1^	9.28 × 10^−1^	1.00 × 10^0^	1.00 × 10^0^	9.98 × 10^−1^	9.98 × 10^−1^	1.00 × 10^0^
19	15	hsa-mir-523	miRNA miR-523	8.96 × 10^−1^	8.44 × 10^−1^	9.62 × 10^−1^	1.00 × 10^0^	1.00 × 10^0^	9.98 × 10^−1^	9.98 × 10^−1^	1.00 × 10^0^
		Height		9.63 × 10^−1^	8.75 × 10^−1^	9.98 × 10^−1^	1.00 × 10^0^	1.00 × 10^0^	9.98 × 10^−1^	9.98 × 10^−1^	1.00 × 10^0^
21	43	PKNOX1	NM_001286258	9.72 × 10^−1^	8.75 × 10^−1^	9.98 × 10^−1^	1.00 × 10^0^	1.00 × 10^0^	9.98 × 10^−1^	9.98 × 10^−1^	1.00 × 10^0^
21	39	UBASH3A	NM_001001895.3	9.93 × 10^−1^	8.75 × 10^−1^	9.98 × 10^−1^	1.00 × 10^0^	1.00 × 10^0^	9.98 × 10^−1^	9.98 × 10^−1^	1.00 × 10^0^
21	53	ABCG1	NM_016818	9.98 × 10^−1^	8.75 × 10^−1^	9.98 × 10^−1^	1.00 × 10^0^	1.00 × 10^0^	9.98 × 10^−1^	9.98 × 10^−1^	1.00 × 10^0^

**Table 5:** Seven most important variables used in top-performing ML models.

GBM		70% Training					75% Training		
Up sample	Accuracy	1.000	Kappa	1.000	Up sample	Accuracy	0.992	Kappa	0.912
**Gene Name**	**Ref Sequence**	**Chromosome**	**Attribute usage**	**Weight**	**Gene Name**	**Ref Sequence**	**Chromosome**	**Attribute usage**	**Weight**
RASGRP4	NM_170604	19	166.77	0.193	hsa-mir-26b	miRNA	2	179.19	0.196
hsa-mir-26b	miRNA	2	131.32	0.152	RASGRP4	NM_170604	19	121.42	0.133
UBASH3A	NM_018961.4	21	91.68	0.106	MA			85.02	0.093
NCAM2	NM_004540.5	21	88.98	0.103	NCAM2	NM_004540.5	21	82.56	0.090
COL6A1	NM_001848	21	56.96	0.066	ENSG00000207147 F2	snoRNA	21	76.16	0.083
MA			51.68	0.060	UBASH3A	NM_018961.4	21	71.55	0.078
ICOSLG	NM_015259	21	50.45	0.058	ENSG00000199856 F1	snoRNA	18	52.92	0.058
**C5.0**		**80% training**					**70% training**		
Original	Accuracy	1.000	Kappa	1.000	Up sample	Accuracy	0.99	Kappa	0.8837
**Gene Name**	**Ref Sequence**	**Chromosome**	**Attribute usage**	**Weight**	**Gene Name**	**Ref Sequence**	**Chromosome**	**Attribute usage**	**Weight**
GART	mRNA/NM_000819	21	100.00	0.052	hsa-mir-98	miRNA	X	70.78	0.260
hsa-mir-26b	miRNA	2	100.00	0.052	hsa-mir-523	miRNA	19	69.05	0.253
hsa-mir-450b	miRNA	X	99.50	0.052	ENSG00000207147 F2	snoRNA	21	60.02	0.220
COL6A1	NM_001848	21	98.87	0.052	hsa-mir-569 F1	miRNA	3	50.98	0.187
ATP5O	NM_001697.3	21	98.62	0.051	hsa-mir-216b	miRNA	2	21.84	0.080
ENSG00000199633 F2	snoRNA	15	98.25	0.051					
DOP1B	NM_005128	21	98.12	0.051					
**RF**		**80% training**					**80% training**		
Up sample	Accuracy	0.995	Kappa	0.945	Original	Accuracy	0.995	Kappa	0.945
**Gene Name**	**Ref Sequence**	**Chromosome**	**Attribute usage**	**Weight**	**Gene Name**	**Ref Sequence**	**Chromosome**	**Attribute usage**	**Weight**
hsa-mir-26b	miRNA	2	31.29	0.085	GART	mRNA/NM_000819	21	4.01	0.105
RASGRP4	NM_170604	19	27.15	0.073	hsa-mir-26b	miRNA	2	2.73	0.071
MA			25.19	0.068	hsa-mir-450b	miRNA	X	2.61	0.068
ICOSLG	NM_015259	21	20.69	0.056	PKNOX1	NM_004571	21	2.15	0.056
UBASH3A	NM_018961.4	21	20.02	0.054	ENSG00000199282	snoRNA	13	2.10	0.055
DOP1B	NM_005128	21	19.74	0.053	hsa-mir-376a-2/1 F2	miRNA	14	2.03	0.053
FAM20A	NR_027751	17	17.87	0.048	ATP5O	NM_001697.3	21	1.89	0.049

## Data Availability

All data is available on request from the investigators and Rosetta Signaling Laboratory LLC.

## Supplementary Materials

The following are available online at https://www.mdpi.com/article/10.3390/diagnostics12061410/s1, Table S1: Characteristics of RNA isolated in Discovery and Confirmation; Table S2: Messenger RNAs results from Discovery. NOTE: Raw *p*-values, fold-change, and *p*-value following FDR correction are listed.; Table S3: Non-coding RNAs results from Discovery. NOTE: Raw *p*-values, fold-change, and *p*-value following false discovery rate (FDR) correction are listed; Table S4: A correlation matrix of RNA marker expression for embryonic T21; Table S5: Machine learning dataset.; Table S6: Top twelve most important variables used in ML algorithms.
