# Supplementary material for: Detection of Embryonic Trisomy 21 in the First Trimester Using Maternal Plasma Cell-Free RNA
Source: Diagnostics (Basel). 2022 Jun 7;12(6):1410. doi: 10.3390/diagnostics12061410 (PMC9221829; doi:10.3390/diagnostics12061410)
Supplement: Supplementary file 1 [file diagnostics-12-01410-s001.zip › diagnostics-1759311-supplementary.pdf]

supplemental Table S1. RNA Isolation

Discovery Sample ID	Comment	260/280	260/230	Conc. (ng/ul)	Total V.(ul)	Total RNA (ug)
28550 01/11/07	control White	1.69	6.94	430.5	40	17.22
83649 23/01/08	control Black	1.73	2.20	416.4	40	16.66
29303 19/08/08	control Black	1.81	10.72	385.6	40	15.42
73676 19/02/09	control Black	1.08	0.24	817.1	40	32.68
99114 19/03/09	control Black	1.74	3.05	258.2	40	10.33
98481 27/03/09	control Black	1.68	5.08	366.9	40	14.68
73836 03/04/09	control Black	1.51	0.22	704.2	40	28.17
99596 17/04/09	control Black	1.65	3.78	423.0	40	16.92
102119 11/06/09	control Black	1.46	0.19	595.8	40	23.83
24504 28/08/09	control Black	1.8	2.99	412.7	40	16.51
109596 09/02/10	control Black	1.51	0.22	527.1	40	21.08
36465 18/01/08	control White	1.41	0.52	1815.7	40	72.63
8922 20/03/08	control White	1.44	0.31	1053.1	40	42.12
43319 09/10/08	control White	1.46	0.34	1117.1	40	44.68
93634 21/10/08	control White	1.53	8.24	459.2	40	18.37
39721 24/10/08	control White	1.35	0.28	943.8	40	37.75
95561 07/10/09	control White	1.65	5.41	383.8	40	15.35
35175 06/04/09	control White	1.43	0.20	615.7	40	24.63
102804 03/08/09	control White	1.21	0.19	619.7	40	24.79
89748 25/01/10	control White	1.49	5.00	448.3	40	17.93
83648 23/01/08	T21 Black	1.91	0.96	244.2	40	9.77
91188 18/08/08	T21 Black	1.6	0.18	595.1	40	23.80
98157 19/02/09	T21 Black	1.08	0.22	733.8	45	33.02
23358 19/03/09	T21 Black	1.68	2.96	296.9	40	11.88
99229 27/03/09	T21 Black	1.84	1.48	268.1	40	10.72
99647 03/04/09	T21 Black	1.47	0.23	731.5	40	29.26
100067 17/04/09	T21 Black	1.92	1.29	240.1	40	9.60
57540 07/07/09	T21 Black	1.54	0.20	637.0	40	25.48
104852 28/08/09	T21 Black	1.79	0.27	858.8	40	34.35
109597 No date	T21 Black	1.51	0.26	751.0	40	30.04
80625 01/11/07	T21 White	1.32	0.17	540.6	40	21.62
83433 18/01/08	T21 White	1.57	0.47	1616.9	40	64.68
73341 20/03/08	T21 White	1.4	0.31	1044.7	40	41.79
69949 09/10/08	T21 White	1.4	0.21	619.4	45	27.87
93623 20/10/08	T21 White	1.37	5.19	364.2	40	14.57
93803 24/10/08	T21 White	1.08	4.35	451.8	40	18.07
96323 07/01/09	T21 White	1.63	5.14	334.1	40	13.36
42825 06/04/09	T21 White	1.48	0.30	982.8	40	39.31
103722 27/07/09	T21 White	1.4	3.06	355.3	40	14.21
109112 25/01/10	T21 White	1.68	3.93	394.4	40	15.78

	260/280	260/230	Conc. (ng/ul)	Total Vol. (ul)	Total RNA (ug)
ave	1.53	2.18	621.37	40.25	25.02
sd	0.21	2.63	349.63	1.10	14.03
count	40	40	40	40	40
min	1.08	0.17	240.10	40.00	9.60
max	1.92	10.72	1815.70	45.00	72.63

Validation Sample ID	260/280	260/230	Conc. (ng/ul)	Total V.(ul)	Total RNA (ug)
58053	1.72	2.92	403.90	20	8.08
49310	1.57	3.12	430.70	20	8.61
58100	1.65	2.84	388.70	20	7.77
58113	1.66	3.78	408.10	20	8.16
57381	1.60	3.34	431.80	20	8.64
29381	1.25	1.71	259.40	20	5.19
58150	1.28	1.58	243.00	20	4.86
58229	1.13	1.44	224.30	20	4.49
58263	1.50	1.01	179.20	20	3.58
44771	1.06	2.21	286.10	20	5.72
11302	1.71	2.74	337.60	20	6.75
58311	1.63	1.51	305.30	20	6.11
57928	1.74	1.78	313.60	20	6.27
58356	1.57	1.54	301.50	20	6.03
58362	1.54	3.25	386.70	20	7.73
58425	1.27	3.82	264.40	20	5.29
58432	1.32	4.33	304.90	20	6.10
23713	1.31	1.69	248.20	20	4.96
58457	1.34	3.12	246.00	20	4.92
57689	1.09	1.46	221.90	20	4.44
38143	1.45	1.62	297.50	20	5.95
30099	1.61	1.16	252.10	20	5.04
58564	1.48	1.66	269.80	20	5.40
27764	1.44	2.34	277.70	20	5.55
58709	1.38	2.15	274.60	20	5.49
46163	1.18	1.82	290.60	20	5.81
39251	1.26	2.84	328.50	20	6.57
28759	1.34	4.16	324.50	20	6.49
58873	1.38	3.99	264.50	20	5.29
58878	1.16	3.61	324.30	20	6.49
21747	1.26	2.34	354.40	20	7.09
59002	1.37	2.08	275.50	20	5.51
59079	1.41	5.95	298.30	20	5.97
59121	1.49	5.26	311.90	20	6.24
59122	1.44	1.61	324.60	20	6.49
59120	1.34	4.63	308.30	20	6.17
59209	1.32	1.41	285.90	20	5.72
59250	1.26	2.52	323.60	20	6.47
36165	1.27	4.77	335.30	20	6.71
59368	1.24	4.02	296.00	20	5.92
59398	1.38	1.53	235.10	20	4.70
59443	1.35	1.64	283.10	20	5.66
59474	1.11	6.36	461.50	20	9.23
59475	1.19	0.22	636.60	20	12.73
59483	1.36	4.50	379.70	20	7.59
59572	1.29	1.81	273.30	20	5.47
59698	1.37	2.17	313.30	20	6.27
59729	1.15	2.17	311.40	20	6.23

59738	1.29	3.27	262.10	20	5.24
59758	1.30	2.52	247.20	20	4.94
58875	1.35	0.75	193.80	20	3.88
41126	1.46	0.95	192.70	20	3.85
59845	1.58	0.91	200.10	20	4.00
59885	1.50	0.87	194.20	20	3.88
60080	1.47	1.72	259.30	20	5.19
60260	1.13	5.15	384.40	20	7.69
33900	1.36	3.19	293.80	20	5.88
60313	1.25	1.81	238.10	20	4.76
35030	1.30	1.50	234.30	20	4.69
60407	1.26	1.53	229.00	20	4.58
60475	1.40	1.56	222.20	20	4.44
60494	1.44	1.32	236.00	20	4.72
60498	1.49	1.01	212.40	20	4.25
60512	1.44	1.31	245.10	20	4.90
60534	1.49	1.55	275.50	20	5.51
60410	1.39	0.33	203.40	20	4.07
60731	1.07	1.33	248.30	20	4.97
60775	1.22	1.45	240.80	20	4.82
60815	1.55	1.11	218.30	20	4.37
60865	1.27	1.24	229.50	20	4.59
60882	1.29	0.63	167.20	20	3.34
60913	1.31	2.24	308.60	20	6.17
35254	1.22	0.75	268.50	20	5.37
60957	1.37	2.01	303.20	20	6.06
60958	1.15	3.61	364.80	20	7.30
60963	1.33	2.58	306.40	20	6.13
60987	1.20	3.34	302.70	20	6.05
61000	1.47	2.84	307.10	20	6.14
61022	1.20	3.00	287.50	20	5.75
61064	1.42	0.99	198.60	20	3.97
61089	1.00	2.07	310.50	20	6.21
61145	1.42	1.39	228.30	20	4.57
39492	1.37	1.18	232.80	20	4.66
61208	1.49	1.71	289.10	20	5.78
61266	1.52	1.79	282.00	20	5.64
61345	1.48	2.14	304.80	20	6.10
61484	1.30	1.47	266.50	20	5.33
61508	1.58	0.79	193.90	20	3.88
61529	1.50	1.76	269.70	20	5.39
61536	1.60	1.11	222.20	20	4.44
61584	1.32	2.20	335.00	20	6.70
61642	1.41	1.69	292.70	20	5.85
61726	1.38	1.64	283.00	20	5.66
61733	1.44	1.45	294.80	20	5.90
35903	1.41	0.83	219.80	20	4.40
60708	1.35	1.85	316.60	20	6.33
61381	1.14	3.73	365.20	20	7.30
62228	1.35	1.54	255.40	20	5.11
62286	1.51	1.05	275.70	20	5.51
62304	1.38	6.11	388.00	20	7.76
62722	1.60	0.38	1172.40	20	23.45

62804	1.38	0.25	754.80	20	15.10
24359	1.51	0.27	813.20	20	16.26
63095	1.43	3.42	420.40	20	8.41
63148	1.55	1.39	309.70	20	6.19
62495	1.48	2.79	332.20	20	6.64
61743	1.47	1.32	223.90	20	4.48
63211	1.49	0.90	184.40	20	3.69
62751	1.50	8.19	260.50	20	5.21
62789	1.48	0.20	531.10	20	10.62
62622	1.36	2.59	222.80	20	4.46
63502	1.30	6.70	249.00	20	4.98
63638	1.46	2.51	220.90	20	4.42
62158	1.37	240.84	277.70	20	5.55
62001	1.40	3.70	249.70	20	4.99
63770	1.16	21.81	306.90	20	6.14
61704	1.40	2.60	227.60	20	4.55
63895	1.34	8.15	269.80	20	5.40
63962	1.38	-13.61	327.20	20	6.54
61684	1.46	-20.76	305.20	20	6.10
63408	1.23	0.86	186.50	20	3.73
64024	1.04	0.88	182.70	20	3.65
64077	1.28	0.85	188.50	20	3.77
63729	1.13	2.01	277.50	20	5.55
63782	1.32	3.15	357.60	20	7.15
64313	1.50	0.66	167.50	20	3.35
62579	1.27	2.27	296.60	20	5.93
64635	1.26	1.13	219.90	20	4.40
57908	1.27	1.89	250.70	20	5.01
64146	1.37	2.82	358.40	20	7.17
64303	1.18	4.14	388.00	20	7.76
62563	1.30	5.36	343.40	20	6.87
64937	1.36	2.60	306.50	20	6.13
64984	1.25	5.17	337.20	20	6.74
65079	1.19	6.66	381.30	20	7.63
63818	1.11	5.02	350.80	20	7.02
65099	1.20	3.86	327.60	20	6.55
63532	1.29	2.49	290.60	20	5.81
64536	1.18	2.73	296.80	20	5.94
27468	1.22	4.30	316.60	20	6.33
48319	1.07	1.22	226.60	20	4.53
64550	1.11	1.26	241.20	20	4.82
64135	1.10	1.31	224.50	20	4.49
65416	1.44	1.45	222.30	20	4.45
64787	1.27	2.56	343.20	20	6.86
26225	1.14	1.37	243.90	20	4.88
63690	1.33	0.45	269.70	20	5.39
64190	1.15	2.26	307.70	20	6.15
65477	1.39	2.60	285.30	20	5.71
64756	1.25	2.53	345.00	20	6.90
64666	1.21	5.19	401.50	20	8.03
65672	1.29	1.81	302.20	20	6.04
65020	1.28	2.18	266.30	20	5.33
65863	1.35	1.26	260.90	20	5.22

65876	1.29	1.76	282.40	20	5.65
65671	1.30	2.60	330.20	20	6.60
65038	1.29	1.40	247.80	20	4.96
65048	1.46	0.89	212.50	20	4.25
64820	1.25	0.68	254.00	20	5.08
65670	1.01	3.38	372.30	20	7.45
64376	1.04	4.02	316.60	20	6.33
65336	1.20	2.31	244.20	20	4.88
65898	0.92	3.25	297.10	20	5.94
65833	1.28	3.10	278.30	20	5.57
66282	1.17	3.69	291.80	20	5.84
36508	1.17	7.36	365.10	20	7.30
65291	1.21	3.09	293.30	20	5.87
58248	1.20	6.46	386.10	20	7.72
66491	1.22	8.20	403.10	20	8.06
64091	1.07	4.92	375.40	20	7.51
65893	1.21	3.55	340.10	20	6.80
35040	1.05	0.18	499.90	20	10.00
66595	1.06	11.82	430.70	20	8.61
66065	1.03	3.88	332.80	20	6.66
65448	0.99	11.50	322.00	20	6.44
33531	1.15	6.65	348.70	20	6.97
66654	1.07	5.83	326.60	20	6.53
65046	1.12	11.60	439.00	20	8.78
65101	1.13	5.14	290.40	20	5.81
65338	1.27	3.69	322.10	20	6.44
66515	1.15	1.75	266.30	20	5.33
65480	1.25	1.42	220.90	20	4.42
65814	1.23	2.28	281.30	20	5.63
64576	1.11	1.91	261.50	20	5.23
66913	1.26	1.99	282.10	20	5.64
66918	1.20	4.78	341.80	20	6.84
31355	1.20	3.38	290.30	20	5.81
66722	1.40	2.85	286.40	20	5.73
65739	1.30	2.88	344.80	20	6.90
67430	1.46	1.52	259.40	20	5.19
36231	1.29	0.16	493.80	20	9.88
67093	1.36	0.19	536.60	20	10.73
40919	1.28	0.17	501.20	20	10.02
43598	1.01	0.18	539.20	20	10.78
67612	1.23	4.54	475.60	20	9.51
67636	1.29	0.17	493.00	20	9.86
67743	1.61	0.16	479.40	20	9.59
67530	1.37	0.17	519.00	20	10.38
66017	1.44	5.10	370.30	20	7.41
66215	1.30	3.89	381.60	20	7.63
67647	0.91	2.71	354.50	20	7.09
11875	1.10	0.65	281.40	20	5.63
67919	1.29	0.59	202.80	20	4.06
44228	1.17	1.13	247.80	20	4.96
66301	1.19	2.02	302.10	20	6.04
38657	1.13	1.35	249.50	20	4.99
5500	1.24	1.12	238.50	20	4.77

67497	1.36	1.89	321.10	20	6.42
66545	1.37	1.16	227.90	20	4.56
38264	1.36	1.75	310.80	20	6.22
67534	1.37	3.32	408.80	20	8.18
66159	1.25	2.34	399.20	20	7.98
68155	1.29	4.60	430.20	20	8.60
43351	1.43	2.35	367.80	20	7.36
68572	1.42	1.80	344.60	20	6.89
67470	1.46	1.93	345.90	20	6.92
67466	1.47	1.53	352.50	20	7.05
68858	1.35	2.55	380.20	20	7.60
42135	1.23	3.56	418.70	20	8.37
67506	1.39	4.74	455.70	20	9.11
67940	1.33	0.48	330.00	20	6.60
39877	1.21	0.89	190.40	20	3.81
28236	1.04	0.87	193.50	20	3.87
67962	1.37	1.14	223.10	20	4.46
68952	1.33	0.23	647.60	20	12.95
68410	1.09	0.23	690.20	20	13.80
68958	0.82	1.91	287.80	20	5.76
68969	0.92	1.46	260.10	20	5.20
68543	1.17	1.05	229.70	20	4.59
47907	0.87	0.87	206.10	20	4.12
68983	1.44	3.62	458.00	20	9.16
68130	1.36	0.19	550.60	20	11.01
66969	1.34	6.92	463.70	20	9.27
67249	1.46	0.17	499.80	20	10.00
67314	1.16	0.22	643.60	20	12.87
69171	1.30	0.19	560.90	20	11.22
67876	1.22	8.03	395.30	20	7.91
68069	1.08	6.45	452.80	20	9.06
68886	1.38	5.04	424.80	20	8.50
68409	1.27	0.17	490.80	20	9.82
67967	1.02	1.06	226.80	20	4.54
69238	1.39	3.29	338.90	20	6.78
68744	1.17	1.88	281.90	20	5.64
68629	1.17	1.35	244.80	20	4.90
69490	1.22	0.41	309.40	20	6.19
68134	1.53	1.48	308.40	20	6.17
68770	1.46	1.46	326.00	20	6.52
22693	1.50	1.64	253.30	20	5.07
31966	1.44	2.86	378.00	20	7.56
68372	1.24	3.61	437.20	20	8.74
68624	1.39	2.64	413.70	20	8.27
68061	1.39	2.57	432.40	20	8.65
34889	1.29	2.97	429.00	20	8.58
32494	1.26	0.18	493.10	20	9.86
69495	1.36	0.18	497.80	20	9.96
69147	1.45	3.27	403.50	20	8.07
68406	1.46	3.08	389.80	20	7.80
68665	1.41	4.80	384.20	20	7.68
69816	1.30	2.42	379.40	20	7.59
69845	1.32	3.01	389.90	20	7.80

68663	1.25	0.17	495.20	20	9.90
68190	1.72	0.49	1570.50	20	31.41
188	1.52	0.43	1377.70	20	27.55
70006	1.62	0.61	2000.80	20	40.02
69079	1.29	0.20	597.90	20	11.96
50986	1.40	1.27	4295.80	20	85.92
70035	1.44	0.43	1156.80	20	23.14
68415	1.57	0.55	1780.30	20	35.61
44171	1.47	0.46	1449.90	20	29.00
68911	1.54	0.55	1789.40	20	35.79
68697	1.18	0.23	710.60	20	14.21
69162	1.19	8.73	408.80	20	8.18
69189	1.25	0.31	968.40	20	19.37
70022	1.29	0.35	1123.80	20	22.48
40856	1.25	0.23	714.60	20	14.29
70696	1.30	0.17	521.20	20	10.42
68763	1.32	0.26	746.50	20	14.93
69492	1.23	0.29	914.10	20	18.28
27742	1.20	0.28	882.60	20	17.65
70427	1.26	0.18	554.60	20	11.09
71070	1.31	0.18	514.80	20	10.30
69789	1.24	0.19	565.70	20	11.31
24343	1.19	0.19	535.10	20	10.70
70384	1.29	0.19	522.20	20	10.44
44708	1.12	0.20	568.60	20	11.37
70409	1.11	0.24	671.00	20	13.42
71193	1.22	0.17	495.50	20	9.91
70155	1.23	8.17	408.10	20	8.16
70208	1.36	4.83	429.00	20	8.58
70562	1.28	5.56	402.30	20	8.05
33580	1.11	0.19	582.60	20	11.65
71387	1.29	0.41	706.40	20	14.13
70152	1.43	6.25	436.20	20	8.72
44190	1.15	0.25	768.50	20	15.37
46137	1.43	0.51	1668.80	20	33.38
21866	1.34	0.39	1238.40	20	24.77
71102	1.24	0.25	759.70	20	15.19
70470	1.39	0.47	1468.10	20	29.36
70501	1.28	0.25	765.50	20	15.31
71730	1.27	0.26	797.00	20	15.94
45635	1.22	3.74	439.00	20	8.78
78920	1.09	3.92	456.00	20	9.12
79192	1.29	2.90	444.50	20	8.89
79240	1.20	3.04	398.80	20	7.98
49147	0.89	0.20	575.50	20	11.51
79682	1.36	3.15	364.50	20	7.29
79896	1.38	3.62	416.30	20	8.33
79886	1.03	4.42	463.50	20	9.27
80129	1.33	2.68	388.90	20	7.78
80188	1.15	4.32	421.40	20	8.43
80404	1.37	0.59	1943.40	20	38.87
80637	1.61	0.79	2583.70	20	51.67
80625	1.32	0.41	1302.50	20	26.05

80647	1.46	0.56	1842.00	20	36.84
52409	1.64	0.82	2688.00	20	53.76
80882	1.41	0.67	2178.10	20	43.56
80944	1.37	0.36	1133.70	20	22.67
80997	1.25	0.31	974.00	20	19.48
81111	1.18	0.24	727.50	20	14.55
81117	1.28	0.23	683.60	20	13.67
81201	1.14	0.29	487.70	20	9.75
81256	1.27	4.44	456.40	20	9.13
81274	1.24	4.31	439.90	20	8.80
80717	1.09	4.93	475.90	20	9.52
50380	1.22	5.48	467.30	20	9.35
81998	1.17	3.92	435.90	20	8.72
82693	0.98	0.16	494.90	20	9.90
77098	1.23	4.48	448.60	20	8.97
83115	1.16	0.18	544.00	20	10.88
49852	1.07	0.16	487.80	20	9.76
83375	1.30	0.19	546.50	20	10.93
83384	1.39	0.36	1120.90	20	22.42
83432	1.76	0.74	2409.90	20	48.20
83435	1.77	0.95	3047.80	20	60.96
83455	1.78	0.92	2982.60	20	59.65
53464	1.31	0.18	549.70	20	10.99
82392	1.28	0.17	513.60	20	10.27
51182	1.32	0.32	992.90	20	19.86
83980	1.26	0.30	936.60	20	18.73
55810	1.30	0.24	735.30	20	14.71
84107	1.34	4.62	432.00	20	8.64
3448	1.28	0.19	577.80	20	11.56
84247	1.26	0.19	570.10	20	11.40
84333	1.32	0.18	536.20	20	10.72
84493	1.40	5.45	462.90	20	9.26
84620	1.20	0.21	637.60	20	12.75
22693	1.28	0.17	503.50	20	10.07
82817	1.15	0.18	536.20	20	10.72
84841	1.33	0.29	868.00	20	17.36
84897	1.11	0.18	527.30	20	10.55
85108	1.41	0.29	922.60	20	18.45
84558	1.50	0.53	1713.80	20	34.28
85241	1.88	0.11	3721.30	20	74.43
85271	1.42	0.26	818.80	20	16.38
61484	1.43	0.64	2104.80	20	42.10
85395	1.38	0.27	850.70	20	17.01
56092	1.35	0.48	1553.90	20	31.08
86026	1.41	0.23	699.70	20	13.99
85585	1.47	0.22	694.10	20	13.88
59483	1.43	0.28	879.10	20	17.58
86274	1.32	0.23	713.50	20	14.27
86329	1.32	0.19	559.80	20	11.20
55558	1.19	0.18	547.00	20	10.94
46011	1.35	4.92	428.30	20	8.57
68061	1.16	0.24	762.90	20	15.26
86707	1.19	0.21	644.00	20	12.88

86757	1.36	0.18	538.10	20	10.76
87204	1.35	0.21	624.30	20	12.49
38121	1.43	5.39	446.90	20	8.94
87434	1.30	0.18	559.70	20	11.19
63081	1.55	0.54	1759.00	20	35.18
87614	1.33	0.30	925.40	20	18.51
2194	1.47	6.18	428.20	20	8.56
87711	1.26	0.21	648.20	20	12.96
33810	1.33	0.25	783.60	20	15.67
87842	1.40	0.22	684.80	20	13.70
44434	1.44	0.34	1082.50	20	21.65
87886	1.34	0.24	738.80	20	14.78
88055	1.36	0.22	647.80	20	12.96
87475	1.30	0.34	901.10	20	18.02
88523	1.31	0.25	755.40	20	15.11
66969	1.27	0.26	815.20	20	16.30
53582	1.18	0.24	754.10	20	15.08
34305	1.22	0.27	830.90	20	16.62
89113	1.40	0.18	544.70	20	10.89
80882	1.22	0.23	716.40	20	14.33
90045	1.24	0.27	827.40	20	16.55
90092	1.35	0.24	741.90	20	14.84
90181	1.28	0.37	1154.20	20	23.08
26686	1.29	0.20	618.40	20	12.37
90650	1.48	0.26	797.60	20	15.95
50570	1.20	0.25	770.60	20	15.41
90883	1.38	0.42	1313.60	20	26.27
40221	1.41	0.41	1316.50	20	26.33
90985	1.31	0.25	748.30	20	14.97
90989	1.37	0.21	627.00	20	12.54
91146	1.35	0.21	636.20	20	12.72
19918	1.30	0.18	546.90	20	10.94
71051	1.35	0.27	827.20	20	16.54
58263	1.21	0.21	642.80	20	12.86
91318	1.30	0.25	770.50	20	15.41
91381	1.26	0.29	908.00	20	18.16
91429	1.32	0.23	721.60	20	14.43
91461	1.34	0.23	690.60	20	13.81
91451	1.30	0.28	860.50	20	17.21
90114	1.22	0.25	766.60	20	15.33
60398	1.34	6.61	473.10	20	9.46
91645	1.30	0.21	643.20	20	12.86
35471	1.34	0.17	502.60	20	10.05
91436	1.27	0.18	547.90	20	10.96
91886	1.37	0.33	1036.50	20	20.73
92090	1.46	0.54	1717.40	20	34.35
92303	1.47	0.54	1746.70	20	34.93
92376	1.30	0.39	1214.40	20	24.29
92629	1.86	1.11	3698.10	20	73.96
92633	1.46	0.55	1747.60	20	34.95
92670	1.38	0.50	1612.20	20	32.24
92688	1.55	0.51	1634.30	20	32.69
92773	1.56	0.77	2511.70	20	50.23

92770	1.23	0.35	1084.80	20	21.70
92489	1.22	0.22	654.00	20	13.08
93648	1.30	0.17	517.50	20	10.35
93714	1.22	0.24	732.80	20	14.66
47501	1.29	0.17	503.90	20	10.08
93173	1.30	0.29	885.70	20	17.71
93882	1.28	0.23	713.80	20	14.28
93949	1.35	0.28	874.10	20	17.48
93948	1.35	0.26	809.60	20	16.19
94027	1.28	0.29	874.40	20	17.49
94196	1.28	0.24	738.10	20	14.76
32137	1.21	0.95	224.20	20	4.48
61904	0.91	2.44	368.70	20	7.37
94479	1.29	0.99	170.40	20	3.41
94607	1.41	3.97	284.60	20	5.69
94867	1.45	1.07	263.70	20	5.27
95008	1.30	6.15	364.90	20	7.30
95125	1.00	1.83	239.90	20	4.80
95190	1.18	0.90	184.20	20	3.68
10723	1.49	3.20	254.00	20	5.08
95480	1.25	1.13	197.60	20	3.95
95539	1.35	3.30	396.70	20	7.93
95536	1.04	6.03	443.90	20	8.88
95588	1.41	2.07	338.80	20	6.78
27381	1.13	2.18	349.90	20	7.00
95814	1.49	1.39	279.80	20	5.60
55643	1.06	5.25	464.80	20	9.30
71978	1.52	4.29	300.20	20	6.00
94619	1.61	2.27	341.40	20	6.83
96188	1.42	1.32	223.50	20	4.47
20451	1.09	2.43	338.90	20	6.78
96489	1.38	0.22	671.40	20	13.43
96481	1.38	0.20	620.00	20	12.40
96501	1.41	0.31	967.60	20	19.35
96487	1.29	0.24	757.50	20	15.15
96536	1.34	0.25	780.90	20	15.62
77070	1.52	0.23	714.80	20	14.30
95478	1.40	0.22	674.70	20	13.49
96687	1.41	0.29	891.20	20	17.82
96700	1.40	0.25	782.20	20	15.64
96723	1.57	0.19	567.20	20	11.34
96790	1.25	0.19	566.00	20	11.32
96759	1.31	0.19	578.50	20	11.57
73894	1.05	0.24	715.30	20	14.31
96756	1.16	0.20	586.50	20	11.73
97660	1.19	0.18	496.60	20	9.93
73914	1.02	0.19	568.20	20	11.36
97766	1.20	4.45	383.80	20	7.68
97874	1.30	3.57	387.50	20	7.75
83600	1.19	0.18	533.80	20	10.68
97970	1.42	5.46	448.10	20	8.96
98123	1.54	0.38	1167.40	20	23.35
98160	1.31	0.23	686.90	20	13.74

98409	1.63	0.56	1788.40	20	35.77
98408	1.28	0.20	598.80	20	11.98
98456	1.49	0.44	1300.80	20	26.02
36483	1.38	0.18	541.70	20	10.83
98462	1.28	0.26	812.30	20	16.25
98467	1.28	0.52	509.50	20	10.19
98670	1.38	0.26	792.60	20	15.85
98744	1.37	0.24	718.60	20	14.37
98749	1.10	0.17	501.80	20	10.04
98767	1.11	0.19	540.10	20	10.80
98790	1.25	0.17	499.10	20	9.98
98434	1.23	0.18	504.60	20	10.09
98885	1.37	0.22	653.90	20	13.08
98920	1.18	0.17	502.20	20	10.04
98938/9893[illegible]	1.35	2.65	390.20	20	7.80
98980	1.17	0.19	588.90	20	11.78
99121	1.18	1.95	334.90	20	6.70
23358	1.26	4.31	449.80	20	9.00
68406	1.32	0.36	577.90	20	11.56
99354	1.29	0.25	764.40	20	15.29
99359	1.77	0.94	3078.90	20	61.58
75228	1.42	0.53	1691.20	20	33.82
98483	1.85	1.06	3490.50	20	69.81
99525	1.36	0.60	1963.60	20	39.27
35175	1.42	0.48	1510.70	20	30.21
99798	1.45	0.41	1297.40	20	25.95
99925	1.34	0.33	1037.60	20	20.75
99571	1.16	0.32	1010.00	20	20.20
99977	1.43	5.75	373.30	20	7.47
100310	1.42	4.51	452.10	20	9.04
100401	1.18	7.39	463.00	20	9.26
100543	1.48	4.42	355.80	20	7.12
100653	1.08	7.79	423.10	20	8.46
100681	1.42	3.72	329.40	20	6.59
100981	1.49	3.79	348.20	20	6.96
100980	1.10	4.31	396.60	20	7.93
76612	1.36	2.19	304.40	20	6.09
101076	1.16	2.26	298.30	20	5.97
101103	1.30	0.21	610.60	20	12.21
61208	1.35	0.29	911.50	20	18.23
74366	1.26	0.25	762.70	20	15.25
101268	1.22	0.25	767.00	20	15.34
5090	1.28	0.21	647.70	20	12.95
101340	1.23	0.29	563.20	20	11.26
101362	1.20	0.23	704.40	20	14.09
101380	1.23	0.26	787.50	20	15.75
101412	1.22	0.21	623.40	20	12.47
100294	1.31	0.20	594.60	20	11.89
101163	1.38	3.51	390.00	20	7.80
40277	1.13	0.19	578.40	20	11.57
62471	1.37	5.49	466.30	20	9.33
101938	1.11	0.19	578.10	20	11.56
102165	1.15	0.18	534.20	20	10.68

102383	1.14	0.18	517.60	20	10.35
102451	1.62	5.16	421.30	20	8.43
102520	1.43	3.67	395.00	20	7.90
46444	1.01	4.22	359.20	20	7.18
102683	1.30	4.11	373.10	20	7.46
102085	1.13	2.41	378.80	20	7.58
102945	1.32	2.54	424.90	20	8.50
103072	1.52	2.57	403.10	20	8.06
103114	1.27	3.84	436.00	20	8.72
69664	1.26	6.58	455.60	20	9.11
80438	1.14	2.12	292.80	20	5.86
103474	1.38	2.09	350.10	20	7.00
68849	1.29	2.06	316.90	20	6.34
16662	1.23	1.85	268.90	20	5.38
103592	1.34	2.45	365.30	20	7.31
103654	1.49	6.16	405.10	20	8.10
103425	1.17	0.19	519.70	20	10.39
103777	1.33	5.56	383.10	20	7.66
103979	1.19	0.82	595.80	20	11.92
104147	1.40	8.86	403.00	20	8.06
104181	1.19	7.74	401.70	20	8.03
41348	1.28	6.00	417.20	20	8.34
68452	1.30	6.41	453.40	20	9.07
104247	1.39	5.52	441.40	20	8.83
104411	1.18	4.95	393.00	20	7.86
104415	1.12	2.45	351.50	20	7.03
104537	1.34	0.20	579.90	20	11.60
104572	1.31	0.24	701.30	20	14.03
65070	1.04	0.74	576.00	20	11.52
105044	1.39	0.67	472.90	20	9.46
16260	1.25	2.69	334.50	20	6.69
105103	1.30	0.20	584.90	20	11.70
105234	1.33	2.31	356.00	20	7.12
105316	1.37	3.00	411.00	20	8.22
105381	1.41	0.27	763.20	20	15.26
105710	1.29	4.02	401.90	20	8.04
105277	1.14	5.58	405.90	20	8.12
105932	1.29	3.95	382.20	20	7.64
105993	1.36	7.17	468.50	20	9.37
34305	1.04	0.22	618.00	20	12.36
106246	1.25	0.70	455.10	20	9.10
73004	1.08	0.17	513.60	20	10.27
106375	1.08	0.21	612.90	20	12.26
106338	1.20	6.59	440.40	20	8.81
106500	1.31	5.49	374.00	20	7.48
106562	1.54	0.27	808.90	20	16.18
106680	1.40	3.90	416.30	20	8.33
106763	1.30	7.00	412.00	20	8.24
106961	1.35	5.58	362.60	20	7.25
106970	1.17	6.95	416.70	20	8.33
40909	1.20	0.20	572.00	20	11.44
64937	1.46	5.53	382.90	20	7.66
63709	1.52	0.26	735.50	20	14.71

11302	1.35	0.20	587.50	20	11.75
89295	1.25	0.18	516.60	20	10.33
73662	1.19	0.17	514.30	20	10.29
107786	1.28	10.05	387.00	20	7.74
107796	1.26	0.18	531.20	20	10.62
20942	1.32	16.85	352.60	20	7.05
108043	1.38	14.56	372.90	20	7.46
66820	1.38	25.27	422.50	20	8.45
108152	1.16	7.74	372.90	20	7.46
108008	1.26	10.36	354.70	20	7.09
108299	1.20	6.45	339.00	20	6.78
108316	1.19	9.91	377.70	20	7.55
61556	1.32	0.25	738.40	20	14.77
108336	1.21	0.20	583.90	20	11.68
108408	1.76	0.58	1859.30	20	37.19
63081	1.50	0.63	2047.60	20	40.95
23051	1.87	0.81	2630.20	20	52.60
108581	1.21	0.20	615.60	20	12.31
108542	1.22	0.25	745.20	20	14.90
108750	1.27	0.31	968.80	20	19.38
108764	1.30	0.23	685.60	20	13.71
108825	1.33	0.34	1076.60	20	21.53
78033	1.52	3.43	328.30	20	6.57
108889	1.33	5.66	448.00	20	8.96
108952	0.96	0.18	540.00	20	10.80
69364	1.18	0.17	503.70	20	10.07
109048	1.37	7.14	388.10	20	7.76
109189	1.25	5.28	346.90	20	6.94
80104	1.33	2.49	277.90	20	5.56
109239	1.45	4.50	338.60	20	6.77
109271	1.14	4.01	346.30	20	6.93
14394	1.16	5.68	352.30	20	7.05
109539	1.32	2.47	369.10	20	7.38
36573	1.26	3.20	368.00	20	7.36
109564	1.27	0.31	933.50	20	18.67
109730	1.34	0.35	1049.70	20	20.99
109946	1.26	4.48	413.50	20	8.27
109988	1.29	0.30	910.80	20	18.22
110029	1.39	4.97	467.80	20	9.36
110122	1.30	3.49	433.00	20	8.66
110136	1.36	0.46	287.50	20	5.75
110139	1.32	2.20	329.60	20	6.59
80684	1.27	6.35	477.00	20	9.54
110232	1.34	3.19	336.10	20	6.72
110290	1.23	4.81	360.00	20	7.20
22061	1.31	3.90	402.90	20	8.06
29004	1.02	6.64	456.70	20	9.13
65863	1.36	6.14	424.00	20	8.48
110384	1.12	4.53	386.60	20	7.73
110383	1.15	3.86	379.60	20	7.59
110405	1.37	4.28	321.40	20	6.43
35040	1.14	3.88	366.00	20	7.32
49157	1.22	0.29	885.40	20	17.71

110559	1.31	0.26	783.00	20	15.66
110593	1.55	0.44	1412.00	20	28.24
89164	1.25	0.28	857.90	20	17.16
81405	1.67	0.58	1841.50	20	36.83
109559	1.35	0.32	982.70	20	19.65
108805	1.65	0.72	2310.40	20	46.21
110691	1.37	0.37	1153.40	20	23.07
110696	1.40	0.42	1305.10	20	26.10
85837	1.23	0.27	815.20	20	16.30
88783	1.31	6.24	430.10	20	8.60
66157	1.25	7.44	459.10	20	9.18
37148	1.31	5.15	447.20	20	8.94
109486	1.38	5.33	429.10	20	8.58
110965	1.19	0.20	606.70	20	12.13
110898	1.24	0.22	664.20	20	13.28
111081	1.14	6.17	436.00	20	8.72
111085	1.40	6.13	406.30	20	8.13
111184	1.12	4.82	458.90	20	9.18
75802	1.24	5.78	400.10	20	8.00
111285	1.79	0.78	2518.90	20	50.38
110065	1.22	0.22	677.80	20	13.56
111337	1.70	0.71	2314.60	20	46.29
106217	1.23	0.28	860.00	20	17.20
111352	1.17	0.28	852.00	20	17.04
111372	1.27	0.24	709.50	20	14.19
111393	1.37	0.35	1068.30	20	21.37
89504	1.48	0.56	1797.60	20	35.95
110012	1.41	0.42	1296.50	20	25.93
111486	1.41	0.31	925.20	20	18.50
80125	1.34	3.11	378.00	20	7.56
63742	1.16	0.17	501.10	20	10.02
55585	1.40	4.66	435.90	20	8.72
93949	1.30	4.93	368.60	20	7.37
68708	1.18	4.85	448.90	20	8.98
111759	1.12	4.70	455.70	20	9.11
111813	1.10	4.50	423.00	20	8.46
66918	1.19	3.50	332.40	20	6.65
111824	1.40	3.25	306.50	20	6.13
82746	1.33	4.20	360.80	20	7.22
89428	1.14	3.66	378.40	20	7.57
94536	1.24	0.18	531.90	20	10.64
79966	1.50	0.35	1069.10	20	21.38
85704	1.10	5.19	359.70	20	7.19
112033	1.52	4.74	473.40	20	9.47
112065	1.30	5.92	480.50	20	9.61
112121	1.28	0.27	793.90	20	15.88
112191	1.38	9.29	448.00	20	8.96
112263	1.45	0.32	965.30	20	19.31
112277	1.21	0.16	469.30	20	9.39
112301	1.17	6.50	432.10	20	8.64
112409	1.37	6.01	452.80	20	9.06
112030	1.09	0.16	496.90	20	9.94
112461	1.38	5.36	383.50	20	7.67

111786	1.34	4.38	370.10	20	7.40
112171	1.27	5.05	391.00	20	7.82
112607	1.08	5.21	383.10	20	7.66
112489	1.29	6.34	454.00	20	9.08
112685	1.14	7.32	442.60	20	8.85
111359	1.05	7.75	445.10	20	8.90
78470	1.67	1.26	4332.90	20	86.66
112699	1.85	1.09	3553.70	20	71.07
112545	1.91	1.11	3664.90	20	73.30
86682	1.63	0.86	2808.10	20	56.16
79633	1.51	1.30	4351.00	20	87.02
95715	1.45	1.30	4401.00	20	88.02
79809	1.71	1.24	4124.40	20	82.49
111857	1.63	1.12	3682.30	20	73.65
17034	1.62	1.26	4264.00	20	85.28
113047	1.68	0.97	3204.00	20	64.08
112550	1.30	4.24	380.20	20	7.60
113086	1.34	4.92	415.40	20	8.31
56277	0.99	0.17	510.80	20	10.22
112921	1.33	5.37	447.80	20	8.96
113265	1.36	4.22	371.40	20	7.43
112380	1.32	6.07	438.30	20	8.77
113685	1.37	5.09	415.20	20	8.30
113561	1.05	0.16	495.20	20	9.90
75614	1.07	6.93	472.20	20	9.44
110318	1.33	0.18	522.10	20	10.44
114143	1.33	0.38	1157.70	20	23.15
113069	1.21	0.26	758.80	20	15.18
113198	1.31	0.28	848.90	20	16.98
13584	1.24	0.37	1146.50	20	22.93
113978	1.28	0.23	680.20	20	13.60
113120	1.30	0.23	692.80	20	13.86
114221	1.44	0.23	685.90	20	13.72
114333	1.37	0.21	633.90	20	12.68
114352	1.85	0.85	2783.10	20	55.66
114450	1.82	0.85	2766.90	20	55.34
92727	1.29	6.67	467.80	20	9.36
90748	1.35	0.18	536.90	20	10.74
114506	1.36	0.19	564.00	20	11.28
114511	1.19	0.20	610.80	20	12.22
89546	1.21	0.20	605.70	20	12.11
114391	1.56	6.77	449.80	20	9.00
114673	1.46	5.10	419.30	20	8.39
114675	1.32	5.54	437.90	20	8.76
114686	1.41	8.75	462.80	20	9.26
83235	1.22	0.18	549.80	20	11.00
92943	1.66	0.56	1772.20	20	35.44
59447	1.45	0.41	1250.60	20	25.01
91381	1.39	0.35	1060.70	20	21.21
113847	1.43	0.20	601.70	20	12.03
113749	1.52	0.42	1217.50	20	24.35
115513	1.33	0.18	544.70	20	10.89
90989	1.36	0.21	582.40	20	11.65

115556	1.21	0.47	1444.70	20	28.89
24943	1.71	0.65	2070.90	20	41.42
94157	1.25	0.31	810.10	20	16.20
115733	1.21	0.19	588.00	20	11.76
114037	1.09	0.22	662.50	20	13.25
114878	1.17	0.21	621.80	20	12.44
114979	1.22	0.19	589.20	20	11.78
33319	1.13	0.21	635.80	20	12.72
115858	1.25	0.17	523.70	20	10.47
107679	1.14	0.18	619.70	20	12.39
115979	1.14	0.20	624.30	20	12.49
116052	1.14	0.19	578.50	20	11.57
61508	1.18	0.18	546.40	20	10.93
114678	1.36	0.27	827.70	20	16.55
116008	1.25	0.19	564.80	20	11.30
114775	1.32	0.32	978.20	20	19.56
115726	1.24	0.19	548.70	20	10.97
65989	1.41	0.19	552.30	20	11.05
114790	1.35	0.19	527.50	20	10.55
53102	1.22	0.19	565.90	20	11.32
116312	1.13	0.20	608.60	20	12.17
114857	1.13	0.18	530.10	20	10.60
115859	1.31	0.19	538.40	20	10.77
116374	1.23	0.18	527.50	20	10.55
116467	1.22	0.18	513.40	20	10.27
116468	1.23	0.18	548.90	20	10.98
116501	1.42	5.01	467.30	20	9.35
116518	1.35	4.52	400.60	20	8.01
116514	1.37	0.18	542.60	20	10.85
116521	1.07	4.73	476.50	20	9.53
35345	1.33	2.00	408.30	20	8.17
67940	1.41	3.62	410.60	20	8.21
115975	1.30	0.17	507.40	20	10.15
116581	1.55	0.26	772.20	20	15.44
116586	1.61	7.56	389.60	20	7.79
80990	1.72	4.48	357.50	20	7.15
115541	1.57	5.49	390.90	20	7.82
116616	1.71	6.78	458.40	20	9.17
115557	1.14	5.68	463.50	20	9.27
116619	1.70	3.71	390.50	20	7.81
116708	1.53	3.74	374.30	20	7.49
116121	1.63	7.68	456.40	20	9.13
19793	1.42	6.28	466.70	20	9.33
116996	1.26	0.22	671.20	20	13.42
117058	1.10	0.21	636.10	20	12.72
116342	1.36	0.21	654.40	20	13.09
118850	1.17	0.29	912.90	20	18.26
120163	1.33	0.23	714.60	20	14.29
124395	1.29	0.21	641.40	20	12.83
25572	1.19	0.26	800.90	20	16.02
112157	1.32	0.17	512.60	20	10.25
69238	1.44	0.18	550.90	20	11.02
131404	1.24	0.19	594.80	20	11.90

112497	1.73	0.52	1628.10	20	32.56
133623	1.51	0.67	2100.70	20	42.01
123985	1.79	0.70	2247.30	20	44.95
123092	1.32	0.35	1098.10	20	21.96
150208	1.72	0.64	2028.10	20	40.56
42154	1.31	0.34	1056.10	20	21.12
157555	1.85	0.80	2589.70	20	51.79
112473	1.62	0.53	1693.20	20	33.86
141508	1.81	0.78	2497.50	20	49.95
100549	1.28	0.38	1198.00	20	23.96
144985	1.29	0.19	565.20	20	11.30
94607	1.13	0.23	699.60	20	13.99
150148	1.10	0.24	732.00	20	14.64
84046	1.19	0.19	565.80	20	11.32
153694	1.31	0.27	839.60	20	16.79
166681	1.29	0.27	825.30	20	16.51
160605	1.18	0.21	626.90	20	12.54
161436	1.15	0.21	620.10	20	12.40
183744	1.24	0.21	629.20	20	12.58
223038	1.32	0.21	650.50	20	13.01
104513	1.88	0.78	2554.70	20	51.09
242142	1.48	0.24	720.20	20	14.40
263552	1.65	0.78	2524.80	20	50.50
263597	1.42	0.37	1154.20	20	23.08
196651	1.70	0.75	2421.40	20	48.43
118981	1.47	0.76	2474.10	20	49.48
175472	1.54	0.36	1130.00	20	22.60
96700	1.67	0.68	2220.50	20	44.41
216212	1.90	0.91	2964.30	20	59.29
3956	1.50	0.36	1127.40	20	22.55
267479	1.26	0.20	596.30	20	11.93
102101	1.32	0.22	644.10	20	12.88
215892	1.15	4.59	395.20	20	7.90
224033	1.23	0.23	689.10	20	13.78
63418	1.39	0.26	678.30	20	13.57
263660	1.15	7.73	446.40	20	8.93
57314	1.19	0.23	693.20	20	13.86
271652	1.25	0.20	577.10	20	11.54
268333	1.27	0.20	586.00	20	11.72
87682	1.34	0.20	610.40	20	12.21
256082	1.76	0.67	2132.00	20	42.64
170521	1.49	0.42	1336.00	20	26.72
234998	1.68	0.79	2585.00	20	51.70
293231	1.46	0.35	1099.50	20	21.99
206181	1.63	0.68	2160.10	20	43.20
263707	1.46	0.49	1543.30	20	30.87
256013	1.74	0.69	2220.20	20	44.40
292706	1.49	0.55	1747.90	20	34.96
315836	1.58	0.67	2156.30	20	43.13
278817	1.47	0.31	939.20	20	18.78
70425	1.41	0.20	576.50	20	11.53
267948	1.33	0.25	740.20	20	14.80
176326	1.33	0.28	823.70	20	16.47

268527	1.36	0.23	696.40	20	13.93
292311	1.27	0.24	696.10	20	13.92
278687	1.36	0.23	669.90	20	13.40
332353	1.23	0.25	745.00	20	14.90
85541	1.24	0.19	554.30	20	11.09
98499	1.14	0.23	679.80	20	13.60
278682	1.38	0.18	526.30	20	10.53
314532	1.66	0.63	2015.30	20	40.31
92670	1.46	0.38	1180.90	20	23.62
185662	1.60	0.53	1655.90	20	33.12
294451	1.38	0.42	1108.90	20	22.18
316216	1.50	0.34	1051.00	20	21.02
133896	1.41	0.32	974.30	20	19.49
322969	1.32	0.25	761.20	20	15.22
315328	1.49	0.34	1038.20	20	20.76
217338	1.77	0.60	1914.90	20	38.30
308251	1.10	0.22	663.50	20	13.27
221113	1.26	0.29	875.10	20	17.50
295486	1.32	0.23	706.00	20	14.12
363322	1.31	0.23	675.50	20	13.51
316487	1.21	0.20	595.30	20	11.91
267069	1.37	0.29	881.60	20	17.63
297410	1.32	0.22	664.60	20	13.29
102302	1.27	0.26	805.90	20	16.12
328985	1.24	0.22	655.50	20	13.11
346418	1.31	0.21	645.40	20	12.91
256101	1.16	0.27	850.00	20	17.00
47907	1.36	0.19	559.20	20	11.18
332360	1.48	0.18	523.30	20	10.47
69202	1.52	7.86	405.50	20	8.11
326090	1.21	0.17	517.00	20	10.34
90704	1.55	6.52	382.00	20	7.64
370650	1.48	6.38	363.60	20	7.27
340145	1.45	5.18	320.80	20	6.42
96554	1.46	8.19	458.40	20	9.17
220524	1.58	9.15	452.20	20	9.04
116144	1.33	5.25	462.10	20	9.24
119046	1.15	0.26	810.20	20	16.20
353004	1.22	0.20	618.60	20	12.37
89352	1.19	0.29	886.60	20	17.73
72358	1.28	0.22	650.20	20	13.00
371609	1.25	0.26	788.30	20	15.77
104051	1.12	0.27	850.30	20	17.01
79794	1.30	0.29	780.10	20	15.60
267683	1.28	0.24	721.50	20	14.43
352748	1.36	0.28	853.60	20	17.07
361817	1.35	0.24	741.20	20	14.82
368588	1.62	0.61	1909.20	20	38.18
28219	1.39	0.41	1277.20	20	25.54
342196	1.39	0.30	436.80	20	8.74
107784	1.43	0.34	1027.50	20	20.55
92045	1.55	0.59	1853.10	20	37.06
368273	1.36	0.24	732.80	20	14.66

376778	1.75	0.68	2148.30	20	42.97
371240	1.25	0.21	620.30	20	12.41
353234	1.64	0.61	1945.40	20	38.91
299046	1.21	0.23	681.60	20	13.63
124668	1.33	0.19	571.30	20	11.43
84761	1.25	0.22	658.60	20	13.17
368010	1.56	10.59	470.20	20	9.40
371562	1.35	0.19	564.20	20	11.28
374724	1.32	0.20	608.70	20	12.17
260864	1.20	0.22	673.40	20	13.47
101938	1.24	0.28	842.60	20	16.85
375898	1.34	0.20	609.00	20	12.18
378419	1.37	0.19	559.40	20	11.19
97766	1.34	0.19	547.10	20	10.94
67506	1.37	0.23	676.50	20	13.53
100397	1.32	4.75	406.20	20	8.12
378503	1.61	0.53	1652.40	20	33.05
93305	1.33	5.26	468.10	20	9.36
91909	1.23	0.21	545.50	20	10.91
376079	1.28	4.42	380.50	20	7.61
308844	1.44	0.33	1001.90	20	20.04
351126	1.26	5.14	435.80	20	8.72
375767	1.27	0.26	766.30	20	15.33
378037	1.27	9.09	461.20	20	9.22
383288	1.19	0.25	741.90	20	14.84
123277	1.22	0.20	579.50	20	11.59
97152	1.23	0.25	748.50	20	14.97
375688	1.29	0.30	881.50	20	17.63
377011	1.19	0.21	609.50	20	12.19
152522	1.31	0.20	581.60	20	11.63
284255	1.30	0.26	792.60	20	15.85
348500	1.15	0.20	576.00	20	11.52
210949	1.16	0.19	567.30	20	11.35
381067	1.07	0.23	639.60	20	12.79
379364	1.17	0.28	846.20	20	16.92
385042	1.28	0.21	652.20	20	13.04
111529	1.32	0.24	690.90	20	13.82
154606	1.24	0.22	633.70	20	12.67
385942	1.27	0.21	626.70	20	12.53
358392	1.32	0.25	740.20	20	14.80
386082	1.32	0.23	680.20	20	13.60
27979	1.20	0.23	690.20	20	13.80
386022	1.25	0.25	765.80	20	15.32
22999	1.31	0.22	648.20	20	12.96
384161	1.85	1.21	3939.00	20	78.78
393314	1.56	0.54	1723.20	20	34.46
389683	1.82	1.23	4014.40	20	80.29
127789	1.66	1.02	3353.30	20	67.07
295658	1.92	1.05	3364.90	20	67.30
385539	1.45	0.46	1432.90	20	28.66
389163	1.89	1.19	3869.50	20	77.39
48433	1.33	0.53	1689.70	20	33.79
390272	1.90	1.11	3665.60	20	73.31

397018	1.52	0.56	1756.60	20	35.13
104965	1.30	0.21	620.80	20	12.42
393279	1.32	0.22	672.80	20	13.46
381653	1.32	0.23	705.00	20	14.10
350193	1.33	0.21	654.20	20	13.08
226784	1.20	0.21	629.10	20	12.58
401896	1.33	0.24	738.90	20	14.78
304085	1.45	0.22	653.70	20	13.07
397453	1.32	0.18	540.80	20	10.82
378497	1.19	0.27	808.60	20	16.17
91886	1.13	0.21	653.10	20	13.06
259497	1.87	1.05	3430.10	20	68.60
105514	1.52	0.79	2568.20	20	51.36
396069	1.71	0.85	2768.30	20	55.37
395734	1.45	0.67	2187.20	20	43.74
80944	1.62	0.64	2023.30	20	40.47
396261	1.30	0.33	1025.20	20	20.50
396187	1.83	1.15	3757.20	20	75.14
373649	1.41	0.34	1047.70	20	20.95
402740	1.71	0.87	2839.30	20	56.79
398206	1.39	0.45	1414.80	20	28.30
398879	1.46	0.21	641.40	20	12.83
391458	1.38	0.22	672.00	20	13.44
405566	1.19	0.23	709.30	20	14.19
190834	1.34	0.25	769.70	20	15.39
219230	1.41	0.21	630.40	20	12.61
343916	1.37	0.20	619.60	20	12.39
281071	1.07	0.26	815.00	20	16.30
112910	1.43	0.22	649.10	20	12.98
312417	0.98	0.24	750.90	20	15.02
402674	1.33	0.19	588.90	20	11.78
84558	1.56	1.31	4343.10	20	86.86
403643	1.64	1.30	4287.00	20	85.74
402671	1.70	1.28	4249.20	20	84.98
359233	1.71	1.27	4198.40	20	83.97
96096	1.58	1.32	4351.60	20	87.03
410893	1.35	0.40	1261.50	20	25.23
168798	1.70	1.28	4278.10	20	85.56
405686	1.72	1.27	4210.00	20	84.20
105222	1.63	1.30	4298.30	20	85.97
305473	1.57	0.63	2006.30	20	40.13
59023	1.52	0.34	1053.00	20	21.06
36467	1.54	0.29	896.00	20	17.92
60754	1.49	0.35	1080.70	20	21.61
62520	1.49	0.33	1023.70	20	20.47
63226	1.53	0.30	949.70	20	18.99
64822	1.48	0.35	1093.00	20	21.86
69861	1.47	0.31	976.40	20	19.53
68565	1.53	0.30	929.80	20	18.60
70944	1.50	0.31	931.80	20	18.64
70459	1.55	0.30	947.60	20	18.95
39351	1.87	1.21	3976.40	20	79.53
96101	1.77	1.23	4083.00	20	81.66

96135	1.71	1.28	4220.80	20	84.42
89860	1.50	0.47	1508.00	20	30.16
97581	1.76	1.28	4223.60	20	84.47
98150	1.41	0.44	1372.80	20	27.46
106263	1.53	1.28	4204.40	20	84.09
108875	1.81	1.17	3850.50	20	77.01
110982	1.70	1.28	4238.50	20	84.77
111090	1.30	0.21	612.00	20	12.24
68813	1.28	0.24	695.40	20	13.91
107855	1.23	0.24	710.30	20	14.21
159859	1.29	0.26	779.60	20	15.59
73826	1.22	0.27	795.90	20	15.92
188023	1.46	0.20	580.80	20	11.62
332930	1.18	0.21	617.10	20	12.34
200433	1.26	0.20	598.30	20	11.97
362925	1.31	0.22	639.40	20	12.79
	260/280	260/230	Conc. (ng/ul)	Total Vol. (ul)	Total RNA (ug)
ave	1.34	2.08	787.14	20.00	15.74
sd	0.18	7.94	790.56	0.00	15.81
count	1018	1018	1018	1018	1018
min	0.82	-20.76	167.20	20.00	3.34
max	1.92	240.84	4401.00	20.00	88.02

supplemental Table S2. mRNAs from Discovery for next step in

workflow GROUP 1. mRNA microarry results: Up-regulated > 1.5 fold

Probeset ID	gene_assignment	Gene Symbol	seqname	Raw p-value (T21)	Fold-Change(T21 vs. Ctrl)
3275312	---	0	---	0.0366917	1.65445
2424546	---	0	---	0.000364734	1.63255
3028397	---	0	---	0.00589025	1.56845
2320403	NM_198545 // C1orf187 /// ENST00000294485 // C1orf187 /// AK075558 // C1orf187	C1orf187	chr1	0.00836328	3.40239
2332841	NM_001017922 // ERMAP /// NM_018538 // ERMAP /// ENST00000372517 // ERMAP /// EN	ERMAP	chr1	0.0267229	2.82256
2326973	NM_032125 // TMEM222 /// ENST00000374076 // TMEM222 /// BC090039 // TMEM222 ///	TMEM222	chr1	0.0266073	2.13592
2348518	NM_014839 // LPPR4 /// NM_001166252 // LPPR4 /// ENST00000370185 // LPPR4 /// AF	LPPR4	chr1	0.0323005	2.12424
2392307	NM_024848 // MORN1 /// ENST00000378531 // MORN1 /// ENST00000418159 // MORN1	MORN1	chr1	0.0224549	2.10934
2336440	NM_015696 // GPX7 /// ENST00000361314 // GPX7 /// AF320068 // GPX7	GPX7	chr1	0.00129266	2.09954
2437125	NM_002456 // MUC1 /// NM_001018016 // MUC1 /// NM_001018017 // MUC1 /// NM_0010	MUC1	chr1	0.00486031	2.04863
2409510	NM_006934 // SLC6A9 /// NM_201649 // SLC6A9 /// NM_001024845 // SLC6A9 /// ENST0	SLC6A9	chr1	0.000902513	2.04373
2413321	NR_002314 // FLJ40434 /// AK097753 // FLJ40434	FLJ40434	chr1	0.00486868	2.02739
2339875	NM_005012 // ROR1 /// NM_001083592 // ROR1 /// ENST00000371079 // ROR1 /// ENST0	ROR1	chr1	0.0215483	2.02729
2407511	NM_002699 // POU3F1 /// ENST00000373012 // POU3F1 /// L26494 // POU3F1	POU3F1	chr1	0.00497019	2.02443
2333645	NM_001384 // DPH2 /// NM_001039589 // DPH2 /// ENST00000255108 // DPH2 /// ENST0	DPH2	chr1	0.0107631	2.00366
2426854	NM_001142550 // WDR47 /// NM_014969 // WDR47 /// NM_001142551 // WDR47 /// ENST0	WDR47	chr1	0.0161046	1.94302
2398818	NM_003000 // SDHB /// ENST00000375499 // SDHB /// BC007840 // SDHB	SDHB	chr1	0.0207456	1.94093
2406586	NM_005202 // COL8A2 /// ENST00000397799 // COL8A2 /// ENST00000303143 // COL8A2	COL8A2	chr1	0.00575651	1.93319
2400917	NM_005529 // HSPG2 /// ENST00000374695 // HSPG2 /// M85289 // HSPG2 /// ENST0000	HSPG2	chr1	0.0154274	1.90474
2410567	NM_017739 // POMGNT1 /// ENST00000371984 // POMGNT1 /// BC001471 // POMGNT1 ///	POMGNT1	chr1	0.027385	1.88933
2438102	NM_144580 // C1orf85 /// ENST00000362007 // C1orf85 /// BC011575 // C1orf85	C1orf85	chr1	4.30206E-05	1.88445
2441401	NM_003617 // RGS5 /// ENST00000313961 // RGS5 /// BX537427 // RGS5 /// ENST00000	RGS5	chr1	0.00823156	1.87753
2336541	NM_001004339 // ZYG11A /// AY271826 // ZYG11A /// ENST00000371532 // ZYG11A ///	ZYG11A	chr1	0.0289312	1.87509
2351764	NM_021797 // CHIA	CHIA	chr1	0.0203596	1.84664
2400921	NM_005529 // HSPG2 /// ENST00000374695 // HSPG2 /// M85289 // HSPG2 /// ENST0000	HSPG2	chr1	0.000611928	1.8463
2347100	NM_001938 // DR1 /// ENST00000370272 // DR1 /// BC035507 // DR1	DR1	chr1	0.0042231	1.84261
2421025	NM_152890 // COL24A1 /// ENST00000370571 // COL24A1 /// AY244357 // COL24A1 ///	COL24A1	chr1	0.0232256	1.8348
2435693	NM_016190 // CRNN /// ENST00000271835 // CRNN /// BC030807 // CRNN	CRNN	chr1	0.0181354	1.83382
2406762	NM_145047 // OSCP1 /// NM_206837 // OSCP1 /// ENST00000356637 // OSCP1 /// ENST0	OSCP1	chr1	0.0277788	1.82644
2319581	NM_001105562 // UBE4B /// NM_006048 // UBE4B /// ENST00000343090 // UBE4B /// EN	UBE4B	chr1	0.0338696	1.82295
2464508	NM_031844 // HNRNPU /// NM_004501 // HNRNPU /// ENST00000366525 // HNRNPU /// EN	HNRNPU	chr1	0.00112368	1.80462
2422406	NM_020063 // BARHL2 /// ENST00000370445 // BARHL2 /// AY338397 // BARHL2	BARHL2	chr1	0.00359934	1.79784
2389794	NM_016002 // SCCPDH /// ENST00000366510 // SCCPDH /// AF151807 // SCCPDH /// ENS	SCCPDH	chr1	0.0193373	1.79195
2402004	NM_001031680 // RUNX3 /// NM_004350 // RUNX3 /// ENST00000308873 // RUNX3 /// EN	RUNX3	chr1	0.0312958	1.78589
2400825	NM_005529 // HSPG2 /// ENST00000374695 // HSPG2 /// M85289 // HSPG2 /// ENST0000	HSPG2	chr1	0.0179581	1.77804
2360578	NM_207191 // ADAM15 /// NM_003815 // ADAM15 /// NM_207194 // ADAM15 /// NM_2071	ADAM15	chr1	0.00903462	1.76776
2406756	NM_145047 // OSCP1 /// NM_206837 // OSCP1 /// ENST00000356637 // OSCP1 /// ENST0	OSCP1	chr1	0.0160366	1.7632
2434258	NM_020205 // OTUD7B /// ENST00000369135 // OTUD7B /// BC072681 // OTUD7B /// ENS	OTUD7B	chr1	0.0139763	1.76146
2409009	NM_022356 // LEPRE1 /// NM_001146289 // LEPRE1 /// ENST00000296388 // LEPRE1 //	LEPRE1	chr1	0.0455254	1.75998
2409239	NM_001159936 // EBNA1BP2 /// NM_006824 // EBNA1BP2 /// ENST00000236051 // EBNA1E	EBNA1BP2	chr1	6.43434E-05	1.75842
2435247	NM_001083965 // TDRKH /// NM_001083963 // TDRKH /// NM_006862 // TDRKH /// NM_00	TDRKH	chr1	0.0303963	1.75644
2392552	NM_018216 // PANK4 /// ENST00000378466 // PANK4 /// BC043496 // PANK4 /// ENST00	PANK4	chr1	0.0102617	1.75613
2323568	NM_016183 // MRTO4 /// ENST00000330263 // MRTO4 /// AF173378 // MRTO4	MRTO4	chr1	0.0112417	1.74895
2321878	NM_015291 // DNAJC16 /// ENST00000375847 // DNAJC16 /// AL833299 // DNAJC16	DNAJC16	chr1	0.0307337	1.74663
2413152	NM_006671 // SLC1A7 /// ENST00000371494 // SLC1A7 /// BC012119 // SLC1A7 /// ENS	SLC1A7	chr1	0.0230087	1.73482
2322807	NM_013358 // PADI1 /// ENST00000375471 // PADI1 /// AK026652 // PADI1 /// ENST00	PADI1	chr1	0.0409625	1.72693

2447100	NM_002065 // GLUL /// NM_001033044 // GLUL /// ENST00000311223 // GLUL /// ENST(	GLUL	chr1	0.0212011	1.72196
2341669	NM_001902 // CTH /// NM_153742 // CTH /// ENST00000370938 // CTH /// ENST0000034	CTH	chr1	0.0449344	1.7187
2370561	NM_000721 // CACNA1E /// ENST00000367570 // CACNA1E /// ENST00000367567 // CACNA	CACNA1E	chr1	0.00606639	1.71496
2333458	NM_014663 // KDM4A /// ENST00000372396 // KDM4A /// BC002558 // KDM4A	KDM4A	chr1	0.0359931	1.71205
2438047	NM_015327 // SMG5 /// ENST00000361813 // SMG5 /// BC038296 // SMG5	SMG5	chr1	0.0468662	1.70652
2358989	NM_020770 // CGN /// ENST00000271636 // CGN /// BC146657 // CGN	CGN	chr1	0.026078	1.70429
2400947	NM_005529 // HSPG2 /// ENST00000374695 // HSPG2 /// M85289 // HSPG2 /// ENST0000(	HSPG2	chr1	0.00475206	1.70291
2398777	NM_022089 // ATP13A2 /// NM_001141973 // ATP13A2 /// NM_001141974 // ATP13A2 //.	ATP13A2	chr1	0.00358761	1.70127
2438903	NM_031281 // FCRL5 /// ENST00000361835 // FCRL5 /// AF343664 // FCRL5 /// ENST0(	FCRL5	chr1	0.00845833	1.69975
2419459	NM_022159 // ELTD1 /// ENST00000370742 // ELTD1 /// AB032017 // ELTD1 /// ENST0(	ELTD1	chr1	0.00554182	1.69847
2458519	NM_014698 // TMEM63A /// ENST00000366835 // TMEM63A /// BC030245 // TMEM63A	TMEM63A	chr1	0.0111577	1.69796
2340155	NM_020925 // CACHD1 /// ENST00000290039 // CACHD1 /// BC039301 // CACHD1 /// ENS	CACHD1	chr1	0.00340119	1.69169
2443413	NM_000130 // F5 /// ENST00000367797 // F5 /// M14335 // F5 /// ENST00000367796 .	F5	chr1	0.0192718	1.69
2450650	NM_000069 // CACNA1S /// ENST00000362061 // CACNA1S /// L33798 // CACNA1S /// EN	CACNA1S	chr1	0.0397434	1.68205
2358122	NM_024579 // C1orf54 /// BC017761 // C1orf54 /// ENST00000369098 // C1orf54 ///	C1orf54	chr1	0.0444777	1.67645
2458613	NM_013328 // PYCR2 /// AK001508 // PYCR2 /// ENST00000316940 // LEFTY1	PYCR2	chr1	0.000249422	1.67588
2401022	NM_030761 // WNT4 /// ENST00000290167 // WNT4 /// AF316543 // WNT4 /// BT020125	WNT4	chr1	0.0143098	1.67577
2320407	NM_198545 // C1orf187 /// ENST00000294485 // C1orf187 /// AK075558 // C1orf187	C1orf187	chr1	0.0399943	1.67365
2382060	NM_152610 // C1orf65 /// ENST00000366875 // C1orf65 /// BC031066 // C1orf65	C1orf65	chr1	0.0157354	1.67318
2333727	NM_152499 // CCDC24 /// ENST00000372318 // CCDC24 /// BC047313 // CCDC24 /// ENS	CCDC24	chr1	0.0031039	1.6653
2334408	NM_172164 // NASP /// NM_002482 // NASP /// NM_152298 // NASP /// ENST0000035003	NASP	chr1	0.01297	1.66088
2436174	---	0	chr1	0.00741705	1.65589
2371171	NM_005562 // LAMC2 /// NM_018891 // LAMC2 /// ENST00000264144 // LAMC2 /// ENST(	LAMC2	chr1	0.0279219	1.65386
2451910	NM_000537 // REN /// ENST00000272190 // REN /// BC033474 // REN /// ENST00000367	REN	chr1	0.0285591	1.65042
2321288	NM_015866 // PRDM2 /// NM_001007257 // PRDM2 /// ENST00000407521 // PRDM2 /// EN	PRDM2	chr1	0.00056713	1.6502
2370815	NM_001137669 // RGSL1 /// AK302768 // RGSL1	RGSL1	chr1	0.00263217	1.64498
2428412	NM_175744 // RHOC /// NM_001042678 // RHOC /// NM_001042679 // RHOC /// ENST0000(	RHOC	chr1	0.0498754	1.6431
2339696	NM_013339 // ALG6 /// ENST00000405765 // ALG6 /// ENST00000371108 // ALG6 /// AF	ALG6	chr1	0.0269933	1.6427
2438315	NM_178229 // IQGAP3 /// ENST00000361170 // IQGAP3 /// AB105103 // IQGAP3	IQGAP3	chr1	0.000815506	1.63493
2401731	NM_152372 // MYOM3 /// ENST00000374434 // MYOM3 /// BC067101 // MYOM3 /// ENST0(	MYOM3	chr1	0.0313306	1.63321
2330183	NM_024852 // EIF2C3 /// NM_177422 // EIF2C3 /// ENST00000373185 // EIF2C3 /// EN	EIF2C3	chr1	0.0263176	1.63195
2371066	NM_002293 // LAMC1 /// ENST00000258341 // LAMC1 /// J03202 // LAMC1	LAMC1	chr1	0.0388131	1.62817
2414420	NM_003713 // PPAP2B /// ENST00000371250 // PPAP2B	PPAP2B	chr1	0.0106366	1.62573
2397749	NM_024758 // AGMAT /// ENST00000375826 // AGMAT /// BC005090 // AGMAT	AGMAT	chr1	0.00159344	1.62486
2376897	ENST00000367106 // DYRK3 /// AF186774 // DYRK3	DYRK3	chr1	0.00727704	1.62428
2392554	NM_018216 // PANK4 /// ENST00000378466 // PANK4 /// BC043496 // PANK4 /// ENST0(	PANK4	chr1	0.00987038	1.62121
2404434	NM_004102 // FABP3 /// ENST00000373713 // FABP3 /// BT006727 // FABP3	FABP3	chr1	0.0399972	1.61897
2334001	NM_018150 // RNF220 /// ENST00000355387 // RNF220 /// ENST00000361799 // RNF220	RNF220	chr1	0.0179046	1.61889
2390197	NM_015431 // TRIM58 /// AF327057 // TRIM58	TRIM58	chr1	0.00936421	1.61683
2394825	NM_014851 // KLHL21 /// ENST00000377658 // KLHL21 /// ENST00000377663 // KLHL21	KLHL21	chr1	0.00351375	1.61569
2436781	NM_001111 // ADAR /// NM_015840 // ADAR /// NM_015841 // ADAR /// NM_001025107 .	ADAR	chr1	0.00150181	1.61072
2363261	NM_002348 // LY9 /// ENST00000368041 // LY9 /// ENST00000263285 // LY9 /// BC12'	LY9	chr1	0.00389349	1.60679
2370532	NM_000721 // CACNA1E /// ENST00000367570 // CACNA1E /// ENST00000367567 // CACNA	CACNA1E	chr1	0.0037611	1.60184
2407951	NM_001135653 // PABPC4 /// NM_001135654 // PABPC4 /// ENST00000372856 // PABPC4	PABPC4	chr1	0.0214239	1.59796
2404611	NM_001856 // COL16A1 /// ENST00000373672 // COL16A1 /// M92642 // COL16A1 /// EN	COL16A1	chr1	0.0498776	1.59555
2318697	NM_016831 // PER3 /// ENST00000361923 // PER3 /// BC146781 // PER3 /// ENST0000(	PER3	chr1	0.0394957	1.59364
2376833	NM_014002 // IKBKE /// ENST00000367120 // IKBKE /// BC105923 // IKBKE /// AK0571	IKBKE	chr1	0.0351933	1.59217
2418009	NM_203350 // ZRANB2 /// NM_005455 // ZRANB2 /// ENST00000370920 // ZRANB2 /// EN	ZRANB2	chr1	0.0324494	1.59156
2334127	NM_006845 // KIF2C /// ENST00000372224 // KIF2C /// BT006759 // KIF2C /// AB2641	KIF2C	chr1	0.0253793	1.59041
2378172	NM_025228 // TRAF3IP3 /// ENST00000367026 // TRAF3IP3 /// ENST00000010338 // TRA	TRAF3IP3	chr1	0.0240008	1.58977
2438622	NM_014215 // INSRR /// ENST00000368195 // INSRR	INSRR	chr1	0.020263	1.58918

2402417	NM_024037 // C1orf135 /// ENST00000374298 // C1orf135	C1orf135	chr1	0.00510987	1.58907
2358597	NM_003568 // ANXA9 /// ENST00000368947 // ANXA9 /// AF230929 // ANXA9	ANXA9	chr1	0.0240034	1.58627
2326719	NM_017837 // PIGV /// ENST00000078527 // PIGV /// ENST00000374145 // PIGV /// BC	PIGV	chr1	0.0075964	1.58258
2351217	NM_033088 // FAM40A /// ENST00000369795 // FAM40A /// ENST00000369796 // FAM40A	FAM40A	chr1	0.0449662	1.58108
2400248	NM_020816 // KIF17 /// NM_001122819 // KIF17 /// ENST00000247986 // KIF17 /// EN	KIF17	chr1	0.0255313	1.57193
2435699	NM_016190 // CRNN /// ENST00000271835 // CRNN /// BC030807 // CRNN /// ENST00000	CRNN	chr1	0.00875497	1.56819
2431118	NM_024408 // NOTCH2 /// ENST00000256646 // NOTCH2 /// AF315356 // NOTCH2	NOTCH2	chr1	0.012824	1.56525
2323995	NM_001039500 // VWA5B1 /// ENST00000414846 // VWA5B1 /// ENST00000375089 // VWA5	VWA5B1	chr1	0.0274091	1.56451
2333333	NM_002840 // PTPRF /// NM_130440 // PTPRF /// ENST00000359947 // PTPRF /// ENST0	PTPRF	chr1	0.0353578	1.56328
2438427	NM_006617 // NES /// ENST00000368223 // NES	NES	chr1	0.0428677	1.56094
2402450	NM_001145454 // STMN1 /// AK056768 // STMN1	STMN1	chr1	0.00124005	1.56006
2435995	NM_002964 // S100A8 /// ENST00000368732 // S100A8 /// ENST00000368733 // S100A8	S100A8	chr1	0.0246018	1.55968
2455831	NM_206933 // USH2A /// ENST00000307340 // USH2A /// AY481573 // USH2A /// ENST00	USH2A	chr1	0.00430521	1.5588
2318128	NM_003636 // KCNAB2 /// NM_172130 // KCNAB2 /// ENST00000378097 // KCNAB2 /// EN	KCNAB2	chr1	0.0301801	1.55778
2398754	NM_022089 // ATP13A2 /// NM_001141973 // ATP13A2 /// NM_001141974 // ATP13A2 //	ATP13A2	chr1	0.0221877	1.5575
2392408	NR_024489 // LOC100129534	LOC100129534	chr1	0.0266178	1.55634
2427232	NM_139053 // EPS8L3 /// NM_133181 // EPS8L3 /// NM_024526 // EPS8L3 /// ENST0000	EPS8L3	chr1	0.0293631	1.55565
2458723	NM_022735 // ACBD3 /// ENST00000366812 // ACBD3 /// BC045533 // ACBD3	ACBD3	chr1	0.0324384	1.55344
2352295	NM_020963 // MOV10 /// NM_001130079 // MOV10 /// ENST00000369648 // MOV10 /// EN	MOV10	chr1	0.0126165	1.54724
2457864	NM_001031685 // TP53BP2 /// NM_005426 // TP53BP2 /// ENST00000343537 // TP53BP2	TP53BP2	chr1	0.0469308	1.54605
2447430	NM_000433 // NCF2 /// NM_001127651 // NCF2 /// ENST00000367536 // NCF2 /// ENST0	NCF2	chr1	0.0462301	1.54595
2337301	NM_004623 // TTC4 /// ENST00000371284 // TTC4 /// ENST00000425300 // TTC4 /// EN	TTC4	chr1	0.0160042	1.54521
2412633	NM_002867 // RAB3B /// ENST00000371655 // RAB3B /// BC005035 // RAB3B	RAB3B	chr1	0.0369897	1.54496
2452763	NM_052934 // SLC26A9 /// NM_134325 // SLC26A9 /// NM_001142600 // SLC26A9 /// EN	SLC26A9	chr1	0.0413676	1.54099
2384167	NM_052843 // OBSCN /// NM_001098623 // OBSCN /// ENST00000422127 // OBSCN /// EN	OBSCN	chr1	0.0416727	1.54075
2436783	NM_001111 // ADAR /// NM_015840 // ADAR /// NM_015841 // ADAR /// NM_001025107 /	ADAR	chr1	0.0434978	1.5391
2370361	NM_016545 // IER5 /// ENST00000367577 // IER5 /// BC000128 // IER5	IER5	chr1	0.0445502	1.53815
2337248	NR_026782 // C1orf175 /// NM_001039464 // C1orf175 /// AK301894 // C1orf175 ///	C1orf175	chr1	0.0264623	1.53806
2417010	NM_005478 // INSL5 /// ENST00000304526 // INSL5 /// AF133816 // INSL5	INSL5	chr1	0.0199522	1.53679
2360946	GU480875 // POU5F1P4 /// GU480894 // POU5F1P4 /// GU480879 // POU5F1P4 /// GU480	POU5F1P4	chr1	0.0124751	1.53487
2444246	NM_005092 // TNFSF18 /// ENST00000404377 // TNFSF18 /// AY358868 // TNFSF18 ///	TNFSF18	chr1	0.00792884	1.53468
4053109	NM_002725 // PRELP /// NM_201348 // PRELP /// ENST00000343110 // PRELP /// ENST0	PRELP	chr1	0.0355681	1.53319
2406754	NM_145047 // OSCP1 /// ENST00000356637 // OSCP1 /// ENST00000433045 // OSCP1 //	OSCP1	chr1	0.00882933	1.5327
2428161	NM_004980 // KCND3 /// NM_172198 // KCND3 /// ENST00000315987 // KCND3 /// ENST0	KCND3	chr1	0.0495564	1.53114
2446230	NM_022347 // TOR1AIP2 /// NM_145034 // TOR1AIP2 /// ENST00000419174 // TOR1AIP2	TOR1AIP2	chr1	0.000797819	1.53078
2351714	NM_004000 // CHI3L2 /// NM_001025197 // CHI3L2 /// NM_001025199 // CHI3L2 /// EN	CHI3L2	chr1	0.040879	1.52955
2465524	NM_024804 // ZNF669 /// NM_001142572 // ZNF669 /// ENST00000343381 // ZNF669 //	ZNF669	chr1	0.010894	1.52905
2357969	NM_016074 // BOLA1 /// ENST00000369153 // BOLA1 /// ENST00000369152 // BOLA1 //	BOLA1	chr1	0.0340624	1.52848
2410375	NM_021639 // GPBP1L1 /// ENST00000290795 // GPBP1L1 /// ENST00000355105 // GPBP1	GPBP1L1	chr1	0.00814559	1.52786
2450816	NM_005558 // LAD1 /// ENST00000391967 // LAD1 /// BC009742 // LAD1 /// ENST00000	LAD1	chr1	0.0272966	1.52523
2436327	NM_014856 // DENND4B /// ENST00000368646 // DENND4B /// ENST00000361217 // DENND	DENND4B	chr1	0.00915691	1.52485
2354660	NM_006623 // PHGDH /// ENST00000369409 // PHGDH /// AK093306 // PHGDH /// ENST00	PHGDH	chr1	0.0417772	1.52432
2329833	NM_005095 // ZMYM4 /// ENST00000314607 // ZMYM4 /// ENST00000373297 // ZMYM4 //	ZMYM4	chr1	0.034942	1.52349
2393545	ENST00000378322 // WDR8	WDR8	chr1	0.0469295	1.52292
2406258	NM_002794 // PSMB2 /// ENST00000373237 // PSMB2 /// BC107901 // PSMB2 /// ENST00	PSMB2	chr1	0.027012	1.52264
2405691	NM_052896 // CSMD2 /// ENST00000241312 // CSMD2 /// AB212622 // CSMD2 /// ENST00	CSMD2	chr1	0.00689563	1.52107
2329504	NM_001134734 // C1orf94 /// NM_032884 // C1orf94 /// ENST00000398041 // C1orf94	C1orf94	chr1	0.0285565	1.51371
2408669	NM_001956 // EDN2 /// ENST00000372587 // EDN2 /// M65199 // EDN2	EDN2	chr1	0.0214476	1.51279
2333656	NM_001384 // DPH2 /// NM_001039589 // DPH2 /// ENST00000255108 // DPH2 /// ENST0	DPH2	chr1	0.0255582	1.51169
2405271	NM_001171941 // FNDC5 /// BC062297 // FNDC5	FNDC5	chr1	0.0347179	1.50874
2439356	NM_001005185 // OR6N1 /// ENST00000335094 // OR6N1 /// BC146847 // OR6N1	OR6N1	chr1	0.0339682	1.50843

2440047	NR_028103 // DCAF8 /// NR_028104 // DCAF8 /// NM_015726 // DCAF8 /// ENST0000036	DCAF8	chr1	0.0138729	1.5063
3253855	NM_020338 // ZMIZ1 /// ENST00000334512 // ZMIZ1 /// AY235683 // ZMIZ1 /// ENST0C	ZMIZ1	chr10	0.00893748	2.35344
3278334	NM_152751 // BEND7 /// NM_001100912 // BEND7 /// ENST00000341083 // BEND7 /// EN	BEND7	chr10	0.0373916	2.16421
3262168	NM_006951 // TAF5 /// ENST00000369839 // TAF5 /// BC136348 // TAF5 /// ENST0000C	TAF5	chr10	0.00623204	1.93853
3254544	NM_030927 // TSPAN14 /// ENST00000429989 // TSPAN14 /// ENST00000372158 // TSPAN	TSPAN14	chr10	0.00223692	1.93021
3256705	NM_000314 // PTEN /// NR_023917 // PTENP1 /// U92436 // PTEN	PTEN	chr10	0.00452581	1.80698
3272676	NM_152643 // KNDC1 /// ENST00000304613 // KNDC1 /// AB000781 // KNDC1 /// ENST0C	KNDC1	chr10	0.00939231	1.77961
3303443	NM_016112 // PKD2L1 /// ENST00000318222 // PKD2L1 /// AF073481 // PKD2L1 /// ENS	PKD2L1	chr10	0.0113066	1.74124
3302232	NM_012083 // FRAT2 /// ENST00000371019 // FRAT2 /// BC020165 // FRAT2	FRAT2	chr10	0.0077708	1.73937
3272617	NM_152643 // KNDC1 /// ENST00000304613 // KNDC1 /// AB000781 // KNDC1 /// ENST0C	KNDC1	chr10	0.0385518	1.70014
3290669	NM_198215 // FAM13C /// NM_001001971 // FAM13C /// NM_001143773 // FAM13C /// NM	FAM13C	chr10	0.004838	1.66655
3236435	NM_016299 // HSPA14 /// ENST00000378372 // HSPA14 /// AK292323 // HSPA14	HSPA14	chr10	0.0408789	1.66511
3255363	NM_002921 // RGR /// NM_001012720 // RGR /// NM_001012722 // RGR /// ENST0000037	RGR	chr10	0.0379965	1.64528
3265178	NM_198795 // TDRD1 /// ENST00000251864 // TDRD1 /// ENST00000369282 // TDRD1 //	TDRD1	chr10	0.0346729	1.62918
3256038	NM_007078 // LDB3 /// NM_001080114 // LDB3 /// NM_001171610 // LDB3 /// ENST0000	LDB3	chr10	0.00465892	1.61439
3294691	NM_001114133 // SYNPO2L /// ENST00000394810 // SYNPO2L /// AB188489 // SYNPO2L /	SYNPO2L	chr10	0.0492764	1.61102
3284617	NM_019619 // PARD3 /// ENST00000374789 // PARD3 /// AF332593 // PARD3 /// AF1961	PARD3	chr10	0.00769424	1.60548
3251248	NM_022124 // CDH23 /// NM_001171933 // CDH23 /// NM_001171934 // CDH23 /// ENSTC	CDH23	chr10	0.018704	1.60452
3265146	NM_000684 // ADRB1 /// ENST00000369295 // ADRB1 /// J03019 // ADRB1	ADRB1	chr10	0.0168505	1.59948
3305026	NM_024928 // OBFC1 /// ENST00000224950 // OBFC1 /// ENST00000369764 // OBFC1 //	OBFC1	chr10	0.0273039	1.5962
3315078	NM_001109 // ADAM8 /// NM_001164489 // ADAM8 /// NM_001164490 // ADAM8 /// ENSTC	ADAM8	chr10	0.00823736	1.59171
3301323	NM_001034954 // SORBS1 /// NM_001034955 // SORBS1 /// NM_001034956 // SORBS1 //	SORBS1	chr10	0.00786642	1.58938
3263757	NM_004419 // DUSP5 /// ENST00000369583 // DUSP5 /// BC062545 // DUSP5	DUSP5	chr10	0.0445427	1.58241
3268191	NM_206862 // TACC2 /// NM_206861 // TACC2 /// NM_206860 // TACC2 /// NM_006997 /	TACC2	chr10	0.0367382	1.57467
3297653	NM_000429 // MAT1A /// ENST00000372213 // MAT1A /// BC018359 // MAT1A /// ENST0C	MAT1A	chr10	0.0228528	1.57429
3265476	NM_001135051 // FAM160B1 /// ENST00000369250 // FAM160B1 /// BC037207 // FAM160E	FAM160B1	chr10	0.031346	1.56598
3244058	AK127450 // LOC439911	LOC439911	chr10	0.00286203	1.55195
3285587	NM_021045 // ZNF248 /// ENST00000395867 // ZNF248 /// BC132915 // ZNF248 /// ENS	ZNF248	chr10	0.03206	1.55149
3303962	NM_022039 // FBXW4 /// ENST00000331272 // FBXW4 /// ENST00000389046 // FBXW4 //	FBXW4	chr10	0.0222114	1.54426
3293233	NM_173555 // TYSND1 /// NM_001040273 // TYSND1 /// ENST00000287078 // TYSND1 //.	TYSND1	chr10	0.0468679	1.54243
3303887	NM_001174084 // POLL /// NM_013274 // POLL /// NM_001174085 // POLL /// NR_0334C	POLL	chr10	0.0104325	1.54158
3279763	NM_001081 // CUBN /// ENST00000377833 // CUBN /// AF034611 // CUBN	CUBN	chr10	0.0153719	1.53346
3249695	NM_032578 // MYPN /// ENST00000358913 // MYPN /// AL834247 // MYPN /// ENST0000C	MYPN	chr10	0.0302919	1.52893
3265634	NM_207303 // ATRNL1 /// ENST00000355044 // ATRNL1 /// AY442317 // ATRNL1 /// ENS	ATRNL1	chr10	0.00668742	1.52664
3304786	NM_001001412 // CALHM1 /// ENST00000329905 // CALHM1 /// BC036193 // CALHM1	CALHM1	chr10	0.0443057	1.52347
3249606	NM_012238 // SIRT1 /// NM_001142498 // SIRT1 /// ENST00000212015 // SIRT1 /// EN	SIRT1	chr10	0.000150706	1.52124
3300217	NM_152429 // FGFBP3 /// ENST00000311575 // FGFBP3 /// BC025966 // FGFBP3	FGFBP3	chr10	0.0233996	1.51836
3260877	NM_018121 // FAM178A /// NM_001136123 // FAM178A /// ENST00000238961 // FAM178A	FAM178A	chr10	0.0348084	1.51677
3258610	NM_016341 // PLCE1 /// NM_001165979 // PLCE1 /// ENST00000260766 // PLCE1 /// EN	PLCE1	chr10	0.0216209	1.51476
3262138	NM_032727 // INA /// ENST00000369849 // INA /// BC006359 // INA	INA	chr10	0.0208592	1.51108
3239169	NM_019590 // KIAA1217 /// ENST00000376454 // KIAA1217 /// BX640796 // KIAA1217 /	KIAA1217	chr10	0.0205273	1.50407
3364736	NM_001017 // RPS13 /// ENST00000228140 // RPS13 /// BC100032 // RPS13	RPS13	chr11	0.0313364	2.63185
3334234	NM_006819 // STIP1 /// ENST00000305218 // STIP1 /// BC039299 // STIP1 /// ENST0C	STIP1	chr11	0.000914985	2.594
3323125	NM_182964 // NAV2 /// NM_145117 // NAV2 /// NM_001111018 // NAV2 /// ENST0000039	NAV2	chr11	0.00325867	2.32863
3335726	NM_006848 // CCDC85B /// ENST00000312579 // CCDC85B /// U63825 // CCDC85B	CCDC85B	chr11	0.0346858	2.2583
3350948	NM_014956 // CEP164 /// ENST00000278935 // CEP164 /// ENST00000375253 // CEP164	CEP164	chr11	0.00907788	2.25342
3358441	NM_145886 // LRDD /// NM_018494 // LRDD /// NM_145887 // LRDD /// ENST000003477	LRDD	chr11	0.0186053	2.21766
3354047	NM_001005198 // OR8G5 /// BC136820 // OR8G5	OR8G5	chr11	0.0228355	2.19468
3337061	NM_001166222 // CARNS1 /// NM_020811 // CARNS1 /// ENST00000307823 // ATPGD1 //	CARNS1	chr11	0.0210006	2.00888
3351864	NM_000190 // HMBS /// NM_001024382 // HMBS /// ENST00000278715 // HMBS /// ENSTC	HMBS	chr11	0.0018519	1.9804
3337609	NM_002335 // LRP5 /// ENST00000294304 // LRP5 /// AF077820 // LRP5	LRP5	chr11	0.0215933	1.95497

3338583	NM_005231 // CTTN /// NM_138565 // CTTN /// ENST00000346329 // CTTN /// ENST0000	CTTN	chr11	0.0126167	1.89937
3371322	NM_004813 // PEX16 /// NM_057174 // PEX16 /// ENST00000378750 // PEX16 /// ENST(	PEX16	chr11	0.0378208	1.8836
3315808	NM_007183 // PKP3 /// ENST00000331563 // PKP3 /// BC000081 // PKP3	PKP3	chr11	0.031659	1.87529
3360367	NM_001005222 // OR52A4 /// ENST00000380369 // OR52A4 /// BC140752 // OR52A4	OR52A4	chr11	0.0389936	1.82938
3378055	NM_053054 // CATSPER1 /// ENST00000312106 // CATSPER1 /// BC032950 // CATSPER1	CATSPER1	chr11	0.0275607	1.80643
3393297	NM_012104 // BACE1 /// NM_138972 // BACE1 /// NM_138971 // BACE1 /// NM_138973 /	BACE1	chr11	0.0230235	1.80092
3394114	NM_001164277 // SLC37A4 /// NM_001164278 // SLC37A4 /// NM_001164279 // SLC37A4	SLC37A4	chr11	0.012498	1.79734
3353045	NM_003105 // SORL1 /// ENST00000260197 // SORL1 /// BC137171 // SORL1	SORL1	chr11	0.00755913	1.7969
3336942	NM_017857 // SSH3 /// ENST00000308127 // SSH3 /// AK094226 // SSH3 /// ENST00000	SSH3	chr11	0.0282273	1.79602
3340598	NM_001235 // SERPINH1 /// ENST00000358171 // SERPINH1 /// ENST00000421448 // SER	SERPINH1	chr11	0.00564237	1.78101
3376186	NM_006362 // NXF1 /// NM_001081491 // NXF1 /// ENST00000294172 // NXF1 /// ENST(	NXF1	chr11	0.0191713	1.74827
3360457	NM_005330 // HBE1 /// ENST00000380237 // HBE1 /// ENST00000292896 // HBE1 /// BC	HBE1	chr11	0.043082	1.74107
3379640	NM_004923 // MTL5 /// NM_001039656 // MTL5 /// ENST00000255087 // MTL5 /// ENST(	MTL5	chr11	0.00233729	1.74018
3335834	---	0	chr11	0.0347492	1.70841
3317540	NM_002555 // SLC22A18 /// NM_183233 // SLC22A18 /// ENST00000347936 // SLC22A18	SLC22A18	chr11	0.0337689	1.70448
3371679	NM_004308 // ARHGAP1 /// ENST00000311956 // ARHGAP1 /// BC018118 // ARHGAP1 ///	ARHGAP1	chr11	0.0429914	1.69754
3325757	NM_024081 // PRRG4 /// ENST00000257836 // PRRG4 /// BC063393 // PRRG4	PRRG4	chr11	0.043324	1.69674
3397607	NM_001143820 // ETS1 /// NM_005238 // ETS1 /// NM_001162422 // ETS1 /// ENST0000	ETS1	chr11	0.00747438	1.69523
3377952	NM_016938 // EFEMP2 /// ENST00000307998 // EFEMP2 /// AB209121 // EFEMP2	EFEMP2	chr11	0.0137578	1.68486
3396119	AY189281 // ESAM /// AK092429 // ESAM	ESAM	chr11	0.0214575	1.66167
3356194	NM_021978 // ST14 /// ENST00000278742 // ST14 /// BC030532 // ST14	ST14	chr11	0.0178831	1.6611
3380195	NM_005247 // FGF3 /// ENST00000334134 // FGF3 /// BC113739 // FGF3	FGF3	chr11	0.0126172	1.65956
3365542	NM_194285 // SPTY2D1 /// ENST00000336349 // SPTY2D1 /// AK304078 // SPTY2D1 ///	SPTY2D1	chr11	0.00361903	1.65377
3378236	NM_006876 // B3GNT1 /// ENST00000311181 // B3GNT1 /// AF029893 // B3GNT1	B3GNT1	chr11	0.0078599	1.65306
3374526	NM_145016 // GLYATL2 /// ENST00000287275 // GLYATL2 /// BC016789 // GLYATL2	GLYATL2	chr11	0.0301478	1.64626
3333721	NM_001012661 // SLC3A2 /// NM_001012662 // SLC3A2 /// NM_002394 // SLC3A2 /// NM	SLC3A2	chr11	0.0334033	1.64419
3323192	NM_182964 // NAV2 /// NM_145117 // NAV2 /// NM_001111018 // NAV2 /// NM_0011110	NAV2	chr11	0.00296915	1.62677
3315815	NM_007183 // PKP3 /// ENST00000331563 // PKP3 /// BC000081 // PKP3	PKP3	chr11	0.00481584	1.62285
3367593	NM_002233 // KCNA4 /// ENST00000328224 // KCNA4 /// M55514 // KCNA4	KCNA4	chr11	0.0433319	1.62111
3375875	NM_004739 // MTA2 /// ENST00000278823 // MTA2 /// BC053650 // MTA2	MTA2	chr11	0.0302895	1.60269
3358427	NM_145886 // LRDD /// NM_018494 // LRDD /// NM_145887 // LRDD /// ENST000003477	LRDD	chr11	0.0233331	1.6019
3358133	NM_173573 // C11orf35 /// BC039077 // C11orf35 /// ENST00000441853 // C11orf35 /	C11orf35	chr11	0.0179209	1.5999
3371973	NM_016223 // PACSIN3 /// ENST00000298838 // PACSIN3 /// AF130979 // PACSIN3 ///	PACSIN3	chr11	0.0467886	1.59676
3373909	NM_003627 // SLC43A1 /// ENST00000278426 // SLC43A1 /// AB103033 // SLC43A1	SLC43A1	chr11	0.00746138	1.57846
3351685	NM_001716 // CXCR5 /// NM_032966 // CXCR5 /// ENST00000292174 // CXCR5 /// BC11(	CXCR5	chr11	0.033757	1.57815
3354194	NM_032811 // TBRG1 /// NR_016021 // TBRG1 /// ENST00000441174 // TBRG1 /// BC041	TBRG1	chr11	0.0403883	1.57732
3375869	NM_004739 // MTA2 /// ENST00000278823 // MTA2 /// BC053650 // MTA2	MTA2	chr11	0.0475759	1.57668
3322508	NM_002478 // MYOD1 /// ENST00000250003 // MYOD1 /// BC064493 // MYOD1	MYOD1	chr11	0.027806	1.57584
3327069	NM_024841 // PRR5L /// ENST00000378867 // PRR5L /// ENST00000311599 // PRR5L	PRR5L	chr11	0.0132198	1.56959
3352738	NM_014619 // GRIK4 /// ENST00000278723 // GRIK4 /// ENST00000438375 // GRIK4 //.	GRIK4	chr11	0.0113148	1.56927
3332980	NM_145017 // C11orf66 /// NM_001170753 // C11orf66 /// ENST00000338608 // C11or	C11orf66	chr11	0.0180731	1.5669
3376494	NM_054108 // HRASLS5 /// NM_001146729 // HRASLS5 /// NM_001146728 // HRASLS5 //.	HRASLS5	chr11	0.00946641	1.56479
3336090	NM_030981 // RAB1B /// ENST00000311481 // RAB1B /// BC071169 // RAB1B	RAB1B	chr11	0.00486533	1.56338
3339818	NM_014786 // ARHGEF17 /// ENST00000263674 // ARHGEF17 /// AF378754 // ARHGEF17	ARHGEF17	chr11	0.0206845	1.55795
3328394	NM_000401 // EXT2 /// ENST00000395673 // EXT2	EXT2	chr11	0.0362935	1.55382
3347701	NM_000051 // ATM /// ENST00000278616 // ATM /// ENST00000299392 // ATM /// U3384	ATM	chr11	0.0120748	1.5505
3328539	NM_002231 // CD82 /// NM_001024844 // CD82 /// ENST00000227155 // CD82 /// ENST(	CD82	chr11	0.0347018	1.54902
3350824	AK303023 // SIDT2	SIDT2	chr11	0.00308034	1.54895
3329738	NM_130470 // MADD /// NM_001135944 // MADD /// NM_130471 // MADD /// NM_130472 /	MADD	chr11	0.0499815	1.53854
3356186	NM_021978 // ST14 /// ENST00000278742 // ST14 /// BC030532 // ST14	ST14	chr11	0.0436868	1.53329
3374887	NM_005142 // GIF /// ENST00000257248 // GIF /// BC037958 // GIF	GIF	chr11	0.048989	1.53304

3396936	NM_003139 // SRPR /// NM_001177842 // SRPR /// ENST00000332118 // SRPR /// BC001	SRPR	chr11	0.00570421	1.53151
3386003	BC051371 // NOX4	NOX4	chr11	0.0304444	1.52899
3365515	NM_001040697 // UEVLD /// NM_018314 // UEVLD /// ENST00000396197 // UEVLD /// EN	UEVLD	chr11	0.0283103	1.5265
3332848	NM_015533 // DAK /// ENST00000394900 // DAK /// ENST00000311463 // DAK /// BC001	DAK	chr11	0.0136333	1.52623
3347592	NM_003478 // CUL5 /// ENST00000299351 // CUL5 /// BC063306 // CUL5 /// ENST0000C	CUL5	chr11	0.00591879	1.52211
3398161	NM_020228 // PRDM10 /// NM_199437 // PRDM10 /// NM_199438 // PRDM10 /// NM_19943	PRDM10	chr11	0.0439162	1.51367
3350860	NM_207343 // RNF214 /// NM_001077239 // RNF214 /// ENST00000300650 // RNF214 //.	RNF214	chr11	0.0170577	1.50862
3340479	NM_007256 // SLCO2B1 /// NM_001145211 // SLCO2B1 /// NM_001145212 // SLCO2B1 //.	SLCO2B1	chr11	0.0183397	1.50553
3343304	AK027167 // CCDC81	CCDC81	chr11	0.00388766	1.50471
3321521	NM_000922 // PDE3B /// ENST00000282096 // PDE3B /// BC150307 // PDE3B /// ENST0C	PDE3B	chr11	0.025377	1.50196
3414071	NM_012284 // KCNH3 /// ENST00000257981 // KCNH3 /// BC150602 // KCNH3	KCNH3	chr12	0.0279408	2.75488
3446307	NM_024730 // RERGL /// ENST00000229002 // RERGL /// BC042888 // RERGL	RERGL	chr12	0.046088	2.32543
3471063	NM_057169 // GIT2 /// NM_057170 // GIT2 /// NM_014776 // GIT2 /// NM_139201 // G	GIT2	chr12	0.0389543	2.23206
3453627	NM_003482 // MLL2 /// ENST00000301067 // MLL2 /// AF010403 // MLL2	MLL2	chr12	0.000803985	2.13744
3414147	NM_001031698 // PRPF40B /// NM_012272 // PRPF40B /// NM_175736 // FMNL3 /// NM_1	PRPF40B	chr12	0.0194152	1.95591
3464604	BC038413 // C12orf50 /// AK093140 // C12orf50 /// AL833338 // C12orf50 /// ENSTC	C12orf50	chr12	0.011159	1.84154
3468255	NM_016053 // CCDC53 /// ENST00000240079 // CCDC53 /// AF151874 // CCDC53	CCDC53	chr12	0.0309922	1.83338
3475084	---	0	chr12	0.0414281	1.78479
3402819	NM_019858 // GPR162 /// ENST00000311268 // GPR162 /// ENST00000382315 // GPR162	GPR162	chr12	0.0207114	1.76452
3475627	NM_002956 // CLIP1 /// NM_198240 // CLIP1 /// ENST00000358808 // CLIP1 /// ENSTC	CLIP1	chr12	0.0195286	1.73563
3431336	NM_032300 // TCHP /// NM_001143852 // TCHP /// ENST00000405876 // TCHP /// ENST0	TCHP	chr12	0.00467187	1.72673
3450713	NM_153634 // CPNE8 /// ENST00000331366 // CPNE8 /// BX538055 // CPNE8	CPNE8	chr12	0.0185542	1.72556
3436241	NM_152437 // ZNF664 /// ENST00000337815 // ZNF664 /// BC051696 // ZNF664	ZNF664	chr12	0.0330889	1.69962
3479230	NM_006231 // POLE /// ENST00000320574 // POLE /// ENST00000455752 // POLE /// BC	POLE	chr12	0.0372827	1.69921
3475216	NM_032590 // KDM2B /// NM_001005366 // KDM2B /// ENST00000377071 // KDM2B /// EN	KDM2B	chr12	0.0153746	1.69001
3456964	NM_001098815 // KIAA0748 /// AK303223 // KIAA0748 /// ENST00000449076 // KIAA074	KIAA0748	chr12	0.000197028	1.68972
3458923	NM_005730 // CTDSP2 /// ENST00000398073 // CTDSP2 /// AF000152 // CTDSP2 /// ENS	CTDSP2	chr12	0.0424469	1.68802
3454844	NM_016293 // BIN2 /// ENST00000267012 // BIN2 /// BC047686 // BIN2 /// ENST0000C	BIN2	chr12	0.0308211	1.68465
3428386	NM_178826 // ANO4 /// ENST00000392979 // ANO4 /// AK293810 // ANO4 /// ENST0000C	ANO4	chr12	0.0198133	1.67299
3469649	NM_014840 // NUAK1 /// ENST00000261402 // NUAK1 /// BC152462 // NUAK1 /// ENST0C	NUAK1	chr12	0.0181213	1.66381
3442442	NM_005768 // LPCAT3 /// ENST00000261407 // LPCAT3 /// BX647983 // LPCAT3	LPCAT3	chr12	0.00430176	1.66112
3457693	NM_014255 // CNPY2 /// ENST00000273308 // CNPY2 /// AY359102 // CNPY2	CNPY2	chr12	0.0379501	1.64526
3456276	BC042363 // ATF7 /// BC140006 // ATF7 /// AK302907 // ATF7	ATF7	chr12	0.0203835	1.64464
3434378	NM_176818 // GATC /// ENST00000229384 // GATC /// AK094319 // GATC	GATC	chr12	0.0325961	1.64441
3444330	NM_023921 // TAS2R10 /// ENST00000240619 // TAS2R10 /// BC101762 // TAS2R1C	TAS2R10	chr12	0.0360091	1.64379
3472096	NM_001024662 // RPL6 /// NM_000970 // RPL6 /// ENST00000202773 // RPL6 /// ENSTC	RPL6	chr12	0.0499134	1.63788
3417869	NM_002332 // LRP1 /// ENST00000243077 // LRP1	LRP1	chr12	0.0013126	1.63177
3408023	NM_018638 // ETNK1 /// NM_001039481 // ETNK1 /// ENST00000266517 // ETNK1 /// EN	ETNK1	chr12	0.0207745	1.62731
3427836	NM_005888 // SLC25A3 /// NM_002635 // SLC25A3 /// NM_213611 // SLC25A3 /// ENSTC	SLC25A3	chr12	0.0150041	1.61399
3460168	NM_002076 // GNS /// ENST00000258145 // GNS /// ENST00000418919 // GNS /// ENSTC	GNS	chr12	0.0404207	1.61041
3429432	NM_013320 // HCFC2 /// ENST00000229330 // HCFC2 /// AF117210 // HCFC2	HCFC2	chr12	0.0386097	1.60072
3427355	NM_001135175 // NEDD1 /// ENST00000457368 // NEDD1	NEDD1	chr12	0.00238058	1.59867
3454595	NM_001174125 // SLC11A2 /// NM_001174126 // SLC11A2 /// NM_001174127 // SLC11A2	SLC11A2	chr12	0.0151791	1.59276
3410244	NR_026806 // FLJ13224 /// ENST00000313737 // FLJ13224	FLJ13224	chr12	0.00544472	1.59113
3474455	NM_000928 // PLA2G1B /// ENST00000308366 // PLA2G1B /// BC106725 // PLA2G1E	PLA2G1B	chr12	0.0186568	1.59088
3440068	NM_172364 // CACNA2D4 /// ENST00000280663 // CACNA2D4 /// ENST00000382722 // CAC	CACNA2D4	chr12	0.0134449	1.58858
3457287	NM_002429 // MMP19 /// ENST00000322569 // MMP19 /// U37791 // MMP19 /// ENST0000C	MMP19	chr12	0.00487515	1.57993
3447354	NM_006940 // SOX5 /// NM_152989 // SOX5 /// NM_178010 // SOX5 /// ENST00000309355	SOX5	chr12	0.026728	1.57403
3474553	---	0	chr12	0.00512604	1.56638
3459979	NM_152440 // C12orf66 /// ENST00000398055 // C12orf66 /// ENST00000311915 // C12	C12orf66	chr12	0.0332932	1.56331
3457117	NM_001144996 // ITGA7 /// NM_002206 // ITGA7 /// NM_001144997 // ITGA7 /// ENSTC	ITGA7	chr12	0.0287704	1.56206

3453346	NM_004818 // DDX23 /// ENST00000308025 // DDX23 /// BC002366 // DDX23 /// ENST0(	DDX23	chr12	0.0130775	1.5421
3443005	NM_018088 // FAM90A1 /// BC042608 // FAM90A1	FAM90A1	chr12	0.0226161	1.53738
3453662	NM_003482 // MLL2 /// ENST00000301067 // MLL2 /// AF010403 // MLL2	MLL2	chr12	0.00111673	1.53531
3406887	NM_004570 // PIK3C2G /// ENST00000433979 // PIK3C2G /// BC144134 // PIK3C2G ///	PIK3C2G	chr12	0.0115588	1.53093
3402472	NM_002342 // LTBR /// ENST00000228918 // LTBR /// BC026262 // LTBR	LTBR	chr12	0.0170644	1.52887
3405754	NM_001423 // EMP1 /// ENST00000256951 // EMP1 /// U77085 // EMP1	EMP1	chr12	0.000160421	1.52527
3412024	NM_201515 // PPHLN1 /// NM_201440 // PPHLN1 /// NM_201438 // PPHLN1 /// ENST0000	PPHLN1	chr12	0.0478754	1.52519
3457860	NM_003920 // TIMELESS /// ENST00000229201 // TIMELESS /// BC050557 // TIMELESS /	TIMELESS	chr12	0.0218181	1.52136
3425633	AK058107 // C12orf37 /// ENST00000359455 // C12orf37	C12orf37	chr12	0.0492216	1.52047
3461866	NM_001109754 // PTPRB /// NM_002837 // PTPRB /// ENST00000334414 // PTPRB /// EN	PTPRB	chr12	0.0353127	1.51743
3458162	NM_000946 // PRIM1 /// ENST00000338193 // PRIM1 /// BC005266 // PRIM1	PRIM1	chr12	0.0385024	1.51607
3415276	NM_021934 // C12orf44 /// ENST00000336854 // C12orf44 /// BC005151 // C12orf44	C12orf44	chr12	0.0190266	1.51029
3453646	NM_003482 // MLL2 /// ENST00000301067 // MLL2 /// AF010403 // MLL2	MLL2	chr12	0.0117473	1.50959
3415817	NM_001004304 // ZNF740 /// ENST00000416904 // ZNF740 /// BC053557 // ZNF740 ///	ZNF740	chr12	0.014258	1.50667
3446887	NM_002300 // LDHB /// NM_001174097 // LDHB /// ENST00000350669 // LDHB /// ENST(	LDHB	chr12	0.0333632	1.50599
3447934	NM_001145728 // IFLTD1 /// NM_152590 // IFLTD1 /// NR_027296 // IFLTD1 /// ENST(	IFLTD1	chr12	0.000686051	1.50526
3476547	NM_006312 // NCOR2 /// NM_001077261 // NCOR2 /// ENST00000356219 // NCOR2 /// EN	NCOR2	chr12	0.00340513	1.50283
3507771	AB064667 // OK/SW-CL.58	OK	chr13	0.00330283	3.69709
3501359	NM_001846 // COL4A2 /// ENST00000360467 // COL4A2 /// AK294920 // COL4A2 /// ENS	COL4A2	chr13	0.00466402	2.30006
3499135	NM_004791 // ITGBL1 /// ENST00000376180 // ITGBL1 /// AF072752 // ITGBL1	ITGBL1	chr13	0.00106069	2.28431
3482131	NM_030979 // PABPC3 /// ENST00000281589 // PABPC3 /// AF132026 // PABPC3	PABPC3	chr13	0.000371326	1.98426
3510168	NM_016179 // TRPC4 /// NM_001135955 // TRPC4 /// NM_001135957 // TRPC4 /// NM_0(	TRPC4	chr13	0.00932123	1.95759
3526459	AK300897 // GRTP1 /// ENST00000326039 // GRTP1	GRTP1	chr13	0.0160453	1.86636
3484926	NM_004795 // KL /// ENST00000380099 // KL /// AB005142 // KL	KL	chr13	0.00205191	1.77432
3501750	NM_003899 // ARHGEF7 /// NM_001113513 // ARHGEF7 /// ENST00000375736 // ARHGEF7	ARHGEF7	chr13	0.00307407	1.73955
3510098	NM_006475 // POSTN /// NM_001135934 // POSTN /// NM_001135935 // POSTN /// NM_0(	POSTN	chr13	0.0171246	1.7366
3496529	NM_004466 // GPC5 /// ENST00000377067 // GPC5 /// BC039730 // GPC5	GPC5	chr13	0.0183536	1.73634
3502675	NM_017905 // TMCO3 /// ENST00000434316 // TMCO3 /// BC068515 // TMCO3	TMCO3	chr13	0.0419096	1.72925
3522362	NM_005073 // SLC15A1 /// ENST00000376503 // SLC15A1 /// U21936 // SLC15A1	SLC15A1	chr13	0.0339973	1.69122
3506096	NM_152704 // FAM123A /// NM_199138 // FAM123A /// ENST00000357816 // FAM123A //.	FAM123A	chr13	0.0330287	1.65484
3489277	NM_001079673 // FNDC3A /// NM_014923 // FNDC3A /// ENST00000337156 // FNDC3A //.	FNDC3A	chr13	0.0193322	1.59847
3518636	NM_015057 // MYCBP2 /// ENST00000357337 // MYCBP2 /// ENST00000407578 // MYCBP2	MYCBP2	chr13	0.00506501	1.59083
3501737	NM_003899 // ARHGEF7 /// NM_145735 // ARHGEF7 /// NM_001113511 // ARHGEF7 /// NM	ARHGEF7	chr13	0.00486271	1.58192
3502547	NM_001008895 // CUL4A /// NM_003589 // CUL4A /// ENST00000375440 // CUL4A /// EN	CUL4A	chr13	0.0409243	1.57931
3490439	NM_031290 // CCDC70 /// ENST00000242819 // CCDC70 /// BC069770 // CCDC70	CCDC70	chr13	0.0270253	1.57579
3489299	NM_001079673 // FNDC3A /// NM_014923 // FNDC3A /// ENST00000337156 // FNDC3A //.	FNDC3A	chr13	0.0135251	1.55148
3501489	NM_198219 // ING1 /// NM_198218 // ING1 /// NM_198217 // ING1 /// NM_005537 //	ING1	chr13	0.0458977	1.53069
3524629	BC071587 // ARGLU1 /// ENST00000375928 // ARGLU1 /// ENST00000375926 // ARGLU1 /	ARGLU1	chr13	0.0354482	1.52993
3525506	NM_017817 // RAB20 /// ENST00000267328 // RAB20 /// BC026025 // RAB20	RAB20	chr13	0.042492	1.52657
3486149	NM_207361 // FREM2 /// ENST00000280481 // FREM2	FREM2	chr13	0.0150781	1.50537
3514861	NM_002498 // NEK3 /// NM_152720 // NEK3 /// NM_001146099 // NEK3 /// NR_027415 .	NEK3	chr13	0.0022395	1.50175
3529703	NM_006084 // IRF9 /// ENST00000396864 // IRF9 /// M87503 // IRF9	IRF9	chr14	0.00350048	4.15274
3530016	NM_015299 // KHNYN /// ENST00000251343 // KIAA0323 /// AK291680 // KHNYN	KHNYN	chr14	0.0148785	3.05619
3549094	NM_001275 // CHGA /// ENST00000216492 // CHGA /// BC006459 // CHGA /// ENST00000	CHGA	chr14	0.0241459	2.283
3531034	NM_016106 // SCFD1 /// NM_182835 // SCFD1 /// ENST00000458591 // SCFD1 /// ENST(	SCFD1	chr14	0.0132483	2.19068
3536710	NM_002306 // LGALS3 /// NM_001177388 // LGALS3 /// ENST00000254301 // LGALS3	LGALS3	chr14	0.0476608	1.97707
3556839	NM_003982 // SLC7A7 /// NM_001126105 // SLC7A7 /// NM_001126106 // SLC7A7 /// EN	SLC7A7	chr14	0.0190442	1.93388
3581169	NM_005163 // AKT1 /// NM_001014432 // AKT1 /// NM_001014431 // AKT1 /// ENST0000	AKT1	chr14	0.00418929	1.92755
3557239	NM_182728 // SLC7A8 /// ENST00000397310 // SLC7A8 /// ENST00000453702 // SLC7A8	SLC7A8	chr14	0.0473305	1.89285
3545745	NM_004796 // NRXN3 /// BC152457 // NRXN3 /// AJ316284 // NRXN3 /// ENST0000033(	NRXN3	chr14	0.00679611	1.84423
3577171	NM_014216 // ITPK1 /// NM_001142593 // ITPK1 /// NM_001142594 // ITPK1 /// ENST(	ITPK1	chr14	0.00787782	1.73589

3529715	NM_006084 // IRF9 /// ENST00000396864 // IRF9 /// M87503 // IRF9 /// ENST0000032	IRF9	chr14	0.00557481	1.73382
3535006	NM_001663 // ARF6 /// ENST00000298316 // ARF6 /// BC008918 // ARF6	ARF6	chr14	0.00694321	1.69888
3580771	NM_001823 // CKB /// ENST00000348956 // CKB /// AK290101 // CKB /// ENST0000042€	CKB	chr14	0.0112398	1.68294
3570607	NM_033141 // MAP3K9 /// ENST00000005198 // MAP3K9 /// AY327900 // MAP3K9 /// ENS	MAP3K9	chr14	0.0264903	1.65681
3535128	NM_001003803 // ATP5S /// NM_001003805 // ATP5S /// NM_015684 // ATP5S /// ENST(	ATP5S	chr14	0.00755309	1.65023
3564091	NM_182944 // NIN /// NM_020921 // NIN /// NM_182946 // NIN /// NM_016350 // NIN	NIN	chr14	0.0101523	1.63472
3571250	NM_021260 // ZFYVE1 /// NM_178441 // ZFYVE1 /// ENST00000318876 // ZFYVE1 /// EN	ZFYVE1	chr14	0.0142096	1.61819
3557918	NM_006405 // TM9SF1 /// NM_001014842 // TM9SF1 /// ENST00000396854 // TM9SF1 //.	TM9SF1	chr14	0.0435581	1.61685
3538790	NM_001172702 // SLC38A6 /// NM_153811 // SLC38A6 /// ENST00000267488 // SLC38A6	SLC38A6	chr14	0.0207232	1.61225
3533459	NM_002687 // PNN /// ENST00000216832 // PNN /// AK292579 // PNN	PNN	chr14	0.033412	1.60916
3567403	NM_017420 // SIX4 /// ENST00000216513 // SIX4 /// BC101934 // SIX4	SIX4	chr14	0.016281	1.60881
3538105	NM_016651 // DACT1 /// NM_001079520 // DACT1 /// ENST00000395153 // DACT1 /// EN	DACT1	chr14	0.00858921	1.58303
3543956	NM_182476 // COQ6 /// NM_182480 // COQ6 /// ENST00000334571 // COQ6 /// ENST0000	COQ6	chr14	0.00960085	1.574
3553548	NM_006291 // TNFAIP2 /// ENST00000333007 // TNFAIP2 /// M92357 // TNFAIP2 /// EN	TNFAIP2	chr14	0.0114784	1.55271
3572250	NM_001040108 // MLH3 /// NM_014381 // MLH3 /// ENST00000355774 // MLH3 /// ENST(	MLH3	chr14	0.00538028	1.54614
3550251	NR_023938 // C14orf132	C14orf132	chr14	0.0470418	1.54305
3553071	BC030654 // WDR20	WDR20	chr14	0.0336582	1.53672
3549530	NM_023112 // OTUB2 /// ENST00000203664 // OTUB2 /// AK025569 // OTUB2	OTUB2	chr14	0.00792619	1.53365
3561961	NM_006364 // SEC23A /// ENST00000307712 // SEC23A /// BC036649 // SEC23A	SEC23A	chr14	0.0147788	1.52946
3548811	NM_017437 // CPSF2 /// ENST00000298875 // CPSF2 /// BC070095 // CPSF2 /// ENST0(	CPSF2	chr14	0.0168441	1.52353
3555466	NM_017807 // OSGEP /// ENST00000206542 // OSGEP /// BC032310 // OSGEP	OSGEP	chr14	0.00146443	1.51497
3568208	NM_001437 // ESR2 /// NM_001040275 // ESR2 /// NM_001040276 // ESR2 /// ENST000(	ESR2	chr14	0.0137006	1.51473
3549807	NR_015340 // SERPINA13 /// AY358238 // SERPINA13 /// ENST00000338704 // SERPINA1	SERPINA13	chr14	0.00192522	1.51258
3556164	---	0	chr14	0.0468885	1.50697
3529656	NM_017999 // RNF31 /// ENST00000324103 // RNF31 /// AK291247 // RNF31 /// ENST0(	RNF31	chr14	0.0451661	1.50571
3629650	NM_004884 // IGDCC3 /// ENST00000327987 // IGDCC3 /// BC042054 // IGDCC3 /// ENS	IGDCC3	chr15	0.0481344	2.6461
3594058	NM_014548 // TMOD2 /// NM_001142885 // TMOD2 /// ENST00000435126 // TMOD2 /// EN	TMOD2	chr15	0.00125032	2.00493
3615608	NM_003257 // TJP1 /// NM_175610 // TJP1 /// ENST00000346128 // TJP1 /// ENST000(	TJP1	chr15	0.0209694	1.77638
3620933	NM_174916 // UBR1 /// ENST00000290650 // UBR1 /// BC113505 // UBR1 /// ENST0000(	UBR1	chr15	0.00406937	1.70514
3619148	NM_007223 // GPR176 /// ENST00000299092 // GPR176	GPR176	chr15	0.0231135	1.68922
3608142	NM_003870 // IQGAP1 /// ENST00000268182 // IQGAP1 /// BC151834 // IQGAP1	IQGAP1	chr15	0.0373337	1.6869
3629657	NM_020962 // IGDCC4 /// ENST00000352385 // IGDCC4 /// AB052622 // IGDCC4 /// ENS	IGDCC4	chr15	0.0306832	1.67489
3604211	NM_018689 // KIAA1199 /// ENST00000394685 // KIAA1199 /// ENST00000356249 // KIA	KIAA1199	chr15	0.00596707	1.65752
3634777	NM_014272 // ADAMTS7 /// ENST00000388820 // ADAMTS7 /// BC061631 // ADAMTS7 ///	ADAMTS7	chr15	0.0125695	1.64148
3589851	NM_033510 // DISP2 /// ENST00000267889 // DISP2 /// AB051529 // DISP2	DISP2	chr15	0.0343011	1.63704
3602126	NM_015492 // C15orf39 /// ENST00000360639 // C15orf39 /// ENST00000394987 // C15	C15orf39	chr15	0.0324336	1.62297
3590217	NM_020857 // VPS18 /// ENST00000220509 // VPS18 /// AF308802 // VPS18	VPS18	chr15	0.0237266	1.61777
3641695	NM_139057 // ADAMTS17 /// ENST00000268070 // ADAMTS17 /// ENST00000378898 // ADA	ADAMTS17	chr15	0.0195409	1.61703
3608515	NM_006122 // MAN2A2 /// ENST00000360468 // MAN2A2 /// L28821 // MAN2A2 /// ENST(	MAN2A2	chr15	0.0149597	1.60817
3638197	NM_178232 // HAPLN3 /// ENST00000359595 // HAPLN3 /// BC062320 // HAPLN3	HAPLN3	chr15	0.0466491	1.56956
3589475	NM_003246 // THBS1 /// ENST00000260356 // THBS1 /// BC136469 // THBS1 /// ENST0(	THBS1	chr15	0.0231141	1.56705
3619620	NM_018145 // FAM82A2 /// ENST00000338376 // FAM82A2 /// ENST00000260385 // FAM82	FAM82A2	chr15	0.0189671	1.56693
3624693	NM_000259 // MYO5A /// NM_001142495 // MYO5A /// ENST00000399231 // MYO5A /// EN	MYO5A	chr15	0.0424276	1.55999
3613353	NM_144599 // NIPA1 /// NM_001142275 // NIPA1 /// ENST00000337435 // NIPA1 /// EN	NIPA1	chr15	0.014953	1.55895
3622207	NM_014080 // DUOX2 /// ENST00000389039 // DUOX2 /// ENST00000267837 // DUOX2 //.	DUOX2	chr15	0.017027	1.5551
3620940	NM_174916 // UBR1 /// ENST00000290650 // UBR1 /// BC113505 // UBR1 /// ENST0000(	UBR1	chr15	0.0272906	1.55054
3620697	NM_153260 // LRRC57 /// ENST00000323443 // LRRC57 /// ENST00000397130 // LRRC57	LRRC57	chr15	0.00531016	1.54957
3642220	NM_138320 // PCSK6 /// NM_138321 // PCSK6 /// ENST00000331826 // PCSK6	PCSK6	chr15	0.0444791	1.547
3606479	NM_006738 // AKAP13 /// NM_007200 // AKAP13 /// NM_144767 // AKAP13 /// ENST000(	AKAP13	chr15	0.03841	1.54681
3633218	NM_020447 // C15orf17 /// ENST00000357635 // C15orf17 /// AK000005 // C15orf17 /	C15orf17	chr15	0.0180689	1.52981
3634086	NM_005724 // TSPAN3 /// NM_198902 // TSPAN3 /// ENST00000346495 // TSPAN3 /// EN	TSPAN3	chr15	0.00944107	1.51879

3629536	NM_003613 // CILP /// ENST00000261883 // CILP /// AF035408 // CILF	CILP	chr15	0.00234674	1.51834
3619724	NM_133639 // RHOV /// ENST00000220507 // RHOV /// BC112945 // RHOV /// ENST0000C	RHOV	chr15	0.0218141	1.51407
3611690	NM_024652 // LRRK1 /// ENST00000388948 // LRRK1 /// BC068080 // LRRK1 /// ENST0C	LRRK1	chr15	0.0287241	1.51147
3590434	NM_015138 // RTF1 /// ENST00000389629 // RTF1 /// BC015052 // RTF1 /// ENST0000C	RTF1	chr15	0.0149528	1.51037
3670955	NM_002661 // PLCG2 /// ENST00000359376 // PLCG2 /// BC007565 // PLCG2	PLCG2	chr16	0.0193495	2.82399
3677677	NM_178844 // NLRC3 /// ENST00000301749 // NLRC3 /// AY601811 // NLRC3 /// ENST0C	NLRC3	chr16	0.0093178	2.56084
3677112	NM_031948 // PRSS27 /// ENST00000302641 // PRSS27 /// BC036912 // PRSS27	PRSS27	chr16	0.0214522	2.31448
3697543	AK299348 // HYDIN	HYDIN	chr16	0.00337188	2.28651
3643998	NM_020825 // CRAMP1L /// ENST00000397412 // CRAMP1L /// ENST00000293925 // CRAMF	CRAMP1L	chr16	0.043269	2.07158
3643269	NM_138769 // RHOT2 /// ENST00000315082 // RHOT2 /// AK024450 // RHOT2	RHOT2	chr16	0.00535012	2.04405
3695317	NM_004062 // CDH16 /// ENST00000299752 // CDH16 /// ENST00000394055 // CDH16 //.	CDH16	chr16	0.00106097	1.87314
3686352	NM_015171 // XPO6 /// ENST00000304658 // XPO6 /// AK123846 // XPO6	XPO6	chr16	0.0158537	1.82949
3693332	NM_005550 // KIFC3 /// NM_001130100 // KIFC3 /// NM_001130099 // KIFC3 /// ENST(	KIFC3	chr16	0.0172893	1.78194
3662847	NM_005682 // GPR56 /// NM_201524 // GPR56 /// NM_201525 // GPR56 /// NM_0011457	GPR56	chr16	0.0246926	1.77118
3676280	NM_005061 // RPL3L /// ENST00000268661 // RPL3L /// BC050413 // RPL3L	RPL3L	chr16	0.00250621	1.7689
3666229	NM_019023 // PRMT7 /// ENST00000449359 // PRMT7 /// ENST00000339507 // PRMT7 //.	PRMT7	chr16	0.00279895	1.76761
3661066	NM_005611 // RBL2 /// ENST00000262133 // RBL2 /// BC034490 // RBL2	RBL2	chr16	0.00866928	1.76693
3696195	NM_018380 // DDX28 /// ENST00000332395 // DDX28	DDX28	chr16	0.0487672	1.75173
3657028	NM_001145808 // ITGAM /// NM_000632 // ITGAM /// ENST00000287497 // ITGAM /// JC	ITGAM	chr16	0.000131717	1.73246
3666230	NM_019023 // PRMT7 /// ENST00000449359 // PRMT7 /// ENST00000339507 // PRMT7 //.	PRMT7	chr16	0.00556413	1.732
3696083	NM_005072 // SLC12A4 /// NM_001145961 // SLC12A4 /// NM_001145962 // SLC12A4 //	SLC12A4	chr16	0.0319952	1.72978
3671746	NM_014861 // ATP2C2 /// ENST00000262429 // ATP2C2 /// AY791884 // ATP2C2 /// ENS	ATP2C2	chr16	0.0218163	1.72844
3665166	NM_025187 // C16orf70 /// ENST00000219139 // C16orf70 /// BC004556 // C16orf70	C16orf70	chr16	0.00647287	1.70269
3665273	NM_003946 // NOL3 /// ENST00000268605 // NOL3 /// AK294145 // NOL3 /// ENST0000C	NOL3	chr16	0.0169029	1.70152
3662522	NM_032206 // NLRC5 /// ENST00000262510 // NLRC5 /// AF389420 // NLRC5 /// ENST0C	NLRC5	chr16	0.00054295	1.69645
3677539	NM_005741 // ZNF263 /// D88827 // ZNF263 /// AF251050 // TIGD7	ZNF263	chr16	0.00969899	1.68647
3643135	AK054811 // FAM195A	FAM195A	chr16	0.022982	1.66976
3701300	NM_152342 // CDYL2 /// ENST00000299564 // CDYL2 /// AY273798 // CDYL2	CDYL2	chr16	0.0139387	1.66094
3657079	NM_000887 // ITGAX /// ENST00000268296 // ITGAX /// M81695 // ITGAX /// ENST0000	ITGAX	chr16	0.0174175	1.65622
3643261	NM_138769 // RHOT2 /// ENST00000315082 // RHOT2 /// AK024450 // RHOT2	RHOT2	chr16	0.0167615	1.65158
3682099	NM_002474 // MYH11 /// NM_001040114 // MYH11 /// NM_022844 // MYH11 /// NM_0010	MYH11	chr16	0.00174859	1.63116
3677841	NM_004380 // CREBBP /// NM_001079846 // CREBBP /// ENST00000382070 // CREBBP //.	CREBBP	chr16	0.0346505	1.62939
3671779	NM_014861 // ATP2C2 /// ENST00000262429 // ATP2C2 /// AY791884 // ATP2C2 /// ENS	ATP2C2	chr16	0.0293504	1.62912
3666196	NM_019023 // PRMT7 /// ENST00000449359 // PRMT7 /// ENST00000339507 // PRMT7 //.	PRMT7	chr16	0.000794496	1.62728
3704445	NM_001142864 // FAM38A /// D87071 // FAM38A /// ENST00000451779 // FAM38A /// EN	FAM38A	chr16	0.0342931	1.62182
3693609	NM_001080492 // PRSS54 /// ENST00000219301 // KLKBL4 /// BC057843 // PRSS54	PRSS54	chr16	0.0363947	1.6111
3691338	NM_002968 // SALL1 /// NM_001127892 // SALL1 /// ENST00000251020 // SALL1 /// AK	SALL1	chr16	0.0256532	1.60971
3682185	NM_001171 // ABCC6 /// ENST00000205557 // ABCC6 /// AF076622 // ABCC6 /// ENST0C	ABCC6	chr16	0.0141114	1.60354
3695833	BC008284 // C16orf48 /// NM_032140 // C16orf48 /// ENST00000243878 // C16orf48	C16orf48	chr16	0.033805	1.60025
3702715	NM_001145548 // ZDHHC7 /// ENST00000344861 // ZDHHC7 /// BC018772 // ZDHHC7	ZDHHC7	chr16	0.0214412	1.59572
3665565	NM_024519 // FAM65A /// ENST00000042381 // FAM65A /// ENST00000422602 // FAM65A	FAM65A	chr16	0.0179524	1.58711
3676136	NM_080861 // SPSB3 /// ENST00000301717 // SPSB3 /// BC065515 // SPSB3 /// ENST0C	SPSB3	chr16	0.0340161	1.58479
3672420	NM_014615 // KIAA0182 /// NM_001134473 // KIAA0182 /// ENST00000253458 // KIAA01	KIAA0182	chr16	0.0114073	1.58433
3667310	NM_012426 // SF3B3 /// ENST00000302516 // SF3B3 /// BC068974 // SF3B3	SF3B3	chr16	0.0276711	1.58426
3690498	NM_032583 // ABCC11 /// NM_033151 // ABCC11 /// NM_145186 // ABCC11 /// ENST0000	ABCC11	chr16	0.0167576	1.58133
3696321	NM_018667 // SMPD3 /// ENST00000219334 // SMPD3 /// BC041828 // SMPD3	SMPD3	chr16	0.0396657	1.57718
3698346	NM_006885 // ZFHX3 /// ENST00000268489 // ZFHX3 /// L32832 // ZFHX3	ZFHX3	chr16	0.0341004	1.57222
3677756	NM_016292 // TRAP1 /// ENST00000246957 // TRAP1 /// AF154108 // TRAP1 /// BX6482	TRAP1	chr16	0.0229194	1.56727
3678373	NM_024589 // ROGDI /// ENST00000322048 // ROGDI /// AK054707 // ROGDI	ROGDI	chr16	0.00105087	1.56702
3683969	NM_017539 // DNAH3 /// ENST00000261383 // DNAH3 /// AF494040 // DNAH3 /// ENST0C	DNAH3	chr16	0.00176763	1.56661
3671203	NM_001257 // CDH13 /// BC030653 // CDH13	CDH13	chr16	0.00482701	1.56445

3662055	NM_018233 // OGFOD1 /// ENST00000336111 // OGFOD1 /// AB046832 // OGFOD1	OGFOD1	chr16	0.0386986	1.56179
3649942	NM_004996 // ABCC1 /// NM_019862 // ABCC1 /// NM_019898 // ABCC1 /// NM_019899 /	ABCC1	chr16	0.0311108	1.56126
3676201	NM_005326 // HAGH /// NM_001040427 // HAGH /// ENST00000397353 // HAGH /// ENST0	HAGH	chr16	0.020857	1.55933
3688397	NM_001136509 // ZNF843 /// ENST00000315678 // ZNF843 /// BC036762 // ZNF843	ZNF843	chr16	0.0140922	1.55789
3665340	NM_024712 // ELMO3 /// ENST00000393997 // ELMO3 /// AK300976 // ELMO3 /// ENST00	ELMO3	chr16	0.0282661	1.55246
3703146	BC009078 // C16orf74	C16orf74	chr16	0.00342094	1.55113
3645561	NM_016639 // TNFRSF12A /// ENST00000326577 // TNFRSF12A /// BC002718 // TNFRSF12	TNFRSF12A	chr16	0.0382293	1.55107
3696547	NM_032382 // COG8 /// ENST00000306875 // COG8 /// ENST00000401849 // COG8 /// AK	COG8	chr16	0.0174949	1.54785
3695553	NM_013241 // FHOD1 /// ENST00000258201 // FHOD1 /// ENST00000438183 // FHOD1 //	FHOD1	chr16	0.0194707	1.54661
3693806	NM_018231 // SLC38A7 /// ENST00000219320 // SLC38A7 /// BC001961 // SLC38A7	SLC38A7	chr16	0.0452858	1.54066
3648343	NM_015914 // TXNDC11 /// BC018635 // TXNDC11	TXNDC11	chr16	0.0106082	1.53969
3646339	NM_015246 // MGRN1 /// NM_001142289 // MGRN1 /// NM_001142290 // MGRN1 /// NM_00	MGRN1	chr16	0.029301	1.53425
3650980	NM_001105248 // TMC5 /// NM_001105249 // TMC5 /// NM_024780 // TMC5 /// ENST0000	TMC5	chr16	0.010457	1.53422
3662830	NM_005682 // GPR56 /// NM_201524 // GPR56 /// NM_201525 // GPR56 /// NM_0011457	GPR56	chr16	0.0203932	1.53355
3644809	NM_001761 // CCNF /// ENST00000397066 // CCNF /// ENST00000293968 // CCNF	CCNF	chr16	0.0152799	1.52816
3642583	NM_024571 // SNRNP25 /// ENST00000383018 // SNRNP25 /// BC001381 // SNRNP25 ///	SNRNP25	chr16	0.0365381	1.52135
3642732	NM_032039 // ITFG3 /// AK294581 // ITFG3 /// BC032112 // ITFG3 /// BC013047 //	ITFG3	chr16	0.0401573	1.51321
3697858	NM_001030007 // AP1G1 /// NM_001128 // AP1G1 /// ENST00000299980 // AP1G1 /// EN	AP1G1	chr16	0.0191859	1.51212
3684788	NM_001802 // CDR2 /// ENST00000268383 // CDR2 /// BC017503 // CDR2	CDR2	chr16	0.00505553	1.5106
3774725	ENST00000392346 // CCDC57 /// ENST00000324808 // CCDC57 /// AK074334 // CCDC57 /	CCDC57	chr17	0.0332095	2.72322
3712994	NM_016239 // MYO15A /// ENST00000205890 // MYO15A /// AF144094 // MYO15A /// ENS	MYO15A	chr17	0.0115477	2.46421
3729847	NM_005994 // TBX2 /// ENST00000424871 // TBX2 /// ENST00000419047 // TBX2 /// AE	TBX2	chr17	0.0098622	2.26475
3729932	NM_018488 // TBX4 /// ENST00000240335 // TBX4 /// ENST00000393853 // TBX4	TBX4	chr17	0.0249306	2.13679
3719967	NM_002795 // PSMB3 /// ENST00000225426 // PSMB3 /// BC013008 // PSMB3	PSMB3	chr17	0.00736081	2.11946
3771509	NM_032134 // QRICH2 /// ENST00000262765 // QRICH2 /// BC131559 // QRICH2 /// ENS	QRICH2	chr17	0.0479786	2.06834
3740239	NM_001080779 // MYO1C /// NM_001080950 // MYO1C /// NM_033375 // MYO1C /// ENST0	MYO1C	chr17	0.038557	2.01933
3715758	NM_003170 // SUPT6H /// ENST00000314616 // SUPT6H /// AL833607 // SUPT6H	SUPT6H	chr17	0.0335998	1.98957
3774518	NM_144998 // STRA13 /// ENST00000306704 // STRA13 /// U95006 // STRA13 /// ENST0	STRA13	chr17	0.0476302	1.95127
3712709	ENST00000395774 // RAI1	RAI1	chr17	0.0228501	1.9276
3745198	NM_002472 // MYH8 /// ENST00000252173 // MYH8 /// ENST00000403437 // MYH8 /// M3	MYH8	chr17	0.0346829	1.92519
3708684	NM_000747 // CHRNB1 /// ENST00000306071 // CHRNB1 /// BC023553 // CHRNB1	CHRNB1	chr17	0.00297093	1.9236
3755589	NM_000723 // CACNB1 /// NM_199247 // CACNB1 /// NM_199248 // CACNB1 /// ENST0000	CACNB1	chr17	0.00763499	1.92188
3764355	NM_004758 // BZRAP1 /// NM_024418 // BZRAP1 /// ENST00000343736 // BZRAP1 /// EN	BZRAP1	chr17	0.0189612	1.89554
3734387	NM_007261 // CD300A /// ENST00000360141 // CD300A /// BC032352 // CD300A /// ENS	CD300A	chr17	0.00114617	1.89402
3770369	NM_139018 // CD300LF /// ENST00000326165 // CD300LF /// AF375480 // CD300LF ///	CD300LF	chr17	0.0216792	1.88627
3750381	NM_000625 // NOS2 /// ENST00000313735 // NOS2 /// AF068236 // NOS2 /// ENST00000	NOS2	chr17	0.0499356	1.86797
3713147	NM_004140 // LLGL1 /// ENST00000316843 // LLGL1 /// BC151838 // LLGL1	LLGL1	chr17	0.0108732	1.8566
3743463	NM_004422 // DVL2 /// ENST00000005340 // DVL2	DVL2	chr17	0.00474185	1.85014
3758212	NR_027254 // LOC388387 /// ENST00000431109 // LOC388387 /// ENST00000393736 // L	LOC388387	chr17	0.0487574	1.84849
3723135	NM_002390 // ADAM11 /// ENST00000200557 // ADAM11 /// AK296512 // ADAM11	ADAM11	chr17	0.04738	1.82987
3749063	NM_014683 // ULK2 /// NM_001142610 // ULK2 /// ENST00000361658 // ULK2 /// ENST0	ULK2	chr17	0.000216404	1.82513
3773709	BC064483 // C17orf56 /// AK127221 // C17orf56 /// AK128728 // C17orf56 /// ENST0	C17orf56	chr17	0.0109354	1.80375
3757594	ENST00000436535 // ZNF385C /// ENST00000453355 // ZNF385C	ZNF385C	chr17	0.00444722	1.77503
3705935	NM_001163809 // WDR81 /// NM_152348 // WDR81 /// NM_001163673 // WDR81 /// NM_00	WDR81	chr17	0.0376586	1.7519
3758041	NM_024927 // PLEKHH3 /// ENST00000293349 // PLEKHH3 /// BC052978 // PLEKHH3 ///	PLEKHH3	chr17	0.00206754	1.7433
3716889	NM_032932 // RAB11FIP4 /// ENST00000394744 // RAB11FIP4 /// ENST00000325874 // R	RAB11FIP4	chr17	0.0161923	1.73699
3726733	NM_003786 // ABCC3 /// ENST00000285238 // ABCC3 /// AF085692 // ABCC3 /// ENST00	ABCC3	chr17	0.0385679	1.72519
3762013	NM_030802 // FAM117A /// ENST00000240364 // FAM117A /// BC037572 // FAM117A	FAM117A	chr17	0.0471942	1.72498
3712976	NM_001388 // DRG2 /// ENST00000225729 // DRG2 /// AB209340 // DRG2 /// ENST00000	DRG2	chr17	0.0142488	1.72456
3709317	NM_001005273 // CHD3 /// NM_005852 // CHD3 /// NM_001005271 // CHD3 /// U91543 /	CHD3	chr17	0.00981285	1.72377
3766353	NM_020198 // CCDC47 /// ENST00000225726 // CCDC47 /// AF226054 // CCDC47 /// ENS	CCDC47	chr17	0.0484592	1.70784

3713976	NM_018242 // SLC47A1 /// ENST00000270570 // SLC47A1 /// BC010661 // SLC47A1 ///	SLC47A1	chr17	0.0313616	1.69063
3728598	NM_000502 // EPX /// ENST00000225371 // EPX	EPX	chr17	0.0310059	1.67623
3743496	NM_007278 // GABARAP /// ENST00000302386 // GABARAP /// BC106748 // GABARAF	GABARAP	chr17	0.0457233	1.67312
3743718	NM_020360 // PLSCR3 /// ENST00000324822 // PLSCR3 /// AK124006 // PLSCR3 /// ENS	PLSCR3	chr17	0.00149186	1.67118
3758800	NM_032376 // TMEM101 /// ENST00000206380 // TMEM101 /// BC007438 // TMEM101	TMEM101	chr17	0.000575658	1.66712
3755725	NM_004774 // MED1 /// ENST00000300651 // MED1 /// AF055994 // MED1 /// ENST0000C	MED1	chr17	0.00302473	1.66212
3756490	NM_181537 // KRT27 /// ENST00000301656 // KRT27 /// AJ564206 // KRT27	KRT27	chr17	0.0123817	1.64686
3720391	NM_002686 // PNMT /// ENST00000269582 // PNMT /// BC037246 // PNMT	PNMT	chr17	0.0233371	1.64607
3709576	NM_012393 // PFAS /// ENST00000314666 // PFAS /// BC146768 // PFAS	PFAS	chr17	0.0240155	1.64396
3726545	NM_017957 // EPN3 /// ENST00000268933 // EPN3 /// AK292977 // EPN3 /// ENST0000C	EPN3	chr17	0.0302189	1.63621
3708814	NM_015670 // SENP3 /// ENST00000321337 // SENP3 /// AY008763 // SENP3 /// ENST0C	SENP3	chr17	0.0257498	1.63236
3751595	NM_032854 // CORO6 /// ENST00000345068 // CORO6 /// ENST00000388767 // CORO6 //.	CORO6	chr17	0.046842	1.63033
3747797	NM_016084 // RASD1 /// ENST00000225688 // RASD1 /// AF069506 // RASD1	RASD1	chr17	0.024397	1.62733
3737238	NM_024110 // CARD14 /// ENST00000344227 // CARD14 /// AF322642 // CARD14 /// ENS	CARD14	chr17	0.048169	1.61885
3741841	NM_005173 // ATP2A3 /// NM_174955 // ATP2A3 /// NM_174956 // ATP2A3 /// NM_17495	ATP2A3	chr17	0.0382544	1.61746
3744042	NM_021210 // TRAPPC1 /// NM_001166621 // TRAPPC1 /// NR_030684 // TRAPPC1 /// NR	TRAPPC1	chr17	0.0235155	1.61494
3771630	NM_024599 // RHBDF2 /// NM_001005498 // RHBDF2 /// ENST00000313080 // RHBDF2 //.	RHBDF2	chr17	0.036778	1.61328
3720238	NM_016507 // CDK12 /// NM_015083 // CDK12 /// ENST00000430627 // CRKRS /// ENSTC	CDK12	chr17	0.00468755	1.60505
3715962	NM_016518 // PIPOX /// ENST00000323372 // PIPOX /// BC008960 // PIPOX	PIPOX	chr17	0.0174838	1.60243
3741007	NM_018128 // TSR1 /// ENST00000301364 // TSR1 /// BC110851 // TSR1	TSR1	chr17	0.0191955	1.60072
3741209	NM_015229 // KIAA0664 /// ENST00000435359 // KIAA0664 /// ENST00000322335 // KIA	KIAA0664	chr17	0.0486214	1.59639
3759443	NM_002055 // GFAP /// NM_001131019 // GFAP /// ENST00000253408 // GFAP /// ENSTC	GFAP	chr17	0.00888691	1.59465
3718280	NM_207313 // TMEM132E /// ENST00000321639 // TMEM132E	TMEM132E	chr17	0.0362617	1.58728
3709395	NM_000180 // GUCY2D /// ENST00000254854 // GUCY2D /// M92432 // GUCY2D	GUCY2D	chr17	0.0402972	1.5853
3733607	NM_000346 // SOX9 /// ENST00000245479 // SOX9 /// BC056420 // SOX9	SOX9	chr17	0.00694054	1.58112
3738096	NM_031945 // TSPAN10 /// ENST00000328585 // TSPAN10 /// BC021923 // TSPAN10	TSPAN10	chr17	0.0489227	1.5768
3745291	NM_005963 // MYH1 /// ENST00000226207 // MYH1 /// BC114545 // MYH1 /// ENST0000C	MYH1	chr17	0.0169686	1.57432
3765268	NM_032582 // USP32 /// ENST00000300896 // USP32 /// AF533230 // USP32 /// ENST0C	USP32	chr17	0.00236591	1.5741
3743082	NM_031220 // PITPNM3 /// NM_001165966 // PITPNM3 /// ENST00000262483 // PITPNM3	PITPNM3	chr17	0.0104831	1.56505
3773727	BC064483 // C17orf56 /// AK127221 // C17orf56 /// AK128728 // C17orf56 /// ENSTC	C17orf56	chr17	0.0458981	1.56481
3740306	NM_006224 // PITPNA /// ENST00000313486 // PITPNA /// BC045108 // PITPNA	PITPNA	chr17	0.0173063	1.56216
3757387	NM_006455 // SC65 /// ENST00000355468 // SC65 /// ENST00000393928 // SC65 /// U4	SC65	chr17	0.0228498	1.56127
3740319	NM_006224 // PITPNA /// ENST00000313486 // PITPNA /// BC045108 // PITPNA	PITPNA	chr17	0.0153674	1.56005
3770448	NM_000835 // GRIN2C /// ENST00000293190 // GRIN2C /// U77782 // GRIN2C /// ENSTC	GRIN2C	chr17	0.045337	1.55577
3734922	NM_004524 // LLGL2 /// NM_001015002 // LLGL2 /// NM_001031803 // LLGL2 /// ENSTC	LLGL2	chr17	0.0193913	1.55428
3734958	NM_004524 // LLGL2 /// NM_001031803 // LLGL2 /// ENST00000167462 // LLGL2 /// EN	LLGL2	chr17	0.0468275	1.5534
3745239	NM_017533 // MYH4 /// ENST00000255381 // MYH4 /// AF111783 // MYH4	MYH4	chr17	0.00791599	1.55268
3721011	NM_001552 // IGFBP4 /// ENST00000269593 // IGFBP4 /// BC016041 // IGFBP4	IGFBP4	chr17	0.0116462	1.54977
3771568	NM_022066 // UBE2O /// ENST00000319380 // UBE2O	UBE2O	chr17	0.0174206	1.54965
3749646	---	0	chr17	0.00335921	1.54803
3768697	NM_007168 // ABCA8 /// ENST00000269080 // ABCA8 /// BC130280 // ABCA8 /// ENST0C	ABCA8	chr17	0.0390913	1.54754
3717671	NM_003457 // ZNF207 /// NM_001032293 // ZNF207 /// NM_001098507 // ZNF207 /// EN	ZNF207	chr17	0.0131551	1.54632
3735214	NM_000213 // ITGB4 /// NM_001005619 // ITGB4 /// NM_001005731 // ITGB4 /// ENSTC	ITGB4	chr17	0.0140948	1.54562
3715949	NM_016518 // PIPOX /// ENST00000323372 // PIPOX /// BC008960 // PIPOX	PIPOX	chr17	0.0454136	1.54156
3726745	NM_003786 // ABCC3 /// ENST00000285238 // ABCC3 /// AF085692 // ABCC3 /// ENST0C	ABCC3	chr17	0.0158032	1.53615
3759721	NM_003954 // MAP3K14 /// ENST00000344686 // MAP3K14 /// Y10256 // MAP3K14 /// EN	MAP3K14	chr17	0.00164028	1.53613
3708519	NM_198154 // TMEM95 /// ENST00000330767 // TMEM95 /// ENST00000389982 // TMEM95	TMEM95	chr17	0.0278647	1.52937
3758038	NM_024927 // PLEKHH3 /// ENST00000293349 // PLEKHH3 /// BC052978 // PLEKHH3 ///	PLEKHH3	chr17	0.0211189	1.52777
3721855	NM_025233 // COASY /// ENST00000393818 // COASY /// BC067254 // COASY	COASY	chr17	0.0248764	1.52216
3757885	NM_139276 // STAT3 /// NM_003150 // STAT3 /// NM_213662 // STAT3 /// ENST0000026	STAT3	chr17	0.0432286	1.51635
3766542	NM_000626 // CD79B /// NM_001039933 // CD79B /// ENST00000006750 // CD79B /// EN	CD79B	chr17	0.0350832	1.51439

3762049	NM_170685 // TAC4 /// NM_001077506 // TAC4 /// NM_001077503 // TAC4 /// NM_00107	TAC4	chr17	0.0164211	1.51321
3737414	NM_020914 // RNF213 /// ENST00000336301 // RNF213 /// ENST00000427003 // RNF213	RNF213	chr17	0.00735143	1.51297
3709361	NM_053051 // CNTROB /// NM_001037144 // CNTROB /// ENST00000420934 // CNTROB ///	CNTROB	chr17	0.00322307	1.5096
3705926	NM_001163809 // WDR81 /// NM_152348 // WDR81 /// NM_001163673 // WDR81 /// NM_00	WDR81	chr17	0.0290525	1.5095
3771634	NM_024599 // RHBDF2 /// NM_001005498 // RHBDF2 /// ENST00000313080 // RHBDF2 ///	RHBDF2	chr17	0.0155171	1.50926
3727023	NM_016001 // UTP18 /// ENST00000225298 // UTP18 /// BC025276 // UTP18	UTP18	chr17	0.00131656	1.50916
3709845	NM_004822 // NTN1 /// ENST00000173229 // NTN1 /// U75586 // NTN1	NTN1	chr17	0.0358956	1.50742
3789731	NM_004852 // ONECUT2 /// ENST00000262095 // ONECUT2	ONECUT2	chr18	0.0368087	2.18775
3797411	NM_173464 // L3MBTL4 /// ENST00000400105 // L3MBTL4 /// ENST00000284898 // L3MBT	L3MBTL4	chr18	0.00161387	1.89251
3779631	NM_001142405 // SLMO1 /// NM_006553 // SLMO1 /// ENST00000440960 // SLMO1 /// EN	SLMO1	chr18	0.0224704	1.66963
3781047	AK293321 // GREB1L	GREB1L	chr18	0.0482666	1.65378
3795905	NM_017512 // ENOSF1 /// NM_001126123 // ENOSF1 /// NM_202758 // ENOSF1 /// ENST0	ENOSF1	chr18	0.0456828	1.61269
3779874	NM_032142 // CEP192 /// ENST00000325971 // CEP192 /// ENST00000399863 // CEP192	CEP192	chr18	0.0482744	1.60929
3790285	NM_006785 // MALT1 /// NM_173844 // MALT1 /// ENST00000348428 // MALT1 /// ENST0	MALT1	chr18	0.0295997	1.59923
3786592	NM_015559 // SETBP1 /// AB022660 // SETBP1 /// BC146776 // SETBP1 /// ENST000002	SETBP1	chr18	0.020325	1.56423
3809644	NM_001012515 // FECH /// NM_000140 // FECH /// ENST00000382873 // FECH /// ENST0	FECH	chr18	0.0405477	1.55002
3785034	NM_020776 // KIAA1328 /// ENST00000280020 // KIAA1328 /// AL832625 // KIAA1328 /	KIAA1328	chr18	0.00208591	1.53288
3781825	NM_198129 // LAMA3 /// NM_001127717 // LAMA3 /// ENST00000313654 // LAMA3 /// EN	LAMA3	chr18	0.0125893	1.52417
3803503	NM_001105528 // C18orf34 /// NM_198995 // C18orf34 /// ENST00000403303 // C18or	C18orf34	chr18	0.0039007	1.50895
3795099	NM_198531 // ATP9B /// ENST00000307671 // ATP9B /// BC125219 // ATP9B /// ENST00	ATP9B	chr18	0.0221805	1.50251
3824567	NM_001161357 // FCHO1 /// NM_015122 // FCHO1 /// NM_001161358 // FCHO1 /// NM_00	FCHO1	chr19	0.0160939	2.35228
3821048	NM_000527 // LDLR /// ENST00000252444 // LDLR /// ENST00000455727 // LDLR /// BX	LDLR	chr19	0.00771118	2.26488
3866177	NM_016457 // PRKD2 /// NM_001079880 // PRKD2 /// NM_001079881 // PRKD2 /// ENST0	PRKD2	chr19	0.0186171	2.25433
3847798	NM_002096 // GTF2F1 /// ENST00000394456 // GTF2F1 /// BC013007 // GTF2F1 /// ENS	GTF2F1	chr19	0.000995651	2.18617
3822730	NM_213560 // PKN1 /// ENST00000342216 // PKN1	PKN1	chr19	0.00505648	2.17963
3829695	NM_000175 // GPI /// ENST00000356487 // GPI /// BC004982 // GPI /// ENST00000415	GPI	chr19	0.0234832	2.15044
3822766	NM_213560 // PKN1 /// NM_002741 // PKN1 /// ENST00000342216 // PKN1 /// ENST0000	PKN1	chr19	0.00727416	2.11473
3837236	NM_178511 // PRR24 /// ENST00000422073 // LOC255783	PRR24	chr19	0.0176773	2.06407
3838861	NM_152359 // CPT1C /// ENST00000323446 // CPT1C /// ENST00000392518 // CPT1C ///	CPT1C	chr19	0.00149593	2.06268
3867041	NM_144577 // CCDC114 /// ENST00000315396 // CCDC114 /// ENST00000377438 // CCDC1	CCDC114	chr19	0.0310529	1.97674
3851597	NM_016145 // C19orf56 /// ENST00000222190 // C19orf56 /// AK091030 // C19orf56	C19orf56	chr19	0.0129891	1.97388
3845455	NM_138813 // ATP8B3 /// NM_001178002 // ATP8B3 /// ENST00000310127 // ATP8B3 ///	ATP8B3	chr19	0.00756317	1.9636
3837962	NM_000511 // FUT2 /// NM_001097638 // FUT2 /// ENST00000425340 // FUT2 /// ENST0	FUT2	chr19	0.00473917	1.96182
3849929	NM_015719 // COL5A3 /// ENST00000264828 // COL5A3 /// AF177941 // COL5A3	COL5A3	chr19	0.013176	1.94928
3815540	NM_002085 // GPX4 /// NM_001039847 // GPX4 /// ENST00000354171 // GPX4 /// BC039	GPX4	chr19	0.0299714	1.94507
3860021	NM_021232 // PRODH2 /// ENST00000301175 // PRODH2 /// U80018 // PRODH2 /// ENST0	PRODH2	chr19	0.0101341	1.92186
3871195	NM_002842 // PTPRH /// NM_001161440 // PTPRH /// ENST00000376350 // PTPRH /// EN	PTPRH	chr19	0.00929842	1.88347
3830841	BC110893 // C19orf55 /// NM_001039887 // C19orf55 /// ENST00000396908 // C19orf5	C19orf55	chr19	0.00683821	1.8734
3861844	NM_178820 // FBXO27 /// ENST00000292853 // FBXO27 /// BC014527 // FBXO27	FBXO27	chr19	0.002862	1.86042
3870536	NM_001145303 // TMC4 /// NM_144686 // TMC4 /// ENST00000301187 // TMC4 /// BC025	TMC4	chr19	0.0232714	1.85257
3845796	NM_018049 // PLEKHJ1 /// ENST00000326631 // PLEKHJ1 /// AK097723 // PLEKHJ1	PLEKHJ1	chr19	0.0349061	1.8275
3820467	NM_001544 // ICAM4 /// NM_022377 // ICAM4 /// NM_001039132 // ICAM4 /// ENST0000	ICAM4	chr19	0.00280274	1.82431
3852152	NM_000068 // CACNA1A /// NM_023035 // CACNA1A /// NM_001127221 // CACNA1A /// NM	CACNA1A	chr19	0.0155354	1.8194
3844985	NM_014963 // SBNO2 /// NM_001100122 // SBNO2 /// ENST00000361757 // SBNO2 /// EN	SBNO2	chr19	0.00399509	1.80259
3871147	NM_178837 // C19orf51 /// ENST00000391720 // C19orf51 /// AK093458 // C19orf51 /	C19orf51	chr19	0.0311912	1.7788
3830220	NR_028406 // FXYD5 /// ENST00000342879 // FXYD5 /// ENST00000423817 // FXYD5	FXYD5	chr19	0.00708495	1.77655
3828191	NM_003796 // C19orf2 /// NM_134447 // C19orf2 /// ENST00000312051 // C19orf2 ///	C19orf2	chr19	0.0232429	1.76957
3839254	NM_001077186 // MYH14 /// NM_024729 // MYH14 /// NM_001145809 // MYH14 /// ENST0	MYH14	chr19	0.00888966	1.76583
3864464	NM_006297 // XRCC1 /// ENST00000262887 // XRCC1 /// M36089 // XRCC1 /// ENST0000	XRCC1	chr19	0.0493132	1.75617
3850935	NM_145045 // CCDC151 /// ENST00000356392 // CCDC151 /// BC142637 // CCDC151	CCDC151	chr19	0.00278755	1.75571
3816844	NM_020170 // NCLN /// ENST00000246117 // NCLN /// BC025926 // NCLN /// ENST0000C	NCLN	chr19	0.00446188	1.74361

3868635	NM_016148 // SHANK1 /// ENST00000293441 // SHANK1 /// AF163302 // SHANK1 /// ENS	SHANK1	chr19	0.0204066	1.72824
3871020	NM_001145971 // RDH13 /// NM_138412 // RDH13 /// NR_027381 // RDH13 /// NR_02738	RDH13	chr19	0.00671396	1.72752
3855316	NM_001492 // GDF1 /// NM_021267 // LASS1 /// NM_198207 // LASS1 /// ENST00000427	GDF1	chr19	0.0256474	1.71949
3825237	NM_001100418 // C19orf60 /// NM_001100419 // C19orf60 /// ENST00000358607 // C19	C19orf60	chr19	0.0264583	1.71608
3838538	NM_153329 // ALDH16A1 /// NM_001145396 // ALDH16A1 /// ENST00000455361 // ALDH16	ALDH16A1	chr19	0.00439976	1.71428
3845716	NM_001077523 // AP3D1 /// NM_003938 // AP3D1 /// ENST00000345016 // AP3D1 /// EN	AP3D1	chr19	0.0108455	1.7133
3862769	NM_024876 // ADCK4 /// NM_001142555 // ADCK4 /// ENST00000324464 // ADCK4 /// EN	ADCK4	chr19	0.0273855	1.71184
3837323	D50924 // DHX34	DHX34	chr19	0.0301266	1.7116
3858878	NM_033103 // RHPN2 /// ENST00000254260 // RHPN2 /// BC036447 // RHPN2	RHPN2	chr19	0.0153568	1.71034
3830600	NM_015302 // HAUS5 /// ENST00000203166 // HAUS5 /// BC064390 // HAUS5 /// ENST00	HAUS5	chr19	0.0436881	1.68064
3821853	NM_004317 // ASNA1 /// ENST00000357332 // ASNA1 /// AF047469 // ASNA1 /// ENST00	ASNA1	chr19	0.019248	1.68029
3853081	NM_006844 // ILVBL /// ENST00000263383 // ILVBL /// AB209065 // ILVBL	ILVBL	chr19	0.0344183	1.67905
3844184	NM_017908 // ZNF446 /// ENST00000335841 // ZNF446 /// ENST00000391694 // ZNF446	ZNF446	chr19	0.032702	1.67224
3871368	NM_144613 // COX6B2 /// ENST00000326529 // COX6B2 /// AK057427 // COX6B2	COX6B2	chr19	0.0272478	1.66707
3848911	NM_198471 // KANK3 /// ENST00000330915 // KANK3 /// BC066124 // KANK3	KANK3	chr19	0.0139026	1.66576
3816452	NM_152988 // SPPL2B /// NM_001077238 // SPPL2B /// ENST00000452401 // SPPL2B //	SPPL2B	chr19	0.00330176	1.66173
3865345	NM_001142502 // PPP1R13L /// NM_006663 // PPP1R13L /// ENST00000418234 // PPP1R1	PPP1R13L	chr19	0.012137	1.66173
3866942	NM_000234 // LIG1 /// ENST00000263274 // LIG1 /// AB208791 // LIG1	LIG1	chr19	0.0358082	1.65424
3861416	NM_001042600 // MAP4K1 /// NM_007181 // MAP4K1 /// ENST00000396857 // MAP4K1 //	MAP4K1	chr19	0.00622646	1.65275
3842354	NM_007279 // U2AF2 /// NM_001012478 // U2AF2 /// ENST00000308924 // U2AF2 /// EN	U2AF2	chr19	0.0203814	1.6513
3837413	NM_015711 // GLTSCR1 /// ENST00000396720 // GLTSCR1 /// AF182077 // GLTSCR1	GLTSCR1	chr19	0.0313768	1.64555
3846072	NM_021217 // ZNF77 /// ENST00000314531 // ZNF77 /// BC043354 // ZNF77 /// ENST00	ZNF77	chr19	0.0367921	1.63584
3851397	NM_152601 // ZNF709 /// BC136746 // ZNF709 /// ENST00000455490 // ZNF564	ZNF709	chr19	0.0271466	1.62152
3815661	NM_001280 // CIRBP /// NR_023312 // CIRBP /// NR_023313 // CIRBP /// ENST0000032	CIRBP	chr19	0.0287555	1.61994
3865513	NM_012155 // EML2 /// ENST00000245925 // EML2 /// AK294668 // EML2	EML2	chr19	0.0443074	1.6142
3841633	NM_006847 // LILRB4 /// NM_001081438 // LILRB4 /// ENST00000270452 // LILRB4 //	LILRB4	chr19	0.0430738	1.61288
3830690	NM_014383 // ZBTB32 /// ENST00000262630 // ZBTB32 /// ENST00000392197 // ZBTB32	ZBTB32	chr19	0.014474	1.61286
3837847	NM_017457 // CYTH2 /// NM_004228 // CYTH2 /// ENST00000452733 // CYTH2 /// ENST0	CYTH2	chr19	0.0165507	1.61124
3845742	NM_001077523 // AP3D1 /// NM_003938 // AP3D1 /// ENST00000345016 // AP3D1 /// EN	AP3D1	chr19	0.0097768	1.60999
3867489	NM_020904 // PLEKHA4 /// NM_001161354 // PLEKHA4 /// ENST00000263265 // PLEKHA4	PLEKHA4	chr19	0.013102	1.60682
3825761	NM_017660 // GATAD2A /// ENST00000360315 // GATAD2A /// ENST00000358713 // GATAD	GATAD2A	chr19	0.016062	1.60544
3853366	NM_014371 // AKAP8L /// ENST00000397410 // AKAP8L /// AK001594 // AKAP8L /// ENS	AKAP8L	chr19	0.0121766	1.60369
3822524	NM_138353 // DCAF15 /// ENST00000254337 // DCAF15	DCAF15	chr19	0.00133521	1.59933
3837842	NM_017457 // CYTH2 /// NM_004228 // CYTH2 /// ENST00000452733 // CYTH2 /// ENST0	CYTH2	chr19	0.020496	1.59792
3859918	NM_001040425 // U2AF1L4 /// NM_144987 // U2AF1L4 /// ENST00000292879 // U2AF1L4	U2AF1L4	chr19	0.0343277	1.5963
3833900	NR_001278 // CYP2B7P1 /// M29873 // CYP2B7P1	CYP2B7P1	chr19	0.0224409	1.59404
3860303	NM_001864 // COX7A1 /// ENST00000292907 // COX7A1 /// BC002757 // COX7A1	COX7A1	chr19	0.00992207	1.58916
3868167	NM_032375 // AKT1S1 /// NM_001098632 // AKT1S1 /// NM_001098633 // AKT1S1 /// EN	AKT1S1	chr19	0.0150832	1.58855
3848264	NM_000208 // INSR /// NM_001079817 // INSR /// ENST00000302850 // INSR /// ENST0	INSR	chr19	0.0356682	1.58221
3837913	NM_020126 // SPHK2 /// ENST00000245222 // SPHK2 /// EF107108 // SPHK2 /// ENST00	SPHK2	chr19	0.0299703	1.57931
3851551	NM_000528 // MAN2B1 /// NM_001173498 // MAN2B1 /// ENST00000456935 // MAN2B1 //	MAN2B1	chr19	0.0246759	1.57819
3845536	NM_020695 // REXO1 /// ENST00000170168 // REXO1 /// BC032244 // REXO1 /// ENST00	REXO1	chr19	0.0223208	1.57345
3862482	NM_001626 // AKT2 /// ENST00000392038 // AKT2 /// BC063421 // AKT2 /// AK122839	AKT2	chr19	0.00503682	1.5717
3847612	NM_000635 // RFX2 /// NM_134433 // RFX2 /// ENST00000303657 // RFX2 /// ENST0000	RFX2	chr19	0.0397076	1.57015
3859925	NM_001040425 // U2AF1L4 /// NM_144987 // U2AF1L4 /// ENST00000292879 // U2AF1L4	U2AF1L4	chr19	0.0425035	1.56371
3838772	NM_021228 // SCAF1 /// ENST00000360565 // SCAF1 /// ENST00000447618 // SCAF1 //	SCAF1	chr19	0.04612	1.56263
3834355	NM_004363 // CEACAM5 /// ENST00000446434 // CEACAM5 /// ENST00000221992 // CEACA	CEACAM5	chr19	0.0238235	1.5623
3837878	NM_004605 // SULT2B1 /// NM_177973 // SULT2B1 /// ENST00000201586 // SULT2B1 //	SULT2B1	chr19	0.00259238	1.55662
3864729	NM_003425 // ZNF45 /// ENST00000269973 // ZNF45 /// ENST00000418980 // ZNF45 //	ZNF45	chr19	0.0314063	1.55566
3866607	NM_003827 // NAPA /// ENST00000263354 // NAPA /// AK126519 // NAPA	NAPA	chr19	0.020838	1.55512
3855244	NM_000095 // COMP /// ENST00000222271 // COMP /// BC033676 // COMP /// ENST0000	COMP	chr19	0.0166839	1.55335

3817002	NM_001136198 // FZR1 /// NM_016263 // FZR1 /// ENST00000395095 // FZR1 /// ENST(	FZR1	chr19	0.0169017	1.55132
3862809	NM_198476 // C19orf54 /// ENST00000378313 // C19orf54 /// AK298068 // C19orf54 /	C19orf54	chr19	0.012717	1.5494
3820336	NM_018381 // C19orf66 /// ENST00000253110 // C19orf66 /// AK096142 // C19orf66 /	C19orf66	chr19	0.0410093	1.54893
3865468	NM_001017989 // OPA3 /// ENST00000323060 // OPA3 /// AK098798 // OPA3	OPA3	chr19	0.0219448	1.54883
3868604	NM_016148 // SHANK1 /// ENST00000293441 // SHANK1 /// AF163302 // SHANK1 /// AF2	SHANK1	chr19	0.0435059	1.54542
3821984	NM_014975 // MAST1 /// ENST00000251472 // MAST1	MAST1	chr19	0.0363772	1.5419
3816855	NM_020170 // NCLN /// ENST00000246117 // NCLN /// BC025926 // NCLN /// ENST00000(	NCLN	chr19	0.0272846	1.53937
3861383	NM_170604 // RASGRP4 /// NM_001146202 // RASGRP4 /// NM_001146203 // RASGRP4 //.	RASGRP4	chr19	0.00117344	1.53752
3827482	NM_203282 // ZNF254 /// ENST00000357002 // ZNF254 /// BC043147 // ZNF254 /// ENS	ZNF254	chr19	0.0362799	1.53601
3841886	NM_004829 // NCR1 /// NM_001145457 // NCR1 /// NM_001145458 // NCR1 /// NR_02704	NCR1	chr19	0.0244387	1.53593
3861560	NM_006149 // LGALS4 /// ENST00000307751 // LGALS4 /// BC005146 // LGALS4	LGALS4	chr19	0.0305526	1.53391
3832596	NM_000540 // RYR1 /// NM_001042723 // RYR1 /// ENST00000359596 // RYR1 /// ENST(	RYR1	chr19	0.0249755	1.53354
3847160	NM_002850 // PTPRS /// NM_130854 // PTPRS /// NM_130853 // PTPRS /// NM_130855 /	PTPRS	chr19	0.0281024	1.52792
3846993	NM_182919 // TICAM1 /// ENST00000248244 // TICAM1 /// AB086380 // TICAM1 /// ENS	TICAM1	chr19	0.0338451	1.52679
3830730	NM_014727 // MLL4 /// ENST00000420124 // MLL4 /// ENST00000222270 // MLL4 /// EN	MLL4	chr19	0.00131774	1.52634
3842116	NM_032701 // SUV420H2 /// ENST00000255613 // SUV420H2 /// BC044889 // SUV420H2 /	SUV420H2	chr19	0.0215987	1.52579
3821896	NM_002229 // JUNB /// ENST00000302754 // JUNB /// BC004250 // JUNB	JUNB	chr19	0.0423102	1.52475
3859844	NM_000704 // ATP4A /// ENST00000262623 // ATP4A /// AK223259 // ATP4A	ATP4A	chr19	0.0437377	1.51902
3824671	NM_002248 // KCNN1 /// U69883 // KCNN1 /// ENST00000222249 // KCNN1	KCNN1	chr19	0.0454235	1.51321
3861511	NM_144691 // CAPN12 /// ENST00000328867 // CAPN12 /// AK125713 // CAPN12	CAPN12	chr19	0.0343187	1.51232
3850107	NM_001130823 // DNMT1 /// NM_001379 // DNMT1 /// ENST00000340748 // DNMT1 /// EN	DNMT1	chr19	0.00193173	1.51162
3853963	NM_006387 // CHERP /// ENST00000198939 // CHERP /// BC021294 // CHERP	CHERP	chr19	0.0226316	1.5107
3870897	NM_145057 // CDC42EP5 /// ENST00000301200 // CDC42EP5 /// BC024327 // CDC42EP5	CDC42EP5	chr19	0.0428265	1.50916
3835890	NM_000041 // APOE /// ENST00000252486 // APOE /// BC003557 // APOE /// K00396 /.	APOE	chr19	0.000787761	1.5091
3838676	NM_020650 // RCN3 /// ENST00000270645 // RCN3 /// AY195859 // RCN3	RCN3	chr19	0.0236968	1.50881
3869091	NM_053003 // SIGLEC12 /// NM_033329 // SIGLEC12 /// ENST00000291707 // SIGLEC12	SIGLEC12	chr19	0.033103	1.50856
3834074	NM_021913 // AXL /// NM_001699 // AXL /// ENST00000301178 // AXL /// ENST0000035	AXL	chr19	0.00547516	1.50775
3834024	NM_030622 // CYP2S1 /// ENST00000310054 // CYP2S1 /// AY358603 // CYP2S1 /// ENS	CYP2S1	chr19	0.0257791	1.50459
3830042	NM_020895 // GRAMD1A /// NM_001136199 // GRAMD1A /// ENST00000317991 // GRAMD1A	GRAMD1A	chr19	0.0362138	1.50347
3818795	NM_015318 // ARHGEF18 /// NM_001130955 // ARHGEF18 /// ENST00000359920 // ARHGEF	ARHGEF18	chr19	0.014347	1.50299
2555335	NM_014709 // USP34 /// ENST00000398571 // USP34 /// ENST00000263989 // USP34 //.	USP34	chr2	0.0138996	3.03899
2599601	NM_017431 // PRKAG3 /// ENST00000233944 // PRKAG3 /// ENST00000439262 // PRKAG3	PRKAG3	chr2	0.0142405	2.68922
2487210	NM_032208 // ANTXR1 /// ENST00000303714 // ANTXR1 /// AF279145 // ANTXR1	ANTXR1	chr2	0.0330743	2.49
2519626	NM_000090 // COL3A1 /// ENST00000304636 // COL3A1	COL3A1	chr2	0.0331154	2.43773
2524019	NM_205863 // PARD3B /// NM_152526 // PARD3B /// NM_057177 // PARD3B /// AF42825(	PARD3B	chr2	0.0437128	2.30173
2468875	NM_003887 // ASAP2 /// NM_001135191 // ASAP2 /// ENST00000281419 // ASAP2 /// EN	ASAP2	chr2	0.0379944	2.27085
2474324	NM_080592 // C2orf28 /// ENST00000405489 // C2orf28 /// ENST00000380171 // C2orf	C2orf28	chr2	0.000901782	2.22346
2475734	NM_182551 // LCLAT1 /// ENST00000309052 // LCLAT1	LCLAT1	chr2	0.0103117	2.21577
2596560	NM_003709 // KLF7 /// ENST00000309446 // KLF7 /// BC012919 // KLF7 /// AB015132	KLF7	chr2	0.0204255	2.07352
2558780	NM_001617 // ADD2 /// NM_017482 // ADD2 /// NM_017488 // ADD2 /// ENST0000040304	ADD2	chr2	0.00725402	2.06957
2528485	NM_001927 // DES /// ENST00000373960 // DES /// BC032116 // DES	DES	chr2	0.0035737	2.04907
2604403	NM_005737 // ARL4C /// ENST00000390645 // ARL4C /// BC089043 // ARL4C /// ENST0(	ARL4C	chr2	0.0241938	1.97128
2472939	NM_181713 // UBXN2A /// ENST00000404924 // UBXN2A /// ENST00000309033 // UBXN2A	UBXN2A	chr2	0.0474378	1.96962
2491709	NM_006590 // USP39 /// ENST00000323701 // USP39 /// ENST00000409470 // USP39 //.	USP39	chr2	0.00535505	1.91377
2466938	NM_015025 // MYT1L /// ENST00000428368 // MYT1L /// BC137272 // MYT1L /// ENST0(	MYT1L	chr2	0.0433753	1.88847
2577579	NM_030923 // TMEM163 /// ENST00000281924 // TMEM163 /// BC026170 // TMEM163	TMEM163	chr2	0.0222619	1.88745
2600222	NM_024536 // CHPF /// ENST00000243776 // CHPF /// AY358403 // CHPF	CHPF	chr2	0.013395	1.87563
2566938	NM_002285 // AFF3 /// NM_001025108 // AFF3 /// ENST00000317233 // AFF3 /// ENST(	AFF3	chr2	0.0187228	1.85297
2591717	NM_000393 // COL5A2 /// ENST00000374866 // COL5A2 /// ENST00000452536 // COL5A2	COL5A2	chr2	0.00961252	1.81995
2531818	NM_025139 // ARMC9 /// ENST00000349938 // ARMC9 /// BX537956 // ARMC9 /// ENST0(	ARMC9	chr2	0.0257633	1.80393
2531330	NM_138402 // SP140L /// ENST00000444636 // SP140L /// ENST00000415673 // SP140L	SP140L	chr2	0.0307449	1.77768

2605358	NM_004369 // COL6A3 /// NM_057166 // COL6A3 /// NM_057167 // COL6A3 /// ENST0000	COL6A3	chr2	0.0212032	1.77433
2503138	NM_020909 // EPB41L5 /// ENST00000263713 // EPB41L5 /// BC054508 // EPB41L5 ///	EPB41L5	chr2	0.0149237	1.77174
2598296	NM_212482 // FN1 /// NM_212475 // FN1 /// NM_002026 // FN1 /// NM_212478 // FN1	FN1	chr2	0.00322761	1.72009
2497175	NM_003853 // IL18RAP /// ENST00000264260 // IL18RAP /// AF077346 // IL18RAP ///	IL18RAP	chr2	0.000659822	1.71848
2603998	NM_019850 // NGEF /// NM_001114090 // NGEF /// ENST00000373552 // NGEF /// ENST0	NGEF	chr2	0.000724967	1.7183
2552081	NM_022055 // KCNK12 /// ENST00000327876 // KCNK12 /// BC047749 // KCNK12	KCNK12	chr2	0.0458988	1.6987
2539141	NM_207315 // CMPK2 /// ENST00000256722 // CMPK2 /// ENST00000458098 // CMPK2 //	CMPK2	chr2	0.0296988	1.69307
2536122	NM_001080437 // SNED1 /// ENST00000310397 // SNED1 /// AF439717 // SNED1 /// ENS	SNED1	chr2	0.0384522	1.69277
2542235	NM_020905 // RDH14 /// AY358511 // RDH14	RDH14	chr2	0.00362492	1.68789
2467704	NM_016030 // TTC15 /// ENST00000324266 // TTC15 /// ENST00000382110 // TTC15 //	TTC15	chr2	0.00644116	1.68554
2476107	NM_014946 // SPAST /// NM_199436 // SPAST /// ENST00000315285 // SPAST /// ENST0	SPAST	chr2	0.0464509	1.67641
2526498	NM_024532 // SPAG16 /// ENST00000331683 // SPAG16 /// AK302351 // SPAG16 /// BC0	SPAG16	chr2	0.0378455	1.67506
2581367	NM_001005747 // CACNB4 /// NM_000726 // CACNB4 /// NM_001005746 // CACNB4 /// NM	CACNB4	chr2	0.0137576	1.6418
2467972	NM_003108 // SOX11 /// ENST00000322002 // SOX11 /// AB028641 // SOX11	SOX11	chr2	0.0338769	1.63354
2596780	NM_003468 // FZD5 /// ENST00000295417 // FZD5 /// AB043702 // FZD5	FZD5	chr2	0.0298319	1.63305
2573601	NM_014553 // TFCP2L1 /// ENST00000263707 // TFCP2L1 /// AF198488 // TFCP2L1	TFCP2L1	chr2	0.0033196	1.63259
2481156	NM_000179 // MSH6 /// ENST00000234420 // MSH6 /// U28946 // MSH6 /// AK293921 /	MSH6	chr2	0.0261329	1.62899
2584756	NM_004490 // GRB14 /// ENST00000263915 // GRB14 /// L76687 // GRB14 /// ENST0000	GRB14	chr2	0.0409266	1.6184
2563830	---	0	chr2	0.0270694	1.6146
2523245	NM_001204 // BMPR2 /// ENST00000374580 // BMPR2 /// BC052985 // BMPR2 /// ENST00	BMPR2	chr2	0.00952737	1.61342
2524144	NM_205863 // PARD3B /// NM_152526 // PARD3B /// NM_057177 // PARD3B /// ENST0000	PARD3B	chr2	0.00747392	1.61202
2565180	---	0	chr2	0.0131815	1.6036
2566034	NM_005735 // ACTR1B /// ENST00000289228 // ACTR1B /// BC010090 // ACTR1B /// ENS	ACTR1B	chr2	0.00972342	1.60033
2596778	NM_003468 // FZD5 /// ENST00000295417 // FZD5 /// AB043702 // FZD5	FZD5	chr2	0.00131382	1.59884
2538494	NM_003310 // TSSC1 /// ENST00000382125 // TSSC1 /// AF019952 // TSSC1 /// BC0024	TSSC1	chr2	0.00582114	1.59692
2491603	NM_198482 // SH2D6 /// ENST00000340326 // SH2D6 /// ENST00000355009 // SH2D6 //	SH2D6	chr2	0.0476513	1.592
2487968	NM_001115116 // ANKRD53 /// NM_024933 // ANKRD53 /// ENST00000360589 // ANKRD53	ANKRD53	chr2	0.00693604	1.58629
2574724	NM_001001665 // CYP27C1 /// ENST00000335247 // CYP27C1 /// ENST00000409327 // CY	CYP27C1	chr2	0.0370127	1.58405
2520238	NM_005966 // NAB1 /// ENST00000337386 // NAB1 /// BC035724 // NAB1 /// ENST00000	NAB1	chr2	0.000291348	1.58143
2502967	NM_001105198 // TMEM177 /// NM_030577 // TMEM177 /// NM_001105199 // TMEM177 //	TMEM177	chr2	0.00626403	1.57991
2500698	NM_153214 // FBLN7 /// ENST00000331203 // FBLN7 /// BC035784 // FBLN7 /// ENST00	FBLN7	chr2	0.00913363	1.57907
2528011	NM_015690 // STK36 /// ENST00000295709 // STK36 /// ENST00000440309 // STK36 //	STK36	chr2	0.00390291	1.57742
2595069	NM_020919 // ALS2 /// ENST00000264276 // ALS2 /// AB053305 // ALS2 /// ENST00000	ALS2	chr2	0.0297129	1.57709
2558878	NM_015717 // CD207 /// ENST00000410009 // CD207 /// ENST00000234388 // CD207 //	CD207	chr2	0.0222663	1.57065
2530532	NM_000091 // COL4A3 /// NM_031362 // COL4A3 /// ENST00000396578 // COL4A3 /// EN	COL4A3	chr2	0.000665652	1.56054
2497937	NM_182640 // MRPS9 /// ENST00000258455 // MRPS9 /// BC057240 // MRPS9	MRPS9	chr2	0.0393085	1.5573
2585974	NM_003742 // ABCB11 /// ENST00000263817 // ABCB11 /// AF091582 // ABCB11 /// ENS	ABCB11	chr2	0.0145443	1.55701
2469395	NM_134421 // HPCAL1 /// ENST00000381765 // HPCAL1 /// AK000596 // HPCAL1	HPCAL1	chr2	0.0243905	1.55652
2592300	NM_007315 // STAT1 /// NM_139266 // STAT1 /// ENST00000361099 // STAT1 /// ENST0	STAT1	chr2	0.0212461	1.55122
2559467	NM_015470 // RAB11FIP5 /// ENST00000258098 // RAB11FIP5 /// AF334812 // RAB11FIF	RAB11FIP5	chr2	0.015317	1.54358
2544702	NM_175629 // DNMT3A /// NM_153759 // DNMT3A /// NM_022552 // DNMT3A /// ENST0000	DNMT3A	chr2	0.00150559	1.5394
2571984	NM_025181 // SLC35F5 /// ENST00000245680 // SLC35F5 /// BC018537 // SLC35F5 ///	SLC35F5	chr2	0.0452741	1.5382
2543002	NM_022460 // HS1BP3 /// ENST00000304031 // HS1BP3 /// BC050636 // HS1BP3	HS1BP3	chr2	0.023698	1.53563
2466984	NM_015025 // MYT1L /// ENST00000428368 // MYT1L /// BC137272 // MYT1L /// ENST00	MYT1L	chr2	0.00885991	1.53525
2577893	NM_002299 // LCT /// ENST00000264162 // LCT /// ENST00000455227 // LCT	LCT	chr2	0.0169244	1.53419
2473290	NM_024322 // CENPO /// ENST00000380834 // CENPO /// ENST00000260662 // CENPO //	CENPO	chr2	0.0288715	1.53198
2528003	NM_015690 // STK36 /// ENST00000295709 // STK36 /// ENST00000440309 // STK36 //	STK36	chr2	0.0163351	1.53168
2521558	NM_138395 // MARS2 /// ENST00000282276 // MARS2 /// AB107013 // MARS2	MARS2	chr2	0.0288783	1.5302
2536963	AK098031 // FLJ40712	FLJ40712	chr2	0.0307832	1.5274
2491296	NM_021103 // TMSB10 /// ENST00000233143 // TMSB10 /// BC107889 // TMSB10	TMSB10	chr2	0.0110792	1.52673
2547763	NR_003143 // MYADML /// BC031088 // MYADML	MYADML	chr2	0.0158698	1.52451

2474578	NM_001168364 // KRTCAP3 /// NM_173853 // KRTCAP3 /// ENST00000288873 // KRTCAP3	KRTCAP3	chr2	0.0251477	1.52402
2491355	NM_020122 // KCMF1 /// ENST00000409785 // KCMF1 /// BC000178 // KCMF1 /// ENST0(	KCMF1	chr2	0.0446527	1.52353
2577705	ENST00000412849 // ZRANB3	ZRANB3	chr2	0.0456417	1.51692
2532964	NM_003648 // DGKD /// NM_152879 // DGKD /// ENST00000409813 // DGKD /// ENST0000(	DGKD	chr2	0.0218404	1.51529
2488899	NR_003683 // ALMS1P	ALMS1P	chr2	0.0492813	1.51336
2581177	NM_001164507 // NEB /// NM_001164508 // NEB /// NM_004543 // NEB /// ENST0000040(	NEB	chr2	0.00892543	1.51098
2573330	NR_027181 // LOC84931 /// ENST00000445555 // LOC84931	LOC84931	chr2	0.0394464	1.51039
2468848	NM_003887 // ASAP2 /// NM_001135191 // ASAP2 /// ENST00000281419 // ASAP2 /// EN	ASAP2	chr2	0.00765452	1.50833
2576537	NR_002826 // LOC401010 /// AK302229 // LOC40101(	LOC401010	chr2	0.0127854	1.5068
2534877	NM_001040445 // ASB1 /// ENST00000264607 // ASB1 /// BC014528 // ASB1 /// ENST0(	ASB1	chr2	0.0483886	1.50429
3882205	NM_025227 // BPIL1 /// ENST00000170150 // BPIL1 /// BC034415 // BPIL1 /// ENST0(	BPIL1	chr20	0.0160951	2.21424
3887176	NM_022104 // PCIF1 /// ENST00000372409 // PCIF1 /// AB050014 // PCIF1 /// ENST0(	PCIF1	chr20	0.00111972	1.97923
3894563	NM_080489 // SDCBP2 /// ENST00000339987 // SDCBP2 /// ENST00000381812 // SDCBP2	SDCBP2	chr20	0.0243933	1.90367
3901868	NM_001042472 // ABHD12 /// NM_015600 // ABHD12 /// ENST00000339157 // ABHD12 //.	ABHD12	chr20	0.0336619	1.88953
3881349	NM_178580 // HM13 /// NM_178581 // HM13 /// ENST00000398174 // HM13 /// ENST0000(	HM13	chr20	0.0238142	1.82886
3891041	NM_001001433 // STX16 /// NM_003763 // STX16 /// NM_001134772 // STX16 /// NM_0(	STX16	chr20	0.00819703	1.79353
3893594	NM_032957 // RTEL1 /// ENST00000360203 // RTEL1 /// ENST00000318100 // RTEL1 //.	RTEL1	chr20	0.000841615	1.77706
3894010	NM_004535 // MYT1 /// ENST00000328439 // MYT1	MYT1	chr20	0.0215067	1.75205
3892457	NM_198935 // SS18L1 /// ENST00000331758 // SS18L1 /// ENST00000421564 // SS18L1	SS18L1	chr20	0.0165308	1.73425
3883755	NM_012156 // EPB41L1 /// ENST00000338074 // EPB41L1 /// BC040259 // EPB41L1 ///	EPB41L1	chr20	0.00971647	1.71145
3891063	NM_024663 // NPEPL1 /// ENST00000356091 // NPEPL1 /// ENST00000371137 // NPEPL1	NPEPL1	chr20	0.00517692	1.70784
3895893	NM_000678 // ADRA1D /// ENST00000379453 // ADRA1D /// S70782 // ADRA1D	ADRA1D	chr20	0.00810251	1.68894
3903382	NM_000687 // AHCY /// NM_001161766 // AHCY /// ENST00000217426 // AHCY /// BC01(	AHCY	chr20	0.0101369	1.67789
3893090	NM_022082 // SLC17A9 /// ENST00000370351 // SLC17A9 /// ENST00000428770 // SLC17	SLC17A9	chr20	0.00988902	1.66355
3902677	NR_002781 // TSPYL3	TSPYL3	chr20	0.00589273	1.66159
3893530	NM_016434 // RTEL1 /// NM_032957 // RTEL1 /// ENST00000370018 // RTEL1 /// ENST(	RTEL1	chr20	0.0286079	1.64624
3877250	NM_024120 // C20orf7 /// NM_001039375 // C20orf7 /// NR_029377 // C20orf7 /// EN	C20orf7	chr20	0.0408361	1.64578
3902595	NM_080611 // DUSP15	DUSP15	chr20	0.0278299	1.64366
3899498	NM_001099407 // C20orf12 /// ENST00000262547 // C20orf12 /// ENST00000358866 //	C20orf12	chr20	0.00617765	1.64146
3911263	NM_199171 // PMEPA1 /// ENST00000265626 // PMEPA1	PMEPA1	chr20	0.00552908	1.63228
3899986	NM_016652 // CRNKL1 /// ENST00000377340 // CRNKL1 /// AF255443 // CRNKL1 /// ENS	CRNKL1	chr20	0.03252	1.63089
3878909	NM_018993 // RIN2 /// ENST00000255006 // RIN2 /// AK094884 // RIN2	RIN2	chr20	0.0370989	1.62771
3889854	NM_018431 // DOK5 /// ENST00000262593 // DOK5 /// ENST00000395939 // DOK5 /// AF	DOK5	chr20	0.00801232	1.60355
3873136	NM_024958 // NRSN2 /// ENST00000382291 // NRSN2 /// ENST00000246039 // NRSN2 //.	NRSN2	chr20	0.0176829	1.60197
3911259	NM_020182 // PMEPA1 /// ENST00000341744 // PMEPA1 /// AF305616 // PMEPA1 /// ENS	PMEPA1	chr20	0.020711	1.60121
3880791	NM_002862 // PYGB /// ENST00000216962 // PYGB /// BC017045 // PYGB	PYGB	chr20	0.00438681	1.5954
3883342	NM_007186 // CEP250 /// ENST00000397527 // CEP250 /// ENST00000356095 // CEP250	CEP250	chr20	0.0155039	1.56152
3895099	NM_006899 // IDH3B /// NM_174855 // IDH3B /// NM_174856 // IDH3B /// ENST0000038	IDH3B	chr20	0.0147126	1.55368
3886148	NM_016276 // SGK2 /// ENST00000341458 // SGK2 /// AF186470 // SGK2 /// AY987010	SGK2	chr20	0.0337832	1.55274
3894100	NM_153269 // C20orf96 /// ENST00000382369 // C20orf96 /// BX640715 // C20orf96 .	C20orf96	chr20	0.0351455	1.55122
3882047	NM_006892 // DNMT3B /// NM_175848 // DNMT3B /// NM_175849 // DNMT3B /// NM_17585	DNMT3B	chr20	0.013659	1.53587
3877952	NM_002594 // PCSK2 /// ENST00000262545 // PCSK2 /// BC040546 // PCSK2 /// ENST0(	PCSK2	chr20	0.0122807	1.53051
3884426	NM_080607 // VSTM2L /// ENST00000373461 // VSTM2L /// BC033818 // VSTM2L /// ENS	VSTM2L	chr20	0.0248897	1.53014
3914234	NM_020713 // ZNF512B /// ENST00000450537 // ZNF512B /// ENST00000217130 // ZNF51	ZNF512B	chr20	0.0300931	1.52743
3885606	NM_002660 // PLCG1 /// NM_182811 // PLCG1 /// ENST00000373271 // PLCG1 /// ENST(	PLCG1	chr20	0.0365695	1.52742
3891708	NM_177980 // CDH26 /// NM_021810 // CDH26 /// ENST00000350849 // CDH26 /// ENST(	CDH26	chr20	0.017333	1.52102
3902016	NM_025176 // NINL /// ENST00000278886 // NINL /// BC036380 // NINL /// ENST0000(	NINL	chr20	0.0148567	1.50748
3914032	NM_012384 // GMEB2 /// ENST00000370077 // GMEB2 /// ENST00000266068 // GMEB2 //.	GMEB2	chr20	0.0459882	1.5036
3878414	BC033985 // MGC44328 /// XR_040563 // MGC44328	MGC44328	chr20	0.0414357	1.50122
3924676	NM_015151 // DIP2A /// NM_206889 // DIP2A /// NM_206890 // DIP2A /// NM_206891 .	DIP2A	chr21	0.00441107	2.21109
3933193	NR_027272 // C21orf129	C21orf129	chr21	0.0127245	2.12425

3926302	NM_002772 // TMPRSS15 /// ENST00000284885 // PRSS7 /// BC111749 // TMPRSS15	TMPRSS15	chr21	0.0188804	2.04411
3934484	NM_004928 // C21orf2 /// ENST00000339818 // C21orf2 /// ENST00000380160 // C21or	C21orf2	chr21	0.00221398	1.75963
3918387	NM_005806 // OLIG2 /// ENST00000382357 // OLIG2 /// ENST00000333337 // OLIG2 //.	OLIG2	chr21	0.0386613	1.70868
3917207	NM_020152 // C21orf7 /// ENST00000399947 // C21orf7 /// AY171599 // C21orf7	C21orf7	chr21	0.00456283	1.66934
3934426	NM_015259 // ICOSLG /// ENST00000400379 // ICOSLG /// BC064637 // ICOSLG /// AF2	ICOSLG	chr21	0.0268113	1.66018
3931529	NM_002240 // KCNJ6 /// ENST00000400482 // KCNJ6 /// D87327 // KCNJ6 /// ENST0000	KCNJ6	chr21	0.0112976	1.63358
3919842	NM_001236 // CBR3 /// ENST00000290354 // CBR3 /// AB041012 // CBR3	CBR3	chr21	0.00961796	1.62271
3935044	NM_194255 // SLC19A1 /// ENST00000311124 // SLC19A1 /// U19720 // SLC19A1 /// EN	SLC19A1	chr21	0.00175527	1.61846
3934452	NM_013369 // DNMT3L /// NM_175867 // DNMT3L /// ENST00000270172 // DNMT3L /// AF	DNMT3L	chr21	0.00315854	1.55931
3928533	NM_181609 // KRTAP19-3 /// AF139541 // KRTAP19-3 /// ENST00000433652 // KRTAP19-	KRTAP19-3	chr21	0.00944015	1.50703
3959676	---	0	chr22	0.0100821	2.23328
3959634	NM_003753 // EIF3D /// ENST00000216190 // EIF3D /// ENST00000405442 // EIF3D //.	EIF3D	chr22	0.0013269	1.98995
3952997	NM_001670 // ARVCF /// ENST00000263207 // ARVCF /// BC137436 // ARVCF /// U51269	ARVCF	chr22	0.00465151	1.9024
3945045	NM_003614 // GALR3 /// ENST00000249041 // GALR3 /// AF073799 // GALR3	GALR3	chr22	0.00995533	1.85609
3962849	NM_173050 // SCUBE1 /// ENST00000360835 // SCUBE1 /// AF525689 // SCUBE1 /// ENS	SCUBE1	chr22	0.00233739	1.80422
3952847	NM_053004 // GNB1L /// ENST00000329517 // GNB1L /// ENST00000403325 // GNB1L //.	GNB1L	chr22	0.0195007	1.79942
3947246	NM_145733 // SEPT3 /// NM_019106 // SEPT3 /// ENST00000396426 // SEPT3 /// ENST0	40789	chr22	0.0134477	1.74979
3960497	NM_152221 // CSNK1E /// NM_001894 // CSNK1E /// ENST00000359867 // CSNK1E /// EN	CSNK1E	chr22	0.00506694	1.71868
3947017	NM_001142964 // C22orf46 /// ENST00000402966 // CTA-216E10.6 /// ENST00000451377	C22orf46	chr22	0.00144409	1.71596
3954881	NM_013378 // VPREB3 /// ENST00000248948 // VPREB3 /// AY359000 // VPREB3 /// BC0	VPREB3	chr22	0.0347898	1.69361
3956776	NM_006477 // RASL10A /// NM_001007279 // RASL10A /// ENST00000216101 // RASL10A	RASL10A	chr22	0.0233499	1.68775
3950670	NM_031454 // SELO /// ENST00000380903 // RP3-402G11.5 /// AY324823 // SELO /// E	SELO	chr22	0.0466918	1.68749
3937163	NM_152906 // C22orf25 /// ENST00000327374 // C22orf25 /// ENST00000420290 // C22	C22orf25	chr22	0.0385348	1.67726
3944969	NM_138632 // TRIOBP /// ENST00000331103 // TRIOBP /// ENST00000407319 // TRIOBP	TRIOBP	chr22	0.012035	1.6692
3950671	NM_031454 // SELO /// ENST00000380903 // RP3-402G11.5 /// AY324823 // SELO /// E	SELO	chr22	0.0478312	1.66373
3964083	NM_014246 // CELSR1 /// ENST00000262738 // CELSR1 /// AF231024 // CELSR1 /// ENS	CELSR1	chr22	0.0462894	1.63828
3947260	NM_152613 // WBP2NL /// ENST00000329620 // WBP2NL /// ENST00000328823 // WBP2NL	WBP2NL	chr22	0.00259425	1.62022
3944580	NM_000395 // CSF2RB /// ENST00000403662 // CSF2RB /// BC070085 // CSF2RB /// ENS	CSF2RB	chr22	0.00662966	1.59974
3955838	NM_022081 // HPS4 /// NM_152841 // HPS4 /// ENST00000398145 // HPS4 /// ENST0000	HPS4	chr22	0.016853	1.57661
3966066	NR_027928 // CHKB-CPT1B /// NM_004377 // CPT1B /// NM_152245 // CPT1B /// NM_152	CHKB-CPT1B	chr22	0.0243059	1.57625
3944970	NM_138632 // TRIOBP /// ENST00000331103 // TRIOBP /// ENST00000407319 // TRIOBP	TRIOBP	chr22	0.0103104	1.57501
3944578	NM_000395 // CSF2RB /// ENST00000403662 // CSF2RB /// BC070085 // CSF2RB /// ENS	CSF2RB	chr22	0.0291225	1.56755
3965320	NM_014577 // BRD1 /// ENST00000404034 // BRD1 /// ENST00000216267 // BRD1 /// AK	BRD1	chr22	0.0301387	1.55933
3945189	NM_012407 // PICK1 /// NM_001039583 // PICK1 /// NM_001039584 // PICK1 /// ENST0	PICK1	chr22	0.0213399	1.55489
3963780	NM_148674 // SMC1B /// ENST00000357450 // SMC1B /// BC126208 // SMC1B /// ENST00	SMC1B	chr22	0.042705	1.55407
3959672	---	0	chr22	0.0330974	1.54494
3940951	NM_021115 // SEZ6L /// ENST00000248933 // SEZ6L /// AY358405 // SEZ6L /// AB0417	SEZ6L	chr22	0.0184683	1.54245
3946026	NM_021096 // CACNA1I /// NM_001003406 // CACNA1I /// ENST00000402142 // CACNA1I	CACNA1I	chr22	0.0175258	1.53169
3936263	NM_015367 // BCL2L13 /// ENST00000317582 // BCL2L13 /// ENST00000355028 // BCL2L	BCL2L13	chr22	0.00147649	1.52874
3962406	NM_145912 // NFAM1 /// ENST00000329021 // NFAM1 /// BC038241 // NFAM1	NFAM1	chr22	0.0219922	1.51962
3936978	NM_002688 // SEPT5 /// ENST00000455784 // SEPT5 /// CR456545 // SEPT5 /// BC0252	40791	chr22	0.0291132	1.51817
3954527	NM_080764 // ZNF280B /// ENST00000360412 // ZNF280B	ZNF280B	chr22	0.0099445	1.51776
3940848	NM_032608 // MYO18B /// AY077700 // MYO18B /// ENST00000418374 // MYO18B /// ENS	MYO18B	chr22	0.0359869	1.50685
3946274	NM_001162501 // TNRC6B /// NM_015088 // TNRC6B /// NM_001024843 // TNRC6B /// AK	TNRC6B	chr22	0.00243572	1.50322
2676624	NM_052859 // RFT1 /// ENST00000296292 // RFT1 /// BC043595 // RFT1 /// ENST0000	RFT1	chr3	0.0203617	3.27036
2673359	NM_000094 // COL7A1 /// ENST00000328333 // COL7A1 /// L02870 // COL7A1 /// ENST0	COL7A1	chr3	0.0020942	2.58823
2641670	NM_052985 // IFT122 /// NM_052989 // IFT122 /// NM_018262 // IFT122 /// NM_05299	IFT122	chr3	0.038902	2.45923
2637842	NM_006952 // UPK1B /// ENST00000264234 // UPK1B /// AB002155 // UPK1B	UPK1B	chr3	0.0267392	2.1123
2676209	NM_022908 // NT5DC2 /// ENST00000307076 // NT5DC2	NT5DC2	chr3	0.00155863	2.10164
2621367	NM_015466 // PTPN23 /// ENST00000265562 // PTPN23 /// AF290614 // PTPN23 /// ENS	PTPN23	chr3	0.045588	2.06921
2672553	NM_014159 // SETD2 /// ENST00000409792 // SETD2 /// AY576987 // SETD2 /// AY5769	SETD2	chr3	0.0278679	2.06264

2662346	NM_003656 // CAMK1 /// ENST00000256460 // CAMK1 /// L41816 // CAMK1 /// ENST0000	CAMK1	chr3	0.0318733	1.89838
2705695	NM_004122 // GHSR /// ENST00000427970 // GHSR	GHSR	chr3	0.000849877	1.89073
2663628	NM_024923 // NUP210 /// ENST00000254508 // NUP210 /// BC067089 // NUP210	NUP210	chr3	0.00395575	1.87894
2672176	NM_002343 // LTF /// ENST00000231751 // LTF /// AY493417 // LTF /// BC015822 //	LTF	chr3	0.0314913	1.78491
2612024	BC030599 // C3orf20 /// AK131481 // C3orf20 /// ENST00000253697 // C3orf20 /// E	C3orf20	chr3	0.0354654	1.77009
2623915	NM_007184 // NISCH /// ENST00000345716 // NISCH /// BC038102 // NISCH /// ENST00	NISCH	chr3	0.0462029	1.76023
2649910	NM_014575 // SCHIP1 /// ENST00000412423 // SCHIP1 /// DQ157848 // SCHIP1 /// ENS	SCHIP1	chr3	0.00407093	1.73501
2663315	NM_018306 // TMEM40 /// ENST00000314124 // TMEM40 /// ENST00000264728 // TMEM40	TMEM40	chr3	0.0334526	1.73131
2642836	NM_015268 // DNAJC13 /// ENST00000260818 // DNAJC13 /// AY779857 // DNAJC13	DNAJC13	chr3	0.0401443	1.71641
2673217	NM_002673 // PLXNB1 /// NM_001130082 // PLXNB1 /// ENST00000296440 // PLXNB1 //	PLXNB1	chr3	0.0273306	1.71047
2666481	NM_001068 // TOP2B /// ENST00000435706 // TOP2B /// ENST00000264331 // TOP2B //	TOP2B	chr3	0.0297496	1.71006
2672211	NM_024750 // LRRC2 /// ENST00000296144 // LRRC2 /// BC029118 // LRRC2	LRRC2	chr3	0.0459231	1.69728
2669714	NM_198056 // SCN5A /// NM_000335 // SCN5A /// NM_001099404 // SCN5A /// NM_0010	SCN5A	chr3	0.0101569	1.67235
2655520	NM_018358 // ABCF3 /// ENST00000429586 // ABCF3 /// ENST00000421340 // ABCF3 //	ABCF3	chr3	0.026431	1.66824
2696324	NM_016201 // AMOTL2 /// ENST00000249883 // AMOTL2 /// ENST00000422605 // AMOTL2	AMOTL2	chr3	0.00738658	1.66302
2609195	NM_000844 // GRM7 /// NM_181874 // GRM7 /// ENST00000357716 // GRM7 /// ENST0000	GRM7	chr3	0.0290889	1.6624
2677361	NM_003392 // WNT5A /// ENST00000264634 // WNT5A /// BC064694 // WNT5A /// ENST00	WNT5A	chr3	0.0165484	1.65362
2694154	NM_003707 // RUVBL1 /// ENST00000322623 // RUVBL1 /// Y18418 // RUVBL1 /// ENST0	RUVBL1	chr3	0.0463397	1.6445
2636081	NM_001171747 // C3orf52 /// NM_024616 // C3orf52 /// ENST00000264848 // C3orf52	C3orf52	chr3	0.000648554	1.64059
2712825	NM_138461 // TM4SF19 /// ENST00000442633 // TM4SF19 /// ENST00000273695 // TM4SF	TM4SF19	chr3	0.000901804	1.63302
2623408	NM_001003931 // PARP3 /// NM_005485 // PARP3 /// ENST00000417220 // PARP3 /// EN	PARP3	chr3	0.0474978	1.61908
2686763	NM_014415 // ZBTB11 /// ENST00000312938 // ZBTB11 /// U69274 // ZBTB11 /// NR_02	ZBTB11	chr3	0.0323694	1.61615
2669948	AB063301 // CSRNP1	CSRNP1	chr3	0.00757624	1.61569
2650259	NM_005496 // SMC4 /// NM_001002800 // SMC4 /// ENST00000357388 // SMC4 /// ENST0	SMC4	chr3	0.00239846	1.60186
2673289	NM_016479 // SHISA5 /// ENST00000296444 // SHISA5 /// AF520698 // SHISA5 /// AK3	SHISA5	chr3	0.0161979	1.5989
2694914	NM_015103 // PLXND1 /// ENST00000324093 // PLXND1 /// AY116661 // PLXND1 /// ENS	PLXND1	chr3	0.0396727	1.59837
2634104	NM_031419 // NFKBIZ /// ENST00000326172 // NFKBIZ /// BC060800 // NFKBIZ /// ENS	NFKBIZ	chr3	0.0247104	1.59345
2692388	NM_183357 // ADCY5 /// ENST00000309879 // ADCY5 /// ENST00000415791 // ADCY5	ADCY5	chr3	0.0197291	1.59184
2618736	NM_198484 // ZNF621 /// NM_001098414 // ZNF621 /// BC101619 // ZNF621 /// ENST00	ZNF621	chr3	0.0390901	1.59112
2655548	NM_018358 // ABCF3 /// ENST00000429586 // ABCF3 /// ENST00000292808 // ABCF3 //	ABCF3	chr3	0.0115485	1.58829
2655388	NM_003907 // EIF2B5 /// ENST00000273783 // EIF2B5	EIF2B5	chr3	0.0121175	1.58829
2676244	NM_018165 // PBRM1 /// NM_018313 // PBRM1 /// NM_181042 // PBRM1 /// ENST0000035	PBRM1	chr3	0.00428949	1.58677
2709801	NM_001706 // BCL6 /// NM_001130845 // BCL6 /// NM_001134738 // BCL6 /// ENST0000	BCL6	chr3	0.0376618	1.58506
2617507	BC129824 // DLEC1	DLEC1	chr3	0.00684139	1.57596
2641046	NM_013336 // SEC61A1 /// ENST00000243253 // SEC61A1 /// AF084458 // SEC61A1 ///	SEC61A1	chr3	0.00656521	1.57586
2622941	NM_004635 // MAPKAPK3 /// ENST00000446044 // MAPKAPK3 /// ENST00000357955 // MAF	MAPKAPK3	chr3	0.0122862	1.57505
2649233	NM_001004316 // LEKR1 /// ENST00000356539 // LEKR1 /// AK131473 // LEKR1	LEKR1	chr3	0.0121233	1.57353
2712966	NM_182627 // WDR53 /// ENST00000332629 // WDR53 /// EF011620 // WDR53 /// ENST00	WDR53	chr3	0.00138696	1.56799
2625628	NM_012096 // APPL1 /// ENST00000288266 // APPL1 /// AF169797 // APPL1	APPL1	chr3	0.0276454	1.55967
2698721	NM_001039547 // GK5 /// NR_033289 // GK5 /// BC032470 // GK5	GK5	chr3	0.0399661	1.55938
2618676	NM_001145082 // ZNF619 /// NM_001145093 // ZNF619 /// NM_001145083 // ZNF619 //	ZNF619	chr3	0.0480912	1.55451
2617469	NM_015873 // VILL /// ENST00000283713 // VILL /// ENST00000383759 // VILL /// BC	VILL	chr3	0.00912881	1.55135
2635316	NM_014648 // DZIP3 /// ENST00000361582 // DZIP3 /// BC063882 // DZIP3 /// ENST00	DZIP3	chr3	0.00559681	1.5506
2634564	NM_001627 // ALCAM /// ENST00000306107 // ALCAM /// DQ486139 // ALCAM /// ENST00	ALCAM	chr3	0.00958959	1.54862
2649935	ENST00000445224 // SCHIP1	SCHIP1	chr3	0.035351	1.54769
2711243	NM_032279 // ATP13A4 /// ENST00000342695 // ATP13A4 /// ENST00000295548 // ATP13	ATP13A4	chr3	0.029001	1.54701
2710546	NM_018192 // LEPREL1 /// ENST00000319332 // LEPREL1	LEPREL1	chr3	0.0299081	1.54456
2712114	NM_012287 // ACAP2 /// ENST00000326793 // ACAP2 /// BC060767 // ACAP2	ACAP2	chr3	0.0242929	1.5409
2642381	NM_014382 // ATP2C1 /// NM_001001487 // ATP2C1 /// NM_001001485 // ATP2C1 /// NM	ATP2C1	chr3	0.00890845	1.53848
2619317	NM_004624 // VIPR1 /// ENST00000325123 // VIPR1 /// EF577396 // VIPR1 /// BC0644	VIPR1	chr3	0.00558056	1.53645
2695320	NM_014065 // ASTE1 /// ENST00000264992 // ASTE1 /// AK294156 // ASTE1 /// ENST00	ASTE1	chr3	0.0402672	1.53524

2611020	NM_133625 // SYN2 /// NM_003178 // SYN2 /// ENST00000341648 // SYN2 /// ENST0000	SYN2	chr3	0.0302208	1.53411
2610299	NM_033084 // FANCD2 /// NM_001018115 // FANCD2 /// ENST00000287647 // FANCD2 //.	FANCD2	chr3	0.00725611	1.53013
2656983	NM_153708 // RTP1 /// ENST00000312295 // RTP1 /// BC065202 // RTP1	RTP1	chr3	0.039058	1.52982
2691604	NM_199420 // POLQ /// ENST00000264233 // POLQ /// AY032677 // POLQ /// ENST00000	POLQ	chr3	0.0146116	1.52914
2622602	NM_144499 // GNAT1 /// NM_000172 // GNAT1 /// ENST00000232461 // GNAT1 /// ENST0	GNAT1	chr3	0.00628938	1.5271
2621726	---	0	chr3	0.0345855	1.52448
2713080	NM_007362 // NCBP2 /// NM_001042540 // NCBP2 /// ENST00000321256 // NCBP2 /// EN	NCBP2	chr3	0.0278254	1.52302
2622579	NM_004186 // SEMA3F /// ENST00000002829 // SEMA3F /// BC042914 // SEMA3F /// U33	SEMA3F	chr3	0.0232931	1.51787
2679381	NM_018008 // FEZF2 /// ENST00000283268 // FEZF2 /// AK290464 // FEZF2	FEZF2	chr3	0.0323109	1.51762
2621663	NM_207102 // FBXW12 /// NM_001159927 // FBXW12 /// NM_001159929 // FBXW12 /// EN	FBXW12	chr3	0.036795	1.51175
2673475	NM_000094 // COL7A1 /// ENST00000328333 // COL7A1 /// L02870 // COL7A1 /// ENST0	COL7A1	chr3	0.0293363	1.51099
2686464	NM_015429 // ABI3BP /// ENST00000284322 // ABI3BP /// AK299938 // ABI3BP /// ENS	ABI3BP	chr3	0.0219861	1.5074
2675111	NM_006764 // IFRD2 /// ENST00000436390 // IFRD2 /// ENST00000417626 // IFRD2 //.	IFRD2	chr3	0.0243687	1.50464
2787089	NM_021833 // UCP1 /// ENST00000262999 // UCP1 /// BC098352 // UCP1	UCP1	chr4	0.000506563	2.10941
2790094	NM_152680 // TMEM154 /// ENST00000304385 // TMEM154 /// BC060775 // TMEM154 ///	TMEM154	chr4	0.00399177	2.044
2775568	NM_031372 // HNRPDL /// NR_003249 // HNRPDL /// ENST00000295470 // HNRPDL	HNRPDL	chr4	0.0276748	1.93741
2768379	NM_003328 // TXK /// ENST00000264316 // TXK /// L27071 // TXK	TXK	chr4	0.012829	1.88144
2728988	NM_015236 // LPHN3 /// ENST00000280009 // LPHN3 /// ENST00000295349 // LPHN3	LPHN3	chr4	0.0148601	1.81509
2781553	NM_198721 // COL25A1 /// NM_032518 // COL25A1 /// ENST00000399132 // COL25A1 //.	COL25A1	chr4	0.0207856	1.80687
2759726	NM_001130083 // ABLIM2 /// NM_001130084 // ABLIM2 /// NM_001130085 // ABLIM2 //	ABLIM2	chr4	0.0108039	1.80389
2745024	NM_001153484 // SCOC /// NM_001153663 // SCOC /// NM_032547 // SCOC /// NM_0011	SCOC	chr4	0.00469573	1.79071
2794816	NM_005429 // VEGFC /// ENST00000280193 // VEGFC /// BC063685 // VEGFC	VEGFC	chr4	0.0359586	1.76825
2714444	NM_000203 // IDUA /// ENST00000247933 // IDUA /// ENST00000453894 // IDUA /// AK	IDUA	chr4	0.00159643	1.74924
2749814	NM_014247 // RAPGEF2 /// ENST00000264431 // RAPGEF2 /// BC117321 // RAPGEF2	RAPGEF2	chr4	0.00351912	1.7067
2725133	NM_014988 // LIMCH1 /// NM_001112717 // LIMCH1 /// NM_001112718 // LIMCH1 /// NM	LIMCH1	chr4	0.000491309	1.68458
2768078	NM_000809 // GABRA4 /// ENST00000264318 // GABRA4 /// AK090780 // GABRA4	GABRA4	chr4	0.0421413	1.6815
2744752	NM_002413 // MGST2 /// ENST00000265498 // MGST2 /// BC025416 // MGST2	MGST2	chr4	0.0118119	1.68029
2775241	NM_006259 // PRKG2 /// ENST00000395578 // PRKG2 /// ENST00000264399 // PRKG2 //.	PRKG2	chr4	0.0103611	1.67395
2779912	NM_005908 // MANBA /// ENST00000226578 // MANBA /// AB209846 // MANBA	MANBA	chr4	0.0130395	1.64682
2758066	NM_001120 // MFSD10 /// NM_001146069 // MFSD10 /// ENST00000355443 // MFSD10 //.	MFSD10	chr4	0.0133881	1.64038
2756818	NM_001347 // DGKQ /// ENST00000273814 // DGKQ /// ENST00000405681 // DGKQ /// BC	DGKQ	chr4	0.0427797	1.60531
2787088	NM_021833 // UCP1 /// ENST00000262999 // UCP1 /// BC098352 // UCP1	UCP1	chr4	0.00909402	1.60059
2766291	NM_024943 // TMEM156 /// ENST00000344606 // TMEM156 /// ENST00000381938 // TMEM1	TMEM156	chr4	0.000146139	1.59995
2779494	NM_002106 // H2AFZ /// ENST00000296417 // H2AFZ /// AK056803 // H2AFZ	H2AFZ	chr4	0.0029898	1.57595
2784197	NM_001130698 // TRPC3 /// NM_003305 // TRPC3 /// ENST00000264811 // TRPC3 /// EN	TRPC3	chr4	0.00848628	1.56947
2779425	NM_021970 // MAPKSP1 /// ENST00000226522 // MAPKSP1 /// AF201947 // MAPKSP1	MAPKSP1	chr4	0.0228329	1.55121
2758872	NM_018659 // CYTL1 /// ENST00000307746 // CYTL1 /// AF193766 // CYTL1	CYTL1	chr4	0.00609271	1.54257
2770337	NM_032495 // HOPX /// NM_139212 // HOPX /// NM_001145460 // HOPX /// ENST0000038	HOPX	chr4	0.00386865	1.53803
2724379	NR_026854 // LOC401127	LOC401127	chr4	0.0133673	1.53595
2759862	NM_003501 // ACOX3 /// NM_001101667 // ACOX3 /// ENST00000356406 // ACOX3 /// EN	ACOX3	chr4	0.00411128	1.53023
2739302	NM_198506 // LRIT3 /// ENST00000379920 // LRIT3 /// ENST00000327908 // LRIT3 //.	LRIT3	chr4	0.0451509	1.52982
2756737	NM_005255 // GAK /// ENST00000314167 // GAK /// D88435 // GAK /// ENST000003829	GAK	chr4	0.0138459	1.52773
2771992	NM_182502 // TMPRSS11B /// ENST00000332644 // TMPRSS11B /// BX537945 // TMPRSS11	TMPRSS11B	chr4	0.0289491	1.51696
2875642	NM_017665 // ZCCHC10 /// ENST00000324170 // ZCCHC10 /// AK122970 // ZCCHC10 ///	ZCCHC10	chr5	0.00883836	2.68571
2853113	NM_000949 // PRLR /// ENST00000382002 // PRLR /// BC059392 // PRLR /// ENST0000C	PRLR	chr5	0.0400976	2.5952
2879188	NM_000800 // FGF1 /// NM_033136 // FGF1 /// NM_033137 // FGF1 /// NM_001144892 .	FGF1	chr5	0.00907665	2.48238
2888300	NM_016391 // NOP16 /// ENST00000389158 // NOP16 /// ENST00000341213 // NOP16 //.	NOP16	chr5	0.0359809	2.37281
2820889	NM_199243 // GPR150 /// ENST00000380007 // GPR150 /// BC030197 // GPR150	GPR150	chr5	0.00623123	2.18525
2800800	NM_020546 // ADCY2 /// ENST00000338316 // ADCY2 /// BC111743 // ADCY2 /// ENST00	ADCY2	chr5	0.00675814	2.08882
2884601	NM_024565 // CCNJL /// ENST00000393977 // CCNJL /// AK294000 // CCNJL /// AK3028	CCNJL	chr5	0.0102919	2.06112
2881330	NM_015981 // CAMK2A /// NM_171825 // CAMK2A /// ENST00000398376 // CAMK2A /// EN	CAMK2A	chr5	0.0201708	2.00608

2886188	NM_003062 // SLIT3 /// ENST00000332966 // SLIT3 /// AY358884 // SLIT3 /// ENST0(	SLIT3	chr5	0.0420695	2.00581
2841821	NM_015980 // HMP19 /// ENST00000303177 // HMP19 /// BC002619 // HMP19 /// ENST0(	HMP19	chr5	0.00151487	1.9626
2875940	NM_020199 // C5orf15 /// ENST00000231512 // C5orf15 /// AF226055 // C5orf15	C5orf15	chr5	0.013789	1.91736
2876259	NM_001033503 // SAR1B /// NM_016103 // SAR1B /// ENST00000402673 // SAR1B /// EN	SAR1B	chr5	0.0266358	1.79194
2832655	NM_018914 // PCDHGA11 /// NM_032091 // PCDHGA11 /// NM_032092 // PCDHGA11 /// AF	PCDHGA11	chr5	0.0318415	1.77239
2842936	NM_002011 // FGFR4 /// NM_022963 // FGFR4 /// NM_213647 // FGFR4 /// ENST0000039	FGFR4	chr5	0.0192931	1.75407
2845268	AK126844 // FLJ44896	FLJ44896	chr5	0.0187017	1.74085
2808467	NM_012343 // NNT /// NM_182977 // NNT /// ENST00000264663 // NNT /// ENST0000034	NNT	chr5	0.0153353	1.73877
2827777	NM_133638 // ADAMTS19 /// ENST00000274487 // ADAMTS19	ADAMTS19	chr5	0.021062	1.7216
2847983	NM_003966 // SEMA5A /// ENST00000382496 // SEMA5A /// U52840 // SEMA5A	SEMA5A	chr5	0.0185138	1.69989
2818023	NM_006909 // RASGRF2 /// ENST00000265080 // RASGRF2 /// BC126112 // RASGRF2	RASGRF2	chr5	0.0358013	1.67061
2845458	NM_007030 // TPPP /// ENST00000360578 // TPPP /// BC131506 // TPPP	TPPP	chr5	0.0206964	1.64933
2837502	NM_173491 // LSM11 /// ENST00000286307 // LSM11 /// BC051353 // LSM11	LSM11	chr5	0.00534278	1.6406
2799216	NM_004553 // NDUFS6 /// ENST00000274137 // NDUFS6 /// AF044959 // NDUFS6	NDUFS6	chr5	0.017	1.62612
2877650	NM_001037633 // SIL1 /// NM_022464 // SIL1 /// ENST00000265195 // SIL1 /// ENST0	SIL1	chr5	0.0316521	1.6204
2834576	NM_001001325 // SPINK14 /// ENST00000356972 // SPINK5L2	SPINK14	chr5	0.0010853	1.61712
2845886	NM_030782 // CLPTM1L /// ENST00000320895 // CLPTM1L /// AB045223 // CLPTM1L ///	CLPTM1L	chr5	0.0199843	1.61338
2874986	NM_015256 // ACSL6 /// NM_001009185 // ACSL6 /// ENST00000379264 // ACSL6 /// EN	ACSL6	chr5	0.00158218	1.60918
2881824	NM_001155 // ANXA6 /// NM_004033 // ANXA6 /// ENST00000354546 // ANXA6 /// ENST(	ANXA6	chr5	0.0143265	1.6084
2886683	BC025707 // KCNMB1	KCNMB1	chr5	0.00460664	1.59879
2826192	NM_005460 // SNCAIP /// ENST00000261368 // SNCAIP /// BC040552 // SNCAIP /// ENS	SNCAIP	chr5	0.034846	1.59747
2850728	NM_004934 // CDH18 /// NM_001167667 // CDH18 /// ENST00000382275 // CDH18 /// BC	CDH18	chr5	0.0325374	1.59702
2886190	NM_003062 // SLIT3 /// ENST00000332966 // SLIT3 /// AY358884 // SLIT3 /// ENST0(	SLIT3	chr5	0.0128386	1.5941
2890550	ENST00000393371 // RASGEF1C	RASGEF1C	chr5	0.0329472	1.59387
2827727	NM_016048 // ISOC1 /// ENST00000173527 // ISOC1 /// BC014105 // ISOC1	ISOC1	chr5	0.0432275	1.59153
2890672	NM_005110 // GFPT2 /// ENST00000253778 // GFPT2 /// AB016789 // GFPT2	GFPT2	chr5	0.0342639	1.58757
2813386	NM_022902 // SLC30A5 /// ENST00000396591 // SLC30A5 /// BX537394 // SLC30A5 ///	SLC30A5	chr5	0.0485712	1.58612
2871076	NM_022140 // EPB41L4A /// ENST00000261486 // EPB41L4A /// ENST00000359109 // EPE	EPB41L4A	chr5	0.0216549	1.58578
2842872	NM_012279 // ZNF346 /// ENST00000358149 // ZNF346 /// BC007775 // ZNF346 /// ENS	ZNF346	chr5	0.0109217	1.56737
2845964	NM_001044 // SLC6A3 /// ENST00000270349 // SLC6A3 /// M95167 // SLC6A3	SLC6A3	chr5	0.0106078	1.56041
2873827	NM_001182 // ALDH7A1 /// BC002515 // ALDH7A1 /// BC071712 // ALDH7A1 /// BC07317	ALDH7A1	chr5	0.0154589	1.55599
2875580	NM_014423 // AFF4 /// ENST00000265343 // AFF4 /// BC137226 // AFF4 /// ENST0000(	AFF4	chr5	0.0111489	1.55307
2814554	NM_018429 // BDP1 /// ENST00000358731 // BDP1 /// BC146792 // BDP1 /// ENST0000(	BDP1	chr5	0.0179878	1.5504
2870230	NM_001163315 // FBXL17	FBXL17	chr5	0.0445323	1.54394
2806272	NM_194283 // DNAJC21 /// NM_001012339 // DNAJC21 /// ENST00000342382 // DNAJC21	DNAJC21	chr5	0.023968	1.54368
2881305	NM_015981 // CAMK2A /// NM_171825 // CAMK2A /// ENST00000398376 // CAMK2A /// EN	CAMK2A	chr5	0.0375915	1.53857
2891103	NM_032765 // TRIM52 /// ENST00000327767 // TRIM52 /// AB209243 // TRIM52	TRIM52	chr5	0.0274076	1.53292
2828655	NM_005732 // RAD50 /// ENST00000265335 // RAD50 /// ENST00000378823 // RAD50 //.	RAD50	chr5	0.00751646	1.51955
2881565	NM_001135643 // DCTN4 /// NM_016221 // DCTN4 /// NM_001135644 // DCTN4 /// ENST(	DCTN4	chr5	0.0322414	1.51752
2831676	NM_031467 // SLC4A9 /// ENST00000437301 // SLC4A9 /// ENST00000230993 // SLC4A9	SLC4A9	chr5	0.0104741	1.51669
2798824	NM_018140 // CEP72 /// ENST00000264935 // CEP72 /// BC000132 // CEP72 /// ENST0(	CEP72	chr5	0.0307227	1.51479
2844279	---	0	chr5	0.0281985	1.50354
2966420	NM_005190 // CCNC /// ENST00000369220 // CCNC /// BC041123 // CCNC /// ENST0000(	CCNC	chr6	0.0222009	2.33242
2955867	NM_015234 // GPR116 /// NM_001098518 // GPR116 /// ENST00000452370 // GPR116 //.	GPR116	chr6	0.023068	2.05788
2937626	NR_026780 // C6orf208	C6orf208	chr6	0.00534507	2.03932
2942356	NM_016495 // TBC1D7 /// NM_001143964 // TBC1D7 /// ENST00000421203 // TBC1D7 //.	TBC1D7	chr6	0.0330108	1.95117
2910725	NM_018214 // LRRC1 /// ENST00000370888 // LRRC1 /// AK021896 // LRRC1 /// ENST0(	LRRC1	chr6	0.0292052	1.92541
2921323	NM_001634 // AMD1 /// NM_001033059 // AMD1 /// ENST00000412644 // AMD1 /// ENST(	AMD1	chr6	0.0110054	1.8733
2898854	NM_017640 // LRRC16A /// NM_001173977 // LRRC16A /// ENST00000399313 // LRRC16A	LRRC16A	chr6	0.0166287	1.87246
2966602	NM_005068 // SIM1 /// ENST00000369208 // SIM1 /// ENST00000262901 // SIM1 /// U7	SIM1	chr6	0.0127145	1.85752
2904590	NM_022047 // DEF6 /// ENST00000316637 // DEF6 /// AY241694 // DEF6	DEF6	chr6	0.029793	1.83249

2951103	NM_006703 // NUDT3 /// ENST00000358797 // NUDT3 /// AF062529 // NUDT3	NUDT3	chr6	0.0222205	1.81983
2981920	NM_003379 // EZR /// NM_001111077 // EZR /// ENST00000367075 // EZR /// ENST0000	EZR	chr6	0.0154416	1.81071
2915157	NM_006670 // TPBG /// NM_001166392 // TPBG /// ENST00000369750 // TPBG /// AK291	TPBG	chr6	0.0087967	1.79078
2948570	NM_014641 // MDC1 /// ENST00000376406 // MDC1 /// ENST00000420019 // MDC1 /// EN	MDC1	chr6	0.00985812	1.78904
2900145	NM_012367 // OR2B6 /// ENST00000244623 // OR2B6 /// BC110457 // OR2B6	OR2B6	chr6	0.0282467	1.78215
2901844	NM_024909 // C6orf134 /// ENST00000329992 // C6orf134 /// ENST00000383583 // C6c	C6orf134	chr6	0.0310147	1.77956
2906114	NM_002062 // GLP1R /// ENST00000373256 // GLP1R /// U01157 // GLP1R	GLP1R	chr6	0.0175192	1.77439
2954050	NM_033502 // TRERF1 /// ENST00000372922 // TRERF1 /// AM404182 // TRERF1 /// ENS	TRERF1	chr6	0.0296082	1.76472
2980051	NM_182961 // SYNE1 /// ENST00000265368 // SYNE1 /// ENST00000367247 // SYNE1 //	SYNE1	chr6	0.0212683	1.7308
2936743	NM_018974 // UNC93A /// NM_001143947 // UNC93A /// ENST00000230256 // UNC93A //	UNC93A	chr6	0.0043018	1.7288
2949545	NM_006709 // EHMT2 /// NM_025256 // EHMT2 /// ENST00000375537 // EHMT2 /// ENST0	EHMT2	chr6	0.000374659	1.71193
2952017	AK124222 // LOC100131043	LOC100131043	chr6	0.0118474	1.67895
2899410	ENST00000297020 // BTN2A3 /// ENST00000377662 // BTN2A3 /// BC143636 // BTN2A3	BTN2A3	chr6	0.00927779	1.67118
2949927	NM_004557 // NOTCH4 /// ENST00000375023 // NOTCH4 /// ENST00000457094 // NOTCH4	NOTCH4	chr6	0.0496423	1.66674
2952842	NM_003740 // KCNK5 /// ENST00000359534 // KCNK5 /// BC060793 // KCNK5	KCNK5	chr6	0.000981937	1.66307
2912991	NM_024576 // OGFRL1 /// ENST00000370435 // OGFRL1 /// BC110999 // OGFRL1	OGFRL1	chr6	0.0391153	1.64618
2950488	NM_021976 // RXRB /// ENST00000374680 // RXRB /// ENST00000383217 // RXRB /// EN	RXRB	chr6	0.0418374	1.63874
2975481	NM_001134831 // AHI1 /// NM_017651 // AHI1 /// NM_001134830 // AHI1 /// NM_00113	AHI1	chr6	0.0270895	1.63331
2974201	NM_004830 // MED23 /// NM_015979 // MED23 /// ENST00000368068 // MED23 /// ENST0	MED23	chr6	3.90052E-05	1.61785
2933386	NM_016224 // SNX9 /// ENST00000392185 // SNX9 /// AF172847 // SNX9	SNX9	chr6	0.000388251	1.61187
2901514	NM_021253 // TRIM39 /// NM_172016 // TRIM39 /// BC034985 // TRIM39	TRIM39	chr6	0.0408378	1.60328
2934392	NM_000876 // IGF2R /// ENST00000356956 // IGF2R /// J03528 // IGF2R /// ENST0000	IGF2R	chr6	0.0350435	1.60112
2977131	NM_002511 // NMBR /// ENST00000258042 // NMBR /// M73482 // NMBR	NMBR	chr6	0.0343837	1.59749
2928415	NM_016485 // VTA1 /// ENST00000367630 // VTA1 /// AF271994 // VTA1 /// ENST00000	VTA1	chr6	0.00403223	1.59155
2927529	NM_006290 // TNFAIP3 /// ENST00000237289 // TNFAIP3 /// M59465 // TNFAIP3	TNFAIP3	chr6	0.0361614	1.59031
2909554	NM_181744 // OPN5 /// NM_001030051 // OPN5 /// ENST00000371211 // OPN5 /// ENST0	OPN5	chr6	0.0222562	1.58889
2941762	NM_207582 // ERVFRDE1 /// BC068585 // ERVFRDE1	ERVFRDE1	chr6	0.0152611	1.58767
2902681	AJ245661 // C6orf26	C6orf26	chr6	0.024628	1.57672
2937181	NM_022138 // SMOC2 /// NM_001166412 // SMOC2 /// ENST00000354536 // SMOC2 /// AE	SMOC2	chr6	0.0133786	1.57358
2949362	NM_025258 // C6orf27 /// ENST00000375688 // C6orf27 /// ENST00000418870 // C6ort	C6orf27	chr6	0.0261821	1.56676
2902859	NM_001710 // CFB /// S67310 // CFB /// BC004143 // CFB /// BC007990 // CFB /// A	CFB	chr6	0.00340875	1.56309
2948514	NM_003587 // DHX16 /// NM_001164239 // DHX16 /// ENST00000376442 // DHX16 /// EN	DHX16	chr6	0.0212114	1.56169
2951072	NM_178508 // C6orf1 /// ENST00000335352 // C6orf1 /// BC047919 // C6orf1	C6orf1	chr6	0.0234086	1.55842
2956094	NM_014452 // TNFRSF21 /// ENST00000296861 // TNFRSF21 /// AY358304 // TNFRSF21	TNFRSF21	chr6	0.0294918	1.55214
2949372	NM_025258 // C6orf27 /// ENST00000375688 // C6orf27 /// ENST00000418870 // C6ort	C6orf27	chr6	0.0181648	1.55098
2977499	NM_182503 // ADAT2 /// ENST00000237283 // ADAT2 /// ENST00000342031 // ADAT2 //	ADAT2	chr6	0.0356989	1.54452
2908123	NM_006502 // POLH /// ENST00000372236 // POLH /// AB024313 // POLH /// ENST00000	POLH	chr6	0.0161686	1.53766
2903423	NM_002121 // HLA-DPB1 /// ENST00000418931 // HLA-DPB1 /// ENST00000433800 // HLA	HLA-DPB1	chr6	0.0177353	1.52372
2934407	NM_000876 // IGF2R /// ENST00000356956 // IGF2R /// J03528 // IGF2R /// ENST0000	IGF2R	chr6	0.0259556	1.52362
2978105	NM_001042683 // SHPRH /// NM_173082 // SHPRH /// ENST00000367503 // SHPRH /// EN	SHPRH	chr6	0.0419707	1.52347
2979137	NM_032832 // LRP11 /// ENST00000239367 // LRP11 /// BC043141 // LRP11 /// ENST00	LRP11	chr6	0.0462407	1.52345
2978121	NM_001042683 // SHPRH /// NM_173082 // SHPRH /// ENST00000367503 // SHPRH /// EN	SHPRH	chr6	0.0138736	1.51934
2949359	NM_025258 // C6orf27 /// ENST00000375688 // C6orf27 /// ENST00000418870 // C6ort	C6orf27	chr6	0.0474236	1.51891
2956076	NM_014452 // TNFRSF21 /// ENST00000296861 // TNFRSF21 /// AY358304 // TNFRSF21 /	TNFRSF21	chr6	0.00229963	1.51783
2949911	NM_004557 // NOTCH4 /// ENST00000375023 // NOTCH4 /// ENST00000457094 // NOTCH4	NOTCH4	chr6	0.0166659	1.5147
2947903	NM_001470 // GABBR1 /// NM_021903 // GABBR1 /// NM_021904 // GABBR1 /// ENST0000	GABBR1	chr6	0.0234329	1.51155
2899546	NM_013375 // ABT1 /// ENST00000274849 // ABT1 /// BC066313 // ABT1	ABT1	chr6	0.0413426	1.50942
3030972	AK124542 // SSPO	SSPO	chr7	0.0303311	3.0903
3066971	NM_175884 // FLJ36031	FLJ36031	chr7	0.00567533	2.8759
3023253	NM_032643 // IRF5 /// NM_001098627 // IRF5 /// NM_001098629 // IRF5 /// NM_00109	IRF5	chr7	0.00134525	2.22332
3070937	NM_015450 // POT1 /// NR_003102 // POT1 /// NR_003103 // POT1 /// NM_001042594 /	POT1	chr7	0.00385138	2.03955

3048605	NM_001220 // CAMK2B /// NM_172078 // CAMK2B /// NM_172079 // CAMK2B /// NM_1720[illegible]	CAMK2B	chr7	0.039429	1.92361
3070546	NM_022444 // SLC13A1 /// ENST00000194130 // SLC13A1 /// BC111775 // SLC13A1 ///	SLC13A1	chr7	0.0190717	1.89826
3032747	NM_001936 // DPP6 /// ENST00000332007 // DPP6 /// ENST00000427557 // DPP[illegible]	DPP6	chr7	0.00854049	1.88159
3062941	NM_018842 // BAIAP2L1 /// ENST00000005260 // BAIAP2L1 /// AF119666 // BAIAP2L1	BAIAP2L1	chr7	0.0127179	1.87794
3035296	NM_001080453 // INTS1 /// ENST00000404767 // INTS1 /// AY358482 // INTS1 /// ENS	INTS1	chr7	0.0329135	1.82146
3007972	AY372054 // WBSCR28 /// ENST00000426490 // WBSCR28	WBSCR28	chr7	0.0403785	1.8162
3031774	NM_007188 // ABCB8 /// ENST00000358849 // ABCB8 /// AF047690 // ABCB8 /// ENST0[illegible]	ABCB8	chr7	0.0137516	1.80171
3071943	NM_001135914 // KCP /// NM_199349 // KCP /// ENST00000441244 // KCP /// ENST000[illegible]	KCP	chr7	0.00321665	1.79989
3076384	NM_004333 // BRAF /// ENST00000288602 // BRAF /// M95712 // BRAF	BRAF	chr7	0.0247846	1.78599
3056256	NM_004603 // STX1A /// NM_001165903 // STX1A /// ENST00000222812 // STX1A /// BC	STX1A	chr7	0.0218912	1.75168
3078704	NR_027788 // ZNF767 /// NR_027789 // ZNF767 /// BC047675 // ZNF767 /// ENST0000[illegible]	ZNF767	chr7	0.00702628	1.75099
3016228	NM_006349 // ZNHIT1 /// ENST00000305105 // ZNHIT1	ZNHIT1	chr7	0.0191975	1.74487
3028867	NM_004445 // EPHB6 /// ENST00000392957 // EPHB6 /// ENST00000442129 // EPHB6 //[illegible]	EPHB6	chr7	0.0247406	1.72936
3000074	NM_021130 // PPIA /// BC137057 // PPIA /// BC137058 // PPIA	PPIA	chr7	0.031365	1.70245
3035617	NM_003550 // MAD1L1 /// NM_001013836 // MAD1L1 /// NM_001013837 // MAD1L1 /// E[illegible]	MAD1L1	chr7	0.0127253	1.70242
2992866	AK127742 // C7orf30 /// ENST00000287543 // C7orf30	C7orf30	chr7	0.0182321	1.67959
3042740	NM_005522 // HOXA1 /// NM_153620 // HOXA1 /// ENST00000355633 // HOXA1 /// ENST[illegible]	HOXA1	chr7	0.0354484	1.67333
3002187	NM_182595 // POM121L12 /// ENST00000408890 // POM121L12	POM121L12	chr7	0.00679258	1.65797
3057651	NM_012479 // YWHAG /// ENST00000307630 // YWHAG /// BC020963 // YWHAG	YWHAG	chr7	0.00509263	1.65396
3055468	NM_031468 // CALN1 /// NM_001017440 // CALN1 /// ENST00000395275 // CALN1 /// E[illegible]	CALN1	chr7	0.0224891	1.65283
3017800	NM_021930 // RINT1 /// ENST00000257700 // RINT1 /// BC068483 // RINT1	RINT1	chr7	0.0327013	1.6504
3063704	NM_005916 // MCM7 /// NM_182776 // MCM7 /// ENST00000303887 // MCM7 /// ENST000[illegible]	MCM7	chr7	0.034031	1.65028
3027540	NM_004546 // NDUFB2 /// ENST00000247866 // NDUFB2 /// BC063026 // NDUFB2 /// ENS	NDUFB2	chr7	0.0355897	1.64234
3035263	NM_182924 // MICALL2 /// ENST00000297508 // MICALL2 /// AK074068 // MICALL2 ///	MICALL2	chr7	0.0134161	1.63141
3031909	NM_031946 // AGAP3 /// NM_001042535 // AGAP3 /// ENST00000397238 // AGAP3 /// BC	AGAP3	chr7	0.0157914	1.62752
3061834	NM_001099401 // SGCE /// NM_003919 // SGCE /// NM_001099400 // SGCE /// ENST000[illegible]	SGCE	chr7	0.0194158	1.61967
3020349	NM_001127500 // MET /// NM_000245 // MET /// ENST00000397752 // MET /// ENST000[illegible]	MET	chr7	0.0367114	1.61943
2992223	NM_003112 // SP4 /// ENST00000222584 // SP4 /// AK289728 // SP4 /// ENST0000044[illegible]	SP4	chr7	0.0111188	1.61533
3064569	NM_001084 // PLOD3 /// ENST00000223127 // PLOD3 /// BC011674 // PLOD3 /// ENST0[illegible]	PLOD3	chr7	0.00750903	1.61252
3048526	NM_001220 // CAMK2B /// NM_172078 // CAMK2B /// NM_172079 // CAMK2B /// NM_1720[illegible]	CAMK2B	chr7	0.0158121	1.60826
3057390	NM_005338 // HIP1 /// ENST00000336926 // HIP1 /// ENST00000404944 // HIP1 /// U7	HIP1	chr7	0.019594	1.59938
3013589	NM_004411 // DYNC1I1 /// NM_001135556 // DYNC1I1 /// NM_001135557 // DYNC1I1 //	DYNC1I1	chr7	0.0260589	1.59799
2988888	NM_006303 // AIMP2 /// ENST00000223029 // AIMP2 /// BC013630 // AIMP2 /// ENST0[illegible]	AIMP2	chr7	0.0238257	1.58641
3018376	NM_002736 // PRKAR2B /// ENST00000265717 // PRKAR2B /// BC075800 // PRKAR2B ///	PRKAR2B	chr7	0.0117537	1.58201
3080867	NM_007349 // PAXIP1 /// ENST00000404141 // PAXIP1 /// ENST00000397192 // PAXIP1	PAXIP1	chr7	0.0349621	1.57279
2987044	NM_001039966 // GPER /// NM_001098201 // GPER /// ENST00000397092 // GPER /// E[illegible]	GPER	chr7	0.00919677	1.57243
3023354	NM_005631 // SMO /// ENST00000249373 // SMO /// BC009989 // SMO	SMO	chr7	0.023577	1.57211
3016479	NM_181552 // CUX1 /// ENST00000292535 // CUX1 /// BC066592 // CUX1 /// ENST0000[illegible]	CUX1	chr7	0.027908	1.57071
3035246	NM_182924 // MICALL2 /// ENST00000297508 // MICALL2 /// AK074068 // MICALL2 ///	MICALL2	chr7	0.021568	1.5681
3007051	NM_022479 // WBSCR17 /// ENST00000333538 // WBSCR17 /// AF410457 // WBSCR17	WBSCR17	chr7	0.00506404	1.56721
3063266	NM_014891 // PDAP1 /// ENST00000350498 // PDAP1	PDAP1	chr7	0.0473688	1.56451
3015722	NM_023948 // MOSPD3 /// NM_001040097 // MOSPD3 /// NM_001040098 // MOSPD3 /// NM	MOSPD3	chr7	0.0141743	1.56261
3075145	NM_194071 // CREB3L2 /// ENST00000330387 // CREB3L2 /// AK293048 // CREB3L2 ///	CREB3L2	chr7	0.0325642	1.56077
3041892	NM_015550 // OSBPL3 /// NM_145320 // OSBPL3 /// NM_145321 // OSBPL3 /// NM_1453[illegible]	OSBPL3	chr7	0.00653168	1.55727
3080092	NM_170606 // MLL3 /// ENST00000262189 // MLL3 /// AY024361 // MLL3 /// AF264750	MLL3	chr7	0.0259589	1.55568
3072406	NM_016478 // ZC3HC1 /// ENST00000358303 // ZC3HC1 /// BC000190 // ZC3HC1 /// ENS	ZC3HC1	chr7	0.032479	1.55138
2995767	NM_194300 // CCDC129 /// ENST00000407970 // CCDC129 /// ENST00000319386 // CCDC1	CCDC129	chr7	0.00696982	1.5327
3011526	NM_021723 // ADAM22 /// NM_021722 // ADAM22 /// NM_016351 // ADAM22 /// NM_0041[illegible]	ADAM22	chr7	0.00693158	1.53247
3082225	NM_017760 // NCAPG2 /// ENST00000356309 // NCAPG2 /// ENST00000409423 // NCAPG2	NCAPG2	chr7	0.0343328	1.53218
3048895	NM_033224 // PURB /// ENST00000395699 // PURB /// BC101735 // PURB	PURB	chr7	0.0246908	1.53014
3048721	NM_013389 // NPC1L1 /// NM_001101648 // NPC1L1 /// ENST00000381160 // NPC1L1 //[illegible]	NPC1L1	chr7	0.0284886	1.52293

3027027	NM_016019 // LUC7L2 /// ENST00000354926 // LUC7L2 /// BC042625 // LUC7L2 /// ENS	LUC7L2	chr7	0.00767705	1.51829
3009522	NM_030570 // UPK3B /// NM_182684 // UPK3B /// ENST00000257632 // UPK3B /// ENST0	UPK3B	chr7	0.0356638	1.50857
3015534	NM_013439 // PILRA /// ENST00000198536 // PILRA /// AF161080 // PILRA	PILRA	chr7	0.0116653	1.50731
3030962	NM_198455 // SSPO /// AK124542 // SSPO	SSPO	chr7	0.0490136	1.50714
3009448	NM_001110354 // ZP3 /// NM_007155 // ZP3 /// ENST00000336517 // ZP3 /// ENST0000	ZP3	chr7	0.0324784	1.50485
3000291	NM_005856 // RAMP3 /// ENST00000242249 // RAMP3 /// BC022304 // RAMP3	RAMP3	chr7	0.0481994	1.50339
3082791	NM_004745 // DLGAP2 /// ENST00000421627 // DLGAP2 /// ENST00000357934 // DLGAP2	DLGAP2	chr8	0.0211341	2.04934
3144409	NM_175634 // RUNX1T1 /// ENST00000265814 // RUNX1T1	RUNX1T1	chr8	0.0213947	1.98047
3130307	NM_013357 // PURG /// NM_001015508 // PURG /// ENST00000339382 // PURG /// AF195	PURG	chr8	0.007449	1.87876
3108691	NM_001145860 // POP1 /// NM_001145861 // POP1 /// NM_015029 // POP1 /// ENST0000	POP1	chr8	0.000656363	1.86993
3104068	NM_024721 // ZFHX4 /// ENST00000455469 // ZFHX4 /// AB083343 // ZFHX4 /// ENST00	ZFHX4	chr8	0.0144426	1.81599
3085337	NM_003747 // TNKS /// ENST00000310430 // TNKS /// BC098394 // TNKS	TNKS	chr8	0.0139832	1.7731
3121778	NM_033225 // CSMD1 /// ENST00000318252 // CSMD1 /// AF333704 // CSMD1 /// ENST00	CSMD1	chr8	0.0170404	1.76466
3158018	NM_032789 // PARP10 /// ENST00000313028 // PARP10 /// AK303317 // PARP10	PARP10	chr8	0.00922509	1.75828
3134489	NR_024605 // EFCAB1 /// NM_024593 // EFCAB1 /// ENST00000262103 // EFCAB1 /// BC	EFCAB1	chr8	0.0249192	1.73958
3155728	NM_152888 // COL22A1 /// ENST00000303045 // COL22A1 /// AF406780 // COL22A1 ///	COL22A1	chr8	0.00401257	1.72489
3158061	NM_017570 // OPLAH /// ENST00000426825 // OPLAH /// BC142672 // OPLAH /// ENST00	OPLAH	chr8	0.0308835	1.71484
3089774	NM_001160036 // RHOBTB2 /// NM_001160037 // RHOBTB2 /// NM_015178 // RHOBTB2 //	RHOBTB2	chr8	0.0381591	1.71348
3158042	NM_032789 // PARP10 /// ENST00000313028 // PARP10 /// AK303317 // PARP10	PARP10	chr8	0.0251803	1.70144
3119979	NM_198572 // SPATC1 /// NM_001134374 // SPATC1 /// ENST00000447830 // SPATC1 //	SPATC1	chr8	0.0446594	1.68932
3114620	NM_007218 // RNF139 /// ENST00000303545 // RNF139 /// AF064801 // RNF139	RNF139	chr8	0.0417688	1.68566
3132346	NM_031940 // TM2D2 /// NM_001024380 // TM2D2 /// NM_001024381 // TM2D2 /// ENST0	TM2D2	chr8	0.0409489	1.67906
3129631	NM_015254 // KIF13B /// ENST00000357808 // KIF13B /// AF279865 // KIF13B /// ENS	KIF13B	chr8	0.0374455	1.65326
3153725	NM_001115 // ADCY8 /// ENST00000286355 // ADCY8 /// ENST00000377928 // ADCY8 //	ADCY8	chr8	0.00418646	1.64372
3113485	NM_022045 // MTBP /// ENST00000305949 // MTBP /// BC013136 // MTBP	MTBP	chr8	0.00108318	1.63955
3157790	NM_182706 // SCRIB /// NM_015356 // SCRIB /// ENST00000320476 // SCRIB /// ENST0	SCRIB	chr8	0.0244881	1.63388
3157850	NM_178564 // NRBP2 /// ENST00000452183 // NRBP2 /// ENST00000327830 // NRBP2 //	NRBP2	chr8	0.023741	1.62982
3148986	NM_014379 // KCNV1 /// ENST00000297404 // KCNV1 /// AF167082 // KCNV1	KCNV1	chr8	0.0297184	1.62704
3111712	NM_004215 // EBAG9 /// NM_198120 // EBAG9 /// ENST00000395785 // EBAG9 /// ENST0	EBAG9	chr8	0.0114325	1.62428
3124109	NM_178857 // RP1L1 /// ENST00000382483 // RP1L1 /// AY168341 // RP1L1	RP1L1	chr8	0.0440656	1.62139
3147781	NM_030780 // SLC25A32 /// NM_015420 // DCAF13 /// ENST00000297578 // SLC25A32 /	SLC25A32	chr8	0.0329088	1.60763
3111066	NM_181354 // OXR1 /// BC032710 // OXR1 /// AK292899 // OXR1 /// ENST00000312046	OXR1	chr8	0.046786	1.59228
3096218	NM_001135694 // VDAC3 /// NM_005662 // VDAC3 /// ENST00000022615 // VDAC3 /// EN	VDAC3	chr8	0.0145753	1.589
3158084	NM_017570 // OPLAH /// ENST00000426825 // OPLAH /// BC142672 // OPLAH /// ENST00	OPLAH	chr8	0.0436179	1.57903
3119606	NM_052924 // RHPN1 /// ENST00000289013 // RHPN1 /// BC025767 // RHPN1	RHPN1	chr8	0.0199774	1.56745
3129167	NM_018492 // PBK /// ENST00000301905 // PBK /// BC015191 // PBK	PBK	chr8	0.00342192	1.55944
3094393	NM_032777 // GPR124 /// ENST00000412232 // GPR124 /// AF378755 // GPR124 /// ENS	GPR124	chr8	0.0147952	1.55189
3140642	NM_001164380 // STAU2 /// NM_001164381 // STAU2 /// NM_001164382 // STAU2 /// NM	STAU2	chr8	0.0117954	1.54357
3091030	NM_004331 // BNIP3L /// ENST00000380629 // BNIP3L /// AF067396 // BNIP3L /// ENS	BNIP3L	chr8	0.004474	1.54255
3096159	NM_001556 // IKBKB /// ENST00000342222 // IKBKB /// AB209090 // IKBKB /// ENST00	IKBKB	chr8	0.00910366	1.53985
3124405	NM_053279 // FAM167A /// ENST00000284486 // FAM167A /// ENST00000398342 // FAM16	FAM167A	chr8	0.022165	1.52782
3108652	NM_173549 // C8orf47 /// NM_001170806 // C8orf47 /// BC062359 // C8orf47 /// ENS	C8orf47	chr8	0.041302	1.52756
3133478	NM_018105 // THAP1 /// NM_199003 // THAP1 /// ENST00000345117 // THAP1 /// ENST0	THAP1	chr8	0.0296954	1.51384
3147536	NM_005655 // KLF10 /// NM_001032282 // KLF10 /// ENST00000285407 // KLF10 /// EN	KLF10	chr8	0.0162879	1.5094
3131920	NM_023034 // WHSC1L1 /// ENST00000317025 // WHSC1L1 /// AF332469 // WHSC1L1 ///	WHSC1L1	chr8	0.0368024	1.50906
3090046	NM_016612 // SLC25A37 /// ENST00000290075 // SLC25A37 /// AY032628 // SLC25A37	SLC25A37	chr8	0.0462948	1.5047
3121792	NM_033225 // CSMD1 /// ENST00000318252 // CSMD1 /// AF333704 // CSMD1 /// ENST00	CSMD1	chr8	0.00555729	1.50042
3181392	NM_004473 // FOXE1 /// ENST00000375123 // FOXE1 /// U89995 // FOXE1	FOXE1	chr9	0.00144631	3.92692
3166651	NM_212558 // TMEM215 /// ENST00000342743 // TMEM215	TMEM215	chr9	0.00725786	3.37937
3186009	NM_032888 // COL27A1 /// ENST00000356083 // COL27A1 /// AY149237 // COL27A1 ///	COL27A1	chr9	0.0272559	2.89049
3204475	NM_004629 // FANCG /// ENST00000378643 // FANCG /// BC011623 // FANCG /// ENST00	FANCG	chr9	0.0239864	2.11481

3189753	NM_032293 // GARNL3 /// ENST00000373387 // GARNL3 /// AK296246 // GARNL3 /// AK2	GARNL3	chr9	0.0407766	1.96339
3194695	BC007542 // KIAA1984	KIAA1984	chr9	0.0174958	1.95638
3168085	NM_001216 // CA9 /// ENST00000378357 // CA9 /// BC014950 // CA9	CA9	chr9	0.0103644	1.92745
3228387	NM_000368 // TSC1 /// NM_001162426 // TSC1 /// NM_001162427 // TSC1 /// ENST0000	TSC1	chr9	0.0360857	1.91741
3220906	NM_022486 // SUSD1 /// ENST00000374270 // SUSD1 /// BC060770 // SUSD1 /// ENST00	SUSD1	chr9	0.020531	1.88348
3191879	NM_031426 // AIF1L /// ENST00000247291 // AIF1L /// BC021253 // AIF1L /// AY3590	AIF1L	chr9	0.0405797	1.84931
3227578	NM_033387 // FAM78A /// ENST00000372271 // FAM78A /// ENST00000247295 // FAM78A	FAM78A	chr9	0.0214873	1.8297
3186974	NM_138554 // TLR4 /// NR_024168 // TLR4 /// ENST00000355622 // TLR4 /// AK290053	TLR4	chr9	0.00552892	1.82936
3229483	NM_182974 // GLT6D1 /// ENST00000371763 // GLT6D1 /// AY336054 // GLT6D1	GLT6D1	chr9	0.020711	1.80835
3194951	NM_021138 // TRAF2 /// ENST00000247668 // TRAF2 /// AK294607 // TRAF2 /// AK2983	TRAF2	chr9	0.0196101	1.79469
3164044	NM_001040272 // ADAMTSL1 /// ENST00000380548 // ADAMTSL1 /// ENST00000380559 //	ADAMTSL1	chr9	0.00751064	1.7928
3230559	NM_018998 // FBXW5 /// ENST00000325285 // FBXW5 /// ENST00000443788 // FBXW5 //	FBXW5	chr9	0.00347839	1.79269
3191354	NM_014286 // NCS1 /// ENST00000372398 // FREQ /// AF134479 // NCS1	NCS1	chr9	0.0444583	1.76639
3179689	AK128259 // C9orf89	C9orf89	chr9	0.0307615	1.74118
3195074	NM_207510 // LCNL1 /// ENST00000408973 // LCNL1 /// AK127236 // LCNL1 /// ENST00	LCNL1	chr9	0.0144032	1.72642
3186081	NM_032888 // COL27A1 /// ENST00000356083 // COL27A1 /// AY149237 // COL27A1 ///	COL27A1	chr9	0.0135329	1.72379
3168861	NM_012203 // GRHPR /// ENST00000318158 // GRHPR /// ENST00000377824 // GRHPR	GRHPR	chr9	0.00120646	1.71892
3214891	NM_001393 // ECM2 /// ENST00000344604 // ECM2 /// BX537976 // ECM2	ECM2	chr9	0.0211525	1.69215
3225342	NM_173690 // SCAI /// NM_001144877 // SCAI /// ENST00000336505 // SCAI /// ENST0	SCAI	chr9	0.00862927	1.69087
3191834	NM_006059 // LAMC3 /// ENST00000361069 // LAMC3 /// AF041835 // LAMC3 /// ENST00	LAMC3	chr9	0.00769348	1.68419
3229944	NM_001039707 // SDCCAG3 /// NM_006643 // SDCCAG3 /// NM_001039708 // SDCCAG3 //	SDCCAG3	chr9	0.0068034	1.68196
3212707	---	0	chr9	0.00645536	1.67897
3164131	NM_001040272 // ADAMTSL1 /// ENST00000380548 // ADAMTSL1 /// ENST00000380559 //	ADAMTSL1	chr9	0.0353134	1.64742
3184588	NM_001004065 // AKAP2 /// NM_001136562 // AKAP2 /// NM_007203 // PALM2-AKAP2 //	AKAP2	chr9	0.036029	1.6374
3175511	NM_001097634 // GCNT1 /// NM_001490 // GCNT1 /// NM_001097633 // GCNT1 /// NM_00	GCNT1	chr9	0.00820936	1.63333
3227659	NM_031432 // UCK1 /// ENST00000372215 // UCK1 /// ENST00000372211 // UCK1 /// EN	UCK1	chr9	0.00695135	1.63027
3195106	BC132809 // C9orf139 /// NM_207511 // C9orf139 /// ENST00000314330 // C9orf139	C9orf139	chr9	0.00747037	1.62232
3230776	NM_178448 // C9orf140 /// ENST00000409687 // C9orf140 /// DQ150361 // C9orf140 /	C9orf140	chr9	0.0467549	1.61595
3193881	NM_014811 // KIAA0649 /// ENST00000356818 // KIAA0649 /// BC047935 // KIAA0649 /	KIAA0649	chr9	0.0184361	1.61233
4050655	NM_006647 // NOXA1 /// ENST00000341349 // NOXA1 /// BC041594 // NOXA1 /// ENST00	NOXA1	chr9	0.00631875	1.60021
3191862	NM_006059 // LAMC3 /// ENST00000361069 // LAMC3 /// AF041835 // LAMC3 /// ENST00	LAMC3	chr9	0.00451274	1.59808
3210155	AK299691 // C9orf41 /// BC034033 // C9orf41 /// BX647386 // C9orf41 /// ENST0000	C9orf41	chr9	0.0357132	1.58913
3190367	NM_005094 // SLC27A4 /// ENST00000300456 // SLC27A4 /// BC065003 // SLC27A4 ///	SLC27A4	chr9	0.019049	1.5855
3195066	NM_207510 // LCNL1 /// ENST00000408973 // LCNL1 /// AK127236 // LCNL1 /// ENST00	LCNL1	chr9	0.0366652	1.57868
3160905	NM_004972 // JAK2 /// ENST00000381652 // JAK2 /// AF058925 // JAK2	JAK2	chr9	0.0206604	1.57025
3159451	NM_203447 // DOCK8 /// ENST00000453981 // DOCK8 /// AB191037 // DOCK8 /// ENST00	DOCK8	chr9	0.00245078	1.56007
3189865	NM_001005373 // LRSAM1 /// ENST00000300417 // LRSAM1 /// AY358830 // LRSAM1 ///	LRSAM1	chr9	0.0279899	1.539
3190413	NM_030914 // URM1 /// NM_001135947 // URM1 /// ENST00000452446 // URM1 /// ENST0	URM1	chr9	0.043964	1.53329
3176722	NM_001001670 // FLJ46321 /// ENST00000344803 // FLJ46321	FLJ46321	chr9	0.0205548	1.53275
3174145	NM_153267 // MAMDC2 /// ENST00000377182 // MAMDC2 /// BC063634 // MAMDC2	MAMDC2	chr9	0.000938675	1.53112
3191222	NM_006676 // USP20 /// ENST00000315480 // USP20	USP20	chr9	0.0417607	1.5256
3205021	NM_019897 // OR2S2 /// ENST00000341959 // OR2S2 /// BC104869 // OR2S2 /// ENST00	OR2S2	chr9	0.0326139	1.52425
3198435	NM_002839 // PTPRD /// ENST00000381196 // PTPRD /// ENST00000356435 // PTPRD //	PTPRD	chr9	0.00567743	1.5227
3214751	NR_024020 // NOL8 /// NM_017948 // NOL8 /// ENST00000442668 // NOL8 /// AB105104	NOL8	chr9	0.0104164	1.51523
3219893	NM_002829 // PTPN3 /// NM_001145368 // PTPN3 /// NM_001145369 // PTPN3 /// NM_00	PTPN3	chr9	0.030501	1.50999
3184946	NM_004125 // DNAJC25-GNG10 /// NM_001015882 // DNAJC25 /// ENST00000313525 // DN	DNAJC25-GNG10	chr9	0.0355107	1.50013
4016814	NM_153448 // ESX1 /// ENST00000372588 // ESX1 /// AK097704 // ESX1	ESX1	chrX	0.00203901	3.34522
4017548	NM_001847 // COL4A6 /// NM_033641 // COL4A6 /// ENST00000372216 // COL4A6 /// EN	COL4A6	chrX	0.0037443	2.35608
3968758	NM_001142 // AMELX /// NM_182681 // AMELX /// NM_182680 // AMELX /// ENST0000038	AMELX	chrX	0.000964827	2.29346
4019603	NM_006978 // RNF113A /// ENST00000371442 // RNF113A /// X98253 // RNF113A	RNF113A	chrX	0.0472011	2.257
3995788	NM_001395 // DUSP9 /// ENST00000370167 // DUSP9 /// ENST00000342782 // DUSP9 //	DUSP9	chrX	0.00642629	1.92895

3990770	NM_003951 // SLC25A14 /// NM_022810 // SLC25A14 /// ENST00000218197 // SLC25A14	SLC25A14	chrX	0.0157398	1.91727
4017507	NM_031273 // TEX13B /// ENST00000302917 // TEX13B /// AF285598 // TEX13E	TEX13B	chrX	0.00431994	1.87287
3994933	NM_005342 // HMGB3 /// ENST00000325307 // HMGB3 /// BX537505 // HMGB3	HMGB3	chrX	0.015036	1.85097
3995945	NM_005393 // PLXNB3 /// NM_001163257 // PLXNB3 /// ENST00000361971 // PLXNB3 //	PLXNB3	chrX	0.00417472	1.84469
3989530	NM_007325 // GRIA3 /// NM_000828 // GRIA3 /// ENST00000371256 // GRIA3 /// ENST(	GRIA3	chrX	0.00222396	1.80422
3976932	NM_001032382 // PQBP1 /// NM_001032383 // PQBP1 /// NM_144495 // PQBP1 /// ENST(	PQBP1	chrX	0.0114752	1.8001
4003969	NM_152787 // TAB3 /// ENST00000378933 // MAP3K7IP3 /// ENST00000378930 // MAP3K7	TAB3	chrX	0.0154453	1.76514
3995912	NM_005393 // PLXNB3 /// NM_001163257 // PLXNB3 /// ENST00000361971 // PLXNB3 //	PLXNB3	chrX	0.0319442	1.76505
4022253	NM_001077188 // HS6ST2 /// NM_147175 // HS6ST2 /// ENST00000370836 // HS6ST2 //,	HS6ST2	chrX	0.00374976	1.74538
4011026	NM_007268 // VSIG4 /// NM_001100431 // VSIG4 /// ENST00000374737 // VSIG4 /// AK	VSIG4	chrX	0.0248832	1.74093
3991139	NM_016542 // MST4 /// NM_001042453 // MST4 /// NM_001042452 // MST4 /// ENST0000(	MST4	chrX	0.0211582	1.73503
4009713	NM_004463 // FGD1 /// ENST00000375135 // FGD1 /// U11690 // FGD1	FGD1	chrX	0.0132854	1.73267
3988742	NM_006667 // PGRMC1 /// ENST00000217971 // PGRMC1 /// BC034238 // PGRMC1	PGRMC1	chrX	0.0229194	1.73176
4027560	NM_001081573 // GAB3 /// NM_080612 // GAB3 /// ENST00000369575 // GAB3 /// AY057	GAB3	chrX	0.0414907	1.68816
4000905	AF226667 // CTPS2 /// ENST00000380207 // CTPS2	CTPS2	chrX	0.00284769	1.65171
3976796	NM_000377 // WAS /// ENST00000376701 // WAS /// U12707 // WAS	WAS	chrX	0.0467681	1.64743
4002390	NM_153270 // KLHL34 /// ENST00000379499 // KLHL34 /// BC109031 // KLHL34	KLHL34	chrX	0.0131681	1.64657
3987619	NM_001004308 // ZCCHC16 /// ENST00000340433 // ZCCHC16 /// BC137430 // ZCCHC16 /	ZCCHC16	chrX	0.00655232	1.63565
3968545	NM_001830 // CLCN4 /// ENST00000380833 // CLCN4 /// AB019432 // CLCN4	CLCN4	chrX	0.0496488	1.63447
4005424	NM_017745 // BCOR /// NM_001123383 // BCOR /// NM_001123384 // BCOR /// NM_00112	BCOR	chrX	0.0353276	1.63262
4027584	AJ223040 // CTAG2	CTAG2	chrX	0.04759	1.62655
3970034	NM_005314 // GRPR /// ENST00000380289 // GRPR /// M73481 // GRPR	GRPR	chrX	0.0200256	1.61388
3971463	NM_000444 // PHEX /// ENST00000379374 // PHEX /// U82970 // PHEX	PHEX	chrX	0.0446002	1.60166
4026124	NM_001011543 // MAGEA10 /// NM_021048 // MAGEA10 /// ENST00000244096 // MAGEA10	MAGEA10	chrX	0.0402662	1.59332
4015889	NM_014782 // ARMCX2 /// NM_177949 // ARMCX2 /// ENST00000330154 // ARMCX2 /// EN	ARMCX2	chrX	0.0412289	1.5925
4000885	AF226667 // CTPS2 /// ENST00000455276 // CTPS2 /// ENST00000380207 // CTPS2	CTPS2	chrX	0.0143209	1.56591
4017215	NM_024657 // MORC4 /// NM_001085354 // MORC4 /// ENST00000355610 // MORC4 /// EN	MORC4	chrX	0.0319216	1.55007
3986660	NM_001170553 // VSIG1 /// NM_182607 // VSIG1 /// ENST00000217957 // VSIG1 /// AK	VSIG1	chrX	0.0488107	1.54836
3994304	NM_002025 // AFF2 /// NM_001169122 // AFF2 /// NM_001169123 // AFF2 /// NM_0011(	AFF2	chrX	0.00375812	1.53583
3982814	NM_003022 // SH3BGRL /// ENST00000373212 // SH3BGRL /// BC103762 // SH3BGRL	SH3BGRL	chrX	0.0400305	1.53262
4012284	NM_018486 // HDAC8 /// NM_001166419 // HDAC8 /// NM_001166420 // HDAC8 /// NM_0(	HDAC8	chrX	0.0275612	1.53135
3970756	NM_006240 // PPEF1 /// NM_152224 // PPEF1 /// NM_152226 // PPEF1 /// ENST0000036	PPEF1	chrX	0.0232719	1.52107
3969076	NM_001039091 // PRPS2 /// NM_002765 // PRPS2 /// ENST00000380668 // PRPS2 /// EN	PRPS2	chrX	0.0301699	1.5182
3976315	NM_001654 // ARAF /// ENST00000377045 // ARAF /// AB208831 // ARAF /// ENST0000(	ARAF	chrX	0.0482527	1.51627
3985204	NR_002216 // NXF4	NXF4	chrX	0.021619	1.51506
4018780	NM_020871 // LRCH2 /// ENST00000371928 // LRCH2 /// ENST00000317135 // LRCH2 //,	LRCH2	chrX	0.033432	1.50683
3979934	NM_000044 // AR /// ENST00000374690 // AR /// M23263 // AR	AR	chrX	0.00734008	1.50372
4011013	NM_007268 // VSIG4 /// NM_001100431 // VSIG4 /// ENST00000374737 // VSIG4 /// AK	VSIG4	chrX	0.0475862	1.5031
4036157	---	0	chrY	0.0202138	1.95935

GROUP 2. mRNA microarry results: Down-regulated > 1.5 fold

Probeset ID	gene_assignment	Gene Symbol	seqname	Raw p-value (T21)	Fold-Change(T21 vs. Ctrl)
2315638	NM_080605 // B3GALT6 /// ENST00000379198 // B3GALT6	B3GALT6	chr1	0.0470841	-1.56537
2316581	ENST00000434662 // RER1 /// ENST00000378512 // RER1	RER1	chr1	0.00717942	-1.51728
2317284	NM_014448 // ARHGEF16 /// ENST00000378378 // ARHGEF16 /// ENST00000340401 // ARH	ARHGEF16	chr1	0.0235832	-1.65533
2317394	NM_005427 // TP73 /// NM_001126240 // TP73 /// NM_001126241 // TP73 /// ENST0000(	TP73	chr1	0.0218948	-1.85338
2317522	AB028912 // DFFB /// ENST00000430539 // DFFB /// ENST00000339350 // DFFB /// ENS	DFFB	chr1	0.0170852	-1.71896
2317746	NM_018836 // AJAP1 /// NM_001042478 // AJAP1 /// ENST00000378191 // AJAP1 /// EN	AJAP1	chr1	0.0034162	-1.75729

2318179	NM_207396 // RNF207 /// ENST00000377939 // RNF207 /// AK056658 // RNF207 /// ENS	RNF207	chr1	0.020962	-1.96391
2318368	NM_005341 // ZBTB48 /// ENST00000377674 // ZBTB48 /// L16896 // ZBTB48 /// ENST(	ZBTB48	chr1	0.0366118	-1.52777
2318406	NM_153812 // PHF13 /// ENST00000377648 // PHF13 /// BC038516 // PHF1:	PHF13	chr1	0.039424	-1.62492
2318414	NM_153812 // PHF13 /// ENST00000377648 // PHF13 /// BC038516 // PHF1:	PHF13	chr1	0.015275	-1.5109
2318744	NM_007262 // PARK7 /// NM_001123377 // PARK7 /// ENST00000338639 // PARK7 /// EN	PARK7	chr1	0.0257755	-1.55663
2319608	NM_001105562 // UBE4B /// NM_006048 // UBE4B /// ENST00000343090 // UBE4B /// EN	UBE4B	chr1	0.00457474	-1.73682
2320399	NM_198545 // C1orf187 /// ENST00000294485 // C1orf187 /// AK075558 // C1orf187	C1orf187	chr1	0.0128237	-1.69394
2320718	NM_001243 // TNFRSF8 /// NM_152942 // TNFRSF8 /// ENST00000263932 // TNFRSF8 //.	TNFRSF8	chr1	0.0200006	-1.68857
2320802	NM_015378 // VPS13D /// NM_018156 // VPS13D /// ENST00000011700 // VPS13D /// EN	VPS13D	chr1	0.0319745	-1.51903
2321252	NM_012231 // PRDM2 /// NM_015866 // PRDM2 /// NM_001135610 // PRDM2 /// ENST000(	PRDM2	chr1	0.0347429	-1.60425
2322139	NM_015001 // SPEN /// ENST00000375759 // SPEN /// AF356524 // SPEN	SPEN	chr1	0.0387668	-1.58454
2322144	NM_015001 // SPEN /// ENST00000375759 // SPEN /// AF356524 // SPEN	SPEN	chr1	0.00372194	-1.67843
2322834	NM_016233 // PADI3 /// ENST00000375460 // PADI3 /// AB026831 // PADI3	PADI3	chr1	0.00374868	-1.80052
2322835	NM_016233 // PADI3 /// ENST00000375460 // PADI3 /// AB026831 // PADI3	PADI3	chr1	0.0351948	-2.07034
2323613	NM_001040125 // PQLC2 /// NM_017765 // PQLC2 /// NM_001040126 // PQLC2 /// ENST(	PQLC2	chr1	0.0249599	-1.58841
2324586	NM_007352 // CELA3B /// ENST00000337107 // CELA3B /// BC005216 // CELA3B /// ENS	CELA3B	chr1	0.0155971	-1.81825
2324878	NM_001114101 // C1QC /// NM_172369 // C1QC /// ENST00000374639 // C1QC /// ENST(	C1QC	chr1	0.0161588	-1.65389
2324981	NM_017449 // EPHB2 /// NM_004442 // EPHB2 /// ENST00000400191 // EPHB2 /// ENST(	EPHB2	chr1	0.0171187	-1.80749
2325403	NM_021180 // GRHL3 /// NM_198173 // GRHL3 /// NM_198174 // GRHL3 /// ENST000003€	GRHL3	chr1	0.0137091	-1.5746
2325452	NM_020448 // NIPAL3 /// ENST00000374399 // NIPAL3 /// BC063583 // NIPAL3 /// BX5	NIPAL3	chr1	0.00633757	-1.7961
2326052	NM_020379 // MAN1C1 /// ENST00000374332 // MAN1C1 /// AF261655 // MAN1C1 /// ENS	MAN1C1	chr1	0.0166486	-1.64768
2327290	NM_004848 // C1orf38 /// NM_001105556 // C1orf38 /// ENST00000373921 // C1orf38	C1orf38	chr1	0.0139828	-1.52653
2327825	NM_133178 // PTPRU /// NM_133177 // PTPRU /// NM_005704 // PTPRU /// ENST0000034	PTPRU	chr1	0.0256038	-1.65407
2328825	NM_003757 // EIF3I /// ENST00000373586 // EIF3I /// BC003140 // EIF3	EIF3I	chr1	0.0140164	-1.58706
2329061	NM_020888 // KIAA1522 /// ENST00000401073 // KIAA1522 /// AB040955 // KIAA1522 .	KIAA1522	chr1	0.00353908	-1.65378
2329095	NM_022753 // S100PBP /// NM_001017406 // S100PBP /// ENST00000373476 // S100PBP	S100PBP	chr1	0.0168544	-1.61614
2330243	NM_014466 // TEKT2 /// ENST00000207457 // TEKT2 /// AF054910 // TEKT2	TEKT2	chr1	0.031157	-1.62891
2330248	NM_014466 // TEKT2 /// ENST00000207457 // TEKT2 /// AF054910 // TEKT2	TEKT2	chr1	0.0229019	-1.62367
2330308	NM_018067 // MAP7D1 /// ENST00000373151 // MAP7D1 /// BC106053 // MAP7D1 /// ENS	MAP7D1	chr1	0.0259357	-1.50888
2330775	ENST00000373055 // CDCA8 /// BC001651 // CDCA8	CDCA8	chr1	0.010178	-1.57627
2331327	NM_012090 // MACF1 /// ENST00000361689 // MACF1 /// AB029290 // MACF1 /// ENST0(	MACF1	chr1	0.00234828	-1.50158
2332535	NM_173642 // RIMKLA /// ENST00000431473 // RIMKLA /// BC039737 // RIMKLA /// ENS	RIMKLA	chr1	0.0224983	-1.51239
2333516	NM_174963 // ST3GAL3 /// NM_174968 // ST3GAL3 /// NM_174971 // ST3GAL3 /// ENST(	ST3GAL3	chr1	0.00338947	-1.79626
2333551	NM_174963 // ST3GAL3 /// NM_006279 // ST3GAL3 /// NM_174964 // ST3GAL3 /// NM_17	ST3GAL3	chr1	0.0110206	-1.58904
2335166	NM_012186 // FOXE3 /// ENST00000335071 // FOXE3 /// AF275722 // FOXE3	FOXE3	chr1	0.00755685	-1.50319
2335184	NM_004474 // FOXD2 /// ENST00000334793 // FOXD2 /// AF042832 // FOXD2	FOXD2	chr1	0.0116571	-1.85894
2337176	NM_015547 // ACOT11 /// NM_147161 // ACOT11 /// ENST00000371316 // ACOT11 /// EN	ACOT11	chr1	0.0321384	-1.68716
2339744	NM_032437 // EFCAB7 /// ENST00000371088 // EFCAB7 /// AK292220 // EFCAB7 /// ENS	EFCAB7	chr1	0.0343335	-1.67128
2339873	NM_005012 // ROR1 /// NM_001083592 // ROR1 /// ENST00000371079 // ROR1 /// ENST(	ROR1	chr1	0.0222485	-1.78883
2340403	NM_014787 // DNAJC6 /// ENST00000395325 // DNAJC6 /// ENST00000371069 // DNAJC6	DNAJC6	chr1	0.000946839	-1.59491
2340859	NM_020948 // MIER1 /// NM_001146110 // MIER1 /// NM_001077700 // MIER1 /// NM_0(	MIER1	chr1	0.0278426	-1.81195
2342488	NM_001001933 // LHX8 /// ENST00000294638 // LHX8 /// BC040321 // LHX8	LHX8	chr1	0.00876309	-1.9064
2342490	NM_001001933 // LHX8 /// ENST00000294638 // LHX8 /// BC040321 // LHX8	LHX8	chr1	0.0305053	-1.57821
2342740	NM_152996 // ST6GALNAC3 /// NM_001160011 // ST6GALNAC3 /// ENST00000328299 // ST	ST6GALNAC3	chr1	0.00164909	-2.11125
2345017	NM_006536 // CLCA2 /// ENST00000370565 // CLCA2 /// BC041096 // CLCA2	CLCA2	chr1	0.000325424	-1.74649
2347635	NM_152369 // SLC44A3 /// ENST00000394212 // SLC44A3	SLC44A3	chr1	0.0443965	-1.551
2348597	NM_017734 // PALMD /// ENST00000263174 // PALMD /// AF262379 // PALMD	PALMD	chr1	0.00824775	-1.54334
2349218	NR_033424 // HEJ1 /// AF395440 // HEJ1	HEJ1	chr1	0.00243959	-1.70023
2350774	NM_153340 // ATXN7L2 /// ENST00000369870 // ATXN7L2 /// AK090460 // ATXN7L2 ///	ATXN7L2	chr1	0.0415271	-1.64813
2350795	NM_182580 // CYB561D1 /// NM_001134403 // CYB561D1 /// NM_001134404 // CYB561D1	CYB561D1	chr1	0.0356782	-1.54243
2351185	ENST00000369794 // FAM40A	FAM40A	chr1	0.0256339	-1.60721

2353511	NM_000701 // ATP1A1 /// NM_001160233 // ATP1A1 /// NM_001160234 // ATP1A1 /// EN	ATP1A1	chr1	0.00574369	-1.54618
2353778	NM_003594 // TTF2 /// ENST00000369466 // TTF2 /// AK291017 // TTF2	TTF2	chr1	0.0445801	-1.52546
2354677	NM_006623 // PHGDH /// ENST00000369409 // PHGDH /// AK093306 // PHGDH /// ENST00	PHGDH	chr1	0.00655443	-1.91442
2356106	NM_213653 // HFE2 /// NM_145277 // HFE2 /// NM_202004 // HFE2 /// ENST000003367	HFE2	chr1	0.0472093	-1.78466
2356207	NM_003846 // PEX11B /// ENST00000369306 // PEX11B /// AF093670 // PEX11B	PEX11B	chr1	0.0199611	-2.06987
2358378	NM_004425 // ECM1 /// NM_022664 // ECM1 /// ENST00000369047 // ECM1 /// ENST000	ECM1	chr1	0.00862113	-1.62208
2358555	NM_001145415 // SETDB1 /// NM_012432 // SETDB1 /// ENST00000368969 // SETDB1 //	SETDB1	chr1	0.0173635	-1.54115
2358626	NM_021222 // PRUNE /// ENST00000271620 // PRUNE /// AF123539 // PRUNE /// BC0634	PRUNE	chr1	0.0440338	-1.52633
2358985	NM_020770 // CGN /// ENST00000271636 // CGN /// BC146657 // CGN	CGN	chr1	0.0353486	-1.50568
2359457	NM_030663 // SMCP /// ENST00000368765 // SMCP /// BC014593 // SMCP	SMCP	chr1	0.00486802	-1.63367
2359998	NM_130898 // CREB3L4 /// ENST00000368607 // CREB3L4 /// ENST00000271889 // CREB3	CREB3L4	chr1	0.0177931	-1.53815
2360277	NM_000565 // IL6R /// NM_181359 // IL6R /// ENST00000368485 // IL6R /// ENST000	IL6R	chr1	0.0169258	-1.8999
2360289	NM_000565 // IL6R /// NM_181359 // IL6R /// ENST00000368485 // IL6R /// ENST000	IL6R	chr1	0.0231619	-1.6486
2360627	NM_207191 // ADAM15 /// NM_003815 // ADAM15 /// NM_207194 // ADAM15 /// NM_2071	ADAM15	chr1	0.0492113	-1.51958
2360738	NM_025058 // TRIM46 /// ENST00000334634 // TRIM46 /// AK304542 // TRIM46 /// AK2	TRIM46	chr1	0.00700005	-1.734
2361325	NM_170707 // LMNA /// NM_005572 // LMNA /// NM_170708 // LMNA /// ENST000003683	LMNA	chr1	0.00825704	-1.58997
2361659	NM_198427 // BCAN /// ENST00000361588 // BCAN /// AF229053 // BCAN	BCAN	chr1	0.0447287	-1.50729
2361717	NM_015997 // C1orf66 /// NM_001142560 // C1orf66 /// ENST00000368216 // C1orf66	C1orf66	chr1	0.0186845	-1.8072
2361774	NM_001012331 // NTRK1 /// NM_002529 // NTRK1 /// NM_001007792 // NTRK1 /// ENST0	NTRK1	chr1	0.0103976	-1.57078
2361842	NM_144702 // C1orf92 /// ENST00000337428 // C1orf92 /// AK292342 // C1orf92	C1orf92	chr1	0.00242662	-1.8477
2362503	NM_021189 // CADM3 /// NM_001127173 // CADM3 /// ENST00000368125 // CADM3 /// EN	CADM3	chr1	0.0112845	-1.62915
2362520	NM_001122951 // DARC /// NM_002036 // DARC /// ENST00000368121 // DARC /// ENST0	DARC	chr1	0.0308304	-2.00183
2364194	NM_003115 // UAP1 /// ENST00000367926 // UAP1 /// S73498 // UAP1 /// AB011004 //	UAP1	chr1	0.00267936	-1.63338
2366361	NR_024160 // MGC4473	MGC4473	chr1	0.0270267	-1.50181
2369157	NM_170692 // RASAL2 /// ENST00000263528 // RASAL2 /// BC110611 // RASAL2	RASAL2	chr1	0.00340606	-1.64474
2369751	NM_173509 // FAM163A /// ENST00000341785 // FAM163A /// AY902195 // FAM163A	FAM163A	chr1	0.010753	-1.85161
2370021	---	0	chr1	0.00191359	-1.50703
2371257	NM_173156 // SMG7 /// NM_201568 // SMG7 /// NM_201569 // SMG7 /// NM_001174061	SMG7	chr1	0.0290994	-1.6672
2373798	NM_133494 // NEK7 /// ENST00000367385 // NEK7 /// AB062450 // NEK7	NEK7	chr1	0.00648328	-1.51326
2376237	NM_001005388 // NFASC /// NM_001160331 // NFASC /// NM_001160332 // NFASC /// NM	NFASC	chr1	0.0204726	-1.68565
2376315	NM_005076 // CNTN2 /// ENST00000331830 // CNTN2 /// BC129986 // CNTN2	CNTN2	chr1	0.00832282	-1.93825
2376392	ENST00000330675 // TMCC2 /// ENST00000367159 // TMCC2	TMCC2	chr1	0.00393343	-1.58053
2377258	NM_001114752 // CD55 /// ENST00000314754 // CD55	CD55	chr1	0.0467863	-2.96656
2377490	NM_002389 // CD46 /// NM_172359 // CD46 /// NM_172351 // CD46 /// NM_153826 // C	CD46	chr1	0.0443379	-1.71319
2378939	NM_016448 // DTL /// ENST00000366991 // DTL /// AF195765 // DTL /// ENST0000042	DTL	chr1	0.00397222	-1.86389
2379136	NM_001674 // ATF3 /// NM_001040619 // ATF3 /// ENST00000426071 // ATF3 /// ENST0	ATF3	chr1	0.0150913	-1.68623
2381181	NM_018650 // MARK1 /// ENST00000366917 // MARK1 /// BC114478 // MARK1 /// ENST00	MARK1	chr1	0.0259615	-1.51347
2381205	NM_018650 // MARK1 /// ENST00000366917 // MARK1 /// BC114478 // MARK1 /// ENST00	MARK1	chr1	0.0261051	-1.55067
2384062	NM_052843 // OBSCN /// NM_001098623 // OBSCN /// ENST00000422127 // OBSCN /// EN	OBSCN	chr1	0.00531653	-1.71786
2386836	NM_080738 // EDARADD /// ENST00000359362 // EDARADD /// AY028914 // EDARADD	EDARADD	chr1	0.030604	-1.612
2387072	NM_000254 // MTR /// ENST00000366577 // MTR /// U73338 // MTR /// ENST0000041774	MTR	chr1	0.0163662	-1.50152
2387317	NM_001035 // RYR2 /// ENST00000366574 // RYR2 /// ENST00000355794 // RYR2 /// EN	RYR2	chr1	0.0379652	-1.55471
2390080	NM_004895 // NLRP3 /// NM_183395 // NLRP3 /// NM_001079821 // NLRP3 /// NM_00112	NLRP3	chr1	0.0164416	-1.63333
2390172	AY358215 // OR9H1P	OR9H1P	chr1	0.0325774	-1.53458
2390497	NM_024836 // ZNF672 /// BC068506 // ZNF672	ZNF672	chr1	0.0114214	-1.58401
2391305	NM_030649 // ACAP3 /// ENST00000354700 // ACAP3 /// ENST00000353662 // ACAP3 //	ACAP3	chr1	0.00406751	-1.55419
2392163	AK024162 // FLJ14100	FLJ14100	chr1	0.048569	-1.67072
2394372	NM_015102 // NPHP4 /// ENST00000378156 // NPHP4 /// ENST00000378169 // NPHP4 //	NPHP4	chr1	0.014418	-1.54678
2394534	NM_015557 // CHD5 /// ENST00000262450 // CHD5 /// AF425231 // CHD5 /// ENST0000	CHD5	chr1	0.0234061	-1.73201
2394764	NM_020631 // PLEKHG5 /// NM_198681 // PLEKHG5 /// NM_001042663 // PLEKHG5 /// NM	PLEKHG5	chr1	0.00269842	-1.91465
2395367	NM_012102 // RERE /// NM_001042681 // RERE /// ENST00000440864 // RERE /// ENST0	RERE	chr1	0.0108963	-1.81991

2395926	NM_001009566 // CLSTN1 /// NM_014944 // CLSTN1 /// ENST00000377298 // CLSTN1 //.	CLSTN1	chr1	0.0263731	-1.56235
2396024	NM_032368 // LZIC /// ENST00000377223 // LZIC /// ENST00000400903 // LZIC /// EN	LZIC	chr1	0.0252495	-1.50995
2397952	NM_004431 // EPHA2 /// ENST00000358432 // EPHA2 /// ENST00000407976 // EPHA2 //.	EPHA2	chr1	0.019557	-1.58277
2398019	NM_153213 // ARHGEF19 /// ENST00000270747 // ARHGEF19 /// BC040640 // ARHGEF19 /	ARHGEF19	chr1	0.0271439	-1.50471
2398844	NM_007365 // PADI2 /// ENST00000375486 // PADI2 /// AB030176 // PADI2 /// ENST0C	PADI2	chr1	0.0315706	-1.62119
2399749	NM_012067 // AKR7A3 /// ENST00000361640 // AKR7A3 /// AF040639 // AKR7A3	AKR7A3	chr1	0.0113498	-1.61534
2400016	NM_014589 // PLA2G2E /// ENST00000375116 // PLA2G2E /// AF189279 // PLA2G2E	PLA2G2E	chr1	0.0432409	-1.70181
2400459	NM_003760 // EIF4G3 /// ENST00000264211 // EIF4G3 /// ENST00000400422 // EIF4G3	EIF4G3	chr1	0.00151458	-1.9397
2400892	NM_005529 // HSPG2 /// ENST00000374695 // HSPG2 /// M85289 // HSPG2 /// ENST000C	HSPG2	chr1	0.041945	-1.63266
2400896	NM_005529 // HSPG2 /// ENST00000374695 // HSPG2 /// M85289 // HSPG2 /// ENST000C	HSPG2	chr1	0.028147	-1.64188
2400936	NM_005529 // HSPG2 /// ENST00000374695 // HSPG2 /// M85289 // HSPG2 /// ENST000C	HSPG2	chr1	0.020206	-1.61123
2401427	NM_017707 // ASAP3 /// NM_001143778 // ASAP3 /// ENST00000437606 // ASAP3 /// EN	ASAP3	chr1	0.0162407	-1.81456
2401748	NM_152372 // MYOM3 /// ENST00000374434 // MYOM3 /// BC067101 // MYOM3 /// ENST0C	MYOM3	chr1	0.0206757	-1.79598
2402425	NM_024037 // C1orf135 /// BC000209 // C1orf135 /// ENST00000374298 // C1orf135	C1orf135	chr1	0.00976674	-1.53622
2402605	NM_145345 // UBXN11 /// NM_183008 // UBXN11 /// NM_001077262 // UBXN11 /// ENST(	UBXN11	chr1	0.0187975	-1.86119
2403828	NM_016011 // MECR /// NM_001024732 // MECR /// ENST00000263702 // MECR /// ENSTC	MECR	chr1	0.0234165	-1.51333
2404228	NM_014654 // SDC3 /// ENST00000339394 // SDC3 /// ENST00000358423 // SDC3 /// EN	SDC3	chr1	0.00800311	-1.56844
2404229	NM_014654 // SDC3 /// ENST00000339394 // SDC3 /// ENST00000336798 // SDC3 /// AE	SDC3	chr1	0.0229075	-1.82338
2404234	NM_014654 // SDC3 /// ENST00000339394 // SDC3 /// AB007937 // SDC3	SDC3	chr1	0.0217752	-1.50388
2404562	NM_001856 // COL16A1 /// ENST00000373672 // COL16A1 /// M92642 // COL16A1 /// EN	COL16A1	chr1	0.0194406	-1.60615
2405004	NM_023009 // MARCKSL1 /// ENST00000329421 // MARCKSL1 /// BC007904 // MARCKSL1	MARCKSL1	chr1	0.0246686	-1.55206
2405232	NM_003680 // YARS /// ENST00000373477 // YARS /// AK125213 // YARS	YARS	chr1	0.0206761	-1.57318
2406037	NM_007167 // ZMYM6 /// ENST00000357182 // ZMYM6 /// AK299293 // ZMYM6 /// AF0554	ZMYM6	chr1	0.00935342	-1.72728
2407241	NM_001038633 // RSPO1 /// ENST00000356545 // RSPO1 /// ENST00000445126 // RSPO1	RSPO1	chr1	0.0184405	-1.58763
2407258	BC018109 // C1orf109 /// NM_017850 // C1orf109 /// ENST00000358011 // C1orf109	C1orf109	chr1	0.0250285	-1.56909
2408273	NM_001852 // COL9A2 /// ENST00000372748 // COL9A2 /// BC136326 // COL9A2 /// ENS	COL9A2	chr1	0.0262821	-1.81633
2408688	NM_024503 // HIVEP3 /// NM_001127714 // HIVEP3 /// ENST00000372583 // HIVEP3 //.	HIVEP3	chr1	0.0335645	-1.60027
2408916	NM_014947 // FOXJ3 /// ENST00000361346 // FOXJ3 /// ENST00000361776 // FOXJ3 //.	FOXJ3	chr1	0.0345146	-1.63938
2409876	NM_003738 // PTCH2 /// NM_001166292 // PTCH2 /// ENST00000372192 // PTCH2 /// AY	PTCH2	chr1	0.0196544	-1.52483
2409987	NM_024602 // HECTD3 /// ENST00000372172 // HECTD3 /// AK301124 // HECTD3 /// ENS	HECTD3	chr1	0.0344624	-1.54226
2412935	NM_001009881 // ZCCHC11 /// NM_015269 // ZCCHC11 /// NM_001009882 // ZCCHC11 //.	ZCCHC11	chr1	0.0143981	-1.55899
2424113	NM_001839 // CNN3 /// ENST00000370206 // CNN3 /// BC025372 // CNN3 /// ENST0000C	CNN3	chr1	0.018273	-1.95194
2428367	NM_017744 // ST7L /// NM_138727 // ST7L /// NM_138728 // ST7L /// NM_138729 // S	ST7L	chr1	0.031894	-1.57194
2431035	NM_005518 // HMGCS2 /// NM_001166107 // HMGCS2 /// ENST00000369406 // HMGCS2 //.	HMGCS2	chr1	0.00355158	-1.78362
2433389	NM_016361 // ACP6 /// ENST00000369238 // ACP6 /// AB209248 // ACP6 /// ENST0000C	ACP6	chr1	0.0305026	-1.50106
2434185	NM_001145862 // MTMR11 /// NM_181873 // MTMR11 /// ENST00000439741 // MTMR11 //.	MTMR11	chr1	0.0151484	-1.74453
2434644	NM_001668 // ARNT /// NM_178427 // ARNT /// ENST00000358595 // ARNT /// ENST0000	ARNT	chr1	0.00497672	-1.63693
2434766	NM_018379 // FAM63A /// NM_001040217 // FAM63A /// NM_001163258 // FAM63A /// NM	FAM63A	chr1	0.0252826	-1.55674
2434960	NM_002651 // PI4KB /// ENST00000368875 // PI4KB /// ENST00000271657 // PI4KB //.	PI4KB	chr1	0.0162178	-1.96109
2434992	NM_000449 // RFX5 /// NM_001025603 // RFX5 /// ENST00000290524 // RFX5 /// ENST(	RFX5	chr1	0.0105166	-1.6457
2435963	NM_020393 // PGLYRP4 /// ENST00000359650 // PGLYRP4 /// BC142636 // PGLYRP4 ///	PGLYRP4	chr1	0.00226094	-1.87753
2436053	NM_002963 // S100A7 /// ENST00000368722 // S100A7 /// ENST00000368723 // S100A7	S100A7	chr1	0.0152979	-1.50144
2436348	NM_181715 // CRTC2 /// ENST00000368633 // CRTC2 /// ENST00000303569 // CRTC2 //.	CRTC2	chr1	0.00191702	-1.7232
2436976	NM_138300 // PYGO2 /// ENST00000368457 // PYGO2 /// BC006132 // PYGO2 /// ENST0C	PYGO2	chr1	0.00812607	-1.5545
2437016	NM_183001 // SHC1 /// NM_001130040 // SHC1 /// ENST00000368445 // SHC1 /// ENST(	SHC1	chr1	0.0066096	-2.06403
2437030	NM_144622 // DCST2 /// ENST00000368424 // DCST2 /// AK057496 // DCST2 /// ENST0C	DCST2	chr1	0.0174663	-1.74
2437199	NM_007112 // THBS3 /// ENST00000368378 // THBS3 /// L38969 // THBS3 /// ENST000C	THBS3	chr1	0.00879663	-1.95005
2438064	NM_015327 // SMG5 /// ENST00000361813 // SMG5 /// BC038296 // SMG5	SMG5	chr1	0.0292816	-1.56704
2438542	NM_004494 // HDGF /// NM_001126050 // HDGF /// NM_001126051 // HDGF /// ENST000C	HDGF	chr1	0.0442927	-1.88582
2439912	ENST00000198587 // IGSF9	IGSF9	chr1	0.0143313	-1.58205
2440485	NM_016946 // F11R /// ENST00000368026 // F11R /// ENST00000335772 // F11R /// EN	F11R	chr1	0.0108642	-1.72057

2440558	NM_001025598 // ARHGAP30 /// NM_181720 // ARHGAP30 /// ENST00000368013 // ARHGAF	ARHGAP30	chr1	0.00446031	-1.72187
2440561	NM_001025598 // ARHGAP30 /// NM_181720 // ARHGAP30 /// ENST00000368013 // ARHGAF	ARHGAP30	chr1	0.0381708	-1.69378
2443394	NM_000130 // F5 /// ENST00000367797 // F5 /// M14335 // F5 /// ENST00000367796	F5	chr1	0.0455227	-1.83401
2443399	NM_000130 // F5 /// ENST00000367797 // F5 /// M14335 // F5 /// ENST00000367796	F5	chr1	0.0173839	-1.51534
2444150	ENST00000367728 // PIGC	PIGC	chr1	0.0329237	-1.58634
2449113	NM_003783 // B3GALT2 /// ENST00000367434 // B3GALT2 /// BC022507 // B3GALT2	B3GALT2	chr1	0.0228231	-1.50362
2450615	NM_000069 // CACNA1S /// ENST00000362061 // CACNA1S /// L33798 // CACNA1S /// EN	CACNA1S	chr1	0.029058	-1.69708
2450633	NM_000069 // CACNA1S /// ENST00000362061 // CACNA1S /// L33798 // CACNA1S /// EN	CACNA1S	chr1	0.0277323	-1.52931
2451974	NM_014935 // PLEKHA6 /// ENST00000272203 // PLEKHA6 /// BC152475 // PLEKHA6 ///	PLEKHA6	chr1	0.0171316	-1.60138
2452899	NM_006893 // LGTN /// ENST00000271764 // LGTN /// AF220417 // LGTN /// ENST0000C	LGTN	chr1	0.0118835	-1.60914
2453024	NM_002644 // PIGR /// ENST00000356495 // PIGR /// AF272149 // PIGR	PIGR	chr1	0.00443373	-1.84534
2453401	NM_025179 // PLXNA2 /// ENST00000367033 // PLXNA2 /// BC132676 // PLXNA2	PLXNA2	chr1	0.00203681	-1.59419
2454581	NM_015434 // INTS7 /// ENST00000366994 // INTS7 /// BC030716 // INTS7 /// BC0205	INTS7	chr1	0.0140959	-1.5333
2458563	NM_014698 // TMEM63A /// ENST00000366835 // TMEM63A /// BC030245 // TMEM63A	TMEM63A	chr1	0.0348889	-1.73282
2459426	NM_024319 // C1orf35 /// ENST00000272139 // C1orf35 /// ENST00000366749 // C1orf	C1orf35	chr1	0.0294521	-1.58168
2459589	NM_016102 // TRIM17 /// NM_001024940 // TRIM17 /// NM_001024941 // TRIM17 /// EN	TRIM17	chr1	0.00492278	-1.71487
2465898	NM_001004692 // OR2T12 /// ENST00000317996 // OR2T12	OR2T12	chr1	0.0136455	-1.72035
2468361	NM_080657 // RSAD2 /// ENST00000382040 // RSAD2 /// AF442151 // RSAD2 /// ENST0C	RSAD2	chr2	0.0462288	-1.55573
2468892	NM_003887 // ASAP2 /// ENST00000281419 // ASAP2	ASAP2	chr2	0.0266262	-1.71023
2469579	AK127836 // ATP6V1C2	ATP6V1C2	chr2	0.0417117	-1.53693
2469900	NM_014668 // GREB1 /// ENST00000381486 // GREB1 /// ENST00000234142 // GREB1 //	GREB1	chr2	0.0264581	-1.61911
2472805	NM_052920 // KLHL29 /// ENST00000288548 // KLHL29 /// AB067508 // KLHL29	KLHL29	chr2	0.033103	-1.60095
2474346	NM_004341 // CAD /// ENST00000264705 // CAD /// BC065510 // CAD /// ENST00000403	CAD	chr2	0.0227759	-1.52671
2474564	NM_013392 // NRBP1 /// ENST00000233557 // NRBP1 /// ENST00000379852 // NRBP1 //	NRBP1	chr2	0.0375976	-1.58349
2475087	NM_153021 // PLB1 /// NM_001170585 // PLB1 /// ENST00000327757 // PLB1 /// BC065	PLB1	chr2	0.0438107	-1.76753
2477397	NM_144736 // C2orf56 /// NM_001083946 // C2orf56 /// ENST00000002125 // C2orf56	C2orf56	chr2	0.000679141	-1.59119
2480434	NM_001430 // EPAS1 /// ENST00000263734 // EPAS1 /// BC051338 // EPAS1 /// ENST0C	EPAS1	chr2	0.0402478	-1.53409
2484460	NM_032506 // KIAA1841 /// ENST00000356719 // KIAA1841 /// ENST00000295031 // KIA	KIAA1841	chr2	0.0114294	-1.67706
2485128	NM_014562 // OTX1 /// ENST00000366671 // OTX1 /// ENST00000282549 // OTX1 /// BC	OTX1	chr2	0.0394186	-1.76038
2486987	NM_001007231 // ARHGAP25 /// NM_014882 // ARHGAP25 /// NM_001166276 // ARHGAP25	ARHGAP25	chr2	0.0103163	-1.52759
2487481	NM_178439 // GMCL1 /// ENST00000282570 // GMCL1 /// BC007420 // GMCL1	GMCL1	chr2	0.0168811	-1.56413
2487910	NM_012476 // VAX2 /// ENST00000234392 // VAX2 /// Y17791 // VAX2	VAX2	chr2	0.000210131	-2.21429
2488080	NM_005791 // MPHOSPH10 /// ENST00000244230 // MPHOSPH10 /// BC126389 // MPHOSPH1	MPHOSPH10	chr2	0.0367706	-1.6796
2489270	NM_031288 // INO80B /// ENST00000233331 // INO80B /// AK295901 // INO80B /// ENS	INO80B	chr2	0.0450481	-1.82249
2491047	NR_003663 // FUNDC2P2 /// BC067852 // FUNDC2P2	FUNDC2P2	chr2	0.0480315	-1.70221
2494571	NM_212481 // ARID5A /// ENST00000357485 // ARID5A /// AK300173 // ARID5A /// AK3	ARID5A	chr2	0.0405442	-1.5755
2497289	NM_003048 // SLC9A2 /// ENST00000233969 // SLC9A2 /// BC136377 // SLC9A2	SLC9A2	chr2	0.0437433	-1.65419
2500702	NM_153214 // FBLN7 /// NM_001128165 // FBLN7 /// ENST00000331203 // FBLN7 /// EN	FBLN7	chr2	0.00191422	-1.79473
2501078	NM_014439 // IL1F7 /// NM_173204 // IL1F7 /// ENST00000263326 // IL1F7 /// ENST(	IL1F7	chr2	0.0402903	-1.9666
2501185	NM_032556 // IL1F10 /// NM_173161 // IL1F10 /// ENST00000341010 // IL1F10 /// EN	IL1F10	chr2	0.00332002	-2.41794
2506988	NM_002410 // MGAT5 /// ENST00000409645 // MGAT5 /// ENST00000281923 // MGAT5 //	MGAT5	chr2	0.0122243	-1.59222
2507407	---	0	chr2	0.0492369	-1.53311
2509978	NM_004522 // KIF5C /// ENST00000435030 // KIF5C /// ENST00000334436 // KIF5C //	KIF5C	chr2	0.00904616	-1.58491
2512763	NM_006593 // TBR1 /// ENST00000389554 // TBR1 /// BC104844 // TBR1 /// ENST0000C	TBR1	chr2	0.00200049	-1.52652
2512769	NM_006593 // TBR1 /// ENST00000389554 // TBR1 /// BC104844 // TBR1 /// ENST0000C	TBR1	chr2	0.0489195	-1.5473
2519235	NM_002210 // ITGAV /// NM_001144999 // ITGAV /// NM_001145000 // ITGAV /// ENSTC	ITGAV	chr2	0.0235172	-1.53465
2526977	NM_001142310 // TMEM169 /// NM_001142311 // TMEM169 /// NM_138390 // TMEM169 //	TMEM169	chr2	0.00127666	-1.55341
2527170	NM_014140 // SMARCAL1 /// NM_001127207 // SMARCAL1 /// ENST00000357276 // SMARCA	SMARCAL1	chr2	0.0429708	-1.50733
2527570	NM_198483 // RUFY4 /// ENST00000344321 // RUFY4 /// ENST00000457754 // RUFY4 //	RUFY4	chr2	0.00137838	-1.82726
2527609	NM_152862 // ARPC2 /// ENST00000315717 // ARPC2 /// AK290268 // ARPC2 /// ENST0C	ARPC2	chr2	0.0148808	-1.56657
2527665	NM_001077191 // GPBAR1 /// NM_001077194 // GPBAR1 /// NM_170699 // GPBAR1 /// EN	GPBAR1	chr2	0.0231406	-1.62597

2527666	NM_001077191 // GPBAR1 /// NM_001077194 // GPBAR1 /// NM_170699 // GPBAR1 /// EN	GPBAR1	chr2	0.00366604	-2.19928
2527998	NM_015690 // STK36 /// ENST00000295709 // STK36 /// ENST00000440309 // STK36 //.	STK36	chr2	0.00303063	-1.80946
2528445	NM_006736 // DNAJB2 /// ENST00000336576 // DNAJB2 /// AK296297 // DNAJB2 /// AK2	DNAJB2	chr2	0.0190417	-1.52381
2528543	NM_005876 // SPEG /// NM_001173476 // SPEG /// ENST00000312358 // SPEG /// BC006	SPEG	chr2	0.0266231	-1.74118
2528741	NM_052902 // STK11IP /// ENST00000456909 // STK11IP /// ENST00000295641 // STK11	STK11IP	chr2	0.031126	-2.34265
2531265	NM_007237 // SP140 /// ENST00000392045 // SP140 /// U36500 // SP140 /// ENST0000	SP140	chr2	0.0446852	-1.51187
2532885	NM_000541 // SAG /// ENST00000409110 // SAG /// ENST00000449594 // SAG /// ENST0	SAG	chr2	0.0284412	-1.83634
2532943	NM_003648 // DGKD /// NM_152879 // DGKD /// ENST00000409813 // DGKD /// ENST0000	DGKD	chr2	0.0195338	-1.63673
2534539	NM_005855 // RAMP1 /// ENST00000254661 // RAMP1 /// BC000548 // RAMP1 /// ENST00	RAMP1	chr2	0.0118294	-1.53731
2534811	AF242456 // TRAF3IP1	TRAF3IP1	chr2	0.0249602	-1.5399
2534820	NM_015650 // TRAF3IP1 /// NM_001139490 // TRAF3IP1 /// ENST00000373327 // TRAF3	TRAF3IP1	chr2	0.0151262	-1.5616
2535805	NM_002081 // GPC1 /// ENST00000264039 // GPC1 /// BC051279 // GPC1 /// X54232 /.	GPC1	chr2	0.0286835	-1.60477
2536124	NM_001080437 // SNED1 /// ENST00000310397 // SNED1 /// AF439717 // SNED1 /// ENS	SNED1	chr2	0.0316087	-1.511
2536193	NM_002712 // PPP1R7 /// ENST00000407025 // PPP1R7 /// ENST00000234038 // PPP1R7	PPP1R7	chr2	0.0230076	-1.77798
2536306	ENST00000429791 // SEPT2 /// ENST00000420786 // SEPT2 /// BC043180 // SEPT2	40788	chr2	0.0187713	-1.55248
2536432	---	0	chr2	0.0189621	-2.10908
2536470	---	0	chr2	0.0331632	-1.63544
2543012	NM_022460 // HS1BP3 /// ENST00000304031 // HS1BP3 /// BC050636 // HS1BP3 /// ENS	HS1BP3	chr2	0.00676003	-2.08402
2543172	NM_000384 // APOB /// ENST00000233242 // APOB /// J02610 // APOB	APOB	chr2	0.0250241	-1.57163
2543190	NM_000384 // APOB /// ENST00000233242 // APOB /// J02610 // APOB	APOB	chr2	0.0450798	-1.54093
2544491	NM_004036 // ADCY3 /// ENST00000260600 // ADCY3 /// BC126235 // ADCY3 /// AF0338	ADCY3	chr2	0.026832	-1.54045
2544843	NM_021907 // DTNB /// NM_033147 // DTNB /// NM_033148 // DTNB /// NM_183360 // D	DTNB	chr2	0.0314516	-1.6479
2545156	NM_001145168 // GPR113 /// NM_001145169 // GPR113 /// NM_153835 // GPR113 /// EN	GPR113	chr2	0.00982377	-1.59315
2545797	NM_144631 // ZNF513 /// ENST00000323703 // ZNF513 /// BC052282 // ZNF513 /// ENS	ZNF513	chr2	0.0110392	-1.5257
2546026	NM_014860 // SUPT7L /// ENST00000337768 // SUPT7L /// ENST00000404798 // SUPT7L	SUPT7L	chr2	0.036085	-1.62988
2546995	NM_024572 // GALNT14 /// ENST00000349752 // GALNT14 /// BC010659 // GALNT14 ///	GALNT14	chr2	0.0141417	-1.96877
2549099	NM_005633 // SOS1 /// ENST00000426016 // SOS1 /// ENST00000402219 // SOS1 /// L1	SOS1	chr2	0.000153638	-1.53662
2550189	NM_133329 // KCNG3 /// NM_172344 // KCNG3 /// ENST00000306078 // KCNG3 /// ENST0	KCNG3	chr2	0.0262633	-1.90165
2550329	NM_148962 // OXER1 /// ENST00000378661 // OXER1 /// BC063549 // OXER1	OXER1	chr2	0.013783	-1.74045
2550529	NM_006887 // ZFP36L2 /// ENST00000282388 // ZFP36L2 /// BC005010 // ZFP36L2	ZFP36L2	chr2	0.0296291	-1.54393
2553958	NM_001122964 // SMEK2 /// NM_020463 // SMEK2 /// ENST00000345102 // SMEK2 /// EN	SMEK2	chr2	0.00838219	-1.52853
2558771	NM_017482 // ADD2 /// NM_017484 // ADD2 /// ENST00000413157 // ADD2 /// ENST0000	ADD2	chr2	0.0260756	-1.68719
2560127	NM_006302 // MOGS /// NM_001146158 // MOGS /// ENST00000233616 // MOGS /// ENST0	MOGS	chr2	0.00179521	-1.90447
2560255	NM_181575 // AUP1 /// ENST00000377526 // AUP1 /// AK055566 // AUP1 /// ENST0000	AUP1	chr2	0.0179961	-1.71301
2561509	ENST00000433224 // LRRTM1 /// ENST00000295057 // LRRTM1 /// AY358310 // LRRTM1 /	LRRTM1	chr2	0.00188342	-1.98827
2565566	NM_144994 // ANKRD23 /// ENST00000443120 // ANKRD39 /// ENST00000418232 // ANKRD	ANKRD23	chr2	0.0095575	-1.66486
2565641	NM_001122646 // FAM178B /// NM_016490 // FAM178B /// NM_001172667 // FAM178B //	FAM178B	chr2	0.0243166	-1.60765
2567199	NM_198461 // LONRF2 /// ENST00000393437 // LONRF2 /// DQ020495 // LONRF2 /// ENS	LONRF2	chr2	0.0265872	-1.99517
2567250	NM_004854 // CHST10 /// ENST00000264249 // CHST10 /// ENST00000409701 // CHST10	CHST10	chr2	0.0187306	-1.61588
2567604	NM_173647 // RNF149 /// ENST00000295317 // RNF149	RNF149	chr2	0.0211519	-1.96842
2569011	ENST00000409032 // UXS1 /// AK027244 // UXS1	UXS1	chr2	0.0138045	-1.55502
2573134	NM_002980 // SCTR /// ENST00000019103 // SCTR /// BC035757 // SCTR	SCTR	chr2	0.0388955	-1.65381
2575167	NM_031445 // AMMECR1L /// ENST00000272647 // AMMECR1L /// ENST00000393001 // AMM	AMMECR1L	chr2	0.0177794	-1.56105
2579075	NM_018557 // LRP1B /// ENST00000389484 // LRP1B /// AF176832 // LRP1B /// ENST00	LRP1B	chr2	0.000483345	-1.83068
2579577	NM_014795 // ZEB2 /// NM_001171653 // ZEB2 /// ENST00000303660 // ZEB2 /// ENST0	ZEB2	chr2	0.0111934	-1.65297
2579591	NM_014795 // ZEB2 /// NM_001171653 // ZEB2 /// ENST00000303660 // ZEB2 /// ENST0	ZEB2	chr2	0.0046816	-1.67899
2586106	NM_004525 // LRP2 /// ENST00000263816 // LRP2 /// U33837 // LRP2	LRP2	chr2	0.0103633	-1.53571
2586784	ENST00000375258 // METTL8 /// ENST00000415699 // METTL8 /// ENST00000447486 // M	METTL8	chr2	0.0104409	-1.84193
2586871	NM_003705 // SLC25A12 /// ENST00000422440 // SLC25A12 /// BC016932 // SLC25A12 .	SLC25A12	chr2	0.0443398	-1.5299
2587005	NM_004405 // DLX2 /// ENST00000234198 // DLX2 /// AB208823 // DLX2	DLX2	chr2	0.042387	-1.62083
2594927	NM_001168221 // ALS2CR11 /// NM_152525 // ALS2CR11 /// NM_001168217 // ALS2CR11	ALS2CR11	chr2	0.0228875	-1.65998

2596168	NM_017759 // INO80D /// ENST00000403263 // INO80D /// BC136278 // INO80D /// ENS	INO80D	chr2	0.043954	-1.53597
2599649	NM_017521 // FEV /// ENST00000295727 // FEV /// BC023511 // FEV	FEV	chr2	0.0353382	-1.61447
2599682	NM_057093 // CRYBA2 /// ENST00000295728 // CRYBA2 /// BC006285 // CRYBA2	CRYBA2	chr2	0.0101547	-1.93468
2599706	NM_194302 // CCDC108 /// ENST00000341552 // CCDC108 /// ENST00000441968 // CCDC1	CCDC108	chr2	0.021788	-1.50179
2600024	NM_005689 // ABCB6 /// ENST00000265316 // ABCB6 /// BC000559 // ABCB6 /// ENST0C	ABCB6	chr2	0.0185219	-1.52597
2600133	NM_002846 // PTPRN /// ENST00000295718 // PTPRN /// L18983 // PTPRN /// AK291469	PTPRN	chr2	0.0357335	-1.58955
2600927	NM_000438 // PAX3 /// ENST00000409828 // PAX3	PAX3	chr2	0.0456146	-1.60935
2602171	NM_000092 // COL4A4 /// ENST00000396625 // COL4A4 /// ENST00000329662 // COL4A4	COL4A4	chr2	0.000843148	-2.72976
2602744	NM_017933 // PID1 /// NM_001100818 // PID1 /// ENST00000392054 // PID1 /// ENST(	PID1	chr2	0.0275855	-1.51033
2602927	NM_004238 // TRIP12 /// ENST00000283943 // TRIP12 /// BC113891 // TRIP12 /// BC1	TRIP12	chr2	0.0216596	-1.50011
2606052	NM_006037 // HDAC4 /// ENST00000345617 // HDAC4 /// BC039904 // HDAC4 /// ENST0C	HDAC4	chr2	0.0228205	-1.52528
2606135	NM_006037 // HDAC4 /// ENST00000345617 // HDAC4 /// BC039904 // HDAC4 /// ENST0C	HDAC4	chr2	0.00549229	-1.60593
2606621	NM_004544 // NDUFA10 /// ENST00000252711 // NDUFA10 /// AF087661 // NDUFA10 ///	NDUFA10	chr2	0.0192768	-1.73584
2608520	NM_001099952 // ITPR1 /// NM_002222 // ITPR1 /// NM_001168272 // ITPR1 /// BC144	ITPR1	chr3	0.0123447	-1.50078
2608767	NM_018184 // ARL8B /// ENST00000256496 // ARL8B /// BC063125 // ARL8B /// ENST0C	ARL8B	chr3	0.0366222	-1.76633
2610113	NM_153461 // IL17RC /// NM_153460 // IL17RC /// NM_032732 // IL17RC /// ENST000(	IL17RC	chr3	0.0394442	-1.78718
2610365	NM_001570 // IRAK2 /// ENST00000256458 // IRAK2 /// BC125184 // IRAK2	IRAK2	chr3	0.0481416	-1.66362
2610400	NM_014760 // TATDN2 /// ENST00000287652 // TATDN2 /// ENST00000448281 // TATDN2	TATDN2	chr3	0.0247926	-1.7251
2610592	NM_014229 // SLC6A11 /// ENST00000254488 // SLC6A11	SLC6A11	chr3	0.0371543	-1.61946
2612374	NM_033083 // EAF1 /// ENST00000396842 // EAF1 /// BC041329 // EAF1 /// ENST0000C	EAF1	chr3	0.030854	-1.53291
2616132	NM_005508 // CCR4 /// ENST00000330953 // CCR4 /// BC071751 // CCR4	CCR4	chr3	0.00494012	-1.50515
2616345	NM_013374 // PDCD6IP /// NM_001162429 // PDCD6IP /// NR_027868 // PDCD6IP /// EN	PDCD6IP	chr3	0.00834837	-1.64996
2617701	NM_005108 // XYLB /// ENST00000207870 // XYLB /// BC137076 // XYLB /// ENST0000(	XYLB	chr3	0.00860867	-1.69199
2617754	NM_001106 // ACVR2B /// ENST00000352511 // ACVR2B	ACVR2B	chr3	0.0347326	-1.88369
2619316	NM_004624 // VIPR1 /// ENST00000325123 // VIPR1 /// EF577396 // VIPR1 /// BC0644	VIPR1	chr3	0.0209418	-1.52046
2620027	NM_173826 // C3orf23 /// NM_001029839 // C3orf23 /// NM_001029840 // C3orf23 //.	C3orf23	chr3	0.0224882	-1.6452
2620122	NM_018651 // ZNF167 /// NM_025169 // ZNF167 /// AY280798 // ZNF167	ZNF167	chr3	0.0321612	-1.72627
2621196	NM_015175 // NBEAL2 /// ENST00000450053 // NBEAL2 /// AY358455 // NBEAL2 /// ENS	NBEAL2	chr3	0.0165791	-1.6203
2621368	NM_015466 // PTPN23 /// ENST00000265562 // PTPN23 /// AF290614 // PTPN23 /// ENS	PTPN23	chr3	0.0304528	-1.83244
2621929	NM_018031 // WDR6 /// ENST00000395474 // WDR6 /// BC101707 // WDR6 /// BC113467	WDR6	chr3	0.0475325	-1.60013
2622047	NM_178173 // CCDC36 /// NM_001135197 // CCDC36 /// ENST00000296449 // CCDC36 //.	CCDC36	chr3	0.00707349	-1.58545
2622256	NM_022064 // RNF123 /// ENST00000327697 // RNF123 /// ENST00000389066 // RNF123	RNF123	chr3	0.00568753	-1.70291
2622475	NM_005778 // RBM5 /// ENST00000347869 // RBM5 /// ENST00000433556 // RBM5 /// EN	RBM5	chr3	0.00401401	-1.51467
2622663	NM_002070 // GNAI2 /// NM_001166425 // GNAI2 /// ENST00000313601 // GNAI2 /// CR	GNAI2	chr3	0.0293786	-1.63841
2622675	NM_002070 // GNAI2 /// NM_001166425 // GNAI2 /// ENST00000313601 // GNAI2 /// CR	GNAI2	chr3	0.0497933	-1.56396
2622706	NM_004636 // SEMA3B /// NM_001005914 // SEMA3B /// ENST00000414456 // SEMA3B //.	SEMA3B	chr3	0.0460187	-1.5992
2622726	NM_004636 // SEMA3B /// NM_001005914 // SEMA3B /// ENST00000414456 // SEMA3B //.	SEMA3B	chr3	0.0288843	-1.91312
2623040	NM_004947 // DOCK3 /// ENST00000266037 // DOCK3 /// AY254099 // DOCK3	DOCK3	chr3	0.00375352	-1.74356
2623276	NM_015926 // TEX264 /// NM_001129884 // TEX264 /// NR_024012 // TEX264 /// ENST(	TEX264	chr3	0.0213312	-1.52674
2623948	NM_015136 // STAB1 /// ENST00000321725 // STAB1 /// AB052956 // STAB1	STAB1	chr3	0.0114219	-1.60813
2624189	NM_002217 // ITIH3 /// ENST00000449956 // ITIH3 /// BC107814 // ITIH3 /// ENST0C	ITIH3	chr3	0.00145929	-1.91317
2628838	NM_198159 // MITF /// NM_198177 // MITF /// NM_006722 // MITF /// NM_000248 // M	MITF	chr3	0.049203	-2.23534
2629352	NM_018971 // GPR27 /// NM_173359 // EIF4E3 /// ENST00000304411 // GPR27 /// AB04	GPR27	chr3	0.0351618	-1.70997
2633776	NM_006070 // TFG /// ENST00000240851 // TFG /// BC009241 // TFG	TFG	chr3	0.0117242	-1.59708
2636622	NM_001690 // ATP6V1A /// ENST00000273398 // ATP6V1A /// AF113129 // ATP6V1A	ATP6V1A	chr3	0.00828282	-1.63818
2638028	NM_016589 // C3orf1 /// ENST00000264244 // C3orf1 /// AF139077 // C3orf1	C3orf1	chr3	0.00124575	-1.50848
2639079	NM_017554 // PARP14 /// ENST00000310276 // PARP14 /// ENST00000398162 // PARP14	PARP14	chr3	0.0341169	-1.53311
2639257	NM_006810 // PDIA5 /// NR_028444 // PDIA5 /// ENST00000316218 // PDIA5 /// BC001	PDIA5	chr3	0.0360304	-1.54911
2641380	NM_014049 // ACAD9 /// NR_033426 // ACAD9 /// ENST00000308982 // ACAD9 /// ENST(	ACAD9	chr3	0.0466101	-1.5433
2643118	NM_003571 // BFSP2 /// ENST00000302334 // BFSP2 /// BC113518 // BFSP2	BFSP2	chr3	0.0183357	-1.73221
2643122	NM_003571 // BFSP2 /// ENST00000302334 // BFSP2 /// BC113518 // BFSP2	BFSP2	chr3	0.0439885	-1.55464

2643640	NM_004441 // EPHB1 /// ENST00000398015 // EPHB1 /// L40636 // EPHB1 /// ENST000(	EPHB1	chr3	0.0423405	-1.54447
2644612	NM_012219 // MRAS /// NM_001085049 // MRAS /// ENST00000289104 // MRAS /// ENST(	MRAS	chr3	0.049711	-1.52558
2646127	ENST00000309575 // CHST2 /// AB021124 // CHST2	CHST2	chr3	0.00856611	-1.63861
2646129	NM_004267 // CHST2 /// ENST00000309575 // CHST2 /// AB021124 // CHST2	CHST2	chr3	0.0160292	-1.64137
2647764	NM_032025 // EIF2A /// ENST00000273435 // EIF2A /// AF212241 // EIF2A /// ENST0(	EIF2A	chr3	0.0197424	-1.60712
2653903	BC026181 // ZNF639	ZNF639	chr3	0.00190199	-1.82341
2655623	NM_014693 // ECE2 /// NM_001037324 // ECE2 /// NM_001100120 // ECE2 /// NM_0011(	ECE2	chr3	0.0220417	-1.58101
2656258	NM_021627 // SENP2 /// ENST00000296257 // SENP2 /// AK304402 // SENP2 /// ENST0(	SENP2	chr3	0.0015034	-2.95524
2658618	NM_005524 // HES1 /// ENST00000232424 // HES1	HES1	chr3	0.0330634	-1.56921
2658789	NM_153690 // FAM43A /// ENST00000329759 // FAM43A /// BC064989 // FAM43A	FAM43A	chr3	0.0405493	-1.8246
2659678	NR_024388 // LOC152217 /// ENST00000449854 // LOC152217	LOC152217	chr3	0.00441537	-1.74721
2662742	NM_001001331 // ATP2B2 /// NM_001683 // ATP2B2 /// ENST00000360273 // ATP2B2 //,	ATP2B2	chr3	0.0138175	-1.72536
2663451	ENST00000450726 // IQSEC1	IQSEC1	chr3	0.02407	-1.77926
2663596	BC067089 // NUP210	NUP210	chr3	0.0471746	-1.57248
2663601	NM_024923 // NUP210 /// ENST00000254508 // NUP210 /// BC067089 // NUP21(	NUP210	chr3	0.0442398	-1.54406
2664364	NM_005677 // COLQ /// NM_080538 // COLQ /// NM_080539 // COLQ /// ENST000003837[illegible]	COLQ	chr3	0.0164322	-1.55952
2664918	NM_001134381 // TBC1D5 /// NM_014744 // TBC1D5 /// NM_001134380 // TBC1D5 /// EN	TBC1D5	chr3	0.00568436	-1.74806
2666231	NM_000461 // THRB /// NM_001128176 // THRB /// NM_001128177 // THRB /// ENST000(	THRB	chr3	0.0412852	-1.5755
2667219	NM_022461 // AZI2 /// NM_001134432 // AZI2 /// NM_001134433 // AZI2 /// ENST000(	AZI2	chr3	0.0406837	-1.59825
2669976	NM_194293 // XIRP1 /// ENST00000340369 // XIRP1 /// AY375160 // XIRP1 /// ENST0(	XIRP1	chr3	0.016411	-1.58612
2670727	NM_000729 // CCK /// NM_001174138 // CCK /// ENST00000396169 // CCK /// ENST000(	CCK	chr3	0.037843	-1.66127
2672247	NM_147129 // ALS2CL /// NM_182775 // ALS2CL /// ENST00000458221 // ALS2CL /// EN	ALS2CL	chr3	0.00971967	-1.66276
2673447	NM_000094 // COL7A1 /// ENST00000328333 // COL7A1 /// L02870 // COL7A1 /// ENST(	COL7A1	chr3	0.00674441	-1.92914
2673835	NM_018031 // WDR6 /// NM_001009996 // DALRD3 /// NM_018114 // DALRD3 /// ENST00(	WDR6	chr3	0.0173166	-1.69703
2673981	NM_005051 // QARS /// ENST00000306125 // QARS /// BC001567 // QARS /// BC000394	QARS	chr3	0.00591627	-1.53972
2674339	---	0	chr3	0.0456727	-1.74658
2674363	---	0	chr3	0.0279937	-1.53917
2674520	NM_000481 // AMT /// NM_001164710 // AMT /// NM_001164712 // AMT /// NR_028435 ,	AMT	chr3	0.00500717	-1.68027
2674926	NM_002447 // MST1R /// ENST00000296474 // MST1R /// AK303234 // MST1R /// ENST0(	MST1R	chr3	0.019562	-1.74381
2675130	NM_003549 // HYAL3 /// ENST00000336307 // HYAL3 /// AF040710 // HYAL3 /// AF036(	HYAL3	chr3	0.0417438	-1.57782
2675348	NM_001005505 // CACNA2D2 /// NM_006030 // CACNA2D2 /// NM_001174051 // CACNA2D2	CACNA2D2	chr3	0.000872085	-1.73487
2676003	NM_017442 // TLR9 /// AF259262 // TLR9 /// ENST00000423623 // TWF2	TLR9	chr3	0.0329198	-1.81051
2676163	NM_020163 // SEMA3G /// ENST00000231721 // SEMA3G /// AB029496 // SEMA3G	SEMA3G	chr3	0.0114736	-1.66875
2676529	NM_001005159 // SFMBT1 /// NM_001005158 // SFMBT1 /// NM_016329 // SFMBT1 /// EN	SFMBT1	chr3	0.0133623	-1.66781
2682146	NM_173359 // EIF4E3 /// NM_001134649 // EIF4E3 /// NM_001134650 // EIF4E3 /// EN	EIF4E3	chr3	0.0417816	-1.53635
2687780	NM_001777 // CD47 /// NM_001025079 // CD47 /// ENST00000398260 // CD47 /// ENST(	CD47	chr3	0.0090713	-3.51447
2687806	NM_001777 // CD47 /// NM_198793 // CD47 /// NM_001025079 // CD47 /// ENST0000039	CD47	chr3	0.00187311	-1.9829
2692263	NM_001031702 // SEMA5B /// ENST00000357599 // SEMA5B /// AY358124 // SEMA5B ///	SEMA5B	chr3	0.000570389	-1.73102
2692327	NM_183357 // ADCY5 /// ENST00000309879 // ADCY5 /// AK124691 // ADCY5	ADCY5	chr3	0.0296285	-1.56098
2694030	NM_007283 // MGLL /// NM_001003794 // MGLL /// ENST00000398104 // MGLL /// ENST(	MGLL	chr3	0.03774	-1.9294
2694349	NM_032638 // GATA2 /// ENST00000341105 // GATA2 /// BC051342 // GATA2	GATA2	chr3	0.0404801	-1.63578
2694367	NM_007354 // C3orf27 /// ENST00000356020 // C3orf27 /// AF008192 // C3orf27	C3orf27	chr3	0.0302439	-1.55094
2694775	BC066975 // C3orf25 /// ENST00000326085 // C3orf25 /// ENST00000439888 // C3orf2	C3orf25	chr3	0.00131977	-1.64183
2695754	---	0	chr3	0.0497896	-1.73935
2697659	NM_023067 // FOXL2 /// ENST00000330315 // FOXL2 /// BC062549 // FOXL2	FOXL2	chr3	0.0443483	-1.55016
2700463	NM_015472 // WWTR1 /// NM_001168280 // WWTR1 /// ENST00000360632 // WWTR1 /// BC	WWTR1	chr3	0.0365719	-1.52356
2704473	NM_001105077 // MECOM /// NM_005241 // MECOM /// NM_001105078 // MECOM /// NM_0(	MECOM	chr3	0.04128	-1.67398
2704814	NM_024727 // LRRC31 /// ENST00000316428 // LRRC31 /// AY358138 // LRRC31 /// ENS	LRRC31	chr3	0.00504096	-1.93913
2707982	NM_015078 // MCF2L2 /// ENST00000328913 // MCF2L2 /// ENST00000447025 // MCF2L2	MCF2L2	chr3	0.0219427	-1.70546
2708483	NM_004366 // CLCN2 /// NM_001171087 // CLCN2 /// NM_001171088 // CLCN2 /// NM_0(	CLCN2	chr3	0.0223521	-1.66957
2709647	NM_001879 // MASP1 /// ENST00000337774 // MASP1	MASP1	chr3	0.0130174	-1.51342

2710612	NM_021101 // CLDN1 /// ENST00000295522 // CLDN1 /// AF101051 // CLDN1	CLDN1	chr3	0.0311307	-1.65806
2711639	NM_004488 // GP5 /// ENST00000323007 // GP5 /// ENST00000401815 // GP5 /// L1123	GP5	chr3	0.0409902	-1.58257
2712386	NM_018406 // MUC4 /// NM_004532 // MUC4 /// NM_138297 // MUC4 /// ENST00000349б(	MUC4	chr3	0.0482967	-1.52305
2714173	NM_000283 // PDE6B /// NM_001145291 // PDE6B /// NM_001145292 // PDE6B /// ENST(	PDE6B	chr4	0.0108346	-1.70857
2715142	NM_133334 // WHSC1 /// ENST00000398261 // WHSC1	WHSC1	chr4	0.0223954	-1.66928
2715443	NM_002938 // RNF4 /// ENST00000314289 // RNF4 /// AB000468 // RNF4	RNF4	chr4	0.0497325	-1.51516
2715880	NM_002111 // HTT /// ENST00000355072 // HTT /// AB016794 // HTT	HTT	chr4	0.0126631	-1.67491
2716118	NM_198229 // RGS12 /// NM_198227 // RGS12 /// ENST00000344733 // RGS12 /// ENST(	RGS12	chr4	0.0287235	-1.83802
2716186	NM_173660 // DOK7 /// NM_001164673 // DOK7 /// ENST00000340083 // DOK7 /// BC141	DOK7	chr4	0.0462801	-1.58568
2716490	NM_014392 // D4S234E /// NM_001040101 // D4S234E /// ENST00000382740 // D4S234E	D4S234E	chr4	0.0146598	-1.67578
2717032	NM_015274 // MAN2B2 /// ENST00000285599 // MAN2B2 /// AB023152 // MAN2B2	MAN2B2	chr4	0.010061	-1.51658
2724701	NM_004310 // RHOH /// ENST00000381799 // RHOH /// BC014261 // RHOH	RHOH	chr4	0.0479435	-1.69105
2727782	NM_024592 // SRD5A3 /// ENST00000264228 // SRD5A3	SRD5A3	chr4	0.012974	-1.59461
2731952	NM_001130016 // ART3 /// NM_001179 // ART3 /// NM_001130017 // ART3 /// ENST0000(	ART3	chr4	0.00133332	-2.84552
2735780	NM_007351 // MMRN1 /// ENST00000394980 // MMRN1 /// ENST00000264790 // MMRN1 //.	MMRN1	chr4	0.00351338	-1.88325
2739475	NM_001977 // ENPEP /// ENST00000265162 // ENPEP /// L12468 // ENPEF	ENPEP	chr4	0.012119	-1.63448
2741778	NM_001034194 // EXOSC9 /// NM_005033 // EXOSC9 /// ENST00000243498 // EXOSC9 //.	EXOSC9	chr4	0.0416441	-1.64966
2748121	NM_015271 // TRIM2 /// NM_001130067 // TRIM2 /// ENST00000437508 // TRIM2 /// EN	TRIM2	chr4	0.0326422	-1.64877
2750758	NM_012464 // TLL1 /// ENST00000061240 // TLL1 /// U91963 // TLL1	TLL1	chr4	0.0394341	-1.69221
2750805	NM_012464 // TLL1 /// ENST00000061240 // TLL1 /// U91963 // TLL1	TLL1	chr4	0.0376038	-1.88988
2751117	NM_001166108 // PALLD /// NM_016081 // PALLD /// NM_001166109 // PALLD /// ENST(	PALLD	chr4	0.00114406	-1.66196
2757561	NM_005663 // WHSC2 /// ENST00000382882 // WHSC2 /// AB044549 // WHSC2 /// AF1014	WHSC2	chr4	0.0297119	-1.6484
2757655	NM_181808 // POLN /// ENST00000382865 // POLN /// AY136549 // POLN /// ENST0000(	POLN	chr4	0.00337734	-1.85472
2757963	NM_024309 // TNIP2 /// ENST00000315423 // TNIP2 /// AF372839 // TNIP2	TNIP2	chr4	0.00958846	-1.87732
2758114	NM_003703 // NOP14 /// ENST00000314262 // NOP14 /// ENST00000416614 // NOP14 //.	NOP14	chr4	0.0426864	-1.63088
2759413	NM_025196 // GRPEL1 /// ENST00000264954 // GRPEL1 /// BC024242 // GRPEL1 /// ENS	GRPEL1	chr4	0.037952	-1.63813
2759861	NM_003501 // ACOX3 /// NM_001101667 // ACOX3 /// ENST00000356406 // ACOX3 /// EN	ACOX3	chr4	0.0486637	-1.60488
2761767	NM_012161 // FBXL5 /// NM_033535 // FBXL5 /// ENST00000422585 // FBXL5 /// ENST(	FBXL5	chr4	0.0187637	-1.51538
2766200	NM_030956 // TLR10 /// NM_001017388 // TLR10 /// ENST00000308973 // TLR10 /// EN	TLR10	chr4	0.00398859	-1.9921
2766898	NM_004307 // APBB2 /// NM_001166050 // APBB2 /// NM_173075 // APBB2 /// NM_0011€	APBB2	chr4	0.0120465	-1.51123
2768070	NM_000809 // GABRA4 /// ENST00000264318 // GABRA4 /// AK090780 // GABRA4	GABRA4	chr4	0.0226224	-1.93464
2769815	NM_002253 // KDR /// ENST00000263923 // KDR /// AF035121 // KDR	KDR	chr4	0.00830824	-1.6859
2770314	NM_001145460 // HOPX /// AF492678 // HOPX /// AF492679 // HOPX /// ENST000003812	HOPX	chr4	0.0412688	-1.59536
2773371	NM_002994 // CXCL5 /// ENST00000296027 // CXCL5 /// BC008376 // CXCL5	CXCL5	chr4	0.011289	-1.509
2776016	NM_006665 // HPSE /// NM_001098540 // HPSE /// NM_001166498 // HPSE /// ENST000(	HPSE	chr4	0.0225863	-1.74998
2776019	NM_006665 // HPSE /// NM_001098540 // HPSE /// NM_001166498 // HPSE /// ENST000(	HPSE	chr4	0.0209393	-1.68768
2776118	NM_139076 // FAM175A /// ENST00000321945 // FAM175A /// BC039573 // FAM175A	FAM175A	chr4	0.0121622	-1.71858
2776488	NM_014991 // WDFY3 /// NM_178583 // WDFY3 /// ENST00000295888 // WDFY3 /// ENST0	WDFY3	chr4	0.00198602	-1.99942
2779265	NM_000668 // ADH1B /// ENST00000305046 // ADH1B /// AF153821 // ADH1B /// ENST0(	ADH1B	chr4	0.0160428	-1.93148
2781201	NM_016269 // LEF1 /// NM_001130713 // LEF1 /// NM_001130714 // LEF1 /// ENST000(	LEF1	chr4	0.0304266	-1.62267
2781742	NM_000204 // CFI /// ENST00000394634 // CFI /// ENST00000394635 // CF	CFI	chr4	0.0101645	-1.89773
2781923	NM_000325 // PITX2 /// ENST00000306732 // PITX2	PITX2	chr4	0.00369084	-2.11604
2789013	NM_000901 // NR3C2 /// NM_001166104 // NR3C2 /// ENST00000358102 // NR3C2 /// M1	NR3C2	chr4	0.0225696	-1.629
2790551	NM_001142552 // DCHS2 /// NM_001142553 // DCHS2 /// ENST00000339452 // DCHS2 //.	DCHS2	chr4	0.0132363	-1.74539
2793956	NM_002129 // HMGB2 /// NM_001130688 // HMGB2 /// NM_001130689 // HMGB2 /// ENST(	HMGB2	chr4	0.00236751	-1.67567
2796969	NM_014476 // PDLIM3 /// ENST00000284770 // PDLIM3 /// BX647263 // PDLIM3 /// ENS	PDLIM3	chr4	0.031694	-1.63566
2797073	NM_001145671 // SORBS2 /// NM_001145673 // SORBS2 /// ENST00000319471 // SORBS2	SORBS2	chr4	2.05403E-05	-2.02377
2797474	NM_005245 // FAT1 /// ENST00000441802 // FAT1 /// ENST00000260147 // FAT1	FAT1	chr4	0.0231198	-1.71778
2798659	NM_020731 // AHRR /// BC151852 // AHRR /// ENST00000448949 // PDCD6 /// ENST000(	AHRR	chr5	0.0475006	-1.57705
2798689	NM_007277 // EXOC3 /// ENST00000315013 // EXOC3 /// AK131103 // EXOC3	EXOC3	chr5	0.00571599	-1.55276
2798970	NM_033120 // NKD2 /// ENST00000296849 // NKD2 /// BC012176 // NKD2 /// ENST0000(	NKD2	chr5	0.0141621	-1.5654

2806270	NM_194283 // DNAJC21 /// NM_001012339 // DNAJC21 /// ENST00000342382 // DNAJC21	DNAJC21	chr5	0.00506295	-2.79749
2807282	NM_152403 // EGFLAM /// NM_182798 // EGFLAM /// NM_182799 // EGFLAM /// NM_1828(	EGFLAM	chr5	0.0221805	-2.15216
2809632	NM_001102575 // SNX18 /// NM_052870 // SNX18 /// NM_001145427 // SNX18 /// ENST(	SNX18	chr5	0.0172922	-1.71985
2812576	NM_001077199 // SFRS12 /// NM_139168 // SFRS12 /// ENST00000380918 // SFRS12 //.	SFRS12	chr5	0.0160446	-1.55515
2812718	NM_198828 // MAST4 /// NM_001164664 // MAST4 /// ENST00000432212 // MAST4 /// EN	MAST4	chr5	0.0208729	-1.88175
2814644	NM_022132 // MCCC2 /// ENST00000340941 // MCCC2 /// AF301000 // MCCC2 /// ENST0(	MCCC2	chr5	0.0345572	-1.7595
2814875	NM_024754 // PTCD2 /// ENST00000380639 // PTCD2 /// ENST00000308077 // PTCD2 //.	PTCD2	chr5	0.0465917	-2.27983
2816063	BC050718 // POLK	POLK	chr5	0.00671731	-1.69381
2816408	NM_006633 // IQGAP2 /// ENST00000274364 // IQGAP2 /// AK291066 // IQGAP2 /// ENS	IQGAP2	chr5	0.0130517	-1.76575
2816460	NM_001992 // F2R /// ENST00000319211 // F2R /// BC002464 // F2R /// ENST0000039(	F2R	chr5	0.00155196	-2.23496
2818107	NM_032280 // ZCCHC9 /// NM_001131035 // ZCCHC9 /// NM_001131036 // ZCCHC9 /// EN	ZCCHC9	chr5	0.0038744	-1.86918
2819049	NM_002890 // RASA1 /// NM_022650 // RASA1 /// ENST00000274376 // RASA1 /// ENST(	RASA1	chr5	0.00314196	-1.55375
2825568	NM_005509 // DMXL1 /// ENST00000311085 // DMXL1 /// BC144537 // DMXL1	DMXL1	chr5	0.00763581	-1.50023
2825937	NM_016644 // PRR16 /// ENST00000379551 // PRR16 /// ENST00000330264 // PRR16 //.	PRR16	chr5	0.0269161	-1.50909
2827382	NM_032446 // MEGF10 /// ENST00000274473 // MEGF10 /// BC152478 // MEGF1(	MEGF10	chr5	0.0406898	-1.52007
2828471	NM_003687 // PDLIM4 /// NM_001131027 // PDLIM4 /// ENST00000253754 // PDLIM4 //.	PDLIM4	chr5	0.0103177	-1.66894
2829973	NM_000358 // TGFBI /// ENST00000442011 // TGFBI /// AK094581 // TGFBI /// ENST0(	TGFBI	chr5	0.0493086	-1.62816
2830841	ENST00000254901 // REEP2 /// AK223611 // REEP2 /// ENST00000378339 // REEP2	REEP2	chr5	0.0242758	-1.53101
2830955	NM_001903 // CTNNA1 /// ENST00000302763 // CTNNA1 /// D13866 // CTNNA1	CTNNA1	chr5	0.016538	-1.68488
2831024	NM_001903 // CTNNA1 /// ENST00000302763 // CTNNA1 /// D13866 // CTNNA1 /// ENST0	CTNNA1	chr5	0.020403	-1.59557
2832471	NR_001281 // PCDHB18 /// AF217743 // PCDHB18 /// ENST00000274705 // PCDHB18	PCDHB18	chr5	0.0201428	-1.73783
2835196	NM_001001669 // ARHGEF37 /// ENST00000333677 // FLJ41603	ARHGEF37	chr5	0.0478459	-1.54222
2836327	NM_000827 // GRIA1 /// NM_001114183 // GRIA1 /// ENST00000340592 // GRIA1 /// EN	GRIA1	chr5	0.00166022	-1.692
2840164	NM_004946 // DOCK2 /// ENST00000256935 // DOCK2 /// BC104900 // DOCK2	DOCK2	chr5	0.0320694	-1.55177
2840710	---	0	chr5	0.0189718	-1.66528
2840716	---	0	chr5	0.018453	-1.78717
2842920	NM_002011 // FGFR4 /// NM_022963 // FGFR4 /// NM_213647 // FGFR4 /// ENST000003(	FGFR4	chr5	0.0155921	-1.84236
2843048	NM_172349 // NSD1 /// NM_022455 // NSD1 /// ENST00000439151 // NSD1 /// ENST000(	NSD1	chr5	0.0270528	-1.58945
2843314	NM_007255 // B4GALT7 /// ENST00000029410 // B4GALT7 /// BC007317 // B4GALT7	B4GALT7	chr5	0.00867939	-1.61457
2844894	NM_197975 // BTNL3 /// ENST00000342868 // BTNL3 /// AY358385 // BTNL3 /// ENST0(	BTNL3	chr5	0.0204967	-1.62107
2847336	NM_017755 // NSUN2 /// ENST00000264670 // NSUN2 /// AB255451 // NSUN2	NSUN2	chr5	0.0312834	-1.50117
2852024	NM_013235 // RNASEN /// NM_001100412 // RNASEN /// ENST00000344624 // RNASEN //.	RNASEN	chr5	0.00179445	-1.51325
2855305	NM_005410 // SEPP1 /// NM_001085486 // SEPP1 /// NM_001093726 // SEPP1 /// BC015	SEPP1	chr5	0.0407114	-1.94538
2858241	AY245867 // PDE4D	PDE4D	chr5	0.0244384	-1.62764
2867287	NM_153216 // POU5F2 /// BC029532 // POU5F2	POU5F2	chr5	0.0190691	-1.79093
2869102	NM_180991 // SLCO4C1 /// ENST00000310954 // SLCO4C1 /// AY273896 // SLCO4C1	SLCO4C1	chr5	0.00304298	-1.53661
2869282	NM_017676 // GIN1 /// ENST00000399004 // GIN1 /// AK295796 // GIN1	GIN1	chr5	0.00776536	-1.54891
2870405	NM_014819 // PJA2 /// ENST00000361189 // PJA2 /// ENST00000361557 // PJA2 /// AK	PJA2	chr5	0.0435683	-1.57018
2871075	NM_022140 // EPB41L4A /// ENST00000261486 // EPB41L4A /// AB030240 // EPB41L4A .	EPB41L4A	chr5	0.0255683	-1.56167
2877179	NM_016603 // FAM13B /// NM_001101800 // FAM13B /// NM_001101801 // FAM13B /// EN	FAM13B	chr5	0.0174316	-1.58079
2877186	NM_016603 // FAM13B /// NM_001101800 // FAM13B /// NM_001101801 // FAM13B /// EN	FAM13B	chr5	0.00791591	-1.60893
2877476	NM_004730 // ETF1 /// ENST00000360541 // ETF1 /// BC014269 // ETF1	ETF1	chr5	0.0148432	-1.61858
2877480	NM_004730 // ETF1 /// ENST00000360541 // ETF1 /// BC014269 // ETF1	ETF1	chr5	0.0169718	-1.62628
2878079	NM_004883 // NRG2 /// NM_013981 // NRG2 /// NM_013982 // NRG2 /// NM_013983 // N	NRG2	chr5	0.00475393	-2.17795
2880077	NM_004576 // PPP2R2B /// NM_181674 // PPP2R2B /// NM_181675 // PPP2R2B /// NM_18	PPP2R2B	chr5	0.00360377	-1.55693
2880743	NM_024577 // SH3TC2 /// ENST00000323829 // SH3TC2 /// AY341075 // SH3TC2 /// ENS	SH3TC2	chr5	0.034347	-1.87828
2881580	NM_001135643 // DCTN4 /// NM_016221 // DCTN4 /// NM_001135644 // DCTN4 /// ENST(	DCTN4	chr5	0.00496078	-1.55134
2882587	NM_018691 // FAM114A2 /// ENST00000351797 // FAM114A2 /// AK303865 // FAM114A2	FAM114A2	chr5	0.0259235	-1.50751
2883388	NM_001100816 // MED7 /// NM_004270 // MED7 /// ENST00000286317 // MED7 /// ENST(	MED7	chr5	0.00531826	-1.58538
2884310	NM_002187 // IL12B /// ENST00000231228 // IL12B /// M65290 // IL12B	IL12B	chr5	0.000472267	-1.60397
2886604	NM_005565 // LCP2 /// ENST00000046794 // LCP2 /// AK292890 // LCP2	LCP2	chr5	0.0256614	-1.51014

2886607	NM_005565 // LCP2 /// ENST00000046794 // LCP2 /// AK292890 // LCP2	LCP2	chr5	0.00109039	-1.56424
2887467	NM_004387 // NKX2-5 /// NM_001166175 // NKX2-5 /// NM_001166176 // NKX2-5 /// EN	NKX2-5	chr5	0.0208944	-1.66293
2888509	NM_002115 // HK3 /// ENST00000292432 // HK3 /// AB208951 // HK3	HK3	chr5	0.000711612	-1.76406
2888689	NM_031300 // MXD3 /// NM_001142935 // MXD3 /// ENST00000439742 // MXD3 /// ENST0	MXD3	chr5	0.0326231	-1.55748
2889863	NM_000843 // GRM6 /// ENST00000231188 // GRM6	GRM6	chr5	0.0140757	-1.50701
2890703	NM_005110 // GFPT2 /// ENST00000253778 // GFPT2 /// AB016789 // GFPT2	GFPT2	chr5	0.00518641	-1.87153
2890861	NM_001114618 // MGAT1 /// NM_002406 // MGAT1 /// NM_001114617 // MGAT1 /// NM_00	MGAT1	chr5	0.000867506	-1.64635
2892753	NM_003913 // PRPF4B /// ENST00000337659 // PRPF4B /// AF283465 // PRPF4B	PRPF4B	chr6	0.00224238	-1.51482
2893563	NM_001168344 // RREB1 /// ENST00000379938 // RREB1	RREB1	chr6	0.0331911	-1.62594
2893821	NM_004415 // DSP /// NM_001008844 // DSP /// ENST00000379802 // DSP /// ENST0000	DSP	chr6	0.0241999	-1.78259
2894619	NM_145655 // GCNT2 /// ENST00000265012 // GCNT2	GCNT2	chr6	0.0149844	-1.54032
2897905	NM_003107 // SOX4 /// ENST00000244745 // SOX4 /// BC072668 // SOX4	SOX4	chr6	0.0277451	-1.53255
2897907	NM_003107 // SOX4 /// ENST00000244745 // SOX4 /// BC072668 // SOX4	SOX4	chr6	0.0263685	-1.73779
2901733	---	0	chr6	0.0154166	-1.64494
2901737	---	0	chr6	0.0279622	-1.57912
2901861	---	0	---	0.0448141	-1.83591
2902410	NM_000595 // LTA /// ENST00000418386 // LTA /// ENST00000412851 // LTA /// ENST0	LTA	chr6	0.0148103	-1.57145
2902476	NM_080686 // BAT2 /// ENST00000376007 // BAT2 /// ENST00000376033 // BAT2 /// BC	BAT2	chr6	0.039148	-1.54523
2902853	NM_001710 // CFB /// S67310 // CFB /// BC004143 // CFB /// BC007990 // CFB /// A	CFB	chr6	0.0461787	-1.55321
2902969	NM_007293 // C4A /// NM_001002029 // C4B /// ENST00000428956 // C4A /// BC151204	C4A	chr6	0.0186194	-1.83691
2903102	AF020544 // PPT2 /// AK292729 // PPT2 /// AK290473 // PPT2 /// ENST00000434075 /	PPT2	chr6	0.022515	-1.80981
2903829	NM_002224 // ITPR3 /// ENST00000374316 // ITPR3 /// U01062 // ITPR3	ITPR3	chr6	0.0435613	-1.65611
2904511	NM_152753 // SCUBE3 /// ENST00000274938 // SCUBE3 /// BC052263 // SCUBE3 /// ENS	SCUBE3	chr6	0.0398588	-1.60981
2905336	NM_173558 // FGD2 /// ENST00000373535 // FGD2 /// ENST00000274963 // FGD2 /// AK	FGD2	chr6	0.0107264	-1.63758
2905471	NM_003958 // RNF8 /// NM_183078 // RNF8 /// ENST00000373479 // RNF8 /// ENST0000	RNF8	chr6	0.0483565	-1.64327
2905791	NM_021943 // ZFAND3 /// ENST00000287218 // ZFAND3 /// ENST00000373391 // ZFAND3	ZFAND3	chr6	0.0375086	-1.56042
2906355	NM_015345 // DAAM2 /// ENST00000274867 // DAAM2 /// ENST00000398904 // DAAM2 //	DAAM2	chr6	0.0015743	-1.80422
2906960	NM_001134493 // TOMM6 /// ENST00000398884 // TOMM6 /// ENST00000335515 // PRICKL	TOMM6	chr6	0.0245434	-1.66157
2907547	NM_006245 // PPP2R5D /// NM_180976 // PPP2R5D /// ENST00000230402 // PPP2R5D //	PPP2R5D	chr6	0.0451008	-1.65386
2908038	NM_001146016 // TJAP1 /// NM_001146017 // TJAP1 /// NM_001146018 // TJAP1 /// NM	TJAP1	chr6	0.0488997	-1.80238
2908386	NM_007058 // CAPN11 /// ENST00000398776 // CAPN11 /// BC033733 // CAPN11	CAPN11	chr6	0.0302064	-1.65863
2908499	NM_007355 // HSP90AB1 /// ENST00000353801 // HSP90AB1 /// ENST00000371646 // HSP	HSP90AB1	chr6	0.00250507	-2.19655
2910697	NM_018214 // LRRC1 /// ENST00000370888 // LRRC1 /// AK021896 // LRRC1 /// ENST00	LRRC1	chr6	0.00636477	-1.70378
2912748	NM_001858 // COL19A1 /// ENST00000322773 // COL19A1 /// BC113362 // COL19A1 ///	COL19A1	chr6	0.0148232	-1.69103
2914114	NM_004999 // MYO6 /// ENST00000369977 // MYO6 /// BC146764 // MYO6 /// ENST0000	MYO6	chr6	0.0104773	-1.70183
2916718	NM_006813 // PNRC1 /// ENST00000336032 // PNRC1 /// U03105 // PNRC1 /// ENST0000	PNRC1	chr6	0.0458575	-1.77316
2919965	NM_016487 // C6orf203 /// NM_001142468 // C6orf203 /// NM_001142470 // C6orf203	C6orf203	chr6	0.0107966	-1.80979
2920727	NM_001083535 // C6orf182	C6orf182	chr6	0.00188686	-1.54534
2924606	NM_138571 // HINT3 /// ENST00000229633 // HINT3 /// BX647228 // HINT3	HINT3	chr6	0.0426734	-1.74031
2925325	NM_000426 // LAMA2 /// NM_001079823 // LAMA2 /// ENST00000421865 // LAMA2 /// EN	LAMA2	chr6	0.00204289	-1.50214
2925426	NM_000426 // LAMA2 /// NM_001079823 // LAMA2 /// ENST00000421865 // LAMA2 /// EN	LAMA2	chr6	0.0048353	-1.82087
2929904	NM_139244 // STXBP5 /// NM_001127715 // STXBP5 /// ENST00000321680 // STXBP5 //	STXBP5	chr6	0.0404826	-1.52636
2930636	NM_015093 // TAB2 /// ENST00000286332 // MAP3K7IP2 /// ENST00000367456 // MAP3K7	TAB2	chr6	0.0362185	-1.64639
2931098	NM_030949 // PPP1R14C /// ENST00000361131 // PPP1R14C /// AF407165 // PPP1R14C	PPP1R14C	chr6	0.0466245	-1.54828
2931411	NM_015440 // MTHFD1L /// ENST00000367321 // MTHFD1L /// AY374130 // MTHFD1L ///	MTHFD1L	chr6	0.0368988	-1.50137
2932226	NM_000914 // OPRM1 /// NM_001008504 // OPRM1 /// NM_001145282 // OPRM1 /// NM_00	OPRM1	chr6	0.0426541	-1.57438
2933450	NM_003898 // SYNJ2 /// ENST00000355585 // SYNJ2 /// AF318616 // SYNJ2 /// ENST00	SYNJ2	chr6	0.0131757	-1.64893
2934177	NM_014161 // MRPL18 /// ENST00000367034 // MRPL18 /// BC001623 // MRPL18	MRPL18	chr6	0.0254851	-1.56511
2934813	NM_005922 // MAP3K4 /// NM_006724 // MAP3K4 /// ENST00000348824 // MAP3K4 /// EN	MAP3K4	chr6	0.034535	-1.53231
2936578	NM_007045 // FGFR1OP /// NM_194429 // FGFR1OP /// ENST00000366847 // FGFR1OP //	FGFR1OP	chr6	0.0269034	-1.70357
2937013	NM_030615 // KIF25 /// NM_005355 // KIF25 /// ENST00000354419 // KIF25 /// ENST0	KIF25	chr6	0.030732	-1.63798

2939058	NM_004155 // SERPINB9 /// ENST00000380698 // SERPINB9 /// BC002538 // SERPINB9	SERPINB9	chr6	0.00666197	-1.51858
2940637	NM_001170692 // CAGE1 /// NM_001170693 // CAGE1 /// NM_205864 // CAGE1 /// ENST0	CAGE1	chr6	0.0176164	-1.70698
2943840	NM_005124 // NUP153 /// ENST00000262077 // NUP153 /// BC052965 // NUP153 /// ENS	NUP153	chr6	0.048327	-1.63622
2945769	NM_014722 // FAM65B /// NM_015864 // FAM65B /// ENST00000259698 // FAM65B /// EN	FAM65B	chr6	0.0244099	-1.67492
2946262	NM_005323 // HIST1H1T /// ENST00000338379 // HIST1H1T /// BC069517 // HIST1H1T	HIST1H1T	chr6	0.0229321	-1.65751
2948488	NM_003587 // DHX16 /// NM_001164239 // DHX16 /// ENST00000376442 // DHX16 /// EN	DHX16	chr6	0.00485542	-1.54294
2948502	NM_003587 // DHX16 /// NM_001164239 // DHX16 /// ENST00000376442 // DHX16 /// EN	DHX16	chr6	0.00911409	-1.50795
2948598	NM_005803 // FLOT1 /// ENST00000376389 // FLOT1 /// ENST00000383382 // FLOT1 //	FLOT1	chr6	0.00961816	-1.68388
2948801	NM_001264 // CDSN /// ENST00000445893 // CDSN /// ENST00000457875 // CDSN /// L2	CDSN	chr6	0.0158523	-2.186
2949097	---	0	chr6	0.0464159	-1.67875
2949374	NM_025258 // C6orf27 /// ENST00000375688 // C6orf27 /// ENST00000418870 // C6orf	C6orf27	chr6	0.0211441	-1.92598
2949391	NM_006295 // VARS /// ENST00000211402 // VARS /// ENST00000375663 // VARS /// EN	VARS	chr6	0.0241353	-1.51869
2949519	NM_025257 // SLC44A4 /// NM_001178044 // SLC44A4 /// NM_001178045 // SLC44A4 //	SLC44A4	chr6	0.0138407	-1.66027
2949654	NM_019105 // TNXB /// ENST00000375244 // TNXB /// ENST00000424713 // TNXB /// EN	TNXB	chr6	0.0331487	-1.54129
2949732	NM_004381 // ATF6B /// NM_001136153 // ATF6B /// ENST00000383156 // ATF6B /// EN	ATF6B	chr6	0.0428066	-1.71734
2949999	NM_019602 // BTNL2 /// ENST00000374993 // BTNL2 /// ENST00000423754 // BTNL2 //	BTNL2	chr6	0.00942679	-1.67289
2950425	NM_080680 // COL11A2 /// NM_080681 // COL11A2 /// NM_080679 // COL11A2 /// ENST0	COL11A2	chr6	0.0465787	-1.59386
2950544	NM_022553 // VPS52 /// ENST00000383210 // VPS52 /// ENST00000445902 // VPS52 //	VPS52	chr6	0.0113481	-1.51953
2950833	NM_054111 // IP6K3 /// NM_001142883 // IP6K3 /// ENST00000451316 // IP6K3 /// EN	IP6K3	chr6	0.0125782	-1.74972
2951193	NM_012391 // SPDEF /// ENST00000374037 // SPDEF /// AF071538 // SPDEF	SPDEF	chr6	0.0203307	-1.58467
2952793	BC022007 // C6orf64 /// AK001963 // C6orf64 /// ENST00000229903 // C6orf64	C6orf64	chr6	0.00762669	-1.5229
2953539	NM_024807 // TREML2 /// ENST00000373108 // TREML2 /// BC125078 // TREML2	TREML2	chr6	0.000655557	-1.70191
2953879	NM_001760 // CCND3 /// NM_001136125 // CCND3 /// ENST00000372991 // CCND3 /// M9	CCND3	chr6	0.00795177	-1.8326
2954387	NM_001168370 // CUL7 /// NM_014780 // CUL7 /// ENST00000265348 // CUL7 /// BC033	CUL7	chr6	0.00698139	-2.27433
2954424	NM_015950 // MRPL2 /// ENST00000388752 // MRPL2 /// ENST00000230413 // MRPL2 //	MRPL2	chr6	0.019097	-1.53541
2954514	NM_206922 // CRIP3 /// ENST00000372569 // CRIP3 /// AY555743 // CRIP3 /// ENST00	CRIP3	chr6	0.00929055	-1.63288
2954600	NM_023932 // DLK2 /// NM_206539 // DLK2 /// ENST00000357338 // DLK2 /// ENST000	DLK2	chr6	0.0340241	-1.91104
2956915	NM_138694 // PKHD1 /// ENST00000371117 // PKHD1 /// AY074797 // PKHD1	PKHD1	chr6	0.0183072	-1.79411
2958467	ENST00000446842 // DST /// ENST00000439203 // DST /// AF400227 // DST /// ENST00	DST	chr6	0.0345312	-1.60396
2960809	NM_030568 // KHDC1 /// BC022080 // KHDC1	KHDC1	chr6	0.0115199	-1.82262
2967325	NM_002726 // PREP /// ENST00000369110 // PREP /// BC030636 // PREP	PREP	chr6	0.0183525	-1.57301
2968333	---	0	chr6	0.0376783	-1.63023
2969832	NR_028338 // TRAF3IP2 /// NM_147686 // TRAF3IP2 /// NM_001164281 // TRAF3IP2 //	TRAF3IP2	chr6	0.0250873	-1.63217
2970991	NM_021648 // TSPYL4 /// ENST00000368611 // TSPYL4 /// ENST00000420283 // TSPYL4	TSPYL4	chr6	0.0242459	-1.52794
2973561	NM_001135648 // PTPRK /// NM_002844 // PTPRK /// ENST00000368226 // PTPRK /// EN	PTPRK	chr6	0.0189956	-1.61665
2975035	NM_005627 // SGK1 /// NM_001143676 // SGK1 /// NM_001143677 // SGK1 /// NM_00114	SGK1	chr6	0.009322	-1.55968
2977539	NM_032020 // FUCA2 /// ENST00000002165 // FUCA2 /// AY358551 // FUCA2 /// ENST00	FUCA2	chr6	0.0451497	-1.67726
2977972	NM_005670 // EPM2A /// ENST00000367519 // EPM2A /// AF284580 // EPM2A /// ENST00	EPM2A	chr6	0.0305674	-1.76823
2982105	NM_152133 // TAGAP /// NM_054114 // TAGAP /// NM_138810 // TAGAP /// ENST0000036	TAGAP	chr6	0.00100219	-1.55405
2984892	NM_003730 // RNASET2 /// ENST00000028008 // RNASET2 /// U85625 // RNASET2 /// BC	RNASET2	chr6	0.0218924	-1.57902
2985830	NM_003247 // THBS2 /// ENST00000366787 // THBS2 /// BC146676 // THBS2 /// ENST00	THBS2	chr6	0.0013119	-2.12969
2986370	NM_005618 // DLL1 /// ENST00000366756 // DLL1 /// AF196571 // DLL1	DLL1	chr6	0.0405181	-1.7637
2986963	NM_017781 // CYP2W1 /// ENST00000308919 // CYP2W1 /// ENST00000340150 // CYP2W1	CYP2W1	chr7	0.0181357	-1.57817
2986975	NM_017781 // CYP2W1 /// ENST00000308919 // CYP2W1 /// ENST00000340150 // CYP2W1	CYP2W1	chr7	0.0138238	-1.98657
2987424	NM_002452 // NUDT1 /// NM_198948 // NUDT1 /// NM_198949 // NUDT1 /// NM_198950 /	NUDT1	chr7	0.0499543	-1.51489
2987449	NM_003751 // EIF3B /// NM_001037283 // EIF3B /// ENST00000360876 // EIF3B /// EN	EIF3B	chr7	0.0321915	-1.528
2987667	NM_025250 // TTYH3 /// ENST00000258796 // TTYH3 /// BC152447 // TTYH3 /// ENST00	TTYH3	chr7	0.0403892	-1.61972
2988424	NR_023384 // RNF216L /// ENST00000360944 // RNF216L /// ENST00000407429 // RNF21	RNF216L	chr7	0.0257536	-1.55587
2988572	NM_015610 // WIPI2 /// NM_016003 // WIPI2 /// ENST00000288828 // WIPI2 /// ENST0	WIPI2	chr7	0.0262254	-1.74053
2988744	NM_003088 // FSCN1 /// ENST00000382361 // FSCN1 /// U03057 // FSCN1 /// U09873 /	FSCN1	chr7	0.0351669	-1.58523
2994848	NM_004067 // CHN2 /// ENST00000222792 // CHN2 /// BC112155 // CHN2 /// U28926 /	CHN2	chr7	0.0222493	-1.52699

2996248	NM_007270 // FKBP9 /// ENST00000242209 // FKBP9 /// AK300328 // FKBP9	FKBP9	chr7	0.0327533	-1.5466
2997963	NM_032016 // STARD3NL /// ENST00000009041 // STARD3NL /// ENST00000396013 // STA	STARD3NL	chr7	0.0139585	-1.54067
2999801	NM_001129 // AEBP1 /// ENST00000223357 // AEBP1 /// BC038588 // AEBP1 /// AK296(	AEBP1	chr7	0.0498861	-1.62485
3000222	NM_001029835 // CCM2 /// NM_031443 // CCM2 /// NM_001167934 // CCM2 /// NM_0011(	CCM2	chr7	0.021087	-1.59049
3002743	NM_005228 // EGFR /// NM_201282 // EGFR /// NM_201283 // EGFR /// NM_201284 // E	EGFR	chr7	0.0263014	-1.65192
3002753	NM_005228 // EGFR /// NM_201282 // EGFR /// NM_201284 // EGFR /// ENST00000275(	EGFR	chr7	0.0166559	-1.52259
3006134	NM_022906 // STAG3L4 /// ENST00000416602 // STAG3L4 /// ENST00000437742 // STAG3	STAG3L4	chr7	0.00209252	-1.57138
3007179	NM_022479 // WBSCR17 /// ENST00000333538 // WBSCR17 /// AF410457 // WBSCR17	WBSCR17	chr7	0.0225147	-1.8708
3008092	NM_000501 // ELN /// NM_001081752 // ELN /// NM_001081753 // ELN /// NM_0010817(	ELN	chr7	0.0167202	-1.50109
3008180	NM_032464 // LAT2 /// NM_032463 // LAT2 /// NM_014146 // LAT2 /// ENST00000344(	LAT2	chr7	0.0481454	-1.64253
3008241	NM_003388 // CLIP2 /// NM_032421 // CLIP2 /// ENST00000223398 // CLIP2 /// ENST(	CLIP2	chr7	0.0189287	-1.85244
3009243	NM_000941 // POR /// ENST00000265302 // POR /// AB051763 // POR /// AF258341 //	POR	chr7	0.0080907	-1.56851
3011347	NM_001143935 // CROT /// NM_021151 // CROT /// ENST00000331536 // CROT /// BC03(	CROT	chr7	0.0262593	-1.78193
3012821	NM_017667 // CCDC132 /// NM_024553 // CCDC132 /// ENST00000305866 // CCDC132 //.	CCDC132	chr7	0.00596088	-2.53303
3013108	NM_000089 // COL1A2 /// ENST00000297268 // COL1A2 /// BC054498 // COL1A2	COL1A2	chr7	0.0118359	-1.72357
3014820	NM_213603 // ZNF789 /// ENST00000331410 // ZNF789 /// BC089424 // ZNF789 /// ENS	ZNF789	chr7	0.029612	-1.60546
3015162	NM_003439 // ZKSCAN1 /// ENST00000324306 // ZKSCAN1 /// BX640646 // ZKSCAN1 ///	ZKSCAN1	chr7	0.01297	-1.68833
3015563	NM_019606 // MEPCE /// ENST00000310512 // MEPCE /// BC016396 // MEPCE /// ENST0(	MEPCE	chr7	0.0195051	-1.66063
3015802	NM_003386 // ZAN /// NM_173059 // ZAN /// ENST00000419263 // ZAN /// ENST0000042	ZAN	chr7	0.00862756	-1.76667
3016494	NM_181552 // CUX1 /// ENST00000292535 // CUX1 /// BC066592 // CUX1 /// ENST0000(	CUX1	chr7	0.011993	-1.80284
3016510	NM_001913 // CUX1 /// NM_181500 // CUX1 /// ENST00000437600 // CUX1 /// ENST000(	CUX1	chr7	0.0307514	-2.99357
3016743	NM_024653 // PRKRIP1 /// ENST00000397912 // PRKRIP1 /// AK098276 // PRKRIP1	PRKRIP1	chr7	0.0317441	-1.93073
3018380	NM_002736 // PRKAR2B /// ENST00000265717 // PRKAR2B /// BC075800 // PRKAR2B ///	PRKAR2B	chr7	0.00839644	-2.48921
3020193	NM_015641 // TES /// ENST00000358204 // TES /// ENST00000257721 // TES /// AF245	TES	chr7	0.0193854	-1.75869
3022424	NM_020369 // FSCN3 /// ENST00000265825 // FSCN3 /// AF281049 // FSCN3 /// ENST0(	FSCN3	chr7	0.00622197	-1.54745
3023115	NM_022742 // CCDC136 /// ENST00000297788 // CCDC136 /// BC150331 // CCDC136 ///	CCDC136	chr7	0.0255379	-1.50489
3023326	NM_178562 // TSPAN33 /// ENST00000289407 // TSPAN33 /// BC044244 // TSPAN33	TSPAN33	chr7	0.0336968	-1.63121
3023855	NM_001869 // CPA2 /// BT007403 // CPA2 /// BC007009 // CPA2 /// BC014571 // CPA2	CPA2	chr7	0.0421992	-1.61417
3024302	NM_013255 // MKLN1 /// ENST00000352689 // MKLN1 /// ENST00000446815 // MKLN1	MKLN1	chr7	0.0370292	-2.56066
3025357	NM_144648 // LRGUK /// ENST00000285928 // LRGUK /// BC104897 // LRGUK	LRGUK	chr7	0.0386774	-1.70209
3025811	NM_182489 // STRA8 /// ENST00000275764 // STRA8 /// AF513502 // STRA8	STRA8	chr7	0.0131335	-2.58319
3026622	NM_015905 // TRIM24 /// NM_003852 // TRIM24 /// ENST00000343526 // TRIM24 /// EN	TRIM24	chr7	0.0363954	-1.55129
3026843	NM_024926 // TTC26 /// NM_001144920 // TTC26 /// NM_001144923 // TTC26 /// ENST(	TTC26	chr7	0.04681	-1.7009
3027506	NM_052853 // ADCK2 /// ENST00000072869 // ADCK2 /// AY302592 // ADCK2	ADCK2	chr7	0.0253122	-1.67616
3028303	---	0	---	0.0342219	-1.57543
3029104	NM_000083 // CLCN1 /// ENST00000343257 // CLCN1 /// BC112156 // CLCN1	CLCN1	chr7	0.0482637	-1.81741
3031389	NM_013400 // REPIN1 /// NM_014374 // REPIN1 /// NM_001099696 // REPIN1 /// NM_0(	REPIN1	chr7	0.0304373	-1.68569
3031482	NM_175571 // GIMAP8 /// ENST00000307271 // GIMAP8 /// BC107037 // GIMAP8	GIMAP8	chr7	0.031132	-1.64096
3032491	NM_020445 // ACTR3B /// NM_001040135 // ACTR3B /// ENST00000377776 // ACTR3B //.	ACTR3B	chr7	0.0154387	-1.8831
3035313	NM_001080453 // INTS1 /// ENST00000404767 // INTS1 /// AY358482 // INTS1 /// ENS	INTS1	chr7	0.0094388	-1.59237
3035800	NM_152743 // C7orf27 /// BC015632 // C7orf27 /// ENST00000340611 // C7orf27 ///	C7orf27	chr7	0.000305869	-1.77414
3035802	NM_152743 // C7orf27 /// BC015632 // C7orf27 /// ENST00000340611 // C7orf27 ///	C7orf27	chr7	0.0429731	-1.71184
3038176	NM_004968 // ICA1 /// ENST00000406470 // ICA1 /// BC008640 // ICA1 /// U38260 /.	ICA1	chr7	0.0300903	-1.90822
3039455	NM_001004320 // TMEM195 /// ENST00000342526 // TMEM195 /// BC108676 // TMEM195 .	TMEM195	chr7	0.0345851	-1.7536
3039471	NM_001004320 // TMEM195 /// ENST00000342526 // TMEM195 /// BC108676 // TMEM195 .	TMEM195	chr7	0.0102977	-1.56531
3042010	NM_018947 // CYCS /// ENST00000305786 // CYCS /// ENST00000409409 // CYCS /// EN	CYCS	chr7	0.0095383	-2.13251
3046324	NM_014800 // ELMO1 /// ENST00000310758 // ELMO1 /// ENST00000442504 // ELMO1 //.	ELMO1	chr7	0.0192559	-1.57919
3046366	NM_014800 // ELMO1 /// ENST00000310758 // ELMO1 /// ENST00000442504 // ELMO1 //.	ELMO1	chr7	0.0270787	-1.55517
3047734	NM_000168 // GLI3 /// ENST00000395925 // GLI3 /// ENST00000395922 // GLI3 /// M5	GLI3	chr7	0.00502084	-1.75086
3048401	BC026306 // POLM /// ENST00000424258 // POLM /// ENST00000458246 // POLM /// ENS	POLM	chr7	0.0214815	-1.66942
3048427	NM_001127218 // POLD2 /// NM_006230 // POLD2 /// ENST00000395803 // POLD2 /// EN	POLD2	chr7	0.018304	-1.64826

3048449	NM_021223 // MYL7 /// ENST00000223364 // MYL7 /// BC027915 // MYL7 /// ENST0000(	MYL7	chr7	0.0401134	-1.81949
3049819	NM_138295 // PKD1L1 /// ENST00000289672 // PKD1L1 /// AB061683 // PKD1L1	PKD1L1	chr7	0.0174105	-2.41038
3050641	NM_015198 // COBL /// ENST00000265136 // COBL /// BC150263 // COBL /// ENST00000	COBL	chr7	0.0359202	-1.63226
3051885	NM_004577 // PSPH /// ENST00000275605 // PSPH /// ENST00000395471 // PSPH /// BC	PSPH	chr7	0.034437	-1.50554
3053830	NR_026873 // NCRNA00174	NCRNA00174	chr7	0.031009	-2.17412
3056155	ENST00000437521 // TBL2 /// ENST00000458466 // TBL2 /// ENST00000426966 // TBL2	TBL2	chr7	0.0206911	-1.64046
3058245	NM_012301 // MAGI2 /// ENST00000354212 // MAGI2 /// BC150277 // MAGI2 /// ENST0(	MAGI2	chr7	0.00757633	-1.65325
3058261	NM_012301 // MAGI2 /// ENST00000354212 // MAGI2 /// BC150277 // MAGI2 /// ENST0(	MAGI2	chr7	0.0447327	-1.81861
3061055	NM_194456 // KRIT1 /// NM_004912 // KRIT1 /// NM_194454 // KRIT1 /// NM_194455 /	KRIT1	chr7	0.037881	-1.72614
3061360	NM_001259 // CDK6 /// NM_001145306 // CDK6 /// ENST00000424848 // CDK6 /// ENST(	CDK6	chr7	0.0142599	-1.68359
3062811	NM_015395 // TECPR1 /// ENST00000447648 // TECPR1 /// BC053591 // TECPR1 /// ENS	TECPR1	chr7	0.0275629	-1.53775
3063117	NM_020429 // SMURF1 /// NM_181349 // SMURF1 /// ENST00000361125 // SMURF1 /// EN	SMURF1	chr7	0.000851397	-1.66906
3063774	AK302342 // C7orf43 /// BC015722 // C7orf43 /// ENST00000419037 // C7orf43 /// E	C7orf43	chr7	0.00394774	-1.80484
3063815	NM_152742 // GPC2 /// ENST00000292377 // GPC2 /// BC027972 // GPC2	GPC2	chr7	0.00018395	-1.58695
3064034	BC031966 // C7orf61 /// ENST00000426357 // C7orf61 /// ENST00000332375 // C7orf6	C7orf61	chr7	0.013206	-1.50713
3064101	AK074175 // LRCH4	LRCH4	chr7	0.0406162	-1.78956
3064107	NM_002319 // LRCH4 /// ENST00000310300 // LRCH4 /// ENST00000438884 // LRCH4 //.	LRCH4	chr7	0.0283991	-1.51994
3064250	NM_022574 // GIGYF1 /// ENST00000275732 // GIGYF1 /// AY176044 // GIGYF1	GIGYF1	chr7	0.00564042	-1.72964
3064560	NM_001084 // PLOD3 /// ENST00000223127 // PLOD3 /// BC011674 // PLOD3 /// ENST0(	PLOD3	chr7	0.0323101	-1.618
3064692	NM_138403 // MYL10 /// BC002778 // MYL10	MYL10	chr7	0.0116408	-1.57381
3066781	NM_006754 // SYPL1 /// NM_182715 // SYPL1 /// ENST00000011473 // SYPL1 /// ENST(	SYPL1	chr7	0.00876646	-1.72049
3069373	NM_003391 // WNT2 /// NR_024047 // WNT2 /// ENST00000265441 // WNT2 /// BC078170	WNT2	chr7	0.00373911	-1.67728
3069518	NM_033427 // CTTNBP2 /// ENST00000160373 // CTTNBP2 /// BC106000 // CTTNBP2 ///	CTTNBP2	chr7	0.00381223	-2.04154
3070892	NM_005302 // GPR37 /// ENST00000303921 // GPR37 /// BC040007 // GPR37	GPR37	chr7	0.0130165	-1.65829
3071633	ENST00000424840 // MGC27345	MGC27345	chr7	0.0225656	-1.50258
3074047	NM_032826 // SLC35B4 /// ENST00000378509 // SLC35B4 /// AB052892 // SLC35B4 ///	SLC35B4	chr7	0.00573297	-1.86375
3074397	NM_013316 // CNOT4 /// NM_001008225 // CNOT4 /// ENST00000428680 // CNOT4 /// EN	CNOT4	chr7	0.00757468	-1.53901
3075589	NM_020119 // ZC3HAV1 /// NM_024625 // ZC3HAV1 /// ENST00000242351 // ZC3HAV1 //.	ZC3HAV1	chr7	0.0136051	-1.65069
3077250	NR_002140 // OR6W1P /// AF286696 // OR6W1P /// ENST00000378175 // OR6W1P /// ENS	OR6W1P	chr7	0.0084994	-1.8289
3078393	NM_004456 // EZH2 /// NM_152998 // EZH2 /// ENST00000320356 // EZH2 /// ENST000(	EZH2	chr7	0.00207092	-1.62084
3078436	NM_004911 // PDIA4 /// ENST00000286091 // PDIA4 /// BC011754 // PDIA4	PDIA4	chr7	0.0217244	-1.56213
3078580	NM_001163474 // ZNF746 /// NM_152557 // ZNF746	ZNF746	chr7	0.0141473	-1.74959
3079280	NM_173681 // ATG9B /// ENST00000377974 // ATG9B /// AY515311 // ATG9B /// ENST0(	ATG9B	chr7	0.0440965	-2.12454
3079496	NM_007189 // ABCF2 /// NM_005692 // ABCF2 /// ENST00000287844 // ABCF2 /// ENST(	ABCF2	chr7	0.00126132	-1.70669
3082801	NM_004745 // DLGAP2 /// ENST00000421627 // DLGAP2 /// ENST00000357934 // DLGAP2	DLGAP2	chr8	0.0248216	-1.54735
3082900	NM_014629 // ARHGEF10 /// ENST00000349830 // ARHGEF10 /// ENST00000398564 // ARH	ARHGEF10	chr8	0.00253895	-1.66553
3085079	NM_153332 // ERI1 /// ENST00000250263 // ERI1 /// AY310909 // ERI1	ERI1	chr8	0.0260816	-1.50424
3088052	AY176665 // NSAP11 /// ENST00000334070 // NSAP11	NSAP11	chr8	0.0121458	-1.79576
3089093	NM_003867 // FGF17 /// ENST00000359441 // FGF17	FGF17	chr8	0.029409	-2.01784
3089169	NM_022749 // FAM160B2 /// ENST00000289921 // FAM160B2 /// ENST00000450006 // FAM	FAM160B2	chr8	0.0333605	-1.54277
3089217	NM_001199 // BMP1 /// NM_006129 // BMP1 /// NR_033403 // BMP1 /// NR_033404 // E	BMP1	chr8	0.00789112	-2.21043
3089565	NM_021630 // PDLIM2 /// NM_198042 // PDLIM2 /// ENST00000308354 // PDLIM2 /// EN	PDLIM2	chr8	0.0122296	-2.01836
3089616	NM_021174 // KIAA1967 /// NM_199205 // KIAA1967 /// ENST00000308511 // KIAA1967	KIAA1967	chr8	0.0361899	-1.59886
3089635	NM_021174 // KIAA1967 /// NM_199205 // KIAA1967 /// ENST00000308511 // KIAA1967	KIAA1967	chr8	0.0323247	-1.56872
3089767	NM_001160036 // RHOBTB2 /// NM_001160037 // RHOBTB2 /// NM_015178 // RHOBTB2 //.	RHOBTB2	chr8	0.0491784	-1.58793
3089870	NM_152272 // CHMP7 /// ENST00000397677 // CHMP7 /// ENST00000313219 // CHMP7 //.	CHMP7	chr8	0.00824502	-1.82523
3091134	NM_001386 // DPYSL2 /// ENST00000311151 // DPYSL2 /// BC056408 // DPYSL2 /// ENS	DPYSL2	chr8	0.0143473	-1.53389
3091638	NM_018091 // ELP3 /// ENST00000256398 // ELP3 /// BC001240 // ELP3 /// ENST0000(	ELP3	chr8	0.011378	-1.85931
3091707	NM_006228 // PNOC /// ENST00000301908 // PNOC /// U48263 // PNOC	PNOC	chr8	0.0226516	-1.63517
3094388	NM_032777 // GPR124 /// ENST00000412232 // GPR124 /// AF378755 // GPR124 /// ENS	GPR124	chr8	0.0339622	-1.52919
3095315	NM_020130 // C8orf4 /// ENST00000315792 // C8orf4 /// BC021672 // C8orf4	C8orf4	chr8	0.0166039	-1.7

3096548	NM_032237 // SGK196 /// ENST00000331373 // SGK196	SGK196	chr8	0.00722615	-1.60992
3101634	NM_144650 // ADHFE1 /// ENST00000396623 // ADHFE1 /// AY033237 // ADHFE1 /// BC0	ADHFE1	chr8	0.0353285	-2.04037
3101646	NM_144650 // ADHFE1 /// ENST00000396623 // ADHFE1 /// AY033237 // ADHFE1 /// BC0	ADHFE1	chr8	0.0441027	-1.58474
3108118	NM_014754 // PTDSS1 /// ENST00000337004 // PTDSS1 /// BC004390 // PTDSS1 /// ENS	PTDSS1	chr8	0.00601202	-1.56407
3108907	NM_152564 // VPS13B /// NM_015243 // VPS13B /// NM_181661 // VPS13B /// NM_01789	VPS13B	chr8	0.00856943	-2.13266
3109724	NM_024915 // GRHL2 /// ENST00000251808 // GRHL2 /// BC069633 // GRHL2 /// ENST00	GRHL2	chr8	0.00945299	-1.85349
3110569	NM_001100117 // RIMS2 /// NM_014677 // RIMS2 /// ENST00000408894 // RIMS2 /// BC	RIMS2	chr8	0.0389741	-1.50307
3116595	NM_016018 // PHF20L1 /// ENST00000395390 // PHF20L1 /// ENST00000395386 // PHF20	PHF20L1	chr8	0.0201662	-1.56225
3116651	NM_003235 // TG /// ENST00000220616 // TG /// U93033 // TG /// ENST00000377869 /	TG	chr8	0.0083606	-1.68953
3118711	NM_014957 // DENND3 /// ENST00000262585 // DENND3 /// BC133023 // DENND3 /// BC1	DENND3	chr8	0.00834267	-1.55187
3119528	NM_030895 // ZNF696 /// ENST00000330143 // ZNF696 /// BC126317 // ZNF696	ZNF696	chr8	0.00389574	-1.92377
3120499	NM_174922 // ADCK5 /// ENST00000308860 // ADCK5 /// BC101659 // ADCK5	ADCK5	chr8	0.00147115	-1.6357
3127761	NM_003840 // TNFRSF10D /// ENST00000312584 // TNFRSF10D /// AY358285 // TNFRSF10	TNFRSF10D	chr8	0.0359487	-1.55362
3127851	NM_002318 // LOXL2 /// U89942 // LOXL2 /// ENST00000404624 // LOXL2	LOXL2	chr8	0.0116242	-1.57966
3128288	NM_006158 // NEFL /// ENST00000221169 // NEFL /// ENST00000380781 // NEFL /// BC	NEFL	chr8	0.0432462	-1.78799
3129086	NM_001831 // CLU /// NM_203339 // CLU /// NM_001171138 // CLU /// ENST0000040514	CLU	chr8	0.0145827	-1.57837
3131909	NM_001102559 // PPAPDC1B /// NM_032483 // PPAPDC1B /// NM_001102560 // PPAPDC1B	PPAPDC1B	chr8	0.0111113	-1.93427
3132967	NM_020476 // ANK1 /// NM_020477 // ANK1 /// NM_000037 // ANK1 /// NM_020475 // A	ANK1	chr8	0.030793	-1.7657
3136933	NM_014729 // TOX /// ENST00000361421 // TOX /// ENST00000456290 // TOX /// AB018	TOX	chr8	0.00682786	-1.8937
3144255	NM_018710 // TMEM55A /// ENST00000285419 // TMEM55A /// BC033892 // TMEM55A	TMEM55A	chr8	0.0109773	-1.6799
3145973	---	0	chr8	0.0224695	-1.56944
3146050	NM_024759 // NIPAL2 /// ENST00000341166 // NIPAL2 /// BC132687 // NIPAL2 /// ENS	NIPAL2	chr8	0.0263267	-1.50873
3150153	NM_000127 // EXT1 /// ENST00000378204 // EXT1 /// BC001174 // EXT1 /// ENST0000C	EXT1	chr8	0.0167654	-1.5776
3151661	NM_058229 // FBXO32 /// ENST00000287396 // FBXO32 /// AY059629 // FBXO32 /// ENS	FBXO32	chr8	0.0353747	-1.79144
3152222	NM_014846 // KIAA0196 /// ENST00000318410 // KIAA0196 /// BC106015 // KIAA0196 /	KIAA0196	chr8	0.00256523	-1.69519
3157734	NM_198488 // FAM83H /// ENST00000395103 // FAM83H /// ENST00000388913 // FAM83H	FAM83H	chr8	0.0160248	-1.65708
3158605	NM_017767 // SLC39A4 /// ENST00000276833 // SLC39A4	SLC39A4	chr8	0.00870661	-1.55632
3158618	AK091979 // VPS28	VPS28	chr8	0.0489053	-1.53688
3158750	NM_003923 // FOXH1 /// ENST00000377317 // FOXH1 /// AF076292 // FOXH1 /// ENST00	FOXH1	chr8	0.00739629	-1.57342
3160006	NM_003070 // SMARCA2 /// NM_139045 // SMARCA2 /// ENST00000349721 // SMARCA2 //	SMARCA2	chr9	0.0490404	-1.70788
3164183	NM_006570 // RRAGA /// ENST00000380527 // RRAGA /// BC009990 // RRAGA	RRAGA	chr9	0.0402995	-2.47286
3167758	NM_006377 // UNC13B /// ENST00000378495 // UNC13B /// AF020202 // UNC13B /// ENS	UNC13B	chr9	0.00640042	-1.65885
3168146	NM_001080496 // RGP1 /// ENST00000456972 // RGP1 /// ENST00000378078 // RGP1 //	RGP1	chr9	0.0384608	-1.8215
3173893	NM_004817 // TJP2 /// NM_201629 // TJP2 /// NM_001170416 // TJP2 /// NM_00117041	TJP2	chr9	0.00638178	-1.60228
3178151	NM_001912 // CTSL1 /// ENST00000343150 // CTSL1 /// AK055599 // CTSL1	CTSL1	chr9	0.00134754	-1.93405
3178429	NM_006717 // SPIN1 /// ENST00000375859 // SPIN1 /// AK092017 // SPIN1	SPIN1	chr9	0.00775862	-1.5592
3181219	NM_014290 // TDRD7 /// ENST00000355295 // TDRD7 /// BC028694 // TDRD7 /// ENST00	TDRD7	chr9	0.00505258	-1.53238
3181288	NM_003275 // TMOD1 /// NM_001166116 // TMOD1 /// ENST00000259365 // TMOD1 /// EN	TMOD1	chr9	0.0145506	-1.61068
3181466	NM_018946 // NANS /// ENST00000210444 // NANS /// ENST00000424455 // NANS	NANS	chr9	0.0128228	-1.66161
3181693	NM_001855 // COL15A1 /// ENST00000375001 // COL15A1 /// L25286 // COL15A1	COL15A1	chr9	0.0359879	-1.70625
3181730	NM_004612 // TGFBR1 /// NM_001130916 // TGFBR1 /// ENST00000374994 // TGFBR1 //	TGFBR1	chr9	0.00125571	-1.74065
3184902	NM_133464 // ZNF483 /// ENST00000309235 // ZNF483 /// AK291267 // ZNF483 /// ENS	ZNF483	chr9	0.00964003	-1.53398
3188998	NM_030978 // ARPC5L /// ENST00000353214 // ARPC5L /// ENST00000259477 // ARPC5L	ARPC5L	chr9	0.0182302	-2.28225
3189096	NM_005833 // RABEPK /// NM_001174152 // RABEPK /// NM_001174153 // RABEPK /// EN	RABEPK	chr9	0.045638	-1.58337
3189724	NM_032293 // GARNL3 /// ENST00000373387 // GARNL3 /// AK296246 // GARNL3 /// AK2	GARNL3	chr9	0.00303942	-1.76703
3190202	NM_005564 // LCN2 /// ENST00000373017 // LCN2 /// ENST00000277480 // LCN2 /// AK	LCN2	chr9	0.0199844	-1.61881
3190486	NM_002540 // ODF2 /// NM_153437 // ODF2 /// ENST00000393527 // ODF2 /// ENST0000	ODF2	chr9	0.0354694	-1.51442
3190521	NM_001003722 // GLE1 /// NM_001499 // GLE1 /// ENST00000309971 // GLE1 /// ENST0	GLE1	chr9	0.00746005	-2.47657
3190645	NM_001130438 // SPTAN1 /// NM_003127 // SPTAN1 /// ENST00000372731 // SPTAN1 //	SPTAN1	chr9	0.000455448	-1.9208
3190915	AK301241 // FAM73B /// AK303036 // FAM73B /// BC009114 // FAM73B /// ENST0000041	FAM73B	chr9	0.0454346	-1.66768
3191166	NM_014506 // TOR1B /// ENST00000259339 // TOR1B /// BC015578 // TOR1B /// ENST00	TOR1B	chr9	0.00155538	-1.52893

3191185	NM_006676 // USP20 /// NM_001008563 // USP20 /// NM_001110303 // USP20 /// ENST(	USP20	chr9	0.0311823	-2.42163
3191999	NM_005085 // NUP214 /// ENST00000359428 // NUP214 /// AK302676 // NUP214 /// AK3	NUP214	chr9	0.0242497	-1.73415
3192188	NM_007171 // POMT1 /// NM_001077365 // POMT1 /// NM_001077366 // POMT1 /// NM_0(	POMT1	chr9	0.0268321	-1.73004
3192510	NM_020064 // BARHL1 /// ENST00000263610 // BARHL1 /// BC136956 // BARHL1	BARHL1	chr9	0.0350215	-1.62373
3193572	NM_000093 // COL5A1 /// ENST00000371817 // COL5A1 /// AB371583 // COL5A1 /// ENS	COL5A1	chr9	0.0199491	-1.93374
3193727	NM_014279 // OLFM1 /// NM_006334 // OLFM1 /// ENST00000252854 // OLFM1 /// ENST(	OLFM1	chr9	0.0160182	-1.85192
3193730	BC000189 // OLFM1 /// ENST00000371799 // OLFM1	OLFM1	chr9	0.0290092	-1.86046
3194558	NM_016215 // EGFL7 /// NM_201446 // EGFL7 /// ENST00000371699 // EGFL7 /// ENST(	EGFL7	chr9	0.0126804	-1.91466
3194769	NM_024718 // C9orf86 /// NM_001173988 // C9orf86 /// ENST00000311502 // C9orf86	C9orf86	chr9	0.0387596	-1.76111
3195151	NM_207309 // UAP1L1 /// ENST00000409858 // UAP1L1 /// ENST00000360271 // UAP1L1	UAP1L1	chr9	0.00565832	-1.51149
3195652	NM_000718 // CACNA1B /// ENST00000371372 // CACNA1B /// ENST00000277549 // CACNA	CACNA1B	chr9	0.0281667	-1.72596
3199824	NM_021144 // PSIP1 /// NM_033222 // PSIP1 /// NM_001128217 // PSIP1 /// ENST000(	PSIP1	chr9	0.0396181	-1.69277
3203593	NM_022917 // NOL6 /// NM_139235 // NOL6 /// ENST00000297990 // NOL6 /// ENST000(	NOL6	chr9	0.0144568	-1.84132
3204070	NM_198573 // ENHO /// ENST00000399775 // ENHO /// ENST00000303992 // ENHO	ENHO	chr9	0.024849	-1.56128
3204186	NM_148178 // C9orf23 /// NM_148179 // C9orf23 /// ENST00000297613 // C9orf23 //,	C9orf23	chr9	0.0355108	-1.77823
3204259	NM_005866 // SIGMAR1 /// NM_147157 // SIGMAR1 /// ENST00000277010 // SIGMAR1 //,	SIGMAR1	chr9	0.0291428	-1.64136
3204650	NM_001782 // CD72 /// ENST00000396757 // CD72 /// ENST00000259633 // CD72 /// EN	CD72	chr9	0.040298	-1.76267
3204700	NM_032818 // C9orf100 /// ENST00000378387 // C9orf100 /// ENST00000378395 // C9c	C9orf100	chr9	0.0160695	-1.60631
3205848	AK093059 // ANKRD18A /// BC131497 // ANKRD18A	ANKRD18A	chr9	0.00994958	-1.80455
3210532	NM_015225 // PRUNE2 /// ENST00000376718 // PRUNE2 /// AB050197 // PRUNE2 /// AY4	PRUNE2	chr9	0.0297954	-1.53974
3213562	NM_178432 // CDK20 /// NM_012119 // CDK20 /// NM_001039803 // CDK20 /// NM_0011	CDK20	chr9	0.0397035	-1.50642
3213865	NM_016848 // SHC3 /// ENST00000375835 // SHC3 /// BC026314 // SHC3 /// ENST0000(	SHC3	chr9	0.00133075	-1.63067
3214421	NM_001698 // AUH /// ENST00000375731 // AUH /// BC020722 // AUH /// ENST0000030	AUH	chr9	0.0320911	-1.55344
3214779	NR_024020 // NOL8 /// NM_017948 // NOL8 /// ENST00000442668 // NOL8 /// ENST000(	NOL8	chr9	0.0280455	-1.68577
3215569	NM_003837 // FBP2 /// ENST00000375337 // FBP2	FBP2	chr9	0.0261397	-1.57747
3216233	NM_000197 // HSD17B3 /// ENST00000375263 // HSD17B3 /// BC034281 // HSD17B3 ///	HSD17B3	chr9	0.015815	-1.78964
3217218	NM_018421 // TBC1D2 /// ENST00000375066 // TBC1D2 /// ENST00000375064 // TBC1D2	TBC1D2	chr9	0.0164977	-1.54554
3219903	NM_002829 // PTPN3 /// NM_001145368 // PTPN3 /// NM_001145369 // PTPN3 /// NM_0(	PTPN3	chr9	0.0494991	-1.60776
3221576	NM_145051 // RNF183 /// AK055759 // RNF183	RNF183	chr9	0.0496462	-1.68711
3221705	AK056275 // FLJ31713	FLJ31713	chr9	0.0275329	-1.50636
3221933	NM_030767 // AKNA /// ENST00000307564 // AKNA /// ENST00000374088 // AKNA /// BC	AKNA	chr9	0.00881314	-1.76262
3222177	NM_002160 // TNC /// ENST00000350763 // TNC /// M55618 // TNC /// ENST000003400	TNC	chr9	0.0073262	-1.62625
3222215	NM_002160 // TNC /// ENST00000350763 // TNC /// M55618 // TNC /// ENST0000034103	TNC	chr9	0.0395303	-1.65092
3223543	NM_018249 // CDK5RAP2 /// NM_001011649 // CDK5RAP2 /// ENST00000349780 // CDK5RA	CDK5RAP2	chr9	0.00211848	-2.98263
3226119	NM_001114753 // ENG /// NM_000118 // ENG /// ENST00000373203 // ENG /// ENST000(	ENG	chr9	0.0277528	-1.60735
3226356	NM_025072 // PTGES2 /// ENST00000338961 // PTGES2 /// AK057049 // PTGES2 /// ENS	PTGES2	chr9	0.0386357	-1.56721
3228990	NM_001134398 // VAV2 /// NM_003371 // VAV2 /// ENST00000371850 // VAV2 /// ENST(	VAV2	chr9	0.00858828	-1.54381
3229548	NM_015447 // CAMSAP1 /// ENST00000389532 // CAMSAP1 /// BC130580 // CAMSAP1 ///	CAMSAP1	chr9	0.0417992	-1.68489
3230202	NM_017617 // NOTCH1 /// ENST00000277541 // NOTCH1 /// AF308602 // NOTCH1	NOTCH1	chr9	0.0067702	-2.3553
3230621	NM_001606 // ABCA2 /// NM_212533 // ABCA2 /// ENST00000341511 // ABCA2 /// ENST(	ABCA2	chr9	0.0318057	-1.55001
3231390	NM_006624 // ZMYND11 /// NM_212479 // ZMYND11 /// NM_001161482 // ZMYND11 /// EN	ZMYND11	chr10	0.0151079	-1.52942
3232361	NM_002627 // PFKP /// ENST00000381125 // PFKP /// ENST00000381188 // PFKP /// EN	PFKP	chr10	0.00287997	-1.71126
3236398	NM_016299 // HSPA14 /// ENST00000378372 // HSPA14 /// AK292323 // HSPA14 /// ENS	HSPA14	chr10	0.00192814	-1.74906
3237091	NM_003473 // STAM /// ENST00000377524 // STAM /// BC030586 // STAM /// ENST0000(	STAM	chr10	0.0201296	-1.57919
3237451	NM_201590 // CACNB2 /// ENST00000377329 // CACNB2	CACNB2	chr10	0.00691289	-1.71741
3237886	NM_032812 // PLXDC2 /// ENST00000377252 // PLXDC2 /// AY358486 // PLXDC2 /// ENS	PLXDC2	chr10	0.00636972	-1.57172
3243890	NM_020975 // RET /// ENST00000340058 // RET /// ENST00000355710 // RET	RET	chr10	0.0121659	-1.7483
3244116	NM_145312 // ZNF485 /// ENST00000361807 // ZNF485 /// ENST00000374435 // ZNF485	ZNF485	chr10	0.0233761	-1.60204
3244659	NM_000698 // ALOX5 /// ENST00000374391 // ALOX5 /// J03571 // ALOX5	ALOX5	chr10	0.00815976	-1.69909
3245816	NM_020945 // WDFY4 /// ENST00000325239 // WDFY4 /// ENST00000413659 // WDFY4 //,	WDFY4	chr10	0.00131114	-2.52241
3248679	NM_014951 // ZNF365 /// ENST00000395254 // ZNF365 /// BC060817 // ZNF365 /// ENS	ZNF365	chr10	0.0395972	-1.64175

3250160	NM_002727 // SRGN /// ENST00000242465 // SRGN /// BC015516 // SRGN	SRGN	chr10	0.00408925	-1.53443
3250302	NM_033496 // HK1 /// ENST00000436817 // HK1 /// ENST00000298649 // HK1	HK1	chr10	0.0128189	-1.70354
3250442	NM_145306 // C10orf35 /// ENST00000373279 // C10orf35 /// BC013587 // C10orf35 /	C10orf35	chr10	0.0197634	-2.01764
3250830	NM_139155 // ADAMTS14 /// NM_080722 // ADAMTS14 /// ENST00000373208 // ADAMTS14	ADAMTS14	chr10	0.0227893	-1.63735
3251568	NM_152635 // OIT3 /// ENST00000334011 // OIT3 /// AY358339 // OIT3 /// ENST0000C	OIT3	chr10	0.00178712	-1.99702
3254129	NR_002724 // MBL1P /// BC071736 // LOC387693	MBL1P	---	0.0358819	-2.04448
3255357	NM_001171971 // CDHR1 /// BC038799 // CDHR1 /// ENST00000332904 // PCDH21	CDHR1	chr10	0.0163333	-1.65621
3256682	NR_028492 // CFLP1 /// BC031631 // CFLP1	CFLP1	chr10	0.0125601	-1.8177
3260341	NM_145285 // NKX2-3 /// ENST00000344586 // NKX2-3 /// AK300856 // NKX2-3	NKX2-3	chr10	0.00735227	-1.54898
3260398	NM_020354 // ENTPD7 /// ENST00000370489 // ENTPD7 /// BC122857 // ENTPD7	ENTPD7	chr10	0.000217804	-1.56908
3260971	NM_032429 // LZTS2 /// ENST00000370223 // LZTS2 /// ENST00000370220 // LZTS2 //.	LZTS2	chr10	0.0111199	-2.06102
3261166	NM_033637 // BTRC /// NM_003939 // BTRC /// ENST00000408038 // BTRC /// ENST0000	BTRC	chr10	0.0477867	-1.61429
3262545	NM_183239 // GSTO2 /// ENST00000369708 // GSTO2 /// ENST00000338595 // GSTO2 //.	GSTO2	chr10	0.0295487	-1.52891
3264069	NM_000681 // ADRA2A /// AF284095 // ADRA2A /// ENST00000449894 // ADRA2A /// ENS	ADRA2A	chr10	0.0030655	-1.81702
3264627	NM_001146274 // TCF7L2 /// NM_030756 // TCF7L2 /// NM_001146283 // TCF7L2 /// NM	TCF7L2	chr10	0.0353002	-1.75844
3265967	NM_005396 // PNLIPRP2 /// AK291492 // PNLIPRP2	PNLIPRP2	chr10	0.0382037	-1.50573
3267105	NM_005308 // GRK5 /// ENST00000392870 // GRK5 /// L15388 // GRK5	GRK5	chr10	0.0132482	-1.50573
3267458	NM_007190 // SEC23IP /// ENST00000369075 // SEC23IP /// BX649119 // SEC23IP	SEC23IP	chr10	0.0291234	-1.51002
3268558	NM_153336 // PSTK /// ENST00000368887 // PSTK /// ENST00000405485 // PSTK /// EN	PSTK	chr10	0.0100651	-2.49907
3268564	NM_153336 // PSTK /// ENST00000368887 // PSTK /// ENST00000405485 // PSTK /// EN	PSTK	chr10	0.0169475	-1.75455
3272601	NM_152643 // KNDC1 /// ENST00000304613 // KNDC1 /// AB000781 // KNDC1 /// ENST0C	KNDC1	chr10	0.0241942	-1.84703
3272709	NM_014468 // VENTX /// ENST00000325980 // VENTX /// AF068006 // VENTX	VENTX	chr10	0.00594906	-1.51491
3272751	NM_145806 // ZNF511 /// ENST00000361518 // ZNF511 /// AK091711 // ZNF511	ZNF511	chr10	0.00498152	-1.98709
3272805	NM_152911 // PAOX /// NM_207127 // PAOX /// NM_207128 // PAOX /// ENST00000278O6	PAOX	chr10	0.013925	-1.76867
3273344	NM_014974 // DIP2C /// ENST00000280886 // DIP2C /// BC063313 // DIP2C /// ENST0C	DIP2C	chr10	0.0110285	-2.35308
3275294	---	0	chr10	0.00710962	-1.88101
3275466	---	0	chr10	0.00979919	-1.5545
3276569	NR_024255 // FLJ45983 /// NR_024256 // FLJ45983 /// BC036297 // FLJ45983	FLJ45983	chr10	0.0359652	-1.7135
3278342	NM_152751 // BEND7 /// NM_001100912 // BEND7 /// ENST00000341083 // BEND7 /// EN	BEND7	chr10	0.0340935	-1.9592
3279657	ENST00000377921 // RSU1 /// BC008691 // RSU1	RSU1	chr10	0.0154188	-1.64904
3282223	NM_139312 // YME1L1 /// NM_014263 // YME1L1 /// ENST00000326799 // YME1L1 /// EN	YME1L1	chr10	0.0350008	-1.57523
3284125	NM_025209 // EPC1 /// ENST00000263062 // EPC1 /// BC036529 // EPC1 /// ENST0000C	EPC1	chr10	0.0428512	-1.52769
3286294	NM_001098208 // HNRNPF /// NM_001098204 // HNRNPF /// NM_004966 // HNRNPF /// NM	HNRNPF	chr10	0.0341088	-1.74941
3293225	NM_173555 // TYSND1 /// ENST00000287078 // TYSND1 /// BC111501 // TYSND1	TYSND1	chr10	0.0329905	-1.93868
3293314	NM_022146 // NPFFR1 /// ENST00000449957 // NPFFR1 /// AF268898 // NPFFR1 /// ENS	NPFFR1	chr10	0.00170092	-2.97308
3293931	NM_015947 // ASCC1 /// ENST00000394919 // ASCC1 /// ENST00000317126 // ASCC1	ASCC1	chr10	0.037777	-1.54185
3294385	NM_145170 // TTC18 /// ENST00000355577 // TTC18 /// ENST00000310715 // TTC18 //.	TTC18	chr10	0.0355612	-1.50631
3294866	NM_172171 // CAMK2G /// NM_172169 // CAMK2G /// NM_172170 // CAMK2G /// NM_00122	CAMK2G	chr10	0.00833885	-1.74901
3298697	NM_017551 // GRID1 /// AK293104 // GRID1	GRID1	chr10	0.0390286	-1.65387
3298941	NM_024756 // MMRN2 /// ENST00000372027 // MMRN2 /// BC094744 // MMRN2 /// ENST0C	MMRN2	chr10	0.00877232	-1.533
3299667	NM_213606 // SLC16A12 /// ENST00000341233 // SLC16A12 /// AK124901 // SLC16A12 .	SLC16A12	chr10	0.0358547	-1.50877
3301335	NM_006434 // SORBS1 /// NM_015385 // SORBS1 /// NM_001034954 // SORBS1 /// NM_0C	SORBS1	chr10	0.0176891	-1.97691
3301747	NM_013314 // BLNK /// NM_001114094 // BLNK /// ENST00000224337 // BLNK /// ENST(	BLNK	chr10	0.0166798	-2.16545
3301803	NM_012465 // TLL2 /// ENST00000357947 // TLL2 /// AF059516 // TLL2	TLL2	chr10	0.0037638	-1.50536
3301813	NM_012465 // TLL2 /// ENST00000357947 // TLL2 /// AF059516 // TLL2	TLL2	chr10	0.0137851	-1.56871
3302368	NM_022362 // MMS19 /// ENST00000370782 // MMS19 /// ENST00000438925 // MMS19 //	MMS19	chr10	0.049408	-1.64374
3302810	NM_000195 // HPS1 /// ENST00000325103 // HPS1 /// ENST00000361490 // HPS1 /// U6	HPS1	chr10	0.0245554	-1.82461
3303001	NM_002079 // GOT1 /// ENST00000370508 // GOT1 /// M37400 // GOT1	GOT1	chr10	0.0191017	-1.66834
3303135	NM_078470 // COX15 /// NM_004376 // COX15 /// ENST00000016171 // COX15 /// ENST(	COX15	chr10	0.0287234	-1.68126
3303711	NM_024895 // PDZD7 /// ENST00000370215 // PDZD7 /// ENST00000370216 // PDZD7 //.	PDZD7	chr10	0.0317726	-1.51034
3304115	NM_014591 // KCNIP2 /// NM_173191 // KCNIP2 /// NM_173192 // KCNIP2 /// NM_1731S	KCNIP2	chr10	0.0340839	-1.93736

3304872	NM_014631 // SH3PXD2A /// ENST00000355946 // SH3PXD2A /// ENST00000420222 // SH3	SH3PXD2A	chr10	0.0394287	-1.73104
3305222	NM_025145 // C10orf79 /// ENST00000389588 // C10orf79 /// ENST00000428666 // C10	C10orf79	chr10	0.0343338	-1.58854
3308897	NM_014904 // RAB11FIP2 /// ENST00000355624 // RAB11FIP2 /// AY037299 // RAB11FIF	RAB11FIP2	chr10	0.0413871	-1.5263
3309249	NM_003750 // EIF3A /// ENST00000369144 // EIF3A /// U78311 // EIF3A	EIF3A	chr10	0.00203001	-1.66764
3311374	NM_212554 // METTL10 /// ENST00000368836 // METTL10 /// BC026167 // METTL10	METTL10	chr10	0.00600962	-2.01
3314775	NM_173572 // C10orf93 /// BC146900 // C10orf93	C10orf93	chr10	0.00172209	-1.61758
3315230	NM_001098483 // C10orf125 /// NM_198472 // C10orf125 /// ENST00000368552 // C10c	C10orf125	chr10	0.026084	-1.82831
3315245	NM_004092 // ECHS1 /// ENST00000368547 // ECHS1 /// BC008906 // ECHS1	ECHS1	chr10	0.00240631	-1.57035
3315732	NM_178537 // B4GALNT4 /// ENST00000329962 // B4GALNT4 /// AB089939 // B4GALNT4	B4GALNT4	chr11	0.000877197	-2.14147
3315921	NM_198075 // LRRC56 /// ENST00000270115 // LRRC56 /// ENST00000422406 // LRRC56	LRRC56	chr11	0.0481644	-1.73478
3317411	NM_000218 // KCNQ1 /// NM_181798 // KCNQ1 /// ENST00000155840 // KCNQ1 /// ENST0	KCNQ1	chr11	0.0462345	-1.60969
3317527	NM_002555 // SLC22A18 /// NM_183233 // SLC22A18 /// ENST00000347936 // SLC22A18	SLC22A18	chr11	0.0359586	-1.69507
3318385	NM_001005162 // OR52B6 /// ENST00000345043 // OR52B6	OR52B6	chr11	0.0222707	-1.50362
3319615	NM_000990 // RPL27A /// ENST00000314138 // RPL27A	RPL27A	chr11	0.0432762	-1.51299
3320172	NM_000480 // AMPD3 /// ENST00000256183 // AMPD3 /// ENST00000444303 // AMPD3 //	AMPD3	chr11	0.0201574	-1.68106
3320840	NM_032867 // MICALCL /// ENST00000256186 // MICALCL	MICALCL	chr11	0.0247555	-1.51302
3320841	NM_032867 // MICALCL /// ENST00000256186 // MICALCL	MICALCL	chr11	0.0418153	-1.81435
3321560	NM_000922 // PDE3B /// ENST00000282096 // PDE3B /// BC150307 // PDE3B	PDE3B	chr11	0.000510014	-1.51176
3322701	---	0	chr11	0.00786156	-2.95742
3322728	NM_005316 // GTF2H1 /// NM_001142307 // GTF2H1 /// ENST00000453096 // GTF2H1 //	GTF2H1	chr11	0.0387501	-1.52756
3324201	NM_001009909 // LUZP2 /// ENST00000336930 // LUZP2 /// AK127695 // LUZP2	LUZP2	chr11	0.0150631	-1.54861
3327077	NM_001160167 // PRR5L /// NM_024841 // PRR5L /// NM_001160169 // PRR5L /// ENST0	PRR5L	chr11	0.0285123	-1.53357
3328633	NM_130783 // TSPAN18 /// ENST00000425677 // TSPAN18 /// ENST00000340160 // TSPAN	TSPAN18	chr11	0.0244362	-1.81885
3329781	NM_130470 // MADD /// NM_001135944 // MADD /// NM_130471 // MADD /// NM_130472	MADD	chr11	0.0385125	-1.78974
3331291	NM_178570 // RTN4RL2 /// ENST00000335099 // RTN4RL2 /// AY250221 // RTN4RL2	RTN4RL2	chr11	0.00495493	-1.7762
3331365	NM_000062 // SERPING1 /// NM_001032295 // SERPING1 /// ENST00000278407 // SERPIN	SERPING1	chr11	0.0244767	-1.94349
3331434	NM_015457 // ZDHHC5 /// ENST00000287169 // ZDHHC5 /// AY894889 // ZDHHC5	ZDHHC5	chr11	0.038576	-1.60695
3331734	NM_053023 // ZFP91 /// NR_024091 // ZFP91-CNTF /// ENST00000316059 // ZFP91 ///	ZFP91	chr11	0.04497	-1.50779
3332681	NM_006725 // CD6 /// ENST00000313421 // CD6 /// BC033755 // CD6 /// BC078669 //	CD6	chr11	0.00411433	-1.83003
3332693	NM_006725 // CD6 /// ENST00000313421 // CD6 /// BC033755 // CD6 /// BC078669 //	CD6	chr11	0.0324729	-1.5572
3333213	NM_013279 // C11orf9 /// NM_001127392 // C11orf9 /// ENST00000278836 // C11orf9	C11orf9	chr11	0.0464157	-1.72821
3333234	NM_004111 // FEN1 /// ENST00000305885 // FEN1 /// BC000323 // FEN1	FEN1	chr11	0.0412688	-1.62646
3334506	NM_012094 // PRDX5 /// NM_181651 // PRDX5 /// ENST00000265462 // PRDX5 /// ENST0	PRDX5	chr11	0.00183463	-1.75249
3334591	NM_003942 // RPS6KA4 /// NM_001006944 // RPS6KA4 /// ENST00000334205 // RPS6KA4	RPS6KA4	chr11	0.00482019	-1.69529
3335296	NM_020680 // SCYL1 /// NM_001048218 // SCYL1 /// ENST00000270176 // SCYL1 /// EN	SCYL1	chr11	0.0465975	-1.65668
3335477	NM_153253 // SIPA1 /// NM_006747 // SIPA1 /// ENST00000394224 // SIPA1 /// ENST0	SIPA1	chr11	0.0489607	-1.57879
3335524	NM_182710 // KAT5 /// NM_006388 // KAT5 /// NM_182709 // KAT5 /// ENST0000037704	KAT5	chr11	0.0312697	-1.61033
3335626	NM_152760 // SNX32 /// ENST00000308342 // SNX32 /// AK055496 // SNX32	SNX32	chr11	0.00240972	-1.58123
3335711	NM_001335 // CTSW /// ENST00000307886 // CTSW /// BC048255 // CTSW	CTSW	chr11	0.00906422	-1.72625
3335850	---	0	chr11	0.00877726	-1.72477
3335886	NM_003860 // BANF1 /// NM_001143985 // BANF1 /// ENST00000312175 // BANF1 /// EN	BANF1	chr11	0.0105993	-1.54652
3336119	NM_153266 // TMEM151A /// ENST00000327259 // TMEM151A /// BC033898 // TMEM151A	TMEM151A	chr11	0.0380319	-1.80213
3336835	NM_001619 // ADRBK1 /// ENST00000308595 // ADRBK1 /// AB209588 // ADRBK1 /// ENS	ADRBK1	chr11	0.0263	-1.61279
3337954	NM_139075 // TPCN2 /// ENST00000294309 // TPCN2 /// BC063008 // TPCN2 /// AK2989	TPCN2	chr11	0.0210756	-1.50658
3338387	NM_018043 // ANO1 /// NR_030691 // ANO1 /// ENST00000355303 // ANO1 /// AY728143	ANO1	chr11	0.0458761	-1.54078
3339437	NM_001567 // INPPL1 /// ENST00000298229 // INPPL1 /// Y14385 // INPPL1	INPPL1	chr11	0.0373643	-1.89343
3339890	NM_032871 // RELT /// NM_152222 // RELT /// ENST00000064780 // RELT /// ENST0000	RELT	chr11	0.0102692	-1.85591
3341149	NM_138706 // B3GNT6 /// ENST00000354301 // B3GNT6 /// AK292773 // B3GNT6 /// ENS	B3GNT6	chr11	0.044705	-2.07469
3341178	NM_004055 // CAPN5 /// ENST00000278559 // CAPN5 /// ENST00000360841 // CAPN5 //	CAPN5	chr11	0.00319833	-1.59533
3346414	BC054869 // C11orf70 /// BC006128 // C11orf70 /// AK094851 // C11orf70 /// ENST0	C11orf70	chr11	0.0415648	-1.51475
3346458	NM_001130145 // YAP1 /// NM_006106 // YAP1 /// ENST00000345877 // YAP1 /// ENST0	YAP1	chr11	0.0244975	-1.61108

3349368	NM_000615 // NCAM1 /// NM_181351 // NCAM1 /// NM_001076682 // NCAM1 /// ENST000(	NCAM1	chr11	0.0378283	-1.59676
3349889	NM_006169 // NNMT /// ENST00000299964 // NNMT /// BC000234 // NNMT	NNMT	chr11	0.0177432	-1.95219
3351357	NM_004788 // UBE4A /// ENST00000252108 // UBE4A /// BC112367 // UBE4A /// ENST0(	UBE4A	chr11	0.00707855	-1.53688
3351718	NM_181721 // FOXR1 /// ENST00000317011 // FOXR1 /// BC028191 // FOXR1 /// ENST0(	FOXR1	chr11	0.0102338	-1.59599
3352036	NM_024618 // NLRX1 /// ENST00000409991 // NLRX1 /// ENST00000292199 // NLRX1 //.	NLRX1	chr11	0.0350955	-1.51054
3353353	NM_032873 // UBASH3B /// ENST00000284273 // UBASH3B /// BC007541 // UBASH3E	UBASH3B	chr11	0.018434	-1.95475
3354333	NM_022370 // ROBO3 /// ENST00000397801 // ROBO3 /// AY509035 // ROBO3	ROBO3	chr11	0.0467356	-1.55327
3354474	NM_198277 // SLC37A2 /// NM_001145290 // SLC37A2 /// ENST00000403796 // SLC37A2	SLC37A2	chr11	0.0173136	-1.55027
3354912	NM_013264 // DDX25 /// ENST00000263576 // DDX25 /// AF155140 // DDX25	DDX25	chr11	0.0115598	-1.66194
3356118	NM_001642 // APLP2 /// NM_001142276 // APLP2 /// NM_001142277 // APLP2 /// NM_0(	APLP2	chr11	0.0420198	-1.94365
3356153	NM_001642 // APLP2 /// NM_001142276 // APLP2 /// NM_001142277 // APLP2 /// NM_0(	APLP2	chr11	0.0344113	-1.54066
3357305	NM_014384 // ACAD8 /// ENST00000281182 // ACAD8 /// ENST00000374752 // ACAD8 //.	ACAD8	chr11	0.00329107	-1.55174
3357347	NM_001080407 // GLB1L3 /// ENST00000410100 // GLB1L3 /// ENST00000431683 // GLB1	GLB1L3	chr11	0.00389242	-1.61719
3357887	NM_001135054 // SIGIRR /// NM_001135053 // SIGIRR /// NM_021805 // SIGIRR /// EN	SIGIRR	chr11	0.00279831	-1.64299
3358180	NM_001572 // IRF7 /// NM_004029 // IRF7 /// NM_004031 // IRF7 /// ENST0000039757	IRF7	chr11	0.023749	-1.6864
3358203	NM_021924 // CDHR5 /// NM_031264 // CDHR5 /// NM_001171968 // CDHR5 /// ENST000(	CDHR5	chr11	0.00304358	-1.60791
3358213	NM_021924 // CDHR5 /// NM_001171968 // CDHR5 /// ENST00000397542 // MUPCDH /// E	CDHR5	chr11	0.0266396	-1.96419
3358244	NM_021920 // SCT /// ENST00000176195 // SCT	SCT	chr11	0.0265826	-1.6198
3360775	NM_032127 // FAM160A2 /// NM_001098794 // FAM160A2 /// ENST00000449352 // FAM16(	FAM160A2	chr11	0.00619535	-1.52192
3360848	NM_001164 // APBB1 /// NM_145689 // APBB1 /// ENST00000389906 // APBB1 /// ENST(	APBB1	chr11	0.00807782	-2.55423
3361392	NM_016229 // CYB5R2 /// ENST00000299498 // CYB5R2 /// AB209000 // CYB5R2 /// ENS	CYB5R2	chr11	0.0250927	-1.65883
3364368	NM_017508 // SOX6 /// NM_033326 // SOX6 /// NM_001145811 // SOX6 /// NM_0011458	SOX6	chr11	0.0367705	-1.52522
3369404	ENST00000395750 // SLC1A2	SLC1A2	chr11	0.0444217	-2.16516
3371019	NM_006034 // TP53I11 /// NM_001076787 // TP53I11 /// ENST00000308212 // TP53I11	TP53I11	chr11	0.0377827	-1.97715
3371124	NM_020826 // SYT13 /// ENST00000020926 // SYT13 /// AK291009 // SYT13	SYT13	chr11	0.0132322	-1.56586
3371321	NM_004813 // PEX16 /// NM_057174 // PEX16 /// ENST00000378750 // PEX16 /// ENST(	PEX16	chr11	0.0292422	-1.73297
3371621	NM_017749 // AMBRA1 /// ENST00000314845 // AMBRA1 /// DQ870924 // AMBRA1 /// BC(	AMBRA1	chr11	0.00444493	-1.65883
3371972	NM_016223 // PACSIN3 /// ENST00000298838 // PACSIN3 /// AF130979 // PACSIN3 ///	PACSIN3	chr11	0.000449543	-1.6357
3373051	NM_001005270 // OR4C12 /// ENST00000335238 // OR4C12 /// BC136850 // OR4C12	OR4C12	chr11	0.0453841	-1.74792
3373704	NM_033396 // TNKS1BP1 /// ENST00000358252 // TNKS1BP1 /// BC150333 // TNKS1BP1 /	TNKS1BP1	chr11	0.0374249	-1.54077
3373876	NM_017611 // SLC43A3 /// ENST00000352187 // SLC43A3 /// AB028927 // SLC43A3	SLC43A3	chr11	0.0163019	-1.5322
3375884	NM_004739 // MTA2 /// ENST00000278823 // MTA2 /// BC053650 // MTA2	MTA2	chr11	0.0232201	-1.5577
3376036	NM_015853 // UBXN1 /// ENST00000294119 // UBXN1 /// BC032689 // UBXN1 /// ENST0(	UBXN1	chr11	0.00591012	-1.64058
3376191	NM_006362 // NXF1 /// NM_001081491 // NXF1 /// ENST00000294172 // NXF1 /// ENST(	NXF1	chr11	0.0164134	-1.73435
3376698	NM_173587 // RCOR2 /// ENST00000301459 // RCOR2 /// BC023587 // RCOR2	RCOR2	chr11	0.00928899	-1.68323
3376798	NM_001033678 // TRPT1 /// NM_031472 // TRPT1 /// NM_001160389 // TRPT1 /// NM_0(	TRPT1	chr11	0.0379127	-1.65074
3376919	NM_015080 // NRXN2 /// NM_138732 // NRXN2 /// NM_138734 // NRXN2 /// ENST0000037	NRXN2	chr11	0.0172454	-2.05511
3377405	NM_005468 // NAALADL1 /// ENST00000358658 // NAALADL1 /// ENST00000355721 // NAA	NAALADL1	chr11	0.0137659	-1.5159
3377679	NM_001130144 // LTBP3 /// NM_021070 // LTBP3 /// NM_001164266 // LTBP3 /// ENST(	LTBP3	chr11	0.00838773	-1.8942
3378057	NM_053054 // CATSPER1 /// ENST00000312106 // CATSPER1 /// BC032950 // CATSPER1	CATSPER1	chr11	0.0144364	-1.54697
3378252	NM_001532 // SLC29A2 /// ENST00000357440 // SLC29A2 /// AK057041 // SLC29A2 ///	SLC29A2	chr11	0.00736039	-1.52964
3378858	NM_206997 // GPR152 /// ENST00000312457 // GPR152 /// AY569571 // GPR152	GPR152	chr11	0.0189566	-1.85264
3378909	NM_004910 // PITPNM1 /// NM_001130848 // PITPNM1 /// ENST00000356404 // PITPNM1	PITPNM1	chr11	0.0132908	-1.56943
3379036	NM_181843 // NUDT8 /// ENST00000301490 // NUDT8 /// BC018644 // NUDT8 /// ENST00	NUDT8	chr11	0.0223198	-1.74187
3379646	NM_001031847 // CPT1A /// ENST00000376618 // CPT1A /// BC000185 // CPT1A	CPT1A	chr11	0.0269693	-2.2427
3379654	NM_001876 // CPT1A /// NM_001031847 // CPT1A /// ENST00000265641 // CPT1A /// EN	CPT1A	chr11	0.0471878	-1.51495
3379715	NM_181515 // MRPL21 /// NM_181514 // MRPL21 /// ENST00000362034 // MRPL21 /// EN	MRPL21	chr11	0.00195999	-3.26011
3381352	NM_006645 // STARD10 /// ENST00000334805 // STARD10 /// BC007919 // STARD10	STARD10	chr11	0.014472	-1.51178
3382178	NM_001005285 // OR2AT4 /// ENST00000305159 // OR2AT4 /// BC136958 // OR2AT4	OR2AT4	chr11	0.00499713	-1.84918
3382225	NM_004041 // ARRB1 /// NM_020251 // ARRB1 /// ENST00000393505 // ARRB1 /// ENST(	ARRB1	chr11	0.0322019	-1.67029
3382544	NM_004626 // WNT11 /// ENST00000322563 // WNT11 /// AB070218 // WNT11 /// ENST00	WNT11	chr11	0.014922	-1.61071

3388725	NM_004771 // MMP20 /// ENST00000260228 // MMP2(	MMP20	chr11	0.00239897	-1.9639
3388758	NM_002424 // MMP8 /// ENST00000236826 // MMP8 /// AK303388 // MMP8 /// ENST0000(	MMP8	chr11	0.0234275	-1.51474
3390103	NM_002519 // NPAT /// ENST00000278612 // NPAT /// D83243 // NPAT /// ENST0000041	NPAT	chr11	0.000181915	-1.5601
3391657	NM_000795 // DRD2 /// NM_016574 // DRD2 /// ENST00000362072 // DRD2 /// ENST000(	DRD2	chr11	0.0142889	-1.69656
3392394	NM_014333 // CADM1 /// NM_001098517 // CADM1 /// ENST00000331581 // CADM1 /// EN	CADM1	chr11	0.0408377	-1.51224
3393994	NM_182557 // BCL9L /// ENST00000334801 // BCL9L /// AB094091 // BCL9L /// ENST0(	BCL9L	chr11	0.0272674	-1.50694
3394021	NM_182557 // BCL9L /// ENST00000334801 // BCL9L /// AB094091 // BCL9L /// ENST0(	BCL9L	chr11	0.0359838	-1.67636
3394103	NM_001164277 // SLC37A4 /// NM_001164278 // SLC37A4 /// NM_001164279 // SLC37A4	SLC37A4	chr11	0.00413236	-1.5684
3394190	NM_002105 // H2AFX /// ENST00000375167 // H2AFX /// BC011694 // H2AF>	H2AFX	chr11	0.00204855	-1.56224
3394399	NM_004205 // USP2 /// ENST00000260187 // USP2 /// BC041366 // USP2	USP2	chr11	0.0441607	-1.62203
3396898	NM_032795 // RPUSD4 /// NM_001144827 // RPUSD4 /// ENST00000298317 // RPUSD4 //.	RPUSD4	chr11	0.0458083	-1.54093
3397027	NM_032531 // KIRREL3 /// NM_001161707 // KIRREL3 /// ENST00000278934 // KIRREL3	KIRREL3	chr11	0.0388527	-1.51396
3397815	NM_145013 // C11orf45 /// BC025756 // C11orf45 /// ENST00000310799 // C11orf45	C11orf45	chr11	0.0466063	-1.60413
3397824	NM_145013 // C11orf45 /// BC025756 // C11orf45 /// ENST00000310799 // C11orf45	C11orf45	chr11	0.0284921	-1.56761
3398106	NM_001143835 // NFRKB /// NM_006165 // NFRKB /// ENST00000304521 // NFRKB /// EN	NFRKB	chr11	0.0330185	-1.87462
3398116	NM_001143835 // NFRKB /// NM_006165 // NFRKB /// ENST00000304521 // NFRKB /// EN	NFRKB	chr11	0.0255683	-1.50259
3401090	NM_199460 // CACNA1C /// NM_001129836 // CACNA1C /// NM_001129837 // CACNA1C //.	CACNA1C	chr12	0.0255773	-1.55599
3402427	NM_018173 // PLEKHG6 /// NM_001144856 // PLEKHG6 /// NM_001144857 // PLEKHG6 //.	PLEKHG6	chr12	0.0267661	-1.82454
3402470	NM_002342 // LTBR /// ENST00000228918 // LTBR /// BC026262 // LTBR	LTBR	chr12	0.000358753	-1.659
3402530	NM_018009 // TAPBPL /// ENST00000266556 // TAPBPL /// AK098035 // TAPBPL	TAPBPL	chr12	0.0373866	-1.55007
3413902	NM_005480 // TROAP /// ENST00000257909 // TROAP /// U04810 // TROAP	TROAP	chr12	0.037142	-1.81444
3414327	---	0	chr12	0.0402272	-1.59096
3414749	NM_014033 // METTL7A /// ENST00000332160 // METTL7A /// BC004492 // METTL7A ///	METTL7A	chr12	0.0395558	-2.12772
3414779	NM_015416 // LETMD1 /// ENST00000262055 // LETMD1 /// AF195651 // LETMD1 /// ENS	LETMD1	chr12	0.0322336	-1.58678
3415093	NM_001130015 // ANKRD33 /// NM_182608 // ANKRD33 /// ENST00000340970 // ANKRD33	ANKRD33	chr12	0.0411209	-1.58855
3415326	NM_005556 // KRT7 /// ENST00000331817 // KRT7 /// ENST00000422319 // KRT7 /// AK	KRT7	chr12	0.00627099	-1.65774
3415977	NM_138473 // SP1 /// NM_003109 // SP1 /// ENST00000426431 // SP1 /// ENST0000032	SP1	chr12	0.0275344	-1.74974
3416010	NM_020547 // AMHR2 /// NM_001164690 // AMHR2 /// NM_001164691 // AMHR2 /// ENST(	AMHR2	chr12	0.0192304	-2.30255
3416538	NM_016057 // COPZ1 /// ENST00000262061 // COPZ1 /// BC002849 // COPZ1 /// AF1518	COPZ1	chr12	0.0127467	-1.65001
3417984	NM_002332 // LRP1 /// ENST00000243077 // LRP1 /// AK122840 // LRP1	LRP1	chr12	0.0354559	-1.62093
3418146	NM_005269 // GLI1 /// NM_001160045 // GLI1 /// NM_001167609 // GLI1 /// ENST000(	GLI1	chr12	0.023533	-1.64525
3418427	NM_001478 // B4GALNT1 /// NM_133489 // SLC26A10 /// ENST00000320442 // SLC26A10	B4GALNT1	chr12	0.00251301	-1.68145
3418558	NM_001172696 // TSFM /// NM_005726 // TSFM /// NM_001172695 // TSFM /// ENST000(	TSFM	chr12	0.00800211	-1.54583
3418617	NM_033276 // XRCC6BP1 /// ENST00000300145 // XRCC6BP1 /// AF078164 // XRCC6BP1	XRCC6BP1	chr12	0.0492077	-1.70423
3423210	NM_015336 // ZDHHC17 /// ENST00000334822 // ZDHHC17 /// ENST00000426126 // ZDHHC	ZDHHC17	chr12	0.00876803	-1.60326
3426509	NM_005761 // PLXNC1 /// ENST00000258526 // PLXNC1 /// AF030339 // PLXNC1	PLXNC1	chr12	0.0145194	-1.63953
3427911	NM_013229 // APAF1 /// NM_001160 // APAF1 /// NM_181861 // APAF1 /// NM_181868	APAF1	chr12	0.00438174	-1.56737
3428151	NM_017988 // SCYL2 /// ENST00000360820 // SCYL2 /// AK292980 // SCYL2 /// ENST0(	SCYL2	chr12	0.00391357	-1.61359
3430140	NM_018082 // POLR3B /// NM_001160708 // POLR3B /// ENST00000228347 // POLR3B //.	POLR3B	chr12	0.0237479	-1.57448
3430180	NM_018082 // POLR3B /// NM_001160708 // POLR3B /// ENST00000228347 // POLR3B //.	POLR3B	chr12	0.0128146	-1.58164
3432575	NM_004416 // DTX1 /// ENST00000257600 // DTX1 /// BC048216 // DTX1	DTX1	chr12	0.0258707	-1.73007
3434146	NM_006253 // PRKAB1 /// ENST00000229328 // PRKAB1 /// BC017671 // PRKAB1	PRKAB1	chr12	0.0112843	-1.5717
3435443	NM_014708 // KNTC1 /// ENST00000333479 // KNTC1 /// BC150278 // KNTC1 /// ENST00	KNTC1	chr12	0.0210121	-1.82785
3435593	NM_003959 // HIP1R /// ENST00000253083 // HIP1R /// AB013384 // HIP1R	HIP1R	chr12	0.0377798	-1.68986
3436349	NM_181709 // FAM101A /// ENST00000324038 // FAM101A /// ENST00000389727 // FAM1(	FAM101A	chr12	0.0202391	-1.72831
3438186	NM_198827 // GPR133 /// ENST00000261654 // GPR133 /// BC143775 // GPR133 /// ENS	GPR133	chr12	0.0110988	-2.08522
3438555	NM_003565 // ULK1 /// ENST00000321867 // ULK1 /// AF045458 // ULK1	ULK1	chr12	0.00516647	-1.88141
3439565	NM_016615 // SLC6A13 /// ENST00000343164 // SLC6A13 /// BC022392 // SLC6A13 ///	SLC6A13	chr12	0.00227677	-1.64899
3439738	NM_016533 // NINJ2 /// ENST00000305108 // NINJ2 /// AK296012 // NINJ2 /// ENST0(	NINJ2	chr12	0.008317	-1.58364
3440069	NM_172364 // CACNA2D4 /// ENST00000280663 // CACNA2D4 /// ENST00000382722 // CAC	CACNA2D4	chr12	0.0295927	-1.90761
3441992	NM_080730 // IFFO1 /// NM_080731 // IFFO1 /// NM_001039670 // IFFO1 /// ENST000(	IFFO1	chr12	0.0401914	-1.65271

3442021	NM_080730 // IFFO1 /// NM_001039670 // IFFO1 /// ENST00000396840 // IFFO1 /// EN	IFFO1	chr12	0.0278699	-1.58429
3442036	NM_006170 // NOP2 /// NM_001033714 // NOP2 /// ENST00000399466 // NOP2 /// M3211	NOP2	chr12	0.0174048	-1.73489
3443468	NM_002864 // PZP /// ENST00000261336 // PZP /// BC111756 // PZP /// ENST00000381	PZP	chr12	0.00186096	-1.57643
3443948	NM_016509 // CLEC1B /// ENST00000298527 // CLEC1B /// AF124841 // CLEC1B /// AY3	CLEC1B	chr12	0.0482705	-1.64893
3445259	NM_182558 // C12orf36 /// ENST00000318426 // C12orf36	C12orf36	chr12	0.00522088	-1.5277
3445757	NM_000900 // MGP /// ENST00000228938 // MGP	MGP	chr12	0.0290952	-1.66468
3451260	NM_173601 // GXYLT1 /// NM_001099650 // GXYLT1 /// ENST00000398675 // GLT8D3 //.	GXYLT1	chr12	0.0334502	-1.57859
3452695	NM_001098531 // RAPGEF3 /// NM_006105 // RAPGEF3 /// NM_001098532 // RAPGEF3 //.	RAPGEF3	chr12	0.0463374	-1.60087
3452729	NM_001098531 // RAPGEF3 /// NM_006105 // RAPGEF3 /// NM_001098532 // RAPGEF3 //.	RAPGEF3	chr12	0.0187801	-1.58517
3454098	NM_001037806 // NCKAP5L /// ENST00000335999 // NCKAP5L /// AB046822 // NCKAP5L /	NCKAP5L	chr12	0.00607187	-1.98698
3455986	NM_032840 // SPRYD3 /// ENST00000301463 // SPRYD3 /// AK074694 // SPRYD3	SPRYD3	chr12	0.0249369	-1.51863
3458161	NM_000946 // PRIM1 /// ENST00000338193 // PRIM1 /// BC005266 // PRIM1	PRIM1	chr12	0.000928631	-1.6573
3460469	NR_026825 // RPSAP52 /// BC107865 // RPSAP52	RPSAP52	chr12	0.0147969	-1.68076
3464675	NM_025114 // CEP290 /// ENST00000309041 // CEP290 /// DQ109808 // CEP290 /// ENS	CEP290	chr12	0.0185918	-1.50564
3470295	BC041638 // SART3	SART3	chr12	0.0210764	-1.57266
3472771	NM_005996 // TBX3 /// NM_016569 // TBX3 /// ENST00000349155 // TBX3 /// ENST0000	TBX3	chr12	0.0243845	-1.52529
3473346	AK298857 // C12orf49 /// AK025068 // C12orf49 /// ENST00000261318 // C12orf49	C12orf49	chr12	0.00126089	-1.89207
3473529	NM_000620 // NOS1 /// ENST00000317775 // NOS1 /// U17327 // NOS1 /// ENST0000039	NOS1	chr12	0.00821733	-1.72363
3473840	NM_016281 // TAOK3 /// ENST00000392533 // TAOK3 /// AF135158 // TAOK3 /// AF1819	TAOK3	chr12	0.0391873	-1.66063
3474141	NM_007174 // CIT /// ENST00000261833 // CIT /// AY681966 // CIT /// ENST00000392	CIT	chr12	0.0179456	-1.72679
3474425	BC052611 // PXN /// AB209034 // PXN /// ENST00000323871 // PXN	PXN	chr12	0.0296655	-1.54772
3474463	NM_002442 // MSI1 /// ENST00000257552 // MSI1	MSI1	chr12	0.0453451	-1.70004
3474903	NM_006549 // CAMKK2 /// NM_153499 // CAMKK2 /// NM_172216 // CAMKK2 /// NM_1535(	CAMKK2	chr12	0.0102008	-1.68311
3475138	---	0	chr12	0.0124966	-1.50279
3475218	NM_032590 // KDM2B /// NM_001005366 // KDM2B /// ENST00000377071 // KDM2B /// EN	KDM2B	chr12	0.000244033	-1.8136
3476481	NM_006312 // NCOR2 /// NM_001077261 // NCOR2 /// ENST00000356219 // NCOR2 /// EN	NCOR2	chr12	0.0194322	-1.67328
3476593	NM_006312 // NCOR2 /// NM_001077261 // NCOR2 /// ENST00000356219 // NCOR2 /// EN	NCOR2	chr12	0.00303936	-1.68826
3479224	NM_006231 // POLE /// ENST00000320574 // POLE /// ENST00000455752 // POLE /// BC	POLE	chr12	0.0110396	-1.50482
3481918	NM_001676 // ATP12A /// ENST00000381946 // ATP12A /// BC031609 // ATP12A /// ENS	ATP12A	chr13	0.00683391	-1.56649
3482285	NM_016529 // ATP8A2 /// ENST00000281620 // ATP8A2 /// ENST00000381655 // ATP8A2	ATP8A2	chr13	0.0453924	-1.7484
3483268	NM_175854 // PAN3 /// ENST00000399613 // PAN3 /// ENST00000282391 // PAN3 /// BC	PAN3	chr13	0.0245932	-1.5137
3483508	NM_015233 // MTUS2 /// ENST00000380808 // KIAA0774 /// ENST00000400542 // KIAA07	MTUS2	chr13	0.012967	-1.68042
3483518	NM_001033602 // MTUS2 /// NM_015233 // MTUS2 /// ENST00000255289 // KIAA0774 //.	MTUS2	chr13	0.000346483	-1.61907
3484530	NM_023037 // FRY /// ENST00000380250 // FRY /// ENST00000267067 // FRY /// ENST(	FRY	chr13	0.0391609	-1.66425
3486038	NM_016617 // UFM1 /// ENST00000239878 // UFM1 /// BC005193 // UFM1 /// AB154404	UFM1	chr13	0.014727	-1.53831
3488946	NM_018283 // NUDT15 /// ENST00000258662 // NUDT15 /// BC064607 // NUDT15	NUDT15	chr13	0.0420503	-1.51483
3494648	NM_144777 // SCEL /// NM_003843 // SCEL /// NM_001160706 // SCEL /// ENST0000034	SCEL	chr13	0.00180852	-1.87986
3495979	NM_015567 // SLITRK5 /// ENST00000325089 // SLITRK5 /// BC098106 // SLITRK5	SLITRK5	chr13	0.0238196	-1.61686
3498788	NM_007129 // ZIC2 /// ENST00000376335 // ZIC2 /// AF193855 // ZIC2 /// ENST0000(	ZIC2	chr13	0.0177174	-1.62194
3499587	NM_017693 // BIVM /// NM_001159596 // BIVM /// ENST00000257336 // BIVM /// ENST(	BIVM	chr13	0.0374174	-1.53612
3501316	NM_001846 // COL4A2 /// ENST00000360467 // COL4A2 /// ENST00000257309 // COL4A2	COL4A2	chr13	0.0300056	-1.61467
3501351	NM_001846 // COL4A2 /// ENST00000360467 // COL4A2 /// AK294920 // COL4A2 /// ENS	COL4A2	chr13	0.0226326	-1.88239
3501674	NM_003899 // ARHGEF7 /// NM_145735 // ARHGEF7 /// NM_001113511 // ARHGEF7 /// EN	ARHGEF7	chr13	0.0157806	-1.6725
3501704	NM_003899 // ARHGEF7 /// NM_145735 // ARHGEF7 /// NM_001113511 // ARHGEF7 /// NM	ARHGEF7	chr13	0.0410064	-1.73309
3502479	NM_003891 // PROZ /// ENST00000375547 // PROZ /// M55671 // PROZ /// ENST0000034	PROZ	chr13	0.00145252	-1.64508
3509530	NM_004734 // DCLK1 /// ENST00000255448 // DCLK1 /// BC152456 // DCLK1 /// ENST0(	DCLK1	chr13	0.0341843	-1.71156
3510905	NM_002015 // FOXO1 /// ENST00000379561 // FOXO1 /// AF032885 // FOXO1	FOXO1	chr13	0.00278053	-1.75089
3511907	NM_017993 // ENOX1 /// NM_001127615 // ENOX1 /// ENST00000261488 // ENOX1 /// EN	ENOX1	chr13	0.0271353	-1.69058
3518427	NM_138444 // KCTD12 /// ENST00000377474 // KCTD12 /// ENST00000317765 // KCTD12	KCTD12	chr13	0.0218685	-1.56355
3525611	NM_024537 // CARS2 /// ENST00000257347 // CARS2 /// BC007220 // CARS2	CARS2	chr13	0.0344478	-1.61879
3527758	NM_001029991 // METT11D1 /// NM_022734 // METT11D1 /// ENST00000339374 // METT11	METT11D1	chr14	0.0153071	-1.76123

3528845	NM_181304 // MRPL52 /// NM_181305 // MRPL52 /// ENST00000397496 // MRPL52 /// AK	MRPL52	chr14	0.0440809	-1.66984
3529605	BC042179 // FITM1	FITM1	chr14	0.00967087	-1.51452
3529886	NM_019839 // LTB4R2 /// NM_001164692 // LTB4R2 /// ENST00000336557 // LTB4R2 //.	LTB4R2	chr14	0.048735	-1.50313
3531041	NM_016106 // SCFD1 /// NM_182835 // SCFD1 /// ENST00000458591 // SCFD1 /// ENST(	SCFD1	chr14	0.0390655	-1.54149
3534825	NM_152329 // PPIL5 /// NM_203467 // PPIL5 /// ENST00000298288 // PPIL5 /// ENST(	PPIL5	chr14	0.0280305	-1.57598
3536562	NM_015589 // SAMD4A /// NM_001161576 // SAMD4A /// NM_001161577 // SAMD4A /// EN	SAMD4A	chr14	0.00167756	-1.56681
3537769	NM_002788 // PSMA3 /// NM_152132 // PSMA3 /// ENST00000216455 // PSMA3 /// ENST(	PSMA3	chr14	0.00662646	-1.50935
3541468	NM_152443 // RDH12 /// ENST00000267502 // RDH12 /// BC025724 // RDH12	RDH12	chr14	0.0267372	-1.53583
3542336	NM_001034852 // SMOC1 /// NM_022137 // SMOC1 /// ENST00000381280 // SMOC1 /// EN	SMOC1	chr14	0.0148603	-1.78077
3545586	NM_020421 // ADCK1 /// NM_001142545 // ADCK1 /// ENST00000341211 // ADCK1 /// EN	ADCK1	chr14	0.0387655	-1.545
3548815	NM_017437 // CPSF2 /// ENST00000298875 // CPSF2 /// BC070095 // CPSF2 /// ENST0(	CPSF2	chr14	0.0084703	-1.56256
3549594	NM_001130080 // IFI27 /// NM_005532 // IFI27 /// ENST00000298902 // IFI27 /// BC	IFI27	chr14	0.0445053	-1.778
3550097	NM_016417 // GLRX5 /// ENST00000331334 // GLRX5 /// BC023528 // GLRX5	GLRX5	chr14	0.0022557	-1.58231
3551459	NM_006668 // CYP46A1 /// ENST00000261835 // CYP46A1 /// BC022539 // CYP46A1	CYP46A1	chr14	0.00794992	-2.13217
3551517	NM_001008707 // EML1 /// NM_004434 // EML1 /// ENST00000262233 // EML1 /// ENST(	EML1	chr14	0.0148593	-1.52971
3551991	NM_024515 // WDR25 /// NM_001161476 // WDR25 /// ENST00000402312 // WDR25 /// EN	WDR25	chr14	0.0392976	-1.71332
3552089	NM_003836 // DLK1 /// ENST00000341267 // DLK1 /// AK289384 // DLK1 /// ENST0000(	DLK1	chr14	0.0254511	-1.62143
3554134	NM_015656 // KIF26A /// ENST00000423312 // KIF26A /// BC009415 // KIF26A /// ENS	KIF26A	chr14	0.0168482	-1.6051
3554327	NM_022489 // INF2 /// NM_001031714 // INF2 /// ENST00000392634 // INF2 /// ENST(	INF2	chr14	0.00372849	-1.86065
3554339	NM_022489 // INF2 /// NM_001031714 // INF2 /// ENST00000392634 // INF2 /// ENST(	INF2	chr14	0.00217327	-2.00013
3554877	NM_001134875 // C14orf80 /// NM_001134876 // C14orf80 /// NM_001134877 // C14or	C14orf80	chr14	0.0264714	-1.92359
3555905	NM_031314 // HNRNPC /// NM_001077442 // HNRNPC /// ENST00000336053 // HNRNPC //.	HNRNPC	chr14	0.0453118	-1.62938
3556046	---	0	chr14	0.00988487	-1.60752
3556366	NM_007192 // SUPT16H /// ENST00000216297 // SUPT16H	SUPT16H	chr14	0.0149939	-1.66328
3556707	---	0	chr14	0.000811794	-1.72679
3557226	NM_012244 // SLC7A8 /// NM_182728 // SLC7A8 /// ENST00000316902 // SLC7A8 /// EN	SLC7A8	chr14	0.0377144	-1.5316
3557286	NM_020834 // HOMEZ /// ENST00000357460 // HOMEZ /// AF463523 // HOMEZ	HOMEZ	chr14	0.00206002	-1.65585
3557424	NM_005864 // EFS /// ENST00000216733 // EFS /// AB001466 // EFS	EFS	chr14	0.00684444	-1.70128
3557466	NM_002471 // MYH6 /// ENST00000356287 // MYH6 /// ENST00000405093 // MYH6 /// D(	MYH6	chr14	0.00398369	-1.78985
3558501	NM_014178 // STXBP6 /// ENST00000419632 // STXBP6	STXBP6	chr14	0.0180784	-1.52928
3559232	NM_002742 // PRKD1 /// ENST00000331968 // PRKD1 /// X75756 // PRKD1 /// ENST000(	PRKD1	chr14	0.0177171	-1.61657
3559649	NM_015382 // HECTD1 /// ENST00000399332 // HECTD1 /// AY254380 // HECTD1 /// ENS	HECTD1	chr14	0.00930159	-1.58856
3561730	NM_004496 // FOXA1 /// ENST00000250448 // FOXA1 /// BC033890 // FOXA1	FOXA1	chr14	0.000177665	-2.16142
3564007	NM_198794 // MAP4K5 /// ENST00000013125 // MAP4K5 /// BC036013 // MAP4K5	MAP4K5	chr14	0.0466068	-1.69322
3568653	NM_198686 // RAB15 /// ENST00000267512 // RAB15 /// BC040679 // RAB15 /// ENST0(	RAB15	chr14	0.038287	-1.55538
3569486	NM_015346 // ZFYVE26 /// ENST00000347230 // ZFYVE26 /// ENST00000411699 // ZFYVE	ZFYVE26	chr14	0.0104988	-2.04816
3569849	NM_001130004 // ACTN1 /// NM_001102 // ACTN1 /// NM_001130005 // ACTN1 /// ENST(	ACTN1	chr14	0.00273329	-1.68934
3569953	NM_003861 // DCAF5 /// ENST00000341516 // DCAF5 /// BC136632 // DCAF5 /// ENST0(	DCAF5	chr14	0.0141234	-1.57716
3570269	NM_003049 // SLC10A1 /// ENST00000216540 // SLC10A1 /// BC069822 // SLC10A1	SLC10A1	chr14	0.0450208	-1.67966
3570416	NM_033262 // SLC8A3 /// NM_058240 // SLC8A3 /// NM_183002 // SLC8A3 /// NM_1829:	SLC8A3	chr14	0.0402792	-1.50021
3570641	NM_033141 // MAP3K9 /// ENST00000005198 // MAP3K9 /// AY327900 // MAP3K9 /// ENS	MAP3K9	chr14	0.0417411	-1.67357
3571951	NM_000428 // LTBP2 /// ENST00000261978 // LTBP2 /// BC078659 // LTBP2	LTBP2	chr14	0.0169972	-1.62114
3572025	NM_000428 // LTBP2 /// ENST00000261978 // LTBP2 /// BC078659 // LTBP2	LTBP2	chr14	0.00602685	-1.89516
3572289	NM_033116 // NEK9 /// ENST00000238616 // NEK9 /// AK128693 // NEK9	NEK9	chr14	7.63264E-05	-1.75906
3577698	NM_175739 // SERPINA9 /// NM_001042518 // SERPINA9 /// ENST00000337425 // SERPIN	SERPINA9	chr14	0.00052877	-1.55125
3577871	NM_177438 // DICER1 /// NM_030621 // DICER1 /// ENST00000343455 // DICER1 /// EN	DICER1	chr14	0.0428466	-1.50092
3580852	NM_001100119 // XRCC3 /// NM_005432 // XRCC3 /// NM_001100118 // XRCC3 /// ENST(	XRCC3	chr14	0.0119053	-1.59666
3581227	NM_138420 // AHNAK2 /// ENST00000333244 // AHNAK2 /// BC090889 // AHNAK2	AHNAK2	chr14	0.00636581	-1.51484
3581458	NM_002226 // JAG2 /// NM_145159 // JAG2 /// ENST00000331782 // JAG2 /// ENST000(	JAG2	chr14	0.0374717	-1.61726
3581650	BC065733 // IGH@ /// BX538077 // IGHA2 /// BC073765 // IGHA2 /// BX640625 // IGH	IGH@	chr14	0.007977	-1.57536
3581895	BC063384 // IGHD /// BC021276 // IGHD /// AK126280 // IGHD	IGHD	chr14	0.013279	-1.70774

3584471	ENST00000401453 // SNURF	SNURF	chr15	0.021165	-1.56419
3587017	NM_015995 // KLF13 /// ENST00000307145 // KLF13 /// AF132599 // KLF13	KLF13	chr15	0.0378748	-1.51836
3588131	NM_175741 // C15orf55 /// ENST00000333756 // C15orf55 /// AF482429 // C15orf55 /	C15orf55	chr15	0.0234904	-1.70798
3589912	NM_002225 // IVD /// NM_001159508 // IVD /// M34192 // IVD /// ENST00000249760 /	IVD	chr15	0.00737777	-1.57874
3590149	NM_001077268 // ZFYVE19 /// ENST00000355341 // ZFYVE19 /// ENST00000336455 // ZF	ZFYVE19	chr15	0.0436897	-1.6445
3590508	NM_006293 // TYRO3 /// ENST00000263798 // TYRO3 /// D17517 // TYRO3	TYRO3	chr15	0.016832	-1.75836
3590710	NR_015346 // JMJD7-PLA2G4B /// NM_005090 // JMJD7-PLA2G4B /// NM_001114632 // JM	JMJD7-PLA2G4B	chr15	0.0051326	-1.67739
3590976	NR_027911 // CAPN3 /// NR_027912 // CAPN3 /// NM_000070 // CAPN3 /// NM_024344 /	CAPN3	chr15	0.0197658	-1.70532
3592407	NM_197955 // C15orf48 /// NM_032413 // C15orf48 /// ENST00000344300 // C15orf48	C15orf48	chr15	0.0454212	-1.53651
3592467	NR_022014 // C15orf21 /// AY271965 // C15orf21 /// AY271964 // C15orf21 /// AY27	C15orf21	chr15	0.0498246	-1.54467
3594866	NM_004855 // PIGB /// ENST00000164305 // PIGB /// BC017711 // PIGB	PIGB	chr15	0.03555	-1.71324
3595443	NM_001018090 // GCOM1 /// NM_001018100 // GCOM1 /// NM_152451 // GCOM1 /// NM_00	GCOM1	chr15	0.0147697	-1.96973
3597176	NM_015059 // TLN2 /// ENST00000306829 // TLN2 /// AF402000 // TLN2	TLN2	chr15	0.0235419	-2.15536
3597376	NM_001018005 // TPM1 /// NM_001018007 // TPM1 /// NM_001018004 // TPM1 /// NM_00	TPM1	chr15	0.0415883	-1.54027
3598211	NM_182703 // ANKDD1A /// BC101274 // ANKDD1A /// ENST00000319597 // PLEKHO2 ///	ANKDD1A	chr15	0.00590321	-1.87358
3599210	NM_145160 // MAP2K5 /// NM_002757 // MAP2K5 /// ENST00000395476 // MAP2K5 /// EN	MAP2K5	chr15	0.0283791	-1.5001
3599586	NM_145658 // SPESP1 /// NM_024505 // NOX5 /// ENST00000260364 // NOX5 /// AY3589	SPESP1	chr15	0.0363887	-1.55538
3601230	NM_001024736 // CD276 /// NM_025240 // CD276 /// ENST00000318443 // CD276 /// EN	CD276	chr15	0.0195841	-1.70976
3601420	NM_033250 // PML /// NM_033240 // PML /// NM_033244 // PML /// NM_033239 // PML	PML	chr15	0.00114677	-1.78289
3602124	NM_015492 // C15orf39 /// ENST00000360639 // C15orf39 /// ENST00000394987 // C15	C15orf39	chr15	0.0293085	-1.88585
3602645	NM_145805 // ISL2 /// ENST00000290759 // ISL2 /// BC012136 // ISL2	ISL2	chr15	0.0399417	-1.66435
3602727	NM_002902 // RCN2 /// ENST00000320963 // RCN2 /// BC004892 // RCN2 /// ENST00000	RCN2	chr15	0.0008754	-1.76833
3608277	NM_022769 // CRTC3 /// NM_001042574 // CRTC3 /// ENST00000268184 // CRTC3 /// EN	CRTC3	chr15	0.0132435	-1.94296
3614406	AB051358 // ATP10A	ATP10A	chr15	0.00953363	-1.59393
3614984	NM_004667 // HERC2 /// ENST00000261609 // HERC2 /// AF071172 // HERC2	HERC2	chr15	0.0174051	-2.41978
3616237	NM_130901 // OTUD7A /// ENST00000307050 // OTUD7A /// ENST00000382902 // OTUD7A	OTUD7A	chr15	0.00925103	-2.09875
3619258	NM_001003940 // BMF /// ENST00000354670 // BMF /// BC070043 // BMF	BMF	chr15	0.0151119	-1.5891
3619346	NM_004573 // PLCB2 /// ENST00000260402 // PLCB2 /// M95678 // PLCB2 /// ENST0000	PLCB2	chr15	0.0132795	-1.55249
3619692	NM_017726 // PPP1R14D /// NM_001130143 // PPP1R14D /// ENST00000299174 // PPP1R1	PPP1R14D	chr15	0.0332753	-1.90245
3619838	NM_017553 // INO80 /// ENST00000361937 // INO80 /// ENST00000401393 // INO80 //	INO80	chr15	0.0088689	-1.61017
3620055	NM_015540 // RPAP1 /// ENST00000304330 // RPAP1 /// AB037824 // RPAP1	RPAP1	chr15	0.0205252	-1.56852
3620161	NM_016642 // SPTBN5 /// ENST00000320955 // SPTBN5 /// AF233523 // SPTBN5	SPTBN5	chr15	0.0312593	-1.51343
3620384	NM_178034 // PLA2G4D /// ENST00000290472 // PLA2G4D /// AB090876 // PLA2G4D	PLA2G4D	chr15	0.0166677	-2.32415
3620755	NM_138477 // CDAN1 /// ENST00000356231 // CDAN1 /// BC066640 // CDAN1 /// ENST00	CDAN1	chr15	0.0134417	-1.96639
3620779	NM_138477 // CDAN1 /// ENST00000356231 // CDAN1 /// BC066640 // CDAN1 /// ENST00	CDAN1	chr15	0.00688249	-2.06309
3621643	NM_025165 // ELL3 /// ENST00000319359 // ELL3 /// AK126384 // ELL3 /// ENST00000	ELL3	chr15	0.00251378	-1.83761
3621719	NM_005926 // MFAP1 /// ENST00000267812 // MFAP1 /// BC050742 // MFAP1	MFAP1	chr15	0.0168999	-2.02478
3622228	NM_014080 // DUOX2 /// ENST00000389039 // DUOX2 /// ENST00000267837 // DUOX2 //	DUOX2	chr15	0.0492047	-1.56715
3623557	NM_024837 // ATP8B4 /// ENST00000284509 // ATP8B4 /// AB075819 // ATP8B4	ATP8B4	chr15	0.00481284	-1.50316
3624470	NM_006578 // GNB5 /// NM_016194 // GNB5 /// ENST00000261837 // GNB5 /// ENST0000	GNB5	chr15	0.00507242	-1.51614
3624704	NM_006628 // ARPP19 /// ENST00000249822 // ARPP19 /// AL833077 // ARPP19	ARPP19	chr15	0.0417217	-1.65286
3625621	NM_006154 // NEDD4 /// ENST00000435532 // NEDD4 /// ENST00000164316 // NEDD4	NEDD4	chr15	0.0465026	-1.76332
3630187	NM_000968 // RPL4 /// ENST00000307961 // RPL4 /// AK125772 // RPL4 /// ENST00000	RPL4	chr15	0.044709	-2.49299
3630245	NM_207338 // LCTL /// ENST00000341509 // LCTL /// AY358729 // LCTL	LCTL	chr15	0.00109927	-1.5048
3630747	NM_001004439 // ITGA11 /// ENST00000315757 // ITGA11 /// AF109681 // ITGA11 ///	ITGA11	chr15	0.0316173	-2.59387
3631221	NM_005078 // TLE3 /// NM_001105192 // TLE3 /// NM_020908 // TLE3 /// ENST0000031	TLE3	chr15	0.033387	-2.16688
3632042	NM_020214 // PARP6 /// ENST00000287196 // PARP6 /// BC110902 // PARP6 /// ENST00	PARP6	chr15	0.0149134	-1.98349
3632434	NM_005477 // HCN4 /// ENST00000261917 // HCN4	HCN4	chr15	0.0480479	-1.6158
3632876	NM_000781 // CYP11A1 /// NM_001099773 // CYP11A1 /// ENST00000268053 // CYP11A1	CYP11A1	chr15	0.00729867	-1.5285
3633584	NM_001897 // CSPG4 /// ENST00000308508 // CSPG4 /// AY359468 // CSPG4	CSPG4	chr15	0.0423272	-2.01214
3634872	BC040275 // RASGRF1	RASGRF1	chr15	0.0285656	-1.81052

3638379	NM_002693 // POLG /// NM_001126131 // POLG /// ENST00000268124 // POLG /// ENST(	POLG	chr15	0.0418698	-1.58315
3638509	NM_198525 // KIF7 /// ENST00000394412 // KIF7 /// AY358384 // KIF7 /// ENST0000(	KIF7	chr15	0.0071197	-1.75381
3639075	NM_018668 // VPS33B /// ENST00000333371 // VPS33B /// AF308803 // VPS33E	VPS33B	chr15	0.0375612	-1.78117
3641584	NM_152449 // LYSMD4 /// ENST00000344791 // LYSMD4 /// BC041097 // LYSMD4 /// ENS	LYSMD4	chr15	0.00113035	-1.84191
3642374	NM_078474 // TM2D3 /// NM_025141 // TM2D3 /// ENST00000333202 // TM2D3 /// ENST(	TM2D3	chr15	0.00451076	-1.56399
3643377	NM_207112 // HAGHL /// NM_032304 // HAGHL /// ENST00000341413 // HAGHL /// ENST(	HAGHL	chr16	0.0220833	-2.73131
3643404	NM_005823 // MSLN /// NM_013404 // MSLN /// NM_001177355 // MSLN /// ENST000003ɛ	MSLN	chr16	0.00232549	-1.82199
3644269	NM_006453 // TBL3 /// ENST00000332704 // TBL3 /// BC010231 // TBL3	TBL3	chr16	0.0330334	-1.65837
3644582	NM_032271 // TRAF7 /// ENST00000326181 // TRAF7 /// AY569455 // TRAF7	TRAF7	chr16	0.00699782	-1.93549
3645303	NM_016333 // SRRM2 /// ENST00000301740 // SRRM2 /// AB016092 // SRRM2 /// ENST0(	SRRM2	chr16	0.00031979	-2.30865
3646192	NM_005147 // DNAJA3 /// NM_001135110 // DNAJA3 /// ENST00000262375 // DNAJA3 //.	DNAJA3	chr16	0.00889876	-1.53488
3646509	NM_014692 // SEC14L5 /// ENST00000251170 // SEC14L5 /// AB007880 // SEC14L5	SEC14L5	chr16	0.0235476	-1.85763
3648026	NM_000246 // CIITA /// AF410154 // CIITA	CIITA	chr16	0.00622238	-1.51602
3652387	NM_173615 // VWA3A /// ENST00000389398 // VWA3A /// ENST00000299840 // VWA3A ///	VWA3A	chr16	0.010176	-1.87723
3652524	NM_018119 // POLR3E /// ENST00000299853 // POLR3E /// ENST00000418581 // POLR3E	POLR3E	chr16	0.0390534	-1.75207
3653549	NM_052944 // SLC5A11 /// ENST00000347898 // SLC5A11 /// AY044906 // SLC5A11 ///	SLC5A11	chr16	0.0375094	-2.48992
3655834	NM_004783 // TAOK2 /// NM_016151 // TAOK2 /// ENST00000308893 // TAOK2 /// ENST(	TAOK2	chr16	0.0105739	-1.77626
3656269	NM_002209 // ITGAL /// NM_001114380 // ITGAL /// ENST00000358164 // ITGAL /// EN	ITGAL	chr16	0.0134932	-1.62746
3656584	NM_014771 // RNF40 /// ENST00000324685 // RNF40 /// AF122819 // RNF40 /// ENST0(	RNF40	chr16	0.0416243	-1.72826
3657018	NM_001145808 // ITGAM /// NM_000632 // ITGAM /// ENST00000287497 // ITGAM /// J(	ITGAM	chr16	0.0179266	-1.61529
3657063	NM_000887 // ITGAX /// ENST00000268296 // ITGAX /// M81695 // ITGAX /// ENST000(	ITGAX	chr16	0.00209266	-1.96552
3657235	NM_003041 // SLC5A2 /// ENST00000330498 // SLC5A2 /// M95549 // SLC5A2 /// ENST(	SLC5A2	chr16	0.00198943	-1.94986
3658989	NM_133443 // GPT2 /// ENST00000340124 // GPT2 /// AY029173 // GPT2	GPT2	chr16	0.0393738	-1.5723
3662867	NM_170776 // GPR97 /// ENST00000333493 // GPR97 /// BC064508 // GPR97 /// ENST0(	GPR97	chr16	0.0356772	-1.5834
3663089	NM_002428 // MMP15 /// ENST00000219271 // MMP15 /// BC036495 // MMP15	MMP15	chr16	0.03528	-1.51028
3663187	NM_014157 // CCDC113 /// NM_001142302 // CCDC113 /// ENST00000219299 // CCDC113	CCDC113	chr16	0.0282533	-1.59106
3663365	NM_001160305 // SETD6 /// NM_024860 // SETD6 /// ENST00000219315 // SETD6 /// EN	SETD6	chr16	0.00140451	-2.33325
3664834	NM_181269 // CMTM1 /// NM_181296 // CMTM1 /// NM_052999 // CMTM1 /// NM_181268 .	CMTM1	chr16	0.00574475	-1.52667
3665308	NM_001950 // E2F4 /// ENST00000379378 // E2F4 /// BC033180 // E2F4	E2F4	chr16	0.0331803	-1.60431
3665338	NM_024712 // ELMO3 /// ENST00000393997 // ELMO3 /// AK300976 // ELMO3 /// ENST0(	ELMO3	chr16	0.00819199	-1.94064
3666207	NM_019023 // PRMT7 /// ENST00000449359 // PRMT7 /// ENST00000339507 // PRMT7 //.	PRMT7	chr16	0.0100312	-2.35811
3667828	NM_001361 // DHODH /// ENST00000219240 // DHODH /// BC065245 // DHODH	DHODH	chr16	0.00325835	-1.61215
3668876	NM_032268 // ZNRF1 /// ENST00000335325 // ZNRF1 /// ENST00000320619 // ZNRF1 //.	ZNRF1	chr16	0.0407697	-1.67595
3671553	NM_019065 // NECAB2 /// ENST00000305202 // NECAB2 /// AY299331 // NECAB2	NECAB2	chr16	0.0205573	-1.61641
3671617	NM_178452 // LRRC50 /// ENST00000378553 // LRRC50 /// ENST00000439133 // LRRC50	LRRC50	chr16	0.0138849	-1.58373
3671969	NM_031476 // CRISPLD2 /// ENST00000262424 // CRISPLD2 /// AY358790 // CRISPLD2 .	CRISPLD2	chr16	0.0133607	-1.64504
3673469	NM_153813 // ZFPM1 /// ENST00000319555 // ZFPM1 /// AF488691 // ZFPM1	ZFPM1	chr16	0.0390064	-1.61055
3673589	NM_013278 // IL17C /// ENST00000244241 // IL17C /// AY358471 // IL17C	IL17C	chr16	0.0428981	-1.71931
3673671	ENST00000333666 // FLJ40448 /// AK097767 // FLJ40448	FLJ40448	chr16	0.0261743	-1.50689
3674131	NM_003119 // SPG7 /// ENST00000268704 // SPG7 /// BC036104 // SPG7	SPG7	chr16	0.00400311	-1.64644
3674330	NM_052988 // CDK10 /// NM_052987 // CDK10 /// NM_001098533 // CDK10 /// NM_0011ɛ	CDK10	chr16	0.0260159	-1.54842
3674331	NM_052988 // CDK10 /// NM_052987 // CDK10 /// NM_001098533 // CDK10 /// NM_0011ɛ	CDK10	chr16	0.0380113	-1.52393
3675040	NM_183337 // RGS11 /// NM_003834 // RGS11 /// ENST00000397770 // RGS11 /// ENST(	RGS11	chr16	0.0277963	-1.52714
3675351	NM_001031737 // CCDC78 /// ENST00000293889 // CCDC78 /// ENST00000439619 // CCDC	CCDC78	chr16	0.0174622	-1.69963
3675396	NM_022493 // NARFL /// ENST00000251588 // NARFL /// ENST00000301694 // NARFL	NARFL	chr16	0.0032572	-1.9644
3675881	NM_001037125 // UNKL /// ENST00000301712 // UNKL /// AM944365 // UNKL /// ENST0(	UNKL	chr16	0.0282927	-1.56896
3675976	NM_001287 // CLCN7 /// NM_001114331 // CLCN7 /// ENST00000428756 // CLCN7 /// BC	CLCN7	chr16	0.0470619	-1.86318
3676151	NM_080861 // SPSB3 /// ENST00000301717 // SPSB3 /// BC065515 // SPSB3	SPSB3	chr16	0.00205965	-1.69691
3676568	NM_020764 // CASKIN1 /// ENST00000343516 // CASKIN1 /// AF451977 // CASKIN1 ///	CASKIN1	chr16	0.00657603	-1.50433
3676580	NM_020764 // CASKIN1 /// ENST00000343516 // CASKIN1 /// AF451977 // CASKIN1 ///	CASKIN1	chr16	0.0216538	-1.74792
3676666	NM_001919 // DCI /// NM_001178029 // DCI /// ENST00000301729 // DCI /// AK291127	DCI	chr16	0.0345441	-1.50151

3676685	NM_006711 // RNPS1 /// NM_080594 // RNPS1 /// ENST00000320225 // RNPS1 /// ENST0	RNPS1	chr16	0.041137	-1.544
3676719	---	0	chr16	0.0258165	-1.50489
3676737	---	0	chr16	0.0147275	-2.18269
3676747	---	0	chr16	0.0308592	-1.52339
3677286	NM_022119 // PRSS22 /// ENST00000161006 // PRSS22 /// AB010779 // PRSS22	PRSS22	chr16	0.00100276	-1.68138
3677758	NM_016292 // TRAP1 /// ENST00000246957 // TRAP1 /// AF154108 // TRAP1 /// BX6482	TRAP1	chr16	0.0365369	-1.57838
3677800	NM_004380 // CREBBP /// NM_001079846 // CREBBP /// ENST00000382070 // CREBBP //.	CREBBP	chr16	0.028317	-1.90778
3677956	NM_001116 // ADCY9 /// ENST00000294016 // ADCY9 /// BC151229 // ADCY9	ADCY9	chr16	0.0136798	-1.58738
3677957	NM_001116 // ADCY9 /// ENST00000294016 // ADCY9 /// BC151229 // ADCY9	ADCY9	chr16	0.0272678	-1.60522
3678120	NM_024535 // CORO7 /// ENST00000251166 // CORO7 /// AL833954 // CORO7 /// ENST00	CORO7	chr16	0.041952	-1.66064
3678173	ENST00000404295 // NMRAL1	NMRAL1	chr16	0.0369365	-1.54004
3678525	NM_016256 // NAGPA /// ENST00000312251 // NAGPA /// BC012194 // NAGPA /// ENST00	NAGPA	chr16	0.0493565	-1.7757
3680603	NM_015659 // RSL1D1 /// ENST00000396503 // RSL1D1 /// AY154473 // RSL1D1 /// ENS	RSL1D1	chr16	0.0321514	-1.57037
3680998	NM_018340 // CPPED1 /// ENST00000261660 // CPPED1 /// ENST00000381774 // CPPED1	CPPED1	chr16	0.0164511	-1.9323
3682203	NM_001171 // ABCC6 /// ENST00000205557 // ABCC6 /// AF076622 // ABCC6 /// ENST00	ABCC6	chr16	0.0487262	-1.75416
3682495	NM_022166 // XYLT1 /// ENST00000261381 // XYLT1	XYLT1	chr16	0.0116729	-2.17594
3685380	NM_033266 // ERN2 /// ENST00000256797 // ERN2 /// EF560739 // ERN2 /// ENST00000	ERN2	chr16	0.0290504	-1.71109
3685408	NM_033266 // ERN2 /// ENST00000256797 // ERN2 /// EF560739 // ERN2 /// ENST00000	ERN2	chr16	0.0132645	-2.19261
3687485	NM_024307 // GDPD3 /// ENST00000406256 // GDPD3 /// ENST00000360688 // GDPD3 //.	GDPD3	chr16	0.0114956	-1.59483
3693411	NM_001297 // CNGB1 /// ENST00000251102 // CNGB1 /// AF042498 // CNGB1 /// ENST00	CNGB1	chr16	0.029644	-1.52488
3693422	NM_001297 // CNGB1 /// ENST00000251102 // CNGB1 /// AF042498 // CNGB1 /// ENST00	CNGB1	chr16	0.0110029	-1.51536
3693611	NM_001080492 // PRSS54 /// ENST00000219301 // KLKBL4 /// BC057843 // PRSS54	PRSS54	chr16	0.00492649	-1.52704
3699433	NM_001170714 // BCAR1 /// NM_001170715 // BCAR1 /// NM_001170716 // BCAR1 /// NM	BCAR1	chr16	0.0273715	-1.60551
3699848	---	0	chr16	0.0257175	-1.6411
3700180	NM_199355 // ADAMTS18 /// ENST00000282849 // ADAMTS18 /// BC063283 // ADAMTS18 /	ADAMTS18	chr16	0.0148099	-1.51797
3701549	NM_052892 // PKD1L2 /// NM_001076780 // PKD1L2 /// ENST00000299598 // PKD1L2 //.	PKD1L2	chr16	0.0312837	-1.81683
3702440	NM_172347 // KCNG4 /// ENST00000308251 // KCNG4 /// BC008969 // KCNG4 /// ENST00	KCNG4	chr16	0.0368976	-1.62379
3702509	NM_020947 // KIAA1609 /// ENST00000343629 // KIAA1609 /// AB046829 // KIAA1609	KIAA1609	chr16	0.00794142	-1.82219
3703117	NM_016095 // GINS2 /// ENST00000253462 // GINS2 /// AK091519 // GINS2	GINS2	chr16	0.0383035	-1.5372
3703814	NM_017566 // KLHDC4 /// ENST00000270583 // KLHDC4 /// BC022969 // KLHDC4 /// ENS	KLHDC4	chr16	0.0162115	-1.89021
3704447	NM_001142864 // FAM38A /// D87071 // FAM38A /// ENST00000451779 // FAM38A /// EN	FAM38A	chr16	0.0203387	-1.66227
3705027	NM_000135 // FANCA /// ENST00000389301 // FANCA /// ENST00000305699 // FANCA //.	FANCA	chr16	0.00456305	-1.67174
3705502	NM_024792 // FAM57A /// ENST00000308278 // FAM57A /// ENST00000301324 // FAM57A	FAM57A	chr17	0.0414645	-1.50244
3706309	NM_014853 // SGSM2 /// NM_001098509 // SGSM2 /// ENST00000426855 // SGSM2 /// EN	SGSM2	chr17	0.00868961	-1.65557
3707283	NM_015716 // MINK1 /// NM_170663 // MINK1 /// NM_153827 // MINK1 /// NM_00102493	MINK1	chr17	0.0306465	-1.50268
3707356	NM_015528 // RNF167 /// ENST00000262482 // RNF167	RNF167	chr17	0.0020777	-1.6608
3707470	NM_006612 // KIF1C /// ENST00000320785 // KIF1C /// BC034993 // KIF1C	KIF1C	chr17	0.0387942	-1.53591
3708224	NM_181844 // BCL6B /// ENST00000293805 // BCL6B /// BC059404 // BCL6B	BCL6B	chr17	0.0353573	-1.59533
3708321	NM_000018 // ACADVL /// NM_001033859 // ACADVL /// ENST00000350303 // ACADVL //.	ACADVL	chr17	0.0164202	-2.00644
3708407	NM_001042 // SLC2A4 /// ENST00000317370 // SLC2A4 /// BC034387 // SLC2A4 /// ENS	SLC2A4	chr17	0.0109182	-1.67497
3708512	NM_001002914 // KCTD11 /// ENST00000333751 // KCTD11 /// AY646650 // KCTD11	KCTD11	chr17	0.00736309	-1.59617
3708675	NM_000747 // CHRNB1 /// ENST00000306071 // CHRNB1 /// BC023553 // CHRNB1	CHRNB1	chr17	0.031595	-1.5751
3708922	NM_001040 // SHBG /// NM_001146279 // SHBG /// NM_001146280 // SHBG /// NM_00114	SHBG	chr17	0.0176521	-1.74705
3708995	NM_001406 // EFNB3 /// ENST00000226091 // EFNB3 /// U66406 // EFNB3	EFNB3	chr17	0.00333337	-1.84933
3709342	NM_053051 // CNTROB /// NM_001037144 // CNTROB /// ENST00000420934 // CNTROB ///	CNTROB	chr17	0.0223922	-1.61639
3709543	NM_012393 // PFAS /// ENST00000314666 // PFAS /// BC146768 // PFAS	PFAS	chr17	0.0161368	-1.54596
3709658	NM_153007 // ODF4 /// ENST00000328248 // ODF4 /// AY237799 // ODF4	ODF4	chr17	0.0021151	-1.57743
3710040	NM_145054 // WDR16 /// NM_001080556 // WDR16 /// ENST00000352665 // WDR16 /// EN	WDR16	chr17	0.0258811	-1.76348
3711900	NM_017775 // TTC19 /// ENST00000261647 // TTC19 /// ENST00000395886 // TTC19 //.	TTC19	chr17	0.00084782	-2.27135
3712092	NM_016113 // TRPV2 /// ENST00000338560 // TRPV2 /// BC018926 // TRPV2	TRPV2	chr17	0.0313361	-1.54317
3712975	NM_001388 // DRG2 /// ENST00000225729 // DRG2 /// AB209340 // DRG2 /// ENST00000	DRG2	chr17	0.00893159	-1.55249

3715571	NM_015077 // SARM1 /// AY444166 // SARM1 /// ENST00000412574 // TMEM199 /// ENST	SARM1	chr17	0.0459852	-1.62872
3715951	NM_016518 // PIPOX /// ENST00000323372 // PIPOX /// BC008960 // PIPOX	PIPOX	chr17	0.0152328	-1.67188
3717413	NM_015355 // SUZ12 /// ENST00000322652 // SUZ12 /// AK290014 // SUZ12	SUZ12	chr17	0.0160269	-1.70269
3717914	NM_173847 // SPACA3 /// ENST00000269053 // SPACA3 /// AY358653 // SPACA3 /// ENS	SPACA3	chr17	0.0163062	-2.26646
3720121	NM_000981 // RPL19 /// ENST00000225430 // RPL19 /// BC095445 // RPL19	RPL19	chr17	0.0232753	-1.71199
3720425	NM_004448 // ERBB2 /// NM_001005862 // ERBB2 /// ENST00000269571 // ERBB2 /// EN	ERBB2	chr17	0.00670719	-2.00116
3720905	NM_001254 // CDC6 /// ENST00000209728 // CDC6 /// BC025232 // CDC6	CDC6	chr17	0.00849046	-1.50506
3722004	NM_003632 // CNTNAP1 /// ENST00000264638 // CNTNAP1 /// U87223 // CNTNAP1	CNTNAP1	chr17	0.00828594	-1.83042
3722062	NM_032353 // VPS25 /// ENST00000253794 // VPS25 /// BC006282 // VPS25	VPS25	chr17	0.0227958	-1.65292
3725785	NM_007067 // MYST2 /// ENST00000259021 // MYST2 /// AF140360 // MYST2 /// AF2175	MYST2	chr17	0.00187751	-2.01781
3725982	---	0	chr17	0.0405562	-1.60961
3726166	NM_002204 // ITGA3 /// NM_005501 // ITGA3 /// ENST00000320031 // ITGA3 /// ENST0	ITGA3	chr17	0.025148	-1.52001
3726476	NM_018346 // RSAD1 /// ENST00000258955 // RSAD1 /// BC050538 // RSAD1 /// ENST00	RSAD1	chr17	0.00348046	-1.83163
3726554	NM_017957 // EPN3 /// ENST00000268933 // EPN3 /// AK292977 // EPN3 /// ENST00000	EPN3	chr17	0.000457233	-1.982
3726744	NM_003786 // ABCC3 /// ENST00000285238 // ABCC3 /// AF085692 // ABCC3 /// ENST00	ABCC3	chr17	0.047285	-1.6421
3726827	NM_175575 // WFIKKN2 /// ENST00000311378 // WFIKKN2 /// AY358142 // WFIKKN2	WFIKKN2	chr17	0.0133769	-1.8418
3727347	NM_032559 // KIF2B /// AF333335 // KIF2B	KIF2B	chr17	0.0149574	-1.75544
3728600	NM_000502 // EPX /// ENST00000225371 // EPX	EPX	chr17	0.00115381	-1.61402
3728899	NM_030938 // TMEM49 /// ENST00000262291 // TMEM49 /// BC009758 // TMEM49	TMEM49	chr17	0.019014	-1.65147
3729570	NM_001099432 // BCAS3 /// NM_017679 // BCAS3 /// ENST00000405070 // BCAS3 /// EN	BCAS3	chr17	0.013826	-1.71042
3729614	NM_001099432 // BCAS3 /// NM_017679 // BCAS3 /// ENST00000390652 // BCAS3 /// EN	BCAS3	chr17	0.00431258	-2.69101
3730003	NM_203425 // C17orf82 /// BC046200 // C17orf82 /// ENST00000335108 // C17orf82	C17orf82	chr17	0.0102988	-1.84958
3730629	NM_152830 // ACE /// NM_001178057 // ACE /// ENST00000290863 // ACE /// ENST0000	ACE	chr17	0.012234	-1.53074
3730785	NM_016360 // TACO1 /// ENST00000258975 // TACO1 /// AK094052 // TACO1	TACO1	chr17	0.00471444	-1.50304
3733961	NM_018714 // COG1 /// ENST00000299886 // COG1 /// ENST00000438720 // COG1 /// AB	COG1	chr17	0.0119373	-1.77579
3734238	NM_032646 // TTYH2 /// ENST00000269346 // TTYH2 /// AF319952 // TTYH2	TTYH2	chr17	0.0226309	-1.75616
3735135	NM_013260 // SAP30BP /// ENST00000355423 // SAP30BP /// BC030233 // SAP30BP ///	SAP30BP	chr17	0.0305267	-1.51103
3735207	NM_000213 // ITGB4 /// NM_001005619 // ITGB4 /// NM_001005731 // ITGB4 /// ENST0	ITGB4	chr17	0.00949144	-1.56108
3735448	NM_182565 // FAM100B /// ENST00000327490 // FAM100B /// BC035511 // FAM100B	FAM100B	chr17	0.0193225	-1.69136
3735499	NM_021972 // SPHK1 /// NM_182965 // SPHK1 /// NM_001142601 // SPHK1 /// NM_0011	SPHK1	chr17	0.00643178	-1.77499
3736168	NM_152468 // TMC8 /// ENST00000318430 // TMC8 /// BC110296 // TMC8 /// ENST0000	TMC8	chr17	0.0304471	-1.65371
3737148	NM_000152 // GAA /// NM_001079803 // GAA /// NM_001079804 // GAA /// ENST000003	GAA	chr17	0.00629227	-1.80986
3737351	NM_020914 // RNF213 /// ENST00000336301 // RNF213 /// AK127358 // RNF213	RNF213	chr17	0.0200578	-1.52324
3738121	NM_199287 // CCDC137 /// ENST00000329214 // CCDC137 /// BC009369 // CCDC137	CCDC137	chr17	0.045991	-1.5645
3738150	NM_004712 // HGS /// ENST00000329138 // HGS /// U43895 // HGS /// ENST000004423	HGS	chr17	0.0175845	-1.57096
3738161	NM_004712 // HGS /// ENST00000329138 // HGS /// U43895 // HGS /// ENST000004423	HGS	chr17	0.0264213	-1.76679
3738235	NM_012140 // SLC25A10 /// ENST00000350690 // SLC25A10 /// BC015797 // SLC25A10	SLC25A10	chr17	0.0465024	-1.63942
3738497	NM_212492 // GPS1 /// ENST00000392358 // GPS1 /// ENST00000355130 // GPS1 /// EN	GPS1	chr17	0.0246374	-1.64928
3738929	NM_012336 // NARF /// NM_031968 // NARF /// NM_001038618 // NARF /// NM_0010836	NARF	chr17	0.00153659	-1.62911
3738950	NM_012336 // NARF /// NM_031968 // NARF /// NM_001038618 // NARF /// NM_0010836	NARF	chr17	0.0138052	-1.59156
3740210	NM_001080779 // MYO1C /// NM_001080950 // MYO1C /// NM_033375 // MYO1C /// ENST0	MYO1C	chr17	0.00535878	-1.55965
3741052	NM_020310 // MNT /// ENST00000174618 // MNT /// ENST00000404961 // MNT /// BC117	MNT	chr17	0.0288444	-1.56603
3741207	NM_015229 // KIAA0664 /// ENST00000435359 // KIAA0664 /// ENST00000322335 // KIA	KIAA0664	chr17	0.0349093	-1.75481
3741315	NM_003555 // OR1G1 /// ENST00000328890 // OR1G1 /// BC095520 // OR1G1 /// ENST00	OR1G1	chr17	0.0457756	-1.57814
3741736	NM_032294 // CAMKK1 /// NM_172206 // CAMKK1 /// NM_172207 // CAMKK1 /// ENST0000	CAMKK1	chr17	0.0177672	-1.50498
3741818	NM_005173 // ATP2A3 /// NM_174955 // ATP2A3 /// NM_174956 // ATP2A3 /// NM_17495	ATP2A3	chr17	0.0400948	-1.7768
3741891	NM_015113 // ZZEF1 /// ENST00000381638 // ZZEF1 /// BC151836 // ZZEF1 /// ENST00	ZZEF1	chr17	0.0172844	-1.86398
3742524	NM_001167986 // INCA1 /// NM_001167987 // INCA1 /// NM_213726 // INCA1 /// NM_00	INCA1	chr17	0.0207984	-1.52357
3743410	NM_001365 // DLG4 /// NM_001128827 // DLG4 /// ENST00000302955 // DLG4 /// ENST0	DLG4	chr17	0.0130397	-1.63733
3743443	NM_004422 // DVL2 /// ENST00000005340 // DVL2 /// BC014844 // DVL2	DVL2	chr17	0.0145106	-1.67578
3744192	NM_002616 // PER1 /// ENST00000317276 // PER1 /// AF022991 // PER1 /// ENST0000	PER1	chr17	0.0479777	-1.50474

3744274	NM_004217 // AURKB /// ENST00000316199 // AURKB /// BC013300 // AURKE	AURKB	chr17	0.00994115	-2.07165
3745158	NM_003802 // MYH13 /// ENST00000252172 // MYH13 /// AF111782 // MYH1:	MYH13	chr17	0.0465003	-1.89148
3745206	NM_002472 // MYH8 /// ENST00000252173 // MYH8 /// ENST00000403437 // MYH8 /// M:	MYH8	chr17	0.00543128	-1.89492
3746889	NM_006311 // NCOR1 /// ENST00000268712 // NCOR1 /// BC167431 // NCOR1 /// AF087ꞓ	NCOR1	chr17	0.00819533	-1.82707
3748203	NM_002018 // FLII /// ENST00000327031 // FLII /// BC025300 // FLII /// ENST0000C	FLII	chr17	0.012954	-1.60781
3749580	NM_015276 // USP22 /// ENST00000261497 // USP22 /// ENST00000455117 // USP22 //.	USP22	chr17	0.0372146	-1.53966
3749650	---	0	chr17	0.00522763	-2.01647
3749716	---	0	chr17	0.00869053	-1.50496
3749732	---	0	chr17	0.0315389	-1.55967
3750581	NR_028334 // FLJ40504 /// AK097823 // FLJ40504	FLJ40504	chr17	0.0436152	-1.59125
3750665	NM_000638 // VTN /// NM_001083896 // SEBOX /// ENST00000226218 // VTN /// BC005C	VTN	chr17	0.048787	-1.55567
3750795	NM_006461 // SPAG5 /// ENST00000321765 // SPAG5 /// AF399910 // SPAG5	SPAG5	chr17	0.00588401	-1.69999
3751358	NM_078471 // MYO18A /// NM_203318 // MYO18A /// ENST00000354329 // MYO18A /// EN	MYO18A	chr17	0.0476566	-1.51848
3751546	NM_001085454 // GIT1 /// NM_014030 // GIT1 /// ENST00000394869 // GIT1 /// ENSTC	GIT1	chr17	0.0346435	-1.68511
3753836	NM_152781 // C17orf66 /// ENST00000311880 // C17orf66 /// BC033734 // C17orf66 /	C17orf66	chr17	0.00366415	-1.5446
3754716	NM_007247 // SYNRG /// NM_080550 // SYNRG /// NM_198882 // SYNRG /// NM_0011635-	SYNRG	chr17	0.0175505	-1.53785
3755105	NM_001004334 // GPR179 /// ENST00000342292 // GPR179 /// AY584611 // GPR179 ///	GPR179	chr17	0.0378077	-1.7482
3755991	NM_014815 // MED24 /// NM_001079518 // MED24 /// ENST00000356271 // MED24 /// EN	MED24	chr17	0.00195114	-1.79751
3756064	NM_021724 // NR1D1 /// ENST00000246672 // NR1D1 /// M24898 // NR1D1	NR1D1	chr17	0.000765508	-1.57152
3757185	NM_005557 // KRT16 /// ENST00000301653 // KRT16 /// BC039169 // KRT16	KRT16	chr17	0.0291407	-1.6885
3757419	NR_033465 // NT5C3L /// ENST00000269534 // NT5C3L /// BC016971 // NT5C3L /// ENS	NT5C3L	chr17	0.0386202	-1.50126
3757616	NM_024119 // DHX58 /// ENST00000251642 // DHX58 /// BC014949 // DHX58 /// ENST0C	DHX58	chr17	0.00921254	-1.73662
3757741	NM_001524 // HCRT /// ENST00000293330 // HCRT /// AF041240 // HCRT	HCRT	chr17	0.0292048	-1.54434
3757875	NM_139276 // STAT3 /// NM_003150 // STAT3 /// NM_213662 // STAT3 /// ENST0000026	STAT3	chr17	0.00215528	-1.52388
3757931	NM_012232 // PTRF /// ENST00000357037 // PTRF /// BC066123 // PTRF /// ENST0000C	PTRF	chr17	0.0383352	-1.6167
3758031	NM_024927 // PLEKHH3 /// ENST00000293349 // PLEKHH3 /// BC052978 // PLEKHH3 ///	PLEKHH3	chr17	0.0385112	-2.36528
3758728	NM_005374 // MPP2 /// ENST00000269095 // MPP2 /// BC030287 // MPP2 /// ENST0000C	MPP2	chr17	0.00474795	-1.75734
3759170	NM_000419 // ITGA2B /// ENST00000262407 // ITGA2B /// J02764 // ITGA2B /// ENST0	ITGA2B	chr17	0.000988802	-1.62364
3759783	NM_199282 // ARHGAP27 /// NM_001159330 // ARHGAP27 /// BC101388 // ARHGAP27	ARHGAP27	chr17	0.0285141	-1.62455
3760958	NM_138355 // SCRN2 /// NM_001145023 // SCRN2 /// ENST00000407215 // SCRN2 /// EN	SCRN2	chr17	0.0260382	-1.82454
3760961	NM_138355 // SCRN2 /// NM_001145023 // SCRN2 /// ENST00000407215 // SCRN2 /// EN	SCRN2	chr17	0.0184735	-1.53618
3762156	NM_032595 // PPP1R9B /// ENST00000316878 // PPP1R9B	PPP1R9B	chr17	0.00409563	-2.20495
3762174	NM_032595 // PPP1R9B /// ENST00000316878 // PPP1R9B	PPP1R9B	chr17	0.00915786	-1.75301
3762346	NM_016504 // MRPL27 /// ENST00000225969 // MRPL27 /// AK023576 // MRPL27 /// ENS	MRPL27	chr17	0.0413467	-1.73202
3762387	NM_001267 // CHAD /// ENST00000258969 // CHAD /// AK292177 // CHAD	CHAD	chr17	0.0097	-1.63251
3764122	NM_006924 // SFRS1 /// NM_001078166 // SFRS1 /// ENST00000258962 // SFRS1 /// BC	SFRS1	chr17	0.0195491	-1.5115
3764264	NM_000250 // MPO /// ENST00000225275 // MPO /// M19507 // MPO /// ENST00000340ꞓ	MPO	chr17	0.0204068	-1.62169
3764344	NM_004758 // BZRAP1 /// NM_024418 // BZRAP1 /// ENST00000343736 // BZRAP1 /// EN	BZRAP1	chr17	0.0207999	-1.56312
3766420	NM_001098426 // SMARCD2 /// ENST00000448276 // SMARCD2 /// AK300939 // SMARCD2 /	SMARCD2	chr17	0.0267273	-1.63562
3767355	NM_006572 // GNA13 /// ENST00000439174 // GNA13 /// BC036756 // GNA13 /// ENST0C	GNA13	chr17	0.000942077	-1.8927
3768478	NM_017983 // WIPI1 /// ENST00000262139 // WIPI1 /// BC039867 // WIPI1 /// AB0232	WIPI1	chr17	0.00437356	-1.53728
3768539	NR_027751 // FAM20A /// NM_017565 // FAM20A /// ENST00000226094 // FAM20A /// BC	FAM20A	chr17	7.64027E-05	-1.72319
3771378	NM_001013839 // EXOC7 /// NM_015219 // EXOC7 /// NR_028133 // EXOC7 /// NM_0011-	EXOC7	chr17	0.00774062	-1.51201
3771604	NM_024599 // RHBDF2 /// NM_001005498 // RHBDF2 /// ENST00000313080 // RHBDF2 //.	RHBDF2	chr17	0.0260615	-1.51772
3771624	NM_024599 // RHBDF2 /// NM_001005498 // RHBDF2 /// ENST00000313080 // RHBDF2 //.	RHBDF2	chr17	0.0311658	-1.67
3772167	NM_003258 // TK1 /// ENST00000301634 // TK1 /// ENST00000405273 // TK1 /// K025ꞓ	TK1	chr17	0.0303405	-1.6364
3773436	NM_002522 // NPTX1 /// ENST00000306773 // NPTX1 /// BC089441 // NPTX1	NPTX1	chr17	0.00288961	-1.99297
3774554	NM_016286 // DCXR /// ENST00000306869 // DCXR /// AF139841 // DCXR	DCXR	chr17	0.0209076	-1.83997
3774840	NM_001893 // CSNK1D /// NM_139062 // CSNK1D /// ENST00000314028 // CSNK1D /// EN	CSNK1D	chr17	0.0278036	-1.7079
3776523	NM_003244 // TGIF1 /// ENST00000343820 // TGIF1 /// ENST00000340165 // TGIF1	TGIF1	chr18	0.00619328	-2.74608
3778069	BC040542 // KIAA0802 /// NM_015210 // KIAA0802 /// BX648911 // KIAA0802 /// ENST	KIAA0802	chr18	0.0371589	-1.59022

3779401	NM_014214 // IMPA2 /// ENST00000269159 // IMPA2 /// AF014398 // IMPA2	IMPA2	chr18	0.015533	-1.78255
3780103	NM_181481 // C18orf1 /// NM_004338 // C18orf1 /// NM_001003674 // C18orf1 /// EN	C18orf1	chr18	0.0446414	-1.79165
3784785	NM_017947 // MOCOS /// ENST00000261326 // MOCOS /// BC012079 // MOCOS	MOCOS	chr18	0.035563	-2.00852
3784821	NM_017947 // MOCOS /// ENST00000261326 // MOCOS /// BC012079 // MOCOS	MOCOS	chr18	0.00793818	-1.54982
3788261	NM_002396 // ME2 /// NM_001168335 // ME2 /// ENST00000321341 // ME2 /// BC000147	ME2	chr18	0.00305308	-1.60559
3792299	NM_033646 // CDH7 /// NM_004361 // CDH7 /// ENST00000323011 // CDH7 /// ENST0000	CDH7	chr18	0.0367798	-1.53955
3795215	NM_172390 // NFATC1 /// NM_006162 // NFATC1 /// NM_172387 // NFATC1 /// NM_17238	NFATC1	chr18	0.0414658	-1.51052
3796744	NM_004746 // DLGAP1 /// ENST00000315677 // DLGAP1 /// AK304101 // DLGAP1	DLGAP1	chr18	0.0353671	-1.66258
3797079	NM_012307 // EPB41L3 /// ENST00000341928 // EPB41L3 /// ENST00000342933 // EPB41	EPB41L3	chr18	0.0245844	-1.62262
3797116	BC008377 // EPB41L3	EPB41L3	chr18	0.00170569	-2.19421
3799667	NM_002828 // PTPN2 /// NM_080422 // PTPN2 /// NM_080423 // PTPN2 /// ENST0000032	PTPN2	chr18	0.00317902	-1.74605
3803152	NM_004775 // B4GALT6 /// ENST00000306851 // B4GALT6 /// AF038664 // B4GALT6 ///	B4GALT6	chr18	0.0464065	-1.61091
3807835	NM_001101654 // CXXC1 /// NM_014593 // CXXC1 /// ENST00000285106 // CXXC1 /// EN	CXXC1	chr18	0.0239318	-1.85371
3808904	NM_001083962 // TCF4 /// NM_003199 // TCF4 /// ENST00000356073 // TCF4 /// ENST0	TCF4	chr18	0.00207934	-1.65595
3809786	---	0	chr18	0.00124084	-1.6646
3813620	NM_175907 // ZADH2 /// ENST00000322342 // ZADH2 /// BC078661 // ZADH2	ZADH2	chr18	0.037128	-1.5539
3815129	NM_002579 // PALM /// NM_001040134 // PALM /// ENST00000338448 // PALM /// ENST0	PALM	chr19	0.0407256	-1.8097
3815907	NM_005883 // APC2 /// ENST00000233607 // APC2 /// AB012162 // APC2	APC2	chr19	0.017049	-1.51501
3815909	NM_005883 // APC2 /// ENST00000233607 // APC2 /// AB012162 // APC2	APC2	chr19	0.0181856	-1.67286
3815915	NM_005883 // APC2 /// ENST00000233607 // APC2 /// AB012162 // APC2 /// ENST0000	APC2	chr19	0.0115882	-1.64986
3816297	NM_032482 // DOT1L /// ENST00000398665 // DOT1L /// AF509504 // DOT1L /// ENST00	DOT1L	chr19	0.0313492	-1.62776
3816522	NM_015675 // GADD45B /// ENST00000215631 // GADD45B /// AF087853 // GADD45B	GADD45B	chr19	0.0254251	-1.76585
3816823	NM_002068 // GNA15 /// ENST00000262958 // GNA15 /// BC013585 // GNA15	GNA15	chr19	0.0235267	-1.5001
3816896	NR_033342 // CELF5 /// NM_021938 // CELF5 /// NM_001172673 // CELF5 /// ENST000	CELF5	chr19	0.0195986	-1.99571
3817169	NM_172251 // MRPL54 /// ENST00000330133 // MRPL54 /// BC065273 // MRPL54	MRPL54	chr19	0.0397224	-1.71571
3818147	NM_004558 // NRTN /// ENST00000303212 // NRTN /// BC137399 // NRTN	NRTN	chr19	0.0345922	-1.51808
3819210	NM_001159944 // EVI5L /// NM_145245 // EVI5L /// ENST00000270530 // EVI5L /// BC	EVI5L	chr19	0.0103003	-1.57566
3819287	NM_145185 // MAP2K7 /// ENST00000397979 // MAP2K7 /// BC038295 // MAP2K7 /// ENS	MAP2K7	chr19	0.000450356	-1.84624
3819326	NM_003083 // SNAPC2 /// NR_030717 // SNAPC2 /// ENST00000221573 // SNAPC2 /// U4	SNAPC2	chr19	0.0335499	-1.59785
3819545	NM_005968 // HNRNPM /// NM_031203 // HNRNPM /// ENST00000325495 // HNRNPM /// EN	HNRNPM	chr19	0.025526	-1.55697
3819633	---	0	chr19	0.00929638	-1.79322
3819711	---	0	chr19	0.0246194	-1.5313
3820584	NM_032885 // ATG4D /// ENST00000309469 // ATG4D /// AK294676 // ATG4D	ATG4D	chr19	0.0457475	-1.58597
3820592	NM_032885 // ATG4D /// ENST00000309469 // ATG4D /// AK294676 // ATG4D	ATG4D	chr19	0.0273383	-1.60939
3820654	NM_020428 // SLC44A2 /// NM_001145056 // SLC44A2 /// ENST00000335757 // SLC44A2	SLC44A2	chr19	0.0256398	-1.51904
3820745	NM_031209 // QTRT1 /// ENST00000250237 // QTRT1	QTRT1	chr19	0.0193672	-1.53228
3821087	NM_018687 // LOC55908 /// AF271350 // LOC55908 /// ENST00000252453 // LOC55908	LOC55908	chr19	0.00177586	-1.78949
3822024	NM_000159 // GCDH /// NM_013976 // GCDH /// ENST00000222214 // GCDH /// ENST0000	GCDH	chr19	0.0452514	-1.54594
3822069	NM_004343 // CALR /// ENST00000316448 // CALR	CALR	chr19	0.00146119	-2.58735
3822159	NM_002501 // NFIX /// ENST00000360105 // NFIX /// BX537794 // NFIX /// ENST0000	NFIX	chr19	0.0291312	-1.68021
3822447	---	0	chr19	0.00484361	-1.67308
3823026	NM_033025 // SYDE1 /// ENST00000342784 // SYDE1 /// AK128870 // SYDE1	SYDE1	chr19	0.026974	-1.72959
3824438	NM_173544 // FAM129C /// NM_001098524 // FAM129C /// ENST00000335393 // FAM129C	FAM129C	chr19	0.012825	-1.81621
3825517	NM_033415 // ARMC6 /// ENST00000392335 // ARMC6 /// ENST00000269932 // ARMC6 //.	ARMC6	chr19	0.028527	-1.93019
3829950	---	0	chr19	0.0155551	-1.59044
3830219	NR_028406 // FXYD5 /// NM_001164605 // FXYD5 /// ENST00000342879 // FXYD5 /// EN	FXYD5	chr19	0.012554	-1.64043
3830302	NM_003367 // USF2 /// NM_207291 // USF2 /// ENST00000222305 // USF2 /// ENST000	USF2	chr19	0.0256486	-1.5151
3830628	NM_024321 // RBM42 /// ENST00000262633 // RBM42 /// BC004204 // RBM42 /// ENST0	RBM42	chr19	0.00616356	-1.57612
3830820	NM_019104 // LIN37 /// AK293116 // LIN37 /// ENST00000301159 // LIN37	LIN37	chr19	0.0364156	-1.55083
3830975	NM_001024807 // APLP1 /// NM_005166 // APLP1 /// ENST00000221891 // APLP1 /// BC	APLP1	chr19	0.0205969	-1.8514
3832562	NM_000540 // RYR1 /// NM_001042723 // RYR1 /// ENST00000359596 // RYR1 /// ENST0	RYR1	chr19	0.0499824	-2.09495

3832585	NM_000540 // RYR1 /// NM_001042723 // RYR1 /// ENST00000359596 // RYR1 /// ENST(	RYR1	chr19	0.013527	-1.75739
3832721	NM_004924 // ACTN4 /// ENST00000252699 // ACTN4 /// BC005033 // ACTN4 /// ENST00	ACTN4	chr19	0.017862	-1.5058
3833333	NM_178544 // ZNF546 /// ENST00000347077 // ZNF546 /// BC045649 // ZNF54€	ZNF546	chr19	0.00674037	-1.52126
3833421	NM_002446 // MAP3K10 /// ENST00000253055 // MAP3K10 /// X90846 // MAP3K1(	MAP3K10	chr19	0.0320148	-1.53896
3833457	NM_001031696 // PLD3 /// NM_012268 // PLD3 /// ENST00000409735 // PLD3 /// ENST(	PLD3	chr19	0.01009	-1.77241
3833524	NM_020971 // SPTBN4 /// ENST00000352632 // SPTBN4 /// ENST00000428507 // SPTBN4	SPTBN4	chr19	0.006135	-1.73252
3833673	NM_001042544 // LTBP4 /// NM_003573 // LTBP4 /// NM_001042545 // LTBP4 /// ENST(	LTBP4	chr19	0.0296302	-1.69478
3834416	D90278 // CEACAM3 /// ENST00000415495 // CEACAM3	CEACAM3	chr19	0.00898017	-1.54331
3834566	NM_199002 // ARHGEF1 /// NM_004706 // ARHGEF1 /// NM_198977 // ARHGEF1 /// ENST(	ARHGEF1	chr19	0.0230488	-2.0426
3834788	NM_001410 // MEGF8 /// ENST00000334370 // MEGF8 /// AY280362 // MEGF8 /// BC153ξ	MEGF8	chr19	0.0265317	-1.50166
3835382	NM_013361 // ZNF223 /// ENST00000434772 // ZNF223 /// BC022466 // ZNF223 /// ENS	ZNF223	chr19	0.0241705	-1.53328
3835689	NM_001127893 // CEACAM19 /// NM_020219 // CEACAM19 /// ENST00000358777 // CEACAM	CEACAM19	chr19	0.0368755	-1.9218
3836171	NM_031417 // MARK4 /// ENST00000300843 // MARK4 /// AB049127 // MARK4 /// ENST0(	MARK4	chr19	0.0318942	-1.71403
3836436	NM_017659 // QPCTL /// NM_001163377 // QPCTL /// ENST00000012049 // QPCTL /// BC	QPCTL	chr19	0.00268443	-1.79077
3836536	NM_004497 // FOXA3 /// ENST00000302177 // FOXA3 /// BC016024 // FOXA3	FOXA3	chr19	0.0190151	-2.21903
3836750	AK299699 // HIF3A /// AK021421 // HIF3A	HIF3A	chr19	0.0357081	-1.54623
3837197	NM_015603 // CCDC9 /// ENST00000221922 // CCDC9 /// AK302153 // CCDC9	CCDC9	chr19	0.0114056	-1.54316
3837213	NM_015603 // CCDC9 /// ENST00000221922 // CCDC9 /// AK302153 // CCDC9	CCDC9	chr19	0.0262586	-1.77411
3837321	NM_014681 // DHX34 /// ENST00000328771 // DHX34 /// ENST00000257252 // DHX34 //.	DHX34	chr19	0.0170292	-1.73727
3838143	NM_006666 // RUVBL2 /// ENST00000221413 // RUVBL2 /// AK057498 // RUVBL2	RUVBL2	chr19	0.0346292	-1.88431
3838440	NM_014419 // DKKL1 /// ENST00000221498 // DKKL1 /// AY358922 // DKKL1	DKKL1	chr19	0.00181207	-2.15196
3838761	NM_021228 // SCAF1 /// ENST00000360565 // SCAF1 /// ENST00000447618 // SCAF1 //.	SCAF1	chr19	0.00724111	-2.02151
3839043	NM_017432 // PTOV1 /// ENST00000391842 // PTOV1 /// BC042921 // PTOV1	PTOV1	chr19	0.0370489	-1.5233
3839072	NM_024682 // TBC1D17 /// NM_001168222 // TBC1D17 /// ENST00000221543 // TBC1D17	TBC1D17	chr19	0.0045001	-2.29864
3839314	NM_002691 // POLD1 /// ENST00000440232 // POLD1 /// ENST00000262266 // POLD1 //.	POLD1	chr19	0.00519272	-1.76563
3839328	NM_002691 // POLD1 /// ENST00000440232 // POLD1 /// ENST00000262266 // POLD1 //.	POLD1	chr19	0.0316948	-1.71864
3839727	NM_001772 // CD33 /// NM_001082618 // CD33 /// NM_001177608 // CD33 /// ENST000(	CD33	chr19	0.0289035	-1.73972
3840382	NM_001172655 // ZNF701 /// NM_018260 // ZNF701 /// ENST00000301093 // ZNF701 //.	ZNF701	chr19	0.0168639	-1.68737
3841328	NM_024075 // TSEN34 /// NM_001077446 // TSEN34 /// ENST00000302937 // TSEN34 //.	TSEN34	chr19	0.00657999	-1.52351
3842095	NM_032430 // BRSK1 /// ENST00000309383 // BRSK1 /// AF479827 // BRSK1 /// ENST0(	BRSK1	chr19	0.0215034	-1.51845
3843469	NM_138347 // ZNF551 /// ENST00000282296 // ZNF551 /// BC005868 // ZNF551 /// ENS	ZNF551	chr19	0.00417268	-2.55324
3843807	NM_016325 // ZNF274 /// NM_133502 // ZNF274 /// ENST00000424679 // ZNF274 /// EN	ZNF274	chr19	0.0374613	-1.71033
3843820	NM_016325 // ZNF274 /// NM_016324 // ZNF274 /// NM_133502 // ZNF274 /// ENST000(	ZNF274	chr19	0.0142803	-1.64252
3843923	NM_021089 // ZNF8 /// ENST00000196548 // ZNF8 /// AK225825 // ZNF8	ZNF8	chr19	0.03755	-1.69066
3844195	NM_017908 // ZNF446 /// ENST00000335841 // ZNF446 /// ENST00000391694 // ZNF446	ZNF446	chr19	0.00725834	-1.61837
3844849	NM_005481 // MED16 /// ENST00000325464 // MED16 /// AF106934 // MED16 /// ENST0(	MED16	chr19	0.0137243	-1.61387
3844931	NM_001033026 // C19orf6 /// NM_033420 // C19orf6 /// ENST00000356663 // C19orf6	C19orf6	chr19	0.0140582	-1.67787
3845099	NM_152769 // C19orf26 /// ENST00000215376 // C19orf26 /// BC028156 // C19orf26 /	C19orf26	chr19	0.0290519	-1.52302
3845100	NM_152769 // C19orf26 /// ENST00000215376 // C19orf26 /// BC028156 // C19orf26 /	C19orf26	chr19	0.00331971	-1.60215
3845253	NM_017573 // PCSK4 /// ENST00000300954 // PCSK4 /// ENST00000441747 // PCSK4 //.	PCSK4	chr19	0.0131388	-1.97687
3845412	NM_003200 // TCF3 /// NM_001136139 // TCF3 /// ENST00000262965 // TCF3 /// ENST(	TCF3	chr19	0.00270278	-1.73902
3845649	NM_017572 // MKNK2 /// NM_199054 // MKNK2 /// ENST00000250896 // MKNK2 /// ENST(	MKNK2	chr19	0.00379537	-1.55208
3845701	NM_130807 // MOBKL2A /// ENST00000357066 // MOBKL2A /// AL832474 // MOBKL2A	MOBKL2A	chr19	0.0353406	-1.81615
3845724	NM_001077523 // AP3D1 /// NM_003938 // AP3D1 /// ENST00000345016 // AP3D1 /// EN	AP3D1	chr19	0.0453987	-1.61027
3845836	NM_198532 // C19orf35 /// ENST00000342063 // C19orf35	C19orf35	chr19	0.0375262	-1.77032
3846523	NM_001348 // DAPK3 /// ENST00000301264 // DAPK3 /// AK027590 // DAPK3 /// ENST0(	DAPK3	chr19	0.0447208	-1.63996
3846551	NM_001961 // EEF2 /// ENST00000309311 // EEF2 /// BC126259 // EEF2 /// ENST0000(	EEF2	chr19	0.0113585	-2.06193
3846934	NM_139159 // DPP9 /// ENST00000262960 // DPP9 /// AF452102 // DPP9 /// ENST0000(	DPP9	chr19	0.00578375	-1.63083
3847010	NM_005817 // PLIN3 /// NM_001164189 // PLIN3 /// NM_001164194 // PLIN3 /// ENST(	PLIN3	chr19	0.021705	-1.52187
3847155	NM_002850 // PTPRS /// NM_130854 // PTPRS /// NM_130853 // PTPRS /// NM_130855 /	PTPRS	chr19	0.0109189	-1.55442
3847473	NM_000150 // FUT6 /// NM_001040701 // FUT6 /// ENST00000318336 // FUT6 /// ENST(	FUT6	chr19	0.00199792	-2.03672

3847828	NM_003685 // KHSRP /// ENST00000398148 // KHSRP /// U94832 // KHSRP /// ENST0000	KHSRP	chr19	0.032296	-1.63252
3847900	NM_024103 // SLC25A23 /// ENST00000301454 // SLC25A23 /// AY750170 // SLC25A23	SLC25A23	chr19	0.025353	-1.68231
3847909	NM_024898 // DENND1C /// ENST00000381480 // DENND1C /// AK026410 // DENND1C	DENND1C	chr19	0.0140565	-1.74735
3847996	NM_001252 // CD70 /// ENST00000245903 // CD70 /// L08096 // CD70	CD70	chr19	0.00366418	-1.51862
3848705	NM_001419 // ELAVL1 /// ENST00000407627 // ELAVL1 /// BC003376 // ELAVL1 /// ENS	ELAVL1	chr19	0.0207511	-1.66236
3848877	NM_016579 // CD320 /// NM_001165895 // CD320 /// ENST00000301458 // CD320 /// AF	CD320	chr19	0.0274144	-1.90294
3849123	NM_030957 // ADAMTS10 /// ENST00000270328 // ADAMTS10 /// ENST00000393912 // ADA	ADAMTS10	chr19	0.013851	-1.59145
3849493	NM_001005191 // OR7D4 /// ENST00000308682 // OR7D4 /// BC137147 // OR7D4	OR7D4	chr19	0.0217283	-1.67213
3850238	NM_133452 // RAVER1 /// ENST00000293677 // RAVER1 /// AB075858 // RAVER1 /// ENS	RAVER1	chr19	0.0389053	-2.11889
3850251	NM_133452 // RAVER1 /// ENST00000293677 // RAVER1 /// AB075858 // RAVER1 /// ENS	RAVER1	chr19	0.0440164	-1.5947
3850827	NM_004283 // RAB3D /// ENST00000222120 // RAB3D /// BC016471 // RAB3D	RAB3D	chr19	0.0436107	-1.58281
3850870	NM_000121 // EPOR /// ENST00000222139 // EPOR /// BC019092 // EPOR	EPOR	chr19	0.0371407	-1.52433
3851313	BC136502 // ZNF44 /// ENST00000397742 // ZNF44	ZNF44	chr19	0.0159698	-2.81123
3851697	NM_001136196 // TNPO2 /// ENST00000450764 // TNPO2	TNPO2	chr19	0.0462963	-1.62678
3852444	NM_002918 // RFX1 /// ENST00000254325 // RFX1 /// BC049826 // RFX1	RFX1	chr19	0.0408268	-1.50032
3852694	NM_005804 // DDX39 /// ENST00000242776 // DDX39 /// U90426 // DDX39 /// ENST0000	DDX39	chr19	0.0487336	-1.86295
3853461	NM_022904 // RASAL3 /// ENST00000343625 // RASAL3 /// AK024488 // RASAL3	RASAL3	chr19	0.0433576	-1.5298
3854166	NM_015692 // CPAMD8 /// ENST00000443236 // CPAMD8 /// ENST00000291440 // CPAMD8	CPAMD8	chr19	0.0130088	-1.51295
3854328	NM_031941 // USHBP1 /// ENST00000252597 // USHBP1 /// AB040046 // USHBP1 /// ENS	USHBP1	chr19	0.000934543	-1.56871
3854964	NM_145256 // LRRC25 /// ENST00000339007 // LRRC25	LRRC25	chr19	0.00459574	-1.55463
3855098	---	0	chr19	0.0017938	-1.6492
3855690	NM_172231 // SF4 /// ENST00000247001 // SF4 /// AY072916 // SF4 /// ENST0000033	SF4	chr19	0.00484619	-1.96425
3855832	NM_025245 // PBX4 /// ENST00000251203 // PBX4 /// BC141859 // PBX4	PBX4	chr19	0.014373	-1.73628
3858786	NM_014270 // SLC7A9 /// NM_001126335 // SLC7A9 /// ENST00000023064 // SLC7A9 //	SLC7A9	chr19	0.00328533	-1.72777
3860774	NM_152655 // ZNF585A /// NM_199126 // ZNF585A /// ENST00000292841 // ZNF585A //	ZNF585A	chr19	0.00461386	-1.66749
3861403	NM_170604 // RASGRP4 /// NM_001146202 // RASGRP4 /// NM_001146203 // RASGRP4 //	RASGRP4	chr19	0.000176683	-1.53031
3861598	NM_001398 // ECH1 /// ENST00000221418 // ECH1 /// BC011792 // ECH1	ECH1	chr19	0.00325626	-1.8016
3862196	NM_003890 // FCGBP /// ENST00000221347 // FCGBP /// D84239 // FCGBP /// ENST0000	FCGBP	chr19	0.0201981	-1.51382
3862617	NM_020956 // PRX /// NM_181882 // PRX /// ENST00000324001 // PRX /// ENST0000029	PRX	chr19	0.0077969	-1.74361
3862664	NM_000713 // BLVRB /// ENST00000263368 // BLVRB /// BC109371 // BLVRB	BLVRB	chr19	0.0467918	-1.5655
3863062	NM_020158 // EXOSC5 /// ENST00000221233 // EXOSC5 /// AF285785 // EXOSC5	EXOSC5	chr19	0.0100299	-1.84107
3863089	NR_030765 // ATP5SL /// NM_001167867 // ATP5SL /// NM_001167868 // ATP5SL /// NM	ATP5SL	chr19	0.0390197	-1.65332
3864535	NM_145296 // CADM4 /// ENST00000222374 // CADM4 /// AF363368 // CADM4	CADM4	chr19	0.0158255	-1.5958
3864537	NM_145296 // CADM4 /// ENST00000222374 // CADM4 /// AF363368 // CADM4	CADM4	chr19	0.0112242	-1.61654
3865208	ENST00000317951 // NKPD1 /// ENST00000438936 // NKPD1 /// NM_198478 // NKPD1 //	NKPD1	chr19	0.0336547	-1.6288
3865373	NM_001142502 // PPP1R13L /// NM_006663 // PPP1R13L /// ENST00000418234 // PPP1R1	PPP1R13L	chr19	0.018009	-2.0965
3865621	NM_175875 // SIX5 /// ENST00000317578 // SIX5	SIX5	chr19	0.0293878	-2.03722
3865783	NM_015649 // IRF2BP1 /// ENST00000302165 // IRF2BP1 /// BC038222 // IRF2BP1	IRF2BP1	chr19	0.0477791	-1.62451
3865985	NM_032040 // CCDC8 /// ENST00000307522 // CCDC8 /// BC025243 // CCDC8	CCDC8	chr19	0.0107894	-2.00324
3866436	NM_001127240 // BBC3 /// NM_001127241 // BBC3 /// NM_001127242 // BBC3 /// NM_01	BBC3	chr19	0.0129257	-1.50654
3867465	NM_020904 // PLEKHA4 /// NM_001161354 // PLEKHA4 /// ENST00000263265 // PLEKHA4	PLEKHA4	chr19	0.0409307	-1.87589
3868133	NM_007254 // PNKP /// ENST00000322344 // PNKP /// BC013034 // PNKP	PNKP	chr19	0.0120226	-1.58043
3868567	NM_032298 // SYT3 /// NM_001160328 // SYT3 /// NM_001160329 // SYT3 /// ENST0000	SYT3	chr19	0.00919022	-1.64916
3868605	NM_016148 // SHANK1 /// ENST00000293441 // SHANK1 /// AF163302 // SHANK1 /// AF2	SHANK1	chr19	0.011758	-1.64396
3868692	NM_002257 // KLK1 /// ENST00000301420 // KLK1 /// AY429508 // KLK1	KLK1	chr19	0.0402973	-1.5498
3868864	NM_019598 // KLK12 /// NM_145894 // KLK12 /// ENST00000319590 // KLK12 /// ENST0	KLK12	chr19	0.0489036	-1.56537
3869901	NR_003578 // ZNF702P	ZNF702P	chr19	0.0150498	-1.53927
3870774	NM_021250 // LILRA5 /// NM_181985 // LILRA5 /// NM_181879 // LILRA5 /// NM_18198	LILRA5	chr19	0.0128705	-1.67137
3870993	NM_001083899 // GP6 /// NM_016363 // GP6 /// ENST00000417454 // GP6 /// ENST0000	GP6	chr19	0.0451983	-1.73151
3872388	NM_001085384 // ZNF154 /// ENST00000426889 // ZNF154 /// BC152561 // ZNF154	ZNF154	chr19	0.00307988	-1.64536
3872687	NM_001145542 // ZSCAN18 /// NM_001145544 // ZSCAN18 /// NM_023926 // ZSCAN18 //	ZSCAN18	chr19	0.029686	-1.84156

3874200	NM_000915 // OXT /// ENST00000217386 // OXT /// BC101841 // OXT	OXT	chr20	0.0381476	-1.54708
3874404	NM_052970 // HSPA12B /// ENST00000254963 // HSPA12B /// BC110881 // HSPA12B ///	HSPA12B	chr20	0.0140056	-1.52888
3878725	NM_020689 // SLC24A3 /// ENST00000328041 // SLC24A3	SLC24A3	chr20	0.0169038	-1.65097
3880276	NM_005492 // CST8 /// ENST00000246012 // CST8 /// AF059244 // CST8 /// ENST0000C	CST8	chr20	0.0326274	-1.71703
3881290	NM_030789 // HM13 /// NM_178580 // HM13 /// NM_178581 // HM13 /// NM_178582 // H	HM13	chr20	0.0267314	-1.54943
3882222	NM_174897 // BPIL3 /// ENST00000349552 // BPIL3 /// AF465767 // BPIL3	BPIL3	chr20	0.00370386	-2.71704
3882345	NR_026760 // BASE /// AY180924 // BASE	BASE	chr20	0.0106498	-1.70416
3883344	NM_007186 // CEP250 /// ENST00000397527 // CEP250 /// ENST00000356095 // CEP250	CEP250	chr20	0.00785618	-1.79376
3883775	NM_012156 // EPB41L1 /// NM_177996 // EPB41L1 /// ENST00000338074 // EPB41L1 //	EPB41L1	chr20	0.00210721	-1.59478
3883879	NM_014902 // DLGAP4 /// NM_183006 // DLGAP4 /// NM_001042486 // DLGAP4 /// ENSTC	DLGAP4	chr20	0.0144595	-1.52501
3883922	NM_006097 // MYL9 /// NM_181526 // MYL9 /// ENST00000346786 // MYL9 /// ENST000C	MYL9	chr20	0.0312475	-1.78265
3885570	NM_002660 // PLCG1 /// NM_182811 // PLCG1 /// ENST00000373271 // PLCG1 /// ENSTC	PLCG1	chr20	0.0245934	-1.60742
3886770	NM_002638 // PI3 /// ENST00000243924 // PI3 /// BC010952 // PI3	PI3	chr20	0.0453905	-1.86552
3886785	NM_003007 // SEMG1 /// ENST00000372781 // SEMG1 /// ENST00000445493 // SEMG1 //	SEMG1	chr20	0.0217802	-1.56968
3887083	AF258570 // SNX21	SNX21	chr20	0.0165083	-1.50761
3887576	NM_005244 // EYA2 /// ENST00000360649 // EYA2 /// ENST00000327619 // EYA2 /// EN	EYA2	chr20	0.00960913	-1.58862
3891073	NM_024663 // NPEPL1 /// ENST00000356091 // NPEPL1 /// ENST00000371137 // NPEPL1	NPEPL1	chr20	0.0449418	-1.83018
3891318	NM_198976 // TH1L /// ENST00000344018 // TH1L /// AK293410 // TH1L	TH1L	chr20	0.0199065	-1.59108
3892234	NM_001794 // CDH4 /// ENST00000360469 // CDH4 /// BC101651 // CDH4	CDH4	chr20	0.0304463	-1.74629
3893359	NM_020882 // COL20A1 /// ENST00000358894 // COL20A1 /// BC043183 // COL20A1 ///	COL20A1	chr20	0.0173588	-1.60443
3893573	NM_016434 // RTEL1 /// NM_032957 // RTEL1 /// ENST00000370018 // RTEL1 /// ENSTC	RTEL1	chr20	0.0356349	-1.73565
3893640	NM_032527 // ZGPAT /// NM_181485 // ZGPAT /// NM_001083113 // ZGPAT /// ENST000C	ZGPAT	chr20	0.00682537	-2.07489
3893683	NM_020062 // SLC2A4RG /// ENST00000266077 // SLC2A4RG /// BC052306 // SLC2A4RG	SLC2A4RG	chr20	0.0447671	-1.6397
3893803	NM_025219 // DNAJC5 /// ENST00000360864 // DNAJC5 /// AK128776 // DNAJC5 /// ENS	DNAJC5	chr20	0.0103832	-1.50814
3894556	NM_080489 // SDCBP2 /// NM_015685 // SDCBP2 /// ENST00000339987 // SDCBP2 /// EN	SDCBP2	chr20	0.016823	-1.58249
3895001	NM_198216 // SNRPB /// NM_003091 // SNRPB /// ENST00000381342 // SNRPB /// ENSTC	SNRPB	chr20	0.0288097	-1.99303
3896042	NM_014737 // RASSF2 /// NM_170774 // RASSF2 /// ENST00000379400 // RASSF2 /// EN	RASSF2	chr20	0.0261319	-1.78082
3896052	NM_014737 // RASSF2 /// NM_170774 // RASSF2 /// ENST00000379400 // RASSF2 /// EN	RASSF2	chr20	0.0459413	-1.64645
3900558	NM_002509 // NKX2-2 /// ENST00000377142 // NKX2-2 /// BC075093 // NKX2-2	NKX2-2	chr20	0.0410916	-1.53119
3903124	NM_031231 // NECAB3 /// NM_031232 // NECAB3 /// ENST00000375238 // NECAB3 /// EN	NECAB3	chr20	0.0079341	-1.78152
3904519	NM_032214 // SLA2 /// NM_175077 // SLA2 /// ENST00000262866 // SLA2 /// ENST000C	SLA2	chr20	0.0235184	-1.68138
3904521	NM_032214 // SLA2 /// NM_175077 // SLA2 /// ENST00000262866 // SLA2 /// ENST000C	SLA2	chr20	0.032926	-1.71325
3905150	NM_004613 // TGM2 /// ENST00000361475 // TGM2 /// M55153 // TGM2	TGM2	chr20	0.0104143	-1.7682
3906064	NM_015035 // ZHX3 /// ENST00000309060 // ZHX3 /// ENST00000373263 // ZHX3 /// AE	ZHX3	chr20	0.0475759	-1.55953
3906535	NM_133170 // PTPRT /// NM_007050 // PTPRT /// ENST00000373198 // PTPRT /// ENST0	PTPRT	chr20	0.0201147	-1.50314
3907589	NM_022095 // ZNF335 /// ENST00000322927 // ZNF335 /// AF395833 // ZNF335 /// ENS	ZNF335	chr20	0.0319853	-1.79686
3907756	NM_021248 // CDH22 /// ENST00000372262 // CDH22 /// BC136526 // CDH22	CDH22	chr20	0.0126942	-1.62363
3907794	NM_173179 // SLC35C2 /// NM_015945 // SLC35C2 /// NM_173073 // SLC35C2 /// ENSTC	SLC35C2	chr20	0.0403017	-1.90441
3908790	NM_017454 // STAU1 /// NM_017452 // STAU1 /// NM_017453 // STAU1 /// NM_004602 /	STAU1	chr20	0.0379151	-1.66248
3909129	NM_199129 // TMEM189 /// NM_001162505 // TMEM189 /// NR_027889 // TMEM189 /// NM	TMEM189	chr20	0.0272653	-1.60107
3911369	ENST00000457363 // ANKRD60 /// ENST00000371167 // ANKRD60 /// XM_001134442 // AN	ANKRD60	chr20	0.0112704	-1.72117
3913065	NM_005560 // LAMA5 /// ENST00000252999 // LAMA5 /// ENST00000370691 // LAMA5 //	LAMA5	chr20	0.0171384	-1.62141
3913069	NM_005560 // LAMA5 /// ENST00000252999 // LAMA5 /// ENST00000370691 // LAMA5 //	LAMA5	chr20	0.0353125	-1.64973
3913121	NM_005560 // LAMA5 /// ENST00000252999 // LAMA5 /// ENST00000370691 // LAMA5 //	LAMA5	chr20	0.0451187	-1.57872
3913840	NM_172107 // KCNQ2 /// NM_172106 // KCNQ2 /// NM_004518 // KCNQ2 /// NM_172108 /	KCNQ2	chr20	0.0200635	-1.65418
3913988	NM_001037335 // PRIC285 /// NM_033405 // PRIC285 /// ENST00000252889 // PRIC285	PRIC285	chr20	0.0199289	-1.51146
3914197	BC033078 // UCKL1 /// ENST00000358711 // UCKL1	UCKL1	chr20	0.0159259	-1.65358
3914328	BC036837 // C20orf201 /// ENST00000308906 // C20orf201	C20orf201	chr20	0.0480678	-1.91016
3915499	NM_001338 // CXADR /// ENST00000284878 // CXADR /// BC010536 // CXADR /// ENST0C	CXADR	chr21	0.00922359	-1.52528
3917248	NM_020152 // C21orf7 /// ENST00000341618 // C21orf7 /// ENST00000399947 // C21oı	C21orf7	chr21	0.01868	-1.56427
3918540	NM_000628 // IL10RB /// ENST00000290200 // IL10RB /// BC001903 // IL10RB /// ENS	IL10RB	chr21	0.0154612	-1.85914

3919964	NM_015358 // MORC3 /// ENST00000400485 // MORC3 /// BC094779 // MORC3 /// BC1327	MORC3	chr21	0.023847	-1.51471
3922504	NM_016818 // ABCG1 /// NM_207174 // ABCG1 /// NM_004915 // ABCG1 /// NM_207627 /	ABCG1	chr21	0.00143882	-1.56682
3923341	NM_003683 // RRP1 /// ENST00000291569 // RRP1 /// BC000380 // RRP1	RRP1	chr21	0.0237994	-1.65067
3923515	NM_005049 // PWP2 /// ENST00000291576 // PWP2 /// U56085 // PWP2	PWP2	chr21	0.0210539	-1.51772
3923545	NM_004649 // C21orf33 /// NM_198155 // C21orf33 /// U53003 // C21orf33 /// ENST0	C21orf33	chr21	0.0107603	-1.56874
3923651	NR_024108 // PFKL /// NM_002626 // PFKL /// ENST00000349048 // PFKL /// ENST0000	PFKL	chr21	0.0282619	-1.5023
3923664	NR_024108 // PFKL /// NM_002626 // PFKL /// ENST00000349048 // PFKL /// ENST0000	PFKL	chr21	0.00674407	-1.74183
3923749	NM_003307 // TRPM2 /// ENST00000300482 // TRPM2 /// ENST00000397928 // TRPM2 //	TRPM2	chr21	0.0315481	-1.71273
3924332	NM_020528 // PCBP3 /// NM_001130141 // PCBP3 /// ENST00000400314 // PCBP3 /// EN	PCBP3	chr21	0.0274302	-1.79444
3924392	NM_001848 // COL6A1 /// ENST00000361866 // COL6A1	COL6A1	chr21	0.00218144	-1.72796
3924453	NM_001849 // COL6A2 /// NM_058175 // COL6A2 /// NM_058174 // COL6A2 /// ENST0000	COL6A2	chr21	0.027477	-2.29693
3924636	NM_006031 // PCNT /// ENST00000359568 // PCNT /// AB007862 // PCNT	PCNT	chr21	0.0293537	-1.53564
3927491	NM_007038 // ADAMTS5 /// ENST00000284987 // ADAMTS5 /// AF142099 // ADAMTS5	ADAMTS5	chr21	0.0100095	-1.70689
3928562	NM_181602 // KRTAP6-1 /// ENST00000329122 // KRTAP6-1 /// AB096954 // KRTAP6-1	KRTAP6-1	chr21	0.0194127	-1.58962
3930814	NM_017438 // SETD4 /// NM_001007259 // SETD4 /// ENST00000399215 // SETD4 /// EN	SETD4	chr21	0.0332881	-1.5844
3931877	NM_004449 // ERG /// NM_001136154 // ERG /// ENST00000398911 // ERG /// ENST0000	ERG	chr21	0.0125387	-1.73034
3933287	NM_022115 // PRDM15 /// NM_001040424 // PRDM15 /// ENST00000433067 // PRDM15 //	PRDM15	chr21	0.00241856	-1.80098
3933553	NM_005423 // TFF2 /// ENST00000291526 // TFF2 /// BC032820 // TFF2	TFF2	chr21	0.0390182	-1.61198
3934899	NM_015227 // POFUT2 /// NR_004858 // POFUT2 /// NM_133635 // POFUT2 /// ENST0000	POFUT2	chr21	0.0256599	-1.59857
3935024	NM_194255 // SLC19A1 /// ENST00000311124 // SLC19A1 /// U19720 // SLC19A1	SLC19A1	chr21	0.00473606	-1.88962
3937272	NM_013373 // ZDHHC8 /// ENST00000320602 // ZDHHC8 /// AY894890 // ZDHHC8 /// ENS	ZDHHC8	chr22	0.0133188	-1.96808
3938525	ENST00000390306 // IGLV2-23	IGLV2-23	chr22	0.0337251	-1.51815
3939280	NR_033408 // FBXW4P1 /// AK024483 // FBXW4P1	FBXW4P1	chr22	0.0338247	-2.0292
3939515	NM_030807 // SLC2A11 /// NM_001024939 // SLC2A11 /// NM_001024938 // SLC2A11 //	SLC2A11	chr22	0.0263168	-1.58596
3939772	NM_012295 // CABIN1 /// ENST00000263119 // CABIN1 /// ENST00000398319 // CABIN1	CABIN1	chr22	0.0482266	-1.61133
3939779	NM_012295 // CABIN1 /// ENST00000263119 // CABIN1 /// ENST00000398319 // CABIN1	CABIN1	chr22	0.00580542	-1.8232
3940455	NM_004076 // CRYBB3 /// ENST00000215855 // CRYBB3 /// BC102022 // CRYBB3 /// ENS	CRYBB3	chr22	0.00859371	-2.09985
3942942	CR456348 // FLJ20464 /// ENST00000436050 // FLJ20464 /// AK000471 // FLJ20464	FLJ20464	chr22	0.0369191	-1.63528
3943058	ENST00000433295 // SFI1	SFI1	chr22	0.00119595	-1.61301
3943159	NM_014662 // DEPDC5 /// NM_001136029 // DEPDC5 /// ENST00000400249 // DEPDC5 //	DEPDC5	chr22	0.0081153	-1.75851
3944820	NM_013365 // GGA1 /// NM_001001560 // GGA1 /// NM_001172687 // GGA1 /// NM_00117	GGA1	chr22	0.0171817	-1.76003
3946305	NM_001162501 // TNRC6B /// NM_015088 // TNRC6B /// NM_001024843 // TNRC6B /// AK	TNRC6B	chr22	0.00708221	-1.86355
3946364	NM_000026 // ADSL /// NM_001123378 // ADSL /// ENST00000216194 // ADSL /// ENST0	ADSL	chr22	0.0171376	-1.58304
3946409	NM_015705 // SGSM3 /// ENST00000248929 // SGSM3 /// AY308849 // SGSM3 /// AB2757	SGSM3	chr22	0.0303801	-1.57194
3946511	NM_022098 // XPNPEP3 /// ENST00000357137 // XPNPEP3 /// ENST00000414396 // XPNPE	XPNPEP3	chr22	0.00667079	-1.7258
3948974	NM_005036 // PPARA /// ENST00000407236 // PPARA /// AK289821 // PPARA /// ENST00	PPARA	chr22	0.00174493	-1.96443
3948997	NM_001001928 // PPARA /// NM_005036 // PPARA /// ENST00000262735 // PPARA /// EN	PPARA	chr22	0.000543343	-2.25239
3950585	NM_018995 // MOV10L1 /// NM_001164105 // MOV10L1 /// NM_001164106 // MOV10L1 //	MOV10L1	chr22	0.00546845	-1.682
3950659	NM_025204 // TRABD /// ENST00000380909 // TRABD /// ENST00000303434 // TRABD //	TRABD	chr22	0.030788	-1.88137
3950943	NM_138433 // KLHDC7B /// BC009980 // KLHDC7B /// ENST00000252783 // KLHDC7B ///	KLHDC7B	chr22	0.0414416	-1.59663
3951789	CR456417 // CECR1 /// AK292689 // CECR1 /// AK295174 // CECR1 /// AK304818 // CE	CECR1	chr22	0.0361605	-1.56252
3952389	NM_016335 // PRODH /// ENST00000357068 // PRODH /// ENST00000420436 // PRODH //	PRODH	chr22	0.0421653	-2.2049
3952845	NM_053004 // GNB1L /// ENST00000329517 // GNB1L /// ENST00000403325 // GNB1L //	GNB1L	chr22	0.0465596	-1.5278
3953867	NM_030573 // THAP7 /// NM_001008695 // THAP7 /// ENST00000215742 // THAP7 /// EN	THAP7	chr22	0.0213351	-2.1191
3954306	NM_014634 // PPM1F /// ENST00000263212 // PPM1F /// ENST00000406981 // PPM1F //	PPM1F	chr22	0.0108929	-1.65471
3954528	NM_080764 // ZNF280B /// ENST00000406426 // ZNF280B /// ENST00000360412 // ZNF28	ZNF280B	chr22	0.012346	-1.62397
3955115	NM_000853 // GSTT1 /// ENST00000248935 // GSTT1 /// BT019951 // GSTT1 /// BC0070	GSTT1	chr22	0.0271067	-1.63824
3955366	ENST00000446942 // C22orf36 /// AK097054 // C22orf36	C22orf36	chr22	0.0164627	-1.61101
3955648	NM_182492 // LRP5L /// ENST00000215872 // LRP5L /// BC137460 // LRP5L	LRP5L	chr22	0.0294285	-1.58127
3955828	NM_022081 // HPS4 /// NM_152841 // HPS4 /// ENST00000398145 // HPS4 /// ENST0000	HPS4	chr22	0.0330216	-1.51515
3955908	NM_001008697 // TFIP11 /// NM_012143 // TFIP11 /// ENST00000407690 // TFIP11 //	TFIP11	chr22	0.0179899	-1.64223

3956260	NM_002430 // MN1 /// ENST00000302326 // MN1	MN1	chr22	0.0128694	-1.62117
3957044	NM_032204 // ASCC2 /// ENST00000307790 // ASCC2 /// ENST00000397771 // ASCC2 //.	ASCC2	chr22	0.0232488	-1.52926
3957439	NM_004861 // GAL3ST1 /// ENST00000401975 // GAL3ST1 /// ENST00000406361 // GAL3S	GAL3ST1	chr22	0.0386669	-1.66466
3958064	AK294232 // C22orf24 /// NM_015372 // C22orf24 /// ENST00000248984 // C22orf24	C22orf24	chr22	0.000524757	-1.5053
3958327	---	0	chr22	0.0164758	-2.06888
3959222	NM_001031695 // RBM9 /// NM_014309 // RBM9 /// NM_001082576 // RBM9 /// NM_0010ε	RBM9	chr22	0.0206813	-1.63709
3959692	---	0	chr22	0.00397187	-2.11021
3960009	NM_031910 // C1QTNF6 /// ENST00000337843 // C1QTNF6	C1QTNF6	chr22	0.000693397	-1.815
3960151	NM_014550 // CARD10 /// ENST00000403299 // CARD10 /// ENST00000251973 // CARD10	CARD10	chr22	0.0482574	-1.7749
3960835	NM_015374 // SUN2 /// ENST00000405510 // UNC84B /// ENST00000216064 // UNC84B /.	SUN2	chr22	0.0134985	-1.5018
3962862	NM_173050 // SCUBE1 /// ENST00000360835 // SCUBE1 /// AF525689 // SCUBE1 /// ENS	SCUBE1	chr22	0.00517273	-2.42049
3963323	NM_032287 // LDOC1L /// ENST00000341255 // LDOC1L /// BC030232 // LDOC1L	LDOC1L	chr22	0.0461099	-1.64354
3965407	NM_024105 // ALG12 /// ENST00000330817 // ALG12 /// ENST00000332276 // ALG12	ALG12	chr22	0.0480785	-2.37648
3965839	NM_002972 // SBF1 /// ENST00000380817 // SBF1 /// BC087612 // SBF1 /// ENST0000C	SBF1	chr22	0.0368025	-1.53843
3965947	NM_033200 // LMF2 /// ENST00000216080 // LMF2 /// BC014652 // LMF2 /// ENST0000C	LMF2	chr22	0.0103553	-1.53863
3966152	NM_000487 // ARSA /// NM_001085425 // ARSA /// NM_001085426 // ARSA /// NM_0010ε	ARSA	chr22	0.0452875	-1.62594
3966166	NM_000487 // ARSA /// NM_001085425 // ARSA /// ENST00000395621 // ARSA /// ENSTC	ARSA	chr22	0.0389757	-2.28437
3968351	NM_001649 // SHROOM2 /// ENST00000380913 // SHROOM2 /// BC140866 // SHROOM2 ///	SHROOM2	chrX	0.0105813	-1.99571
3969048	NM_001039091 // PRPS2 /// NM_002765 // PRPS2 /// ENST00000380668 // PRPS2 /// EN	PRPS2	chrX	0.0399311	-1.62552
3970148	NR_033181 // SYAP1 /// NM_032796 // SYAP1 /// ENST00000380155 // SYAP1 /// AF16ε	SYAP1	chrX	0.0342793	-1.59634
3972727	NM_014271 // IL1RAPL1 /// ENST00000378993 // IL1RAPL1 /// ENST00000302196 // IL1	IL1RAPL1	chrX	0.0123736	-1.60319
3974799	NM_001039590 // USP9X /// NM_001039591 // USP9X /// ENST00000324545 // USP9X //	USP9X	chrX	0.0446361	-1.7447
3976184	NM_004651 // USP11 /// ENST00000218348 // USP11 /// ENST00000377107 // USP11 //.	USP11	chrX	0.00391239	-1.68722
3976667	NM_022825 // PORCN /// NM_203473 // PORCN /// NM_203474 // PORCN /// NM_203475 .	PORCN	chrX	0.00296345	-1.71849
3976792	NM_000377 // WAS /// ENST00000376701 // WAS /// U12707 // WAS	WAS	chrX	0.0378401	-1.99128
3977055	NM_024859 // MAGIX /// NM_001099680 // MAGIX /// NM_001099681 // MAGIX /// NM_0(	MAGIX	chrX	0.00891983	-1.56628
3979921	NM_000044 // AR /// ENST00000374690 // AR /// M23263 // AR	AR	chrX	2.07911E-05	-1.64178
3980859	NM_181303 // NLGN3 /// NM_018977 // NLGN3 /// NM_001166660 // NLGN3 /// ENST000C	NLGN3	chrX	0.000295768	-1.58918
3981029	NM_004606 // TAF1 /// NM_138923 // TAF1 /// ENST00000373790 // TAF1 /// ENST000C	TAF1	chrX	0.0246376	-1.6285
3981594	NM_005193 // CDX4 /// ENST00000373514 // CDX4 /// BC128233 // CDX4	CDX4	chrX	0.0259552	-1.563
3984471	NM_014467 // SRPX2 /// ENST00000373004 // SRPX2 /// AF393649 // SRPX2	SRPX2	chrX	0.0171297	-1.87704
3984917	NM_016608 // ARMCX1 /// ENST00000372829 // ARMCX1 /// AB039670 // ARMCX1	ARMCX1	chrX	0.0273185	-1.68893
3988578	NM_001560 // IL13RA1 /// ENST00000371666 // IL13RA1 /// ENST00000371637 // IL13F	IL13RA1	chrX	0.0217046	-1.77535
3988784	NM_145305 // SLC25A43 /// ENST00000217909 // SLC25A43 /// AK303932 // SLC25A43 .	SLC25A43	chrX	0.0187999	-1.62131
3990498	NM_003399 // XPNPEP2 /// ENST00000371106 // XPNPEP2 /// BC126174 // XPNPEP2	XPNPEP2	chrX	0.0141552	-2.50596
3992055	NR_024359 // NCRNA00086	NCRNA00086	chrX	0.0277257	-1.73023
3994459	NM_178124 // CXorf40A /// NM_001171907 // CXorf40A /// AK055235 // CXorf40A	CXorf40A	chrX	0.0290393	-1.93942
3995917	NM_005393 // PLXNB3 /// NM_001163257 // PLXNB3 /// ENST00000361971 // PLXNB3 //	PLXNB3	chrX	0.0406764	-1.51889
3995948	NM_014370 // SRPK3 /// NM_001170760 // SRPK3 /// NM_001170761 // SRPK3 /// ENSTC	SRPK3	chrX	0.00844561	-1.59009
3996386	NM_001183 // ATP6AP1 /// ENST00000369762 // ATP6AP1 /// ENST00000422890 // ATP6A	ATP6AP1	chrX	0.0197841	-1.69995
3996507	NM_017514 // PLXNA3 /// ENST00000369682 // PLXNA3	PLXNA3	chrX	0.0479989	-1.66922
3999190	NM_000273 // GPR143 /// ENST00000380929 // GPR143 /// BC068977 // GPR143	GPR143	chrX	0.00176101	-1.64915
3999672	NM_013427 // ARHGAP6 /// NM_006125 // ARHGAP6 /// ENST00000380718 // ARHGAP6 //.	ARHGAP6	chrX	0.00728013	-1.64718
4001598	NM_000292 // PHKA2 /// ENST00000379942 // PHKA2 /// D38616 // PHKA2	PHKA2	chrX	0.00268266	-1.69895
4005709	NM_004229 // MED14 /// ENST00000324817 // MED14 /// AF304448 // MED14	MED14	chrX	2.98258E-05	-1.77095
4007100	NM_007130 // ZNF41 /// NM_153380 // ZNF41 /// ENST00000377065 // ZNF41 /// ENSTC	ZNF41	chrX	0.0221965	-1.59949
4007595	NM_001032289 // SLC35A2 /// NM_001042498 // SLC35A2 /// ENST00000445167 // SLC35	SLC35A2	chrX	0.0465411	-1.68555
4007832	U66359 // GPKOW	GPKOW	chrX	0.0376328	-1.62855
4007887	NM_006150 // PRICKLE3 /// ENST00000376317 // PRICKLE3 /// BC016856 // PRICKLE3 .	PRICKLE3	chrX	0.0250812	-1.58043
4007921	NM_005183 // CACNA1F /// ENST00000376265 // CACNA1F /// AJ224874 // CACNA1F ///	CACNA1F	chrX	0.0296771	-1.5471
4008020	NM_014009 // FOXP3 /// NM_001114377 // FOXP3 /// ENST00000376207 // FOXP3 /// EN	FOXP3	chrX	0.0265588	-1.57884

4008187	NM_003886 // AKAP4 /// NM_139289 // AKAP4 /// ENST00000358526 // AKAP4 /// ENST(	AKAP4	chrX	0.00153021	-1.70611
4009084	NM_004187 // KDM5C /// NM_001146702 // KDM5C /// ENST00000375401 // KDM5C /// EN	KDM5C	chrX	0.0350415	-1.51918
4009714	NM_004463 // FGD1 /// ENST00000375135 // FGD1 /// U11690 // FGD1	FGD1	chrX	0.00961409	-1.85574
4010907	NM_031206 // LAS1L /// NM_001170649 // LAS1L /// NM_001170650 // LAS1L /// ENST(	LAS1L	chrX	0.0310669	-1.71343
4013386	NM_032121 // MAGT1 /// ENST00000358075 // MAGT1 /// BC063037 // MAGT1 /// BC041(	MAGT1	chrX	0.0062327	-1.54316
4017599	NM_001847 // COL4A6 /// NM_033641 // COL4A6 /// ENST00000372216 // COL4A6 /// EN	COL4A6	chrX	0.0190629	-1.51173
4017802	NM_012282 // KCNE1L /// ENST00000372101 // KCNE1L /// AK223306 // KCNE1L	KCNE1L	chrX	0.0154782	-1.65193
4018492	NM_133265 // AMOT /// ENST00000304758 // AMOT /// AF286598 // AMOT	AMOT	chrX	0.0297484	-1.71241
4021501	NM_004208 // AIFM1 /// NM_145812 // AIFM1 /// NM_145813 // AIFM1 /// NM_0011308-	AIFM1	chrX	0.040842	-1.90343
4022126	NM_018388 // MBNL3 /// NM_133486 // MBNL3 /// NM_001170701 // MBNL3 /// NM_0011	MBNL3	chrX	0.0495505	-1.54346
4024132	NM_005369 // MCF2 /// NM_001171877 // MCF2 /// NM_001171878 // MCF2 /// NM_0011	MCF2	chrX	0.0301234	-1.94235
4026996	NM_005334 // HCFC1 /// ENST00000310441 // HCFC1 /// BC063435 // HCFC1 /// ENST0(	HCFC1	chrX	0.0156882	-1.68402
4027059	NM_004992 // MECP2 /// ENST00000303391 // MECP2 /// GU479943 // MECP2 /// AF1581	MECP2	chrX	0.0387893	-1.51053
4027180	NM_001456 // FLNA /// NM_001110556 // FLNA /// ENST00000369863 // FLNA /// ENST(	FLNA	chrX	0.0368818	-1.5789
4027229	NM_001456 // FLNA /// NM_001110556 // FLNA /// ENST00000369863 // FLNA /// ENST(	FLNA	chrX	0.0105598	-1.53179
4027775	NM_001289 // CLIC2 /// ENST00000369449 // CLIC2 /// AK292785 // CLIC2 /// ENST0(	CLIC2	chrX	0.0154616	-1.52797
4044550	---	0	chr1	0.00211849	-2.08221
4046235	NM_173821 // C2orf85 /// ENST00000343216 // C2orf85 /// AK090909 // C2orf85 ///	C2orf85	chr2	0.00363645	-1.5748
4046483	---	0	chr2	0.0338512	-1.6091
4051739	NM_001033113 // ENTPD8 /// NM_198585 // ENTPD8 /// ENST00000371506 // ENTPD8 //.	ENTPD8	chr9	0.0419932	-1.58945
4053510	NM_032129 // PLEKHN1 /// NM_001160184 // PLEKHN1 /// ENST00000379410 // PLEKHN1	PLEKHN1	chr1	0.0243091	-1.66714

supplemental Table S3. Non-coding RNAs from Discovery for next step in

workflow GROUP 1. noncoding RNA microarry results: Up-regulated

Probe Set ID	Transcript ID(Array Design)	Raw p-value	Fold-Change (T21 vs. Ctrl)
ACA16_st	LINK to snoRNABase:ACA16)"	0.0161397	1.10574
ACA19_st	LINK to snoRNABase:ACA19)"	0.0488238	1.08483
ACA27_x_st	LINK to snoRNABase:ACA27)"	0.0498376	1.10612
ACA33_st	LINK to snoRNABase:ACA33)"	0.0298503	1.1639
ACA4_st	LINK to snoRNABase:ACA4)"	0.0308535	1.12488
ACA54_st	LINK to snoRNABase:ACA54)"	0.0144769	1.11476
ENSG00000199282_st	LINK to EnsEMBL:ENSG00000199282)"	0.00551938	1.12355
ENSG00000199633_st	LINK to EnsEMBL:ENSG00000199633)"	0.000099	1.30231
ENSG00000199783_st	LINK to EnsEMBL:ENSG00000199783)"	0.012856	1.15181
ENSG00000200620_st	LINK to EnsEMBL:ENSG00000200620)"	0.043115	1.08742
ENSG00000201551_st	LINK to EnsEMBL:ENSG00000201551)"	0.0306353	1.13479
ENSG00000201898_x_st	LINK to EnsEMBL:ENSG00000201898)"	0.022466	1.17744
ENSG00000201980_st	LINK to EnsEMBL:ENSG00000201980)"	0.00210067	1.16939
ENSG00000202231_x_st	LINK to EnsEMBL:ENSG00000202231)"	0.00442109	1.15569
ENSG00000206853_st	LINK to EnsEMBL:ENSG00000206853)"	0.0497243	1.12239
ENSG00000206976_st	LINK to EnsEMBL:ENSG00000206976)"	0.015575	1.12188
ENSG00000207147_st	LINK to EnsEMBL:ENSG00000207147)"	0.00851488	1.18521
ENSG00000212277_st	LINK to EnsEMBL:ENSG00000212277)"	0.0427717	1.12722
ENSG00000212342_st	LINK to EnsEMBL:ENSG00000212342)"	0.017189	1.13696
ENSG00000212363_st	LINK to EnsEMBL:ENSG00000212363)"	0.00433048	1.14444
ENSG00000212581_st	LINK to EnsEMBL:ENSG00000212581)"	0.0486271	1.12901
HBII-276_st	LINK to snoRNABase:HBII-276)"	0.00844437	1.13068
HBII-52-41_x_st	LINK to snoRNABase:HBII-52-41)"	0.0163907	1.11431
HBII-52-5_x_st	LINK to snoRNABase:HBII-52-5)"	0.0284517	1.14086
HBII-85-3_x_st	LINK to snoRNABase:HBII-85-3)"	0.0223134	1.13418
hsa-let-7d_st	LINK to miRBase:hsa-let-7d)"	0.0079206	1.30382
hsa-miR-1286_st	LINK to miRBase:hsa-mir-1286)"	0.000265011	1.29375
hsa-miR-148b-star_st	LINK to miRBase:hsa-mir-148b)"	0.0474602	1.22081
hsa-miR-151-5p_st	LINK to miRBase:hsa-mir-151)"	0.0364688	1.11986
hsa-miR-181b_st	LINK to miRBase:hsa-mir-181b-1)"	0.0473221	1.16141
hsa-miR-185-star_st	LINK to miRBase:hsa-mir-185)"	0.0235802	1.17568
hsa-miR-22-star_st	LINK to miRBase:hsa-mir-22)"	0.0367718	1.21361
hsa-miR-331-5p_st	LINK to miRBase:hsa-mir-331)"	0.0493594	1.22856
hsa-miR-335_st	LINK to miRBase:hsa-mir-335)"	0.042215	1.32246
hsa-miR-499-3p_st	LINK to miRBase:hsa-mir-499)"	0.00818447	1.2299
hsa-miR-541_st	LINK to miRBase:hsa-mir-541)"	0.0332965	1.19625

hsa-miR-548l_st	LINK to miRBase:hsa-mir-548l)"	0.0059975	1.231
hsa-miR-548m_st	LINK to miRBase:hsa-mir-548m)"	0.0494985	1.1709
hsa-miR-591_st	LINK to miRBase:hsa-mir-591)"	0.0109054	1.13852
hsa-miR-654-3p_st	LINK to miRBase:hsa-mir-654)"	0.0288782	1.2714
hsa-miR-933_st	LINK to miRBase:hsa-mir-933)"	0.0202272	1.39658
hsa-miR-99b-star_st	LINK to miRBase:hsa-mir-99b)"	0.026711	1.22661
mgh28S-2409_st	LINK to snoRNABase:mgh28S-2409)"	0.0267346	1.10388
U108_x_st	LINK to snoRNABase:U108)"	0.0455585	1.18615
U44_st	LINK to snoRNABase:U44)"	0.0310674	1.14249
U49B_x_st	LINK to snoRNABase:U49B)"	0.0355	1.12667
U71b_st	LINK to snoRNABase:U71b)"	0.0484609	1.17112
U93_st	LINK to snoRNABase:U93)"	0.0312531	1.12552
U95_st	LINK to snoRNABase:U95)"	0.0160735	1.12758
U99_st	LINK to snoRNABase:U99)"	0.0468811	1.13188

GROUP 1. noncoding RNA microarry results: Down-regulated

Probe Set ID	Transcript ID(Array Design)	p-value	Fold-Change (T21 vs. Ctrl)
14qII-4_st	LINK to snoRNABase:14q(II-4))"	0.0283627	-1.126
ACA11_st	LINK to snoRNABase:ACA11)"	0.0264542	-1.14069
ACA26_st	LINK to snoRNABase:ACA26)"	0.0269577	-1.20014
ENSG00000199856_st	LINK to EnsEMBL:ENSG00000199856)"	0.0430042	-1.12167
ENSG00000199856_x_st	LINK to EnsEMBL:ENSG00000199856)"	0.0091478	-1.13078
ENSG00000201791_x_st	LINK to EnsEMBL:ENSG00000201791)"	0.0267029	-1.14915
ENSG00000202440_x_st	LINK to EnsEMBL:ENSG00000202440)"	0.0352478	-1.14053
ENSG00000207217_st	LINK to EnsEMBL:ENSG00000207217)"	0.0291033	-1.14222
ENSG00000212528_st	LINK to EnsEMBL:ENSG00000212528)"	0.0234352	-1.11412
ENSG00000212551_st	LINK to EnsEMBL:ENSG00000212551)"	0.0343054	-1.1303
ENSG00000212604_x_st	LINK to EnsEMBL:ENSG00000212604)"	0.0382121	-1.13849
HBII-438A_s_st	LINK to snoRNABase:HBII-438A)"	0.013594	-1.14261
HBII-52-21_x_st	LINK to snoRNABase:HBII-52-21)"	0.0264105	-1.11103
HBII-85-4_x_st	LINK to snoRNABase:HBII-85-4)"	0.0437779	-1.14266
hsa-miR-1253_st	LINK to miRBase:hsa-mir-1253)"	0.0445783	-1.13437
hsa-miR-1277_st	LINK to miRBase:hsa-mir-1277)"	0.0101794	-1.19411
hsa-miR-129-3p_st	LINK to miRBase:hsa-mir-129-2)"	0.0105544	-1.28449
hsa-miR-148b_st	LINK to miRBase:hsa-mir-148b)"	0.0496496	-1.1608
hsa-miR-186-star_st	LINK to miRBase:hsa-mir-186)"	0.0482628	-1.26223
hsa-miR-216b_st	LINK to miRBase:hsa-mir-216b)"	0.00204849	-1.29243
hsa-miR-26b_st	LINK to miRBase:hsa-mir-26b)"	0.00130882	-1.27088

hsa-miR-30d-star_st	LINK to miRBase:hsa-mir-30d)"	0.0492595	-1.15544
hsa-miR-31_st	LINK to miRBase:hsa-mir-31)"	0.0181009	-1.24627
hsa-miR-376a_st	LINK to miRBase:hsa-mir-376a-2)"	0.0088482	-1.1976
hsa-miR-432_st	LINK to miRBase:hsa-mir-432)"	0.0195265	-1.26277
hsa-miR-450b-5p_st	LINK to miRBase:hsa-mir-450b)"	0.00710121	-1.22073
hsa-miR-493-star_st	LINK to miRBase:hsa-mir-493)"	0.0457028	-1.15138
hsa-miR-505_st	LINK to miRBase:hsa-mir-505)"	0.0334433	-1.21463
hsa-miR-516a-3p_st	LINK to miRBase:hsa-mir-516a-1)"	0.0242036	-1.13252
hsa-miR-520c-5p_st	LINK to miRBase:hsa-mir-520c)"	0.00515938	-1.22238
hsa-miR-523-star_st	LINK to miRBase:hsa-mir-523)"	0.00861596	-1.19767
hsa-miR-543_st	LINK to miRBase:hsa-mir-543)"	0.0482199	-1.26569
hsa-miR-548i_st	LINK to miRBase:hsa-mir-548i-1)"	0.0235467	-1.20759
hsa-miR-548p_st	LINK to miRBase:hsa-mir-548p)"	0.0250089	-1.17659
hsa-miR-550_st	LINK to miRBase:hsa-mir-550-1)"	0.0166807	-1.29982
hsa-miR-567_st	LINK to miRBase:hsa-mir-567)"	0.0443335	-1.16603
hsa-miR-568_st	LINK to miRBase:hsa-mir-568)"	0.0359771	-1.18586
hsa-miR-569_st	LINK to miRBase:hsa-mir-569)"	0.00228971	-1.34492
hsa-miR-581_st	LINK to miRBase:hsa-mir-581)"	0.0018385	-1.26672
hsa-miR-607_st	LINK to miRBase:hsa-mir-607)"	0.020689	-1.20511
hsa-miR-877-star_st	LINK to miRBase:hsa-mir-877)"	0.042145	-1.31816
hsa-miR-93_st	LINK to miRBase:hsa-mir-93)"	0.00263873	-1.19393
hsa-miR-937_st	LINK to miRBase:hsa-mir-937)"	0.0142397	-1.20015
hsa-miR-944_st	LINK to miRBase:hsa-mir-944)"	0.0278583	-1.25927
hsa-miR-98_st	LINK to miRBase:hsa-mir-98)"	0.00841137	-1.28828
U105_st	LINK to snoRNABase:U105)"	0.0431998	-1.10671
U19-2_st	LINK to snoRNABase:U19-2)"	0.0396618	-1.13734
U28_st	LINK to snoRNABase:U28)"	0.0373596	-1.12887

supplemental table S4. Correlation matrix

	VAR.1	VAR.2	VAR.3	VAR.4	VAR.5	VAR.6	VAR.7	VAR.8	VAR.9	VAR.10	VAR.11	VAR.12	VAR.13	VAR.14	VAR.15	VAR.16	VAR.17	VAR.18	VAR.19	VAR.20	VAR.21	VAR.22	VAR.23	VAR.24
VAR.2	1																							
VAR.3	0.96	0.96																						
VAR.4	0.84	0.84	0.86																					
VAR.5	1	1	0.96	0.84																				
VAR.6	1	1	0.96	0.84	1																			
VAR.7	1	1	0.97	0.84	1	1																		
VAR.8	-0.01	-0.01	0.01	0.06	-0.01	-0.1	0																	
VAR.9	1	1	0.96	0.84	1	1	1	-0.01																
VAR.10	0.65	0.84	0.72	0.53	0.85	0.65	0.69	0.08	0.65															
VAR.11	-0.03	-0.03	0.02	0.28	-0.03	-0.03	-0.04	-0.05	-0.03	-0.07														
VAR.12	-0.01	-0.01	-0.02	-0.02	-0.01	-0.01	-0.01	-0.08	-0.01	-0.01	-0.01													
VAR.13	-0.07	-0.07	-0.03	-0.1	-0.07	-0.07	-0.02	-0.01	-0.07	0.4	0.01	0.02												
VAR.14	-0.01	-0.01	-0.02	-0.02	-0.01	-0.01	-0.01	-0.01	-0.01	-0.01	-0.01	1	0.02											
VAR.15	0.1	0.1	0.12	0.12	0.11	0.1	0.14	-0.01	0.1	0.41	-0.07	0.11	0.79	0.12										
VAR.16	-0.01	0.01	-0.02	-0.02	-0.01	-0.01	-0.01	-0.01	-0.01	-0.01	-0.01	1	0.01	1	0.1									
VAR.17	-0.01	-0.01	-0.02	-0.02	-0.01	-0.01	-0.01	-0.01	-0.01	0	-0.01	1	0.03	1	0.12	1								
VAR.18	-0.09	-0.09	-0.03	-0.1	-0.08	-0.09	-0.09	0.01	-0.08	0.26	-0.06	0.21	0.76	0.21	0.78	0.19	0.21							
VAR.19	-0.01	-0.01	-0.02	0.01	-0.01	-0.01	-0.01	0.01	-0.01	-0.02	0.03	-0.01	-0.01	-0.01	-0.01	-0.01	-0.01	-0.03						
VAR.20	-0.05	-0.05	-0.06	-0.06	-0.05	-0.05	-0.05	-0.05	-0.05	-0.05	-0.01	0	0.02	-0.01	0.01	-0.01	-0.01	0.01	-0.03					
VAR.21	-0.01	-0.01	-0.01	-0.01	-0.01	-0.01	-0.01	0.38	-0.01	-0.01	0.01	0	-0.01	0	-0.02	0	0	0.03	0	-0.01				
VAR.22	-0.02	-0.02	-0.03	-0.03	-0.02	-0.02	-0.02	-0.02	-0.02	-0.03	-0.01	-0.01	-0.02	-0.01	-0.03	-0.01	-0.01	0	-0.01	0.66	0			
VAR.23	0	0	-0.01	-0.01	0	0	0	0.38	0	0	-0.01	0	-0.01	0	-0.01	0	0	0.03	0	-0.02	0.99	-0.01		
VAR.24	0.08	0.08	0.1	0.07	0.08	0.08	0.08	-0.02	0.08	0.08	-0.01	-0.01	-0.01	-0.01	-0.02	-0.01	-0.01	0	-0.02	-0.07	-0.01	-0.02	-0.02	
VAR.25	-0.04	-0.04	-0.05	-0.04	-0.04	-0.04	-0.04	-0.02	-0.04	-0.03	0.02	-0.01	0.01	-0.01	0.01	-0.01	-0.01	0	-0.02	0.15	-0.01	-0.02	-0.02	-0.07
VAR.26	-0.01	-0.01	-0.01	-0.02	-0.01	-0.01	-0.01	0.24	-0.01	0	-0.01	0.01	-0.01	-0.01	0.02	-0.01	-0.01	0.01	-0.01	-0.05	-0.01	-0.02	-0.01	-0.04
VAR.27	-0.02	-0.02	-0.03	0.01	-0.02	-0.02	-0.02	-0.03	-0.02	-0.07	0.05	-0.02	-0.01	-0.02	-0.02	-0.02	-0.02	-0.03	0.48	0.13	0	0.03	-0.01	-0.06
VAR.28	0.07	0.07	0.07	0.05	0.07	0.07	0.07	-0.01	0.07	0.04	-0.01	0.15	0	0.16	0.03	0.16	0.16	0.02	-0.01	-0.03	0	-0.01	-0.01	-0.02
VAR.29	0.01	0.01	0	0	0.01	0.01	0.01	-0.02	0.01	-0.01	0.01	-0.01	0	-0.01	0.03	-0.01	-0.01	-0.01	-0.01	-0.05	-0.01	-0.02	-0.01	-0.03
VAR.30	-0.02	-0.02	-0.02	0.01	-0.02	-0.02	-0.02	0.01	-0.02	-0.05	0.04	-0.01	-0.02	-0.01	0	-0.01	-0.01	0	0.01	-0.03	0	-0.01	0	-0.03
VAR.31	0.05	0.05	0.05	0.04	0.05	0.05	0.05	0	0.05	0.05	0	-0.01	-0.01	-0.01	0.03	-0.01	-0.01	-0.02	0.1	-0.05	-0.01	-0.01	-0.01	-0.04
VAR.32	0.04	0.04	0.05	0.03	0.04	0.04	0.04	-0.01	0.04	0.04	0.01	-0.01	-0.01	-0.01	0.02	-0.01	-0.01	0.02	0.02	-0.04	0	-0.02	-0.01	0.11
VAR.33	-0.01	-0.01	0.01	0.03	-0.01	-0.01	-0.02	0.07	-0.01	-0.01	0.06	-0.01	-0.01	-0.01	0.01	-0.01	-0.01	0	-0.02	-0.05	-0.01	-0.01	-0.01	-0.05
VAR.34	-0.03	-0.03	0	0.12	-0.03	-0.03	-0.03	-0.03	-0.03	-0.05	0.39	-0.01	-0.01	-0.01	-0.04	0	-0.01	-0.01	-0.02	-0.01	0.02	-0.02	-0.01	-0.04
VAR.35	-0.03	-0.03	-0.04	-0.01	-0.03	-0.03	-0.03	-0.05	-0.03	-0.02	0	0.07	0.07	0.07	0.06	0.07	0.07	0.07	-0.03	0.48	0.02	0.32	0.01	-0.17
VAR.36	0.01	0.01	0.01	0.01	0.01	0.01	0.01	-0.05	0.01	0.01	0	-0.02	-0.01	-0.01	-0.02	-0.01	-0.01	-0.01	-0.06	0.38	-0.01	0.02	-0.01	0.09
VAR.37	-0.01	-0.01	0.02	0.17	-0.01	-0.01	-0.01	-0.01	-0.01	-0.02	0.45	0	0	0	-0.02	0	0	-0.02	0	0	0.02	-0.01	0	-0.01
VAR.38	-0.02	-0.02	-0.03	-0.02	-0.02	-0.02	-0.02	-0.02	-0.02	-0.03	0	0	-0.01	-0.01	-0.02	0	-0.01	0	-0.01	0.67	0	1	-0.01	-0.02
VAR.39	0	0	-0.01	0	0	0	0	0.04	0	-0.01	0.01	-0.01	-0.01	-0.01	0	-0.01	-0.01	-0.01	0.84	-0.05	0	-0.01	0.02	-0.04
VAR.40	-0.01	-0.01	-0.02	0.02	-0.01	-0.01	-0.01	0	-0.01	-0.02	-0.01	0	0	0	-0.01	0	0	-0.01	-0.01	-0.02	0	0	0	-0.01
VAR.41	0.04	0.04	0.03	0.05	0.04	0.04	0.04	0.01	0.04	0	0.02	-0.01	-0.01	-0.01	0	-0.01	-0.01	-0.04	0.93	-0.03	0	-0.01	0	-0.03
VAR.42	-0.01	-0.01	-0.02	-0.03	-0.01	-0.01	-0.02	0.31	-0.01	-0.03	-0.01	-0.01	-0.01	-0.01	-0.03	-0.01	-0.01	-0.01	0.02	-0.07	0.85	-0.02	0.87	-0.06
VAR.43	-0.02	-0.02	-0.01	-0.01	-0.02	-0.02	-0.02	-0.02	-0.02	-0.03	0.03	0	0	0	-0.04	0	0	-0.03	0.01	-0.02	0	-0.01	-0.01	-0.02
VAR.44	-0.02	-0.02	-0.04	-0.01	-0.02	-0.02	-0.02	0.01	-0.02	-0.03	0.03	0	0	0	0	0	0	-0.02	0.58	-0.04	0	-0.01	0	-0.03
VAR.45	-0.03	-0.03	-0.04	-0.04	-0.03	-0.03	-0.03	0.19	-0.03	-0.04	-0.01	-0.01	-0.01	-0.01	-0.04	-0.01	-0.01	0	-0.01	-0.05	-0.01	-0.01	-0.01	-0.04
VAR.46	-0.03	-0.03	-0.04	-0.03	-0.03	-0.03	-0.03	-0.01	-0.03	-0.02	0	-0.01	-0.01	-0.01	0.01	-0.01	-0.01	0.01	-0.01	-0.04	0	-0.01	0	-0.04
VAR.47	-0.01	-0.01	0.02	0.15	-0.01	-0.01	-0.01	-0.02	-0.01	-0.01	0.4	-0.01	-0.01	-0.01	0	-0.01	-0.01	-0.02	0	-0.01	0.02	-0.01	-0.01	-0.03
VAR.48	-0.01	-0.01	-0.01	-0.01	-0.01	-0.01	-0.01	-0.01	-0.01	-0.02	0	-0.01	-0.01	-0.01	-0.01	-0.01	-0.01	0	-0.01	0.03	0	-0.01	0	-0.03
VAR.49	0.11	0.1	0.1	0.11	0.11	0.11	0.1	0	0.11	0.04	0.01	-0.01	-0.01	-0.01	0.02	-0.01	-0.01	0.01	-0.01	-0.04	0	-0.01	-0.01	-0.04
VAR.50	-0.01	-0.01	-0.01	-0.02	-0.01	-0.01	-0.02	-0.02	-0.01	-0.03	-0.01	0	0	0	-0.04	0	0	-0.03	-0.01	-0.02	0	-0.01	0	-0.02
VAR.51	-0.01	-0.01	-0.02	-0.02	-0.01	-0.01	-0.02	-0.04	-0.01	-0.02	-0.01	-0.02	-0.02	-0.02	-0.03	-0.02	-0.02	-0.06	-0.04	-0.13	-0.01	-0.04	-0.03	-0.11
VAR.52	-0.04	-0.04	-0.04	0	-0.03	-0.03	-0.03	0.01	-0.04	0.01	0	-0.01	0	-0.01	0	-0.01	-0.01	0.01	0	-0.06	-0.01	-0.02	-0.01	-0.05
VAR.53	-0.02	-0.02	-0.01	0.08	-0.02	-0.02	-0.02	0	-0.02	-0.05	0.15	-0.01	-0.01	-0.01	-0.01	-0.01	-0.01	-0.01	0.08	-0.04	0	-0.01	-0.01	-0.04
VAR.54	-0.01	-0.01	-0.02	0.17	-0.01	-0.01	-0.01	0	-0.01	-0.02	0.45	0	0.04	0	-0.02	0	0	-0.02	-0.01	0	0.05	-0.01	0.02	-0.01

VAR.25	VAR.26	VAR.27	VAR.28	VAR.29	VAR.30	VAR.31	VAR.32	VAR.33	VAR.34	VAR.35	VAR.36	VAR.37	VAR.38	VAR.39	VAR.40	VAR.41	VAR.42	VAR.43	VAR.44	VAR.45	VAR.46	VAR.47	VAR.48
0.13																							
0.02	-0.05																						
-0.02	-0.02	0.13																					
-0.04	-0.01	0.1	-0.01																				
-0.03	-0.02	0.71	-0.01	-0.02																			
-0.04	-0.03	0.01	-0.01	-0.03	-0.01																		
-0.03	-0.02	0.23	0.41	0.04	0.28	-0.03																	
0.18	0.35	-0.04	-0.02	-0.03	0	-0.03	-0.01																
0.03	-0.03	-0.01	-0.01	-0.03	-0.01	-0.02	-0.03	-0.03															
0.07	-0.08	0.33	-0.01	-0.12	0.27	-0.07	0.03	-0.11	-0.03														
0.12	-0.11	0.25	-0.04	0.05	0.06	0.03	0	0.05	-0.11	0.58													
0.07	-0.01	0.03	0	-0.01	0.01	0	0	-0.01	0.88	0.03	0												
-0.02	-0.01	0.04	-0.01	-0.02	-0.01	-0.01	-0.02	-0.02	-0.01	0.33	0.02	0											
-0.04	-0.03	0.41	-0.01	-0.02	-0.02	0.06	-0.03	-0.02	-0.02	0.06	0.17	-0.01	-0.01										
0.79	-0.01	-0.02	0	-0.01	-0.01	-0.01	-0.01	-0.01	-0.01	-0.02	-0.02	0.02	0	-0.01									
-0.02	-0.02	0.43	0	-0.01	-0.01	0.08	-0.02	-0.01	-0.01	-0.07	-0.09	0	-0.01	0.78	0								
0.01	-0.04	-0.05	-0.02	-0.04	-0.01	-0.04	-0.04	-0.04	-0.03	-0.11	-0.11	-0.01	-0.02	0	-0.01	0.01							
0.01	-0.01	0.36	-0.01	-0.01	-0.01	-0.01	-0.01	-0.01	0.07	-0.03	-0.06	0.08	-0.01	0.01	0	0.03	-0.02						
-0.03	0.01	0.42	-0.01	-0.01	-0.01	0.07	-0.02	-0.01	-0.02	-0.08	-0.08	-0.01	-0.01	0.75	-0.01	0.83	0.02	0.01					
-0.04	-0.03	0.03	-0.01	-0.02	0.05	-0.02	-0.01	0	-0.02	-0.04	0.01	-0.01	-0.01	0.06	-0.01	-0.02	-0.04	-0.01	-0.02				
-0.03	-0.02	-0.01	-0.01	-0.02	-0.01	-0.02	-0.02	-0.03	-0.02	-0.04	-0.08	-0.01	-0.01	-0.02	-0.01	-0.01	-0.03	-0.01	0.39	-0.02			
0.05	-0.02	0.07	-0.01	-0.02	0.02	0.2	-0.02	-0.02	0.79	0	-0.02	0.8	-0.01	-0.01	-0.01	0	-0.02	0.17	0.15	-0.02	0.32		
0.01	-0.01	0	-0.01	-0.02	-0.01	-0.02	-0.02	0.08	-0.02	0.03	0.04	-0.01	-0.01	-0.01	-0.01	-0.01	-0.02	-0.01	-0.01	-0.02	-0.01	-0.01	
-0.03	-0.02	-0.05	-0.01	-0.02	-0.01	-0.02	-0.03	0.09	0	-0.11	-0.08	0.02	-0.01	-0.02	-0.01	0.03	-0.03	-0.01	-0.01	-0.02	-0.02	0.01	0.05
0.01	-0.01	0.35	-0.01	-0.01	-0.01	-0.01	-0.01	-0.01	0.01	-0.03	-0.05	0.01	-0.01	-0.01	0	0.01	-0.02	1	-0.01	-0.01	-0.01	0.11	-0.01
-0.1	-0.07	-0.14	-0.04	-0.07	-0.05	-0.07	-0.08	-0.07	-0.06	-0.28	-0.21	-0.02	-0.04	-0.07	-0.02	-0.04	0.44	-0.02	-0.02	-0.06	-0.06	-0.05	-0.04
-0.05	-0.03	-0.02	-0.02	-0.03	-0.02	-0.03	-0.03	-0.04	-0.03	-0.03	-0.06	-0.01	-0.02	-0.01	-0.01	0	-0.05	-0.01	0.32	-0.03	0.85	0.25	-0.02
-0.01	-0.02	0.73	0.07	-0.02	0.96	-0.01	0.3	-0.01	0.21	0.28	0.05	0.25	-0.01	0.04	-0.01	0.06	-0.01	0.01	0.05	0.04	-0.02	0.24	-0.01
0.06	-0.01	0.03	0	-0.01	0.01	0	-0.01	-0.01	0.88	0.03	0	1	0	-0.01	0	0	0.01	0.07	-0.01	0	-0.01	0.9	0

VAR.49	VAR.50	VAR.51	VAR.52	VAR.53
-0.01				
-0.06	-0.02			
-0.03	-0.01	-0.02		
-0.01	-0.01	-0.04	-0.02	
0.02	0	-0.02	-0.01	0.25

supplemental Table S5. Machine Learning dataset

GROUP	Ethnicity	GA (weeks)	MA (years)	Height (cm)	Weight (kg)	1<br>ENSG00000199633 F2	2<br>ENSG00000207147 F2	3<br>hsa-let-7d F1	4<br>hsa-mir-569 F1
a-T21	White	13.3	40.7	165.1	63	5.149749697	91.20545006	38.88911341	0.033011786
a-T21	White	13.3	44.2	168	65	1.337393805	12.86655969	159.4393552	14.83386403
a-T21	White	13.8	40.6	157.5	44.5	2.807920933	1.380988875	732.3489923	1583.831318
a-T21	White	12.1	41.3	165.1	72	2.547632095	0.145788477	0.000866812	0.045665567
a-T21	White	13.3	27.8	170	76	0.826641584	7.490897727	44.37680926	58.0515879
a-T21	White	11.5	36.5	170.2	76	0.380202563	98.14087356	13.26077713	3.556822003
a-T21	White	12.7	36.7	170.2	63	1.971915332	9.952332303	176.845314	0.010668025
a-T21	White	12.8	42.9	177.8	65	5.149749697	91.20545006	38.88911341	0.033011786
a-T21	White	12.5	40.6	157.5	72	0.128453702	5.22394108	103.3743204	0.003843553
a-T21	White	13	28.8	172.7	79	1.280571903	179.1512944	59.43571166	1.355912071
a-T21	White	13.8	36.8	172.7	82	2.547632095	0.145788477	0.000866812	0.045665567
a-T21	White	12.5	40.8	175.3	84	1.971915332	9.952332303	176.845314	0.010668025
a-T21	White	12.7	44.8	165.1	61.7	5.149749697	91.20545006	38.88911341	0.033011786
a-T21	White	13.4	37	162.6	63	5.149749697	91.20545006	38.88911341	0.033011786
a-T21	White	13.8	40.3	164	63	2.547632095	0.145788477	0.000866812	0.045665567
a-T21	White	12.7	34.6	182.9	91	0.101169068	0.787299745	22.59252012	0.009481569
a-T21	White	13.1	38.7	170.2	61.7	2.807920933	1.380988875	732.3489923	1583.831318
a-T21	White	12.9	41.2	166	63	0.128453702	5.22394108	103.3743204	0.003843553
a-T21	White	13.2	32	165.1	59	2.807920933	1.380988875	732.3489923	1583.831318
a-T21	White	13.1	33.1	165.1	67	1.895837563	18.00034565	615.0526601	753.0575435
a-T21	White	14	38	167.6	79	5.149749697	91.20545006	38.88911341	0.033011786
a-T21	White	12.5	35.5	157.5	47	5.149749697	91.20545006	38.88911341	0.033011786
a-T21	White	12.5	40.2	162.6	64	0.444417463	7.110912269	94.79466978	7.487255017
a-T21	White	12.8	33.4	175.3	71	0.051096337	0.223756268	40.68198553	0.018190659
a-T21	White	14	31	175.3	86	0.128453702	5.22394108	103.3743204	0.003843553
a-T21	White	12.7	36.2	152	53.9	0.101169068	0.787299745	22.59252012	0.009481569
a-T21	White	13.7	32.8	175.3	71	2.547632095	0.145788477	0.000866812	0.045665567
a-T21	White	12.5	41.6	162.6	99.2	0.00583987	0.14777394	3.559133681	0.248217966
a-T21	White	12.2	41.6	162.6	58	0.051096337	0.223756268	40.68198553	0.018190659
a-T21	White	12.3	37.4	160	58	0.051096337	0.223756268	40.68198553	0.018190659
a-T21	White	12.9	40.1	162	55.4	0.213977798	0.824085688	6.741207665	0.019926533
a-T21	White	14.1	37	157	86	0.826641584	7.490897727	44.37680926	58.0515879
a-T21	White	13.8	43.4	149.9	60.4	0.380202563	98.14087356	13.26077713	3.556822003
a-T21	White	12.1	42.1	162.6	69	0.051096337	0.223756268	40.68198553	0.018190659
a-T21	White	13.6	37.9	160	63	0.380202563	98.14087356	13.26077713	3.556822003
a-T21	White	11.3	35.5	164	60.5	0.277389948	0.245824685	0.464427871	52.86075039
a-T21	White	12.3	26.4	169	59	0.380202563	98.14087356	13.26077713	3.556822003

a-T21	White	12.8	31.7	162	60	1.895837563	18.00034565	615.0526601	753.0575435
a-T21	White	13.9	37	162.6	65	1.247712647	17.15095015	67.94566125	0.046686837
a-T21	White	13.8	36.3	165	65.6	0.213977798	0.824085688	6.741207665	0.019926533
a-T21	White	12.8	36.1	165	70	0.15856028	2.594442408	21.20784493	0.026211467
a-T21	White	12.8	40	165.1	91.3	1.280571903	179.1512944	59.43571166	1.355912071
a-T21	Black	13.6	39.3	157.5	60	0.00583987	0.14777394	3.559133681	0.248217966
a-T21	Black	13.4	31.4	154	72	0.059561877	2.922528179	8.223613653	0.017331417
a-T21	Black	13.3	38	170.2	76	2.547632095	0.145788477	0.000866812	0.045665567
a-T21	Black	12.2	41.9	160	69.9	2.807920933	1.380988875	732.3489923	1583.831318
a-T21	Black	12.5	39.4	166	74.5	0.051096337	0.223756268	40.68198553	0.018190659
a-T21	Black	14.1	46	151.8	60	1.971915332	9.952332303	176.845314	0.010668025
a-T21	East Asian	12.7	34.9	157.5	63.4	0.795830458	173.9468263	0.001653991	0.738121607
a-T21	East Asian	13.1	38.7	155	64	0.051096337	0.223756268	40.68198553	0.018190659
Normal	White	11.2	21.6	153	53	0.313012978	11.39411208	0.000243309	0.802508159
Normal	White	12.8	29.2	170	57	1.147189178	6.149309885	0.00195811	0.016852717
Normal	White	13.4	22.1	165	58	3.176441134	10.90333478	0.002849085	268.9775465
Normal	White	12.5	33.6	162.6	59	2.169851296	7.166690776	288.5378219	48.60199882
Normal	White	12.8	29.9	172.7	60	0.270972897	0.122640088	12.6595894	0.309648549
Normal	White	13.1	30.9	170	60	4.180572961	6.709695547	253.6370017	134.7686942
Normal	White	13.3	23	157	61	0.618821907	1.282406956	142.5895547	0.252183263
Normal	White	11.3	27.4	165.1	61	0.092415497	0.060841519	6.17E-05	0.00601206
Normal	White	12.4	36.8	169	61.7	1.147189178	6.149309885	0.00195811	0.016852717
Normal	White	12.1	35.5	165	64	0.875915925	0.42339554	164.5471986	0.697413605
Normal	White	12.2	26.9	172.7	64	1.759948264	110.663424	184.3518633	0.629789935
Normal	White	12.8	36.9	168	64	0.618821907	1.282406956	142.5895547	0.252183263
Normal	White	12.5	34.1	170.2	65	0.092415497	0.060841519	6.17E-05	0.00601206
Normal	White	13.9	33	160	66	3.176441134	10.90333478	0.002849085	268.9775465
Normal	White	12.6	23.1	165	68	3.176441134	10.90333478	0.002849085	268.9775465
Normal	White	13.4	27.1	157	70	0.648238947	0.170956069	40.9669193	157.8339256
Normal	White	12.5	34.4	158	70	0.124746359	2.37215778	6.904368641	0.008922686
Normal	White	11.9	31.8	167.6	71	0.092415497	0.060841519	6.17E-05	0.00601206
Normal	White	11.9	27.5	169	71	0.092415497	0.060841519	6.17E-05	0.00601206
Normal	White	14.1	34.1	161	72	0.313012978	11.39411208	0.000243309	0.802508159
Normal	White	13	33.8	172	74	3.295126487	47.53328687	0.380011491	0.334692287
Normal	White	12.9	28	164	75	4.180572961	6.709695547	253.6370017	134.7686942
Normal	White	12.8	38	169	76	4.180572961	6.709695547	253.6370017	134.7686942
Normal	White	12.6	28.8	157.5	76	0.270972897	0.122640088	12.6595894	0.309648549
Normal	White	12.4	29.1	165	76	0.875915925	0.42339554	164.5471986	0.697413605
Normal	White	11.9	29.6	184	79	3.295126487	47.53328687	0.380011491	0.334692287
Normal	White	13.6	37.6	165	87	0.201765231	9.28E-05	3.367934242	1.300935568
Normal	White	13.4	33.7	162.6	54	0.092415497	0.060841519	6.17E-05	0.00601206

Normal	White	13.6	38.4	157	55	0.020311987	0.613446173	2.087025386	0.000275459
Normal	White	12.2	35.9	162.6	55	4.180572961	6.709695547	253.6370017	134.7686942
Normal	White	12.4	35.4	155	55	3.176441134	10.90333478	0.002849085	268.9775465
Normal	White	13.3	36.4	160	55.4	0.020311987	0.613446173	2.087025386	0.000275459
Normal	White	13.3	34.4	164	56	0.176715723	0.002940006	7.642872435	0.000308719
Normal	White	12.8	23.7	164	57	0.648238947	0.170956069	40.9669193	157.8339256
Normal	White	12.8	35.5	160	59	8686.730764	18787.72221	2180.022227	947.1359042
Normal	White	11.7	25.1	156	59	0.176715723	0.002940006	7.642872435	0.000308719
Normal	White	13.6	31.3	157.5	59.3	0.020311987	0.613446173	2.087025386	0.000275459
Normal	White	12.9	31	163	60	8686.730764	18787.72221	2180.022227	947.1359042
Normal	White	13.5	24.6	169	60	0.092415497	0.060841519	6.17E-05	0.00601206
Normal	White	12.5	33.3	160	60	3.295126487	47.53328687	0.380011491	0.334692287
Normal	White	12.7	33.8	169	62	0.313012978	11.39411208	0.000243309	0.802508159
Normal	White	12.8	34.4	160	62	0.313012978	11.39411208	0.000243309	0.802508159
Normal	White	13.3	33.4	159	63	1.147189178	6.149309885	0.00195811	0.016852717
Normal	White	13.5	38.3	170.2	66	1.147189178	6.149309885	0.00195811	0.016852717
Normal	White	12.8	36	172.7	67	4.00861349	0.002557265	0.036603836	0.008711746
Normal	White	12.8	30.1	153	68	0.092415497	0.060841519	6.17E-05	0.00601206
Normal	White	12.6	41.3	164	69	0.72936366	3.388629009	0.000257766	10.84859942
Normal	White	13.4	25.9	161	70	2.169851296	7.166690776	288.5378219	48.60199882
Normal	White	12.9	34.5	166	71	0.124746359	2.37215778	6.904368641	0.008922686
Normal	White	12.6	27.3	159	73	0.176715723	0.002940006	7.642872435	0.000308719
Normal	White	12.7	38	166	73	0.270972897	0.122640088	12.6595894	0.309648549
Normal	White	13.3	33.5	162.6	75	3.565910837	0.952643719	335.8641579	72.33527946
Normal	White	12.1	37.3	172	76	0.270972897	0.122640088	12.6595894	0.309648549
Normal	White	12.6	31.5	158	76	0.124746359	2.37215778	6.904368641	0.008922686
Normal	White	13.1	39.1	163	81	0.020311987	0.613446173	2.087025386	0.000275459
Normal	White	13.4	30.2	172	82	0.020311987	0.613446173	2.087025386	0.000275459
Normal	White	13	20.2	165	83	0.313012978	11.39411208	0.000243309	0.802508159
Normal	White	13.1	22.4	164	86	0.251954358	0.128625385	0.013844905	2.329187365
Normal	White	12.8	36.4	157.5	51	0.251954358	0.128625385	0.013844905	2.329187365
Normal	White	13.8	34.2	163	54	0.201765231	9.28E-05	3.367934242	1.300935568
Normal	White	12.1	30.3	157	54	0.251954358	0.128625385	0.013844905	2.329187365
Normal	White	13.7	25.8	160	55	1.147189178	6.149309885	0.00195811	0.016852717
Normal	White	13	35.1	162.6	56	1.147189178	6.149309885	0.00195811	0.016852717
Normal	White	13.8	34.9	154.9	56.5	0.618821907	1.282406956	142.5895547	0.252183263
Normal	White	13.2	35.5	163	57	0.648238947	0.170956069	40.9669193	157.8339256
Normal	White	12.4	28.6	155.5	58.5	3.295126487	47.53328687	0.380011491	0.334692287
Normal	White	13	33	162	59	0.313012978	11.39411208	0.000243309	0.802508159
Normal	White	11.3	31.7	170	60	0.648238947	0.170956069	40.9669193	157.8339256
Normal	White	12.4	26.3	147	60	1.759948264	110.663424	184.3518633	0.629789935

Normal	White	12.8	38	156	61	0.124746359	2.37215778	6.904368641	0.008922686
Normal	White	12.5	30.1	162	61	4.180572961	6.709695547	253.6370017	134.7686942
Normal	White	12.8	35.1	166	63	0.092415497	0.060841519	6.17E-05	0.00601206
Normal	White	13.6	30.4	165.1	63	3.176441134	10.90333478	0.002849085	268.9775465
Normal	White	12.9	30.3	164	64	0.618821907	1.282406956	142.5895547	0.252183263
Normal	White	13.1	30.5	161	65	1.147189178	6.149309885	0.00195811	0.016852717
Normal	White	13.1	35.3	172.7	65	0.875915925	0.42339554	164.5471986	0.697413605
Normal	White	12.1	25.6	168	65	0.648238947	0.170956069	40.9669193	157.8339256
Normal	White	13	31.8	170	67	0.875915925	0.42339554	164.5471986	0.697413605
Normal	White	13.2	29.8	171	68	0.648238947	0.170956069	40.9669193	157.8339256
Normal	White	12.3	32.4	157	68	1.147189178	6.149309885	0.00195811	0.016852717
Normal	White	13	35.4	154	69	4.180572961	6.709695547	253.6370017	134.7686942
Normal	White	11.3	36	168.5	72	0.176715723	0.002940006	7.642872435	0.000308719
Normal	White	12.6	34.9	164	73	4.00861349	0.002557265	0.036603836	0.008711746
Normal	White	12.6	36.1	172.7	76	1.147189178	6.149309885	0.00195811	0.016852717
Normal	White	12.2	33.8	180.3	78	0.72936366	3.388629009	0.000257766	10.84859942
Normal	White	12.3	33.2	163	98	0.618821907	1.282406956	142.5895547	0.252183263
Normal	White	12.8	31.5	152	48	0.618821907	1.282406956	142.5895547	0.252183263
Normal	White	12.2	33.7	154.5	49	0.72936366	3.388629009	0.000257766	10.84859942
Normal	White	11.6	36.8	149	53	0.251954358	0.128625385	0.013844905	2.329187365
Normal	White	12.3	31.6	153	55	0.313012978	11.39411208	0.000243309	0.802508159
Normal	White	12.8	29.4	164	58	0.72936366	3.388629009	0.000257766	10.84859942
Normal	White	12.7	33.4	173	59	3.176441134	10.90333478	0.002849085	268.9775465
Normal	White	12.9	29	158	59	0.201765231	9.28E-05	3.367934242	1.300935568
Normal	White	13.1	39	167.6	59.5	0.270972897	0.122640088	12.6595894	0.309648549
Normal	White	12.7	24	168	60	2.169851296	7.166690776	288.5378219	48.60199882
Normal	White	13.2	35.6	167	60	0.313012978	11.39411208	0.000243309	0.802508159
Normal	White	12.7	33.2	160	60	2.169851296	7.166690776	288.5378219	48.60199882
Normal	White	12.3	36	158	60	4.00861349	0.002557265	0.036603836	0.008711746
Normal	White	13.8	38.3	157	61	1.147189178	6.149309885	0.00195811	0.016852717
Normal	White	13.5	28.8	160.5	62	1.759948264	110.663424	184.3518633	0.629789935
Normal	White	12.7	31.8	158	65	3.176441134	10.90333478	0.002849085	268.9775465
Normal	White	12.4	21.7	158	67	0.201765231	9.28E-05	3.367934242	1.300935568
Normal	White	12.3	41	162	69	2.169851296	7.166690776	288.5378219	48.60199882
Normal	White	13	38.1	161	69	4.180572961	6.709695547	253.6370017	134.7686942
Normal	White	12.3	35.6	175	70	0.176715723	0.002940006	7.642872435	0.000308719
Normal	White	12.5	41	170.2	70	0.124746359	2.37215778	6.904368641	0.008922686
Normal	White	12.8	32.3	177.8	71	1.147189178	6.149309885	0.00195811	0.016852717
Normal	White	12	40.9	162.6	73	1.147189178	6.149309885	0.00195811	0.016852717
Normal	White	12.6	41.4	163	74	3.295126487	47.53328687	0.380011491	0.334692287
Normal	White	12.7	35.3	167.6	79.5	3.176441134	10.90333478	0.002849085	268.9775465

Normal	White	13.6	35.4	168	95	0.176715723	0.002940006	7.642872435	0.000308719
Normal	White	12.1	35.3	160	52	0.251954358	0.128625385	0.013844905	2.329187365
Normal	White	12.9	33.3	160	55	2.169851296	7.166690776	288.5378219	48.60199882
Normal	White	13.1	38.7	156	56	1.759948264	110.663424	184.3518633	0.629789935
Normal	White	12.7	36.3	164	56	0.270972897	0.122640088	12.6595894	0.309648549
Normal	White	11.8	29.7	167.6	58	0.648238947	0.170956069	40.9669193	157.8339256
Normal	White	12.9	28.3	166	59	0.270972897	0.122640088	12.6595894	0.309648549
Normal	White	12.6	33	155	61	0.124746359	2.37215778	6.904368641	0.008922686
Normal	White	12.9	22.7	157	61	3.176441134	10.90333478	0.002849085	268.9775465
Normal	White	13	21.3	158	62	1.147189178	6.149309885	0.00195811	0.016852717
Normal	White	12.5	23.6	158	62	0.875915925	0.42339554	164.5471986	0.697413605
Normal	White	12.8	27.8	153	62	4.180572961	6.709695547	253.6370017	134.7686942
Normal	White	12.8	34.5	165.1	63	0.270972897	0.122640088	12.6595894	0.309648549
Normal	White	12.9	30.6	170.2	64	3.565910837	0.952643719	335.8641579	72.33527946
Normal	White	12.9	33.3	165	65	4.180572961	6.709695547	253.6370017	134.7686942
Normal	White	13.2	33.4	168	66	0.618821907	1.282406956	142.5895547	0.252183263
Normal	White	13.2	35.5	165	67	3.176441134	10.90333478	0.002849085	268.9775465
Normal	White	12.7	29.1	165.1	67	0.875915925	0.42339554	164.5471986	0.697413605
Normal	White	12.2	37.7	159	67	0.618821907	1.282406956	142.5895547	0.252183263
Normal	White	11.8	32.1	175.3	67	0.648238947	0.170956069	40.9669193	157.8339256
Normal	White	12.7	35.4	157	70	0.618821907	1.282406956	142.5895547	0.252183263
Normal	White	11.6	33.7	159.5	70	2.169851296	7.166690776	288.5378219	48.60199882
Normal	White	13.3	34.7	176	72	3.295126487	47.53328687	0.380011491	0.334692287
Normal	White	12.5	26.7	171	72	3.565910837	0.952643719	335.8641579	72.33527946
Normal	White	12.5	30.1	160	72	3.176441134	10.90333478	0.002849085	268.9775465
Normal	White	12.4	35.3	172.7	78	0.124746359	2.37215778	6.904368641	0.008922686
Normal	White	13.4	35.9	179	82	0.72936366	3.388629009	0.000257766	10.84859942
Normal	White	13.8	35.5	174	83	0.72936366	3.388629009	0.000257766	10.84859942
Normal	White	14	22.3	180.3	84	3.176441134	10.90333478	0.002849085	268.9775465
Normal	White	12.9	34.8	167.6	94	0.648238947	0.170956069	40.9669193	157.8339256
Normal	White	12.8	19.5	159	51	3.565910837	0.952643719	335.8641579	72.33527946
Normal	White	12.2	20.9	157	53	8686.730764	18787.72221	2180.022227	947.1359042
Normal	White	12.8	43.6	158	56	3.176441134	10.90333478	0.002849085	268.9775465
Normal	White	12.4	30	167.6	58.5	4.00861349	0.002557265	0.036603836	0.008711746
Normal	White	13.4	36.7	162.6	60	0.313012978	11.39411208	0.000243309	0.802508159
Normal	White	12.7	21.5	177	61	3.295126487	47.53328687	0.380011491	0.334692287
Normal	White	13.5	19.5	164	62	0.092415497	0.060841519	6.17E-05	0.00601206
Normal	White	12.3	33.2	165	63	0.875915925	0.42339554	164.5471986	0.697413605
Normal	White	12.7	38.3	162.6	63	3.176441134	10.90333478	0.002849085	268.9775465
Normal	White	12.4	37.6	164	65	8686.730764	18787.72221	2180.022227	947.1359042
Normal	White	13.1	40.2	175	65	8686.730764	18787.72221	2180.022227	947.1359042

Normal	White	12.9	35.5	167	65	3.565910837	0.952643719	335.8641579	72.33527946
Normal	White	12.6	22	165.1	66	0.618821907	1.282406956	142.5895547	0.252183263
Normal	White	12.6	30.1	172	68	3.565910837	0.952643719	335.8641579	72.33527946
Normal	White	12.8	41.3	159	70	3.565910837	0.952643719	335.8641579	72.33527946
Normal	White	11.9	33.4	173	70	0.270972897	0.122640088	12.6595894	0.309648549
Normal	White	13	37.3	163	71	1.147189178	6.149309885	0.00195811	0.016852717
Normal	White	12.9	34.4	163	72	4.00861349	0.002557265	0.036603836	0.008711746
Normal	White	11.8	37.6	165.1	72	4.00861349	0.002557265	0.036603836	0.008711746
Normal	White	12.9	28.1	160	78	0.618821907	1.282406956	142.5895547	0.252183263
Normal	White	13	30.9	170.2	82.6	0.270972897	0.122640088	12.6595894	0.309648549
Normal	White	13.2	20	163	83	0.313012978	11.39411208	0.000243309	0.802508159
Normal	White	12.9	24.4	167	100	0.201765231	9.28E-05	3.367934242	1.300935568
Normal	White	12.4	29.9	163	52	8686.730764	18787.72221	2180.022227	947.1359042
Normal	White	12.3	25.2	157	52	3.176441134	10.90333478	0.002849085	268.9775465
Normal	White	12.5	29.1	164	53	0.313012978	11.39411208	0.000243309	0.802508159
Normal	White	12.1	32.9	164	55	0.72936366	3.388629009	0.000257766	10.84859942
Normal	White	12.4	28.2	163	56	0.875915925	0.42339554	164.5471986	0.697413605
Normal	White	12.5	32.6	164	58	1.759948264	110.663424	184.3518633	0.629789935
Normal	White	13.4	31.3	160	60	2.169851296	7.166690776	288.5378219	48.60199882
Normal	White	12.6	20.9	165	60	3.565910837	0.952643719	335.8641579	72.33527946
Normal	White	12.9	43.6	175	60	0.201765231	9.28E-05	3.367934242	1.300935568
Normal	White	13.1	27.2	167.6	61	0.72936366	3.388629009	0.000257766	10.84859942
Normal	White	12.4	23.3	155	61	2.169851296	7.166690776	288.5378219	48.60199882
Normal	White	12.5	38.7	173	61	0.618821907	1.282406956	142.5895547	0.252183263
Normal	White	12	30.8	165	62	0.648238947	0.170956069	40.9669193	157.8339256
Normal	White	12.2	21.3	165	62	0.124746359	2.37215778	6.904368641	0.008922686
Normal	White	12.7	37.1	166	62	0.201765231	9.28E-05	3.367934242	1.300935568
Normal	White	13.2	37.6	174	63.6	0.313012978	11.39411208	0.000243309	0.802508159
Normal	White	12.8	26.7	177	64	3.176441134	10.90333478	0.002849085	268.9775465
Normal	White	12.6	29.4	160	64	1.759948264	110.663424	184.3518633	0.629789935
Normal	White	12.8	21.7	170.2	64	0.72936366	3.388629009	0.000257766	10.84859942
Normal	White	12.4	31.2	176	65	3.176441134	10.90333478	0.002849085	268.9775465
Normal	White	13.5	34.8	163	65	4.00861349	0.002557265	0.036603836	0.008711746
Normal	White	12.7	22.2	166	67	0.72936366	3.388629009	0.000257766	10.84859942
Normal	White	12.6	33.5	164	69	0.875915925	0.42339554	164.5471986	0.697413605
Normal	White	13.1	34.5	160	69	0.313012978	11.39411208	0.000243309	0.802508159
Normal	White	13.1	36.7	155	70	3.176441134	10.90333478	0.002849085	268.9775465
Normal	White	13.6	36	158	70	1.759948264	110.663424	184.3518633	0.629789935
Normal	White	14.1	44.9	172.7	73.5	0.251954358	0.128625385	0.013844905	2.329187365
Normal	White	13.3	38.8	167	74	4.00861349	0.002557265	0.036603836	0.008711746
Normal	White	12.2	34.1	172.7	76	4.00861349	0.002557265	0.036603836	0.008711746

Normal	White	12.4	37.5	171	80	4.00861349	0.002557265	0.036603836	0.008711746
Normal	White	13	27.2	167.6	55	0.270972897	0.122640088	12.6595894	0.309648549
Normal	White	11.9	28.6	169	56	0.201765231	9.28E-05	3.367934242	1.300935568
Normal	White	12.9	20.7	168	56	0.020311987	0.613446173	2.087025386	0.000275459
Normal	White	13	34.7	167.6	57	8686.730764	18787.72221	2180.022227	947.1359042
Normal	White	13.7	29	160	57	3.176441134	10.90333478	0.002849085	268.9775465
Normal	White	11.9	37.8	159	57	0.020311987	0.613446173	2.087025386	0.000275459
Normal	White	12.8	32.2	152.4	59	0.875915925	0.42339554	164.5471986	0.697413605
Normal	White	12.5	37.4	167.6	59	0.648238947	0.170956069	40.9669193	157.8339256
Normal	White	13.9	36	164	60	0.72936366	3.388629009	0.000257766	10.84859942
Normal	White	12.5	31.6	157	60	0.020311987	0.613446173	2.087025386	0.000275459
Normal	White	11.8	35.5	165.1	60	0.124746359	2.37215778	6.904368641	0.008922686
Normal	White	13.2	39.3	157.5	61	4.00861349	0.002557265	0.036603836	0.008711746
Normal	White	13.9	41.6	157.5	61	3.565910837	0.952643719	335.8641579	72.33527946
Normal	White	11.9	23.8	161	61	0.251954358	0.128625385	0.013844905	2.329187365
Normal	White	12	33.3	170.2	61	3.565910837	0.952643719	335.8641579	72.33527946
Normal	White	12	31.2	167.6	62	1.147189178	6.149309885	0.00195811	0.016852717
Normal	White	12.6	27.7	160	62	0.092415497	0.060841519	6.17E-05	0.00601206
Normal	White	12.7	43.7	170.2	63	0.270972897	0.122640088	12.6595894	0.309648549
Normal	White	13.1	33.1	167.6	63	1.147189178	6.149309885	0.00195811	0.016852717
Normal	White	12.6	34.7	162.6	63	0.618821907	1.282406956	142.5895547	0.252183263
Normal	White	11.7	31.9	170.2	65	4.00861349	0.002557265	0.036603836	0.008711746
Normal	White	12.7	35.9	161	66	0.020311987	0.613446173	2.087025386	0.000275459
Normal	White	12.9	33.9	162.6	67	0.618821907	1.282406956	142.5895547	0.252183263
Normal	White	12.8	38.6	167.6	69.9	3.176441134	10.90333478	0.002849085	268.9775465
Normal	White	12.8	33	167.6	72	0.270972897	0.122640088	12.6595894	0.309648549
Normal	White	13.1	35.5	172.7	73	0.313012978	11.39411208	0.000243309	0.802508159
Normal	White	13.3	41.4	170.2	82	0.270972897	0.122640088	12.6595894	0.309648549
Normal	White	12.1	27.6	172.7	95	0.618821907	1.282406956	142.5895547	0.252183263
Normal	White	12.6	24.6	177.8	100	1.759948264	110.663424	184.3518633	0.629789935
Normal	White	13.3	37.7	154.9	44	3.565910837	0.952643719	335.8641579	72.33527946
Normal	White	12.6	25.2	147.3	45	0.251954358	0.128625385	0.013844905	2.329187365
Normal	White	12.7	37.4	165	57	0.72936366	3.388629009	0.000257766	10.84859942
Normal	White	13	35	166	58	1.759948264	110.663424	184.3518633	0.629789935
Normal	White	13.5	33.2	157.5	58	0.270972897	0.122640088	12.6595894	0.309648549
Normal	White	12.5	35.2	170.2	59.5	0.201765231	9.28E-05	3.367934242	1.300935568
Normal	White	12.6	31.8	162	60	3.565910837	0.952643719	335.8641579	72.33527946
Normal	White	12.3	35	160	60.5	0.313012978	11.39411208	0.000243309	0.802508159
Normal	White	13	32.2	159	63	2.169851296	7.166690776	288.5378219	48.60199882
Normal	White	13.5	41.4	170.2	64	0.124746359	2.37215778	6.904368641	0.008922686
Normal	White	13.6	35.6	166	66	0.618821907	1.282406956	142.5895547	0.252183263

Normal	White	13.5	34.4	160	66	1.759948264	110.663424	184.3518633	0.629789935
Normal	White	12.5	36.5	175	67	4.180572961	6.709695547	253.6370017	134.7686942
Normal	White	13	43.2	172.7	70	0.313012978	11.39411208	0.000243309	0.802508159
Normal	White	12.4	35.3	172	72	0.875915925	0.42339554	164.5471986	0.697413605
Normal	White	11.8	36.8	173	74	0.092415497	0.060841519	6.17E-05	0.00601206
Normal	White	12.5	36.7	170.2	76	4.00861349	0.002557265	0.036603836	0.008711746
Normal	White	12.2	31.6	174	76	0.648238947	0.170956069	40.9669193	157.8339256
Normal	White	13.2	33.1	165	77	0.092415497	0.060841519	6.17E-05	0.00601206
Normal	White	13.2	34.2	170.2	78	4.00861349	0.002557265	0.036603836	0.008711746
Normal	White	12.6	21.9	161	83	4.180572961	6.709695547	253.6370017	134.7686942
Normal	White	11.7	24.8	159	86	1.759948264	110.663424	184.3518633	0.629789935
Normal	White	12.9	40	168	85	3.176441134	10.90333478	0.002849085	268.9775465
Normal	White	12.9	20.5	167	80	0.875915925	0.42339554	164.5471986	0.697413605
Normal	White	11.6	32.3	175.3	76	4.00861349	0.002557265	0.036603836	0.008711746
Normal	White	13.1	33.8	170.2	69	0.875915925	0.42339554	164.5471986	0.697413605
Normal	White	13.1	33.4	172.7	60	0.270972897	0.122640088	12.6595894	0.309648549
Normal	White	13.1	38.2	169	77	0.313012978	11.39411208	0.000243309	0.802508159
Normal	White	12.3	32.9	162.6	55	0.124746359	2.37215778	6.904368641	0.008922686
Normal	White	12.5	31.6	156	72	0.72936366	3.388629009	0.000257766	10.84859942
Normal	White	12.8	38.7	182.9	80	3.565910837	0.952643719	335.8641579	72.33527946
Normal	White	12.8	29.5	162.6	58	4.00861349	0.002557265	0.036603836	0.008711746
Normal	White	12.8	24.4	158	56	0.313012978	11.39411208	0.000243309	0.802508159
Normal	White	13	44.9	153	62	8686.730764	18787.72221	2180.022227	947.1359042
Normal	White	13	33.9	160	55	8686.730764	18787.72221	2180.022227	947.1359042
Normal	White	11.7	26.4	166	64	0.875915925	0.42339554	164.5471986	0.697413605
Normal	White	11.7	20.5	173	53	1.147189178	6.149309885	0.00195811	0.016852717
Normal	White	12.5	35.3	179	88	3.565910837	0.952643719	335.8641579	72.33527946
Normal	White	12.6	35.1	165.1	77	0.201765231	9.28E-05	3.367934242	1.300935568
Normal	White	13.1	30.1	157.5	55	0.313012978	11.39411208	0.000243309	0.802508159
Normal	White	14	31.8	161	49	4.180572961	6.709695547	253.6370017	134.7686942
Normal	White	13.2	32	173	61	0.201765231	9.28E-05	3.367934242	1.300935568
Normal	White	12.2	34.2	185.4	78	0.020311987	0.613446173	2.087025386	0.000275459
Normal	White	12.6	28.2	180.3	67	0.313012978	11.39411208	0.000243309	0.802508159
Normal	White	12.9	30.1	166	52	2.169851296	7.166690776	288.5378219	48.60199882
Normal	White	12	26.5	162.6	62	0.092415497	0.060841519	6.17E-05	0.00601206
Normal	White	13	34	175.3	70	0.875915925	0.42339554	164.5471986	0.697413605
Normal	White	13.7	41.4	174	80	1.759948264	110.663424	184.3518633	0.629789935
Normal	White	12.3	34.1	160	64	0.201765231	9.28E-05	3.367934242	1.300935568
Normal	White	12	33.5	167	65	0.201765231	9.28E-05	3.367934242	1.300935568
Normal	White	12.2	33.8	147	45	4.00861349	0.002557265	0.036603836	0.008711746
Normal	White	13.1	26.5	158	55	0.251954358	0.128625385	0.013844905	2.329187365

Normal	White	12.8	40.7	168	73	0.875915925	0.42339554	164.5471986	0.697413605
Normal	White	12.3	30.2	168	57	4.180572961	6.709695547	253.6370017	134.7686942
Normal	White	12.5	35.5	163	65	0.72936366	3.388629009	0.000257766	10.84859942
Normal	White	11.9	36.2	170.2	64	0.313012978	11.39411208	0.000243309	0.802508159
Normal	White	12.8	40.4	172.7	77	3.565910837	0.952643719	335.8641579	72.33527946
Normal	White	12.4	32.9	172.7	64	2.169851296	7.166690776	288.5378219	48.60199882
Normal	White	12.8	24.7	172.7	64	0.313012978	11.39411208	0.000243309	0.802508159
Normal	White	12.3	41	167.6	62	4.180572961	6.709695547	253.6370017	134.7686942
Normal	White	13.2	36.9	178	63	0.201765231	9.28E-05	3.367934242	1.300935568
Normal	White	12.1	42.1	168	59	1.759948264	110.663424	184.3518633	0.629789935
Normal	White	12.9	36.2	159	65	0.020311987	0.613446173	2.087025386	0.000275459
Normal	White	12.6	31.9	175	64	0.270972897	0.122640088	12.6595894	0.309648549
Normal	White	12.8	36.2	167.6	55	3.176441134	10.90333478	0.002849085	268.9775465
Normal	White	12.2	36.2	170.2	74	0.270972897	0.122640088	12.6595894	0.309648549
Normal	White	12.4	30.8	157.5	57	0.270972897	0.122640088	12.6595894	0.309648549
Normal	White	12.7	34	163	80	8686.730764	18787.72221	2180.022227	947.1359042
Normal	White	12.4	29.4	167	61	4.180572961	6.709695547	253.6370017	134.7686942
Normal	White	12.7	28.8	162.6	53	8686.730764	18787.72221	2180.022227	947.1359042
Normal	White	12.4	21.5	165.1	47.7	0.176715723	0.002940006	7.642872435	0.000308719
Normal	White	12.4	28.8	144	41	0.251954358	0.128625385	0.013844905	2.329187365
Normal	White	13.8	28.2	157.5	60	3.295126487	47.53328687	0.380011491	0.334692287
Normal	White	12	33.5	177.8	66	0.092415497	0.060841519	6.17E-05	0.00601206
Normal	White	11.8	32.7	168	55	3.295126487	47.53328687	0.380011491	0.334692287
Normal	White	12.5	29.1	163	49	0.251954358	0.128625385	0.013844905	2.329187365
Normal	White	13.7	27.5	163	80	0.648238947	0.170956069	40.9669193	157.8339256
Normal	White	12.8	34.9	172.7	74	0.201765231	9.28E-05	3.367934242	1.300935568
Normal	White	12.7	36.5	171	76	3.565910837	0.952643719	335.8641579	72.33527946
Normal	White	12.6	35.6	162.6	61	3.176441134	10.90333478	0.002849085	268.9775465
Normal	White	12.6	33.7	167.6	69	0.092415497	0.060841519	6.17E-05	0.00601206
Normal	White	12.6	37.3	170.2	56.3	0.201765231	9.28E-05	3.367934242	1.300935568
Normal	White	13.3	21.4	161	64	3.565910837	0.952643719	335.8641579	72.33527946
Normal	White	12.5	34.3	165	65	1.759948264	110.663424	184.3518633	0.629789935
Normal	White	12.6	37.9	165.1	92	0.618821907	1.282406956	142.5895547	0.252183263
Normal	White	12	33.8	172.7	62	3.565910837	0.952643719	335.8641579	72.33527946
Normal	White	13	31.8	160	63.6	4.180572961	6.709695547	253.6370017	134.7686942
Normal	White	12.2	27.2	173	90	0.270972897	0.122640088	12.6595894	0.309648549
Normal	White	12.6	24.4	160	64	0.020311987	0.613446173	2.087025386	0.000275459
Normal	White	12.2	32.8	168	51.3	0.72936366	3.388629009	0.000257766	10.84859942
Normal	White	12.8	32.8	175.3	61	8686.730764	18787.72221	2180.022227	947.1359042
Normal	White	12.7	33.7	170	68	0.251954358	0.128625385	0.013844905	2.329187365
Normal	White	12.7	31.3	160	61	1.759948264	110.663424	184.3518633	0.629789935

Normal	White	13.2	40.9	162.6	60	0.648238947	0.170956069	40.9669193	157.8339256
Normal	White	12.8	30.5	174	82	4.180572961	6.709695547	253.6370017	134.7686942
Normal	White	13.3	35	170.2	98	3.565910837	0.952643719	335.8641579	72.33527946
Normal	White	11.6	26	170.2	85	0.201765231	9.28E-05	3.367934242	1.300935568
Normal	White	12.4	39.5	175.3	82	0.72936366	3.388629009	0.000257766	10.84859942
Normal	White	12.5	37.6	162.6	86	0.251954358	0.128625385	0.013844905	2.329187365
Normal	White	12.7	31.8	165.1	60	0.875915925	0.42339554	164.5471986	0.697413605
Normal	White	12.7	31.5	168	88	0.648238947	0.170956069	40.9669193	157.8339256
Normal	White	13.9	38.5	157.5	84	0.201765231	9.28E-05	3.367934242	1.300935568
Normal	White	12.3	35.8	161	56	3.295126487	47.53328687	0.380011491	0.334692287
Normal	White	13.2	28.7	162.6	60	0.313012978	11.39411208	0.000243309	0.802508159
Normal	White	13.8	29.7	168	55	0.251954358	0.128625385	0.013844905	2.329187365
Normal	White	12.8	32.5	172.7	69	0.020311987	0.613446173	2.087025386	0.000275459
Normal	White	13.3	35.4	159	80	0.618821907	1.282406956	142.5895547	0.252183263
Normal	White	12	33.7	172.7	74	0.648238947	0.170956069	40.9669193	157.8339256
Normal	White	12.6	29.7	161	51	0.092415497	0.060841519	6.17E-05	0.00601206
Normal	White	12.3	33.1	167.6	60	4.00861349	0.002557265	0.036603836	0.008711746
Normal	White	13.1	30.7	158	56	0.313012978	11.39411208	0.000243309	0.802508159
Normal	White	13.1	33.2	165	68	0.313012978	11.39411208	0.000243309	0.802508159
Normal	White	13.8	21.4	160	56	0.201765231	9.28E-05	3.367934242	1.300935568
Normal	White	13.7	25.1	157.5	58	0.201765231	9.28E-05	3.367934242	1.300935568
Normal	White	12.2	36.7	165.1	50	1.147189178	6.149309885	0.00195811	0.016852717
Normal	White	12.2	37.6	172.7	60	0.092415497	0.060841519	6.17E-05	0.00601206
Normal	White	12.5	30.6	165.1	83	4.180572961	6.709695547	253.6370017	134.7686942
Normal	White	13.3	40.7	167.6	73	0.618821907	1.282406956	142.5895547	0.252183263
Normal	White	12.8	34.2	157.5	55	3.565910837	0.952643719	335.8641579	72.33527946
Normal	White	12.5	37.3	172.7	68.5	0.092415497	0.060841519	6.17E-05	0.00601206
Normal	White	13.6	33.3	165	73	0.020311987	0.613446173	2.087025386	0.000275459
Normal	White	13	33.6	150	75	2.169851296	7.166690776	288.5378219	48.60199882
Normal	White	13.2	37.3	177.8	82.6	0.618821907	1.282406956	142.5895547	0.252183263
Normal	White	12	31.8	162.6	65	4.00861349	0.002557265	0.036603836	0.008711746
Normal	White	12.5	30.7	176	62	1.759948264	110.663424	184.3518633	0.629789935
Normal	White	11.5	37.4	162	67	3.565910837	0.952643719	335.8641579	72.33527946
Normal	White	12.4	37.3	160	54	0.648238947	0.170956069	40.9669193	157.8339256
Normal	White	12.6	31.9	163	64	3.565910837	0.952643719	335.8641579	72.33527946
Normal	White	12.7	24.2	168	68	3.295126487	47.53328687	0.380011491	0.334692287
Normal	White	12.3	34.9	173	75	0.648238947	0.170956069	40.9669193	157.8339256
Normal	White	13.5	37.2	165.1	60	0.648238947	0.170956069	40.9669193	157.8339256
Normal	White	12.7	32.6	157.5	60	1.759948264	110.663424	184.3518633	0.629789935
Normal	White	12.1	40.6	172.7	52	0.875915925	0.42339554	164.5471986	0.697413605
Normal	White	12.4	36	160	56	3.176441134	10.90333478	0.002849085	268.9775465

Normal	White	12.4	31.9	160	59	0.648238947	0.170956069	40.9669193	157.8339256
Normal	White	13.3	29.3	167.6	68	0.020311987	0.613446173	2.087025386	0.000275459
Normal	White	12.4	21.8	153	47	0.648238947	0.170956069	40.9669193	157.8339256
Normal	White	12.9	20.7	170.2	50	4.180572961	6.709695547	253.6370017	134.7686942
Normal	White	12.6	42.8	165.1	69	0.648238947	0.170956069	40.9669193	157.8339256
Normal	White	12.9	43.4	170.2	56	0.124746359	2.37215778	6.904368641	0.008922686
Normal	White	13.1	29.1	174	65	4.180572961	6.709695547	253.6370017	134.7686942
Normal	White	13.9	28.1	165.1	65	2.169851296	7.166690776	288.5378219	48.60199882
Normal	White	12.4	36.2	162.6	59	0.251954358	0.128625385	0.013844905	2.329187365
Normal	White	13.3	23.5	160	60	0.176715723	0.002940006	7.642872435	0.000308719
Normal	White	13.1	35.1	169	61.8	0.092415497	0.060841519	6.17E-05	0.00601206
Normal	White	13.3	26.3	163	65	0.270972897	0.122640088	12.6595894	0.309648549
Normal	White	12.2	36.4	171	67.2	0.124746359	2.37215778	6.904368641	0.008922686
Normal	White	13	37.6	168	62.9	8686.730764	18787.72221	2180.022227	947.1359042
Normal	White	12.4	30	165.1	55	3.295126487	47.53328687	0.380011491	0.334692287
Normal	White	12.9	32.4	177.8	70.8	0.124746359	2.37215778	6.904368641	0.008922686
Normal	White	13.2	35.8	161	68	0.092415497	0.060841519	6.17E-05	0.00601206
Normal	White	13.7	37.9	156	72	8686.730764	18787.72221	2180.022227	947.1359042
Normal	White	11.8	36.9	167.6	62.6	3.295126487	47.53328687	0.380011491	0.334692287
Normal	White	12.8	19.3	161	56.6	0.313012978	11.39411208	0.000243309	0.802508159
Normal	White	11.8	32.7	158	56.6	0.124746359	2.37215778	6.904368641	0.008922686
Normal	White	12	38.3	167.6	64.6	0.270972897	0.122640088	12.6595894	0.309648549
Normal	White	12.5	33.8	170.2	79.1	0.176715723	0.002940006	7.642872435	0.000308719
Normal	White	12.2	29.6	165	55	2.169851296	7.166690776	288.5378219	48.60199882
Normal	White	13.5	36	165.1	75.4	0.313012978	11.39411208	0.000243309	0.802508159
Normal	White	12.8	31.3	167.6	63.1	0.270972897	0.122640088	12.6595894	0.309648549
Normal	White	12.5	26.4	166	55.7	3.176441134	10.90333478	0.002849085	268.9775465
Normal	White	12.4	34.4	170	67.5	0.270972897	0.122640088	12.6595894	0.309648549
Normal	White	11.8	27.4	175.3	96.7	4.180572961	6.709695547	253.6370017	134.7686942
Normal	White	12.5	34.7	154.9	49	0.72936366	3.388629009	0.000257766	10.84859942
Normal	White	12.2	35.8	157.5	56.9	4.180572961	6.709695547	253.6370017	134.7686942
Normal	White	13.7	34.4	157.5	53	1.759948264	110.663424	184.3518633	0.629789935
Normal	White	12	33.2	162.6	54.3	3.565910837	0.952643719	335.8641579	72.33527946
Normal	White	12.8	33.1	167.6	56	8686.730764	18787.72221	2180.022227	947.1359042
Normal	White	11.3	26	160	72.7	3.295126487	47.53328687	0.380011491	0.334692287
Normal	White	11.6	33.9	165.1	65	2.169851296	7.166690776	288.5378219	48.60199882
Normal	White	12.1	30.5	167.6	62.8	0.270972897	0.122640088	12.6595894	0.309648549
Normal	White	12.2	31.4	160	65	0.176715723	0.002940006	7.642872435	0.000308719
Normal	White	13.4	30	195.6	66	0.020311987	0.613446173	2.087025386	0.000275459
Normal	White	12.8	29.6	160	51	2.169851296	7.166690776	288.5378219	48.60199882
Normal	White	12.1	18.1	172	61	0.201765231	9.28E-05	3.367934242	1.300935568

Normal	White	12.3	22.1	172.7	54	0.648238947	0.170956069	40.9669193	157.8339256
Normal	White	12.2	35.1	168	70.3	0.72936366	3.388629009	0.000257766	10.84859942
Normal	White	13.7	22.5	177	61.9	3.295126487	47.53328687	0.380011491	0.334692287
Normal	White	12.9	33.4	169	70	4.00861349	0.002557265	0.036603836	0.008711746
Normal	White	13.3	39.5	162.6	61	1.147189178	6.149309885	0.00195811	0.016852717
Normal	White	12.9	35.2	167.6	57.2	3.176441134	10.90333478	0.002849085	268.9775465
Normal	White	12.7	23.3	160	55.9	0.201765231	9.28E-05	3.367934242	1.300935568
Normal	White	12.9	37.8	177.8	69.5	0.313012978	11.39411208	0.000243309	0.802508159
Normal	White	12.7	35.1	167.6	65.2	0.251954358	0.128625385	0.013844905	2.329187365
Normal	White	12.1	41.1	172.7	72.1	3.176441134	10.90333478	0.002849085	268.9775465
Normal	White	12.2	27.5	162.6	55.5	0.72936366	3.388629009	0.000257766	10.84859942
Normal	White	13.4	33.2	170.2	69	3.176441134	10.90333478	0.002849085	268.9775465
Normal	White	12.2	38	160	63.5	0.092415497	0.060841519	6.17E-05	0.00601206
Normal	White	11.9	29.8	160	61.5	1.147189178	6.149309885	0.00195811	0.016852717
Normal	White	12.2	39.2	160	75	0.251954358	0.128625385	0.013844905	2.329187365
Normal	White	12	32.2	160.7	76.9	0.176715723	0.002940006	7.642872435	0.000308719
Normal	White	13.2	38.5	165	64.4	0.124746359	2.37215778	6.904368641	0.008922686
Normal	White	13	38.9	168	65	0.124746359	2.37215778	6.904368641	0.008922686
Normal	White	12.3	39.5	163	71	4.180572961	6.709695547	253.6370017	134.7686942
Normal	White	11.4	30.7	157.5	63	0.618821907	1.282406956	142.5895547	0.252183263
Normal	White	12.9	32	170.2	70	0.648238947	0.170956069	40.9669193	157.8339256
Normal	White	12.2	27.9	165	65	0.72936366	3.388629009	0.000257766	10.84859942
Normal	White	12.9	29.9	169	73.2	4.180572961	6.709695547	253.6370017	134.7686942
Normal	White	12.7	34.2	167.6	64.5	8686.730764	18787.72221	2180.022227	947.1359042
Normal	White	12.7	39.6	157.5	57	0.020311987	0.613446173	2.087025386	0.000275459
Normal	White	13	41.4	162.6	59	0.176715723	0.002940006	7.642872435	0.000308719
Normal	White	11.4	37.5	185.4	76.9	0.092415497	0.060841519	6.17E-05	0.00601206
Normal	White	12	33.9	162	60.4	1.759948264	110.663424	184.3518633	0.629789935
Normal	White	12.5	27.5	167.6	58.5	0.72936366	3.388629009	0.000257766	10.84859942
Normal	White	12.4	24.8	169.5	83.6	8686.730764	18787.72221	2180.022227	947.1359042
Normal	White	13.2	35	165.1	64	0.251954358	0.128625385	0.013844905	2.329187365
Normal	White	12.5	32.8	169	60.3	1.147189178	6.149309885	0.00195811	0.016852717
Normal	White	12.8	33.1	170	84.5	0.618821907	1.282406956	142.5895547	0.252183263
Normal	White	12.6	28.8	153	54.7	3.295126487	47.53328687	0.380011491	0.334692287
Normal	White	12.8	30.9	172.7	92	0.020311987	0.613446173	2.087025386	0.000275459
Normal	White	12.5	29.5	160	49.6	2.169851296	7.166690776	288.5378219	48.60199882
Normal	White	12.2	36.6	165.1	59.2	0.124746359	2.37215778	6.904368641	0.008922686
Normal	White	12.3	36.7	169	57.4	0.020311987	0.613446173	2.087025386	0.000275459
Normal	White	12.9	38.8	149	74.1	8686.730764	18787.72221	2180.022227	947.1359042
Normal	White	12.6	31.4	161	79.5	0.251954358	0.128625385	0.013844905	2.329187365
Normal	White	12.1	37.3	165.1	85	0.124746359	2.37215778	6.904368641	0.008922686

Normal	White	12.6	37	170	100.8	1.147189178	6.149309885	0.00195811	0.016852717
Normal	White	12.4	35.6	172	73	0.72936366	3.388629009	0.000257766	10.84859942
Normal	White	12.2	29.2	171	69.7	0.72936366	3.388629009	0.000257766	10.84859942
Normal	White	12.3	30.9	170.2	63.7	4.00861349	0.002557265	0.036603836	0.008711746
Normal	White	12.6	23.8	167.6	62	8686.730764	18787.72221	2180.022227	947.1359042
Normal	White	12.6	33.7	167	65.2	1.759948264	110.663424	184.3518633	0.629789935
Normal	White	12.1	37.4	170.2	69.9	0.124746359	2.37215778	6.904368641	0.008922686
Normal	White	11.2	29	170.2	56	0.270972897	0.122640088	12.6595894	0.309648549
Normal	White	12.7	31	165.1	51.8	8686.730764	18787.72221	2180.022227	947.1359042
Normal	White	12.8	29.2	168	56	0.176715723	0.002940006	7.642872435	0.000308719
Normal	White	12.6	28.2	160	59.1	0.72936366	3.388629009	0.000257766	10.84859942
Normal	White	12.7	27.8	178	61.9	0.270972897	0.122640088	12.6595894	0.309648549
Normal	White	13.9	33.7	172	93.4	1.147189178	6.149309885	0.00195811	0.016852717
Normal	White	12.1	35	160	60.4	1.147189178	6.149309885	0.00195811	0.016852717
Normal	White	13.8	22.1	150	40	0.875915925	0.42339554	164.5471986	0.697413605
Normal	White	12.7	37.2	158	60	8686.730764	18787.72221	2180.022227	947.1359042
Normal	White	13.4	33.6	167.6	65	3.295126487	47.53328687	0.380011491	0.334692287
Normal	White	12.4	31.8	167	68	0.176715723	0.002940006	7.642872435	0.000308719
Normal	White	13.2	23.8	165	48.6	0.313012978	11.39411208	0.000243309	0.802508159
Normal	White	12.6	36.1	169	60	0.72936366	3.388629009	0.000257766	10.84859942
Normal	White	13.2	34.3	172	62	0.251954358	0.128625385	0.013844905	2.329187365
Normal	White	12.2	37.5	160	73.5	2.169851296	7.166690776	288.5378219	48.60199882
Normal	White	14	32.7	162	65.3	0.875915925	0.42339554	164.5471986	0.697413605
Normal	White	12.7	31.8	154	78.5	1.147189178	6.149309885	0.00195811	0.016852717
Normal	White	13.5	23.3	162	94.5	0.875915925	0.42339554	164.5471986	0.697413605
Normal	White	13.2	23.6	168	55.6	4.00861349	0.002557265	0.036603836	0.008711746
Normal	White	12.6	24.3	167	96.7	0.875915925	0.42339554	164.5471986	0.697413605
Normal	White	12.4	29.5	164	50.2	0.875915925	0.42339554	164.5471986	0.697413605
Normal	White	12.5	33.3	155	55	4.180572961	6.709695547	253.6370017	134.7686942
Normal	White	13.2	30.4	177.8	63.6	4.180572961	6.709695547	253.6370017	134.7686942
Normal	White	12.7	31.4	167	65.4	0.875915925	0.42339554	164.5471986	0.697413605
Normal	White	13.3	30.8	165.1	67	0.201765231	9.28E-05	3.367934242	1.300935568
Normal	White	13	34.3	162	70.7	3.176441134	10.90333478	0.002849085	268.9775465
Normal	White	12.7	26.6	174	79.9	8686.730764	18787.72221	2180.022227	947.1359042
Normal	White	12.7	36.6	170	91.7	1.759948264	110.663424	184.3518633	0.629789935
Normal	White	12.5	33.1	167	99.6	0.875915925	0.42339554	164.5471986	0.697413605
Normal	White	12.6	30.6	172.7	75.7	3.565910837	0.952643719	335.8641579	72.33527946
Normal	White	13.1	41.6	165.1	57.2	3.176441134	10.90333478	0.002849085	268.9775465
Normal	White	12.5	44.4	157	64.5	0.72936366	3.388629009	0.000257766	10.84859942
Normal	White	13	34	182	74.8	0.648238947	0.170956069	40.9669193	157.8339256
Normal	White	14.1	26.4	178	67.7	4.180572961	6.709695547	253.6370017	134.7686942

Normal	White	12.8	35.5	176	73	0.618821907	1.282406956	142.5895547	0.252183263
Normal	White	12.8	36	166.5	86.9	0.618821907	1.282406956	142.5895547	0.252183263
Normal	White	12.4	31.3	167	72	4.00861349	0.002557265	0.036603836	0.008711746
Normal	White	12.6	30.4	172.7	61	0.313012978	11.39411208	0.000243309	0.802508159
Normal	White	12.3	19.8	167.6	91.5	3.565910837	0.952643719	335.8641579	72.33527946
Normal	White	12.6	31.7	161	51	4.180572961	6.709695547	253.6370017	134.7686942
Normal	White	12.4	37.6	172.7	66.6	3.565910837	0.952643719	335.8641579	72.33527946
Normal	White	12.7	34.8	163	62	0.270972897	0.122640088	12.6595894	0.309648549
Normal	White	12.8	32.7	167	55.6	4.180572961	6.709695547	253.6370017	134.7686942
Normal	White	12.1	31	165	64.5	1.759948264	110.663424	184.3518633	0.629789935
Normal	White	13.3	36.5	170	69.2	3.295126487	47.53328687	0.380011491	0.334692287
Normal	White	12.4	32.2	171	59.6	1.759948264	110.663424	184.3518633	0.629789935
Normal	White	12.1	30.5	162.6	73	0.092415497	0.060841519	6.17E-05	0.00601206
Normal	White	12.3	30.3	161.5	57.5	2.169851296	7.166690776	288.5378219	48.60199882
Normal	White	12.8	41.5	165.1	61	0.251954358	0.128625385	0.013844905	2.329187365
Normal	White	11.5	36.5	172	62.7	3.176441134	10.90333478	0.002849085	268.9775465
Normal	White	12.4	29.6	170	73	4.00861349	0.002557265	0.036603836	0.008711746
Normal	White	12	29.2	163.5	87.6	0.648238947	0.170956069	40.9669193	157.8339256
Normal	White	13.5	32.7	169	119.7	0.270972897	0.122640088	12.6595894	0.309648549
Normal	White	11.5	32.4	168	62	0.270972897	0.122640088	12.6595894	0.309648549
Normal	White	11.4	34.6	163	58.2	0.875915925	0.42339554	164.5471986	0.697413605
Normal	White	11.3	31.2	162.6	51.5	3.176441134	10.90333478	0.002849085	268.9775465
Normal	White	13.5	32.8	162.6	65.3	2.169851296	7.166690776	288.5378219	48.60199882
Normal	White	12.9	34.4	163	59	0.201765231	9.28E-05	3.367934242	1.300935568
Normal	White	12.6	35.3	180.3	65	0.251954358	0.128625385	0.013844905	2.329187365
Normal	White	13.2	39.6	160	55	1.759948264	110.663424	184.3518633	0.629789935
Normal	White	13.1	28	158	53.7	0.176715723	0.002940006	7.642872435	0.000308719
Normal	White	13	36.9	170.2	62	0.618821907	1.282406956	142.5895547	0.252183263
Normal	White	11.9	31.9	172	68	0.313012978	11.39411208	0.000243309	0.802508159
Normal	White	12.5	30.3	162.6	61.9	0.124746359	2.37215778	6.904368641	0.008922686
Normal	White	12.3	29.1	163	105.8	0.092415497	0.060841519	6.17E-05	0.00601206
Normal	White	12.4	31.4	169	62.7	0.270972897	0.122640088	12.6595894	0.309648549
Normal	White	12.4	33.1	167.6	56.4	0.313012978	11.39411208	0.000243309	0.802508159
Normal	White	11.7	22.8	166	60	2.169851296	7.166690776	288.5378219	48.60199882
Normal	White	13.2	31.9	164	65	4.00861349	0.002557265	0.036603836	0.008711746
Normal	White	11.9	39.8	168	63.1	1.147189178	6.149309885	0.00195811	0.016852717
Normal	White	13.2	25.8	165.1	51.9	0.270972897	0.122640088	12.6595894	0.309648549
Normal	White	12	25.2	160	62.8	3.565910837	0.952643719	335.8641579	72.33527946
Normal	White	11.6	30.3	158	60	8686.730764	18787.72221	2180.022227	947.1359042
Normal	White	12.7	28.1	162	64	0.618821907	1.282406956	142.5895547	0.252183263
Normal	White	12	28.4	165.1	57.9	0.251954358	0.128625385	0.013844905	2.329187365

Normal	White	12.6	33.9	162	59	0.251954358	0.128625385	0.013844905	2.329187365
Normal	White	12.6	25.2	168	61	0.72936366	3.388629009	0.000257766	10.84859942
Normal	White	11.9	30.5	170	79	3.565910837	0.952643719	335.8641579	72.33527946
Normal	White	13	34.1	172.7	69.1	3.176441134	10.90333478	0.002849085	268.9775465
Normal	White	12.4	30.4	170	67	1.147189178	6.149309885	0.00195811	0.016852717
Normal	White	12.3	38.7	165.1	65.6	3.176441134	10.90333478	0.002849085	268.9775465
Normal	White	13	34.9	166	73.2	0.124746359	2.37215778	6.904368641	0.008922686
Normal	White	13.1	38.2	169	58	0.875915925	0.42339554	164.5471986	0.697413605
Normal	White	12.1	34.9	165.1	64	8686.730764	18787.72221	2180.022227	947.1359042
Normal	White	12.5	40.2	173	65.9	8686.730764	18787.72221	2180.022227	947.1359042
Normal	White	13.1	35.5	165.1	83.9	1.147189178	6.149309885	0.00195811	0.016852717
Normal	White	12.6	34.6	162.6	62.2	0.648238947	0.170956069	40.9669193	157.8339256
Normal	White	12.4	28.4	159	55	4.00861349	0.002557265	0.036603836	0.008711746
Normal	White	12.9	31.3	163	49.6	0.251954358	0.128625385	0.013844905	2.329187365
Normal	White	12.9	36.5	163	77	0.020311987	0.613446173	2.087025386	0.000275459
Normal	White	12.5	33.9	161	53.5	0.020311987	0.613446173	2.087025386	0.000275459
Normal	White	12.3	18.3	158	53.8	0.201765231	9.28E-05	3.367934242	1.300935568
Normal	White	12.9	34.9	170.2	69.7	0.270972897	0.122640088	12.6595894	0.309648549
Normal	White	12.1	35.7	177.8	65.5	0.201765231	9.28E-05	3.367934242	1.300935568
Normal	White	12.7	37.9	157.5	57	3.176441134	10.90333478	0.002849085	268.9775465
Normal	White	13.3	20.5	160	52	4.00861349	0.002557265	0.036603836	0.008711746
Normal	White	12.8	29.2	164	65	0.176715723	0.002940006	7.642872435	0.000308719
Normal	White	11.8	31.3	170.2	75.8	3.565910837	0.952643719	335.8641579	72.33527946
Normal	White	12.6	34	167	57.3	8686.730764	18787.72221	2180.022227	947.1359042
Normal	White	13.4	23.9	161	59.1	0.092415497	0.060841519	6.17E-05	0.00601206
Normal	White	12.5	34.9	178	65.3	0.270972897	0.122640088	12.6595894	0.309648549
Normal	White	12.4	39.2	170.2	66	0.72936366	3.388629009	0.000257766	10.84859942
Normal	White	13.1	23.9	162	69	0.875915925	0.42339554	164.5471986	0.697413605
Normal	White	12.1	27.9	175.3	108	0.648238947	0.170956069	40.9669193	157.8339256
Normal	White	12.8	33.2	170.2	58.1	0.313012978	11.39411208	0.000243309	0.802508159
Normal	White	13.2	27.1	157.5	58	0.201765231	9.28E-05	3.367934242	1.300935568
Normal	White	12.9	32.5	169	72.3	3.565910837	0.952643719	335.8641579	72.33527946
Normal	White	12.4	30	162	57	0.251954358	0.128625385	0.013844905	2.329187365
Normal	White	12.2	28.8	162.6	95	0.201765231	9.28E-05	3.367934242	1.300935568
Normal	White	11.4	28.2	166	61.2	3.295126487	47.53328687	0.380011491	0.334692287
Normal	White	12.6	30.1	150	82.2	0.618821907	1.282406956	142.5895547	0.252183263
Normal	White	11.3	31.7	162	52	1.759948264	110.663424	184.3518633	0.629789935
Normal	White	12.3	38.5	157.5	87	1.147189178	6.149309885	0.00195811	0.016852717
Normal	White	11.8	30.2	170.2	91.4	0.270972897	0.122640088	12.6595894	0.309648549
Normal	White	12.5	31.8	164	58.8	3.176441134	10.90333478	0.002849085	268.9775465
Normal	White	13.6	27.7	161	69.1	0.176715723	0.002940006	7.642872435	0.000308719

Normal	White	12.9	30.5	160	59.8	3.176441134	10.90333478	0.002849085	268.9775465
Normal	White	11.4	27.1	165.1	69	0.092415497	0.060841519	6.17E-05	0.00601206
Normal	White	11.8	28.7	152.4	48	2.169851296	7.166690776	288.5378219	48.60199882
Normal	White	13.2	38.5	165	75.4	3.565910837	0.952643719	335.8641579	72.33527946
Normal	White	12.2	32	166.3	59.2	0.648238947	0.170956069	40.9669193	157.8339256
Normal	White	11.9	24.6	151	54	0.72936366	3.388629009	0.000257766	10.84859942
Normal	White	12.5	27.3	163	58	2.169851296	7.166690776	288.5378219	48.60199882
Normal	White	12.5	32.9	168	62.5	0.124746359	2.37215778	6.904368641	0.008922686
Normal	White	12.3	33.9	165	63	0.618821907	1.282406956	142.5895547	0.252183263
Normal	White	12.9	31.4	169	65	0.648238947	0.170956069	40.9669193	157.8339256
Normal	White	12.9	40.2	171	71.3	4.180572961	6.709695547	253.6370017	134.7686942
Normal	White	12.6	29.2	182	76	8686.730764	18787.72221	2180.022227	947.1359042
Normal	White	11.9	19.7	161	78.3	0.270972897	0.122640088	12.6595894	0.309648549
Normal	White	12.3	22.7	160	80	0.618821907	1.282406956	142.5895547	0.252183263
Normal	White	12.1	28.2	170.2	82	0.020311987	0.613446173	2.087025386	0.000275459
Normal	White	13.3	29.7	161	95	1.759948264	110.663424	184.3518633	0.629789935
Normal	White	12.2	19.8	170	59.6	0.72936366	3.388629009	0.000257766	10.84859942
Normal	White	11.8	37.3	160	48.6	1.147189178	6.149309885	0.00195811	0.016852717
Normal	White	12.9	29	163	57.8	0.875915925	0.42339554	164.5471986	0.697413605
Normal	White	12.2	30.8	166	61	0.648238947	0.170956069	40.9669193	157.8339256
Normal	White	12.1	38.5	172	73	4.00861349	0.002557265	0.036603836	0.008711746
Normal	White	13	28.2	170.5	54	3.295126487	47.53328687	0.380011491	0.334692287
Normal	White	13.2	24.6	172.7	86	3.176441134	10.90333478	0.002849085	268.9775465
Normal	White	12.8	36.9	159	71	0.124746359	2.37215778	6.904368641	0.008922686
Normal	White	12.8	32.2	164	55	1.759948264	110.663424	184.3518633	0.629789935
Normal	White	13.4	23.2	172.5	74.1	1.147189178	6.149309885	0.00195811	0.016852717
Normal	White	12.7	39.2	171	66.8	1.147189178	6.149309885	0.00195811	0.016852717
Normal	White	12.2	33.7	156	62	0.201765231	9.28E-05	3.367934242	1.300935568
Normal	White	12.6	35.1	154	66	0.251954358	0.128625385	0.013844905	2.329187365
Normal	White	12.2	35.4	164	54	0.270972897	0.122640088	12.6595894	0.309648549
Normal	White	12.7	40.8	162	62	0.176715723	0.002940006	7.642872435	0.000308719
Normal	White	13.5	37.8	168	67	0.092415497	0.060841519	6.17E-05	0.00601206
Normal	White	12.5	33.8	167	72.2	0.201765231	9.28E-05	3.367934242	1.300935568
Normal	White	12	29	166	67.7	3.565910837	0.952643719	335.8641579	72.33527946
Normal	White	12.4	27.1	170	63	4.180572961	6.709695547	253.6370017	134.7686942
Normal	White	12.8	29.3	170.7	57.6	0.092415497	0.060841519	6.17E-05	0.00601206
Normal	White	12.2	23.7	162.5	55.2	0.020311987	0.613446173	2.087025386	0.000275459
Normal	White	12.3	26.3	161	54	0.618821907	1.282406956	142.5895547	0.252183263
Normal	White	13.4	24.4	160	60	0.020311987	0.613446173	2.087025386	0.000275459
Normal	White	11.9	31.3	165.5	63	1.759948264	110.663424	184.3518633	0.629789935
Normal	White	12.3	29.6	162	63.8	0.648238947	0.170956069	40.9669193	157.8339256

Normal	White	13.1	22.2	167	65	2.169851296	7.166690776	288.5378219	48.60199882
Normal	White	11.8	45.1	164	68	0.618821907	1.282406956	142.5895547	0.252183263
Normal	White	13.1	21.1	154	69.3	0.618821907	1.282406956	142.5895547	0.252183263
Normal	White	12.2	36.9	153	50.5	4.00861349	0.002557265	0.036603836	0.008711746
Normal	White	12	23.8	158	57.9	0.72936366	3.388629009	0.000257766	10.84859942
Normal	White	11.6	21.1	154	53.8	0.092415497	0.060841519	6.17E-05	0.00601206
Normal	White	12.4	34.6	161	59.8	0.251954358	0.128625385	0.013844905	2.329187365
Normal	White	12.5	38.9	154	79.7	3.565910837	0.952643719	335.8641579	72.33527946
Normal	White	11.6	35.9	167.2	62.8	2.169851296	7.166690776	288.5378219	48.60199882
Normal	White	12.8	38	171	71.9	3.565910837	0.952643719	335.8641579	72.33527946
Normal	White	12.8	31.6	166	76.3	3.295126487	47.53328687	0.380011491	0.334692287
Normal	White	12.3	27.5	167	81.8	0.618821907	1.282406956	142.5895547	0.252183263
Normal	White	13.4	32.1	163	71	0.648238947	0.170956069	40.9669193	157.8339256
Normal	White	12.3	38.9	159	60	0.176715723	0.002940006	7.642872435	0.000308719
Normal	White	13.6	18.1	162	72.8	0.092415497	0.060841519	6.17E-05	0.00601206
Normal	White	12.6	26	163	73	3.176441134	10.90333478	0.002849085	268.9775465
Normal	White	13.1	40.1	165	82.3	3.176441134	10.90333478	0.002849085	268.9775465
Normal	White	12.8	38.1	154	51	0.201765231	9.28E-05	3.367934242	1.300935568
Normal	White	13.2	38.8	171	62.2	1.147189178	6.149309885	0.00195811	0.016852717
Normal	White	11.8	31.6	165	59.9	4.180572961	6.709695547	253.6370017	134.7686942
Normal	White	11.5	29.9	174	66.4	0.313012978	11.39411208	0.000243309	0.802508159
Normal	White	12.5	36.3	172	60	0.875915925	0.42339554	164.5471986	0.697413605
Normal	White	12.4	35.3	165.1	52	0.251954358	0.128625385	0.013844905	2.329187365
Normal	White	12	20.5	157	48.3	0.648238947	0.170956069	40.9669193	157.8339256
Normal	White	12.2	37.3	161	79	0.875915925	0.42339554	164.5471986	0.697413605
Normal	White	12.2	36	168	71	3.176441134	10.90333478	0.002849085	268.9775465
Normal	White	13.2	39.5	162.5	52	0.313012978	11.39411208	0.000243309	0.802508159
Normal	White	12.3	39.2	164	59.1	1.147189178	6.149309885	0.00195811	0.016852717
Normal	White	13.9	32	180	72.1	8686.730764	18787.72221	2180.022227	947.1359042
Normal	White	12.3	38.7	158	56	0.72936366	3.388629009	0.000257766	10.84859942
Normal	White	12.3	23.6	164	59	0.092415497	0.060841519	6.17E-05	0.00601206
Normal	White	12.7	32.6	154.9	67	3.295126487	47.53328687	0.380011491	0.334692287
Normal	White	12.6	39.9	175.3	72.2	0.648238947	0.170956069	40.9669193	157.8339256
Normal	White	12.5	33.4	174	73.7	8686.730764	18787.72221	2180.022227	947.1359042
Normal	White	12.8	25.1	172.7	79	0.618821907	1.282406956	142.5895547	0.252183263
Normal	White	12.8	35.8	158	57	0.313012978	11.39411208	0.000243309	0.802508159
Normal	White	12.6	38.9	175	67.8	3.565910837	0.952643719	335.8641579	72.33527946
Normal	White	12.2	37.8	172.7	71.5	0.251954358	0.128625385	0.013844905	2.329187365
Normal	White	12.6	30.5	178	67	1.147189178	6.149309885	0.00195811	0.016852717
Normal	White	11.9	32	162.6	53	0.176715723	0.002940006	7.642872435	0.000308719
Normal	White	13.2	20.7	166	65.3	0.124746359	2.37215778	6.904368641	0.008922686

Normal	White	13.2	39.3	167.6	59	2.169851296	7.166690776	288.5378219	48.60199882
Normal	White	12.9	32.3	169	63	0.648238947	0.170956069	40.9669193	157.8339256
Normal	White	12.5	37.4	162	57	0.201765231	9.28E-05	3.367934242	1.300935568
Normal	White	13.4	38.3	175.1	74	0.618821907	1.282406956	142.5895547	0.252183263
Normal	White	12.5	21.1	179	61.2	1.759948264	110.663424	184.3518633	0.629789935
Normal	White	12.9	40.6	160	70.6	0.648238947	0.170956069	40.9669193	157.8339256
Normal	White	12.6	29.3	166	62.3	1.147189178	6.149309885	0.00195811	0.016852717
Normal	White	13.3	29.6	158	62.3	0.313012978	11.39411208	0.000243309	0.802508159
Normal	White	12.3	36.4	161	65	0.618821907	1.282406956	142.5895547	0.252183263
Normal	White	11.6	35	171	62	0.201765231	9.28E-05	3.367934242	1.300935568
Normal	White	12.2	33.7	172	59.7	0.020311987	0.613446173	2.087025386	0.000275459
Normal	White	12.8	27.1	171	68	8686.730764	18787.72221	2180.022227	947.1359042
Normal	White	12.5	31.9	166	87.5	4.180572961	6.709695547	253.6370017	134.7686942
Normal	White	12	33.3	173	64	0.72936366	3.388629009	0.000257766	10.84859942
Normal	White	12.2	30.4	164	55	0.875915925	0.42339554	164.5471986	0.697413605
Normal	White	13.4	35.2	175	74.3	0.270972897	0.122640088	12.6595894	0.309648549
Normal	White	13	29.8	163	68	0.313012978	11.39411208	0.000243309	0.802508159
Normal	White	12.3	36.5	165	65	0.72936366	3.388629009	0.000257766	10.84859942
Normal	White	12.9	33.4	167	60.5	2.169851296	7.166690776	288.5378219	48.60199882
Normal	White	11.8	35.3	156	61	0.201765231	9.28E-05	3.367934242	1.300935568
Normal	White	11.8	23.2	154.9	52	8686.730764	18787.72221	2180.022227	947.1359042
Normal	White	12.5	33.1	164	57.1	3.565910837	0.952643719	335.8641579	72.33527946
Normal	White	12.7	30.6	162.6	58	0.092415497	0.060841519	6.17E-05	0.00601206
Normal	White	12	33.5	164.7	63.4	4.00861349	0.002557265	0.036603836	0.008711746
Normal	White	13.7	43.9	163	67	4.00861349	0.002557265	0.036603836	0.008711746
Normal	White	13.2	35.9	168	68	3.565910837	0.952643719	335.8641579	72.33527946
Normal	White	12.4	33.7	176	68	3.295126487	47.53328687	0.380011491	0.334692287
Normal	White	11.8	31.6	175.3	69.3	0.648238947	0.170956069	40.9669193	157.8339256
Normal	White	12.7	25.6	169	78.4	0.201765231	9.28E-05	3.367934242	1.300935568
Normal	White	12.1	24	164	60.2	0.124746359	2.37215778	6.904368641	0.008922686
Normal	White	11.5	28.1	173.3	60	2.169851296	7.166690776	288.5378219	48.60199882
Normal	White	13.5	37.6	160	61	0.313012978	11.39411208	0.000243309	0.802508159
Normal	White	12	26.3	155.2	66	1.147189178	6.149309885	0.00195811	0.016852717
Normal	White	12.3	23.5	163	74.5	4.180572961	6.709695547	253.6370017	134.7686942
Normal	White	11.7	30.8	160	58.6	8686.730764	18787.72221	2180.022227	947.1359042
Normal	White	12.6	18.5	164	62.9	0.251954358	0.128625385	0.013844905	2.329187365
Normal	White	11.7	31	157	51	0.176715723	0.002940006	7.642872435	0.000308719
Normal	White	12.7	34.9	162.6	57	0.020311987	0.613446173	2.087025386	0.000275459
Normal	White	13.3	40.8	165	57.4	4.180572961	6.709695547	253.6370017	134.7686942
Normal	White	12.5	26.1	157.5	58	0.618821907	1.282406956	142.5895547	0.252183263
Normal	White	12.4	32.2	170	62.6	4.180572961	6.709695547	253.6370017	134.7686942

Normal	White	13.9	32.2	166	64	0.313012978	11.39411208	0.000243309	0.802508159
Normal	White	12.6	36.4	167.6	65	0.875915925	0.42339554	164.5471986	0.697413605
Normal	White	13	30.1	159.5	65.3	3.565910837	0.952643719	335.8641579	72.33527946
Normal	White	12	34.4	167.6	66	1.147189178	6.149309885	0.00195811	0.016852717
Normal	White	12.2	33.3	153	67	1.759948264	110.663424	184.3518633	0.629789935
Normal	White	13	34.6	157.5	68	0.124746359	2.37215778	6.904368641	0.008922686
Normal	White	12.8	34.9	175	69	4.00861349	0.002557265	0.036603836	0.008711746
Normal	White	13.2	25.4	160	73	0.72936366	3.388629009	0.000257766	10.84859942
Normal	White	13.9	37.5	165	75	8686.730764	18787.72221	2180.022227	947.1359042
Normal	White	12.4	36	183	78.9	0.648238947	0.170956069	40.9669193	157.8339256
Normal	White	13.7	35	170.2	82	0.201765231	9.28E-05	3.367934242	1.300935568
Normal	Black	11.5	28.2	160	68	0.618821907	1.282406956	142.5895547	0.252183263
Normal	Black	11.5	24.3	168	95	8686.730764	18787.72221	2180.022227	947.1359042
Normal	Black	12.2	24.1	153	61	0.618821907	1.282406956	142.5895547	0.252183263
Normal	Black	12.5	39.1	155	62	0.618821907	1.282406956	142.5895547	0.252183263
Normal	Black	12.7	31.5	169	84	0.270972897	0.122640088	12.6595894	0.309648549
Normal	Black	12.6	33.9	142	89	0.251954358	0.128625385	0.013844905	2.329187365
Normal	Black	12.6	23.2	167	59	8686.730764	18787.72221	2180.022227	947.1359042
Normal	Black	12.6	39.5	174	70	4.180572961	6.709695547	253.6370017	134.7686942
Normal	Black	13.5	19	153	51	0.020311987	0.613446173	2.087025386	0.000275459
Normal	Black	12.4	22	159	59	0.618821907	1.282406956	142.5895547	0.252183263
Normal	Black	12.8	22.1	162	94	3.176441134	10.90333478	0.002849085	268.9775465
Normal	Black	12.8	28.2	155	66	4.00861349	0.002557265	0.036603836	0.008711746
Normal	Black	13.2	26.4	165	75	0.270972897	0.122640088	12.6595894	0.309648549
Normal	Black	13	22.3	161	96	3.295126487	47.53328687	0.380011491	0.334692287
Normal	Black	12.5	18.3	157.5	60	0.201765231	9.28E-05	3.367934242	1.300935568
Normal	Black	12.3	32.2	163	65	0.648238947	0.170956069	40.9669193	157.8339256
Normal	Black	12.3	34.4	164	84	0.270972897	0.122640088	12.6595894	0.309648549
Normal	Black	12.7	41.2	166	129	0.251954358	0.128625385	0.013844905	2.329187365
Normal	Black	12.7	28.8	165	66	0.875915925	0.42339554	164.5471986	0.697413605
Normal	Black	13	31.7	162	66	3.565910837	0.952643719	335.8641579	72.33527946
Normal	Black	13.5	31.2	157	72	0.875915925	0.42339554	164.5471986	0.697413605
Normal	Black	13.2	19	167	81	3.176441134	10.90333478	0.002849085	268.9775465
Normal	Black	13.4	22.7	178	81	0.201765231	9.28E-05	3.367934242	1.300935568
Normal	Black	12	40.3	160	74	0.176715723	0.002940006	7.642872435	0.000308719
Normal	Black	12.3	31.3	162.6	83	0.270972897	0.122640088	12.6595894	0.309648549
Normal	Black	12.1	20.7	148	53	0.270972897	0.122640088	12.6595894	0.309648549
Normal	Black	12.5	33.3	165	59	3.565910837	0.952643719	335.8641579	72.33527946
Normal	Black	13.7	36.2	161	81	0.176715723	0.002940006	7.642872435	0.000308719
Normal	Black	13.5	20.6	161	81	1.759948264	110.663424	184.3518633	0.629789935
Normal	Black	11.9	19.9	164	98	1.147189178	6.149309885	0.00195811	0.016852717

Normal	Black	13.1	38.2	170.2	106	2.169851296	7.166690776	288.5378219	48.60199882
Normal	Black	14	36.6	165	71	0.176715723	0.002940006	7.642872435	0.000308719
Normal	Black	11.9	20.2	165.1	76.3	0.875915925	0.42339554	164.5471986	0.697413605
Normal	Black	11.9	25.1	162.6	99	0.020311987	0.613446173	2.087025386	0.000275459
Normal	Black	13.5	31.4	154	65	4.00861349	0.002557265	0.036603836	0.008711746
Normal	Black	12.7	26.6	162	61	0.201765231	9.28E-05	3.367934242	1.300935568
Normal	Black	11.9	18.5	167.6	68	4.180572961	6.709695547	253.6370017	134.7686942
Normal	Black	14	33.2	163	64	0.124746359	2.37215778	6.904368641	0.008922686
Normal	Black	13.4	32.4	162.6	66	3.176441134	10.90333478	0.002849085	268.9775465
Normal	Black	12.8	34.2	167	95	3.565910837	0.952643719	335.8641579	72.33527946
Normal	Black	12.5	35.2	172	73	0.201765231	9.28E-05	3.367934242	1.300935568
Normal	Black	12.6	31.1	172.7	63	1.147189178	6.149309885	0.00195811	0.016852717
Normal	Black	13	30.3	167.6	77	1.147189178	6.149309885	0.00195811	0.016852717
Normal	Black	13.1	29.1	166	49	0.875915925	0.42339554	164.5471986	0.697413605
Normal	Black	11.7	27.4	183	104	4.180572961	6.709695547	253.6370017	134.7686942
Normal	Black	11.7	19.7	170	75	3.295126487	47.53328687	0.380011491	0.334692287
Normal	Black	12.4	28.9	163	70	0.201765231	9.28E-05	3.367934242	1.300935568
Normal	Black	12.7	37.1	172	109	3.176441134	10.90333478	0.002849085	268.9775465
Normal	Black	12.2	34.1	180	89	0.124746359	2.37215778	6.904368641	0.008922686
Normal	Black	13.3	23.1	154	62	0.176715723	0.002940006	7.642872435	0.000308719
Normal	Black	13.2	33.8	160	76	3.176441134	10.90333478	0.002849085	268.9775465
Normal	Black	12.3	18.2	160	61	0.201765231	9.28E-05	3.367934242	1.300935568
Normal	Black	11.7	31.7	157.5	87	1.759948264	110.663424	184.3518633	0.629789935
Normal	Black	12.8	33.1	170	89	1.759948264	110.663424	184.3518633	0.629789935
Normal	Black	13.1	38.4	157.5	65	0.251954358	0.128625385	0.013844905	2.329187365
Normal	Black	13	39.7	182.9	72	3.176441134	10.90333478	0.002849085	268.9775465
Normal	Black	13	18.1	159	55	4.180572961	6.709695547	253.6370017	134.7686942
Normal	Black	13.2	38.6	165.1	54	0.270972897	0.122640088	12.6595894	0.309648549
Normal	Black	12.6	35.5	172.7	60	0.092415497	0.060841519	6.17E-05	0.00601206
Normal	Black	11.4	28.8	182	76	3.176441134	10.90333478	0.002849085	268.9775465
Normal	Black	12.7	32.3	159	70	3.176441134	10.90333478	0.002849085	268.9775465
Normal	Black	13.1	36.6	163	52	4.180572961	6.709695547	253.6370017	134.7686942
Normal	Black	12	18.6	154.9	72	0.251954358	0.128625385	0.013844905	2.329187365
Normal	Black	13.2	30.3	162.6	66	4.180572961	6.709695547	253.6370017	134.7686942
Normal	Black	12.3	32.5	168	90	0.201765231	9.28E-05	3.367934242	1.300935568
Normal	Black	12.4	21.5	162	55	3.295126487	47.53328687	0.380011491	0.334692287
Normal	Black	12.5	19.8	167	63	0.270972897	0.122640088	12.6595894	0.309648549
Normal	Black	12.8	34.1	170	84	0.092415497	0.060841519	6.17E-05	0.00601206
Normal	Black	12.3	40.1	168	115	0.648238947	0.170956069	40.9669193	157.8339256
Normal	Black	12.8	21.5	168	76	3.565910837	0.952643719	335.8641579	72.33527946
Normal	Black	13.9	36	157.5	75	0.313012978	11.39411208	0.000243309	0.802508159

Normal	Black	12.6	27.3	159	57	0.092415497	0.060841519	6.17E-05	0.00601206
Normal	Black	13.5	45	170.2	76	3.565910837	0.952643719	335.8641579	72.33527946
Normal	Black	13.3	27	149.9	69.5	0.092415497	0.060841519	6.17E-05	0.00601206
Normal	Black	12.8	18.5	157.5	53	4.00861349	0.002557265	0.036603836	0.008711746
Normal	Black	13.8	31.1	159	68	4.00861349	0.002557265	0.036603836	0.008711746
Normal	Black	13.2	34.6	172.7	70	0.648238947	0.170956069	40.9669193	157.8339256
Normal	Black	12.5	29.2	161	86	0.251954358	0.128625385	0.013844905	2.329187365
Normal	Black	13.1	39.3	150	50	0.124746359	2.37215778	6.904368641	0.008922686
Normal	Black	13.2	25.3	162	65	0.313012978	11.39411208	0.000243309	0.802508159
Normal	Black	13.4	36.1	160	75	0.251954358	0.128625385	0.013844905	2.329187365
Normal	Black	12.5	22.4	162	59.2	3.176441134	10.90333478	0.002849085	268.9775465
Normal	Black	12.3	26.5	159	74.5	0.875915925	0.42339554	164.5471986	0.697413605
Normal	Black	12.8	28.1	163	91	3.295126487	47.53328687	0.380011491	0.334692287
Normal	Black	12.3	33.1	160	66.7	3.295126487	47.53328687	0.380011491	0.334692287
Normal	Black	11.5	21.5	177.8	79	4.180572961	6.709695547	253.6370017	134.7686942
Normal	Black	12.5	26.2	162.6	69.7	1.759948264	110.663424	184.3518633	0.629789935
Normal	Black	11.9	34.1	170.2	72	4.180572961	6.709695547	253.6370017	134.7686942
Normal	Black	13.2	23.6	160	79	0.251954358	0.128625385	0.013844905	2.329187365
Normal	Black	12.7	21.6	164	56	0.176715723	0.002940006	7.642872435	0.000308719
Normal	Black	13.2	29.4	170	84	0.270972897	0.122640088	12.6595894	0.309648549
Normal	Black	13.3	29.5	159	71	0.618821907	1.282406956	142.5895547	0.252183263
Normal	Black	12.3	27.6	167.6	60	0.201765231	9.28E-05	3.367934242	1.300935568
Normal	Black	12.7	34.9	169	77	0.251954358	0.128625385	0.013844905	2.329187365
Normal	Black	11.7	32.9	167.6	95.6	0.176715723	0.002940006	7.642872435	0.000308719
Normal	Black	12.6	27.7	167	71.9	0.618821907	1.282406956	142.5895547	0.252183263
Normal	Black	12.7	29.9	170.2	76.3	0.72936366	3.388629009	0.000257766	10.84859942
Normal	Black	12.2	24.4	161	60.1	3.295126487	47.53328687	0.380011491	0.334692287
Normal	Black	12.8	24.5	168	102.6	0.648238947	0.170956069	40.9669193	157.8339256
Normal	Black	12.4	28.4	146	63.8	1.759948264	110.663424	184.3518633	0.629789935
Normal	Black	13.3	40.8	160	101.1	3.295126487	47.53328687	0.380011491	0.334692287
Normal	Black	12.7	39.9	154	71.3	1.147189178	6.149309885	0.00195811	0.016852717
Normal	Black	11.9	32.5	163.6	71.6	0.092415497	0.060841519	6.17E-05	0.00601206
Normal	Black	11.7	22.8	148	68	3.565910837	0.952643719	335.8641579	72.33527946
Normal	Black	13.1	24.5	168	80	0.020311987	0.613446173	2.087025386	0.000275459
Normal	Black	12.6	32.9	160	55	0.270972897	0.122640088	12.6595894	0.309648549
Normal	Black	13.3	42.2	166	91.7	0.124746359	2.37215778	6.904368641	0.008922686
Normal	Black	13.7	30.5	163	80.7	0.092415497	0.060841519	6.17E-05	0.00601206
Normal	Black	12.7	35.8	155.3	80.9	3.565910837	0.952643719	335.8641579	72.33527946
Normal	Black	12.8	38.6	160	67.9	0.270972897	0.122640088	12.6595894	0.309648549
Normal	Black	12.4	29.2	171.6	77.8	3.295126487	47.53328687	0.380011491	0.334692287
Normal	Black	12.3	41.2	169	77.4	0.875915925	0.42339554	164.5471986	0.697413605

Normal	Black	12.3	24.7	162.6	82	4.00861349	0.002557265	0.036603836	0.008711746
Normal	Black	13	32.1	169	78.5	4.00861349	0.002557265	0.036603836	0.008711746
Normal	Black	13.8	30.2	174	88.5	0.270972897	0.122640088	12.6595894	0.309648549
Normal	Black	11.8	30.3	162	102	0.875915925	0.42339554	164.5471986	0.697413605
Normal	Black	12.4	28.7	173	117	0.875915925	0.42339554	164.5471986	0.697413605
Normal	Black	12	27.2	161	56	2.169851296	7.166690776	288.5378219	48.60199882
Normal	Black	13	35.5	170.2	79.9	3.565910837	0.952643719	335.8641579	72.33527946
Normal	Black	12.2	20.7	156	72.5	0.618821907	1.282406956	142.5895547	0.252183263
Normal	Black	12.2	35.7	182	88.6	0.72936366	3.388629009	0.000257766	10.84859942
Normal	Black	12	26.1	164	75.9	4.180572961	6.709695547	253.6370017	134.7686942
Normal	Black	13.6	38.3	160.5	83.3	1.759948264	110.663424	184.3518633	0.629789935
Normal	Black	13.3	28.3	165	58	0.313012978	11.39411208	0.000243309	0.802508159
Normal	Black	12.7	41.1	166	60	0.648238947	0.170956069	40.9669193	157.8339256
Normal	Black	13.6	31	166	94	0.124746359	2.37215778	6.904368641	0.008922686
Normal	Black	12.1	34.2	175	68.1	3.176441134	10.90333478	0.002849085	268.9775465
Normal	Black	12.5	27.1	162.1	47	4.00861349	0.002557265	0.036603836	0.008711746
Normal	Black	12	38.8	153	71.7	4.00861349	0.002557265	0.036603836	0.008711746
Normal	Black	12.1	26.4	166	63	4.180572961	6.709695547	253.6370017	134.7686942
Normal	Black	12.9	22.2	167	64.6	0.092415497	0.060841519	6.17E-05	0.00601206
Normal	Black	12.4	21.7	164.5	62	0.618821907	1.282406956	142.5895547	0.252183263
Normal	Black	12.2	28.5	170.2	71.5	0.092415497	0.060841519	6.17E-05	0.00601206
Normal	Black	12.1	21.5	170	76.8	4.00861349	0.002557265	0.036603836	0.008711746
Normal	Black	13.5	35.4	164	85	0.201765231	9.28E-05	3.367934242	1.300935568
Normal	Black	12.9	30.4	161	60	0.648238947	0.170956069	40.9669193	157.8339256
Normal	Black	12.5	29.2	163	67.6	0.875915925	0.42339554	164.5471986	0.697413605
Normal	Black	13	20.8	151	49	0.176715723	0.002940006	7.642872435	0.000308719
Normal	Black	12.3	19.4	154	43.9	0.875915925	0.42339554	164.5471986	0.697413605
Normal	Black	12.7	31.9	157	65	3.176441134	10.90333478	0.002849085	268.9775465
Normal	Black	13	28.5	167	75.6	0.092415497	0.060841519	6.17E-05	0.00601206
Normal	Black	11.7	32.8	162	86	0.092415497	0.060841519	6.17E-05	0.00601206
Normal	Black	12.1	34	170	97	3.565910837	0.952643719	335.8641579	72.33527946
Normal	Black	12.9	30.7	175	100	1.759948264	110.663424	184.3518633	0.629789935
Normal	East Asian	13.4	37.3	154	52	0.875915925	0.42339554	164.5471986	0.697413605
Normal	East Asian	13.1	21.4	157	61	0.618821907	1.282406956	142.5895547	0.252183263
Normal	East Asian	11.8	31.4	152	56	0.251954358	0.128625385	0.013844905	2.329187365
Normal	East Asian	13.6	33	172.7	65	0.092415497	0.060841519	6.17E-05	0.00601206
Normal	East Asian	13.1	29.4	156	74	4.00861349	0.002557265	0.036603836	0.008711746
Normal	East Asian	13.4	43.3	157	57	2.169851296	7.166690776	288.5378219	48.60199882
Normal	East Asian	12.8	29.9	160	60	4.180572961	6.709695547	253.6370017	134.7686942
Normal	East Asian	12	29.9	162.6	60	0.020311987	0.613446173	2.087025386	0.000275459
Normal	East Asian	12.7	24.5	159	64	0.618821907	1.282406956	142.5895547	0.252183263

Normal	East Asian	12	30.6	157	54.9	0.270972897	0.122640088	12.6595894	0.309648549
Normal	East Asian	13.4	33.8	150	56.9	1.759948264	110.663424	184.3518633	0.629789935
Normal	East Asian	13.2	26.6	165.1	60	1.759948264	110.663424	184.3518633	0.629789935
Normal	East Asian	12.4	34	158	67	1.147189178	6.149309885	0.00195811	0.016852717
Normal	East Asian	12.7	37.1	154	52	0.270972897	0.122640088	12.6595894	0.309648549
Normal	East Asian	13.3	33.5	157.5	50.7	1.147189178	6.149309885	0.00195811	0.016852717
Normal	East Asian	12.4	33.1	162	66	0.201765231	9.28E-05	3.367934242	1.300935568
Normal	East Asian	11.7	23.9	163	51.2	0.176715723	0.002940006	7.642872435	0.000308719
Normal	East Asian	12.1	27.2	160	59	3.176441134	10.90333478	0.002849085	268.9775465
Normal	East Asian	12.2	32.9	162	75	0.201765231	9.28E-05	3.367934242	1.300935568
Normal	East Asian	12.4	26	170	50	0.020311987	0.613446173	2.087025386	0.000275459
Normal	East Asian	12.9	30.8	170	71.3	0.251954358	0.128625385	0.013844905	2.329187365
Normal	East Asian	13.1	38.5	153.5	52.6	0.020311987	0.613446173	2.087025386	0.000275459
Normal	East Asian	12.2	40.9	158	53	0.313012978	11.39411208	0.000243309	0.802508159
Normal	East Asian	12.2	26.7	159	57	4.00861349	0.002557265	0.036603836	0.008711746
Normal	Mixed	12.4	35.5	165	62	0.270972897	0.122640088	12.6595894	0.309648549
Normal	Mixed	11.7	19	167.6	61	0.020311987	0.613446173	2.087025386	0.000275459
Normal	Mixed	12.7	42.4	162.6	56	0.72936366	3.388629009	0.000257766	10.84859942
Normal	Mixed	13.1	39.4	150.5	65	0.201765231	9.28E-05	3.367934242	1.300935568
Normal	Mixed	12.7	24.1	154	74	0.201765231	9.28E-05	3.367934242	1.300935568
Normal	Mixed	13.9	27.2	138	57	0.648238947	0.170956069	40.9669193	157.8339256
Normal	Mixed	12.7	36.3	163	63	0.124746359	2.37215778	6.904368641	0.008922686
Normal	Mixed	13.1	21.9	163	66	3.176441134	10.90333478	0.002849085	268.9775465
Normal	Mixed	13.3	41.5	160	75	3.176441134	10.90333478	0.002849085	268.9775465
Normal	Mixed	11.8	24.2	178	82	4.180572961	6.709695547	253.6370017	134.7686942
Normal	Mixed	11.8	30.5	150	69	0.270972897	0.122640088	12.6595894	0.309648549
Normal	Mixed	13.1	25.3	165.1	74	0.176715723	0.002940006	7.642872435	0.000308719
Normal	Mixed	12.8	34.7	168	65	0.092415497	0.060841519	6.17E-05	0.00601206
Normal	Mixed	13.4	43	147.3	55	3.295126487	47.53328687	0.380011491	0.334692287
Normal	Mixed	12.5	34.3	175.3	80	8686.730764	18787.72221	2180.022227	947.1359042
Normal	Mixed	12	33	155	46	4.00861349	0.002557265	0.036603836	0.008711746
Normal	Mixed	11.8	24.8	165	59	0.201765231	9.28E-05	3.367934242	1.300935568
Normal	Mixed	12.9	21.9	170.2	60	0.618821907	1.282406956	142.5895547	0.252183263
Normal	Mixed	13.1	35.4	161	49	1.147189178	6.149309885	0.00195811	0.016852717
Normal	Mixed	12.5	41.1	167.6	50	0.618821907	1.282406956	142.5895547	0.252183263
Normal	Mixed	14.1	34.2	162.6	53	0.020311987	0.613446173	2.087025386	0.000275459
Normal	Mixed	12.4	36	167.6	70	4.00861349	0.002557265	0.036603836	0.008711746
Normal	Mixed	12.9	30.5	163	62	0.176715723	0.002940006	7.642872435	0.000308719
Normal	Mixed	12.8	38.4	155	42	0.020311987	0.613446173	2.087025386	0.000275459
Normal	Mixed	12.3	23.5	162.6	72	0.648238947	0.170956069	40.9669193	157.8339256
Normal	Mixed	13.2	26.5	170.2	72	2.169851296	7.166690776	288.5378219	48.60199882

Normal	Mixed	11.9	29.7	160	74	0.648238947	0.170956069	40.9669193	157.8339256
Normal	Mixed	13.1	19	165.1	49.5	2.169851296	7.166690776	288.5378219	48.60199882
Normal	Mixed	12.2	33.8	165.1	61	3.565910837	0.952643719	335.8641579	72.33527946
Normal	Mixed	13.4	26.8	164	71	0.020311987	0.613446173	2.087025386	0.000275459
Normal	Mixed	12.8	30.1	158	79.1	0.618821907	1.282406956	142.5895547	0.252183263
Normal	Mixed	12.3	34.9	160	59	0.618821907	1.282406956	142.5895547	0.252183263
Normal	Mixed	11.9	27	163	53.7	4.180572961	6.709695547	253.6370017	134.7686942
Normal	Mixed	12.3	34.4	166	55	3.176441134	10.90333478	0.002849085	268.9775465
Normal	Mixed	13.2	23.1	172	69	3.565910837	0.952643719	335.8641579	72.33527946
Normal	Mixed	12.8	31.9	162.6	69	2.169851296	7.166690776	288.5378219	48.60199882
Normal	Mixed	13	37	157.5	46	0.092415497	0.060841519	6.17E-05	0.00601206
Normal	South Asian	12.1	33.2	156	49	0.270972897	0.122640088	12.6595894	0.309648549
Normal	South Asian	12.6	33.1	160	55	1.759948264	110.663424	184.3518633	0.629789935
Normal	South Asian	12	31.5	158	64	1.147189178	6.149309885	0.00195811	0.016852717
Normal	South Asian	12.3	21.7	152	49	3.565910837	0.952643719	335.8641579	72.33527946
Normal	South Asian	12.8	23	159	113	0.72936366	3.388629009	0.000257766	10.84859942
Normal	South Asian	12.1	29.3	162.6	45	0.72936366	3.388629009	0.000257766	10.84859942
Normal	South Asian	11.9	26.2	157	49	0.201765231	9.28E-05	3.367934242	1.300935568
Normal	South Asian	12.6	30.6	159	53	0.201765231	9.28E-05	3.367934242	1.300935568
Normal	South Asian	12.8	39.2	158	54	0.270972897	0.122640088	12.6595894	0.309648549
Normal	South Asian	13.3	38.6	158	49	4.00861349	0.002557265	0.036603836	0.008711746
Normal	South Asian	12.8	33.3	155	54	4.00861349	0.002557265	0.036603836	0.008711746
Normal	South Asian	12.4	24.5	160	59	4.180572961	6.709695547	253.6370017	134.7686942
Normal	South Asian	12	28.1	155	66	0.270972897	0.122640088	12.6595894	0.309648549
Normal	South Asian	11.9	37.3	157	74	3.295126487	47.53328687	0.380011491	0.334692287
Normal	South Asian	12.3	26.3	164	75	3.565910837	0.952643719	335.8641579	72.33527946
Normal	South Asian	12	34.4	165.1	61	3.295126487	47.53328687	0.380011491	0.334692287
Normal	South Asian	12.6	30.1	154.9	62	1.147189178	6.149309885	0.00195811	0.016852717
Normal	South Asian	12.3	25	159	65	0.201765231	9.28E-05	3.367934242	1.300935568
Normal	South Asian	12.5	28.1	159	52	0.270972897	0.122640088	12.6595894	0.309648549
Normal	South Asian	12.9	25.9	159	55	8686.730764	18787.72221	2180.022227	947.1359042
Normal	South Asian	12.9	30.5	175.3	88	1.147189178	6.149309885	0.00195811	0.016852717
Normal	South Asian	13	24.7	152.4	59	3.176441134	10.90333478	0.002849085	268.9775465
Normal	South Asian	12.5	27.5	164	75	3.176441134	10.90333478	0.002849085	268.9775465
Normal	South Asian	11.7	31.8	167	61	0.124746359	2.37215778	6.904368641	0.008922686
Normal	South Asian	13.2	31.5	169	71	3.176441134	10.90333478	0.002849085	268.9775465
Normal	South Asian	12.9	32.7	156	62	1.147189178	6.149309885	0.00195811	0.016852717
Normal	South Asian	12.7	36.8	156	84	0.875915925	0.42339554	164.5471986	0.697413605
Normal	South Asian	12.5	33.5	162.6	50	1.147189178	6.149309885	0.00195811	0.016852717
Normal	South Asian	12.2	26.3	155	65	0.875915925	0.42339554	164.5471986	0.697413605
Normal	South Asian	12.7	36.9	157	50	0.270972897	0.122640088	12.6595894	0.309648549

Normal	South Asian	11.7	21	152.4	51.6	0.092415497	0.060841519	6.17E-05	0.00601206
Normal	South Asian	11.9	34.5	158	55.1	0.251954358	0.128625385	0.013844905	2.329187365
Normal	South Asian	12.1	33.6	154.9	56	0.313012978	11.39411208	0.000243309	0.802508159
Normal	South Asian	12	29.1	153	67	0.72936366	3.388629009	0.000257766	10.84859942
Normal	South Asian	13.5	28.5	158	71.6	0.201765231	9.28E-05	3.367934242	1.300935568
Normal	South Asian	13.3	40.1	159	63	0.124746359	2.37215778	6.904368641	0.008922686
Normal	South Asian	12.3	30.1	159	58.7	1.759948264	110.663424	184.3518633	0.629789935
Normal	South Asian	13.3	38.2	160	67	3.565910837	0.952643719	335.8641579	72.33527946
Normal	South Asian	13.4	28.5	160	63	0.176715723	0.002940006	7.642872435	0.000308719
Normal	South Asian	12.3	25.1	166	74.8	0.875915925	0.42339554	164.5471986	0.697413605
Normal	South Asian	13.4	36.1	160	54	3.295126487	47.53328687	0.380011491	0.334692287
Normal	South Asian	13	36.6	156	69	0.251954358	0.128625385	0.013844905	2.329187365
Normal	South Asian	14.1	24.3	162	56.4	0.020311987	0.613446173	2.087025386	0.000275459
Normal	South Asian	12.7	27.4	151	62	0.313012978	11.39411208	0.000243309	0.802508159
Normal	South Asian	13.3	27.1	152	57	0.020311987	0.613446173	2.087025386	0.000275459
Normal	South Asian	11.8	29.5	159	55.1	0.201765231	9.28E-05	3.367934242	1.300935568
Normal	South Asian	11.8	31.8	159	44	4.00861349	0.002557265	0.036603836	0.008711746
Normal	South Asian	11.6	30.2	165	63	0.020311987	0.613446173	2.087025386	0.000275459
						1	2	3	4
						ENSG00000199633 F2	ENSG00000207147 F2	hsa-let-7d F1	hsa-mir-569 F1

5	6	7	8	9	10	11	12	13
hsa-mir-548l	ENSG00000201980	ENSG00000202231	hsa-mir-216b	hsa-mir-98	hsa-mir-26b	hsa-mir-581 F1	hsa-mir-450b	ENSG00000212363
303.9870933	67.95795037	0.205001333	607.8100429	85.71941897	11.42042502	0	0.007268955	0.010804219
38.38376995	18.04149783	0.094070807	63.40420194	16.57568076	1.111808947	0	1.635865853	0
0.082680879	11.17698316	0.024539241	0.013326891	0.003769332	0	2827.575427	2.947959282	174.4790074
0.756417046	7.409260467	0.000673171	12.79840703	2.610058741	0.01877719	1.267048243	0.487952254	0.208154861
62.33964255	51.19054509	4.114317036	1.921167114	0.892389327	21.78762285	0	0.752651778	0.002527409
0.023597951	0.482833569	25.29002041	0.107985078	0.492930471	0.280648169	0.094716653	0.93239683	0.000927776
31.38964043	1.019166397	21.3980257	4.981724256	17.0187867	0.685350403	0.626228861	1.433492799	0.001555803
303.9870933	67.95795037	0.205001333	607.8100429	85.71941897	11.42042502	0	0.007268955	0.010804219
0.000577626	10.18538835	0.032061697	0.283528801	17.50229411	0	0.969129863	64.66046125	0.187748723
0.215095334	4.38956658	0.703046362	1.073247653	10.78531358	0.029669512	0	0.43544069	0
0.756417046	7.409260467	0.000673171	12.79840703	2.610058741	0.01877719	1.267048243	0.487952254	0.208154861
31.38964043	1.019166397	21.3980257	4.981724256	17.0187867	0.685350403	0.626228861	1.433492799	0.001555803
303.9870933	67.95795037	0.205001333	607.8100429	85.71941897	11.42042502	0	0.007268955	0.010804219
303.9870933	67.95795037	0.205001333	607.8100429	85.71941897	11.42042502	0	0.007268955	0.010804219
0.756417046	7.409260467	0.000673171	12.79840703	2.610058741	0.01877719	1.267048243	0.487952254	0.208154861
0.000398068	1.400749092	9.42E-05	2.036380508	0.823280562	0.005591937	0.021193445	0.145370905	0
0.082680879	11.17698316	0.024539241	0.013326891	0.003769332	0	2827.575427	2.947959282	174.4790074
0.000577626	10.18538835	0.032061697	0.283528801	17.50229411	0	0.969129863	64.66046125	0.187748723
0.082680879	11.17698316	0.024539241	0.013326891	0.003769332	0	2827.575427	2.947959282	174.4790074
0.082015021	42.33216281	0.007880437	44.29033091	0.000756116	10.50163655	0	0.872392725	71.25540454
303.9870933	67.95795037	0.205001333	607.8100429	85.71941897	11.42042502	0	0.007268955	0.010804219
303.9870933	67.95795037	0.205001333	607.8100429	85.71941897	11.42042502	0	0.007268955	0.010804219
0.236342109	48.6183519	4.086559482	24.04518093	10.96225071	0.376425663	632.8895936	1.175535091	50.28623367
25.3382908	0.147582942	162.5357989	3.35728458	16.61087635	0.025561654	0.388461485	0.44904269	9.047860574
0.000577626	10.18538835	0.032061697	0.283528801	17.50229411	0	0.969129863	64.66046125	0.187748723
0.000398068	1.400749092	9.42E-05	2.036380508	0.823280562	0.005591937	0.021193445	0.145370905	0
0.756417046	7.409260467	0.000673171	12.79840703	2.610058741	0.01877719	1.267048243	0.487952254	0.208154861
2.349276622	0.001808294	0.003131765	0.006671814	2.02669023	0.006603021	0	1801.589297	0
25.3382908	0.147582942	162.5357989	3.35728458	16.61087635	0.025561654	0.388461485	0.44904269	9.047860574
25.3382908	0.147582942	162.5357989	3.35728458	16.61087635	0.025561654	0.388461485	0.44904269	9.047860574
7.765495472	6.554063346	4.741150929	0	0.000745927	0.004522465	0.152209404	0.103300884	0
62.33964255	51.19054509	4.114317036	1.921167114	0.892389327	21.78762285	0	0.752651778	0.002527409
0.023597951	0.482833569	25.29002041	0.107985078	0.492930471	0.280648169	0.094716653	0.93239683	0.000927776
25.3382908	0.147582942	162.5357989	3.35728458	16.61087635	0.025561654	0.388461485	0.44904269	9.047860574
0.023597951	0.482833569	25.29002041	0.107985078	0.492930471	0.280648169	0.094716653	0.93239683	0.000927776
4.846952612	1.832540053	0	2.618327402	1.93175734	0.023989459	2.271499668	0.534168297	165.0989224
0.023597951	0.482833569	25.29002041	0.107985078	0.492930471	0.280648169	0.094716653	0.93239683	0.000927776

0.082015021	42.33216281	0.007880437	44.29033091	0.000756116	10.50163655	0	0.872392725	71.25540454
0.008961583	2.261671843	0.058484137	23.86547395	7.64E-05	0.098217756	0	1.705334141	0.001220658
7.765495472	6.554063346	4.741150929	0	0.000745927	0.004522465	0.152209404	0.103300884	0
0.040864975	0.299880357	0.613720197	0.066384168	4.592754005	4.992296395	0	0.082187024	202.7873149
0.215095334	4.38956658	0.703046362	1.073247653	10.78531358	0.029669512	0	0.43544069	0
2.349276622	0.001808294	0.003131765	0.006671814	2.02669023	0.006603021	0	1801.589297	0
1.148543443	1.649420842	0.000189133	2.611523228	2.902931609	0.075990038	0	0.828149401	0
0.756417046	7.409260467	0.000673171	12.79840703	2.610058741	0.01877719	1.267048243	0.487952254	0.208154861
0.082680879	11.17698316	0.024539241	0.013326891	0.003769332	0	2827.575427	2.947959282	174.4790074
25.3382908	0.147582942	162.5357989	3.35728458	16.61087635	0.025561654	0.388461485	0.44904269	9.047860574
31.38964043	1.019166397	21.3980257	4.981724256	17.0187867	0.685350403	0.626228861	1.433492799	0.001555803
19.08678947	6.382309171	1.168120567	0.028919988	8.504562979	0	3.259273909	0.715214851	469.0417534
25.3382908	0.147582942	162.5357989	3.35728458	16.61087635	0.025561654	0.388461485	0.44904269	9.047860574
28.49628011	0.991344866	0.056525884	7.603402523	0.000690954	1.809089301	0.126285588	0.487990346	0.004370079
4.86638069	115.2345147	0.094371895	1.043598804	27.06276051	0.085760223	301.2456423	0.109543666	82.77163117
21.22755434	0.362004239	0.458625717	60.87982682	84.45411124	0.260792482	0	0.200468657	0.028570969
0.041776317	60.62310832	1.039558825	1.234503664	47.21986769	0.033034676	0.453684698	0.220871964	154.4573253
0.202660752	0.267587504	26.53112047	0.355134453	0.687651412	3.95060498	0.093089477	3.908049116	1.600701297
455.7871995	1.073543725	0.032222759	3.003501052	170.0095678	0.263363077	1.510223325	4.689826844	0
13.89972963	0.014078114	217.5251456	0.140033911	0.000468885	162.4022311	0	16.60279417	1255.161428
0.000277632	0.140479681	12.80283743	0.035237556	1.01902267	0.003195477	0.881575129	1.258687003	0
4.86638069	115.2345147	0.094371895	1.043598804	27.06276051	0.085760223	301.2456423	0.109543666	82.77163117
31.55519048	0.037055819	43.22421264	0.604429302	12.12653733	0.073629724	0	0.562789221	2.345622231
0.006830418	1.291992672	0.001294496	4.568261285	3.191565412	0.795624527	0.214944542	1.177884063	0.190483938
13.89972963	0.014078114	217.5251456	0.140033911	0.000468885	162.4022311	0	16.60279417	1255.161428
0.000277632	0.140479681	12.80283743	0.035237556	1.01902267	0.003195477	0.881575129	1.258687003	0
21.22755434	0.362004239	0.458625717	60.87982682	84.45411124	0.260792482	0	0.200468657	0.028570969
21.22755434	0.362004239	0.458625717	60.87982682	84.45411124	0.260792482	0	0.200468657	0.028570969
1.551536401	3.519831878	0.001530975	5.325409069	9.584778975	4.470502157	0.365076679	4.016569328	0.330986488
0	0.057759406	0.11188111	0.18521563	5.42E-06	0.003848417	0.083913861	8.158583921	97.25208044
0.000277632	0.140479681	12.80283743	0.035237556	1.01902267	0.003195477	0.881575129	1.258687003	0
0.000277632	0.140479681	12.80283743	0.035237556	1.01902267	0.003195477	0.881575129	1.258687003	0
28.49628011	0.991344866	0.056525884	7.603402523	0.000690954	1.809089301	0.126285588	0.487990346	0.004370079
0.06621524	10.76762568	5.922218781	0.004727466	59.13728961	21.28584628	9.648578112	2.394832096	228.8283998
455.7871995	1.073543725	0.032222759	3.003501052	170.0095678	0.263363077	1.510223325	4.689826844	0
455.7871995	1.073543725	0.032222759	3.003501052	170.0095678	0.263363077	1.510223325	4.689826844	0
0.202660752	0.267587504	26.53112047	0.355134453	0.687651412	3.95060498	0.093089477	3.908049116	1.600701297
31.55519048	0.037055819	43.22421264	0.604429302	12.12653733	0.073629724	0	0.562789221	2.345622231
0.06621524	10.76762568	5.922218781	0.004727466	59.13728961	21.28584628	9.648578112	2.394832096	228.8283998
0.482199379	0.004768307	0.02096569	0.782719448	0.048339941	4.304443401	0	0.201595404	2.005680679
0.000277632	0.140479681	12.80283743	0.035237556	1.01902267	0.003195477	0.881575129	1.258687003	0

0.067078768	0.06197952	0.858165504	0.583227345	0.000315376	0.276784403	0.041675687	0.210912513	0.003810947
455.7871995	1.073543725	0.032222759	3.003501052	170.0095678	0.263363077	1.510223325	4.689826844	0
21.22755434	0.362004239	0.458625717	60.87982682	84.45411124	0.260792482	0	0.200468657	0.028570969
0.067078768	0.06197952	0.858165504	0.583227345	0.000315376	0.276784403	0.041675687	0.210912513	0.003810947
0.3868333	2.244195808	4.4847836	0.915005983	0.008291391	0.65022329	38.46864448	0.219898314	0.013367053
1.551536401	3.519831878	0.001530975	5.325409069	9.584778975	4.470502157	0.365076679	4.016569328	0.330986488
59508.89074	21551.58748	4301.118895	11.27391116	195972.9386	246.3375064	0	1.387957088	0.010741314
0.3868333	2.244195808	4.4847836	0.915005983	0.008291391	0.65022329	38.46864448	0.219898314	0.013367053
0.067078768	0.06197952	0.858165504	0.583227345	0.000315376	0.276784403	0.041675687	0.210912513	0.003810947
59508.89074	21551.58748	4301.118895	11.27391116	195972.9386	246.3375064	0	1.387957088	0.010741314
0.000277632	0.140479681	12.80283743	0.035237556	1.01902267	0.003195477	0.881575129	1.258687003	0
0.06621524	10.76762568	5.922218781	0.004727466	59.13728961	21.28584628	9.648578112	2.394832096	228.8283998
28.49628011	0.991344866	0.056525884	7.603402523	0.000690954	1.809089301	0.126285588	0.487990346	0.004370079
28.49628011	0.991344866	0.056525884	7.603402523	0.000690954	1.809089301	0.126285588	0.487990346	0.004370079
4.86638069	115.2345147	0.094371895	1.043598804	27.06276051	0.085760223	301.2456423	0.109543666	82.77163117
4.86638069	115.2345147	0.094371895	1.043598804	27.06276051	0.085760223	301.2456423	0.109543666	82.77163117
39.63717417	77.93303846	0.003041902	0.890247112	0.357375193	42.90560519	0.752499175	0.944722788	0
0.000277632	0.140479681	12.80283743	0.035237556	1.01902267	0.003195477	0.881575129	1.258687003	0
0.002944483	0.350527017	0	1.713718644	6.298087843	0.064775353	0	1.652685136	75.1631163
0.041776317	60.62310832	1.039558825	1.234503664	47.21986769	0.033034676	0.453684698	0.220871964	154.4573253
0	0.057759406	0.11188111	0.18521563	5.42E-06	0.003848417	0.083913861	8.158583921	97.25208044
0.3868333	2.244195808	4.4847836	0.915005983	0.008291391	0.65022329	38.46864448	0.219898314	0.013367053
0.202660752	0.267587504	26.53112047	0.355134453	0.687651412	3.95060498	0.093089477	3.908049116	1.600701297
166.2297343	0.027677477	170.570441	58.34376362	69.99236234	187.5554183	0	0.617498982	0.005107595
0.202660752	0.267587504	26.53112047	0.355134453	0.687651412	3.95060498	0.093089477	3.908049116	1.600701297
0	0.057759406	0.11188111	0.18521563	5.42E-06	0.003848417	0.083913861	8.158583921	97.25208044
0.067078768	0.06197952	0.858165504	0.583227345	0.000315376	0.276784403	0.041675687	0.210912513	0.003810947
0.067078768	0.06197952	0.858165504	0.583227345	0.000315376	0.276784403	0.041675687	0.210912513	0.003810947
28.49628011	0.991344866	0.056525884	7.603402523	0.000690954	1.809089301	0.126285588	0.487990346	0.004370079
7.53E-05	0.660942902	0	0.065900288	0.564872126	0.149430295	0	1.004142332	0.006487485
7.53E-05	0.660942902	0	0.065900288	0.564872126	0.149430295	0	1.004142332	0.006487485
0.482199379	0.004768307	0.02096569	0.782719448	0.048339941	4.304443401	0	0.201595404	2.005680679
7.53E-05	0.660942902	0	0.065900288	0.564872126	0.149430295	0	1.004142332	0.006487485
4.86638069	115.2345147	0.094371895	1.043598804	27.06276051	0.085760223	301.2456423	0.109543666	82.77163117
4.86638069	115.2345147	0.094371895	1.043598804	27.06276051	0.085760223	301.2456423	0.109543666	82.77163117
13.89972963	0.014078114	217.5251456	0.140033911	0.000468885	162.4022311	0	16.60279417	1255.161428
1.551536401	3.519831878	0.001530975	5.325409069	9.584778975	4.470502157	0.365076679	4.016569328	0.330986488
0.06621524	10.76762568	5.922218781	0.004727466	59.13728961	21.28584628	9.648578112	2.394832096	228.8283998
28.49628011	0.991344866	0.056525884	7.603402523	0.000690954	1.809089301	0.126285588	0.487990346	0.004370079
1.551536401	3.519831878	0.001530975	5.325409069	9.584778975	4.470502157	0.365076679	4.016569328	0.330986488
0.006830418	1.291992672	0.001294496	4.568261285	3.191565412	0.795624527	0.214944542	1.177884063	0.190483938

0	0.057759406	0.11188111	0.18521563	5.42E-06	0.003848417	0.083913861	8.158583921	97.25208044
455.7871995	1.073543725	0.032222759	3.003501052	170.0095678	0.263363077	1.510223325	4.689826844	0
0.000277632	0.140479681	12.80283743	0.035237556	1.01902267	0.003195477	0.881575129	1.258687003	0
21.22755434	0.362004239	0.458625717	60.87982682	84.45411124	0.260792482	0	0.200468657	0.028570969
13.89972963	0.014078114	217.5251456	0.140033911	0.000468885	162.4022311	0	16.60279417	1255.161428
4.86638069	115.2345147	0.094371895	1.043598804	27.06276051	0.085760223	301.2456423	0.109543666	82.77163117
31.55519048	0.037055819	43.22421264	0.604429302	12.12653733	0.073629724	0	0.562789221	2.345622231
1.551536401	3.519831878	0.001530975	5.325409069	9.584778975	4.470502157	0.365076679	4.016569328	0.330986488
31.55519048	0.037055819	43.22421264	0.604429302	12.12653733	0.073629724	0	0.562789221	2.345622231
1.551536401	3.519831878	0.001530975	5.325409069	9.584778975	4.470502157	0.365076679	4.016569328	0.330986488
4.86638069	115.2345147	0.094371895	1.043598804	27.06276051	0.085760223	301.2456423	0.109543666	82.77163117
455.7871995	1.073543725	0.032222759	3.003501052	170.0095678	0.263363077	1.510223325	4.689826844	0
0.3868333	2.244195808	4.4847836	0.915005983	0.008291391	0.65022329	38.46864448	0.219898314	0.013367053
39.63717417	77.93303846	0.003041902	0.890247112	0.357375193	42.90560519	0.752499175	0.944722788	0
4.86638069	115.2345147	0.094371895	1.043598804	27.06276051	0.085760223	301.2456423	0.109543666	82.77163117
0.002944483	0.350527017	0	1.713718644	6.298087843	0.064775353	0	1.652685136	75.1631163
13.89972963	0.014078114	217.5251456	0.140033911	0.000468885	162.4022311	0	16.60279417	1255.161428
13.89972963	0.014078114	217.5251456	0.140033911	0.000468885	162.4022311	0	16.60279417	1255.161428
0.002944483	0.350527017	0	1.713718644	6.298087843	0.064775353	0	1.652685136	75.1631163
7.53E-05	0.660942902	0	0.065900288	0.564872126	0.149430295	0	1.004142332	0.006487485
28.49628011	0.991344866	0.056525884	7.603402523	0.000690954	1.809089301	0.126285588	0.487990346	0.004370079
0.002944483	0.350527017	0	1.713718644	6.298087843	0.064775353	0	1.652685136	75.1631163
21.22755434	0.362004239	0.458625717	60.87982682	84.45411124	0.260792482	0	0.200468657	0.028570969
0.482199379	0.004768307	0.02096569	0.782719448	0.048339941	4.304443401	0	0.201595404	2.005680679
0.202660752	0.267587504	26.53112047	0.355134453	0.687651412	3.95060498	0.093089477	3.908049116	1.600701297
0.041776317	60.62310832	1.039558825	1.234503664	47.21986769	0.033034676	0.453684698	0.220871964	154.4573253
28.49628011	0.991344866	0.056525884	7.603402523	0.000690954	1.809089301	0.126285588	0.487990346	0.004370079
0.041776317	60.62310832	1.039558825	1.234503664	47.21986769	0.033034676	0.453684698	0.220871964	154.4573253
39.63717417	77.93303846	0.003041902	0.890247112	0.357375193	42.90560519	0.752499175	0.944722788	0
4.86638069	115.2345147	0.094371895	1.043598804	27.06276051	0.085760223	301.2456423	0.109543666	82.77163117
0.006830418	1.291992672	0.001294496	4.568261285	3.191565412	0.795624527	0.214944542	1.177884063	0.190483938
21.22755434	0.362004239	0.458625717	60.87982682	84.45411124	0.260792482	0	0.200468657	0.028570969
0.482199379	0.004768307	0.02096569	0.782719448	0.048339941	4.304443401	0	0.201595404	2.005680679
0.041776317	60.62310832	1.039558825	1.234503664	47.21986769	0.033034676	0.453684698	0.220871964	154.4573253
455.7871995	1.073543725	0.032222759	3.003501052	170.0095678	0.263363077	1.510223325	4.689826844	0
0.3868333	2.244195808	4.4847836	0.915005983	0.008291391	0.65022329	38.46864448	0.219898314	0.013367053
0	0.057759406	0.11188111	0.18521563	5.42E-06	0.003848417	0.083913861	8.158583921	97.25208044
4.86638069	115.2345147	0.094371895	1.043598804	27.06276051	0.085760223	301.2456423	0.109543666	82.77163117
4.86638069	115.2345147	0.094371895	1.043598804	27.06276051	0.085760223	301.2456423	0.109543666	82.77163117
0.06621524	10.76762568	5.922218781	0.004727466	59.13728961	21.28584628	9.648578112	2.394832096	228.8283998
21.22755434	0.362004239	0.458625717	60.87982682	84.45411124	0.260792482	0	0.200468657	0.028570969

0.3868333	2.244195808	4.4847836	0.915005983	0.008291391	0.65022329	38.46864448	0.219898314	0.013367053
7.53E-05	0.660942902	0	0.065900288	0.564872126	0.149430295	0	1.004142332	0.006487485
0.041776317	60.62310832	1.039558825	1.234503664	47.21986769	0.033034676	0.453684698	0.220871964	154.4573253
0.006830418	1.291992672	0.001294496	4.568261285	3.191565412	0.795624527	0.214944542	1.177884063	0.190483938
0.202660752	0.267587504	26.53112047	0.355134453	0.687651412	3.95060498	0.093089477	3.908049116	1.600701297
1.551536401	3.519831878	0.001530975	5.325409069	9.584778975	4.470502157	0.365076679	4.016569328	0.330986488
0.202660752	0.267587504	26.53112047	0.355134453	0.687651412	3.95060498	0.093089477	3.908049116	1.600701297
0	0.057759406	0.11188111	0.18521563	5.42E-06	0.003848417	0.083913861	8.158583921	97.25208044
21.22755434	0.362004239	0.458625717	60.87982682	84.45411124	0.260792482	0	0.200468657	0.028570969
4.86638069	115.2345147	0.094371895	1.043598804	27.06276051	0.085760223	301.2456423	0.109543666	82.77163117
31.55519048	0.037055819	43.22421264	0.604429302	12.12653733	0.073629724	0	0.562789221	2.345622231
455.7871995	1.073543725	0.032222759	3.003501052	170.0095678	0.263363077	1.510223325	4.689826844	0
0.202660752	0.267587504	26.53112047	0.355134453	0.687651412	3.95060498	0.093089477	3.908049116	1.600701297
166.2297343	0.027677477	170.570441	58.34376362	69.99236234	187.5554183	0	0.617498982	0.005107595
455.7871995	1.073543725	0.032222759	3.003501052	170.0095678	0.263363077	1.510223325	4.689826844	0
13.89972963	0.014078114	217.5251456	0.140033911	0.000468885	162.4022311	0	16.60279417	1255.161428
21.22755434	0.362004239	0.458625717	60.87982682	84.45411124	0.260792482	0	0.200468657	0.028570969
31.55519048	0.037055819	43.22421264	0.604429302	12.12653733	0.073629724	0	0.562789221	2.345622231
13.89972963	0.014078114	217.5251456	0.140033911	0.000468885	162.4022311	0	16.60279417	1255.161428
1.551536401	3.519831878	0.001530975	5.325409069	9.584778975	4.470502157	0.365076679	4.016569328	0.330986488
13.89972963	0.014078114	217.5251456	0.140033911	0.000468885	162.4022311	0	16.60279417	1255.161428
0.041776317	60.62310832	1.039558825	1.234503664	47.21986769	0.033034676	0.453684698	0.220871964	154.4573253
0.06621524	10.76762568	5.922218781	0.004727466	59.13728961	21.28584628	9.648578112	2.394832096	228.8283998
166.2297343	0.027677477	170.570441	58.34376362	69.99236234	187.5554183	0	0.617498982	0.005107595
21.22755434	0.362004239	0.458625717	60.87982682	84.45411124	0.260792482	0	0.200468657	0.028570969
0	0.057759406	0.11188111	0.18521563	5.42E-06	0.003848417	0.083913861	8.158583921	97.25208044
0.002944483	0.350527017	0	1.713718644	6.298087843	0.064775353	0	1.652685136	75.1631163
0.002944483	0.350527017	0	1.713718644	6.298087843	0.064775353	0	1.652685136	75.1631163
21.22755434	0.362004239	0.458625717	60.87982682	84.45411124	0.260792482	0	0.200468657	0.028570969
1.551536401	3.519831878	0.001530975	5.325409069	9.584778975	4.470502157	0.365076679	4.016569328	0.330986488
166.2297343	0.027677477	170.570441	58.34376362	69.99236234	187.5554183	0	0.617498982	0.005107595
59508.89074	21551.58748	4301.118895	11.27391116	195972.9386	246.3375064	0	1.387957088	0.010741314
21.22755434	0.362004239	0.458625717	60.87982682	84.45411124	0.260792482	0	0.200468657	0.028570969
39.63717417	77.93303846	0.003041902	0.890247112	0.357375193	42.90560519	0.752499175	0.944722788	0
28.49628011	0.991344866	0.056525884	7.603402523	0.000690954	1.809089301	0.126285588	0.487990346	0.004370079
0.06621524	10.76762568	5.922218781	0.004727466	59.13728961	21.28584628	9.648578112	2.394832096	228.8283998
0.000277632	0.140479681	12.80283743	0.035237556	1.01902267	0.003195477	0.881575129	1.258687003	0
31.55519048	0.037055819	43.22421264	0.604429302	12.12653733	0.073629724	0	0.562789221	2.345622231
21.22755434	0.362004239	0.458625717	60.87982682	84.45411124	0.260792482	0	0.200468657	0.028570969
59508.89074	21551.58748	4301.118895	11.27391116	195972.9386	246.3375064	0	1.387957088	0.010741314
59508.89074	21551.58748	4301.118895	11.27391116	195972.9386	246.3375064	0	1.387957088	0.010741314

166.2297343	0.027677477	170.570441	58.34376362	69.99236234	187.5554183	0	0.617498982	0.005107595
13.89972963	0.014078114	217.5251456	0.140033911	0.000468885	162.4022311	0	16.60279417	1255.161428
166.2297343	0.027677477	170.570441	58.34376362	69.99236234	187.5554183	0	0.617498982	0.005107595
166.2297343	0.027677477	170.570441	58.34376362	69.99236234	187.5554183	0	0.617498982	0.005107595
0.202660752	0.267587504	26.53112047	0.355134453	0.687651412	3.95060498	0.093089477	3.908049116	1.600701297
4.86638069	115.2345147	0.094371895	1.043598804	27.06276051	0.085760223	301.2456423	0.109543666	82.77163117
39.63717417	77.93303846	0.003041902	0.890247112	0.357375193	42.90560519	0.752499175	0.944722788	0
39.63717417	77.93303846	0.003041902	0.890247112	0.357375193	42.90560519	0.752499175	0.944722788	0
13.89972963	0.014078114	217.5251456	0.140033911	0.000468885	162.4022311	0	16.60279417	1255.161428
0.202660752	0.267587504	26.53112047	0.355134453	0.687651412	3.95060498	0.093089477	3.908049116	1.600701297
28.49628011	0.991344866	0.056525884	7.603402523	0.000690954	1.809089301	0.126285588	0.487990346	0.004370079
0.482199379	0.004768307	0.02096569	0.782719448	0.048339941	4.304443401	0	0.201595404	2.005680679
59508.89074	21551.58748	4301.118895	11.27391116	195972.9386	246.3375064	0	1.387957088	0.010741314
21.22755434	0.362004239	0.458625717	60.87982682	84.45411124	0.260792482	0	0.200468657	0.028570969
28.49628011	0.991344866	0.056525884	7.603402523	0.000690954	1.809089301	0.126285588	0.487990346	0.004370079
0.002944483	0.350527017	0	1.713718644	6.298087843	0.064775353	0	1.652685136	75.1631163
31.55519048	0.037055819	43.22421264	0.604429302	12.12653733	0.073629724	0	0.562789221	2.345622231
0.006830418	1.291992672	0.001294496	4.568261285	3.191565412	0.795624527	0.214944542	1.177884063	0.190483938
0.041776317	60.62310832	1.039558825	1.234503664	47.21986769	0.033034676	0.453684698	0.220871964	154.4573253
166.2297343	0.027677477	170.570441	58.34376362	69.99236234	187.5554183	0	0.617498982	0.005107595
0.482199379	0.004768307	0.02096569	0.782719448	0.048339941	4.304443401	0	0.201595404	2.005680679
0.002944483	0.350527017	0	1.713718644	6.298087843	0.064775353	0	1.652685136	75.1631163
0.041776317	60.62310832	1.039558825	1.234503664	47.21986769	0.033034676	0.453684698	0.220871964	154.4573253
13.89972963	0.014078114	217.5251456	0.140033911	0.000468885	162.4022311	0	16.60279417	1255.161428
1.551536401	3.519831878	0.001530975	5.325409069	9.584778975	4.470502157	0.365076679	4.016569328	0.330986488
0	0.057759406	0.11188111	0.18521563	5.42E-06	0.003848417	0.083913861	8.158583921	97.25208044
0.482199379	0.004768307	0.02096569	0.782719448	0.048339941	4.304443401	0	0.201595404	2.005680679
28.49628011	0.991344866	0.056525884	7.603402523	0.000690954	1.809089301	0.126285588	0.487990346	0.004370079
21.22755434	0.362004239	0.458625717	60.87982682	84.45411124	0.260792482	0	0.200468657	0.028570969
0.006830418	1.291992672	0.001294496	4.568261285	3.191565412	0.795624527	0.214944542	1.177884063	0.190483938
0.002944483	0.350527017	0	1.713718644	6.298087843	0.064775353	0	1.652685136	75.1631163
21.22755434	0.362004239	0.458625717	60.87982682	84.45411124	0.260792482	0	0.200468657	0.028570969
39.63717417	77.93303846	0.003041902	0.890247112	0.357375193	42.90560519	0.752499175	0.944722788	0
0.002944483	0.350527017	0	1.713718644	6.298087843	0.064775353	0	1.652685136	75.1631163
31.55519048	0.037055819	43.22421264	0.604429302	12.12653733	0.073629724	0	0.562789221	2.345622231
28.49628011	0.991344866	0.056525884	7.603402523	0.000690954	1.809089301	0.126285588	0.487990346	0.004370079
21.22755434	0.362004239	0.458625717	60.87982682	84.45411124	0.260792482	0	0.200468657	0.028570969
0.006830418	1.291992672	0.001294496	4.568261285	3.191565412	0.795624527	0.214944542	1.177884063	0.190483938
7.53E-05	0.660942902	0	0.065900288	0.564872126	0.149430295	0	1.004142332	0.006487485
39.63717417	77.93303846	0.003041902	0.890247112	0.357375193	42.90560519	0.752499175	0.944722788	0
39.63717417	77.93303846	0.003041902	0.890247112	0.357375193	42.90560519	0.752499175	0.944722788	0

39.63717417	77.93303846	0.003041902	0.890247112	0.357375193	42.90560519	0.752499175	0.944722788	0
0.202660752	0.267587504	26.53112047	0.355134453	0.687651412	3.95060498	0.093089477	3.908049116	1.600701297
0.482199379	0.004768307	0.02096569	0.782719448	0.048339941	4.304443401	0	0.201595404	2.005680679
0.067078768	0.06197952	0.858165504	0.583227345	0.000315376	0.276784403	0.041675687	0.210912513	0.003810947
59508.89074	21551.58748	4301.118895	11.27391116	195972.9386	246.3375064	0	1.387957088	0.010741314
21.22755434	0.362004239	0.458625717	60.87982682	84.45411124	0.260792482	0	0.200468657	0.028570969
0.067078768	0.06197952	0.858165504	0.583227345	0.000315376	0.276784403	0.041675687	0.210912513	0.003810947
31.55519048	0.037055819	43.22421264	0.604429302	12.12653733	0.073629724	0	0.562789221	2.345622231
1.551536401	3.519831878	0.001530975	5.325409069	9.584778975	4.470502157	0.365076679	4.016569328	0.330986488
0.002944483	0.350527017	0	1.713718644	6.298087843	0.064775353	0	1.652685136	75.1631163
0.067078768	0.06197952	0.858165504	0.583227345	0.000315376	0.276784403	0.041675687	0.210912513	0.003810947
0	0.057759406	0.11188111	0.18521563	5.42E-06	0.003848417	0.083913861	8.158583921	97.25208044
39.63717417	77.93303846	0.003041902	0.890247112	0.357375193	42.90560519	0.752499175	0.944722788	0
166.2297343	0.027677477	170.570441	58.34376362	69.99236234	187.5554183	0	0.617498982	0.005107595
7.53E-05	0.660942902	0	0.065900288	0.564872126	0.149430295	0	1.004142332	0.006487485
166.2297343	0.027677477	170.570441	58.34376362	69.99236234	187.5554183	0	0.617498982	0.005107595
4.86638069	115.2345147	0.094371895	1.043598804	27.06276051	0.085760223	301.2456423	0.109543666	82.77163117
0.000277632	0.140479681	12.80283743	0.035237556	1.01902267	0.003195477	0.881575129	1.258687003	0
0.202660752	0.267587504	26.53112047	0.355134453	0.687651412	3.95060498	0.093089477	3.908049116	1.600701297
4.86638069	115.2345147	0.094371895	1.043598804	27.06276051	0.085760223	301.2456423	0.109543666	82.77163117
13.89972963	0.014078114	217.5251456	0.140033911	0.000468885	162.4022311	0	16.60279417	1255.161428
39.63717417	77.93303846	0.003041902	0.890247112	0.357375193	42.90560519	0.752499175	0.944722788	0
0.067078768	0.06197952	0.858165504	0.583227345	0.000315376	0.276784403	0.041675687	0.210912513	0.003810947
13.89972963	0.014078114	217.5251456	0.140033911	0.000468885	162.4022311	0	16.60279417	1255.161428
21.22755434	0.362004239	0.458625717	60.87982682	84.45411124	0.260792482	0	0.200468657	0.028570969
0.202660752	0.267587504	26.53112047	0.355134453	0.687651412	3.95060498	0.093089477	3.908049116	1.600701297
28.49628011	0.991344866	0.056525884	7.603402523	0.000690954	1.809089301	0.126285588	0.487990346	0.004370079
0.202660752	0.267587504	26.53112047	0.355134453	0.687651412	3.95060498	0.093089477	3.908049116	1.600701297
13.89972963	0.014078114	217.5251456	0.140033911	0.000468885	162.4022311	0	16.60279417	1255.161428
0.006830418	1.291992672	0.001294496	4.568261285	3.191565412	0.795624527	0.214944542	1.177884063	0.190483938
166.2297343	0.027677477	170.570441	58.34376362	69.99236234	187.5554183	0	0.617498982	0.005107595
7.53E-05	0.660942902	0	0.065900288	0.564872126	0.149430295	0	1.004142332	0.006487485
0.002944483	0.350527017	0	1.713718644	6.298087843	0.064775353	0	1.652685136	75.1631163
0.006830418	1.291992672	0.001294496	4.568261285	3.191565412	0.795624527	0.214944542	1.177884063	0.190483938
0.202660752	0.267587504	26.53112047	0.355134453	0.687651412	3.95060498	0.093089477	3.908049116	1.600701297
0.482199379	0.004768307	0.02096569	0.782719448	0.048339941	4.304443401	0	0.201595404	2.005680679
166.2297343	0.027677477	170.570441	58.34376362	69.99236234	187.5554183	0	0.617498982	0.005107595
28.49628011	0.991344866	0.056525884	7.603402523	0.000690954	1.809089301	0.126285588	0.487990346	0.004370079
0.041776317	60.62310832	1.039558825	1.234503664	47.21986769	0.033034676	0.453684698	0.220871964	154.4573253
0	0.057759406	0.11188111	0.18521563	5.42E-06	0.003848417	0.083913861	8.158583921	97.25208044
13.89972963	0.014078114	217.5251456	0.140033911	0.000468885	162.4022311	0	16.60279417	1255.161428

0.006830418	1.291992672	0.001294496	4.568261285	3.191565412	0.795624527	0.214944542	1.177884063	0.190483938
455.7871995	1.073543725	0.032222759	3.003501052	170.0095678	0.263363077	1.510223325	4.689826844	0
28.49628011	0.991344866	0.056525884	7.603402523	0.000690954	1.809089301	0.126285588	0.487990346	0.004370079
31.55519048	0.037055819	43.22421264	0.604429302	12.12653733	0.073629724	0	0.562789221	2.345622231
0.000277632	0.140479681	12.80283743	0.035237556	1.01902267	0.003195477	0.881575129	1.258687003	0
39.63717417	77.93303846	0.003041902	0.890247112	0.357375193	42.90560519	0.752499175	0.944722788	0
1.551536401	3.519831878	0.001530975	5.325409069	9.584778975	4.470502157	0.365076679	4.016569328	0.330986488
0.000277632	0.140479681	12.80283743	0.035237556	1.01902267	0.003195477	0.881575129	1.258687003	0
39.63717417	77.93303846	0.003041902	0.890247112	0.357375193	42.90560519	0.752499175	0.944722788	0
455.7871995	1.073543725	0.032222759	3.003501052	170.0095678	0.263363077	1.510223325	4.689826844	0
0.006830418	1.291992672	0.001294496	4.568261285	3.191565412	0.795624527	0.214944542	1.177884063	0.190483938
21.22755434	0.362004239	0.458625717	60.87982682	84.45411124	0.260792482	0	0.200468657	0.028570969
31.55519048	0.037055819	43.22421264	0.604429302	12.12653733	0.073629724	0	0.562789221	2.345622231
39.63717417	77.93303846	0.003041902	0.890247112	0.357375193	42.90560519	0.752499175	0.944722788	0
31.55519048	0.037055819	43.22421264	0.604429302	12.12653733	0.073629724	0	0.562789221	2.345622231
0.202660752	0.267587504	26.53112047	0.355134453	0.687651412	3.95060498	0.093089477	3.908049116	1.600701297
28.49628011	0.991344866	0.056525884	7.603402523	0.000690954	1.809089301	0.126285588	0.487990346	0.004370079
0	0.057759406	0.11188111	0.18521563	5.42E-06	0.003848417	0.083913861	8.158583921	97.25208044
0.002944483	0.350527017	0	1.713718644	6.298087843	0.064775353	0	1.652685136	75.1631163
166.2297343	0.027677477	170.570441	58.34376362	69.99236234	187.5554183	0	0.617498982	0.005107595
39.63717417	77.93303846	0.003041902	0.890247112	0.357375193	42.90560519	0.752499175	0.944722788	0
28.49628011	0.991344866	0.056525884	7.603402523	0.000690954	1.809089301	0.126285588	0.487990346	0.004370079
59508.89074	21551.58748	4301.118895	11.27391116	195972.9386	246.3375064	0	1.387957088	0.010741314
59508.89074	21551.58748	4301.118895	11.27391116	195972.9386	246.3375064	0	1.387957088	0.010741314
31.55519048	0.037055819	43.22421264	0.604429302	12.12653733	0.073629724	0	0.562789221	2.345622231
4.86638069	115.2345147	0.094371895	1.043598804	27.06276051	0.085760223	301.2456423	0.109543666	82.77163117
166.2297343	0.027677477	170.570441	58.34376362	69.99236234	187.5554183	0	0.617498982	0.005107595
0.482199379	0.004768307	0.02096569	0.782719448	0.048339941	4.304443401	0	0.201595404	2.005680679
28.49628011	0.991344866	0.056525884	7.603402523	0.000690954	1.809089301	0.126285588	0.487990346	0.004370079
455.7871995	1.073543725	0.032222759	3.003501052	170.0095678	0.263363077	1.510223325	4.689826844	0
0.482199379	0.004768307	0.02096569	0.782719448	0.048339941	4.304443401	0	0.201595404	2.005680679
0.067078768	0.06197952	0.858165504	0.583227345	0.000315376	0.276784403	0.041675687	0.210912513	0.003810947
28.49628011	0.991344866	0.056525884	7.603402523	0.000690954	1.809089301	0.126285588	0.487990346	0.004370079
0.041776317	60.62310832	1.039558825	1.234503664	47.21986769	0.033034676	0.453684698	0.220871964	154.4573253
0.000277632	0.140479681	12.80283743	0.035237556	1.01902267	0.003195477	0.881575129	1.258687003	0
31.55519048	0.037055819	43.22421264	0.604429302	12.12653733	0.073629724	0	0.562789221	2.345622231
0.006830418	1.291992672	0.001294496	4.568261285	3.191565412	0.795624527	0.214944542	1.177884063	0.190483938
0.482199379	0.004768307	0.02096569	0.782719448	0.048339941	4.304443401	0	0.201595404	2.005680679
0.482199379	0.004768307	0.02096569	0.782719448	0.048339941	4.304443401	0	0.201595404	2.005680679
39.63717417	77.93303846	0.003041902	0.890247112	0.357375193	42.90560519	0.752499175	0.944722788	0
7.53E-05	0.660942902	0	0.065900288	0.564872126	0.149430295	0	1.004142332	0.006487485

31.55519048	0.037055819	43.22421264	0.604429302	12.12653733	0.073629724	0	0.562789221	2.345622231
455.7871995	1.073543725	0.032222759	3.003501052	170.0095678	0.263363077	1.510223325	4.689826844	0
0.002944483	0.350527017	0	1.713718644	6.298087843	0.064775353	0	1.652685136	75.1631163
28.49628011	0.991344866	0.056525884	7.603402523	0.000690954	1.809089301	0.126285588	0.487990346	0.004370079
166.2297343	0.027677477	170.570441	58.34376362	69.99236234	187.5554183	0	0.617498982	0.005107595
0.041776317	60.62310832	1.039558825	1.234503664	47.21986769	0.033034676	0.453684698	0.220871964	154.4573253
28.49628011	0.991344866	0.056525884	7.603402523	0.000690954	1.809089301	0.126285588	0.487990346	0.004370079
455.7871995	1.073543725	0.032222759	3.003501052	170.0095678	0.263363077	1.510223325	4.689826844	0
0.482199379	0.004768307	0.02096569	0.782719448	0.048339941	4.304443401	0	0.201595404	2.005680679
0.006830418	1.291992672	0.001294496	4.568261285	3.191565412	0.795624527	0.214944542	1.177884063	0.190483938
0.067078768	0.06197952	0.858165504	0.583227345	0.000315376	0.276784403	0.041675687	0.210912513	0.003810947
0.202660752	0.267587504	26.53112047	0.355134453	0.687651412	3.95060498	0.093089477	3.908049116	1.600701297
21.22755434	0.362004239	0.458625717	60.87982682	84.45411124	0.260792482	0	0.200468657	0.028570969
0.202660752	0.267587504	26.53112047	0.355134453	0.687651412	3.95060498	0.093089477	3.908049116	1.600701297
0.202660752	0.267587504	26.53112047	0.355134453	0.687651412	3.95060498	0.093089477	3.908049116	1.600701297
59508.89074	21551.58748	4301.118895	11.27391116	195972.9386	246.3375064	0	1.387957088	0.010741314
455.7871995	1.073543725	0.032222759	3.003501052	170.0095678	0.263363077	1.510223325	4.689826844	0
59508.89074	21551.58748	4301.118895	11.27391116	195972.9386	246.3375064	0	1.387957088	0.010741314
0.3868333	2.244195808	4.4847836	0.915005983	0.008291391	0.65022329	38.46864448	0.219898314	0.013367053
7.53E-05	0.660942902	0	0.065900288	0.564872126	0.149430295	0	1.004142332	0.006487485
0.06621524	10.76762568	5.922218781	0.004727466	59.13728961	21.28584628	9.648578112	2.394832096	228.8283998
0.000277632	0.140479681	12.80283743	0.035237556	1.01902267	0.003195477	0.881575129	1.258687003	0
0.06621524	10.76762568	5.922218781	0.004727466	59.13728961	21.28584628	9.648578112	2.394832096	228.8283998
7.53E-05	0.660942902	0	0.065900288	0.564872126	0.149430295	0	1.004142332	0.006487485
1.551536401	3.519831878	0.001530975	5.325409069	9.584778975	4.470502157	0.365076679	4.016569328	0.330986488
0.482199379	0.004768307	0.02096569	0.782719448	0.048339941	4.304443401	0	0.201595404	2.005680679
166.2297343	0.027677477	170.570441	58.34376362	69.99236234	187.5554183	0	0.617498982	0.005107595
21.22755434	0.362004239	0.458625717	60.87982682	84.45411124	0.260792482	0	0.200468657	0.028570969
0.000277632	0.140479681	12.80283743	0.035237556	1.01902267	0.003195477	0.881575129	1.258687003	0
0.482199379	0.004768307	0.02096569	0.782719448	0.048339941	4.304443401	0	0.201595404	2.005680679
166.2297343	0.027677477	170.570441	58.34376362	69.99236234	187.5554183	0	0.617498982	0.005107595
0.006830418	1.291992672	0.001294496	4.568261285	3.191565412	0.795624527	0.214944542	1.177884063	0.190483938
13.89972963	0.014078114	217.5251456	0.140033911	0.000468885	162.4022311	0	16.60279417	1255.161428
166.2297343	0.027677477	170.570441	58.34376362	69.99236234	187.5554183	0	0.617498982	0.005107595
455.7871995	1.073543725	0.032222759	3.003501052	170.0095678	0.263363077	1.510223325	4.689826844	0
0.202660752	0.267587504	26.53112047	0.355134453	0.687651412	3.95060498	0.093089477	3.908049116	1.600701297
0.067078768	0.06197952	0.858165504	0.583227345	0.000315376	0.276784403	0.041675687	0.210912513	0.003810947
0.002944483	0.350527017	0	1.713718644	6.298087843	0.064775353	0	1.652685136	75.1631163
59508.89074	21551.58748	4301.118895	11.27391116	195972.9386	246.3375064	0	1.387957088	0.010741314
7.53E-05	0.660942902	0	0.065900288	0.564872126	0.149430295	0	1.004142332	0.006487485
0.006830418	1.291992672	0.001294496	4.568261285	3.191565412	0.795624527	0.214944542	1.177884063	0.190483938

1.551536401	3.519831878	0.001530975	5.325409069	9.584778975	4.470502157	0.365076679	4.016569328	0.330986488
455.7871995	1.073543725	0.032222759	3.003501052	170.0095678	0.263363077	1.510223325	4.689826844	0
166.2297343	0.027677477	170.570441	58.34376362	69.99236234	187.5554183	0	0.617498982	0.005107595
0.482199379	0.004768307	0.02096569	0.782719448	0.048339941	4.304443401	0	0.201595404	2.005680679
0.002944483	0.350527017	0	1.713718644	6.298087843	0.064775353	0	1.652685136	75.1631163
7.53E-05	0.660942902	0	0.065900288	0.564872126	0.149430295	0	1.004142332	0.006487485
31.55519048	0.037055819	43.22421264	0.604429302	12.12653733	0.073629724	0	0.562789221	2.345622231
1.551536401	3.519831878	0.001530975	5.325409069	9.584778975	4.470502157	0.365076679	4.016569328	0.330986488
0.482199379	0.004768307	0.02096569	0.782719448	0.048339941	4.304443401	0	0.201595404	2.005680679
0.06621524	10.76762568	5.922218781	0.004727466	59.13728961	21.28584628	9.648578112	2.394832096	228.8283998
28.49628011	0.991344866	0.056525884	7.603402523	0.000690954	1.809089301	0.126285588	0.487990346	0.004370079
7.53E-05	0.660942902	0	0.065900288	0.564872126	0.149430295	0	1.004142332	0.006487485
0.067078768	0.06197952	0.858165504	0.583227345	0.000315376	0.276784403	0.041675687	0.210912513	0.003810947
13.89972963	0.014078114	217.5251456	0.140033911	0.000468885	162.4022311	0	16.60279417	1255.161428
1.551536401	3.519831878	0.001530975	5.325409069	9.584778975	4.470502157	0.365076679	4.016569328	0.330986488
0.000277632	0.140479681	12.80283743	0.035237556	1.01902267	0.003195477	0.881575129	1.258687003	0
39.63717417	77.93303846	0.003041902	0.890247112	0.357375193	42.90560519	0.752499175	0.944722788	0
28.49628011	0.991344866	0.056525884	7.603402523	0.000690954	1.809089301	0.126285588	0.487990346	0.004370079
28.49628011	0.991344866	0.056525884	7.603402523	0.000690954	1.809089301	0.126285588	0.487990346	0.004370079
0.482199379	0.004768307	0.02096569	0.782719448	0.048339941	4.304443401	0	0.201595404	2.005680679
0.482199379	0.004768307	0.02096569	0.782719448	0.048339941	4.304443401	0	0.201595404	2.005680679
4.86638069	115.2345147	0.094371895	1.043598804	27.06276051	0.085760223	301.2456423	0.109543666	82.77163117
0.000277632	0.140479681	12.80283743	0.035237556	1.01902267	0.003195477	0.881575129	1.258687003	0
455.7871995	1.073543725	0.032222759	3.003501052	170.0095678	0.263363077	1.510223325	4.689826844	0
13.89972963	0.014078114	217.5251456	0.140033911	0.000468885	162.4022311	0	16.60279417	1255.161428
166.2297343	0.027677477	170.570441	58.34376362	69.99236234	187.5554183	0	0.617498982	0.005107595
0.000277632	0.140479681	12.80283743	0.035237556	1.01902267	0.003195477	0.881575129	1.258687003	0
0.067078768	0.06197952	0.858165504	0.583227345	0.000315376	0.276784403	0.041675687	0.210912513	0.003810947
0.041776317	60.62310832	1.039558825	1.234503664	47.21986769	0.033034676	0.453684698	0.220871964	154.4573253
13.89972963	0.014078114	217.5251456	0.140033911	0.000468885	162.4022311	0	16.60279417	1255.161428
39.63717417	77.93303846	0.003041902	0.890247112	0.357375193	42.90560519	0.752499175	0.944722788	0
0.006830418	1.291992672	0.001294496	4.568261285	3.191565412	0.795624527	0.214944542	1.177884063	0.190483938
166.2297343	0.027677477	170.570441	58.34376362	69.99236234	187.5554183	0	0.617498982	0.005107595
1.551536401	3.519831878	0.001530975	5.325409069	9.584778975	4.470502157	0.365076679	4.016569328	0.330986488
166.2297343	0.027677477	170.570441	58.34376362	69.99236234	187.5554183	0	0.617498982	0.005107595
0.06621524	10.76762568	5.922218781	0.004727466	59.13728961	21.28584628	9.648578112	2.394832096	228.8283998
1.551536401	3.519831878	0.001530975	5.325409069	9.584778975	4.470502157	0.365076679	4.016569328	0.330986488
1.551536401	3.519831878	0.001530975	5.325409069	9.584778975	4.470502157	0.365076679	4.016569328	0.330986488
0.006830418	1.291992672	0.001294496	4.568261285	3.191565412	0.795624527	0.214944542	1.177884063	0.190483938
31.55519048	0.037055819	43.22421264	0.604429302	12.12653733	0.073629724	0	0.562789221	2.345622231
21.22755434	0.362004239	0.458625717	60.87982682	84.45411124	0.260792482	0	0.200468657	0.028570969

1.551536401	3.519831878	0.001530975	5.325409069	9.584778975	4.470502157	0.365076679	4.016569328	0.330986488
0.067078768	0.06197952	0.858165504	0.583227345	0.000315376	0.276784403	0.041675687	0.210912513	0.003810947
1.551536401	3.519831878	0.001530975	5.325409069	9.584778975	4.470502157	0.365076679	4.016569328	0.330986488
455.7871995	1.073543725	0.032222759	3.003501052	170.0095678	0.263363077	1.510223325	4.689826844	0
1.551536401	3.519831878	0.001530975	5.325409069	9.584778975	4.470502157	0.365076679	4.016569328	0.330986488
0	0.057759406	0.11188111	0.18521563	5.42E-06	0.003848417	0.083913861	8.158583921	97.25208044
455.7871995	1.073543725	0.032222759	3.003501052	170.0095678	0.263363077	1.510223325	4.689826844	0
0.041776317	60.62310832	1.039558825	1.234503664	47.21986769	0.033034676	0.453684698	0.220871964	154.4573253
7.53E-05	0.660942902	0	0.065900288	0.564872126	0.149430295	0	1.004142332	0.006487485
0.3868333	2.244195808	4.4847836	0.915005983	0.008291391	0.65022329	38.46864448	0.219898314	0.013367053
0.000277632	0.140479681	12.80283743	0.035237556	1.01902267	0.003195477	0.881575129	1.258687003	0
0.202660752	0.267587504	26.53112047	0.355134453	0.687651412	3.95060498	0.093089477	3.908049116	1.600701297
0	0.057759406	0.11188111	0.18521563	5.42E-06	0.003848417	0.083913861	8.158583921	97.25208044
59508.89074	21551.58748	4301.118895	11.27391116	195972.9386	246.3375064	0	1.387957088	0.010741314
0.06621524	10.76762568	5.922218781	0.004727466	59.13728961	21.28584628	9.648578112	2.394832096	228.8283998
0	0.057759406	0.11188111	0.18521563	5.42E-06	0.003848417	0.083913861	8.158583921	97.25208044
0.000277632	0.140479681	12.80283743	0.035237556	1.01902267	0.003195477	0.881575129	1.258687003	0
59508.89074	21551.58748	4301.118895	11.27391116	195972.9386	246.3375064	0	1.387957088	0.010741314
0.06621524	10.76762568	5.922218781	0.004727466	59.13728961	21.28584628	9.648578112	2.394832096	228.8283998
28.49628011	0.991344866	0.056525884	7.603402523	0.000690954	1.809089301	0.126285588	0.487990346	0.004370079
0	0.057759406	0.11188111	0.18521563	5.42E-06	0.003848417	0.083913861	8.158583921	97.25208044
0.202660752	0.267587504	26.53112047	0.355134453	0.687651412	3.95060498	0.093089477	3.908049116	1.600701297
0.3868333	2.244195808	4.4847836	0.915005983	0.008291391	0.65022329	38.46864448	0.219898314	0.013367053
0.041776317	60.62310832	1.039558825	1.234503664	47.21986769	0.033034676	0.453684698	0.220871964	154.4573253
28.49628011	0.991344866	0.056525884	7.603402523	0.000690954	1.809089301	0.126285588	0.487990346	0.004370079
0.202660752	0.267587504	26.53112047	0.355134453	0.687651412	3.95060498	0.093089477	3.908049116	1.600701297
21.22755434	0.362004239	0.458625717	60.87982682	84.45411124	0.260792482	0	0.200468657	0.028570969
0.202660752	0.267587504	26.53112047	0.355134453	0.687651412	3.95060498	0.093089477	3.908049116	1.600701297
455.7871995	1.073543725	0.032222759	3.003501052	170.0095678	0.263363077	1.510223325	4.689826844	0
0.002944483	0.350527017	0	1.713718644	6.298087843	0.064775353	0	1.652685136	75.1631163
455.7871995	1.073543725	0.032222759	3.003501052	170.0095678	0.263363077	1.510223325	4.689826844	0
0.006830418	1.291992672	0.001294496	4.568261285	3.191565412	0.795624527	0.214944542	1.177884063	0.190483938
166.2297343	0.027677477	170.570441	58.34376362	69.99236234	187.5554183	0	0.617498982	0.005107595
59508.89074	21551.58748	4301.118895	11.27391116	195972.9386	246.3375064	0	1.387957088	0.010741314
0.06621524	10.76762568	5.922218781	0.004727466	59.13728961	21.28584628	9.648578112	2.394832096	228.8283998
0.041776317	60.62310832	1.039558825	1.234503664	47.21986769	0.033034676	0.453684698	0.220871964	154.4573253
0.202660752	0.267587504	26.53112047	0.355134453	0.687651412	3.95060498	0.093089477	3.908049116	1.600701297
0.3868333	2.244195808	4.4847836	0.915005983	0.008291391	0.65022329	38.46864448	0.219898314	0.013367053
0.067078768	0.06197952	0.858165504	0.583227345	0.000315376	0.276784403	0.041675687	0.210912513	0.003810947
0.041776317	60.62310832	1.039558825	1.234503664	47.21986769	0.033034676	0.453684698	0.220871964	154.4573253
0.482199379	0.004768307	0.02096569	0.782719448	0.048339941	4.304443401	0	0.201595404	2.005680679

1.551536401	3.519831878	0.001530975	5.325409069	9.584778975	4.470502157	0.365076679	4.016569328	0.330986488
0.002944483	0.350527017	0	1.713718644	6.298087843	0.064775353	0	1.652685136	75.1631163
0.06621524	10.76762568	5.922218781	0.004727466	59.13728961	21.28584628	9.648578112	2.394832096	228.8283998
39.63717417	77.93303846	0.003041902	0.890247112	0.357375193	42.90560519	0.752499175	0.944722788	0
4.86638069	115.2345147	0.094371895	1.043598804	27.06276051	0.085760223	301.2456423	0.109543666	82.77163117
21.22755434	0.362004239	0.458625717	60.87982682	84.45411124	0.260792482	0	0.200468657	0.028570969
0.482199379	0.004768307	0.02096569	0.782719448	0.048339941	4.304443401	0	0.201595404	2.005680679
28.49628011	0.991344866	0.056525884	7.603402523	0.000690954	1.809089301	0.126285588	0.487990346	0.004370079
7.53E-05	0.660942902	0	0.065900288	0.564872126	0.149430295	0	1.004142332	0.006487485
21.22755434	0.362004239	0.458625717	60.87982682	84.45411124	0.260792482	0	0.200468657	0.028570969
0.002944483	0.350527017	0	1.713718644	6.298087843	0.064775353	0	1.652685136	75.1631163
21.22755434	0.362004239	0.458625717	60.87982682	84.45411124	0.260792482	0	0.200468657	0.028570969
0.000277632	0.140479681	12.80283743	0.035237556	1.01902267	0.003195477	0.881575129	1.258687003	0
4.86638069	115.2345147	0.094371895	1.043598804	27.06276051	0.085760223	301.2456423	0.109543666	82.77163117
7.53E-05	0.660942902	0	0.065900288	0.564872126	0.149430295	0	1.004142332	0.006487485
0.3868333	2.244195808	4.4847836	0.915005983	0.008291391	0.65022329	38.46864448	0.219898314	0.013367053
0	0.057759406	0.11188111	0.18521563	5.42E-06	0.003848417	0.083913861	8.158583921	97.25208044
0	0.057759406	0.11188111	0.18521563	5.42E-06	0.003848417	0.083913861	8.158583921	97.25208044
455.7871995	1.073543725	0.032222759	3.003501052	170.0095678	0.263363077	1.510223325	4.689826844	0
13.89972963	0.014078114	217.5251456	0.140033911	0.000468885	162.4022311	0	16.60279417	1255.161428
1.551536401	3.519831878	0.001530975	5.325409069	9.584778975	4.470502157	0.365076679	4.016569328	0.330986488
0.002944483	0.350527017	0	1.713718644	6.298087843	0.064775353	0	1.652685136	75.1631163
455.7871995	1.073543725	0.032222759	3.003501052	170.0095678	0.263363077	1.510223325	4.689826844	0
59508.89074	21551.58748	4301.118895	11.27391116	195972.9386	246.3375064	0	1.387957088	0.010741314
0.067078768	0.06197952	0.858165504	0.583227345	0.000315376	0.276784403	0.041675687	0.210912513	0.003810947
0.3868333	2.244195808	4.4847836	0.915005983	0.008291391	0.65022329	38.46864448	0.219898314	0.013367053
0.000277632	0.140479681	12.80283743	0.035237556	1.01902267	0.003195477	0.881575129	1.258687003	0
0.006830418	1.291992672	0.001294496	4.568261285	3.191565412	0.795624527	0.214944542	1.177884063	0.190483938
0.002944483	0.350527017	0	1.713718644	6.298087843	0.064775353	0	1.652685136	75.1631163
59508.89074	21551.58748	4301.118895	11.27391116	195972.9386	246.3375064	0	1.387957088	0.010741314
7.53E-05	0.660942902	0	0.065900288	0.564872126	0.149430295	0	1.004142332	0.006487485
4.86638069	115.2345147	0.094371895	1.043598804	27.06276051	0.085760223	301.2456423	0.109543666	82.77163117
13.89972963	0.014078114	217.5251456	0.140033911	0.000468885	162.4022311	0	16.60279417	1255.161428
0.06621524	10.76762568	5.922218781	0.004727466	59.13728961	21.28584628	9.648578112	2.394832096	228.8283998
0.067078768	0.06197952	0.858165504	0.583227345	0.000315376	0.276784403	0.041675687	0.210912513	0.003810947
0.041776317	60.62310832	1.039558825	1.234503664	47.21986769	0.033034676	0.453684698	0.220871964	154.4573253
0	0.057759406	0.11188111	0.18521563	5.42E-06	0.003848417	0.083913861	8.158583921	97.25208044
0.067078768	0.06197952	0.858165504	0.583227345	0.000315376	0.276784403	0.041675687	0.210912513	0.003810947
59508.89074	21551.58748	4301.118895	11.27391116	195972.9386	246.3375064	0	1.387957088	0.010741314
7.53E-05	0.660942902	0	0.065900288	0.564872126	0.149430295	0	1.004142332	0.006487485
0	0.057759406	0.11188111	0.18521563	5.42E-06	0.003848417	0.083913861	8.158583921	97.25208044

4.86638069	115.2345147	0.094371895	1.043598804	27.06276051	0.085760223	301.2456423	0.109543666	82.77163117
0.002944483	0.350527017	0	1.713718644	6.298087843	0.064775353	0	1.652685136	75.1631163
0.002944483	0.350527017	0	1.713718644	6.298087843	0.064775353	0	1.652685136	75.1631163
39.63717417	77.93303846	0.003041902	0.890247112	0.357375193	42.90560519	0.752499175	0.944722788	0
59508.89074	21551.58748	4301.118895	11.27391116	195972.9386	246.3375064	0	1.387957088	0.010741314
0.006830418	1.291992672	0.001294496	4.568261285	3.191565412	0.795624527	0.214944542	1.177884063	0.190483938
0	0.057759406	0.11188111	0.18521563	5.42E-06	0.003848417	0.083913861	8.158583921	97.25208044
0.202660752	0.267587504	26.53112047	0.355134453	0.687651412	3.95060498	0.093089477	3.908049116	1.600701297
59508.89074	21551.58748	4301.118895	11.27391116	195972.9386	246.3375064	0	1.387957088	0.010741314
0.3868333	2.244195808	4.4847836	0.915005983	0.008291391	0.65022329	38.46864448	0.219898314	0.013367053
0.002944483	0.350527017	0	1.713718644	6.298087843	0.064775353	0	1.652685136	75.1631163
0.202660752	0.267587504	26.53112047	0.355134453	0.687651412	3.95060498	0.093089477	3.908049116	1.600701297
4.86638069	115.2345147	0.094371895	1.043598804	27.06276051	0.085760223	301.2456423	0.109543666	82.77163117
4.86638069	115.2345147	0.094371895	1.043598804	27.06276051	0.085760223	301.2456423	0.109543666	82.77163117
31.55519048	0.037055819	43.22421264	0.604429302	12.12653733	0.073629724	0	0.562789221	2.345622231
59508.89074	21551.58748	4301.118895	11.27391116	195972.9386	246.3375064	0	1.387957088	0.010741314
0.06621524	10.76762568	5.922218781	0.004727466	59.13728961	21.28584628	9.648578112	2.394832096	228.8283998
0.3868333	2.244195808	4.4847836	0.915005983	0.008291391	0.65022329	38.46864448	0.219898314	0.013367053
28.49628011	0.991344866	0.056525884	7.603402523	0.000690954	1.809089301	0.126285588	0.487990346	0.004370079
0.002944483	0.350527017	0	1.713718644	6.298087843	0.064775353	0	1.652685136	75.1631163
7.53E-05	0.660942902	0	0.065900288	0.564872126	0.149430295	0	1.004142332	0.006487485
0.041776317	60.62310832	1.039558825	1.234503664	47.21986769	0.033034676	0.453684698	0.220871964	154.4573253
31.55519048	0.037055819	43.22421264	0.604429302	12.12653733	0.073629724	0	0.562789221	2.345622231
4.86638069	115.2345147	0.094371895	1.043598804	27.06276051	0.085760223	301.2456423	0.109543666	82.77163117
31.55519048	0.037055819	43.22421264	0.604429302	12.12653733	0.073629724	0	0.562789221	2.345622231
39.63717417	77.93303846	0.003041902	0.890247112	0.357375193	42.90560519	0.752499175	0.944722788	0
31.55519048	0.037055819	43.22421264	0.604429302	12.12653733	0.073629724	0	0.562789221	2.345622231
31.55519048	0.037055819	43.22421264	0.604429302	12.12653733	0.073629724	0	0.562789221	2.345622231
455.7871995	1.073543725	0.032222759	3.003501052	170.0095678	0.263363077	1.510223325	4.689826844	0
455.7871995	1.073543725	0.032222759	3.003501052	170.0095678	0.263363077	1.510223325	4.689826844	0
31.55519048	0.037055819	43.22421264	0.604429302	12.12653733	0.073629724	0	0.562789221	2.345622231
0.482199379	0.004768307	0.02096569	0.782719448	0.048339941	4.304443401	0	0.201595404	2.005680679
21.22755434	0.362004239	0.458625717	60.87982682	84.45411124	0.260792482	0	0.200468657	0.028570969
59508.89074	21551.58748	4301.118895	11.27391116	195972.9386	246.3375064	0	1.387957088	0.010741314
0.006830418	1.291992672	0.001294496	4.568261285	3.191565412	0.795624527	0.214944542	1.177884063	0.190483938
31.55519048	0.037055819	43.22421264	0.604429302	12.12653733	0.073629724	0	0.562789221	2.345622231
166.2297343	0.027677477	170.570441	58.34376362	69.99236234	187.5554183	0	0.617498982	0.005107595
21.22755434	0.362004239	0.458625717	60.87982682	84.45411124	0.260792482	0	0.200468657	0.028570969
0.002944483	0.350527017	0	1.713718644	6.298087843	0.064775353	0	1.652685136	75.1631163
1.551536401	3.519831878	0.001530975	5.325409069	9.584778975	4.470502157	0.365076679	4.016569328	0.330986488
455.7871995	1.073543725	0.032222759	3.003501052	170.0095678	0.263363077	1.510223325	4.689826844	0

13.89972963	0.014078114	217.5251456	0.140033911	0.000468885	162.4022311	0	16.60279417	1255.161428
13.89972963	0.014078114	217.5251456	0.140033911	0.000468885	162.4022311	0	16.60279417	1255.161428
39.63717417	77.93303846	0.003041902	0.890247112	0.357375193	42.90560519	0.752499175	0.944722788	0
28.49628011	0.991344866	0.056525884	7.603402523	0.000690954	1.809089301	0.126285588	0.487990346	0.004370079
166.2297343	0.027677477	170.570441	58.34376362	69.99236234	187.5554183	0	0.617498982	0.005107595
455.7871995	1.073543725	0.032222759	3.003501052	170.0095678	0.263363077	1.510223325	4.689826844	0
166.2297343	0.027677477	170.570441	58.34376362	69.99236234	187.5554183	0	0.617498982	0.005107595
0.202660752	0.267587504	26.53112047	0.355134453	0.687651412	3.95060498	0.093089477	3.908049116	1.600701297
455.7871995	1.073543725	0.032222759	3.003501052	170.0095678	0.263363077	1.510223325	4.689826844	0
0.006830418	1.291992672	0.001294496	4.568261285	3.191565412	0.795624527	0.214944542	1.177884063	0.190483938
0.06621524	10.76762568	5.922218781	0.004727466	59.13728961	21.28584628	9.648578112	2.394832096	228.8283998
0.006830418	1.291992672	0.001294496	4.568261285	3.191565412	0.795624527	0.214944542	1.177884063	0.190483938
0.000277632	0.140479681	12.80283743	0.035237556	1.01902267	0.003195477	0.881575129	1.258687003	0
0.041776317	60.62310832	1.039558825	1.234503664	47.21986769	0.033034676	0.453684698	0.220871964	154.4573253
7.53E-05	0.660942902	0	0.065900288	0.564872126	0.149430295	0	1.004142332	0.006487485
21.22755434	0.362004239	0.458625717	60.87982682	84.45411124	0.260792482	0	0.200468657	0.028570969
39.63717417	77.93303846	0.003041902	0.890247112	0.357375193	42.90560519	0.752499175	0.944722788	0
1.551536401	3.519831878	0.001530975	5.325409069	9.584778975	4.470502157	0.365076679	4.016569328	0.330986488
0.202660752	0.267587504	26.53112047	0.355134453	0.687651412	3.95060498	0.093089477	3.908049116	1.600701297
0.202660752	0.267587504	26.53112047	0.355134453	0.687651412	3.95060498	0.093089477	3.908049116	1.600701297
31.55519048	0.037055819	43.22421264	0.604429302	12.12653733	0.073629724	0	0.562789221	2.345622231
21.22755434	0.362004239	0.458625717	60.87982682	84.45411124	0.260792482	0	0.200468657	0.028570969
0.041776317	60.62310832	1.039558825	1.234503664	47.21986769	0.033034676	0.453684698	0.220871964	154.4573253
0.482199379	0.004768307	0.02096569	0.782719448	0.048339941	4.304443401	0	0.201595404	2.005680679
7.53E-05	0.660942902	0	0.065900288	0.564872126	0.149430295	0	1.004142332	0.006487485
0.006830418	1.291992672	0.001294496	4.568261285	3.191565412	0.795624527	0.214944542	1.177884063	0.190483938
0.3868333	2.244195808	4.4847836	0.915005983	0.008291391	0.65022329	38.46864448	0.219898314	0.013367053
13.89972963	0.014078114	217.5251456	0.140033911	0.000468885	162.4022311	0	16.60279417	1255.161428
28.49628011	0.991344866	0.056525884	7.603402523	0.000690954	1.809089301	0.126285588	0.487990346	0.004370079
0	0.057759406	0.11188111	0.18521563	5.42E-06	0.003848417	0.083913861	8.158583921	97.25208044
0.000277632	0.140479681	12.80283743	0.035237556	1.01902267	0.003195477	0.881575129	1.258687003	0
0.202660752	0.267587504	26.53112047	0.355134453	0.687651412	3.95060498	0.093089477	3.908049116	1.600701297
28.49628011	0.991344866	0.056525884	7.603402523	0.000690954	1.809089301	0.126285588	0.487990346	0.004370079
0.041776317	60.62310832	1.039558825	1.234503664	47.21986769	0.033034676	0.453684698	0.220871964	154.4573253
39.63717417	77.93303846	0.003041902	0.890247112	0.357375193	42.90560519	0.752499175	0.944722788	0
4.86638069	115.2345147	0.094371895	1.043598804	27.06276051	0.085760223	301.2456423	0.109543666	82.77163117
0.202660752	0.267587504	26.53112047	0.355134453	0.687651412	3.95060498	0.093089477	3.908049116	1.600701297
166.2297343	0.027677477	170.570441	58.34376362	69.99236234	187.5554183	0	0.617498982	0.005107595
59508.89074	21551.58748	4301.118895	11.27391116	195972.9386	246.3375064	0	1.387957088	0.010741314
13.89972963	0.014078114	217.5251456	0.140033911	0.000468885	162.4022311	0	16.60279417	1255.161428
7.53E-05	0.660942902	0	0.065900288	0.564872126	0.149430295	0	1.004142332	0.006487485

7.53E-05	0.660942902	0	0.065900288	0.564872126	0.149430295	0	1.004142332	0.006487485
0.002944483	0.350527017	0	1.713718644	6.298087843	0.064775353	0	1.652685136	75.1631163
166.2297343	0.027677477	170.570441	58.34376362	69.99236234	187.5554183	0	0.617498982	0.005107595
21.22755434	0.362004239	0.458625717	60.87982682	84.45411124	0.260792482	0	0.200468657	0.028570969
4.86638069	115.2345147	0.094371895	1.043598804	27.06276051	0.085760223	301.2456423	0.109543666	82.77163117
21.22755434	0.362004239	0.458625717	60.87982682	84.45411124	0.260792482	0	0.200468657	0.028570969
0	0.057759406	0.11188111	0.18521563	5.42E-06	0.003848417	0.083913861	8.158583921	97.25208044
31.55519048	0.037055819	43.22421264	0.604429302	12.12653733	0.073629724	0	0.562789221	2.345622231
59508.89074	21551.58748	4301.118895	11.27391116	195972.9386	246.3375064	0	1.387957088	0.010741314
59508.89074	21551.58748	4301.118895	11.27391116	195972.9386	246.3375064	0	1.387957088	0.010741314
4.86638069	115.2345147	0.094371895	1.043598804	27.06276051	0.085760223	301.2456423	0.109543666	82.77163117
1.551536401	3.519831878	0.001530975	5.325409069	9.584778975	4.470502157	0.365076679	4.016569328	0.330986488
39.63717417	77.93303846	0.003041902	0.890247112	0.357375193	42.90560519	0.752499175	0.944722788	0
7.53E-05	0.660942902	0	0.065900288	0.564872126	0.149430295	0	1.004142332	0.006487485
0.067078768	0.06197952	0.858165504	0.583227345	0.000315376	0.276784403	0.041675687	0.210912513	0.003810947
0.067078768	0.06197952	0.858165504	0.583227345	0.000315376	0.276784403	0.041675687	0.210912513	0.003810947
0.482199379	0.004768307	0.02096569	0.782719448	0.048339941	4.304443401	0	0.201595404	2.005680679
0.202660752	0.267587504	26.53112047	0.355134453	0.687651412	3.95060498	0.093089477	3.908049116	1.600701297
0.482199379	0.004768307	0.02096569	0.782719448	0.048339941	4.304443401	0	0.201595404	2.005680679
21.22755434	0.362004239	0.458625717	60.87982682	84.45411124	0.260792482	0	0.200468657	0.028570969
39.63717417	77.93303846	0.003041902	0.890247112	0.357375193	42.90560519	0.752499175	0.944722788	0
0.3868333	2.244195808	4.4847836	0.915005983	0.008291391	0.65022329	38.46864448	0.219898314	0.013367053
166.2297343	0.027677477	170.570441	58.34376362	69.99236234	187.5554183	0	0.617498982	0.005107595
59508.89074	21551.58748	4301.118895	11.27391116	195972.9386	246.3375064	0	1.387957088	0.010741314
0.000277632	0.140479681	12.80283743	0.035237556	1.01902267	0.003195477	0.881575129	1.258687003	0
0.202660752	0.267587504	26.53112047	0.355134453	0.687651412	3.95060498	0.093089477	3.908049116	1.600701297
0.002944483	0.350527017	0	1.713718644	6.298087843	0.064775353	0	1.652685136	75.1631163
31.55519048	0.037055819	43.22421264	0.604429302	12.12653733	0.073629724	0	0.562789221	2.345622231
1.551536401	3.519831878	0.001530975	5.325409069	9.584778975	4.470502157	0.365076679	4.016569328	0.330986488
28.49628011	0.991344866	0.056525884	7.603402523	0.000690954	1.809089301	0.126285588	0.487990346	0.004370079
0.482199379	0.004768307	0.02096569	0.782719448	0.048339941	4.304443401	0	0.201595404	2.005680679
166.2297343	0.027677477	170.570441	58.34376362	69.99236234	187.5554183	0	0.617498982	0.005107595
7.53E-05	0.660942902	0	0.065900288	0.564872126	0.149430295	0	1.004142332	0.006487485
0.482199379	0.004768307	0.02096569	0.782719448	0.048339941	4.304443401	0	0.201595404	2.005680679
0.06621524	10.76762568	5.922218781	0.004727466	59.13728961	21.28584628	9.648578112	2.394832096	228.8283998
13.89972963	0.014078114	217.5251456	0.140033911	0.000468885	162.4022311	0	16.60279417	1255.161428
0.006830418	1.291992672	0.001294496	4.568261285	3.191565412	0.795624527	0.214944542	1.177884063	0.190483938
4.86638069	115.2345147	0.094371895	1.043598804	27.06276051	0.085760223	301.2456423	0.109543666	82.77163117
0.202660752	0.267587504	26.53112047	0.355134453	0.687651412	3.95060498	0.093089477	3.908049116	1.600701297
21.22755434	0.362004239	0.458625717	60.87982682	84.45411124	0.260792482	0	0.200468657	0.028570969
0.3868333	2.244195808	4.4847836	0.915005983	0.008291391	0.65022329	38.46864448	0.219898314	0.013367053

21.22755434	0.362004239	0.458625717	60.87982682	84.45411124	0.260792482	0	0.200468657	0.028570969
0.000277632	0.140479681	12.80283743	0.035237556	1.01902267	0.003195477	0.881575129	1.258687003	0
0.041776317	60.62310832	1.039558825	1.234503664	47.21986769	0.033034676	0.453684698	0.220871964	154.4573253
166.2297343	0.027677477	170.570441	58.34376362	69.99236234	187.5554183	0	0.617498982	0.005107595
1.551536401	3.519831878	0.001530975	5.325409069	9.584778975	4.470502157	0.365076679	4.016569328	0.330986488
0.002944483	0.350527017	0	1.713718644	6.298087843	0.064775353	0	1.652685136	75.1631163
0.041776317	60.62310832	1.039558825	1.234503664	47.21986769	0.033034676	0.453684698	0.220871964	154.4573253
0	0.057759406	0.11188111	0.18521563	5.42E-06	0.003848417	0.083913861	8.158583921	97.25208044
13.89972963	0.014078114	217.5251456	0.140033911	0.000468885	162.4022311	0	16.60279417	1255.161428
1.551536401	3.519831878	0.001530975	5.325409069	9.584778975	4.470502157	0.365076679	4.016569328	0.330986488
455.7871995	1.073543725	0.032222759	3.003501052	170.0095678	0.263363077	1.510223325	4.689826844	0
59508.89074	21551.58748	4301.118895	11.27391116	195972.9386	246.3375064	0	1.387957088	0.010741314
0.202660752	0.267587504	26.53112047	0.355134453	0.687651412	3.95060498	0.093089477	3.908049116	1.600701297
13.89972963	0.014078114	217.5251456	0.140033911	0.000468885	162.4022311	0	16.60279417	1255.161428
0.067078768	0.06197952	0.858165504	0.583227345	0.000315376	0.276784403	0.041675687	0.210912513	0.003810947
0.006830418	1.291992672	0.001294496	4.568261285	3.191565412	0.795624527	0.214944542	1.177884063	0.190483938
0.002944483	0.350527017	0	1.713718644	6.298087843	0.064775353	0	1.652685136	75.1631163
4.86638069	115.2345147	0.094371895	1.043598804	27.06276051	0.085760223	301.2456423	0.109543666	82.77163117
31.55519048	0.037055819	43.22421264	0.604429302	12.12653733	0.073629724	0	0.562789221	2.345622231
1.551536401	3.519831878	0.001530975	5.325409069	9.584778975	4.470502157	0.365076679	4.016569328	0.330986488
39.63717417	77.93303846	0.003041902	0.890247112	0.357375193	42.90560519	0.752499175	0.944722788	0
0.06621524	10.76762568	5.922218781	0.004727466	59.13728961	21.28584628	9.648578112	2.394832096	228.8283998
21.22755434	0.362004239	0.458625717	60.87982682	84.45411124	0.260792482	0	0.200468657	0.028570969
0	0.057759406	0.11188111	0.18521563	5.42E-06	0.003848417	0.083913861	8.158583921	97.25208044
0.006830418	1.291992672	0.001294496	4.568261285	3.191565412	0.795624527	0.214944542	1.177884063	0.190483938
4.86638069	115.2345147	0.094371895	1.043598804	27.06276051	0.085760223	301.2456423	0.109543666	82.77163117
4.86638069	115.2345147	0.094371895	1.043598804	27.06276051	0.085760223	301.2456423	0.109543666	82.77163117
0.482199379	0.004768307	0.02096569	0.782719448	0.048339941	4.304443401	0	0.201595404	2.005680679
7.53E-05	0.660942902	0	0.065900288	0.564872126	0.149430295	0	1.004142332	0.006487485
0.202660752	0.267587504	26.53112047	0.355134453	0.687651412	3.95060498	0.093089477	3.908049116	1.600701297
0.3868333	2.244195808	4.4847836	0.915005983	0.008291391	0.65022329	38.46864448	0.219898314	0.013367053
0.000277632	0.140479681	12.80283743	0.035237556	1.01902267	0.003195477	0.881575129	1.258687003	0
0.482199379	0.004768307	0.02096569	0.782719448	0.048339941	4.304443401	0	0.201595404	2.005680679
166.2297343	0.027677477	170.570441	58.34376362	69.99236234	187.5554183	0	0.617498982	0.005107595
455.7871995	1.073543725	0.032222759	3.003501052	170.0095678	0.263363077	1.510223325	4.689826844	0
0.000277632	0.140479681	12.80283743	0.035237556	1.01902267	0.003195477	0.881575129	1.258687003	0
0.067078768	0.06197952	0.858165504	0.583227345	0.000315376	0.276784403	0.041675687	0.210912513	0.003810947
13.89972963	0.014078114	217.5251456	0.140033911	0.000468885	162.4022311	0	16.60279417	1255.161428
0.067078768	0.06197952	0.858165504	0.583227345	0.000315376	0.276784403	0.041675687	0.210912513	0.003810947
0.006830418	1.291992672	0.001294496	4.568261285	3.191565412	0.795624527	0.214944542	1.177884063	0.190483938
1.551536401	3.519831878	0.001530975	5.325409069	9.584778975	4.470502157	0.365076679	4.016569328	0.330986488

0.041776317	60.62310832	1.039558825	1.234503664	47.21986769	0.033034676	0.453684698	0.220871964	154.4573253
13.89972963	0.014078114	217.5251456	0.140033911	0.000468885	162.4022311	0	16.60279417	1255.161428
13.89972963	0.014078114	217.5251456	0.140033911	0.000468885	162.4022311	0	16.60279417	1255.161428
39.63717417	77.93303846	0.003041902	0.890247112	0.357375193	42.90560519	0.752499175	0.944722788	0
0.002944483	0.350527017	0	1.713718644	6.298087843	0.064775353	0	1.652685136	75.1631163
0.000277632	0.140479681	12.80283743	0.035237556	1.01902267	0.003195477	0.881575129	1.258687003	0
7.53E-05	0.660942902	0	0.065900288	0.564872126	0.149430295	0	1.004142332	0.006487485
166.2297343	0.027677477	170.570441	58.34376362	69.99236234	187.5554183	0	0.617498982	0.005107595
0.041776317	60.62310832	1.039558825	1.234503664	47.21986769	0.033034676	0.453684698	0.220871964	154.4573253
166.2297343	0.027677477	170.570441	58.34376362	69.99236234	187.5554183	0	0.617498982	0.005107595
0.06621524	10.76762568	5.922218781	0.004727466	59.13728961	21.28584628	9.648578112	2.394832096	228.8283998
13.89972963	0.014078114	217.5251456	0.140033911	0.000468885	162.4022311	0	16.60279417	1255.161428
1.551536401	3.519831878	0.001530975	5.325409069	9.584778975	4.470502157	0.365076679	4.016569328	0.330986488
0.3868333	2.244195808	4.4847836	0.915005983	0.008291391	0.65022329	38.46864448	0.219898314	0.013367053
0.000277632	0.140479681	12.80283743	0.035237556	1.01902267	0.003195477	0.881575129	1.258687003	0
21.22755434	0.362004239	0.458625717	60.87982682	84.45411124	0.260792482	0	0.200468657	0.028570969
21.22755434	0.362004239	0.458625717	60.87982682	84.45411124	0.260792482	0	0.200468657	0.028570969
0.482199379	0.004768307	0.02096569	0.782719448	0.048339941	4.304443401	0	0.201595404	2.005680679
4.86638069	115.2345147	0.094371895	1.043598804	27.06276051	0.085760223	301.2456423	0.109543666	82.77163117
455.7871995	1.073543725	0.032222759	3.003501052	170.0095678	0.263363077	1.510223325	4.689826844	0
28.49628011	0.991344866	0.056525884	7.603402523	0.000690954	1.809089301	0.126285588	0.487990346	0.004370079
31.55519048	0.037055819	43.22421264	0.604429302	12.12653733	0.073629724	0	0.562789221	2.345622231
7.53E-05	0.660942902	0	0.065900288	0.564872126	0.149430295	0	1.004142332	0.006487485
1.551536401	3.519831878	0.001530975	5.325409069	9.584778975	4.470502157	0.365076679	4.016569328	0.330986488
31.55519048	0.037055819	43.22421264	0.604429302	12.12653733	0.073629724	0	0.562789221	2.345622231
21.22755434	0.362004239	0.458625717	60.87982682	84.45411124	0.260792482	0	0.200468657	0.028570969
28.49628011	0.991344866	0.056525884	7.603402523	0.000690954	1.809089301	0.126285588	0.487990346	0.004370079
4.86638069	115.2345147	0.094371895	1.043598804	27.06276051	0.085760223	301.2456423	0.109543666	82.77163117
59508.89074	21551.58748	4301.118895	11.27391116	195972.9386	246.3375064	0	1.387957088	0.010741314
0.002944483	0.350527017	0	1.713718644	6.298087843	0.064775353	0	1.652685136	75.1631163
0.000277632	0.140479681	12.80283743	0.035237556	1.01902267	0.003195477	0.881575129	1.258687003	0
0.06621524	10.76762568	5.922218781	0.004727466	59.13728961	21.28584628	9.648578112	2.394832096	228.8283998
1.551536401	3.519831878	0.001530975	5.325409069	9.584778975	4.470502157	0.365076679	4.016569328	0.330986488
59508.89074	21551.58748	4301.118895	11.27391116	195972.9386	246.3375064	0	1.387957088	0.010741314
13.89972963	0.014078114	217.5251456	0.140033911	0.000468885	162.4022311	0	16.60279417	1255.161428
28.49628011	0.991344866	0.056525884	7.603402523	0.000690954	1.809089301	0.126285588	0.487990346	0.004370079
166.2297343	0.027677477	170.570441	58.34376362	69.99236234	187.5554183	0	0.617498982	0.005107595
7.53E-05	0.660942902	0	0.065900288	0.564872126	0.149430295	0	1.004142332	0.006487485
4.86638069	115.2345147	0.094371895	1.043598804	27.06276051	0.085760223	301.2456423	0.109543666	82.77163117
0.3868333	2.244195808	4.4847836	0.915005983	0.008291391	0.65022329	38.46864448	0.219898314	0.013367053
0	0.057759406	0.11188111	0.18521563	5.42E-06	0.003848417	0.083913861	8.158583921	97.25208044

0.041776317	60.62310832	1.039558825	1.234503664	47.21986769	0.033034676	0.453684698	0.220871964	154.4573253
1.551536401	3.519831878	0.001530975	5.325409069	9.584778975	4.470502157	0.365076679	4.016569328	0.330986488
0.482199379	0.004768307	0.02096569	0.782719448	0.048339941	4.304443401	0	0.201595404	2.005680679
13.89972963	0.014078114	217.5251456	0.140033911	0.000468885	162.4022311	0	16.60279417	1255.161428
0.006830418	1.291992672	0.001294496	4.568261285	3.191565412	0.795624527	0.214944542	1.177884063	0.190483938
1.551536401	3.519831878	0.001530975	5.325409069	9.584778975	4.470502157	0.365076679	4.016569328	0.330986488
4.86638069	115.2345147	0.094371895	1.043598804	27.06276051	0.085760223	301.2456423	0.109543666	82.77163117
28.49628011	0.991344866	0.056525884	7.603402523	0.000690954	1.809089301	0.126285588	0.487990346	0.004370079
13.89972963	0.014078114	217.5251456	0.140033911	0.000468885	162.4022311	0	16.60279417	1255.161428
0.482199379	0.004768307	0.02096569	0.782719448	0.048339941	4.304443401	0	0.201595404	2.005680679
0.067078768	0.06197952	0.858165504	0.583227345	0.000315376	0.276784403	0.041675687	0.210912513	0.003810947
59508.89074	21551.58748	4301.118895	11.27391116	195972.9386	246.3375064	0	1.387957088	0.010741314
455.7871995	1.073543725	0.032222759	3.003501052	170.0095678	0.263363077	1.510223325	4.689826844	0
0.002944483	0.350527017	0	1.713718644	6.298087843	0.064775353	0	1.652685136	75.1631163
31.55519048	0.037055819	43.22421264	0.604429302	12.12653733	0.073629724	0	0.562789221	2.345622231
0.202660752	0.267587504	26.53112047	0.355134453	0.687651412	3.95060498	0.093089477	3.908049116	1.600701297
28.49628011	0.991344866	0.056525884	7.603402523	0.000690954	1.809089301	0.126285588	0.487990346	0.004370079
0.002944483	0.350527017	0	1.713718644	6.298087843	0.064775353	0	1.652685136	75.1631163
0.041776317	60.62310832	1.039558825	1.234503664	47.21986769	0.033034676	0.453684698	0.220871964	154.4573253
0.482199379	0.004768307	0.02096569	0.782719448	0.048339941	4.304443401	0	0.201595404	2.005680679
59508.89074	21551.58748	4301.118895	11.27391116	195972.9386	246.3375064	0	1.387957088	0.010741314
166.2297343	0.027677477	170.570441	58.34376362	69.99236234	187.5554183	0	0.617498982	0.005107595
0.000277632	0.140479681	12.80283743	0.035237556	1.01902267	0.003195477	0.881575129	1.258687003	0
39.63717417	77.93303846	0.003041902	0.890247112	0.357375193	42.90560519	0.752499175	0.944722788	0
39.63717417	77.93303846	0.003041902	0.890247112	0.357375193	42.90560519	0.752499175	0.944722788	0
166.2297343	0.027677477	170.570441	58.34376362	69.99236234	187.5554183	0	0.617498982	0.005107595
0.06621524	10.76762568	5.922218781	0.004727466	59.13728961	21.28584628	9.648578112	2.394832096	228.8283998
1.551536401	3.519831878	0.001530975	5.325409069	9.584778975	4.470502157	0.365076679	4.016569328	0.330986488
0.482199379	0.004768307	0.02096569	0.782719448	0.048339941	4.304443401	0	0.201595404	2.005680679
0	0.057759406	0.11188111	0.18521563	5.42E-06	0.003848417	0.083913861	8.158583921	97.25208044
0.041776317	60.62310832	1.039558825	1.234503664	47.21986769	0.033034676	0.453684698	0.220871964	154.4573253
28.49628011	0.991344866	0.056525884	7.603402523	0.000690954	1.809089301	0.126285588	0.487990346	0.004370079
4.86638069	115.2345147	0.094371895	1.043598804	27.06276051	0.085760223	301.2456423	0.109543666	82.77163117
455.7871995	1.073543725	0.032222759	3.003501052	170.0095678	0.263363077	1.510223325	4.689826844	0
59508.89074	21551.58748	4301.118895	11.27391116	195972.9386	246.3375064	0	1.387957088	0.010741314
7.53E-05	0.660942902	0	0.065900288	0.564872126	0.149430295	0	1.004142332	0.006487485
0.3868333	2.244195808	4.4847836	0.915005983	0.008291391	0.65022329	38.46864448	0.219898314	0.013367053
0.067078768	0.06197952	0.858165504	0.583227345	0.000315376	0.276784403	0.041675687	0.210912513	0.003810947
455.7871995	1.073543725	0.032222759	3.003501052	170.0095678	0.263363077	1.510223325	4.689826844	0
13.89972963	0.014078114	217.5251456	0.140033911	0.000468885	162.4022311	0	16.60279417	1255.161428
455.7871995	1.073543725	0.032222759	3.003501052	170.0095678	0.263363077	1.510223325	4.689826844	0

28.49628011	0.991344866	0.056525884	7.603402523	0.000690954	1.809089301	0.126285588	0.487990346	0.004370079
31.55519048	0.037055819	43.22421264	0.604429302	12.12653733	0.073629724	0	0.562789221	2.345622231
166.2297343	0.027677477	170.570441	58.34376362	69.99236234	187.5554183	0	0.617498982	0.005107595
4.86638069	115.2345147	0.094371895	1.043598804	27.06276051	0.085760223	301.2456423	0.109543666	82.77163117
0.006830418	1.291992672	0.001294496	4.568261285	3.191565412	0.795624527	0.214944542	1.177884063	0.190483938
0	0.057759406	0.11188111	0.18521563	5.42E-06	0.003848417	0.083913861	8.158583921	97.25208044
39.63717417	77.93303846	0.003041902	0.890247112	0.357375193	42.90560519	0.752499175	0.944722788	0
0.002944483	0.350527017	0	1.713718644	6.298087843	0.064775353	0	1.652685136	75.1631163
59508.89074	21551.58748	4301.118895	11.27391116	195972.9386	246.3375064	0	1.387957088	0.010741314
1.551536401	3.519831878	0.001530975	5.325409069	9.584778975	4.470502157	0.365076679	4.016569328	0.330986488
0.482199379	0.004768307	0.02096569	0.782719448	0.048339941	4.304443401	0	0.201595404	2.005680679
13.89972963	0.014078114	217.5251456	0.140033911	0.000468885	162.4022311	0	16.60279417	1255.161428
59508.89074	21551.58748	4301.118895	11.27391116	195972.9386	246.3375064	0	1.387957088	0.010741314
13.89972963	0.014078114	217.5251456	0.140033911	0.000468885	162.4022311	0	16.60279417	1255.161428
13.89972963	0.014078114	217.5251456	0.140033911	0.000468885	162.4022311	0	16.60279417	1255.161428
0.202660752	0.267587504	26.53112047	0.355134453	0.687651412	3.95060498	0.093089477	3.908049116	1.600701297
7.53E-05	0.660942902	0	0.065900288	0.564872126	0.149430295	0	1.004142332	0.006487485
59508.89074	21551.58748	4301.118895	11.27391116	195972.9386	246.3375064	0	1.387957088	0.010741314
455.7871995	1.073543725	0.032222759	3.003501052	170.0095678	0.263363077	1.510223325	4.689826844	0
0.067078768	0.06197952	0.858165504	0.583227345	0.000315376	0.276784403	0.041675687	0.210912513	0.003810947
13.89972963	0.014078114	217.5251456	0.140033911	0.000468885	162.4022311	0	16.60279417	1255.161428
21.22755434	0.362004239	0.458625717	60.87982682	84.45411124	0.260792482	0	0.200468657	0.028570969
39.63717417	77.93303846	0.003041902	0.890247112	0.357375193	42.90560519	0.752499175	0.944722788	0
0.202660752	0.267587504	26.53112047	0.355134453	0.687651412	3.95060498	0.093089477	3.908049116	1.600701297
0.06621524	10.76762568	5.922218781	0.004727466	59.13728961	21.28584628	9.648578112	2.394832096	228.8283998
0.482199379	0.004768307	0.02096569	0.782719448	0.048339941	4.304443401	0	0.201595404	2.005680679
1.551536401	3.519831878	0.001530975	5.325409069	9.584778975	4.470502157	0.365076679	4.016569328	0.330986488
0.202660752	0.267587504	26.53112047	0.355134453	0.687651412	3.95060498	0.093089477	3.908049116	1.600701297
7.53E-05	0.660942902	0	0.065900288	0.564872126	0.149430295	0	1.004142332	0.006487485
31.55519048	0.037055819	43.22421264	0.604429302	12.12653733	0.073629724	0	0.562789221	2.345622231
166.2297343	0.027677477	170.570441	58.34376362	69.99236234	187.5554183	0	0.617498982	0.005107595
31.55519048	0.037055819	43.22421264	0.604429302	12.12653733	0.073629724	0	0.562789221	2.345622231
21.22755434	0.362004239	0.458625717	60.87982682	84.45411124	0.260792482	0	0.200468657	0.028570969
0.482199379	0.004768307	0.02096569	0.782719448	0.048339941	4.304443401	0	0.201595404	2.005680679
0.3868333	2.244195808	4.4847836	0.915005983	0.008291391	0.65022329	38.46864448	0.219898314	0.013367053
0.202660752	0.267587504	26.53112047	0.355134453	0.687651412	3.95060498	0.093089477	3.908049116	1.600701297
0.202660752	0.267587504	26.53112047	0.355134453	0.687651412	3.95060498	0.093089477	3.908049116	1.600701297
166.2297343	0.027677477	170.570441	58.34376362	69.99236234	187.5554183	0	0.617498982	0.005107595
0.3868333	2.244195808	4.4847836	0.915005983	0.008291391	0.65022329	38.46864448	0.219898314	0.013367053
0.006830418	1.291992672	0.001294496	4.568261285	3.191565412	0.795624527	0.214944542	1.177884063	0.190483938
4.86638069	115.2345147	0.094371895	1.043598804	27.06276051	0.085760223	301.2456423	0.109543666	82.77163117

0.041776317	60.62310832	1.039558825	1.234503664	47.21986769	0.033034676	0.453684698	0.220871964	154.4573253
0.3868333	2.244195808	4.4847836	0.915005983	0.008291391	0.65022329	38.46864448	0.219898314	0.013367053
31.55519048	0.037055819	43.22421264	0.604429302	12.12653733	0.073629724	0	0.562789221	2.345622231
0.067078768	0.06197952	0.858165504	0.583227345	0.000315376	0.276784403	0.041675687	0.210912513	0.003810947
39.63717417	77.93303846	0.003041902	0.890247112	0.357375193	42.90560519	0.752499175	0.944722788	0
0.482199379	0.004768307	0.02096569	0.782719448	0.048339941	4.304443401	0	0.201595404	2.005680679
455.7871995	1.073543725	0.032222759	3.003501052	170.0095678	0.263363077	1.510223325	4.689826844	0
0	0.057759406	0.11188111	0.18521563	5.42E-06	0.003848417	0.083913861	8.158583921	97.25208044
21.22755434	0.362004239	0.458625717	60.87982682	84.45411124	0.260792482	0	0.200468657	0.028570969
166.2297343	0.027677477	170.570441	58.34376362	69.99236234	187.5554183	0	0.617498982	0.005107595
0.482199379	0.004768307	0.02096569	0.782719448	0.048339941	4.304443401	0	0.201595404	2.005680679
4.86638069	115.2345147	0.094371895	1.043598804	27.06276051	0.085760223	301.2456423	0.109543666	82.77163117
4.86638069	115.2345147	0.094371895	1.043598804	27.06276051	0.085760223	301.2456423	0.109543666	82.77163117
31.55519048	0.037055819	43.22421264	0.604429302	12.12653733	0.073629724	0	0.562789221	2.345622231
455.7871995	1.073543725	0.032222759	3.003501052	170.0095678	0.263363077	1.510223325	4.689826844	0
0.06621524	10.76762568	5.922218781	0.004727466	59.13728961	21.28584628	9.648578112	2.394832096	228.8283998
0.482199379	0.004768307	0.02096569	0.782719448	0.048339941	4.304443401	0	0.201595404	2.005680679
21.22755434	0.362004239	0.458625717	60.87982682	84.45411124	0.260792482	0	0.200468657	0.028570969
0	0.057759406	0.11188111	0.18521563	5.42E-06	0.003848417	0.083913861	8.158583921	97.25208044
0.3868333	2.244195808	4.4847836	0.915005983	0.008291391	0.65022329	38.46864448	0.219898314	0.013367053
21.22755434	0.362004239	0.458625717	60.87982682	84.45411124	0.260792482	0	0.200468657	0.028570969
0.482199379	0.004768307	0.02096569	0.782719448	0.048339941	4.304443401	0	0.201595404	2.005680679
0.006830418	1.291992672	0.001294496	4.568261285	3.191565412	0.795624527	0.214944542	1.177884063	0.190483938
0.006830418	1.291992672	0.001294496	4.568261285	3.191565412	0.795624527	0.214944542	1.177884063	0.190483938
7.53E-05	0.660942902	0	0.065900288	0.564872126	0.149430295	0	1.004142332	0.006487485
21.22755434	0.362004239	0.458625717	60.87982682	84.45411124	0.260792482	0	0.200468657	0.028570969
455.7871995	1.073543725	0.032222759	3.003501052	170.0095678	0.263363077	1.510223325	4.689826844	0
0.202660752	0.267587504	26.53112047	0.355134453	0.687651412	3.95060498	0.093089477	3.908049116	1.600701297
0.000277632	0.140479681	12.80283743	0.035237556	1.01902267	0.003195477	0.881575129	1.258687003	0
21.22755434	0.362004239	0.458625717	60.87982682	84.45411124	0.260792482	0	0.200468657	0.028570969
21.22755434	0.362004239	0.458625717	60.87982682	84.45411124	0.260792482	0	0.200468657	0.028570969
455.7871995	1.073543725	0.032222759	3.003501052	170.0095678	0.263363077	1.510223325	4.689826844	0
7.53E-05	0.660942902	0	0.065900288	0.564872126	0.149430295	0	1.004142332	0.006487485
455.7871995	1.073543725	0.032222759	3.003501052	170.0095678	0.263363077	1.510223325	4.689826844	0
0.482199379	0.004768307	0.02096569	0.782719448	0.048339941	4.304443401	0	0.201595404	2.005680679
0.06621524	10.76762568	5.922218781	0.004727466	59.13728961	21.28584628	9.648578112	2.394832096	228.8283998
0.202660752	0.267587504	26.53112047	0.355134453	0.687651412	3.95060498	0.093089477	3.908049116	1.600701297
0.000277632	0.140479681	12.80283743	0.035237556	1.01902267	0.003195477	0.881575129	1.258687003	0
1.551536401	3.519831878	0.001530975	5.325409069	9.584778975	4.470502157	0.365076679	4.016569328	0.330986488
166.2297343	0.027677477	170.570441	58.34376362	69.99236234	187.5554183	0	0.617498982	0.005107595
28.49628011	0.991344866	0.056525884	7.603402523	0.000690954	1.809089301	0.126285588	0.487990346	0.004370079

0.000277632	0.140479681	12.80283743	0.035237556	1.01902267	0.003195477	0.881575129	1.258687003	0
166.2297343	0.027677477	170.570441	58.34376362	69.99236234	187.5554183	0	0.617498982	0.005107595
0.000277632	0.140479681	12.80283743	0.035237556	1.01902267	0.003195477	0.881575129	1.258687003	0
39.63717417	77.93303846	0.003041902	0.890247112	0.357375193	42.90560519	0.752499175	0.944722788	0
39.63717417	77.93303846	0.003041902	0.890247112	0.357375193	42.90560519	0.752499175	0.944722788	0
1.551536401	3.519831878	0.001530975	5.325409069	9.584778975	4.470502157	0.365076679	4.016569328	0.330986488
7.53E-05	0.660942902	0	0.065900288	0.564872126	0.149430295	0	1.004142332	0.006487485
0	0.057759406	0.11188111	0.18521563	5.42E-06	0.003848417	0.083913861	8.158583921	97.25208044
28.49628011	0.991344866	0.056525884	7.603402523	0.000690954	1.809089301	0.126285588	0.487990346	0.004370079
7.53E-05	0.660942902	0	0.065900288	0.564872126	0.149430295	0	1.004142332	0.006487485
21.22755434	0.362004239	0.458625717	60.87982682	84.45411124	0.260792482	0	0.200468657	0.028570969
31.55519048	0.037055819	43.22421264	0.604429302	12.12653733	0.073629724	0	0.562789221	2.345622231
0.06621524	10.76762568	5.922218781	0.004727466	59.13728961	21.28584628	9.648578112	2.394832096	228.8283998
0.06621524	10.76762568	5.922218781	0.004727466	59.13728961	21.28584628	9.648578112	2.394832096	228.8283998
455.7871995	1.073543725	0.032222759	3.003501052	170.0095678	0.263363077	1.510223325	4.689826844	0
0.006830418	1.291992672	0.001294496	4.568261285	3.191565412	0.795624527	0.214944542	1.177884063	0.190483938
455.7871995	1.073543725	0.032222759	3.003501052	170.0095678	0.263363077	1.510223325	4.689826844	0
7.53E-05	0.660942902	0	0.065900288	0.564872126	0.149430295	0	1.004142332	0.006487485
0.3868333	2.244195808	4.4847836	0.915005983	0.008291391	0.65022329	38.46864448	0.219898314	0.013367053
0.202660752	0.267587504	26.53112047	0.355134453	0.687651412	3.95060498	0.093089477	3.908049116	1.600701297
13.89972963	0.014078114	217.5251456	0.140033911	0.000468885	162.4022311	0	16.60279417	1255.161428
0.482199379	0.004768307	0.02096569	0.782719448	0.048339941	4.304443401	0	0.201595404	2.005680679
7.53E-05	0.660942902	0	0.065900288	0.564872126	0.149430295	0	1.004142332	0.006487485
0.3868333	2.244195808	4.4847836	0.915005983	0.008291391	0.65022329	38.46864448	0.219898314	0.013367053
13.89972963	0.014078114	217.5251456	0.140033911	0.000468885	162.4022311	0	16.60279417	1255.161428
0.002944483	0.350527017	0	1.713718644	6.298087843	0.064775353	0	1.652685136	75.1631163
0.06621524	10.76762568	5.922218781	0.004727466	59.13728961	21.28584628	9.648578112	2.394832096	228.8283998
1.551536401	3.519831878	0.001530975	5.325409069	9.584778975	4.470502157	0.365076679	4.016569328	0.330986488
0.006830418	1.291992672	0.001294496	4.568261285	3.191565412	0.795624527	0.214944542	1.177884063	0.190483938
0.06621524	10.76762568	5.922218781	0.004727466	59.13728961	21.28584628	9.648578112	2.394832096	228.8283998
4.86638069	115.2345147	0.094371895	1.043598804	27.06276051	0.085760223	301.2456423	0.109543666	82.77163117
0.000277632	0.140479681	12.80283743	0.035237556	1.01902267	0.003195477	0.881575129	1.258687003	0
166.2297343	0.027677477	170.570441	58.34376362	69.99236234	187.5554183	0	0.617498982	0.005107595
0.067078768	0.06197952	0.858165504	0.583227345	0.000315376	0.276784403	0.041675687	0.210912513	0.003810947
0.202660752	0.267587504	26.53112047	0.355134453	0.687651412	3.95060498	0.093089477	3.908049116	1.600701297
0	0.057759406	0.11188111	0.18521563	5.42E-06	0.003848417	0.083913861	8.158583921	97.25208044
0.000277632	0.140479681	12.80283743	0.035237556	1.01902267	0.003195477	0.881575129	1.258687003	0
166.2297343	0.027677477	170.570441	58.34376362	69.99236234	187.5554183	0	0.617498982	0.005107595
0.202660752	0.267587504	26.53112047	0.355134453	0.687651412	3.95060498	0.093089477	3.908049116	1.600701297
0.06621524	10.76762568	5.922218781	0.004727466	59.13728961	21.28584628	9.648578112	2.394832096	228.8283998
31.55519048	0.037055819	43.22421264	0.604429302	12.12653733	0.073629724	0	0.562789221	2.345622231

39.63717417	77.93303846	0.003041902	0.890247112	0.357375193	42.90560519	0.752499175	0.944722788	0
39.63717417	77.93303846	0.003041902	0.890247112	0.357375193	42.90560519	0.752499175	0.944722788	0
0.202660752	0.267587504	26.53112047	0.355134453	0.687651412	3.95060498	0.093089477	3.908049116	1.600701297
31.55519048	0.037055819	43.22421264	0.604429302	12.12653733	0.073629724	0	0.562789221	2.345622231
31.55519048	0.037055819	43.22421264	0.604429302	12.12653733	0.073629724	0	0.562789221	2.345622231
0.041776317	60.62310832	1.039558825	1.234503664	47.21986769	0.033034676	0.453684698	0.220871964	154.4573253
166.2297343	0.027677477	170.570441	58.34376362	69.99236234	187.5554183	0	0.617498982	0.005107595
13.89972963	0.014078114	217.5251456	0.140033911	0.000468885	162.4022311	0	16.60279417	1255.161428
0.002944483	0.350527017	0	1.713718644	6.298087843	0.064775353	0	1.652685136	75.1631163
455.7871995	1.073543725	0.032222759	3.003501052	170.0095678	0.263363077	1.510223325	4.689826844	0
0.006830418	1.291992672	0.001294496	4.568261285	3.191565412	0.795624527	0.214944542	1.177884063	0.190483938
28.49628011	0.991344866	0.056525884	7.603402523	0.000690954	1.809089301	0.126285588	0.487990346	0.004370079
1.551536401	3.519831878	0.001530975	5.325409069	9.584778975	4.470502157	0.365076679	4.016569328	0.330986488
0	0.057759406	0.11188111	0.18521563	5.42E-06	0.003848417	0.083913861	8.158583921	97.25208044
21.22755434	0.362004239	0.458625717	60.87982682	84.45411124	0.260792482	0	0.200468657	0.028570969
39.63717417	77.93303846	0.003041902	0.890247112	0.357375193	42.90560519	0.752499175	0.944722788	0
39.63717417	77.93303846	0.003041902	0.890247112	0.357375193	42.90560519	0.752499175	0.944722788	0
455.7871995	1.073543725	0.032222759	3.003501052	170.0095678	0.263363077	1.510223325	4.689826844	0
0.000277632	0.140479681	12.80283743	0.035237556	1.01902267	0.003195477	0.881575129	1.258687003	0
13.89972963	0.014078114	217.5251456	0.140033911	0.000468885	162.4022311	0	16.60279417	1255.161428
0.000277632	0.140479681	12.80283743	0.035237556	1.01902267	0.003195477	0.881575129	1.258687003	0
39.63717417	77.93303846	0.003041902	0.890247112	0.357375193	42.90560519	0.752499175	0.944722788	0
0.482199379	0.004768307	0.02096569	0.782719448	0.048339941	4.304443401	0	0.201595404	2.005680679
1.551536401	3.519831878	0.001530975	5.325409069	9.584778975	4.470502157	0.365076679	4.016569328	0.330986488
31.55519048	0.037055819	43.22421264	0.604429302	12.12653733	0.073629724	0	0.562789221	2.345622231
0.3868333	2.244195808	4.4847836	0.915005983	0.008291391	0.65022329	38.46864448	0.219898314	0.013367053
31.55519048	0.037055819	43.22421264	0.604429302	12.12653733	0.073629724	0	0.562789221	2.345622231
21.22755434	0.362004239	0.458625717	60.87982682	84.45411124	0.260792482	0	0.200468657	0.028570969
0.000277632	0.140479681	12.80283743	0.035237556	1.01902267	0.003195477	0.881575129	1.258687003	0
0.000277632	0.140479681	12.80283743	0.035237556	1.01902267	0.003195477	0.881575129	1.258687003	0
166.2297343	0.027677477	170.570441	58.34376362	69.99236234	187.5554183	0	0.617498982	0.005107595
0.006830418	1.291992672	0.001294496	4.568261285	3.191565412	0.795624527	0.214944542	1.177884063	0.190483938
31.55519048	0.037055819	43.22421264	0.604429302	12.12653733	0.073629724	0	0.562789221	2.345622231
13.89972963	0.014078114	217.5251456	0.140033911	0.000468885	162.4022311	0	16.60279417	1255.161428
7.53E-05	0.660942902	0	0.065900288	0.564872126	0.149430295	0	1.004142332	0.006487485
0.000277632	0.140479681	12.80283743	0.035237556	1.01902267	0.003195477	0.881575129	1.258687003	0
39.63717417	77.93303846	0.003041902	0.890247112	0.357375193	42.90560519	0.752499175	0.944722788	0
0.041776317	60.62310832	1.039558825	1.234503664	47.21986769	0.033034676	0.453684698	0.220871964	154.4573253
455.7871995	1.073543725	0.032222759	3.003501052	170.0095678	0.263363077	1.510223325	4.689826844	0
0.067078768	0.06197952	0.858165504	0.583227345	0.000315376	0.276784403	0.041675687	0.210912513	0.003810947
13.89972963	0.014078114	217.5251456	0.140033911	0.000468885	162.4022311	0	16.60279417	1255.161428

0.202660752	0.267587504	26.53112047	0.355134453	0.687651412	3.95060498	0.093089477	3.908049116	1.600701297
0.006830418	1.291992672	0.001294496	4.568261285	3.191565412	0.795624527	0.214944542	1.177884063	0.190483938
0.006830418	1.291992672	0.001294496	4.568261285	3.191565412	0.795624527	0.214944542	1.177884063	0.190483938
4.86638069	115.2345147	0.094371895	1.043598804	27.06276051	0.085760223	301.2456423	0.109543666	82.77163117
0.202660752	0.267587504	26.53112047	0.355134453	0.687651412	3.95060498	0.093089477	3.908049116	1.600701297
4.86638069	115.2345147	0.094371895	1.043598804	27.06276051	0.085760223	301.2456423	0.109543666	82.77163117
0.482199379	0.004768307	0.02096569	0.782719448	0.048339941	4.304443401	0	0.201595404	2.005680679
0.3868333	2.244195808	4.4847836	0.915005983	0.008291391	0.65022329	38.46864448	0.219898314	0.013367053
21.22755434	0.362004239	0.458625717	60.87982682	84.45411124	0.260792482	0	0.200468657	0.028570969
0.482199379	0.004768307	0.02096569	0.782719448	0.048339941	4.304443401	0	0.201595404	2.005680679
0.067078768	0.06197952	0.858165504	0.583227345	0.000315376	0.276784403	0.041675687	0.210912513	0.003810947
7.53E-05	0.660942902	0	0.065900288	0.564872126	0.149430295	0	1.004142332	0.006487485
0.067078768	0.06197952	0.858165504	0.583227345	0.000315376	0.276784403	0.041675687	0.210912513	0.003810947
28.49628011	0.991344866	0.056525884	7.603402523	0.000690954	1.809089301	0.126285588	0.487990346	0.004370079
39.63717417	77.93303846	0.003041902	0.890247112	0.357375193	42.90560519	0.752499175	0.944722788	0
0.202660752	0.267587504	26.53112047	0.355134453	0.687651412	3.95060498	0.093089477	3.908049116	1.600701297
0.067078768	0.06197952	0.858165504	0.583227345	0.000315376	0.276784403	0.041675687	0.210912513	0.003810947
0.002944483	0.350527017	0	1.713718644	6.298087843	0.064775353	0	1.652685136	75.1631163
0.482199379	0.004768307	0.02096569	0.782719448	0.048339941	4.304443401	0	0.201595404	2.005680679
0.482199379	0.004768307	0.02096569	0.782719448	0.048339941	4.304443401	0	0.201595404	2.005680679
1.551536401	3.519831878	0.001530975	5.325409069	9.584778975	4.470502157	0.365076679	4.016569328	0.330986488
0	0.057759406	0.11188111	0.18521563	5.42E-06	0.003848417	0.083913861	8.158583921	97.25208044
21.22755434	0.362004239	0.458625717	60.87982682	84.45411124	0.260792482	0	0.200468657	0.028570969
21.22755434	0.362004239	0.458625717	60.87982682	84.45411124	0.260792482	0	0.200468657	0.028570969
455.7871995	1.073543725	0.032222759	3.003501052	170.0095678	0.263363077	1.510223325	4.689826844	0
0.202660752	0.267587504	26.53112047	0.355134453	0.687651412	3.95060498	0.093089477	3.908049116	1.600701297
0.3868333	2.244195808	4.4847836	0.915005983	0.008291391	0.65022329	38.46864448	0.219898314	0.013367053
0.000277632	0.140479681	12.80283743	0.035237556	1.01902267	0.003195477	0.881575129	1.258687003	0
0.06621524	10.76762568	5.922218781	0.004727466	59.13728961	21.28584628	9.648578112	2.394832096	228.8283998
59508.89074	21551.58748	4301.118895	11.27391116	195972.9386	246.3375064	0	1.387957088	0.010741314
39.63717417	77.93303846	0.003041902	0.890247112	0.357375193	42.90560519	0.752499175	0.944722788	0
0.482199379	0.004768307	0.02096569	0.782719448	0.048339941	4.304443401	0	0.201595404	2.005680679
13.89972963	0.014078114	217.5251456	0.140033911	0.000468885	162.4022311	0	16.60279417	1255.161428
4.86638069	115.2345147	0.094371895	1.043598804	27.06276051	0.085760223	301.2456423	0.109543666	82.77163117
13.89972963	0.014078114	217.5251456	0.140033911	0.000468885	162.4022311	0	16.60279417	1255.161428
0.067078768	0.06197952	0.858165504	0.583227345	0.000315376	0.276784403	0.041675687	0.210912513	0.003810947
39.63717417	77.93303846	0.003041902	0.890247112	0.357375193	42.90560519	0.752499175	0.944722788	0
0.3868333	2.244195808	4.4847836	0.915005983	0.008291391	0.65022329	38.46864448	0.219898314	0.013367053
0.067078768	0.06197952	0.858165504	0.583227345	0.000315376	0.276784403	0.041675687	0.210912513	0.003810947
1.551536401	3.519831878	0.001530975	5.325409069	9.584778975	4.470502157	0.365076679	4.016569328	0.330986488
0.041776317	60.62310832	1.039558825	1.234503664	47.21986769	0.033034676	0.453684698	0.220871964	154.4573253

1.551536401	3.519831878	0.001530975	5.325409069	9.584778975	4.470502157	0.365076679	4.016569328	0.330986488
0.041776317	60.62310832	1.039558825	1.234503664	47.21986769	0.033034676	0.453684698	0.220871964	154.4573253
166.2297343	0.027677477	170.570441	58.34376362	69.99236234	187.5554183	0	0.617498982	0.005107595
0.067078768	0.06197952	0.858165504	0.583227345	0.000315376	0.276784403	0.041675687	0.210912513	0.003810947
13.89972963	0.014078114	217.5251456	0.140033911	0.000468885	162.4022311	0	16.60279417	1255.161428
13.89972963	0.014078114	217.5251456	0.140033911	0.000468885	162.4022311	0	16.60279417	1255.161428
455.7871995	1.073543725	0.032222759	3.003501052	170.0095678	0.263363077	1.510223325	4.689826844	0
21.22755434	0.362004239	0.458625717	60.87982682	84.45411124	0.260792482	0	0.200468657	0.028570969
166.2297343	0.027677477	170.570441	58.34376362	69.99236234	187.5554183	0	0.617498982	0.005107595
0.041776317	60.62310832	1.039558825	1.234503664	47.21986769	0.033034676	0.453684698	0.220871964	154.4573253
0.000277632	0.140479681	12.80283743	0.035237556	1.01902267	0.003195477	0.881575129	1.258687003	0
0.202660752	0.267587504	26.53112047	0.355134453	0.687651412	3.95060498	0.093089477	3.908049116	1.600701297
0.006830418	1.291992672	0.001294496	4.568261285	3.191565412	0.795624527	0.214944542	1.177884063	0.190483938
4.86638069	115.2345147	0.094371895	1.043598804	27.06276051	0.085760223	301.2456423	0.109543666	82.77163117
166.2297343	0.027677477	170.570441	58.34376362	69.99236234	187.5554183	0	0.617498982	0.005107595
0.002944483	0.350527017	0	1.713718644	6.298087843	0.064775353	0	1.652685136	75.1631163
0.002944483	0.350527017	0	1.713718644	6.298087843	0.064775353	0	1.652685136	75.1631163
0.482199379	0.004768307	0.02096569	0.782719448	0.048339941	4.304443401	0	0.201595404	2.005680679
0.482199379	0.004768307	0.02096569	0.782719448	0.048339941	4.304443401	0	0.201595404	2.005680679
0.202660752	0.267587504	26.53112047	0.355134453	0.687651412	3.95060498	0.093089477	3.908049116	1.600701297
39.63717417	77.93303846	0.003041902	0.890247112	0.357375193	42.90560519	0.752499175	0.944722788	0
39.63717417	77.93303846	0.003041902	0.890247112	0.357375193	42.90560519	0.752499175	0.944722788	0
455.7871995	1.073543725	0.032222759	3.003501052	170.0095678	0.263363077	1.510223325	4.689826844	0
0.202660752	0.267587504	26.53112047	0.355134453	0.687651412	3.95060498	0.093089477	3.908049116	1.600701297
0.06621524	10.76762568	5.922218781	0.004727466	59.13728961	21.28584628	9.648578112	2.394832096	228.8283998
166.2297343	0.027677477	170.570441	58.34376362	69.99236234	187.5554183	0	0.617498982	0.005107595
0.06621524	10.76762568	5.922218781	0.004727466	59.13728961	21.28584628	9.648578112	2.394832096	228.8283998
4.86638069	115.2345147	0.094371895	1.043598804	27.06276051	0.085760223	301.2456423	0.109543666	82.77163117
0.482199379	0.004768307	0.02096569	0.782719448	0.048339941	4.304443401	0	0.201595404	2.005680679
0.202660752	0.267587504	26.53112047	0.355134453	0.687651412	3.95060498	0.093089477	3.908049116	1.600701297
59508.89074	21551.58748	4301.118895	11.27391116	195972.9386	246.3375064	0	1.387957088	0.010741314
4.86638069	115.2345147	0.094371895	1.043598804	27.06276051	0.085760223	301.2456423	0.109543666	82.77163117
21.22755434	0.362004239	0.458625717	60.87982682	84.45411124	0.260792482	0	0.200468657	0.028570969
21.22755434	0.362004239	0.458625717	60.87982682	84.45411124	0.260792482	0	0.200468657	0.028570969
0	0.057759406	0.11188111	0.18521563	5.42E-06	0.003848417	0.083913861	8.158583921	97.25208044
21.22755434	0.362004239	0.458625717	60.87982682	84.45411124	0.260792482	0	0.200468657	0.028570969
4.86638069	115.2345147	0.094371895	1.043598804	27.06276051	0.085760223	301.2456423	0.109543666	82.77163117
31.55519048	0.037055819	43.22421264	0.604429302	12.12653733	0.073629724	0	0.562789221	2.345622231
4.86638069	115.2345147	0.094371895	1.043598804	27.06276051	0.085760223	301.2456423	0.109543666	82.77163117
31.55519048	0.037055819	43.22421264	0.604429302	12.12653733	0.073629724	0	0.562789221	2.345622231
0.202660752	0.267587504	26.53112047	0.355134453	0.687651412	3.95060498	0.093089477	3.908049116	1.600701297

0.000277632	0.140479681	12.80283743	0.035237556	1.01902267	0.003195477	0.881575129	1.258687003	0
7.53E-05	0.660942902	0	0.065900288	0.564872126	0.149430295	0	1.004142332	0.006487485
28.49628011	0.991344866	0.056525884	7.603402523	0.000690954	1.809089301	0.126285588	0.487990346	0.004370079
0.002944483	0.350527017	0	1.713718644	6.298087843	0.064775353	0	1.652685136	75.1631163
0.482199379	0.004768307	0.02096569	0.782719448	0.048339941	4.304443401	0	0.201595404	2.005680679
0	0.057759406	0.11188111	0.18521563	5.42E-06	0.003848417	0.083913861	8.158583921	97.25208044
0.006830418	1.291992672	0.001294496	4.568261285	3.191565412	0.795624527	0.214944542	1.177884063	0.190483938
166.2297343	0.027677477	170.570441	58.34376362	69.99236234	187.5554183	0	0.617498982	0.005107595
0.3868333	2.244195808	4.4847836	0.915005983	0.008291391	0.65022329	38.46864448	0.219898314	0.013367053
31.55519048	0.037055819	43.22421264	0.604429302	12.12653733	0.073629724	0	0.562789221	2.345622231
0.06621524	10.76762568	5.922218781	0.004727466	59.13728961	21.28584628	9.648578112	2.394832096	228.8283998
7.53E-05	0.660942902	0	0.065900288	0.564872126	0.149430295	0	1.004142332	0.006487485
0.067078768	0.06197952	0.858165504	0.583227345	0.000315376	0.276784403	0.041675687	0.210912513	0.003810947
28.49628011	0.991344866	0.056525884	7.603402523	0.000690954	1.809089301	0.126285588	0.487990346	0.004370079
0.067078768	0.06197952	0.858165504	0.583227345	0.000315376	0.276784403	0.041675687	0.210912513	0.003810947
0.482199379	0.004768307	0.02096569	0.782719448	0.048339941	4.304443401	0	0.201595404	2.005680679
39.63717417	77.93303846	0.003041902	0.890247112	0.357375193	42.90560519	0.752499175	0.944722788	0
0.067078768	0.06197952	0.858165504	0.583227345	0.000315376	0.276784403	0.041675687	0.210912513	0.003810947
5	6	7	8	9	10	11	12	13
hsa-mir-548l	ENSG00000201980	ENSG00000202231	hsa-mir-216b	hsa-mir-98	hsa-mir-26b	hsa-mir-581 F1	hsa-mir-450b	ENSG00000212363

14	15	16	17	18	19	20
ENSG00000199282	hsa-mir-523	hsa-mir-376a-2/1 F2	ENSG00000199856 F1	HBII-276 F2	RASGRP4-Hs01073179_m1	FAM20A-Hs01034071_m1
0.119203401	0.029279411	2.109358697	0.013621435	42.52432992	0.537256281	0.252080729
4.426904132	0.084613835	0.003441173	272.9917946	85.0364028	0.003660394	98.79935885
4.473171684	0.076842067	18.84694345	0.013625472	0.076963163	0.68490409	354028.3969
0.080286327	11.19987043	0.285204293	0.026667805	15.13250151	1.457575901	0.193734365
0.253923696	3.955262463	0.388654976	19.8726695	28.64235035	0.95201081	0.126733591
0.035823056	1.430901638	1.271245691	0.13808893	8.638026232	6.934406425	0.001357146
0.5070676	0.190717571	1.262592096	0.001397551	2.57731033	2.279271904	0.293543945
0.119203401	0.029279411	2.109358697	0.013621435	42.52432992	0.012402781	0.002675154
92.75059664	22.92764044	136.8974438	1.745129449	0.773949798	0.030980833	0.000303502
0.178123738	0.050917688	0.342800111	0.111057257	0.005623125	0.941138757	0.654166576
0.080286327	11.19987043	0.285204293	0.026667805	15.13250151	0.241399688	0.009831155
0.5070676	0.190717571	1.262592096	0.001397551	2.57731033	1.457575901	0.193734365
0.119203401	0.029279411	2.109358697	0.013621435	42.52432992	0.162454545	1.696772287
0.119203401	0.029279411	2.109358697	0.013621435	42.52432992	0.030980833	0.000303502
0.080286327	11.19987043	0.285204293	0.026667805	15.13250151	7.79E-05	125.6834219
0.387705712	9.046223838	1.026712467	0.000692681	0.013733268	109.6184513	10.28913504
4.473171684	0.076842067	18.84694345	0.013625472	0.076963163	2.544174395	0.160409644
92.75059664	22.92764044	136.8974438	1.745129449	0.773949798	5.54985007	10376091.68
4.473171684	0.076842067	18.84694345	0.013625472	0.076963163	0.68490409	354028.3969
0.25730499	1.003482072	3.432601874	0.147105717	0.06663811	1.83506595	12.44200598
0.119203401	0.029279411	2.109358697	0.013621435	42.52432992	109.6184513	10.28913504
0.119203401	0.029279411	2.109358697	0.013621435	42.52432992	0.48613162	0.091637471
0.054797682	0.076910682	0.242603877	14.84717204	12.40461821	23.770735	818.9848289
16.62552482	0.955293521	0.000427897	0.007789606	0.083975682	0.241399688	0.009831155
92.75059664	22.92764044	136.8974438	1.745129449	0.773949798	0.95201081	0.126733591
0.387705712	9.046223838	1.026712467	0.000692681	0.013733268	23.770735	818.9848289
0.080286327	11.19987043	0.285204293	0.026667805	15.13250151	0.941138757	0.654166576
15829.49903	56.38797756	29457.10391	1418886.715	136.9497239	0.241399688	0.009831155
16.62552482	0.955293521	0.000427897	0.007789606	0.083975682	7.79E-05	125.6834219
16.62552482	0.955293521	0.000427897	0.007789606	0.083975682	0.030980833	0.000303502
0.104782275	2.928019815	0.712680807	16.19095592	0.004677679	0.537256281	0.252080729
0.253923696	3.955262463	0.388654976	19.8726695	28.64235035	20.37140179	1.406067894
0.035823056	1.430901638	1.271245691	0.13808893	8.638026232	109.6184513	10.28913504
16.62552482	0.955293521	0.000427897	0.007789606	0.083975682	6.934406425	0.001357146
0.035823056	1.430901638	1.271245691	0.13808893	8.638026232	0.152264752	0.349909753
0.082523665	0.768079633	0.220114788	12.26669345	0.001626569	0.941138757	0.654166576
0.035823056	1.430901638	1.271245691	0.13808893	8.638026232	0.48613162	0.091637471

0.25730499	1.003482072	3.432601874	0.147105717	0.06663811	23.770735	818.9848289
0.280382186	0.426172578	0.138339462	2.71570727	6.459684835	6.934406425	0.001357146
0.104782275	2.928019815	0.712680807	16.19095592	0.004677679	2569.44178	1.000491928
0.279452408	7.278686543	0.605816047	0.039376179	6.718436675	109.6184513	10.28913504
0.178123738	0.050917688	0.342800111	0.111057257	0.005623125	0.941138757	0.654166576
15829.49903	56.38797756	29457.10391	1418886.715	136.9497239	6.934406425	0.001357146
0.916411587	0.301042719	1.727580032	0.331645482	18.79421738	20.37140179	1.406067894
0.080286327	11.19987043	0.285204293	0.026667805	15.13250151	1.83506595	12.44200598
4.473171684	0.076842067	18.84694345	0.013625472	0.076963163	0.95201081	0.126733591
16.62552482	0.955293521	0.000427897	0.007789606	0.083975682	2569.44178	1.000491928
0.5070676	0.190717571	1.262592096	0.001397551	2.57731033	0.95201081	0.126733591
1.01442236	4.903369331	1.004944801	0.348315586	2.979999097	0.68490409	354028.3969
16.62552482	0.955293521	0.000427897	0.007789606	0.083975682	0.48613162	0.091637471
3.021486852	53.40437571	2.793263543	85.11011316	12.7755358	2.861748399	5.044338165
0.434237766	0.083081926	0.339648941	23.65470768	0.345081411	1203.152028	8852.522525
0.189064125	31.25389488	0.017825268	0.218741389	0.064588327	564.8652401	35.15101961
1.131873684	0.104495598	0.409746435	0.658133378	0.010463819	994.735656	7.927859603
6.976323472	0.07789373	6.48504354	0.00650483	0.041251428	5.561172778	0.000704147
1.834601339	23.40729065	0.01153332	0.001577605	82.17921804	46.20629421	1878431.817
146.2283384	108.9343921	159.5737034	14487.86218	138.0280438	5.460699865	0.288803204
2.996728801	0.005077949	6.29290126	506.7283609	0.121232565	994.735656	7.927859603
0.434237766	0.083081926	0.339648941	23.65470768	0.345081411	5.783017396	27.19953855
1.233624785	0.002468732	1.324569921	19.35224231	0.223754321	109.85648	0.961963177
1.435246096	0.019050387	1.926961808	67.24171692	0.166229381	564.8652401	35.15101961
146.2283384	108.9343921	159.5737034	14487.86218	138.0280438	3.130594869	0.030139416
2.996728801	0.005077949	6.29290126	506.7283609	0.121232565	46.20629421	1878431.817
0.189064125	31.25389488	0.017825268	0.218741389	0.064588327	89.45877868	159.4564655
0.189064125	31.25389488	0.017825268	0.218741389	0.064588327	3.130594869	0.030139416
1.606268382	1.31810948	2.161378439	5.246904741	1.315227066	994.735656	7.927859603
1.294216626	1.472267813	0.017485495	0.233785035	0.133971022	46.20629421	1878431.817
2.996728801	0.005077949	6.29290126	506.7283609	0.121232565	1.749696823	0.000359589
2.996728801	0.005077949	6.29290126	506.7283609	0.121232565	5.461427683	1077080.988
3.021486852	53.40437571	2.793263543	85.11011316	12.7755358	5.783017396	27.19953855
0.616126345	7.810474176	4.322315691	0.000859768	29.67646416	3.130594869	0.030139416
1.834601339	23.40729065	0.01153332	0.001577605	82.17921804	1382.594644	0.305071512
1.834601339	23.40729065	0.01153332	0.001577605	82.17921804	994.735656	7.927859603
6.976323472	0.07789373	6.48504354	0.00650483	0.041251428	5.460699865	0.288803204
1.233624785	0.002468732	1.324569921	19.35224231	0.223754321	3.130594869	0.030139416
0.616126345	7.810474176	4.322315691	0.000859768	29.67646416	994.735656	7.927859603
0.054042941	0.067966332	0.323370206	0.005361322	3.543836129	3.130594869	0.030139416
2.996728801	0.005077949	6.29290126	506.7283609	0.121232565	3.709490109	4.052575238

0.10440408	0.106161149	0.220974484	13.00925642	4.61085615	0.417959285	7322.272056
1.834601339	23.40729065	0.01153332	0.001577605	82.17921804	1203.152028	8852.522525
0.189064125	31.25389488	0.017825268	0.218741389	0.064588327	564.8652401	35.15101961
0.10440408	0.106161149	0.220974484	13.00925642	4.61085615	5.561172778	0.000704147
0.068248803	25.03655374	2.29750437	0.005371323	40.85597868	3.096116985	7490724.43
1.606268382	1.31810948	2.161378439	5.246904741	1.315227066	5.783017396	27.19953855
1.467858565	26.83914793	4.171133489	16.73814337	0.033727705	3.709490109	4.052575238
0.068248803	25.03655374	2.29750437	0.005371323	40.85597868	1813.425348	119142.7764
0.10440408	0.106161149	0.220974484	13.00925642	4.61085615	5.783017396	27.19953855
1.467858565	26.83914793	4.171133489	16.73814337	0.033727705	0.350754565	0.29243781
2.996728801	0.005077949	6.29290126	506.7283609	0.121232565	1382.594644	0.305071512
0.616126345	7.810474176	4.322315691	0.000859768	29.67646416	0.435715791	121.8315447
3.021486852	53.40437571	2.793263543	85.11011316	12.7755358	46.20629421	1878431.817
3.021486852	53.40437571	2.793263543	85.11011316	12.7755358	994.735656	7.927859603
0.434237766	0.083081926	0.339648941	23.65470768	0.345081411	46.20629421	1878431.817
0.434237766	0.083081926	0.339648941	23.65470768	0.345081411	3.130594869	0.030139416
0.045881857	0.060830394	1.121507667	37.59574032	0.842340423	3.130594869	0.030139416
2.996728801	0.005077949	6.29290126	506.7283609	0.121232565	3.130594869	0.030139416
2.991096614	2.049696262	1.065523117	0.179610991	14.80615949	3.130594869	0.030139416
1.131873684	0.104495598	0.409746435	0.658133378	0.010463819	3.130594869	0.030139416
1.294216626	1.472267813	0.017485495	0.233785035	0.133971022	10.66908927	0.028558397
0.068248803	25.03655374	2.29750437	0.005371323	40.85597868	5.783017396	27.19953855
6.976323472	0.07789373	6.48504354	0.00650483	0.041251428	994.735656	7.927859603
0.58449341	3.212285593	1.219583574	0.000101639	0.501844355	564.8652401	35.15101961
6.976323472	0.07789373	6.48504354	0.00650483	0.041251428	994.735656	7.927859603
1.294216626	1.472267813	0.017485495	0.233785035	0.133971022	46.20629421	1878431.817
0.10440408	0.106161149	0.220974484	13.00925642	4.61085615	5.783017396	27.19953855
0.10440408	0.106161149	0.220974484	13.00925642	4.61085615	0.038147525	12.2829238
3.021486852	53.40437571	2.793263543	85.11011316	12.7755358	3.130594869	0.030139416
7.979498795	0.010492223	6.538933844	233.7877338	0.053036836	3.130594869	0.030139416
7.979498795	0.010492223	6.538933844	233.7877338	0.053036836	537.637133	0.502829598
0.054042941	0.067966332	0.323370206	0.005361322	3.543836129	0.235283773	761.746319
7.979498795	0.010492223	6.538933844	233.7877338	0.053036836	0.038147525	12.2829238
0.434237766	0.083081926	0.339648941	23.65470768	0.345081411	46.20629421	1878431.817
0.434237766	0.083081926	0.339648941	23.65470768	0.345081411	0.046588643	1.783326953
146.2283384	108.9343921	159.5737034	14487.86218	138.0280438	109.85648	0.961963177
1.606268382	1.31810948	2.161378439	5.246904741	1.315227066	2.861748399	5.044338165
0.616126345	7.810474176	4.322315691	0.000859768	29.67646416	1813.425348	119142.7764
3.021486852	53.40437571	2.793263543	85.11011316	12.7755358	5.561172778	0.000704147
1.606268382	1.31810948	2.161378439	5.246904741	1.315227066	46.20629421	1878431.817
1.435246096	0.019050387	1.926961808	67.24171692	0.166229381	1813.425348	119142.7764

1.294216626	1.472267813	0.017485495	0.233785035	0.133971022	3.130594869	0.030139416
1.834601339	23.40729065	0.01153332	0.001577605	82.17921804	7.972298343	92.02553404
2.996728801	0.005077949	6.29290126	506.7283609	0.121232565	46.20629421	1878431.817
0.189064125	31.25389488	0.017825268	0.218741389	0.064588327	109.85648	0.961963177
146.2283384	108.9343921	159.5737034	14487.86218	138.0280438	994.735656	7.927859603
0.434237766	0.083081926	0.339648941	23.65470768	0.345081411	46.20629421	1878431.817
1.233624785	0.002468732	1.324569921	19.35224231	0.223754321	564.8652401	35.15101961
1.606268382	1.31810948	2.161378439	5.246904741	1.315227066	7.972298343	92.02553404
1.233624785	0.002468732	1.324569921	19.35224231	0.223754321	46.20629421	1878431.817
1.606268382	1.31810948	2.161378439	5.246904741	1.315227066	994.735656	7.927859603
0.434237766	0.083081926	0.339648941	23.65470768	0.345081411	3.130594869	0.030139416
1.834601339	23.40729065	0.01153332	0.001577605	82.17921804	5.460699865	0.288803204
0.068248803	25.03655374	2.29750437	0.005371323	40.85597868	46.20629421	1878431.817
0.045881857	0.060830394	1.121507667	37.59574032	0.842340423	994.735656	7.927859603
0.434237766	0.083081926	0.339648941	23.65470768	0.345081411	46.20629421	1878431.817
2.991096614	2.049696262	1.065523117	0.179610991	14.80615949	5.460699865	0.288803204
146.2283384	108.9343921	159.5737034	14487.86218	138.0280438	46.20629421	1878431.817
146.2283384	108.9343921	159.5737034	14487.86218	138.0280438	204.3719434	2827259.242
2.991096614	2.049696262	1.065523117	0.179610991	14.80615949	0.038147525	12.2829238
7.979498795	0.010492223	6.538933844	233.7877338	0.053036836	1382.594644	0.305071512
3.021486852	53.40437571	2.793263543	85.11011316	12.7755358	5.256930015	0.006286635
2.991096614	2.049696262	1.065523117	0.179610991	14.80615949	10.66908927	0.028558397
0.189064125	31.25389488	0.017825268	0.218741389	0.064588327	994.735656	7.927859603
0.054042941	0.067966332	0.323370206	0.005361322	3.543836129	5.461427683	1077080.988
6.976323472	0.07789373	6.48504354	0.00650483	0.041251428	5.561172778	0.000704147
1.131873684	0.104495598	0.409746435	0.658133378	0.010463819	0.235283773	761.746319
3.021486852	53.40437571	2.793263543	85.11011316	12.7755358	7.972298343	92.02553404
1.131873684	0.104495598	0.409746435	0.658133378	0.010463819	5.460699865	0.288803204
0.045881857	0.060830394	1.121507667	37.59574032	0.842340423	1.401537911	0.002591964
0.434237766	0.083081926	0.339648941	23.65470768	0.345081411	5.256930015	0.006286635
1.435246096	0.019050387	1.926961808	67.24171692	0.166229381	994.735656	7.927859603
0.189064125	31.25389488	0.017825268	0.218741389	0.064588327	3.709490109	4.052575238
0.054042941	0.067966332	0.323370206	0.005361322	3.543836129	7.972298343	92.02553404
1.131873684	0.104495598	0.409746435	0.658133378	0.010463819	994.735656	7.927859603
1.834601339	23.40729065	0.01153332	0.001577605	82.17921804	5.561172778	0.000704147
0.068248803	25.03655374	2.29750437	0.005371323	40.85597868	3.130594869	0.030139416
1.294216626	1.472267813	0.017485495	0.233785035	0.133971022	46.20629421	1878431.817
0.434237766	0.083081926	0.339648941	23.65470768	0.345081411	1203.152028	8852.522525
0.434237766	0.083081926	0.339648941	23.65470768	0.345081411	994.735656	7.927859603
0.616126345	7.810474176	4.322315691	0.000859768	29.67646416	994.735656	7.927859603
0.189064125	31.25389488	0.017825268	0.218741389	0.064588327	5.460699865	0.288803204

0.068248803	25.03655374	2.29750437	0.005371323	40.85597868	1813.425348	119142.7764
7.979498795	0.010492223	6.538933844	233.7877338	0.053036836	10.66908927	0.028558397
1.131873684	0.104495598	0.409746435	0.658133378	0.010463819	98.37328866	205.7148389
1.435246096	0.019050387	1.926961808	67.24171692	0.166229381	0.024990864	512.289609
6.976323472	0.07789373	6.48504354	0.00650483	0.041251428	0.474239135	71.62581966
1.606268382	1.31810948	2.161378439	5.246904741	1.315227066	1382.594644	0.305071512
6.976323472	0.07789373	6.48504354	0.00650483	0.041251428	7.972298343	92.02553404
1.294216626	1.472267813	0.017485495	0.233785035	0.133971022	5.783017396	27.19953855
0.189064125	31.25389488	0.017825268	0.218741389	0.064588327	109.85648	0.961963177
0.434237766	0.083081926	0.339648941	23.65470768	0.345081411	0.038147525	12.2829238
1.233624785	0.002468732	1.324569921	19.35224231	0.223754321	5.256930015	0.006286635
1.834601339	23.40729065	0.01153332	0.001577605	82.17921804	3.709490109	4.052575238
6.976323472	0.07789373	6.48504354	0.00650483	0.041251428	3.130594869	0.030139416
0.58449341	3.212285593	1.219583574	0.000101639	0.501844355	3.130594869	0.030139416
1.834601339	23.40729065	0.01153332	0.001577605	82.17921804	5.783017396	27.19953855
146.2283384	108.9343921	159.5737034	14487.86218	138.0280438	3.130594869	0.030139416
0.189064125	31.25389488	0.017825268	0.218741389	0.064588327	3.130594869	0.030139416
1.233624785	0.002468732	1.324569921	19.35224231	0.223754321	3.130594869	0.030139416
146.2283384	108.9343921	159.5737034	14487.86218	138.0280438	3.130594869	0.030139416
1.606268382	1.31810948	2.161378439	5.246904741	1.315227066	5.783017396	27.19953855
146.2283384	108.9343921	159.5737034	14487.86218	138.0280438	46.20629421	1878431.817
1.131873684	0.104495598	0.409746435	0.658133378	0.010463819	3.130594869	0.030139416
0.616126345	7.810474176	4.322315691	0.000859768	29.67646416	994.735656	7.927859603
0.58449341	3.212285593	1.219583574	0.000101639	0.501844355	3.130594869	0.030139416
0.189064125	31.25389488	0.017825268	0.218741389	0.064588327	1.749696823	0.000359589
1.294216626	1.472267813	0.017485495	0.233785035	0.133971022	0.235283773	761.746319
2.991096614	2.049696262	1.065523117	0.179610991	14.80615949	3.709490109	4.052575238
2.991096614	2.049696262	1.065523117	0.179610991	14.80615949	3.709490109	4.052575238
0.189064125	31.25389488	0.017825268	0.218741389	0.064588327	1203.152028	8852.522525
1.606268382	1.31810948	2.161378439	5.246904741	1.315227066	0.148368246	0.032231981
0.58449341	3.212285593	1.219583574	0.000101639	0.501844355	3.130594869	0.030139416
1.467858565	26.83914793	4.171133489	16.73814337	0.033727705	994.735656	7.927859603
0.189064125	31.25389488	0.017825268	0.218741389	0.064588327	1.401537911	0.002591964
0.045881857	0.060830394	1.121507667	37.59574032	0.842340423	0.001671576	0.001066916
3.021486852	53.40437571	2.793263543	85.11011316	12.7755358	994.735656	7.927859603
0.616126345	7.810474176	4.322315691	0.000859768	29.67646416	1.401537911	0.002591964
2.996728801	0.005077949	6.29290126	506.7283609	0.121232565	1813.425348	119142.7764
1.233624785	0.002468732	1.324569921	19.35224231	0.223754321	564.8652401	35.15101961
0.189064125	31.25389488	0.017825268	0.218741389	0.064588327	994.735656	7.927859603
1.467858565	26.83914793	4.171133489	16.73814337	0.033727705	994.735656	7.927859603
1.467858565	26.83914793	4.171133489	16.73814337	0.033727705	3.130594869	0.030139416

0.58449341	3.212285593	1.219583574	0.000101639	0.501844355	1.401537911	0.002591964
146.2283384	108.9343921	159.5737034	14487.86218	138.0280438	5.783017396	27.19953855
0.58449341	3.212285593	1.219583574	0.000101639	0.501844355	3.130594869	0.030139416
0.58449341	3.212285593	1.219583574	0.000101639	0.501844355	0.235283773	761.746319
6.976323472	0.07789373	6.48504354	0.00650483	0.041251428	994.735656	7.927859603
0.434237766	0.083081926	0.339648941	23.65470768	0.345081411	0.417959285	7322.272056
0.045881857	0.060830394	1.121507667	37.59574032	0.842340423	5.561172778	0.000704147
0.045881857	0.060830394	1.121507667	37.59574032	0.842340423	0.417959285	7322.272056
146.2283384	108.9343921	159.5737034	14487.86218	138.0280438	109.85648	0.961963177
6.976323472	0.07789373	6.48504354	0.00650483	0.041251428	5.256930015	0.006286635
3.021486852	53.40437571	2.793263543	85.11011316	12.7755358	5.460699865	0.288803204
0.054042941	0.067966332	0.323370206	0.005361322	3.543836129	46.20629421	1878431.817
1.467858565	26.83914793	4.171133489	16.73814337	0.033727705	994.735656	7.927859603
0.189064125	31.25389488	0.017825268	0.218741389	0.064588327	5.561172778	0.000704147
3.021486852	53.40437571	2.793263543	85.11011316	12.7755358	3.130594869	0.030139416
2.991096614	2.049696262	1.065523117	0.179610991	14.80615949	5.460699865	0.288803204
1.233624785	0.002468732	1.324569921	19.35224231	0.223754321	10.66908927	0.028558397
1.435246096	0.019050387	1.926961808	67.24171692	0.166229381	0.435715791	121.8315447
1.131873684	0.104495598	0.409746435	0.658133378	0.010463819	1203.152028	8852.522525
0.58449341	3.212285593	1.219583574	0.000101639	0.501844355	1.401537911	0.002591964
0.054042941	0.067966332	0.323370206	0.005361322	3.543836129	0.235283773	761.746319
2.991096614	2.049696262	1.065523117	0.179610991	14.80615949	46.20629421	1878431.817
1.131873684	0.104495598	0.409746435	0.658133378	0.010463819	1813.425348	119142.7764
146.2283384	108.9343921	159.5737034	14487.86218	138.0280438	3.709490109	4.052575238
1.606268382	1.31810948	2.161378439	5.246904741	1.315227066	1382.594644	0.305071512
1.294216626	1.472267813	0.017485495	0.233785035	0.133971022	46.20629421	1878431.817
0.054042941	0.067966332	0.323370206	0.005361322	3.543836129	3.130594869	0.030139416
3.021486852	53.40437571	2.793263543	85.11011316	12.7755358	3.130594869	0.030139416
0.189064125	31.25389488	0.017825268	0.218741389	0.064588327	5.561172778	0.000704147
1.435246096	0.019050387	1.926961808	67.24171692	0.166229381	3.130594869	0.030139416
2.991096614	2.049696262	1.065523117	0.179610991	14.80615949	5.460699865	0.288803204
0.189064125	31.25389488	0.017825268	0.218741389	0.064588327	3.130594869	0.030139416
0.045881857	0.060830394	1.121507667	37.59574032	0.842340423	3.130594869	0.030139416
2.991096614	2.049696262	1.065523117	0.179610991	14.80615949	46.20629421	1878431.817
1.233624785	0.002468732	1.324569921	19.35224231	0.223754321	46.20629421	1878431.817
3.021486852	53.40437571	2.793263543	85.11011316	12.7755358	994.735656	7.927859603
0.189064125	31.25389488	0.017825268	0.218741389	0.064588327	5.783017396	27.19953855
1.435246096	0.019050387	1.926961808	67.24171692	0.166229381	46.20629421	1878431.817
7.979498795	0.010492223	6.538933844	233.7877338	0.053036836	1382.594644	0.305071512
0.045881857	0.060830394	1.121507667	37.59574032	0.842340423	3.130594869	0.030139416
0.045881857	0.060830394	1.121507667	37.59574032	0.842340423	994.735656	7.927859603

0.045881857	0.060830394	1.121507667	37.59574032	0.842340423	5.460699865	0.288803204
6.976323472	0.07789373	6.48504354	0.00650483	0.041251428	1.401537911	0.002591964
0.054042941	0.067966332	0.323370206	0.005361322	3.543836129	5.783017396	27.19953855
0.10440408	0.106161149	0.220974484	13.00925642	4.61085615	5.461427683	1077080.988
1.467858565	26.83914793	4.171133489	16.73814337	0.033727705	3.709490109	4.052575238
0.189064125	31.25389488	0.017825268	0.218741389	0.064588327	0.417959285	7322.272056
0.10440408	0.106161149	0.220974484	13.00925642	4.61085615	5.561172778	0.000704147
1.233624785	0.002468732	1.324569921	19.35224231	0.223754321	0.235283773	761.746319
1.606268382	1.31810948	2.161378439	5.246904741	1.315227066	5.256930015	0.006286635
2.991096614	2.049696262	1.065523117	0.179610991	14.80615949	994.735656	7.927859603
0.10440408	0.106161149	0.220974484	13.00925642	4.61085615	5.561172778	0.000704147
1.294216626	1.472267813	0.017485495	0.233785035	0.133971022	5.256930015	0.006286635
0.045881857	0.060830394	1.121507667	37.59574032	0.842340423	0.235283773	761.746319
0.58449341	3.212285593	1.219583574	0.000101639	0.501844355	1813.425348	119142.7764
7.979498795	0.010492223	6.538933844	233.7877338	0.053036836	7.972298343	92.02553404
0.58449341	3.212285593	1.219583574	0.000101639	0.501844355	994.735656	7.927859603
0.434237766	0.083081926	0.339648941	23.65470768	0.345081411	1382.594644	0.305071512
2.996728801	0.005077949	6.29290126	506.7283609	0.121232565	10.66908927	0.028558397
6.976323472	0.07789373	6.48504354	0.00650483	0.041251428	3.709490109	4.052575238
0.434237766	0.083081926	0.339648941	23.65470768	0.345081411	3.130594869	0.030139416
146.2283384	108.9343921	159.5737034	14487.86218	138.0280438	5.561172778	0.000704147
0.045881857	0.060830394	1.121507667	37.59574032	0.842340423	3.130594869	0.030139416
0.10440408	0.106161149	0.220974484	13.00925642	4.61085615	994.735656	7.927859603
146.2283384	108.9343921	159.5737034	14487.86218	138.0280438	1382.594644	0.305071512
0.189064125	31.25389488	0.017825268	0.218741389	0.064588327	3.130594869	0.030139416
6.976323472	0.07789373	6.48504354	0.00650483	0.041251428	46.20629421	1878431.817
3.021486852	53.40437571	2.793263543	85.11011316	12.7755358	5.256930015	0.006286635
6.976323472	0.07789373	6.48504354	0.00650483	0.041251428	5.256930015	0.006286635
146.2283384	108.9343921	159.5737034	14487.86218	138.0280438	89.45877868	159.4564655
1.435246096	0.019050387	1.926961808	67.24171692	0.166229381	994.735656	7.927859603
0.58449341	3.212285593	1.219583574	0.000101639	0.501844355	1382.594644	0.305071512
7.979498795	0.010492223	6.538933844	233.7877338	0.053036836	3.130594869	0.030139416
2.991096614	2.049696262	1.065523117	0.179610991	14.80615949	0.223448506	0.834984272
1.435246096	0.019050387	1.926961808	67.24171692	0.166229381	3.130594869	0.030139416
6.976323472	0.07789373	6.48504354	0.00650483	0.041251428	5.256930015	0.006286635
0.054042941	0.067966332	0.323370206	0.005361322	3.543836129	0.235283773	761.746319
0.58449341	3.212285593	1.219583574	0.000101639	0.501844355	5.460699865	0.288803204
3.021486852	53.40437571	2.793263543	85.11011316	12.7755358	7.972298343	92.02553404
1.131873684	0.104495598	0.409746435	0.658133378	0.010463819	994.735656	7.927859603
1.294216626	1.472267813	0.017485495	0.233785035	0.133971022	3.130594869	0.030139416
146.2283384	108.9343921	159.5737034	14487.86218	138.0280438	46.20629421	1878431.817

1.435246096	0.019050387	1.926961808	67.24171692	0.166229381	46.20629421	1878431.817
1.834601339	23.40729065	0.01153332	0.001577605	82.17921804	994.735656	7.927859603
3.021486852	53.40437571	2.793263543	85.11011316	12.7755358	3.130594869	0.030139416
1.233624785	0.002468732	1.324569921	19.35224231	0.223754321	0.223448506	0.834984272
2.996728801	0.005077949	6.29290126	506.7283609	0.121232565	3.130594869	0.030139416
0.045881857	0.060830394	1.121507667	37.59574032	0.842340423	3.130594869	0.030139416
1.606268382	1.31810948	2.161378439	5.246904741	1.315227066	46.20629421	1878431.817
2.996728801	0.005077949	6.29290126	506.7283609	0.121232565	5.460699865	0.288803204
0.045881857	0.060830394	1.121507667	37.59574032	0.842340423	994.735656	7.927859603
1.834601339	23.40729065	0.01153332	0.001577605	82.17921804	0.235283773	761.746319
1.435246096	0.019050387	1.926961808	67.24171692	0.166229381	5.783017396	27.19953855
0.189064125	31.25389488	0.017825268	0.218741389	0.064588327	0.024990864	512.289609
1.233624785	0.002468732	1.324569921	19.35224231	0.223754321	3.130594869	0.030139416
0.045881857	0.060830394	1.121507667	37.59574032	0.842340423	46.20629421	1878431.817
1.233624785	0.002468732	1.324569921	19.35224231	0.223754321	5.783017396	27.19953855
6.976323472	0.07789373	6.48504354	0.00650483	0.041251428	10.02766643	2.691111002
3.021486852	53.40437571	2.793263543	85.11011316	12.7755358	5.256930015	0.006286635
1.294216626	1.472267813	0.017485495	0.233785035	0.133971022	3.096116985	7490724.43
2.991096614	2.049696262	1.065523117	0.179610991	14.80615949	5.460699865	0.288803204
0.58449341	3.212285593	1.219583574	0.000101639	0.501844355	5.256930015	0.006286635
0.045881857	0.060830394	1.121507667	37.59574032	0.842340423	1.401537911	0.002591964
3.021486852	53.40437571	2.793263543	85.11011316	12.7755358	2.861748399	5.044338165
1.467858565	26.83914793	4.171133489	16.73814337	0.033727705	537.637133	0.502829598
1.467858565	26.83914793	4.171133489	16.73814337	0.033727705	1813.425348	119142.7764
1.233624785	0.002468732	1.324569921	19.35224231	0.223754321	994.735656	7.927859603
0.434237766	0.083081926	0.339648941	23.65470768	0.345081411	10.66908927	0.028558397
0.58449341	3.212285593	1.219583574	0.000101639	0.501844355	1.401537911	0.002591964
0.054042941	0.067966332	0.323370206	0.005361322	3.543836129	3.709490109	4.052575238
3.021486852	53.40437571	2.793263543	85.11011316	12.7755358	0.435715791	121.8315447
1.834601339	23.40729065	0.01153332	0.001577605	82.17921804	0.223448506	0.834984272
0.054042941	0.067966332	0.323370206	0.005361322	3.543836129	0.235283773	761.746319
0.10440408	0.106161149	0.220974484	13.00925642	4.61085615	3.709490109	4.052575238
3.021486852	53.40437571	2.793263543	85.11011316	12.7755358	994.735656	7.927859603
1.131873684	0.104495598	0.409746435	0.658133378	0.010463819	537.637133	0.502829598
2.996728801	0.005077949	6.29290126	506.7283609	0.121232565	89.45877868	159.4564655
1.233624785	0.002468732	1.324569921	19.35224231	0.223754321	994.735656	7.927859603
1.435246096	0.019050387	1.926961808	67.24171692	0.166229381	7.972298343	92.02553404
0.054042941	0.067966332	0.323370206	0.005361322	3.543836129	5.256930015	0.006286635
0.054042941	0.067966332	0.323370206	0.005361322	3.543836129	994.735656	7.927859603
0.045881857	0.060830394	1.121507667	37.59574032	0.842340423	994.735656	7.927859603
7.979498795	0.010492223	6.538933844	233.7877338	0.053036836	10.02766643	2.691111002

1.233624785	0.002468732	1.324569921	19.35224231	0.223754321	3.130594869	0.030139416
1.834601339	23.40729065	0.01153332	0.001577605	82.17921804	0.855106219	3030.198032
2.991096614	2.049696262	1.065523117	0.179610991	14.80615949	994.735656	7.927859603
3.021486852	53.40437571	2.793263543	85.11011316	12.7755358	46.20629421	1878431.817
0.58449341	3.212285593	1.219583574	0.000101639	0.501844355	0.235283773	761.746319
1.131873684	0.104495598	0.409746435	0.658133378	0.010463819	5.561172778	0.000704147
3.021486852	53.40437571	2.793263543	85.11011316	12.7755358	5.256930015	0.006286635
1.834601339	23.40729065	0.01153332	0.001577605	82.17921804	994.735656	7.927859603
0.054042941	0.067966332	0.323370206	0.005361322	3.543836129	46.20629421	1878431.817
1.435246096	0.019050387	1.926961808	67.24171692	0.166229381	1382.594644	0.305071512
0.10440408	0.106161149	0.220974484	13.00925642	4.61085615	994.735656	7.927859603
6.976323472	0.07789373	6.48504354	0.00650483	0.041251428	3.130594869	0.030139416
0.189064125	31.25389488	0.017825268	0.218741389	0.064588327	0.001671576	0.001066916
6.976323472	0.07789373	6.48504354	0.00650483	0.041251428	46.20629421	1878431.817
6.976323472	0.07789373	6.48504354	0.00650483	0.041251428	994.735656	7.927859603
1.467858565	26.83914793	4.171133489	16.73814337	0.033727705	994.735656	7.927859603
1.834601339	23.40729065	0.01153332	0.001577605	82.17921804	1382.594644	0.305071512
1.467858565	26.83914793	4.171133489	16.73814337	0.033727705	46.20629421	1878431.817
0.068248803	25.03655374	2.29750437	0.005371323	40.85597868	3.096116985	7490724.43
7.979498795	0.010492223	6.538933844	233.7877338	0.053036836	2.861748399	5.044338165
0.616126345	7.810474176	4.322315691	0.000859768	29.67646416	537.637133	0.502829598
2.996728801	0.005077949	6.29290126	506.7283609	0.121232565	994.735656	7.927859603
0.616126345	7.810474176	4.322315691	0.000859768	29.67646416	994.735656	7.927859603
7.979498795	0.010492223	6.538933844	233.7877338	0.053036836	5.460699865	0.288803204
1.606268382	1.31810948	2.161378439	5.246904741	1.315227066	1.401537911	0.002591964
0.054042941	0.067966332	0.323370206	0.005361322	3.543836129	0.148368246	0.032231981
0.58449341	3.212285593	1.219583574	0.000101639	0.501844355	5.783017396	27.19953855
0.189064125	31.25389488	0.017825268	0.218741389	0.064588327	0.038147525	12.2829238
2.996728801	0.005077949	6.29290126	506.7283609	0.121232565	46.20629421	1878431.817
0.054042941	0.067966332	0.323370206	0.005361322	3.543836129	5.256930015	0.006286635
0.58449341	3.212285593	1.219583574	0.000101639	0.501844355	3.709490109	4.052575238
1.435246096	0.019050387	1.926961808	67.24171692	0.166229381	3.130594869	0.030139416
146.2283384	108.9343921	159.5737034	14487.86218	138.0280438	0.417959285	7322.272056
0.58449341	3.212285593	1.219583574	0.000101639	0.501844355	994.735656	7.927859603
1.834601339	23.40729065	0.01153332	0.001577605	82.17921804	46.20629421	1878431.817
6.976323472	0.07789373	6.48504354	0.00650483	0.041251428	3.130594869	0.030139416
0.10440408	0.106161149	0.220974484	13.00925642	4.61085615	5.783017396	27.19953855
2.991096614	2.049696262	1.065523117	0.179610991	14.80615949	0.084945152	0.666990842
1.467858565	26.83914793	4.171133489	16.73814337	0.033727705	994.735656	7.927859603
7.979498795	0.010492223	6.538933844	233.7877338	0.053036836	994.735656	7.927859603
1.435246096	0.019050387	1.926961808	67.24171692	0.166229381	994.735656	7.927859603

1.606268382	1.31810948	2.161378439	5.246904741	1.315227066	0.235283773	761.746319
1.834601339	23.40729065	0.01153332	0.001577605	82.17921804	109.85648	0.961963177
0.58449341	3.212285593	1.219583574	0.000101639	0.501844355	3.130594869	0.030139416
0.054042941	0.067966332	0.323370206	0.005361322	3.543836129	994.735656	7.927859603
2.991096614	2.049696262	1.065523117	0.179610991	14.80615949	89.45877868	159.4564655
7.979498795	0.010492223	6.538933844	233.7877338	0.053036836	3.130594869	0.030139416
1.233624785	0.002468732	1.324569921	19.35224231	0.223754321	5.783017396	27.19953855
1.606268382	1.31810948	2.161378439	5.246904741	1.315227066	3.709490109	4.052575238
0.054042941	0.067966332	0.323370206	0.005361322	3.543836129	0.417959285	7322.272056
0.616126345	7.810474176	4.322315691	0.000859768	29.67646416	98.37328866	205.7148389
3.021486852	53.40437571	2.793263543	85.11011316	12.7755358	109.85648	0.961963177
7.979498795	0.010492223	6.538933844	233.7877338	0.053036836	109.85648	0.961963177
0.10440408	0.106161149	0.220974484	13.00925642	4.61085615	5.783017396	27.19953855
146.2283384	108.9343921	159.5737034	14487.86218	138.0280438	0.235283773	761.746319
1.606268382	1.31810948	2.161378439	5.246904741	1.315227066	994.735656	7.927859603
2.996728801	0.005077949	6.29290126	506.7283609	0.121232565	46.20629421	1878431.817
0.045881857	0.060830394	1.121507667	37.59574032	0.842340423	994.735656	7.927859603
3.021486852	53.40437571	2.793263543	85.11011316	12.7755358	46.20629421	1878431.817
3.021486852	53.40437571	2.793263543	85.11011316	12.7755358	3.130594869	0.030139416
0.054042941	0.067966332	0.323370206	0.005361322	3.543836129	5.561172778	0.000704147
0.054042941	0.067966332	0.323370206	0.005361322	3.543836129	3.096116985	7490724.43
0.434237766	0.083081926	0.339648941	23.65470768	0.345081411	100175.4644	0.151690343
2.996728801	0.005077949	6.29290126	506.7283609	0.121232565	46.20629421	1878431.817
1.834601339	23.40729065	0.01153332	0.001577605	82.17921804	46.20629421	1878431.817
146.2283384	108.9343921	159.5737034	14487.86218	138.0280438	109.85648	0.961963177
0.58449341	3.212285593	1.219583574	0.000101639	0.501844355	10.66908927	0.028558397
2.996728801	0.005077949	6.29290126	506.7283609	0.121232565	46.20629421	1878431.817
0.10440408	0.106161149	0.220974484	13.00925642	4.61085615	46.20629421	1878431.817
1.131873684	0.104495598	0.409746435	0.658133378	0.010463819	5.460699865	0.288803204
146.2283384	108.9343921	159.5737034	14487.86218	138.0280438	564.8652401	35.15101961
0.045881857	0.060830394	1.121507667	37.59574032	0.842340423	5.460699865	0.288803204
1.435246096	0.019050387	1.926961808	67.24171692	0.166229381	5.460699865	0.288803204
0.58449341	3.212285593	1.219583574	0.000101639	0.501844355	3.130594869	0.030139416
1.606268382	1.31810948	2.161378439	5.246904741	1.315227066	1.401537911	0.002591964
0.58449341	3.212285593	1.219583574	0.000101639	0.501844355	5.783017396	27.19953855
0.616126345	7.810474176	4.322315691	0.000859768	29.67646416	46.20629421	1878431.817
1.606268382	1.31810948	2.161378439	5.246904741	1.315227066	537.637133	0.502829598
1.606268382	1.31810948	2.161378439	5.246904741	1.315227066	89.45877868	159.4564655
1.435246096	0.019050387	1.926961808	67.24171692	0.166229381	3.709490109	4.052575238
1.233624785	0.002468732	1.324569921	19.35224231	0.223754321	46.20629421	1878431.817
0.189064125	31.25389488	0.017825268	0.218741389	0.064588327	994.735656	7.927859603

1.606268382	1.31810948	2.161378439	5.246904741	1.315227066	109.85648	0.961963177
0.10440408	0.106161149	0.220974484	13.00925642	4.61085615	46.20629421	1878431.817
1.606268382	1.31810948	2.161378439	5.246904741	1.315227066	1.401537911	0.002591964
1.834601339	23.40729065	0.01153332	0.001577605	82.17921804	0.084945152	0.666990842
1.606268382	1.31810948	2.161378439	5.246904741	1.315227066	3.130594869	0.030139416
1.294216626	1.472267813	0.017485495	0.233785035	0.133971022	0.235283773	761.746319
1.834601339	23.40729065	0.01153332	0.001577605	82.17921804	109.85648	0.961963177
1.131873684	0.104495598	0.409746435	0.658133378	0.010463819	89.45877868	159.4564655
7.979498795	0.010492223	6.538933844	233.7877338	0.053036836	10.66908927	0.028558397
0.068248803	25.03655374	2.29750437	0.005371323	40.85597868	10.66908927	0.028558397
2.996728801	0.005077949	6.29290126	506.7283609	0.121232565	5.561172778	0.000704147
6.976323472	0.07789373	6.48504354	0.00650483	0.041251428	5.460699865	0.288803204
1.294216626	1.472267813	0.017485495	0.233785035	0.133971022	3.130594869	0.030139416
1.467858565	26.83914793	4.171133489	16.73814337	0.033727705	564.8652401	35.15101961
0.616126345	7.810474176	4.322315691	0.000859768	29.67646416	5.783017396	27.19953855
1.294216626	1.472267813	0.017485495	0.233785035	0.133971022	7.972298343	92.02553404
2.996728801	0.005077949	6.29290126	506.7283609	0.121232565	5.783017396	27.19953855
1.467858565	26.83914793	4.171133489	16.73814337	0.033727705	3.709490109	4.052575238
0.616126345	7.810474176	4.322315691	0.000859768	29.67646416	994.735656	7.927859603
3.021486852	53.40437571	2.793263543	85.11011316	12.7755358	89.45877868	159.4564655
1.294216626	1.472267813	0.017485495	0.233785035	0.133971022	1813.425348	119142.7764
6.976323472	0.07789373	6.48504354	0.00650483	0.041251428	3.130594869	0.030139416
0.068248803	25.03655374	2.29750437	0.005371323	40.85597868	89.45877868	159.4564655
1.131873684	0.104495598	0.409746435	0.658133378	0.010463819	2.861748399	5.044338165
3.021486852	53.40437571	2.793263543	85.11011316	12.7755358	994.735656	7.927859603
6.976323472	0.07789373	6.48504354	0.00650483	0.041251428	0.235283773	761.746319
0.189064125	31.25389488	0.017825268	0.218741389	0.064588327	1382.594644	0.305071512
6.976323472	0.07789373	6.48504354	0.00650483	0.041251428	46.20629421	1878431.817
1.834601339	23.40729065	0.01153332	0.001577605	82.17921804	3.709490109	4.052575238
2.991096614	2.049696262	1.065523117	0.179610991	14.80615949	1203.152028	8852.522525
1.834601339	23.40729065	0.01153332	0.001577605	82.17921804	5.460699865	0.288803204
1.435246096	0.019050387	1.926961808	67.24171692	0.166229381	5.256930015	0.006286635
0.58449341	3.212285593	1.219583574	0.000101639	0.501844355	7.972298343	92.02553404
1.467858565	26.83914793	4.171133489	16.73814337	0.033727705	3.130594869	0.030139416
0.616126345	7.810474176	4.322315691	0.000859768	29.67646416	0.024990864	512.289609
1.131873684	0.104495598	0.409746435	0.658133378	0.010463819	3.709490109	4.052575238
6.976323472	0.07789373	6.48504354	0.00650483	0.041251428	46.20629421	1878431.817
0.068248803	25.03655374	2.29750437	0.005371323	40.85597868	994.735656	7.927859603
0.10440408	0.106161149	0.220974484	13.00925642	4.61085615	3.709490109	4.052575238
1.131873684	0.104495598	0.409746435	0.658133378	0.010463819	994.735656	7.927859603
0.054042941	0.067966332	0.323370206	0.005361322	3.543836129	5.561172778	0.000704147

1.606268382	1.31810948	2.161378439	5.246904741	1.315227066	3.096116985	7490724.43
2.991096614	2.049696262	1.065523117	0.179610991	14.80615949	5.783017396	27.19953855
0.616126345	7.810474176	4.322315691	0.000859768	29.67646416	564.8652401	35.15101961
0.045881857	0.060830394	1.121507667	37.59574032	0.842340423	3.709490109	4.052575238
0.434237766	0.083081926	0.339648941	23.65470768	0.345081411	0.235283773	761.746319
0.189064125	31.25389488	0.017825268	0.218741389	0.064588327	109.85648	0.961963177
0.054042941	0.067966332	0.323370206	0.005361322	3.543836129	994.735656	7.927859603
3.021486852	53.40437571	2.793263543	85.11011316	12.7755358	3.130594869	0.030139416
7.979498795	0.010492223	6.538933844	233.7877338	0.053036836	3.130594869	0.030139416
0.189064125	31.25389488	0.017825268	0.218741389	0.064588327	0.855106219	3030.198032
2.991096614	2.049696262	1.065523117	0.179610991	14.80615949	0.435715791	121.8315447
0.189064125	31.25389488	0.017825268	0.218741389	0.064588327	3.709490109	4.052575238
2.996728801	0.005077949	6.29290126	506.7283609	0.121232565	994.735656	7.927859603
0.434237766	0.083081926	0.339648941	23.65470768	0.345081411	46.20629421	1878431.817
7.979498795	0.010492223	6.538933844	233.7877338	0.053036836	3.130594869	0.030139416
0.068248803	25.03655374	2.29750437	0.005371323	40.85597868	1.401537911	0.002591964
1.294216626	1.472267813	0.017485495	0.233785035	0.133971022	0.235283773	761.746319
1.294216626	1.472267813	0.017485495	0.233785035	0.133971022	3.130594869	0.030139416
1.834601339	23.40729065	0.01153332	0.001577605	82.17921804	0.855106219	3030.198032
146.2283384	108.9343921	159.5737034	14487.86218	138.0280438	5.256930015	0.006286635
1.606268382	1.31810948	2.161378439	5.246904741	1.315227066	3.130594869	0.030139416
2.991096614	2.049696262	1.065523117	0.179610991	14.80615949	5.783017396	27.19953855
1.834601339	23.40729065	0.01153332	0.001577605	82.17921804	3.709490109	4.052575238
1.467858565	26.83914793	4.171133489	16.73814337	0.033727705	3.709490109	4.052575238
0.10440408	0.106161149	0.220974484	13.00925642	4.61085615	0.024990864	512.289609
0.068248803	25.03655374	2.29750437	0.005371323	40.85597868	564.8652401	35.15101961
2.996728801	0.005077949	6.29290126	506.7283609	0.121232565	564.8652401	35.15101961
1.435246096	0.019050387	1.926961808	67.24171692	0.166229381	3.709490109	4.052575238
2.991096614	2.049696262	1.065523117	0.179610991	14.80615949	5.461427683	1077080.988
1.467858565	26.83914793	4.171133489	16.73814337	0.033727705	5.561172778	0.000704147
7.979498795	0.010492223	6.538933844	233.7877338	0.053036836	994.735656	7.927859603
0.434237766	0.083081926	0.339648941	23.65470768	0.345081411	89.45877868	159.4564655
146.2283384	108.9343921	159.5737034	14487.86218	138.0280438	0.148368246	0.032231981
0.616126345	7.810474176	4.322315691	0.000859768	29.67646416	0.223448506	0.834984272
0.10440408	0.106161149	0.220974484	13.00925642	4.61085615	0.855106219	3030.198032
1.131873684	0.104495598	0.409746435	0.658133378	0.010463819	0.148368246	0.032231981
1.294216626	1.472267813	0.017485495	0.233785035	0.133971022	3.130594869	0.030139416
0.10440408	0.106161149	0.220974484	13.00925642	4.61085615	0.038147525	12.2829238
1.467858565	26.83914793	4.171133489	16.73814337	0.033727705	994.735656	7.927859603
7.979498795	0.010492223	6.538933844	233.7877338	0.053036836	0.038147525	12.2829238
1.294216626	1.472267813	0.017485495	0.233785035	0.133971022	994.735656	7.927859603

0.434237766	0.083081926	0.339648941	23.65470768	0.345081411		3.130594869	0.030139416
2.991096614	2.049696262	1.065523117	0.179610991	14.80615949		3.130594869	0.030139416
2.991096614	2.049696262	1.065523117	0.179610991	14.80615949		994.735656	7.927859603
0.045881857	0.060830394	1.121507667	37.59574032	0.842340423		3.130594869	0.030139416
1.467858565	26.83914793	4.171133489	16.73814337	0.033727705		7.972298343	92.02553404
1.435246096	0.019050387	1.926961808	67.24171692	0.166229381		994.735656	7.927859603
1.294216626	1.472267813	0.017485495	0.233785035	0.133971022		994.735656	7.927859603
6.976323472	0.07789373	6.48504354	0.00650483	0.041251428		100175.4644	0.151690343
1.467858565	26.83914793	4.171133489	16.73814337	0.033727705		98.37328866	205.7148389
0.068248803	25.03655374	2.29750437	0.005371323	40.85597868		0.001671576	0.001066916
2.991096614	2.049696262	1.065523117	0.179610991	14.80615949		0.435715791	121.8315447
6.976323472	0.07789373	6.48504354	0.00650483	0.041251428		5.256930015	0.006286635
0.434237766	0.083081926	0.339648941	23.65470768	0.345081411		0.350754565	0.29243781
0.434237766	0.083081926	0.339648941	23.65470768	0.345081411		1813.425348	119142.7764
1.233624785	0.002468732	1.324569921	19.35224231	0.223754321		5.461427683	1077080.988
1.467858565	26.83914793	4.171133489	16.73814337	0.033727705		3.709490109	4.052575238
0.616126345	7.810474176	4.322315691	0.000859768	29.67646416		5.783017396	27.19953855
0.068248803	25.03655374	2.29750437	0.005371323	40.85597868		3.130594869	0.030139416
3.021486852	53.40437571	2.793263543	85.11011316	12.7755358		89.45877868	159.4564655
2.991096614	2.049696262	1.065523117	0.179610991	14.80615949		10.66908927	0.028558397
7.979498795	0.010492223	6.538933844	233.7877338	0.053036836		3.130594869	0.030139416
1.131873684	0.104495598	0.409746435	0.658133378	0.010463819		7.972298343	92.02553404
1.233624785	0.002468732	1.324569921	19.35224231	0.223754321		5.460699865	0.288803204
0.434237766	0.083081926	0.339648941	23.65470768	0.345081411		3.130594869	0.030139416
1.233624785	0.002468732	1.324569921	19.35224231	0.223754321		994.735656	7.927859603
0.045881857	0.060830394	1.121507667	37.59574032	0.842340423		10.66908927	0.028558397
1.233624785	0.002468732	1.324569921	19.35224231	0.223754321		46.20629421	1878431.817
1.233624785	0.002468732	1.324569921	19.35224231	0.223754321		5.461427683	1077080.988
1.834601339	23.40729065	0.01153332	0.001577605	82.17921804		0.350754565	0.29243781
1.834601339	23.40729065	0.01153332	0.001577605	82.17921804		89.45877868	159.4564655
1.233624785	0.002468732	1.324569921	19.35224231	0.223754321		3.709490109	4.052575238
0.054042941	0.067966332	0.323370206	0.005361322	3.543836129		5.460699865	0.288803204
0.189064125	31.25389488	0.017825268	0.218741389	0.064588327		0.235283773	761.746319
1.467858565	26.83914793	4.171133489	16.73814337	0.033727705		7.972298343	92.02553404
1.435246096	0.019050387	1.926961808	67.24171692	0.166229381		5.783017396	27.19953855
1.233624785	0.002468732	1.324569921	19.35224231	0.223754321		3.130594869	0.030139416
0.58449341	3.212285593	1.219583574	0.000101639	0.501844355		3.709490109	4.052575238
0.189064125	31.25389488	0.017825268	0.218741389	0.064588327		5.256930015	0.006286635
2.991096614	2.049696262	1.065523117	0.179610991	14.80615949		5.460699865	0.288803204
1.606268382	1.31810948	2.161378439	5.246904741	1.315227066		46.20629421	1878431.817
1.834601339	23.40729065	0.01153332	0.001577605	82.17921804		5.561172778	0.000704147

146.2283384	108.9343921	159.5737034	14487.86218	138.0280438	1.401537911	0.002591964
146.2283384	108.9343921	159.5737034	14487.86218	138.0280438	994.735656	7.927859603
0.045881857	0.060830394	1.121507667	37.59574032	0.842340423	994.735656	7.927859603
3.021486852	53.40437571	2.793263543	85.11011316	12.7755358	564.8652401	35.15101961
0.58449341	3.212285593	1.219583574	0.000101639	0.501844355	46.20629421	1878431.817
1.834601339	23.40729065	0.01153332	0.001577605	82.17921804	0.024990864	512.289609
0.58449341	3.212285593	1.219583574	0.000101639	0.501844355	3.130594869	0.030139416
6.976323472	0.07789373	6.48504354	0.00650483	0.041251428	3.130594869	0.030139416
1.834601339	23.40729065	0.01153332	0.001577605	82.17921804	537.637133	0.502829598
1.435246096	0.019050387	1.926961808	67.24171692	0.166229381	994.735656	7.927859603
0.616126345	7.810474176	4.322315691	0.000859768	29.67646416	3.130594869	0.030139416
1.435246096	0.019050387	1.926961808	67.24171692	0.166229381	3.709490109	4.052575238
2.996728801	0.005077949	6.29290126	506.7283609	0.121232565	89.45877868	159.4564655
1.131873684	0.104495598	0.409746435	0.658133378	0.010463819	5.460699865	0.288803204
7.979498795	0.010492223	6.538933844	233.7877338	0.053036836	3.130594869	0.030139416
0.189064125	31.25389488	0.017825268	0.218741389	0.064588327	46.20629421	1878431.817
0.045881857	0.060830394	1.121507667	37.59574032	0.842340423	3.130594869	0.030139416
1.606268382	1.31810948	2.161378439	5.246904741	1.315227066	5.460699865	0.288803204
6.976323472	0.07789373	6.48504354	0.00650483	0.041251428	0.038147525	12.2829238
6.976323472	0.07789373	6.48504354	0.00650483	0.041251428	3.709490109	4.052575238
1.233624785	0.002468732	1.324569921	19.35224231	0.223754321	89.45877868	159.4564655
0.189064125	31.25389488	0.017825268	0.218741389	0.064588327	100175.4644	0.151690343
1.131873684	0.104495598	0.409746435	0.658133378	0.010463819	5.783017396	27.19953855
0.054042941	0.067966332	0.323370206	0.005361322	3.543836129	46.20629421	1878431.817
7.979498795	0.010492223	6.538933844	233.7877338	0.053036836	5.460699865	0.288803204
1.435246096	0.019050387	1.926961808	67.24171692	0.166229381	204.3719434	2827259.242
0.068248803	25.03655374	2.29750437	0.005371323	40.85597868	10.66908927	0.028558397
146.2283384	108.9343921	159.5737034	14487.86218	138.0280438	3.130594869	0.030139416
3.021486852	53.40437571	2.793263543	85.11011316	12.7755358	46.20629421	1878431.817
1.294216626	1.472267813	0.017485495	0.233785035	0.133971022	109.85648	0.961963177
2.996728801	0.005077949	6.29290126	506.7283609	0.121232565	994.735656	7.927859603
6.976323472	0.07789373	6.48504354	0.00650483	0.041251428	3.130594869	0.030139416
3.021486852	53.40437571	2.793263543	85.11011316	12.7755358	98.37328866	205.7148389
1.131873684	0.104495598	0.409746435	0.658133378	0.010463819	3.130594869	0.030139416
0.045881857	0.060830394	1.121507667	37.59574032	0.842340423	0.235283773	761.746319
0.434237766	0.083081926	0.339648941	23.65470768	0.345081411	5.460699865	0.288803204
6.976323472	0.07789373	6.48504354	0.00650483	0.041251428	0.001671576	0.001066916
0.58449341	3.212285593	1.219583574	0.000101639	0.501844355	5.561172778	0.000704147
1.467858565	26.83914793	4.171133489	16.73814337	0.033727705	564.8652401	35.15101961
146.2283384	108.9343921	159.5737034	14487.86218	138.0280438	5.561172778	0.000704147
7.979498795	0.010492223	6.538933844	233.7877338	0.053036836	1382.594644	0.305071512

7.979498795	0.010492223	6.538933844	233.7877338	0.053036836	46.20629421	1878431.817
2.991096614	2.049696262	1.065523117	0.179610991	14.80615949	5.460699865	0.288803204
0.58449341	3.212285593	1.219583574	0.000101639	0.501844355	5.561172778	0.000704147
0.189064125	31.25389488	0.017825268	0.218741389	0.064588327	109.85648	0.961963177
0.434237766	0.083081926	0.339648941	23.65470768	0.345081411	46.20629421	1878431.817
0.189064125	31.25389488	0.017825268	0.218741389	0.064588327	3.130594869	0.030139416
1.294216626	1.472267813	0.017485495	0.233785035	0.133971022	1813.425348	119142.7764
1.233624785	0.002468732	1.324569921	19.35224231	0.223754321	0.235283773	761.746319
1.467858565	26.83914793	4.171133489	16.73814337	0.033727705	109.85648	0.961963177
1.467858565	26.83914793	4.171133489	16.73814337	0.033727705	994.735656	7.927859603
0.434237766	0.083081926	0.339648941	23.65470768	0.345081411	0.235283773	761.746319
1.606268382	1.31810948	2.161378439	5.246904741	1.315227066	0.235283773	761.746319
0.045881857	0.060830394	1.121507667	37.59574032	0.842340423	3.709490109	4.052575238
7.979498795	0.010492223	6.538933844	233.7877338	0.053036836	1.064090239	0.001329755
0.10440408	0.106161149	0.220974484	13.00925642	4.61085615	3.130594869	0.030139416
0.10440408	0.106161149	0.220974484	13.00925642	4.61085615	537.637133	0.502829598
0.054042941	0.067966332	0.323370206	0.005361322	3.543836129	3.130594869	0.030139416
6.976323472	0.07789373	6.48504354	0.00650483	0.041251428	3.130594869	0.030139416
0.054042941	0.067966332	0.323370206	0.005361322	3.543836129	994.735656	7.927859603
0.189064125	31.25389488	0.017825268	0.218741389	0.064588327	3.130594869	0.030139416
0.045881857	0.060830394	1.121507667	37.59574032	0.842340423	3.096116985	7490724.43
0.068248803	25.03655374	2.29750437	0.005371323	40.85597868	1813.425348	119142.7764
0.58449341	3.212285593	1.219583574	0.000101639	0.501844355	994.735656	7.927859603
1.467858565	26.83914793	4.171133489	16.73814337	0.033727705	89.45877868	159.4564655
2.996728801	0.005077949	6.29290126	506.7283609	0.121232565	2.861748399	5.044338165
6.976323472	0.07789373	6.48504354	0.00650483	0.041251428	46.20629421	1878431.817
2.991096614	2.049696262	1.065523117	0.179610991	14.80615949	5.783017396	27.19953855
1.233624785	0.002468732	1.324569921	19.35224231	0.223754321	3.130594869	0.030139416
1.606268382	1.31810948	2.161378439	5.246904741	1.315227066	994.735656	7.927859603
3.021486852	53.40437571	2.793263543	85.11011316	12.7755358	109.85648	0.961963177
0.054042941	0.067966332	0.323370206	0.005361322	3.543836129	994.735656	7.927859603
0.58449341	3.212285593	1.219583574	0.000101639	0.501844355	1.064090239	0.001329755
7.979498795	0.010492223	6.538933844	233.7877338	0.053036836	10.02766643	2.691111002
0.054042941	0.067966332	0.323370206	0.005361322	3.543836129	3.130594869	0.030139416
0.616126345	7.810474176	4.322315691	0.000859768	29.67646416	46.20629421	1878431.817
146.2283384	108.9343921	159.5737034	14487.86218	138.0280438	46.20629421	1878431.817
1.435246096	0.019050387	1.926961808	67.24171692	0.166229381	5.783017396	27.19953855
0.434237766	0.083081926	0.339648941	23.65470768	0.345081411	89.45877868	159.4564655
6.976323472	0.07789373	6.48504354	0.00650483	0.041251428	3.130594869	0.030139416
0.189064125	31.25389488	0.017825268	0.218741389	0.064588327	537.637133	0.502829598
0.068248803	25.03655374	2.29750437	0.005371323	40.85597868	994.735656	7.927859603

0.189064125	31.25389488	0.017825268	0.218741389	0.064588327	46.20629421	1878431.817
2.996728801	0.005077949	6.29290126	506.7283609	0.121232565	5.460699865	0.288803204
1.131873684	0.104495598	0.409746435	0.658133378	0.010463819	564.8652401	35.15101961
0.58449341	3.212285593	1.219583574	0.000101639	0.501844355	0.235283773	761.746319
1.606268382	1.31810948	2.161378439	5.246904741	1.315227066	46.20629421	1878431.817
2.991096614	2.049696262	1.065523117	0.179610991	14.80615949	5.783017396	27.19953855
1.131873684	0.104495598	0.409746435	0.658133378	0.010463819	537.637133	0.502829598
1.294216626	1.472267813	0.017485495	0.233785035	0.133971022	3.130594869	0.030139416
146.2283384	108.9343921	159.5737034	14487.86218	138.0280438	109.85648	0.961963177
1.606268382	1.31810948	2.161378439	5.246904741	1.315227066	46.20629421	1878431.817
1.834601339	23.40729065	0.01153332	0.001577605	82.17921804	3.130594869	0.030139416
1.467858565	26.83914793	4.171133489	16.73814337	0.033727705	3.130594869	0.030139416
6.976323472	0.07789373	6.48504354	0.00650483	0.041251428	994.735656	7.927859603
146.2283384	108.9343921	159.5737034	14487.86218	138.0280438	46.20629421	1878431.817
0.10440408	0.106161149	0.220974484	13.00925642	4.61085615	5.460699865	0.288803204
1.435246096	0.019050387	1.926961808	67.24171692	0.166229381	3.130594869	0.030139416
2.991096614	2.049696262	1.065523117	0.179610991	14.80615949	5.460699865	0.288803204
0.434237766	0.083081926	0.339648941	23.65470768	0.345081411	109.85648	0.961963177
1.233624785	0.002468732	1.324569921	19.35224231	0.223754321	7.972298343	92.02553404
1.606268382	1.31810948	2.161378439	5.246904741	1.315227066	109.85648	0.961963177
0.045881857	0.060830394	1.121507667	37.59574032	0.842340423	46.20629421	1878431.817
0.616126345	7.810474176	4.322315691	0.000859768	29.67646416	109.85648	0.961963177
0.189064125	31.25389488	0.017825268	0.218741389	0.064588327	994.735656	7.927859603
1.294216626	1.472267813	0.017485495	0.233785035	0.133971022	10.02766643	2.691111002
1.435246096	0.019050387	1.926961808	67.24171692	0.166229381	0.038147525	12.2829238
0.434237766	0.083081926	0.339648941	23.65470768	0.345081411	3.130594869	0.030139416
0.434237766	0.083081926	0.339648941	23.65470768	0.345081411	3.709490109	4.052575238
0.054042941	0.067966332	0.323370206	0.005361322	3.543836129	5.460699865	0.288803204
7.979498795	0.010492223	6.538933844	233.7877338	0.053036836	564.8652401	35.15101961
6.976323472	0.07789373	6.48504354	0.00650483	0.041251428	5.460699865	0.288803204
0.068248803	25.03655374	2.29750437	0.005371323	40.85597868	46.20629421	1878431.817
2.996728801	0.005077949	6.29290126	506.7283609	0.121232565	0.235283773	761.746319
0.054042941	0.067966332	0.323370206	0.005361322	3.543836129	10.02766643	2.691111002
0.58449341	3.212285593	1.219583574	0.000101639	0.501844355	564.8652401	35.15101961
1.834601339	23.40729065	0.01153332	0.001577605	82.17921804	5.783017396	27.19953855
2.996728801	0.005077949	6.29290126	506.7283609	0.121232565	3.709490109	4.052575238
0.10440408	0.106161149	0.220974484	13.00925642	4.61085615	5.783017396	27.19953855
146.2283384	108.9343921	159.5737034	14487.86218	138.0280438	46.20629421	1878431.817
0.10440408	0.106161149	0.220974484	13.00925642	4.61085615	1382.594644	0.305071512
1.435246096	0.019050387	1.926961808	67.24171692	0.166229381	3.130594869	0.030139416
1.606268382	1.31810948	2.161378439	5.246904741	1.315227066	46.20629421	1878431.817

1.131873684	0.104495598	0.409746435	0.658133378	0.010463819	3.709490109	4.052575238
146.2283384	108.9343921	159.5737034	14487.86218	138.0280438	3.130594869	0.030139416
146.2283384	108.9343921	159.5737034	14487.86218	138.0280438	994.735656	7.927859603
0.045881857	0.060830394	1.121507667	37.59574032	0.842340423	2.861748399	5.044338165
2.991096614	2.049696262	1.065523117	0.179610991	14.80615949	994.735656	7.927859603
2.996728801	0.005077949	6.29290126	506.7283609	0.121232565	994.735656	7.927859603
7.979498795	0.010492223	6.538933844	233.7877338	0.053036836	3.130594869	0.030139416
0.58449341	3.212285593	1.219583574	0.000101639	0.501844355	3.709490109	4.052575238
1.131873684	0.104495598	0.409746435	0.658133378	0.010463819	5.783017396	27.19953855
0.58449341	3.212285593	1.219583574	0.000101639	0.501844355	46.20629421	1878431.817
0.616126345	7.810474176	4.322315691	0.000859768	29.67646416	3.130594869	0.030139416
146.2283384	108.9343921	159.5737034	14487.86218	138.0280438	46.20629421	1878431.817
1.606268382	1.31810948	2.161378439	5.246904741	1.315227066	1.064090239	0.001329755
0.068248803	25.03655374	2.29750437	0.005371323	40.85597868	994.735656	7.927859603
2.996728801	0.005077949	6.29290126	506.7283609	0.121232565	994.735656	7.927859603
0.189064125	31.25389488	0.017825268	0.218741389	0.064588327	564.8652401	35.15101961
0.189064125	31.25389488	0.017825268	0.218741389	0.064588327	1.401537911	0.002591964
0.054042941	0.067966332	0.323370206	0.005361322	3.543836129	5.783017396	27.19953855
0.434237766	0.083081926	0.339648941	23.65470768	0.345081411	5.460699865	0.288803204
1.834601339	23.40729065	0.01153332	0.001577605	82.17921804	0.435715791	121.8315447
3.021486852	53.40437571	2.793263543	85.11011316	12.7755358	46.20629421	1878431.817
1.233624785	0.002468732	1.324569921	19.35224231	0.223754321	1382.594644	0.305071512
7.979498795	0.010492223	6.538933844	233.7877338	0.053036836	0.235283773	761.746319
1.606268382	1.31810948	2.161378439	5.246904741	1.315227066	5.256930015	0.006286635
1.233624785	0.002468732	1.324569921	19.35224231	0.223754321	109.85648	0.961963177
0.189064125	31.25389488	0.017825268	0.218741389	0.064588327	2.861748399	5.044338165
3.021486852	53.40437571	2.793263543	85.11011316	12.7755358	204.3719434	2827259.242
0.434237766	0.083081926	0.339648941	23.65470768	0.345081411	10.66908927	0.028558397
1.467858565	26.83914793	4.171133489	16.73814337	0.033727705	0.350754565	0.29243781
2.991096614	2.049696262	1.065523117	0.179610991	14.80615949	46.20629421	1878431.817
2.996728801	0.005077949	6.29290126	506.7283609	0.121232565	5.783017396	27.19953855
0.616126345	7.810474176	4.322315691	0.000859768	29.67646416	3.709490109	4.052575238
1.606268382	1.31810948	2.161378439	5.246904741	1.315227066	1203.152028	8852.522525
1.467858565	26.83914793	4.171133489	16.73814337	0.033727705	994.735656	7.927859603
146.2283384	108.9343921	159.5737034	14487.86218	138.0280438	3.130594869	0.030139416
3.021486852	53.40437571	2.793263543	85.11011316	12.7755358	0.350754565	0.29243781
0.58449341	3.212285593	1.219583574	0.000101639	0.501844355	5.256930015	0.006286635
7.979498795	0.010492223	6.538933844	233.7877338	0.053036836	994.735656	7.927859603
0.434237766	0.083081926	0.339648941	23.65470768	0.345081411	994.735656	7.927859603
0.068248803	25.03655374	2.29750437	0.005371323	40.85597868	0.435715791	121.8315447
1.294216626	1.472267813	0.017485495	0.233785035	0.133971022	5.561172778	0.000704147

1.131873684	0.104495598	0.409746435	0.658133378	0.010463819	537.637133	0.502829598
1.606268382	1.31810948	2.161378439	5.246904741	1.315227066	994.735656	7.927859603
0.054042941	0.067966332	0.323370206	0.005361322	3.543836129	5.461427683	1077080.988
146.2283384	108.9343921	159.5737034	14487.86218	138.0280438	994.735656	7.927859603
1.435246096	0.019050387	1.926961808	67.24171692	0.166229381	3.130594869	0.030139416
1.606268382	1.31810948	2.161378439	5.246904741	1.315227066	5.561172778	0.000704147
0.434237766	0.083081926	0.339648941	23.65470768	0.345081411	3.709490109	4.052575238
3.021486852	53.40437571	2.793263543	85.11011316	12.7755358	994.735656	7.927859603
146.2283384	108.9343921	159.5737034	14487.86218	138.0280438	5.561172778	0.000704147
0.054042941	0.067966332	0.323370206	0.005361322	3.543836129	0.235283773	761.746319
0.10440408	0.106161149	0.220974484	13.00925642	4.61085615	994.735656	7.927859603
1.467858565	26.83914793	4.171133489	16.73814337	0.033727705	994.735656	7.927859603
1.834601339	23.40729065	0.01153332	0.001577605	82.17921804	0.038147525	12.2829238
2.991096614	2.049696262	1.065523117	0.179610991	14.80615949	46.20629421	1878431.817
1.233624785	0.002468732	1.324569921	19.35224231	0.223754321	537.637133	0.502829598
6.976323472	0.07789373	6.48504354	0.00650483	0.041251428	46.20629421	1878431.817
3.021486852	53.40437571	2.793263543	85.11011316	12.7755358	994.735656	7.927859603
2.991096614	2.049696262	1.065523117	0.179610991	14.80615949	3.709490109	4.052575238
1.131873684	0.104495598	0.409746435	0.658133378	0.010463819	1382.594644	0.305071512
0.054042941	0.067966332	0.323370206	0.005361322	3.543836129	3.130594869	0.030139416
1.467858565	26.83914793	4.171133489	16.73814337	0.033727705	2.861748399	5.044338165
0.58449341	3.212285593	1.219583574	0.000101639	0.501844355	564.8652401	35.15101961
2.996728801	0.005077949	6.29290126	506.7283609	0.121232565	46.20629421	1878431.817
0.045881857	0.060830394	1.121507667	37.59574032	0.842340423	3.709490109	4.052575238
0.045881857	0.060830394	1.121507667	37.59574032	0.842340423	994.735656	7.927859603
0.58449341	3.212285593	1.219583574	0.000101639	0.501844355	994.735656	7.927859603
0.616126345	7.810474176	4.322315691	0.000859768	29.67646416	3.130594869	0.030139416
1.606268382	1.31810948	2.161378439	5.246904741	1.315227066	3.130594869	0.030139416
0.054042941	0.067966332	0.323370206	0.005361322	3.543836129	3.130594869	0.030139416
1.294216626	1.472267813	0.017485495	0.233785035	0.133971022	109.85648	0.961963177
1.131873684	0.104495598	0.409746435	0.658133378	0.010463819	3.130594869	0.030139416
3.021486852	53.40437571	2.793263543	85.11011316	12.7755358	564.8652401	35.15101961
0.434237766	0.083081926	0.339648941	23.65470768	0.345081411	994.735656	7.927859603
1.834601339	23.40729065	0.01153332	0.001577605	82.17921804	994.735656	7.927859603
1.467858565	26.83914793	4.171133489	16.73814337	0.033727705	2.861748399	5.044338165
7.979498795	0.010492223	6.538933844	233.7877338	0.053036836	5.256930015	0.006286635
0.068248803	25.03655374	2.29750437	0.005371323	40.85597868	10.66908927	0.028558397
0.10440408	0.106161149	0.220974484	13.00925642	4.61085615	0.148368246	0.032231981
1.834601339	23.40729065	0.01153332	0.001577605	82.17921804	1813.425348	119142.7764
146.2283384	108.9343921	159.5737034	14487.86218	138.0280438	5.561172778	0.000704147
1.834601339	23.40729065	0.01153332	0.001577605	82.17921804	3.130594869	0.030139416

3.021486852	53.40437571	2.793263543	85.11011316	12.7755358	564.8652401	35.15101961
1.233624785	0.002468732	1.324569921	19.35224231	0.223754321	0.235283773	761.746319
0.58449341	3.212285593	1.219583574	0.000101639	0.501844355	0.235283773	761.746319
0.434237766	0.083081926	0.339648941	23.65470768	0.345081411	0.235283773	761.746319
1.435246096	0.019050387	1.926961808	67.24171692	0.166229381	994.735656	7.927859603
1.294216626	1.472267813	0.017485495	0.233785035	0.133971022	3.130594869	0.030139416
0.045881857	0.060830394	1.121507667	37.59574032	0.842340423	994.735656	7.927859603
2.991096614	2.049696262	1.065523117	0.179610991	14.80615949	46.20629421	1878431.817
1.467858565	26.83914793	4.171133489	16.73814337	0.033727705	5.561172778	0.000704147
1.606268382	1.31810948	2.161378439	5.246904741	1.315227066	46.20629421	1878431.817
0.054042941	0.067966332	0.323370206	0.005361322	3.543836129	1813.425348	119142.7764
146.2283384	108.9343921	159.5737034	14487.86218	138.0280438	3.130594869	0.030139416
1.467858565	26.83914793	4.171133489	16.73814337	0.033727705	994.735656	7.927859603
146.2283384	108.9343921	159.5737034	14487.86218	138.0280438	5.460699865	0.288803204
146.2283384	108.9343921	159.5737034	14487.86218	138.0280438	46.20629421	1878431.817
6.976323472	0.07789373	6.48504354	0.00650483	0.041251428	0.855106219	3030.198032
7.979498795	0.010492223	6.538933844	233.7877338	0.053036836	3.130594869	0.030139416
1.467858565	26.83914793	4.171133489	16.73814337	0.033727705	3.130594869	0.030139416
1.834601339	23.40729065	0.01153332	0.001577605	82.17921804	5.460699865	0.288803204
0.10440408	0.106161149	0.220974484	13.00925642	4.61085615	1382.594644	0.305071512
146.2283384	108.9343921	159.5737034	14487.86218	138.0280438	3.130594869	0.030139416
0.189064125	31.25389488	0.017825268	0.218741389	0.064588327	1203.152028	8852.522525
0.045881857	0.060830394	1.121507667	37.59574032	0.842340423	46.20629421	1878431.817
6.976323472	0.07789373	6.48504354	0.00650483	0.041251428	5.783017396	27.19953855
0.616126345	7.810474176	4.322315691	0.000859768	29.67646416	5.460699865	0.288803204
0.054042941	0.067966332	0.323370206	0.005361322	3.543836129	0.038147525	12.2829238
1.606268382	1.31810948	2.161378439	5.246904741	1.315227066	3.130594869	0.030139416
6.976323472	0.07789373	6.48504354	0.00650483	0.041251428	7.972298343	92.02553404
7.979498795	0.010492223	6.538933844	233.7877338	0.053036836	1.401537911	0.002591964
1.233624785	0.002468732	1.324569921	19.35224231	0.223754321	46.20629421	1878431.817
0.58449341	3.212285593	1.219583574	0.000101639	0.501844355	5.561172778	0.000704147
1.233624785	0.002468732	1.324569921	19.35224231	0.223754321	0.235283773	761.746319
0.189064125	31.25389488	0.017825268	0.218741389	0.064588327	46.20629421	1878431.817
0.054042941	0.067966332	0.323370206	0.005361322	3.543836129	3.130594869	0.030139416
0.068248803	25.03655374	2.29750437	0.005371323	40.85597868	0.024990864	512.289609
6.976323472	0.07789373	6.48504354	0.00650483	0.041251428	994.735656	7.927859603
6.976323472	0.07789373	6.48504354	0.00650483	0.041251428	537.637133	0.502829598
0.58449341	3.212285593	1.219583574	0.000101639	0.501844355	0.001671576	0.001066916
0.068248803	25.03655374	2.29750437	0.005371323	40.85597868	3.130594869	0.030139416
1.435246096	0.019050387	1.926961808	67.24171692	0.166229381	994.735656	7.927859603
0.434237766	0.083081926	0.339648941	23.65470768	0.345081411	564.8652401	35.15101961

1.131873684	0.104495598	0.409746435	0.658133378	0.010463819	3.130594869	0.030139416
0.068248803	25.03655374	2.29750437	0.005371323	40.85597868	0.223448506	0.834984272
1.233624785	0.002468732	1.324569921	19.35224231	0.223754321	5.783017396	27.19953855
0.10440408	0.106161149	0.220974484	13.00925642	4.61085615	994.735656	7.927859603
0.045881857	0.060830394	1.121507667	37.59574032	0.842340423	3.130594869	0.030139416
0.054042941	0.067966332	0.323370206	0.005361322	3.543836129	46.20629421	1878431.817
1.834601339	23.40729065	0.01153332	0.001577605	82.17921804	46.20629421	1878431.817
1.294216626	1.472267813	0.017485495	0.233785035	0.133971022	46.20629421	1878431.817
0.189064125	31.25389488	0.017825268	0.218741389	0.064588327	3.130594869	0.030139416
0.58449341	3.212285593	1.219583574	0.000101639	0.501844355	994.735656	7.927859603
0.054042941	0.067966332	0.323370206	0.005361322	3.543836129	3.130594869	0.030139416
0.434237766	0.083081926	0.339648941	23.65470768	0.345081411	3.130594869	0.030139416
0.434237766	0.083081926	0.339648941	23.65470768	0.345081411	994.735656	7.927859603
1.233624785	0.002468732	1.324569921	19.35224231	0.223754321	0.855106219	3030.198032
1.834601339	23.40729065	0.01153332	0.001577605	82.17921804	0.024990864	512.289609
0.616126345	7.810474176	4.322315691	0.000859768	29.67646416	46.20629421	1878431.817
0.054042941	0.067966332	0.323370206	0.005361322	3.543836129	3.130594869	0.030139416
0.189064125	31.25389488	0.017825268	0.218741389	0.064588327	994.735656	7.927859603
1.294216626	1.472267813	0.017485495	0.233785035	0.133971022	994.735656	7.927859603
0.068248803	25.03655374	2.29750437	0.005371323	40.85597868	1382.594644	0.305071512
0.189064125	31.25389488	0.017825268	0.218741389	0.064588327	994.735656	7.927859603
0.054042941	0.067966332	0.323370206	0.005361322	3.543836129	89.45877868	159.4564655
1.435246096	0.019050387	1.926961808	67.24171692	0.166229381	1813.425348	119142.7764
1.435246096	0.019050387	1.926961808	67.24171692	0.166229381	3.130594869	0.030139416
7.979498795	0.010492223	6.538933844	233.7877338	0.053036836	5.561172778	0.000704147
0.189064125	31.25389488	0.017825268	0.218741389	0.064588327	3.130594869	0.030139416
1.834601339	23.40729065	0.01153332	0.001577605	82.17921804	3.130594869	0.030139416
6.976323472	0.07789373	6.48504354	0.00650483	0.041251428	204.3719434	2827259.242
2.996728801	0.005077949	6.29290126	506.7283609	0.121232565	564.8652401	35.15101961
0.189064125	31.25389488	0.017825268	0.218741389	0.064588327	0.235283773	761.746319
0.189064125	31.25389488	0.017825268	0.218741389	0.064588327	994.735656	7.927859603
1.834601339	23.40729065	0.01153332	0.001577605	82.17921804	1.401537911	0.002591964
7.979498795	0.010492223	6.538933844	233.7877338	0.053036836	7.972298343	92.02553404
1.834601339	23.40729065	0.01153332	0.001577605	82.17921804	5.256930015	0.006286635
0.054042941	0.067966332	0.323370206	0.005361322	3.543836129	46.20629421	1878431.817
0.616126345	7.810474176	4.322315691	0.000859768	29.67646416	994.735656	7.927859603
6.976323472	0.07789373	6.48504354	0.00650483	0.041251428	46.20629421	1878431.817
2.996728801	0.005077949	6.29290126	506.7283609	0.121232565	0.223448506	0.834984272
1.606268382	1.31810948	2.161378439	5.246904741	1.315227066	89.45877868	159.4564655
0.58449341	3.212285593	1.219583574	0.000101639	0.501844355	3.130594869	0.030139416
3.021486852	53.40437571	2.793263543	85.11011316	12.7755358	1382.594644	0.305071512

2.996728801	0.005077949	6.29290126	506.7283609	0.121232565	7.972298343	92.02553404
0.58449341	3.212285593	1.219583574	0.000101639	0.501844355	3.130594869	0.030139416
2.996728801	0.005077949	6.29290126	506.7283609	0.121232565	46.20629421	1878431.817
0.045881857	0.060830394	1.121507667	37.59574032	0.842340423	5.461427683	1077080.988
0.045881857	0.060830394	1.121507667	37.59574032	0.842340423	994.735656	7.927859603
1.606268382	1.31810948	2.161378439	5.246904741	1.315227066	46.20629421	1878431.817
7.979498795	0.010492223	6.538933844	233.7877338	0.053036836	46.20629421	1878431.817
1.294216626	1.472267813	0.017485495	0.233785035	0.133971022	98.37328866	205.7148389
3.021486852	53.40437571	2.793263543	85.11011316	12.7755358	1382.594644	0.305071512
7.979498795	0.010492223	6.538933844	233.7877338	0.053036836	0.235283773	761.746319
0.189064125	31.25389488	0.017825268	0.218741389	0.064588327	994.735656	7.927859603
1.233624785	0.002468732	1.324569921	19.35224231	0.223754321	3.130594869	0.030139416
0.616126345	7.810474176	4.322315691	0.000859768	29.67646416	994.735656	7.927859603
0.616126345	7.810474176	4.322315691	0.000859768	29.67646416	1813.425348	119142.7764
1.834601339	23.40729065	0.01153332	0.001577605	82.17921804	5.256930015	0.006286635
1.435246096	0.019050387	1.926961808	67.24171692	0.166229381	46.20629421	1878431.817
1.834601339	23.40729065	0.01153332	0.001577605	82.17921804	5.783017396	27.19953855
7.979498795	0.010492223	6.538933844	233.7877338	0.053036836	564.8652401	35.15101961
0.068248803	25.03655374	2.29750437	0.005371323	40.85597868	0.084945152	0.666990842
6.976323472	0.07789373	6.48504354	0.00650483	0.041251428	0.350754565	0.29243781
146.2283384	108.9343921	159.5737034	14487.86218	138.0280438	0.148368246	0.032231981
0.054042941	0.067966332	0.323370206	0.005361322	3.543836129	537.637133	0.502829598
7.979498795	0.010492223	6.538933844	233.7877338	0.053036836	5.460699865	0.288803204
0.068248803	25.03655374	2.29750437	0.005371323	40.85597868	0.038147525	12.2829238
146.2283384	108.9343921	159.5737034	14487.86218	138.0280438	3.130594869	0.030139416
2.991096614	2.049696262	1.065523117	0.179610991	14.80615949	994.735656	7.927859603
0.616126345	7.810474176	4.322315691	0.000859768	29.67646416	5.256930015	0.006286635
1.606268382	1.31810948	2.161378439	5.246904741	1.315227066	5.561172778	0.000704147
1.435246096	0.019050387	1.926961808	67.24171692	0.166229381	5.783017396	27.19953855
0.616126345	7.810474176	4.322315691	0.000859768	29.67646416	46.20629421	1878431.817
0.434237766	0.083081926	0.339648941	23.65470768	0.345081411	46.20629421	1878431.817
2.996728801	0.005077949	6.29290126	506.7283609	0.121232565	3.130594869	0.030139416
0.58449341	3.212285593	1.219583574	0.000101639	0.501844355	994.735656	7.927859603
0.10440408	0.106161149	0.220974484	13.00925642	4.61085615	564.8652401	35.15101961
6.976323472	0.07789373	6.48504354	0.00650483	0.041251428	5.461427683	1077080.988
1.294216626	1.472267813	0.017485495	0.233785035	0.133971022	1.749696823	0.000359589
2.996728801	0.005077949	6.29290126	506.7283609	0.121232565	3.130594869	0.030139416
0.58449341	3.212285593	1.219583574	0.000101639	0.501844355	994.735656	7.927859603
6.976323472	0.07789373	6.48504354	0.00650483	0.041251428	109.85648	0.961963177
0.616126345	7.810474176	4.322315691	0.000859768	29.67646416	994.735656	7.927859603
1.233624785	0.002468732	1.324569921	19.35224231	0.223754321	3.709490109	4.052575238

0.045881857	0.060830394	1.121507667	37.59574032	0.842340423	5.460699865	0.288803204
0.045881857	0.060830394	1.121507667	37.59574032	0.842340423	46.20629421	1878431.817
6.976323472	0.07789373	6.48504354	0.00650483	0.041251428	564.8652401	35.15101961
1.233624785	0.002468732	1.324569921	19.35224231	0.223754321	5.783017396	27.19953855
1.233624785	0.002468732	1.324569921	19.35224231	0.223754321	1813.425348	119142.7764
1.131873684	0.104495598	0.409746435	0.658133378	0.010463819	3.130594869	0.030139416
0.58449341	3.212285593	1.219583574	0.000101639	0.501844355	0.235283773	761.746319
146.2283384	108.9343921	159.5737034	14487.86218	138.0280438	0.148368246	0.032231981
2.991096614	2.049696262	1.065523117	0.179610991	14.80615949	5.256930015	0.006286635
1.834601339	23.40729065	0.01153332	0.001577605	82.17921804	3.709490109	4.052575238
1.435246096	0.019050387	1.926961808	67.24171692	0.166229381	5.783017396	27.19953855
3.021486852	53.40437571	2.793263543	85.11011316	12.7755358	204.3719434	2827259.242
1.606268382	1.31810948	2.161378439	5.246904741	1.315227066	0.235283773	761.746319
1.294216626	1.472267813	0.017485495	0.233785035	0.133971022	0.024990864	512.289609
0.189064125	31.25389488	0.017825268	0.218741389	0.064588327	994.735656	7.927859603
0.045881857	0.060830394	1.121507667	37.59574032	0.842340423	0.235283773	761.746319
0.045881857	0.060830394	1.121507667	37.59574032	0.842340423	994.735656	7.927859603
1.834601339	23.40729065	0.01153332	0.001577605	82.17921804	5.783017396	27.19953855
2.996728801	0.005077949	6.29290126	506.7283609	0.121232565	46.20629421	1878431.817
146.2283384	108.9343921	159.5737034	14487.86218	138.0280438	5.783017396	27.19953855
2.996728801	0.005077949	6.29290126	506.7283609	0.121232565	3.130594869	0.030139416
0.045881857	0.060830394	1.121507667	37.59574032	0.842340423	46.20629421	1878431.817
0.054042941	0.067966332	0.323370206	0.005361322	3.543836129	3.130594869	0.030139416
1.606268382	1.31810948	2.161378439	5.246904741	1.315227066	1.401537911	0.002591964
1.233624785	0.002468732	1.324569921	19.35224231	0.223754321	5.783017396	27.19953855
0.068248803	25.03655374	2.29750437	0.005371323	40.85597868	7.972298343	92.02553404
1.233624785	0.002468732	1.324569921	19.35224231	0.223754321	537.637133	0.502829598
0.189064125	31.25389488	0.017825268	0.218741389	0.064588327	46.20629421	1878431.817
2.996728801	0.005077949	6.29290126	506.7283609	0.121232565	5.460699865	0.288803204
2.996728801	0.005077949	6.29290126	506.7283609	0.121232565	46.20629421	1878431.817
0.58449341	3.212285593	1.219583574	0.000101639	0.501844355	1.401537911	0.002591964
1.435246096	0.019050387	1.926961808	67.24171692	0.166229381	5.783017396	27.19953855
1.233624785	0.002468732	1.324569921	19.35224231	0.223754321	0.435715791	121.8315447
146.2283384	108.9343921	159.5737034	14487.86218	138.0280438	5.256930015	0.006286635
7.979498795	0.010492223	6.538933844	233.7877338	0.053036836	204.3719434	2827259.242
2.996728801	0.005077949	6.29290126	506.7283609	0.121232565	5.256930015	0.006286635
0.045881857	0.060830394	1.121507667	37.59574032	0.842340423	3.130594869	0.030139416
1.131873684	0.104495598	0.409746435	0.658133378	0.010463819	1.749696823	0.000359589
1.834601339	23.40729065	0.01153332	0.001577605	82.17921804	5.783017396	27.19953855
0.10440408	0.106161149	0.220974484	13.00925642	4.61085615	5.561172778	0.000704147
146.2283384	108.9343921	159.5737034	14487.86218	138.0280438	89.45877868	159.4564655

6.976323472	0.07789373	6.48504354	0.00650483	0.041251428	1.749696823	0.000359589
1.435246096	0.019050387	1.926961808	67.24171692	0.166229381	0.038147525	12.2829238
1.435246096	0.019050387	1.926961808	67.24171692	0.166229381	994.735656	7.927859603
0.434237766	0.083081926	0.339648941	23.65470768	0.345081411	994.735656	7.927859603
6.976323472	0.07789373	6.48504354	0.00650483	0.041251428	3.130594869	0.030139416
0.434237766	0.083081926	0.339648941	23.65470768	0.345081411	0.435715791	121.8315447
0.054042941	0.067966332	0.323370206	0.005361322	3.543836129	109.85648	0.961963177
0.068248803	25.03655374	2.29750437	0.005371323	40.85597868	0.474239135	71.62581966
0.189064125	31.25389488	0.017825268	0.218741389	0.064588327	5.460699865	0.288803204
0.054042941	0.067966332	0.323370206	0.005361322	3.543836129	0.435715791	121.8315447
0.10440408	0.106161149	0.220974484	13.00925642	4.61085615	0.046588643	1.783326953
7.979498795	0.010492223	6.538933844	233.7877338	0.053036836	994.735656	7.927859603
0.10440408	0.106161149	0.220974484	13.00925642	4.61085615	564.8652401	35.15101961
3.021486852	53.40437571	2.793263543	85.11011316	12.7755358	0.001671576	0.001066916
0.045881857	0.060830394	1.121507667	37.59574032	0.842340423	1.064090239	0.001329755
6.976323472	0.07789373	6.48504354	0.00650483	0.041251428	0.435715791	121.8315447
0.10440408	0.106161149	0.220974484	13.00925642	4.61085615	89.45877868	159.4564655
2.991096614	2.049696262	1.065523117	0.179610991	14.80615949	5.783017396	27.19953855
0.054042941	0.067966332	0.323370206	0.005361322	3.543836129	994.735656	7.927859603
0.054042941	0.067966332	0.323370206	0.005361322	3.543836129	10.02766643	2.691111002
1.606268382	1.31810948	2.161378439	5.246904741	1.315227066	46.20629421	1878431.817
1.294216626	1.472267813	0.017485495	0.233785035	0.133971022	5.561172778	0.000704147
0.189064125	31.25389488	0.017825268	0.218741389	0.064588327	3.130594869	0.030139416
0.189064125	31.25389488	0.017825268	0.218741389	0.064588327	10.66908927	0.028558397
1.834601339	23.40729065	0.01153332	0.001577605	82.17921804	0.235283773	761.746319
6.976323472	0.07789373	6.48504354	0.00650483	0.041251428	3.709490109	4.052575238
0.068248803	25.03655374	2.29750437	0.005371323	40.85597868	1203.152028	8852.522525
2.996728801	0.005077949	6.29290126	506.7283609	0.121232565	46.20629421	1878431.817
0.616126345	7.810474176	4.322315691	0.000859768	29.67646416	5.561172778	0.000704147
1.467858565	26.83914793	4.171133489	16.73814337	0.033727705	3.709490109	4.052575238
0.045881857	0.060830394	1.121507667	37.59574032	0.842340423	5.783017396	27.19953855
0.054042941	0.067966332	0.323370206	0.005361322	3.543836129	0.435715791	121.8315447
146.2283384	108.9343921	159.5737034	14487.86218	138.0280438	5.561172778	0.000704147
0.434237766	0.083081926	0.339648941	23.65470768	0.345081411	1.749696823	0.000359589
146.2283384	108.9343921	159.5737034	14487.86218	138.0280438	0.474239135	71.62581966
0.10440408	0.106161149	0.220974484	13.00925642	4.61085615	0.435715791	121.8315447
0.045881857	0.060830394	1.121507667	37.59574032	0.842340423	994.735656	7.927859603
0.068248803	25.03655374	2.29750437	0.005371323	40.85597868	7.972298343	92.02553404
0.10440408	0.106161149	0.220974484	13.00925642	4.61085615	0.435715791	121.8315447
1.606268382	1.31810948	2.161378439	5.246904741	1.315227066	0.235283773	761.746319
1.131873684	0.104495598	0.409746435	0.658133378	0.010463819	994.735656	7.927859603

1.606268382	1.31810948	2.161378439	5.246904741	1.315227066	46.20629421	1878431.817
1.131873684	0.104495598	0.409746435	0.658133378	0.010463819	10.02766643	2.691111002
0.58449341	3.212285593	1.219583574	0.000101639	0.501844355	3.709490109	4.052575238
0.10440408	0.106161149	0.220974484	13.00925642	4.61085615	0.024990864	512.289609
146.2283384	108.9343921	159.5737034	14487.86218	138.0280438	1813.425348	119142.7764
146.2283384	108.9343921	159.5737034	14487.86218	138.0280438	10.66908927	0.028558397
1.834601339	23.40729065	0.01153332	0.001577605	82.17921804	1382.594644	0.305071512
0.189064125	31.25389488	0.017825268	0.218741389	0.064588327	0.235283773	761.746319
0.58449341	3.212285593	1.219583574	0.000101639	0.501844355	0.235283773	761.746319
1.131873684	0.104495598	0.409746435	0.658133378	0.010463819	3.130594869	0.030139416
2.996728801	0.005077949	6.29290126	506.7283609	0.121232565	5.561172778	0.000704147
6.976323472	0.07789373	6.48504354	0.00650483	0.041251428	3.709490109	4.052575238
1.435246096	0.019050387	1.926961808	67.24171692	0.166229381	89.45877868	159.4564655
0.434237766	0.083081926	0.339648941	23.65470768	0.345081411	46.20629421	1878431.817
0.58449341	3.212285593	1.219583574	0.000101639	0.501844355	1813.425348	119142.7764
2.991096614	2.049696262	1.065523117	0.179610991	14.80615949	3.130594869	0.030139416
2.991096614	2.049696262	1.065523117	0.179610991	14.80615949	46.20629421	1878431.817
0.054042941	0.067966332	0.323370206	0.005361322	3.543836129	0.417959285	7322.272056
0.054042941	0.067966332	0.323370206	0.005361322	3.543836129	98.37328866	205.7148389
6.976323472	0.07789373	6.48504354	0.00650483	0.041251428	1813.425348	119142.7764
0.045881857	0.060830394	1.121507667	37.59574032	0.842340423	0.350754565	0.29243781
0.045881857	0.060830394	1.121507667	37.59574032	0.842340423	89.45877868	159.4564655
1.834601339	23.40729065	0.01153332	0.001577605	82.17921804	1382.594644	0.305071512
6.976323472	0.07789373	6.48504354	0.00650483	0.041251428	5.460699865	0.288803204
0.616126345	7.810474176	4.322315691	0.000859768	29.67646416	1.064090239	0.001329755
0.58449341	3.212285593	1.219583574	0.000101639	0.501844355	5.256930015	0.006286635
0.616126345	7.810474176	4.322315691	0.000859768	29.67646416	3.709490109	4.052575238
0.434237766	0.083081926	0.339648941	23.65470768	0.345081411	564.8652401	35.15101961
0.054042941	0.067966332	0.323370206	0.005361322	3.543836129	994.735656	7.927859603
6.976323472	0.07789373	6.48504354	0.00650483	0.041251428	5.461427683	1077080.988
1.467858565	26.83914793	4.171133489	16.73814337	0.033727705	0.855106219	3030.198032
0.434237766	0.083081926	0.339648941	23.65470768	0.345081411	46.20629421	1878431.817
0.189064125	31.25389488	0.017825268	0.218741389	0.064588327	89.45877868	159.4564655
0.189064125	31.25389488	0.017825268	0.218741389	0.064588327	3.709490109	4.052575238
1.294216626	1.472267813	0.017485495	0.233785035	0.133971022	3.709490109	4.052575238
0.189064125	31.25389488	0.017825268	0.218741389	0.064588327	0.350754565	0.29243781
0.434237766	0.083081926	0.339648941	23.65470768	0.345081411	5.561172778	0.000704147
1.233624785	0.002468732	1.324569921	19.35224231	0.223754321	1.749696823	0.000359589
0.434237766	0.083081926	0.339648941	23.65470768	0.345081411	0.024990864	512.289609
1.233624785	0.002468732	1.324569921	19.35224231	0.223754321	0.235283773	761.746319
6.976323472	0.07789373	6.48504354	0.00650483	0.041251428	0.001671576	0.001066916

2.996728801	0.005077949	6.29290126	506.7283609	0.121232565	0.046588643	1.783326953
7.979498795	0.010492223	6.538933844	233.7877338	0.053036836	46.20629421	1878431.817
3.021486852	53.40437571	2.793263543	85.11011316	12.7755358	564.8652401	35.15101961
2.991096614	2.049696262	1.065523117	0.179610991	14.80615949	0.235283773	761.746319
0.054042941	0.067966332	0.323370206	0.005361322	3.543836129	46.20629421	1878431.817
1.294216626	1.472267813	0.017485495	0.233785035	0.133971022	5.460699865	0.288803204
1.435246096	0.019050387	1.926961808	67.24171692	0.166229381	0.435715791	121.8315447
0.58449341	3.212285593	1.219583574	0.000101639	0.501844355	5.561172778	0.000704147
0.068248803	25.03655374	2.29750437	0.005371323	40.85597868	89.45877868	159.4564655
1.233624785	0.002468732	1.324569921	19.35224231	0.223754321	3.130594869	0.030139416
0.616126345	7.810474176	4.322315691	0.000859768	29.67646416	5.561172778	0.000704147
7.979498795	0.010492223	6.538933844	233.7877338	0.053036836	994.735656	7.927859603
0.10440408	0.106161149	0.220974484	13.00925642	4.61085615	0.001671576	0.001066916
3.021486852	53.40437571	2.793263543	85.11011316	12.7755358	0.235283773	761.746319
0.10440408	0.106161149	0.220974484	13.00925642	4.61085615	10.66908927	0.028558397
0.054042941	0.067966332	0.323370206	0.005361322	3.543836129	3.130594869	0.030139416
0.045881857	0.060830394	1.121507667	37.59574032	0.842340423	10.66908927	0.028558397
0.10440408	0.106161149	0.220974484	13.00925642	4.61085615	3.130594869	0.030139416
14	15	16	17	18	19	20
ENSG00000199282	hsa-mir-523	hsa-mir-376a-2/1 F2	ENSG00000199856 F1	HBII-276 F2	RASGRP4-Hs01073179_m1	FAM20A-Hs01034071_m1

21	22	23	24	25	26
FAM20A-Hs01034070_m1	NEK9-Hs00929602_m1	NEK9-Hs00929594_m1	ABCC1-Hs01561504_m1	SORBS2-Hs01125202_m1	SORBS2-Hs00243432_m1
4.201129015	13.31083275	120.8426322	8.693260019	3.261151618	127.0351491
2.29E-05	77.30813646	0.008522228	26.2535707	6.60E-05	29.24473361
3622526.011	0.008667257	1567.530984	0.040325128	974004.4066	0.174126792
82.09520209	0.003124926	12210.5566	505.9823121	8738.83947	0.130670943
50.8682042	66.98785879	50.36100203	1113.979827	55964.3554	608.5811021
121.9456688	0.003024855	0.012464776	1358.009196	0.002572878	500.0785355
0.000763898	1048.212877	150.2773483	0.310058345	99.28606947	36564.5614
3.087238517	3.661576444	0.051274012	0.000288236	1.964298564	0.001572733
1.541377957	0.050721911	1.438812932	2.799676923	31233.84496	23686552.89
0.000429801	572453.1253	647580.3116	0.003401511	0.001241459	50.83032856
3.346371755	57.65125892	2.386195416	0.003475588	0.001268495	0.018964239
82.09520209	0.003124926	12210.5566	505.9823121	8738.83947	0.130670943
196231178.5	65.16541478	46728075.07	8.99580733	24.63396858	1004.490647
1.541377957	0.050721911	1.438812932	2.799676923	31233.84496	23686552.89
19.76270796	18239.10908	64.7223915	1197.292549	11224672.12	77.92872483
0.016256901	0.000636636	2.955101313	0.001220587	1003.51346	0.006660026
13.42683781	3609.173242	2.982823888	22.77493565	1033180.709	118.7520414
6.363584693	16.00281719	0.000114459	20.33236256	0.000369945	63.35755265
3622526.011	0.008667257	1567.530984	0.040325128	974004.4066	0.174126792
0.483859469	3.417629738	0.179642558	1904.263075	508083.8373	0.084835548
0.016256901	0.000636636	2.955101313	0.001220587	1003.51346	0.006660026
1.683872036	302.35441	0.000640012	210.367052	198.471201	357.3258222
255.3617576	969.1698409	1263.390847	982.817445	477.8042377	2064.469134
3.346371755	57.65125892	2.386195416	0.003475588	0.001268495	0.018964239
50.8682042	66.98785879	50.36100203	1113.979827	55964.3554	608.5811021
255.3617576	969.1698409	1263.390847	982.817445	477.8042377	2064.469134
0.000429801	572453.1253	647580.3116	0.003401511	0.001241459	50.83032856
3.346371755	57.65125892	2.386195416	0.003475588	0.001268495	0.018964239
19.76270796	18239.10908	64.7223915	1197.292549	11224672.12	77.92872483
1.541377957	0.050721911	1.438812932	2.799676923	31233.84496	23686552.89
4.201129015	13.31083275	120.8426322	8.693260019	3.261151618	127.0351491
1.183470452	94.91985695	201.7096781	1584.237066	19976.65713	0.042484392
0.016256901	0.000636636	2.955101313	0.001220587	1003.51346	0.006660026
121.9456688	0.003024855	0.012464776	1358.009196	0.002572878	500.0785355
2.463601515	0.848361737	0.700903747	31.26765214	3.396078394	45.14766929
0.000429801	572453.1253	647580.3116	0.003401511	0.001241459	50.83032856
1.683872036	302.35441	0.000640012	210.367052	198.471201	357.3258222

255.3617576	969.1698409	1263.390847	982.817445	477.8042377	2064.469134
121.9456688	0.003024855	0.012464776	1358.009196	0.002572878	500.0785355
102.9794617	16.46687386	108.644713	86.16468494	4162549.24	57.93633538
0.016256901	0.000636636	2.955101313	0.001220587	1003.51346	0.006660026
0.000429801	572453.1253	647580.3116	0.003401511	0.001241459	50.83032856
121.9456688	0.003024855	0.012464776	1358.009196	0.002572878	500.0785355
1.183470452	94.91985695	201.7096781	1584.237066	19976.65713	0.042484392
0.483859469	3.417629738	0.179642558	1904.263075	508083.8373	0.084835548
50.8682042	66.98785879	50.36100203	1113.979827	55964.3554	608.5811021
102.9794617	16.46687386	108.644713	86.16468494	4162549.24	57.93633538
50.8682042	66.98785879	50.36100203	1113.979827	55964.3554	608.5811021
3622526.011	0.008667257	1567.530984	0.040325128	974004.4066	0.174126792
1.683872036	302.35441	0.000640012	210.367052	198.471201	357.3258222
4487.45971	6.258662585	0.004241355	25.95868285	0.012502668	28.44240761
632.5494359	810.1076369	13220.25995	6589.651217	719.7321552	0.171411922
486.4054891	550.1680199	52.72873241	2140.935077	3244.630204	0.033449865
108.4214341	200.637552	11.0883451	164538.7808	2019.579319	541641.1111
10.30718391	46.38520534	1269169.494	130.1502729	0.001334923	452.1043041
0.00200755	2555.109525	0.044279049	0.015888065	792840.1904	0.086691829
0.000979891	9952.926727	31.44350634	0.007755013	1012.918964	52.36532071
108.4214341	200.637552	11.0883451	164538.7808	2019.579319	541641.1111
6.644190091	0.000207344	1.429212492	0.000386378	0.000141018	67.98780398
6455.737782	32.00136959	98.01578473	78.38941417	47.3651068	1111.206106
486.4054891	550.1680199	52.72873241	2140.935077	3244.630204	0.033449865
59211.87468	10052.71621	1850.202975	0.004365964	0.001593459	176.1041742
0.00200755	2555.109525	0.044279049	0.015888065	792840.1904	0.086691829
173.8163838	2312.472214	442.8152069	876.1962988	9.995255165	279547.1921
59211.87468	10052.71621	1850.202975	0.004365964	0.001593459	176.1041742
108.4214341	200.637552	11.0883451	164538.7808	2019.579319	541641.1111
0.00200755	2555.109525	0.044279049	0.015888065	792840.1904	0.086691829
5.003893849	2.341300122	14.36543789	0.001867833	938.2246245	299.9465697
1147.454244	3174.217162	16.9213751	1098.70442	2526.74145	2599.907783
6.644190091	0.000207344	1.429212492	0.000386378	0.000141018	67.98780398
59211.87468	10052.71621	1850.202975	0.004365964	0.001593459	176.1041742
0.214874278	12.5041051	0.022006554	26.02689118	26.22470417	0.001728281
108.4214341	200.637552	11.0883451	164538.7808	2019.579319	541641.1111
0.000979891	9952.926727	31.44350634	0.007755013	1012.918964	52.36532071
59211.87468	10052.71621	1850.202975	0.004365964	0.001593459	176.1041742
108.4214341	200.637552	11.0883451	164538.7808	2019.579319	541641.1111
59211.87468	10052.71621	1850.202975	0.004365964	0.001593459	176.1041742
7097.862071	21.65632535	0.001125336	8503177.364	23.41964411	197.3457173

161500.6741	33.9454569	22.14867525	112.5713209	46.91761774	0.017789605
632.5494359	810.1076369	13220.25995	6589.651217	719.7321552	0.171411922
486.4054891	550.1680199	52.72873241	2140.935077	3244.630204	0.033449865
10.30718391	46.38520534	1269169.494	130.1502729	0.001334923	452.1043041
265807.5481	532053037	75.47818323	12.09862041	805.9681736	0.469246636
6.644190091	0.000207344	1.429212492	0.000386378	0.000141018	67.98780398
7097.862071	21.65632535	0.001125336	8503177.364	23.41964411	197.3457173
55.77654081	51.25832249	41967.41926	2355584.738	469.0155301	850.067247
6.644190091	0.000207344	1.429212492	0.000386378	0.000141018	67.98780398
8877.672958	0.001017116	0.004604691	2.15924121	207.7786831	1924.755597
0.214874278	12.5041051	0.022006554	26.02689118	26.22470417	0.001728281
4116.526367	26379051.1	11.66451751	65376.39617	44939.91948	0.328534252
0.00200755	2555.109525	0.044279049	0.015888065	792840.1904	0.086691829
108.4214341	200.637552	11.0883451	164538.7808	2019.579319	541641.1111
0.00200755	2555.109525	0.044279049	0.015888065	792840.1904	0.086691829
59211.87468	10052.71621	1850.202975	0.004365964	0.001593459	176.1041742
59211.87468	10052.71621	1850.202975	0.004365964	0.001593459	176.1041742
59211.87468	10052.71621	1850.202975	0.004365964	0.001593459	176.1041742
59211.87468	10052.71621	1850.202975	0.004365964	0.001593459	176.1041742
59211.87468	10052.71621	1850.202975	0.004365964	0.001593459	176.1041742
386922.3396	1166.118064	123.3711813	20.97927111	0.000560707	935.5945599
6.644190091	0.000207344	1.429212492	0.000386378	0.000141018	67.98780398
108.4214341	200.637552	11.0883451	164538.7808	2019.579319	541641.1111
486.4054891	550.1680199	52.72873241	2140.935077	3244.630204	0.033449865
108.4214341	200.637552	11.0883451	164538.7808	2019.579319	541641.1111
0.00200755	2555.109525	0.044279049	0.015888065	792840.1904	0.086691829
6.644190091	0.000207344	1.429212492	0.000386378	0.000141018	67.98780398
0.000965308	2.558042527	8.081052703	7033.539162	54422.90696	417.0029921
59211.87468	10052.71621	1850.202975	0.004365964	0.001593459	176.1041742
59211.87468	10052.71621	1850.202975	0.004365964	0.001593459	176.1041742
0.002392787	0.002471021	0.002138362	86.80309912	0.006911452	1318.899463
0.000704862	1.20139047	410.0019847	219.6530538	6.447011111	743.0723488
0.000965308	2.558042527	8.081052703	7033.539162	54422.90696	417.0029921
0.00200755	2555.109525	0.044279049	0.015888065	792840.1904	0.086691829
71724.35614	49.03588381	3.647385731	0.008117099	0.002962521	354.9515936
6455.737782	32.00136959	98.01578473	78.38941417	47.3651068	1111.206106
4487.45971	6.258662585	0.004241355	25.95868285	0.012502668	28.44240761
55.77654081	51.25832249	41967.41926	2355584.738	469.0155301	850.067247
10.30718391	46.38520534	1269169.494	130.1502729	0.001334923	452.1043041
0.00200755	2555.109525	0.044279049	0.015888065	792840.1904	0.086691829
55.77654081	51.25832249	41967.41926	2355584.738	469.0155301	850.067247

59211.87468	10052.71621	1850.202975	0.004365964	0.001593459	176.1041742
4429.448416	1390.450894	2.418900266	31.11679763	137.5906427	1559.604734
0.00200755	2555.109525	0.044279049	0.015888065	792840.1904	0.086691829
6455.737782	32.00136959	98.01578473	78.38941417	47.3651068	1111.206106
108.4214341	200.637552	11.0883451	164538.7808	2019.579319	541641.1111
0.00200755	2555.109525	0.044279049	0.015888065	792840.1904	0.086691829
486.4054891	550.1680199	52.72873241	2140.935077	3244.630204	0.033449865
4429.448416	1390.450894	2.418900266	31.11679763	137.5906427	1559.604734
0.00200755	2555.109525	0.044279049	0.015888065	792840.1904	0.086691829
108.4214341	200.637552	11.0883451	164538.7808	2019.579319	541641.1111
59211.87468	10052.71621	1850.202975	0.004365964	0.001593459	176.1041742
0.000979891	9952.926727	31.44350634	0.007755013	1012.918964	52.36532071
0.00200755	2555.109525	0.044279049	0.015888065	792840.1904	0.086691829
108.4214341	200.637552	11.0883451	164538.7808	2019.579319	541641.1111
0.00200755	2555.109525	0.044279049	0.015888065	792840.1904	0.086691829
0.000979891	9952.926727	31.44350634	0.007755013	1012.918964	52.36532071
0.00200755	2555.109525	0.044279049	0.015888065	792840.1904	0.086691829
0.572194492	128.43259	39.85754938	904776.6251	59.50828257	0.01595297
0.000965308	2.558042527	8.081052703	7033.539162	54422.90696	417.0029921
0.214874278	12.5041051	0.022006554	26.02689118	26.22470417	0.001728281
0.15580288	413430.0189	68.20551738	0.000347322	1807781.746	2847349.069
386922.3396	1166.118064	123.3711813	20.97927111	0.000560707	935.5945599
108.4214341	200.637552	11.0883451	164538.7808	2019.579319	541641.1111
1147.454244	3174.217162	16.9213751	1098.70442	2526.74145	2599.907783
10.30718391	46.38520534	1269169.494	130.1502729	0.001334923	452.1043041
0.000704862	1.20139047	410.0019847	219.6530538	6.447011111	743.0723488
4429.448416	1390.450894	2.418900266	31.11679763	137.5906427	1559.604734
0.000979891	9952.926727	31.44350634	0.007755013	1012.918964	52.36532071
0.130456738	22.40148469	0.629752132	5.150075028	0.004913846	679.5452071
0.15580288	413430.0189	68.20551738	0.000347322	1807781.746	2847349.069
108.4214341	200.637552	11.0883451	164538.7808	2019.579319	541641.1111
7097.862071	21.65632535	0.001125336	8503177.364	23.41964411	197.3457173
4429.448416	1390.450894	2.418900266	31.11679763	137.5906427	1559.604734
108.4214341	200.637552	11.0883451	164538.7808	2019.579319	541641.1111
10.30718391	46.38520534	1269169.494	130.1502729	0.001334923	452.1043041
59211.87468	10052.71621	1850.202975	0.004365964	0.001593459	176.1041742
0.00200755	2555.109525	0.044279049	0.015888065	792840.1904	0.086691829
632.5494359	810.1076369	13220.25995	6589.651217	719.7321552	0.171411922
108.4214341	200.637552	11.0883451	164538.7808	2019.579319	541641.1111
108.4214341	200.637552	11.0883451	164538.7808	2019.579319	541641.1111
0.000979891	9952.926727	31.44350634	0.007755013	1012.918964	52.36532071

55.77654081	51.25832249	41967.41926	2355584.738	469.0155301	850.067247
386922.3396	1166.118064	123.3711813	20.97927111	0.000560707	935.5945599
28.15890429	17.26754598	9.142346424	0.012909416	3040.886627	0.04266276
0.852943194	0.001249056	125.3972904	0.009572251	18.62968355	113423.7866
12.81732419	0.000824651	0.142698834	135.390057	0.492581713	65.4100407
0.214874278	12.5041051	0.022006554	26.02689118	26.22470417	0.001728281
4429.448416	1390.450894	2.418900266	31.11679763	137.5906427	1559.604734
6.644190091	0.000207344	1.429212492	0.000386378	0.000141018	67.98780398
6455.737782	32.00136959	98.01578473	78.38941417	47.3651068	1111.206106
0.000965308	2.558042527	8.081052703	7033.539162	54422.90696	417.0029921
0.15580288	413430.0189	68.20551738	0.000347322	1807781.746	2847349.069
7097.862071	21.65632535	0.001125336	8503177.364	23.41964411	197.3457173
59211.87468	10052.71621	1850.202975	0.004365964	0.001593459	176.1041742
59211.87468	10052.71621	1850.202975	0.004365964	0.001593459	176.1041742
6.644190091	0.000207344	1.429212492	0.000386378	0.000141018	67.98780398
59211.87468	10052.71621	1850.202975	0.004365964	0.001593459	176.1041742
59211.87468	10052.71621	1850.202975	0.004365964	0.001593459	176.1041742
59211.87468	10052.71621	1850.202975	0.004365964	0.001593459	176.1041742
59211.87468	10052.71621	1850.202975	0.004365964	0.001593459	176.1041742
6.644190091	0.000207344	1.429212492	0.000386378	0.000141018	67.98780398
0.00200755	2555.109525	0.044279049	0.015888065	792840.1904	0.086691829
59211.87468	10052.71621	1850.202975	0.004365964	0.001593459	176.1041742
108.4214341	200.637552	11.0883451	164538.7808	2019.579319	541641.1111
59211.87468	10052.71621	1850.202975	0.004365964	0.001593459	176.1041742
5.003893849	2.341300122	14.36543789	0.001867833	938.2246245	299.9465697
0.000704862	1.20139047	410.0019847	219.6530538	6.447011111	743.0723488
7097.862071	21.65632535	0.001125336	8503177.364	23.41964411	197.3457173
7097.862071	21.65632535	0.001125336	8503177.364	23.41964411	197.3457173
632.5494359	810.1076369	13220.25995	6589.651217	719.7321552	0.171411922
0.161350568	53.25262734	4.46E-05	1.110009499	0.003486747	0.002153378
59211.87468	10052.71621	1850.202975	0.004365964	0.001593459	176.1041742
108.4214341	200.637552	11.0883451	164538.7808	2019.579319	541641.1111
0.130456738	22.40148469	0.629752132	5.150075028	0.004913846	679.5452071
7.124341844	804.3862342	3430.738433	57.89850054	688.2011452	5.41277124
108.4214341	200.637552	11.0883451	164538.7808	2019.579319	541641.1111
0.130456738	22.40148469	0.629752132	5.150075028	0.004913846	679.5452071
55.77654081	51.25832249	41967.41926	2355584.738	469.0155301	850.067247
486.4054891	550.1680199	52.72873241	2140.935077	3244.630204	0.033449865
108.4214341	200.637552	11.0883451	164538.7808	2019.579319	541641.1111
108.4214341	200.637552	11.0883451	164538.7808	2019.579319	541641.1111
59211.87468	10052.71621	1850.202975	0.004365964	0.001593459	176.1041742

0.130456738	22.40148469	0.629752132	5.150075028	0.004913846	679.5452071
6.644190091	0.000207344	1.429212492	0.000386378	0.000141018	67.98780398
59211.87468	10052.71621	1850.202975	0.004365964	0.001593459	176.1041742
0.000704862	1.20139047	410.0019847	219.6530538	6.447011111	743.0723488
108.4214341	200.637552	11.0883451	164538.7808	2019.579319	541641.1111
161500.6741	33.9454569	22.14867525	112.5713209	46.91761774	0.017789605
10.30718391	46.38520534	1269169.494	130.1502729	0.001334923	452.1043041
161500.6741	33.9454569	22.14867525	112.5713209	46.91761774	0.017789605
6455.737782	32.00136959	98.01578473	78.38941417	47.3651068	1111.206106
0.15580288	413430.0189	68.20551738	0.000347322	1807781.746	2847349.069
0.000979891	9952.926727	31.44350634	0.007755013	1012.918964	52.36532071
0.00200755	2555.109525	0.044279049	0.015888065	792840.1904	0.086691829
108.4214341	200.637552	11.0883451	164538.7808	2019.579319	541641.1111
10.30718391	46.38520534	1269169.494	130.1502729	0.001334923	452.1043041
59211.87468	10052.71621	1850.202975	0.004365964	0.001593459	176.1041742
0.000979891	9952.926727	31.44350634	0.007755013	1012.918964	52.36532071
386922.3396	1166.118064	123.3711813	20.97927111	0.000560707	935.5945599
4116.526367	26379051.1	11.66451751	65376.39617	44939.91948	0.328534252
632.5494359	810.1076369	13220.25995	6589.651217	719.7321552	0.171411922
0.130456738	22.40148469	0.629752132	5.150075028	0.004913846	679.5452071
0.000704862	1.20139047	410.0019847	219.6530538	6.447011111	743.0723488
0.00200755	2555.109525	0.044279049	0.015888065	792840.1904	0.086691829
55.77654081	51.25832249	41967.41926	2355584.738	469.0155301	850.067247
7097.862071	21.65632535	0.001125336	8503177.364	23.41964411	197.3457173
0.214874278	12.5041051	0.022006554	26.02689118	26.22470417	0.001728281
0.00200755	2555.109525	0.044279049	0.015888065	792840.1904	0.086691829
59211.87468	10052.71621	1850.202975	0.004365964	0.001593459	176.1041742
59211.87468	10052.71621	1850.202975	0.004365964	0.001593459	176.1041742
10.30718391	46.38520534	1269169.494	130.1502729	0.001334923	452.1043041
59211.87468	10052.71621	1850.202975	0.004365964	0.001593459	176.1041742
0.000979891	9952.926727	31.44350634	0.007755013	1012.918964	52.36532071
59211.87468	10052.71621	1850.202975	0.004365964	0.001593459	176.1041742
59211.87468	10052.71621	1850.202975	0.004365964	0.001593459	176.1041742
0.00200755	2555.109525	0.044279049	0.015888065	792840.1904	0.086691829
0.00200755	2555.109525	0.044279049	0.015888065	792840.1904	0.086691829
108.4214341	200.637552	11.0883451	164538.7808	2019.579319	541641.1111
6.644190091	0.000207344	1.429212492	0.000386378	0.000141018	67.98780398
0.00200755	2555.109525	0.044279049	0.015888065	792840.1904	0.086691829
0.214874278	12.5041051	0.022006554	26.02689118	26.22470417	0.001728281
59211.87468	10052.71621	1850.202975	0.004365964	0.001593459	176.1041742
108.4214341	200.637552	11.0883451	164538.7808	2019.579319	541641.1111

0.000979891	9952.926727	31.44350634	0.007755013	1012.918964	52.36532071
0.130456738	22.40148469	0.629752132	5.150075028	0.004913846	679.5452071
6.644190091	0.000207344	1.429212492	0.000386378	0.000141018	67.98780398
1147.454244	3174.217162	16.9213751	1098.70442	2526.74145	2599.907783
7097.862071	21.65632535	0.001125336	8503177.364	23.41964411	197.3457173
161500.6741	33.9454569	22.14867525	112.5713209	46.91761774	0.017789605
10.30718391	46.38520534	1269169.494	130.1502729	0.001334923	452.1043041
0.000704862	1.20139047	410.0019847	219.6530538	6.447011111	743.0723488
0.15580288	413430.0189	68.20551738	0.000347322	1807781.746	2847349.069
108.4214341	200.637552	11.0883451	164538.7808	2019.579319	541641.1111
10.30718391	46.38520534	1269169.494	130.1502729	0.001334923	452.1043041
0.15580288	413430.0189	68.20551738	0.000347322	1807781.746	2847349.069
0.000704862	1.20139047	410.0019847	219.6530538	6.447011111	743.0723488
55.77654081	51.25832249	41967.41926	2355584.738	469.0155301	850.067247
4429.448416	1390.450894	2.418900266	31.11679763	137.5906427	1559.604734
108.4214341	200.637552	11.0883451	164538.7808	2019.579319	541641.1111
0.214874278	12.5041051	0.022006554	26.02689118	26.22470417	0.001728281
386922.3396	1166.118064	123.3711813	20.97927111	0.000560707	935.5945599
7097.862071	21.65632535	0.001125336	8503177.364	23.41964411	197.3457173
59211.87468	10052.71621	1850.202975	0.004365964	0.001593459	176.1041742
10.30718391	46.38520534	1269169.494	130.1502729	0.001334923	452.1043041
59211.87468	10052.71621	1850.202975	0.004365964	0.001593459	176.1041742
108.4214341	200.637552	11.0883451	164538.7808	2019.579319	541641.1111
0.214874278	12.5041051	0.022006554	26.02689118	26.22470417	0.001728281
59211.87468	10052.71621	1850.202975	0.004365964	0.001593459	176.1041742
0.00200755	2555.109525	0.044279049	0.015888065	792840.1904	0.086691829
0.15580288	413430.0189	68.20551738	0.000347322	1807781.746	2847349.069
0.15580288	413430.0189	68.20551738	0.000347322	1807781.746	2847349.069
173.8163838	2312.472214	442.8152069	876.1962988	9.995255165	279547.1921
108.4214341	200.637552	11.0883451	164538.7808	2019.579319	541641.1111
0.214874278	12.5041051	0.022006554	26.02689118	26.22470417	0.001728281
59211.87468	10052.71621	1850.202975	0.004365964	0.001593459	176.1041742
0.008732014	170.6406387	0.000842577	0.007461689	0.002723315	66.30174889
59211.87468	10052.71621	1850.202975	0.004365964	0.001593459	176.1041742
0.15580288	413430.0189	68.20551738	0.000347322	1807781.746	2847349.069
0.000704862	1.20139047	410.0019847	219.6530538	6.447011111	743.0723488
0.000979891	9952.926727	31.44350634	0.007755013	1012.918964	52.36532071
4429.448416	1390.450894	2.418900266	31.11679763	137.5906427	1559.604734
108.4214341	200.637552	11.0883451	164538.7808	2019.579319	541641.1111
59211.87468	10052.71621	1850.202975	0.004365964	0.001593459	176.1041742
0.00200755	2555.109525	0.044279049	0.015888065	792840.1904	0.086691829

0.00200755	2555.109525	0.044279049	0.015888065	792840.1904	0.086691829
108.4214341	200.637552	11.0883451	164538.7808	2019.579319	541641.1111
59211.87468	10052.71621	1850.202975	0.004365964	0.001593459	176.1041742
0.008732014	170.6406387	0.000842577	0.007461689	0.002723315	66.30174889
59211.87468	10052.71621	1850.202975	0.004365964	0.001593459	176.1041742
59211.87468	10052.71621	1850.202975	0.004365964	0.001593459	176.1041742
0.00200755	2555.109525	0.044279049	0.015888065	792840.1904	0.086691829
0.000979891	9952.926727	31.44350634	0.007755013	1012.918964	52.36532071
108.4214341	200.637552	11.0883451	164538.7808	2019.579319	541641.1111
0.000704862	1.20139047	410.0019847	219.6530538	6.447011111	743.0723488
6.644190091	0.000207344	1.429212492	0.000386378	0.000141018	67.98780398
0.852943194	0.001249056	125.3972904	0.009572251	18.62968355	113423.7866
59211.87468	10052.71621	1850.202975	0.004365964	0.001593459	176.1041742
0.00200755	2555.109525	0.044279049	0.015888065	792840.1904	0.086691829
6.644190091	0.000207344	1.429212492	0.000386378	0.000141018	67.98780398
0.134488121	0.220479148	1782.48211	4518.051803	262821.2282	0.448635626
0.15580288	413430.0189	68.20551738	0.000347322	1807781.746	2847349.069
265807.5481	532053037	75.47818323	12.09862041	805.9681736	0.469246636
0.000979891	9952.926727	31.44350634	0.007755013	1012.918964	52.36532071
0.15580288	413430.0189	68.20551738	0.000347322	1807781.746	2847349.069
0.130456738	22.40148469	0.629752132	5.150075028	0.004913846	679.5452071
4487.45971	6.258662585	0.004241355	25.95868285	0.012502668	28.44240761
0.002392787	0.002471021	0.002138362	86.80309912	0.006911452	1318.899463
55.77654081	51.25832249	41967.41926	2355584.738	469.0155301	850.067247
108.4214341	200.637552	11.0883451	164538.7808	2019.579319	541641.1111
386922.3396	1166.118064	123.3711813	20.97927111	0.000560707	935.5945599
0.130456738	22.40148469	0.629752132	5.150075028	0.004913846	679.5452071
7097.862071	21.65632535	0.001125336	8503177.364	23.41964411	197.3457173
4116.526367	26379051.1	11.66451751	65376.39617	44939.91948	0.328534252
0.008732014	170.6406387	0.000842577	0.007461689	0.002723315	66.30174889
0.000704862	1.20139047	410.0019847	219.6530538	6.447011111	743.0723488
7097.862071	21.65632535	0.001125336	8503177.364	23.41964411	197.3457173
108.4214341	200.637552	11.0883451	164538.7808	2019.579319	541641.1111
0.002392787	0.002471021	0.002138362	86.80309912	0.006911452	1318.899463
173.8163838	2312.472214	442.8152069	876.1962988	9.995255165	279547.1921
108.4214341	200.637552	11.0883451	164538.7808	2019.579319	541641.1111
4429.448416	1390.450894	2.418900266	31.11679763	137.5906427	1559.604734
0.15580288	413430.0189	68.20551738	0.000347322	1807781.746	2847349.069
108.4214341	200.637552	11.0883451	164538.7808	2019.579319	541641.1111
108.4214341	200.637552	11.0883451	164538.7808	2019.579319	541641.1111
0.134488121	0.220479148	1782.48211	4518.051803	262821.2282	0.448635626

59211.87468	10052.71621	1850.202975	0.004365964	0.001593459	176.1041742
1.585550628	23.17957111	8.792781472	192.1439536	21.46622391	45894.84745
108.4214341	200.637552	11.0883451	164538.7808	2019.579319	541641.1111
0.00200755	2555.109525	0.044279049	0.015888065	792840.1904	0.086691829
0.000704862	1.20139047	410.0019847	219.6530538	6.447011111	743.0723488
10.30718391	46.38520534	1269169.494	130.1502729	0.001334923	452.1043041
0.15580288	413430.0189	68.20551738	0.000347322	1807781.746	2847349.069
108.4214341	200.637552	11.0883451	164538.7808	2019.579319	541641.1111
0.00200755	2555.109525	0.044279049	0.015888065	792840.1904	0.086691829
0.214874278	12.5041051	0.022006554	26.02689118	26.22470417	0.001728281
108.4214341	200.637552	11.0883451	164538.7808	2019.579319	541641.1111
59211.87468	10052.71621	1850.202975	0.004365964	0.001593459	176.1041742
7.124341844	804.3862342	3430.738433	57.89850054	688.2011452	5.41277124
0.00200755	2555.109525	0.044279049	0.015888065	792840.1904	0.086691829
108.4214341	200.637552	11.0883451	164538.7808	2019.579319	541641.1111
108.4214341	200.637552	11.0883451	164538.7808	2019.579319	541641.1111
0.214874278	12.5041051	0.022006554	26.02689118	26.22470417	0.001728281
0.00200755	2555.109525	0.044279049	0.015888065	792840.1904	0.086691829
265807.5481	532053037	75.47818323	12.09862041	805.9681736	0.469246636
4487.45971	6.258662585	0.004241355	25.95868285	0.012502668	28.44240761
0.002392787	0.002471021	0.002138362	86.80309912	0.006911452	1318.899463
108.4214341	200.637552	11.0883451	164538.7808	2019.579319	541641.1111
108.4214341	200.637552	11.0883451	164538.7808	2019.579319	541641.1111
0.000979891	9952.926727	31.44350634	0.007755013	1012.918964	52.36532071
0.130456738	22.40148469	0.629752132	5.150075028	0.004913846	679.5452071
0.161350568	53.25262734	4.46E-05	1.110009499	0.003486747	0.002153378
6.644190091	0.000207344	1.429212492	0.000386378	0.000141018	67.98780398
0.000965308	2.558042527	8.081052703	7033.539162	54422.90696	417.0029921
0.00200755	2555.109525	0.044279049	0.015888065	792840.1904	0.086691829
0.15580288	413430.0189	68.20551738	0.000347322	1807781.746	2847349.069
7097.862071	21.65632535	0.001125336	8503177.364	23.41964411	197.3457173
59211.87468	10052.71621	1850.202975	0.004365964	0.001593459	176.1041742
161500.6741	33.9454569	22.14867525	112.5713209	46.91761774	0.017789605
108.4214341	200.637552	11.0883451	164538.7808	2019.579319	541641.1111
0.00200755	2555.109525	0.044279049	0.015888065	792840.1904	0.086691829
59211.87468	10052.71621	1850.202975	0.004365964	0.001593459	176.1041742
6.644190091	0.000207344	1.429212492	0.000386378	0.000141018	67.98780398
1516.819221	1.119040896	3.505037531	2.790089432	4.312795428	83.73719396
108.4214341	200.637552	11.0883451	164538.7808	2019.579319	541641.1111
108.4214341	200.637552	11.0883451	164538.7808	2019.579319	541641.1111
108.4214341	200.637552	11.0883451	164538.7808	2019.579319	541641.1111

0.000704862	1.20139047	410.0019847	219.6530538	6.447011111	743.0723488
6455.737782	32.00136959	98.01578473	78.38941417	47.3651068	1111.206106
59211.87468	10052.71621	1850.202975	0.004365964	0.001593459	176.1041742
108.4214341	200.637552	11.0883451	164538.7808	2019.579319	541641.1111
173.8163838	2312.472214	442.8152069	876.1962988	9.995255165	279547.1921
59211.87468	10052.71621	1850.202975	0.004365964	0.001593459	176.1041742
6.644190091	0.000207344	1.429212492	0.000386378	0.000141018	67.98780398
7097.862071	21.65632535	0.001125336	8503177.364	23.41964411	197.3457173
161500.6741	33.9454569	22.14867525	112.5713209	46.91761774	0.017789605
28.15890429	17.26754598	9.142346424	0.012909416	3040.886627	0.04266276
6455.737782	32.00136959	98.01578473	78.38941417	47.3651068	1111.206106
6455.737782	32.00136959	98.01578473	78.38941417	47.3651068	1111.206106
6.644190091	0.000207344	1.429212492	0.000386378	0.000141018	67.98780398
0.000704862	1.20139047	410.0019847	219.6530538	6.447011111	743.0723488
108.4214341	200.637552	11.0883451	164538.7808	2019.579319	541641.1111
0.00200755	2555.109525	0.044279049	0.015888065	792840.1904	0.086691829
108.4214341	200.637552	11.0883451	164538.7808	2019.579319	541641.1111
0.00200755	2555.109525	0.044279049	0.015888065	792840.1904	0.086691829
59211.87468	10052.71621	1850.202975	0.004365964	0.001593459	176.1041742
10.30718391	46.38520534	1269169.494	130.1502729	0.001334923	452.1043041
265807.5481	532053037	75.47818323	12.09862041	805.9681736	0.469246636
62392.68191	4.694219831	179886.3683	0.001197696	318.6963706	166.896168
0.00200755	2555.109525	0.044279049	0.015888065	792840.1904	0.086691829
0.00200755	2555.109525	0.044279049	0.015888065	792840.1904	0.086691829
6455.737782	32.00136959	98.01578473	78.38941417	47.3651068	1111.206106
386922.3396	1166.118064	123.3711813	20.97927111	0.000560707	935.5945599
0.00200755	2555.109525	0.044279049	0.015888065	792840.1904	0.086691829
0.00200755	2555.109525	0.044279049	0.015888065	792840.1904	0.086691829
0.000979891	9952.926727	31.44350634	0.007755013	1012.918964	52.36532071
486.4054891	550.1680199	52.72873241	2140.935077	3244.630204	0.033449865
0.000979891	9952.926727	31.44350634	0.007755013	1012.918964	52.36532071
0.000979891	9952.926727	31.44350634	0.007755013	1012.918964	52.36532071
59211.87468	10052.71621	1850.202975	0.004365964	0.001593459	176.1041742
0.130456738	22.40148469	0.629752132	5.150075028	0.004913846	679.5452071
6.644190091	0.000207344	1.429212492	0.000386378	0.000141018	67.98780398
0.00200755	2555.109525	0.044279049	0.015888065	792840.1904	0.086691829
0.002392787	0.002471021	0.002138362	86.80309912	0.006911452	1318.899463
173.8163838	2312.472214	442.8152069	876.1962988	9.995255165	279547.1921
7097.862071	21.65632535	0.001125336	8503177.364	23.41964411	197.3457173
0.00200755	2555.109525	0.044279049	0.015888065	792840.1904	0.086691829
108.4214341	200.637552	11.0883451	164538.7808	2019.579319	541641.1111

6455.737782	32.00136959	98.01578473	78.38941417	47.3651068	1111.206106
0.00200755	2555.109525	0.044279049	0.015888065	792840.1904	0.086691829
0.130456738	22.40148469	0.629752132	5.150075028	0.004913846	679.5452071
1516.819221	1.119040896	3.505037531	2.790089432	4.312795428	83.73719396
59211.87468	10052.71621	1850.202975	0.004365964	0.001593459	176.1041742
0.000704862	1.20139047	410.0019847	219.6530538	6.447011111	743.0723488
6455.737782	32.00136959	98.01578473	78.38941417	47.3651068	1111.206106
173.8163838	2312.472214	442.8152069	876.1962988	9.995255165	279547.1921
386922.3396	1166.118064	123.3711813	20.97927111	0.000560707	935.5945599
386922.3396	1166.118064	123.3711813	20.97927111	0.000560707	935.5945599
10.30718391	46.38520534	1269169.494	130.1502729	0.001334923	452.1043041
0.000979891	9952.926727	31.44350634	0.007755013	1012.918964	52.36532071
59211.87468	10052.71621	1850.202975	0.004365964	0.001593459	176.1041742
486.4054891	550.1680199	52.72873241	2140.935077	3244.630204	0.033449865
6.644190091	0.000207344	1.429212492	0.000386378	0.000141018	67.98780398
4429.448416	1390.450894	2.418900266	31.11679763	137.5906427	1559.604734
6.644190091	0.000207344	1.429212492	0.000386378	0.000141018	67.98780398
7097.862071	21.65632535	0.001125336	8503177.364	23.41964411	197.3457173
108.4214341	200.637552	11.0883451	164538.7808	2019.579319	541641.1111
173.8163838	2312.472214	442.8152069	876.1962988	9.995255165	279547.1921
55.77654081	51.25832249	41967.41926	2355584.738	469.0155301	850.067247
59211.87468	10052.71621	1850.202975	0.004365964	0.001593459	176.1041742
173.8163838	2312.472214	442.8152069	876.1962988	9.995255165	279547.1921
4487.45971	6.258662585	0.004241355	25.95868285	0.012502668	28.44240761
108.4214341	200.637552	11.0883451	164538.7808	2019.579319	541641.1111
0.000704862	1.20139047	410.0019847	219.6530538	6.447011111	743.0723488
0.214874278	12.5041051	0.022006554	26.02689118	26.22470417	0.001728281
0.00200755	2555.109525	0.044279049	0.015888065	792840.1904	0.086691829
7097.862071	21.65632535	0.001125336	8503177.364	23.41964411	197.3457173
632.5494359	810.1076369	13220.25995	6589.651217	719.7321552	0.171411922
0.000979891	9952.926727	31.44350634	0.007755013	1012.918964	52.36532071
0.15580288	413430.0189	68.20551738	0.000347322	1807781.746	2847349.069
4429.448416	1390.450894	2.418900266	31.11679763	137.5906427	1559.604734
59211.87468	10052.71621	1850.202975	0.004365964	0.001593459	176.1041742
0.852943194	0.001249056	125.3972904	0.009572251	18.62968355	113423.7866
7097.862071	21.65632535	0.001125336	8503177.364	23.41964411	197.3457173
0.00200755	2555.109525	0.044279049	0.015888065	792840.1904	0.086691829
108.4214341	200.637552	11.0883451	164538.7808	2019.579319	541641.1111
7097.862071	21.65632535	0.001125336	8503177.364	23.41964411	197.3457173
108.4214341	200.637552	11.0883451	164538.7808	2019.579319	541641.1111
10.30718391	46.38520534	1269169.494	130.1502729	0.001334923	452.1043041

265807.5481	532053037	75.47818323	12.09862041	805.9681736	0.469246636
6.644190091	0.000207344	1.429212492	0.000386378	0.000141018	67.98780398
486.4054891	550.1680199	52.72873241	2140.935077	3244.630204	0.033449865
7097.862071	21.65632535	0.001125336	8503177.364	23.41964411	197.3457173
0.000704862	1.20139047	410.0019847	219.6530538	6.447011111	743.0723488
6455.737782	32.00136959	98.01578473	78.38941417	47.3651068	1111.206106
108.4214341	200.637552	11.0883451	164538.7808	2019.579319	541641.1111
59211.87468	10052.71621	1850.202975	0.004365964	0.001593459	176.1041742
59211.87468	10052.71621	1850.202975	0.004365964	0.001593459	176.1041742
1.585550628	23.17957111	8.792781472	192.1439536	21.46622391	45894.84745
4116.526367	26379051.1	11.66451751	65376.39617	44939.91948	0.328534252
7097.862071	21.65632535	0.001125336	8503177.364	23.41964411	197.3457173
108.4214341	200.637552	11.0883451	164538.7808	2019.579319	541641.1111
0.00200755	2555.109525	0.044279049	0.015888065	792840.1904	0.086691829
59211.87468	10052.71621	1850.202975	0.004365964	0.001593459	176.1041742
0.130456738	22.40148469	0.629752132	5.150075028	0.004913846	679.5452071
0.000704862	1.20139047	410.0019847	219.6530538	6.447011111	743.0723488
59211.87468	10052.71621	1850.202975	0.004365964	0.001593459	176.1041742
1.585550628	23.17957111	8.792781472	192.1439536	21.46622391	45894.84745
0.15580288	413430.0189	68.20551738	0.000347322	1807781.746	2847349.069
59211.87468	10052.71621	1850.202975	0.004365964	0.001593459	176.1041742
6.644190091	0.000207344	1.429212492	0.000386378	0.000141018	67.98780398
7097.862071	21.65632535	0.001125336	8503177.364	23.41964411	197.3457173
7097.862071	21.65632535	0.001125336	8503177.364	23.41964411	197.3457173
0.852943194	0.001249056	125.3972904	0.009572251	18.62968355	113423.7866
486.4054891	550.1680199	52.72873241	2140.935077	3244.630204	0.033449865
486.4054891	550.1680199	52.72873241	2140.935077	3244.630204	0.033449865
7097.862071	21.65632535	0.001125336	8503177.364	23.41964411	197.3457173
1147.454244	3174.217162	16.9213751	1098.70442	2526.74145	2599.907783
10.30718391	46.38520534	1269169.494	130.1502729	0.001334923	452.1043041
108.4214341	200.637552	11.0883451	164538.7808	2019.579319	541641.1111
173.8163838	2312.472214	442.8152069	876.1962988	9.995255165	279547.1921
0.161350568	53.25262734	4.46E-05	1.110009499	0.003486747	0.002153378
0.008732014	170.6406387	0.000842577	0.007461689	0.002723315	66.30174889
1.585550628	23.17957111	8.792781472	192.1439536	21.46622391	45894.84745
0.161350568	53.25262734	4.46E-05	1.110009499	0.003486747	0.002153378
59211.87468	10052.71621	1850.202975	0.004365964	0.001593459	176.1041742
0.000965308	2.558042527	8.081052703	7033.539162	54422.90696	417.0029921
108.4214341	200.637552	11.0883451	164538.7808	2019.579319	541641.1111
0.000965308	2.558042527	8.081052703	7033.539162	54422.90696	417.0029921
108.4214341	200.637552	11.0883451	164538.7808	2019.579319	541641.1111

59211.87468	10052.71621	1850.202975	0.004365964	0.001593459	176.1041742
59211.87468	10052.71621	1850.202975	0.004365964	0.001593459	176.1041742
108.4214341	200.637552	11.0883451	164538.7808	2019.579319	541641.1111
59211.87468	10052.71621	1850.202975	0.004365964	0.001593459	176.1041742
4429.448416	1390.450894	2.418900266	31.11679763	137.5906427	1559.604734
108.4214341	200.637552	11.0883451	164538.7808	2019.579319	541641.1111
108.4214341	200.637552	11.0883451	164538.7808	2019.579319	541641.1111
62392.68191	4.694219831	179886.3683	0.001197696	318.6963706	166.896168
28.15890429	17.26754598	9.142346424	0.012909416	3040.886627	0.04266276
7.124341844	804.3862342	3430.738433	57.89850054	688.2011452	5.41277124
4116.526367	26379051.1	11.66451751	65376.39617	44939.91948	0.328534252
0.15580288	413430.0189	68.20551738	0.000347322	1807781.746	2847349.069
8877.672958	0.001017116	0.004604691	2.15924121	207.7786831	1924.755597
55.77654081	51.25832249	41967.41926	2355584.738	469.0155301	850.067247
1147.454244	3174.217162	16.9213751	1098.70442	2526.74145	2599.907783
7097.862071	21.65632535	0.001125336	8503177.364	23.41964411	197.3457173
6.644190091	0.000207344	1.429212492	0.000386378	0.000141018	67.98780398
59211.87468	10052.71621	1850.202975	0.004365964	0.001593459	176.1041742
173.8163838	2312.472214	442.8152069	876.1962988	9.995255165	279547.1921
386922.3396	1166.118064	123.3711813	20.97927111	0.000560707	935.5945599
59211.87468	10052.71621	1850.202975	0.004365964	0.001593459	176.1041742
4429.448416	1390.450894	2.418900266	31.11679763	137.5906427	1559.604734
0.000979891	9952.926727	31.44350634	0.007755013	1012.918964	52.36532071
59211.87468	10052.71621	1850.202975	0.004365964	0.001593459	176.1041742
108.4214341	200.637552	11.0883451	164538.7808	2019.579319	541641.1111
386922.3396	1166.118064	123.3711813	20.97927111	0.000560707	935.5945599
0.00200755	2555.109525	0.044279049	0.015888065	792840.1904	0.086691829
1147.454244	3174.217162	16.9213751	1098.70442	2526.74145	2599.907783
8877.672958	0.001017116	0.004604691	2.15924121	207.7786831	1924.755597
173.8163838	2312.472214	442.8152069	876.1962988	9.995255165	279547.1921
7097.862071	21.65632535	0.001125336	8503177.364	23.41964411	197.3457173
0.000979891	9952.926727	31.44350634	0.007755013	1012.918964	52.36532071
0.000704862	1.20139047	410.0019847	219.6530538	6.447011111	743.0723488
4429.448416	1390.450894	2.418900266	31.11679763	137.5906427	1559.604734
6.644190091	0.000207344	1.429212492	0.000386378	0.000141018	67.98780398
59211.87468	10052.71621	1850.202975	0.004365964	0.001593459	176.1041742
7097.862071	21.65632535	0.001125336	8503177.364	23.41964411	197.3457173
0.15580288	413430.0189	68.20551738	0.000347322	1807781.746	2847349.069
0.000979891	9952.926727	31.44350634	0.007755013	1012.918964	52.36532071
0.00200755	2555.109525	0.044279049	0.015888065	792840.1904	0.086691829
10.30718391	46.38520534	1269169.494	130.1502729	0.001334923	452.1043041

0.130456738	22.40148469	0.629752132	5.150075028	0.004913846	679.5452071
108.4214341	200.637552	11.0883451	164538.7808	2019.579319	541641.1111
108.4214341	200.637552	11.0883451	164538.7808	2019.579319	541641.1111
486.4054891	550.1680199	52.72873241	2140.935077	3244.630204	0.033449865
0.00200755	2555.109525	0.044279049	0.015888065	792840.1904	0.086691829
0.852943194	0.001249056	125.3972904	0.009572251	18.62968355	113423.7866
59211.87468	10052.71621	1850.202975	0.004365964	0.001593459	176.1041742
59211.87468	10052.71621	1850.202975	0.004365964	0.001593459	176.1041742
0.002392787	0.002471021	0.002138362	86.80309912	0.006911452	1318.899463
108.4214341	200.637552	11.0883451	164538.7808	2019.579319	541641.1111
59211.87468	10052.71621	1850.202975	0.004365964	0.001593459	176.1041742
7097.862071	21.65632535	0.001125336	8503177.364	23.41964411	197.3457173
173.8163838	2312.472214	442.8152069	876.1962988	9.995255165	279547.1921
0.000979891	9952.926727	31.44350634	0.007755013	1012.918964	52.36532071
59211.87468	10052.71621	1850.202975	0.004365964	0.001593459	176.1041742
0.00200755	2555.109525	0.044279049	0.015888065	792840.1904	0.086691829
59211.87468	10052.71621	1850.202975	0.004365964	0.001593459	176.1041742
0.000979891	9952.926727	31.44350634	0.007755013	1012.918964	52.36532071
0.000965308	2.558042527	8.081052703	7033.539162	54422.90696	417.0029921
7097.862071	21.65632535	0.001125336	8503177.364	23.41964411	197.3457173
173.8163838	2312.472214	442.8152069	876.1962988	9.995255165	279547.1921
62392.68191	4.694219831	179886.3683	0.001197696	318.6963706	166.896168
6.644190091	0.000207344	1.429212492	0.000386378	0.000141018	67.98780398
0.00200755	2555.109525	0.044279049	0.015888065	792840.1904	0.086691829
0.000979891	9952.926727	31.44350634	0.007755013	1012.918964	52.36532071
0.572194492	128.43259	39.85754938	904776.6251	59.50828257	0.01595297
386922.3396	1166.118064	123.3711813	20.97927111	0.000560707	935.5945599
59211.87468	10052.71621	1850.202975	0.004365964	0.001593459	176.1041742
0.00200755	2555.109525	0.044279049	0.015888065	792840.1904	0.086691829
6455.737782	32.00136959	98.01578473	78.38941417	47.3651068	1111.206106
108.4214341	200.637552	11.0883451	164538.7808	2019.579319	541641.1111
59211.87468	10052.71621	1850.202975	0.004365964	0.001593459	176.1041742
28.15890429	17.26754598	9.142346424	0.012909416	3040.886627	0.04266276
59211.87468	10052.71621	1850.202975	0.004365964	0.001593459	176.1041742
0.000704862	1.20139047	410.0019847	219.6530538	6.447011111	743.0723488
0.000979891	9952.926727	31.44350634	0.007755013	1012.918964	52.36532071
7.124341844	804.3862342	3430.738433	57.89850054	688.2011452	5.41277124
10.30718391	46.38520534	1269169.494	130.1502729	0.001334923	452.1043041
486.4054891	550.1680199	52.72873241	2140.935077	3244.630204	0.033449865
10.30718391	46.38520534	1269169.494	130.1502729	0.001334923	452.1043041
0.214874278	12.5041051	0.022006554	26.02689118	26.22470417	0.001728281

0.00200755	2555.109525	0.044279049	0.015888065	792840.1904	0.086691829
0.000979891	9952.926727	31.44350634	0.007755013	1012.918964	52.36532071
10.30718391	46.38520534	1269169.494	130.1502729	0.001334923	452.1043041
6455.737782	32.00136959	98.01578473	78.38941417	47.3651068	1111.206106
0.00200755	2555.109525	0.044279049	0.015888065	792840.1904	0.086691829
59211.87468	10052.71621	1850.202975	0.004365964	0.001593459	176.1041742
55.77654081	51.25832249	41967.41926	2355584.738	469.0155301	850.067247
0.000704862	1.20139047	410.0019847	219.6530538	6.447011111	743.0723488
6455.737782	32.00136959	98.01578473	78.38941417	47.3651068	1111.206106
108.4214341	200.637552	11.0883451	164538.7808	2019.579319	541641.1111
0.000704862	1.20139047	410.0019847	219.6530538	6.447011111	743.0723488
0.000704862	1.20139047	410.0019847	219.6530538	6.447011111	743.0723488
7097.862071	21.65632535	0.001125336	8503177.364	23.41964411	197.3457173
2.279519937	21.00594094	0.000779967	6.889552441	0.002520951	4585.452414
59211.87468	10052.71621	1850.202975	0.004365964	0.001593459	176.1041742
0.002392787	0.002471021	0.002138362	86.80309912	0.006911452	1318.899463
59211.87468	10052.71621	1850.202975	0.004365964	0.001593459	176.1041742
59211.87468	10052.71621	1850.202975	0.004365964	0.001593459	176.1041742
108.4214341	200.637552	11.0883451	164538.7808	2019.579319	541641.1111
59211.87468	10052.71621	1850.202975	0.004365964	0.001593459	176.1041742
265807.5481	532053037	75.47818323	12.09862041	805.9681736	0.469246636
55.77654081	51.25832249	41967.41926	2355584.738	469.0155301	850.067247
108.4214341	200.637552	11.0883451	164538.7808	2019.579319	541641.1111
173.8163838	2312.472214	442.8152069	876.1962988	9.995255165	279547.1921
4487.45971	6.258662585	0.004241355	25.95868285	0.012502668	28.44240761
0.00200755	2555.109525	0.044279049	0.015888065	792840.1904	0.086691829
6.644190091	0.000207344	1.429212492	0.000386378	0.000141018	67.98780398
59211.87468	10052.71621	1850.202975	0.004365964	0.001593459	176.1041742
108.4214341	200.637552	11.0883451	164538.7808	2019.579319	541641.1111
6455.737782	32.00136959	98.01578473	78.38941417	47.3651068	1111.206106
108.4214341	200.637552	11.0883451	164538.7808	2019.579319	541641.1111
2.279519937	21.00594094	0.000779967	6.889552441	0.002520951	4585.452414
0.134488121	0.220479148	1782.48211	4518.051803	262821.2282	0.448635626
59211.87468	10052.71621	1850.202975	0.004365964	0.001593459	176.1041742
0.00200755	2555.109525	0.044279049	0.015888065	792840.1904	0.086691829
0.00200755	2555.109525	0.044279049	0.015888065	792840.1904	0.086691829
6.644190091	0.000207344	1.429212492	0.000386378	0.000141018	67.98780398
173.8163838	2312.472214	442.8152069	876.1962988	9.995255165	279547.1921
59211.87468	10052.71621	1850.202975	0.004365964	0.001593459	176.1041742
0.002392787	0.002471021	0.002138362	86.80309912	0.006911452	1318.899463
108.4214341	200.637552	11.0883451	164538.7808	2019.579319	541641.1111

0.00200755	2555.109525	0.044279049	0.015888065	792840.1904	0.086691829
0.000979891	9952.926727	31.44350634	0.007755013	1012.918964	52.36532071
486.4054891	550.1680199	52.72873241	2140.935077	3244.630204	0.033449865
0.000704862	1.20139047	410.0019847	219.6530538	6.447011111	743.0723488
0.00200755	2555.109525	0.044279049	0.015888065	792840.1904	0.086691829
6.644190091	0.000207344	1.429212492	0.000386378	0.000141018	67.98780398
0.002392787	0.002471021	0.002138362	86.80309912	0.006911452	1318.899463
59211.87468	10052.71621	1850.202975	0.004365964	0.001593459	176.1041742
6455.737782	32.00136959	98.01578473	78.38941417	47.3651068	1111.206106
0.00200755	2555.109525	0.044279049	0.015888065	792840.1904	0.086691829
59211.87468	10052.71621	1850.202975	0.004365964	0.001593459	176.1041742
59211.87468	10052.71621	1850.202975	0.004365964	0.001593459	176.1041742
108.4214341	200.637552	11.0883451	164538.7808	2019.579319	541641.1111
0.00200755	2555.109525	0.044279049	0.015888065	792840.1904	0.086691829
0.000979891	9952.926727	31.44350634	0.007755013	1012.918964	52.36532071
59211.87468	10052.71621	1850.202975	0.004365964	0.001593459	176.1041742
0.000979891	9952.926727	31.44350634	0.007755013	1012.918964	52.36532071
6455.737782	32.00136959	98.01578473	78.38941417	47.3651068	1111.206106
4429.448416	1390.450894	2.418900266	31.11679763	137.5906427	1559.604734
6455.737782	32.00136959	98.01578473	78.38941417	47.3651068	1111.206106
0.00200755	2555.109525	0.044279049	0.015888065	792840.1904	0.086691829
6455.737782	32.00136959	98.01578473	78.38941417	47.3651068	1111.206106
108.4214341	200.637552	11.0883451	164538.7808	2019.579319	541641.1111
0.134488121	0.220479148	1782.48211	4518.051803	262821.2282	0.448635626
0.000965308	2.558042527	8.081052703	7033.539162	54422.90696	417.0029921
59211.87468	10052.71621	1850.202975	0.004365964	0.001593459	176.1041742
7097.862071	21.65632535	0.001125336	8503177.364	23.41964411	197.3457173
0.000979891	9952.926727	31.44350634	0.007755013	1012.918964	52.36532071
486.4054891	550.1680199	52.72873241	2140.935077	3244.630204	0.033449865
0.000979891	9952.926727	31.44350634	0.007755013	1012.918964	52.36532071
0.00200755	2555.109525	0.044279049	0.015888065	792840.1904	0.086691829
0.000704862	1.20139047	410.0019847	219.6530538	6.447011111	743.0723488
0.134488121	0.220479148	1782.48211	4518.051803	262821.2282	0.448635626
486.4054891	550.1680199	52.72873241	2140.935077	3244.630204	0.033449865
6.644190091	0.000207344	1.429212492	0.000386378	0.000141018	67.98780398
7097.862071	21.65632535	0.001125336	8503177.364	23.41964411	197.3457173
6.644190091	0.000207344	1.429212492	0.000386378	0.000141018	67.98780398
0.00200755	2555.109525	0.044279049	0.015888065	792840.1904	0.086691829
0.214874278	12.5041051	0.022006554	26.02689118	26.22470417	0.001728281
59211.87468	10052.71621	1850.202975	0.004365964	0.001593459	176.1041742
0.00200755	2555.109525	0.044279049	0.015888065	792840.1904	0.086691829

7097.862071	21.65632535	0.001125336	8503177.364	23.41964411	197.3457173
59211.87468	10052.71621	1850.202975	0.004365964	0.001593459	176.1041742
108.4214341	200.637552	11.0883451	164538.7808	2019.579319	541641.1111
4487.45971	6.258662585	0.004241355	25.95868285	0.012502668	28.44240761
108.4214341	200.637552	11.0883451	164538.7808	2019.579319	541641.1111
108.4214341	200.637552	11.0883451	164538.7808	2019.579319	541641.1111
59211.87468	10052.71621	1850.202975	0.004365964	0.001593459	176.1041742
7097.862071	21.65632535	0.001125336	8503177.364	23.41964411	197.3457173
6.644190091	0.000207344	1.429212492	0.000386378	0.000141018	67.98780398
0.00200755	2555.109525	0.044279049	0.015888065	792840.1904	0.086691829
59211.87468	10052.71621	1850.202975	0.004365964	0.001593459	176.1041742
0.00200755	2555.109525	0.044279049	0.015888065	792840.1904	0.086691829
2.279519937	21.00594094	0.000779967	6.889552441	0.002520951	4585.452414
108.4214341	200.637552	11.0883451	164538.7808	2019.579319	541641.1111
108.4214341	200.637552	11.0883451	164538.7808	2019.579319	541641.1111
486.4054891	550.1680199	52.72873241	2140.935077	3244.630204	0.033449865
0.130456738	22.40148469	0.629752132	5.150075028	0.004913846	679.5452071
6.644190091	0.000207344	1.429212492	0.000386378	0.000141018	67.98780398
0.000979891	9952.926727	31.44350634	0.007755013	1012.918964	52.36532071
4116.526367	26379051.1	11.66451751	65376.39617	44939.91948	0.328534252
0.00200755	2555.109525	0.044279049	0.015888065	792840.1904	0.086691829
0.214874278	12.5041051	0.022006554	26.02689118	26.22470417	0.001728281
0.000704862	1.20139047	410.0019847	219.6530538	6.447011111	743.0723488
0.15580288	413430.0189	68.20551738	0.000347322	1807781.746	2847349.069
6455.737782	32.00136959	98.01578473	78.38941417	47.3651068	1111.206106
4487.45971	6.258662585	0.004241355	25.95868285	0.012502668	28.44240761
0.572194492	128.43259	39.85754938	904776.6251	59.50828257	0.01595297
386922.3396	1166.118064	123.3711813	20.97927111	0.000560707	935.5945599
8877.672958	0.001017116	0.004604691	2.15924121	207.7786831	1924.755597
0.00200755	2555.109525	0.044279049	0.015888065	792840.1904	0.086691829
6.644190091	0.000207344	1.429212492	0.000386378	0.000141018	67.98780398
7097.862071	21.65632535	0.001125336	8503177.364	23.41964411	197.3457173
632.5494359	810.1076369	13220.25995	6589.651217	719.7321552	0.171411922
108.4214341	200.637552	11.0883451	164538.7808	2019.579319	541641.1111
59211.87468	10052.71621	1850.202975	0.004365964	0.001593459	176.1041742
8877.672958	0.001017116	0.004604691	2.15924121	207.7786831	1924.755597
0.15580288	413430.0189	68.20551738	0.000347322	1807781.746	2847349.069
108.4214341	200.637552	11.0883451	164538.7808	2019.579319	541641.1111
108.4214341	200.637552	11.0883451	164538.7808	2019.579319	541641.1111
4116.526367	26379051.1	11.66451751	65376.39617	44939.91948	0.328534252
10.30718391	46.38520534	1269169.494	130.1502729	0.001334923	452.1043041

0.002392787	0.002471021	0.002138362	86.80309912	0.006911452	1318.899463
108.4214341	200.637552	11.0883451	164538.7808	2019.579319	541641.1111
1147.454244	3174.217162	16.9213751	1098.70442	2526.74145	2599.907783
108.4214341	200.637552	11.0883451	164538.7808	2019.579319	541641.1111
59211.87468	10052.71621	1850.202975	0.004365964	0.001593459	176.1041742
10.30718391	46.38520534	1269169.494	130.1502729	0.001334923	452.1043041
7097.862071	21.65632535	0.001125336	8503177.364	23.41964411	197.3457173
108.4214341	200.637552	11.0883451	164538.7808	2019.579319	541641.1111
10.30718391	46.38520534	1269169.494	130.1502729	0.001334923	452.1043041
0.000704862	1.20139047	410.0019847	219.6530538	6.447011111	743.0723488
108.4214341	200.637552	11.0883451	164538.7808	2019.579319	541641.1111
108.4214341	200.637552	11.0883451	164538.7808	2019.579319	541641.1111
0.000965308	2.558042527	8.081052703	7033.539162	54422.90696	417.0029921
0.00200755	2555.109525	0.044279049	0.015888065	792840.1904	0.086691829
0.002392787	0.002471021	0.002138362	86.80309912	0.006911452	1318.899463
0.00200755	2555.109525	0.044279049	0.015888065	792840.1904	0.086691829
108.4214341	200.637552	11.0883451	164538.7808	2019.579319	541641.1111
7097.862071	21.65632535	0.001125336	8503177.364	23.41964411	197.3457173
0.214874278	12.5041051	0.022006554	26.02689118	26.22470417	0.001728281
59211.87468	10052.71621	1850.202975	0.004365964	0.001593459	176.1041742
4487.45971	6.258662585	0.004241355	25.95868285	0.012502668	28.44240761
486.4054891	550.1680199	52.72873241	2140.935077	3244.630204	0.033449865
0.00200755	2555.109525	0.044279049	0.015888065	792840.1904	0.086691829
7097.862071	21.65632535	0.001125336	8503177.364	23.41964411	197.3457173
108.4214341	200.637552	11.0883451	164538.7808	2019.579319	541641.1111
108.4214341	200.637552	11.0883451	164538.7808	2019.579319	541641.1111
59211.87468	10052.71621	1850.202975	0.004365964	0.001593459	176.1041742
59211.87468	10052.71621	1850.202975	0.004365964	0.001593459	176.1041742
59211.87468	10052.71621	1850.202975	0.004365964	0.001593459	176.1041742
6455.737782	32.00136959	98.01578473	78.38941417	47.3651068	1111.206106
59211.87468	10052.71621	1850.202975	0.004365964	0.001593459	176.1041742
486.4054891	550.1680199	52.72873241	2140.935077	3244.630204	0.033449865
108.4214341	200.637552	11.0883451	164538.7808	2019.579319	541641.1111
108.4214341	200.637552	11.0883451	164538.7808	2019.579319	541641.1111
4487.45971	6.258662585	0.004241355	25.95868285	0.012502668	28.44240761
0.15580288	413430.0189	68.20551738	0.000347322	1807781.746	2847349.069
386922.3396	1166.118064	123.3711813	20.97927111	0.000560707	935.5945599
0.161350568	53.25262734	4.46E-05	1.110009499	0.003486747	0.002153378
55.77654081	51.25832249	41967.41926	2355584.738	469.0155301	850.067247
10.30718391	46.38520534	1269169.494	130.1502729	0.001334923	452.1043041
59211.87468	10052.71621	1850.202975	0.004365964	0.001593459	176.1041742

486.4054891	550.1680199	52.72873241	2140.935077	3244.630204	0.033449865
0.000704862	1.20139047	410.0019847	219.6530538	6.447011111	743.0723488
0.000704862	1.20139047	410.0019847	219.6530538	6.447011111	743.0723488
0.000704862	1.20139047	410.0019847	219.6530538	6.447011111	743.0723488
108.4214341	200.637552	11.0883451	164538.7808	2019.579319	541641.1111
59211.87468	10052.71621	1850.202975	0.004365964	0.001593459	176.1041742
108.4214341	200.637552	11.0883451	164538.7808	2019.579319	541641.1111
0.00200755	2555.109525	0.044279049	0.015888065	792840.1904	0.086691829
10.30718391	46.38520534	1269169.494	130.1502729	0.001334923	452.1043041
0.00200755	2555.109525	0.044279049	0.015888065	792840.1904	0.086691829
55.77654081	51.25832249	41967.41926	2355584.738	469.0155301	850.067247
59211.87468	10052.71621	1850.202975	0.004365964	0.001593459	176.1041742
108.4214341	200.637552	11.0883451	164538.7808	2019.579319	541641.1111
0.000979891	9952.926727	31.44350634	0.007755013	1012.918964	52.36532071
0.00200755	2555.109525	0.044279049	0.015888065	792840.1904	0.086691829
1.585550628	23.17957111	8.792781472	192.1439536	21.46622391	45894.84745
59211.87468	10052.71621	1850.202975	0.004365964	0.001593459	176.1041742
59211.87468	10052.71621	1850.202975	0.004365964	0.001593459	176.1041742
0.000979891	9952.926727	31.44350634	0.007755013	1012.918964	52.36532071
0.214874278	12.5041051	0.022006554	26.02689118	26.22470417	0.001728281
59211.87468	10052.71621	1850.202975	0.004365964	0.001593459	176.1041742
632.5494359	810.1076369	13220.25995	6589.651217	719.7321552	0.171411922
0.00200755	2555.109525	0.044279049	0.015888065	792840.1904	0.086691829
6.644190091	0.000207344	1.429212492	0.000386378	0.000141018	67.98780398
0.000979891	9952.926727	31.44350634	0.007755013	1012.918964	52.36532071
0.000965308	2.558042527	8.081052703	7033.539162	54422.90696	417.0029921
59211.87468	10052.71621	1850.202975	0.004365964	0.001593459	176.1041742
4429.448416	1390.450894	2.418900266	31.11679763	137.5906427	1559.604734
0.130456738	22.40148469	0.629752132	5.150075028	0.004913846	679.5452071
0.00200755	2555.109525	0.044279049	0.015888065	792840.1904	0.086691829
10.30718391	46.38520534	1269169.494	130.1502729	0.001334923	452.1043041
0.000704862	1.20139047	410.0019847	219.6530538	6.447011111	743.0723488
0.00200755	2555.109525	0.044279049	0.015888065	792840.1904	0.086691829
59211.87468	10052.71621	1850.202975	0.004365964	0.001593459	176.1041742
0.852943194	0.001249056	125.3972904	0.009572251	18.62968355	113423.7866
108.4214341	200.637552	11.0883451	164538.7808	2019.579319	541641.1111
0.002392787	0.002471021	0.002138362	86.80309912	0.006911452	1318.899463
7.124341844	804.3862342	3430.738433	57.89850054	688.2011452	5.41277124
59211.87468	10052.71621	1850.202975	0.004365964	0.001593459	176.1041742
108.4214341	200.637552	11.0883451	164538.7808	2019.579319	541641.1111
486.4054891	550.1680199	52.72873241	2140.935077	3244.630204	0.033449865

59211.87468	10052.71621	1850.202975	0.004365964	0.001593459	176.1041742
0.008732014	170.6406387	0.000842577	0.007461689	0.002723315	66.30174889
6.644190091	0.000207344	1.429212492	0.000386378	0.000141018	67.98780398
108.4214341	200.637552	11.0883451	164538.7808	2019.579319	541641.1111
59211.87468	10052.71621	1850.202975	0.004365964	0.001593459	176.1041742
0.00200755	2555.109525	0.044279049	0.015888065	792840.1904	0.086691829
0.00200755	2555.109525	0.044279049	0.015888065	792840.1904	0.086691829
0.00200755	2555.109525	0.044279049	0.015888065	792840.1904	0.086691829
59211.87468	10052.71621	1850.202975	0.004365964	0.001593459	176.1041742
108.4214341	200.637552	11.0883451	164538.7808	2019.579319	541641.1111
59211.87468	10052.71621	1850.202975	0.004365964	0.001593459	176.1041742
59211.87468	10052.71621	1850.202975	0.004365964	0.001593459	176.1041742
108.4214341	200.637552	11.0883451	164538.7808	2019.579319	541641.1111
1.585550628	23.17957111	8.792781472	192.1439536	21.46622391	45894.84745
0.852943194	0.001249056	125.3972904	0.009572251	18.62968355	113423.7866
0.00200755	2555.109525	0.044279049	0.015888065	792840.1904	0.086691829
59211.87468	10052.71621	1850.202975	0.004365964	0.001593459	176.1041742
108.4214341	200.637552	11.0883451	164538.7808	2019.579319	541641.1111
108.4214341	200.637552	11.0883451	164538.7808	2019.579319	541641.1111
0.214874278	12.5041051	0.022006554	26.02689118	26.22470417	0.001728281
108.4214341	200.637552	11.0883451	164538.7808	2019.579319	541641.1111
173.8163838	2312.472214	442.8152069	876.1962988	9.995255165	279547.1921
55.77654081	51.25832249	41967.41926	2355584.738	469.0155301	850.067247
59211.87468	10052.71621	1850.202975	0.004365964	0.001593459	176.1041742
10.30718391	46.38520534	1269169.494	130.1502729	0.001334923	452.1043041
59211.87468	10052.71621	1850.202975	0.004365964	0.001593459	176.1041742
59211.87468	10052.71621	1850.202975	0.004365964	0.001593459	176.1041742
0.572194492	128.43259	39.85754938	904776.6251	59.50828257	0.01595297
486.4054891	550.1680199	52.72873241	2140.935077	3244.630204	0.033449865
0.000704862	1.20139047	410.0019847	219.6530538	6.447011111	743.0723488
108.4214341	200.637552	11.0883451	164538.7808	2019.579319	541641.1111
0.130456738	22.40148469	0.629752132	5.150075028	0.004913846	679.5452071
4429.448416	1390.450894	2.418900266	31.11679763	137.5906427	1559.604734
0.15580288	413430.0189	68.20551738	0.000347322	1807781.746	2847349.069
0.00200755	2555.109525	0.044279049	0.015888065	792840.1904	0.086691829
108.4214341	200.637552	11.0883451	164538.7808	2019.579319	541641.1111
0.00200755	2555.109525	0.044279049	0.015888065	792840.1904	0.086691829
0.008732014	170.6406387	0.000842577	0.007461689	0.002723315	66.30174889
173.8163838	2312.472214	442.8152069	876.1962988	9.995255165	279547.1921
59211.87468	10052.71621	1850.202975	0.004365964	0.001593459	176.1041742
0.214874278	12.5041051	0.022006554	26.02689118	26.22470417	0.001728281

4429.448416	1390.450894	2.418900266	31.11679763	137.5906427	1559.604734
59211.87468	10052.71621	1850.202975	0.004365964	0.001593459	176.1041742
0.00200755	2555.109525	0.044279049	0.015888065	792840.1904	0.086691829
1147.454244	3174.217162	16.9213751	1098.70442	2526.74145	2599.907783
108.4214341	200.637552	11.0883451	164538.7808	2019.579319	541641.1111
0.00200755	2555.109525	0.044279049	0.015888065	792840.1904	0.086691829
0.00200755	2555.109525	0.044279049	0.015888065	792840.1904	0.086691829
28.15890429	17.26754598	9.142346424	0.012909416	3040.886627	0.04266276
0.214874278	12.5041051	0.022006554	26.02689118	26.22470417	0.001728281
0.000704862	1.20139047	410.0019847	219.6530538	6.447011111	743.0723488
108.4214341	200.637552	11.0883451	164538.7808	2019.579319	541641.1111
59211.87468	10052.71621	1850.202975	0.004365964	0.001593459	176.1041742
108.4214341	200.637552	11.0883451	164538.7808	2019.579319	541641.1111
55.77654081	51.25832249	41967.41926	2355584.738	469.0155301	850.067247
0.15580288	413430.0189	68.20551738	0.000347322	1807781.746	2847349.069
0.00200755	2555.109525	0.044279049	0.015888065	792840.1904	0.086691829
6.644190091	0.000207344	1.429212492	0.000386378	0.000141018	67.98780398
486.4054891	550.1680199	52.72873241	2140.935077	3244.630204	0.033449865
1516.819221	1.119040896	3.505037531	2.790089432	4.312795428	83.73719396
8877.672958	0.001017116	0.004604691	2.15924121	207.7786831	1924.755597
0.161350568	53.25262734	4.46E-05	1.110009499	0.003486747	0.002153378
0.002392787	0.002471021	0.002138362	86.80309912	0.006911452	1318.899463
0.000979891	9952.926727	31.44350634	0.007755013	1012.918964	52.36532071
0.000965308	2.558042527	8.081052703	7033.539162	54422.90696	417.0029921
59211.87468	10052.71621	1850.202975	0.004365964	0.001593459	176.1041742
108.4214341	200.637552	11.0883451	164538.7808	2019.579319	541641.1111
0.15580288	413430.0189	68.20551738	0.000347322	1807781.746	2847349.069
10.30718391	46.38520534	1269169.494	130.1502729	0.001334923	452.1043041
6.644190091	0.000207344	1.429212492	0.000386378	0.000141018	67.98780398
0.00200755	2555.109525	0.044279049	0.015888065	792840.1904	0.086691829
0.00200755	2555.109525	0.044279049	0.015888065	792840.1904	0.086691829
59211.87468	10052.71621	1850.202975	0.004365964	0.001593459	176.1041742
108.4214341	200.637552	11.0883451	164538.7808	2019.579319	541641.1111
486.4054891	550.1680199	52.72873241	2140.935077	3244.630204	0.033449865
1147.454244	3174.217162	16.9213751	1098.70442	2526.74145	2599.907783
5.003893849	2.341300122	14.36543789	0.001867833	938.2246245	299.9465697
59211.87468	10052.71621	1850.202975	0.004365964	0.001593459	176.1041742
108.4214341	200.637552	11.0883451	164538.7808	2019.579319	541641.1111
6455.737782	32.00136959	98.01578473	78.38941417	47.3651068	1111.206106
108.4214341	200.637552	11.0883451	164538.7808	2019.579319	541641.1111
7097.862071	21.65632535	0.001125336	8503177.364	23.41964411	197.3457173

0.000979891	9952.926727	31.44350634	0.007755013	1012.918964	52.36532071
0.00200755	2555.109525	0.044279049	0.015888065	792840.1904	0.086691829
486.4054891	550.1680199	52.72873241	2140.935077	3244.630204	0.033449865
6.644190091	0.000207344	1.429212492	0.000386378	0.000141018	67.98780398
55.77654081	51.25832249	41967.41926	2355584.738	469.0155301	850.067247
59211.87468	10052.71621	1850.202975	0.004365964	0.001593459	176.1041742
0.000704862	1.20139047	410.0019847	219.6530538	6.447011111	743.0723488
0.161350568	53.25262734	4.46E-05	1.110009499	0.003486747	0.002153378
0.15580288	413430.0189	68.20551738	0.000347322	1807781.746	2847349.069
7097.862071	21.65632535	0.001125336	8503177.364	23.41964411	197.3457173
6.644190091	0.000207344	1.429212492	0.000386378	0.000141018	67.98780398
0.572194492	128.43259	39.85754938	904776.6251	59.50828257	0.01595297
0.000704862	1.20139047	410.0019847	219.6530538	6.447011111	743.0723488
0.852943194	0.001249056	125.3972904	0.009572251	18.62968355	113423.7866
108.4214341	200.637552	11.0883451	164538.7808	2019.579319	541641.1111
0.000704862	1.20139047	410.0019847	219.6530538	6.447011111	743.0723488
108.4214341	200.637552	11.0883451	164538.7808	2019.579319	541641.1111
6.644190091	0.000207344	1.429212492	0.000386378	0.000141018	67.98780398
0.00200755	2555.109525	0.044279049	0.015888065	792840.1904	0.086691829
6.644190091	0.000207344	1.429212492	0.000386378	0.000141018	67.98780398
59211.87468	10052.71621	1850.202975	0.004365964	0.001593459	176.1041742
0.00200755	2555.109525	0.044279049	0.015888065	792840.1904	0.086691829
59211.87468	10052.71621	1850.202975	0.004365964	0.001593459	176.1041742
0.130456738	22.40148469	0.629752132	5.150075028	0.004913846	679.5452071
6.644190091	0.000207344	1.429212492	0.000386378	0.000141018	67.98780398
4429.448416	1390.450894	2.418900266	31.11679763	137.5906427	1559.604734
0.002392787	0.002471021	0.002138362	86.80309912	0.006911452	1318.899463
0.00200755	2555.109525	0.044279049	0.015888065	792840.1904	0.086691829
0.000979891	9952.926727	31.44350634	0.007755013	1012.918964	52.36532071
0.00200755	2555.109525	0.044279049	0.015888065	792840.1904	0.086691829
0.130456738	22.40148469	0.629752132	5.150075028	0.004913846	679.5452071
6.644190091	0.000207344	1.429212492	0.000386378	0.000141018	67.98780398
4116.526367	26379051.1	11.66451751	65376.39617	44939.91948	0.328534252
0.15580288	413430.0189	68.20551738	0.000347322	1807781.746	2847349.069
0.572194492	128.43259	39.85754938	904776.6251	59.50828257	0.01595297
0.15580288	413430.0189	68.20551738	0.000347322	1807781.746	2847349.069
59211.87468	10052.71621	1850.202975	0.004365964	0.001593459	176.1041742
5.003893849	2.341300122	14.36543789	0.001867833	938.2246245	299.9465697
6.644190091	0.000207344	1.429212492	0.000386378	0.000141018	67.98780398
10.30718391	46.38520534	1269169.494	130.1502729	0.001334923	452.1043041
173.8163838	2312.472214	442.8152069	876.1962988	9.995255165	279547.1921

5.003893849	2.341300122	14.36543789	0.001867833	938.2246245	299.9465697
0.000965308	2.558042527	8.081052703	7033.539162	54422.90696	417.0029921
108.4214341	200.637552	11.0883451	164538.7808	2019.579319	541641.1111
108.4214341	200.637552	11.0883451	164538.7808	2019.579319	541641.1111
59211.87468	10052.71621	1850.202975	0.004365964	0.001593459	176.1041742
4116.526367	26379051.1	11.66451751	65376.39617	44939.91948	0.328534252
6455.737782	32.00136959	98.01578473	78.38941417	47.3651068	1111.206106
12.81732419	0.000824651	0.142698834	135.390057	0.492581713	65.4100407
0.000979891	9952.926727	31.44350634	0.007755013	1012.918964	52.36532071
4116.526367	26379051.1	11.66451751	65376.39617	44939.91948	0.328534252
71724.35614	49.03588381	3.647385731	0.008117099	0.002962521	354.9515936
108.4214341	200.637552	11.0883451	164538.7808	2019.579319	541641.1111
486.4054891	550.1680199	52.72873241	2140.935077	3244.630204	0.033449865
7.124341844	804.3862342	3430.738433	57.89850054	688.2011452	5.41277124
2.279519937	21.00594094	0.000779967	6.889552441	0.002520951	4585.452414
4116.526367	26379051.1	11.66451751	65376.39617	44939.91948	0.328534252
173.8163838	2312.472214	442.8152069	876.1962988	9.995255165	279547.1921
6.644190091	0.000207344	1.429212492	0.000386378	0.000141018	67.98780398
108.4214341	200.637552	11.0883451	164538.7808	2019.579319	541641.1111
0.134488121	0.220479148	1782.48211	4518.051803	262821.2282	0.448635626
0.00200755	2555.109525	0.044279049	0.015888065	792840.1904	0.086691829
10.30718391	46.38520534	1269169.494	130.1502729	0.001334923	452.1043041
59211.87468	10052.71621	1850.202975	0.004365964	0.001593459	176.1041742
386922.3396	1166.118064	123.3711813	20.97927111	0.000560707	935.5945599
0.000704862	1.20139047	410.0019847	219.6530538	6.447011111	743.0723488
7097.862071	21.65632535	0.001125336	8503177.364	23.41964411	197.3457173
632.5494359	810.1076369	13220.25995	6589.651217	719.7321552	0.171411922
0.00200755	2555.109525	0.044279049	0.015888065	792840.1904	0.086691829
10.30718391	46.38520534	1269169.494	130.1502729	0.001334923	452.1043041
7097.862071	21.65632535	0.001125336	8503177.364	23.41964411	197.3457173
6.644190091	0.000207344	1.429212492	0.000386378	0.000141018	67.98780398
4116.526367	26379051.1	11.66451751	65376.39617	44939.91948	0.328534252
10.30718391	46.38520534	1269169.494	130.1502729	0.001334923	452.1043041
5.003893849	2.341300122	14.36543789	0.001867833	938.2246245	299.9465697
12.81732419	0.000824651	0.142698834	135.390057	0.492581713	65.4100407
4116.526367	26379051.1	11.66451751	65376.39617	44939.91948	0.328534252
108.4214341	200.637552	11.0883451	164538.7808	2019.579319	541641.1111
4429.448416	1390.450894	2.418900266	31.11679763	137.5906427	1559.604734
4116.526367	26379051.1	11.66451751	65376.39617	44939.91948	0.328534252
0.000704862	1.20139047	410.0019847	219.6530538	6.447011111	743.0723488
108.4214341	200.637552	11.0883451	164538.7808	2019.579319	541641.1111

0.00200755	2555.109525	0.044279049	0.015888065	792840.1904	0.086691829
0.134488121	0.220479148	1782.48211	4518.051803	262821.2282	0.448635626
7097.862071	21.65632535	0.001125336	8503177.364	23.41964411	197.3457173
0.852943194	0.001249056	125.3972904	0.009572251	18.62968355	113423.7866
55.77654081	51.25832249	41967.41926	2355584.738	469.0155301	850.067247
386922.3396	1166.118064	123.3711813	20.97927111	0.000560707	935.5945599
0.214874278	12.5041051	0.022006554	26.02689118	26.22470417	0.001728281
0.000704862	1.20139047	410.0019847	219.6530538	6.447011111	743.0723488
0.000704862	1.20139047	410.0019847	219.6530538	6.447011111	743.0723488
59211.87468	10052.71621	1850.202975	0.004365964	0.001593459	176.1041742
10.30718391	46.38520534	1269169.494	130.1502729	0.001334923	452.1043041
7097.862071	21.65632535	0.001125336	8503177.364	23.41964411	197.3457173
173.8163838	2312.472214	442.8152069	876.1962988	9.995255165	279547.1921
0.00200755	2555.109525	0.044279049	0.015888065	792840.1904	0.086691829
55.77654081	51.25832249	41967.41926	2355584.738	469.0155301	850.067247
59211.87468	10052.71621	1850.202975	0.004365964	0.001593459	176.1041742
0.00200755	2555.109525	0.044279049	0.015888065	792840.1904	0.086691829
161500.6741	33.9454569	22.14867525	112.5713209	46.91761774	0.017789605
28.15890429	17.26754598	9.142346424	0.012909416	3040.886627	0.04266276
55.77654081	51.25832249	41967.41926	2355584.738	469.0155301	850.067247
8877.672958	0.001017116	0.004604691	2.15924121	207.7786831	1924.755597
173.8163838	2312.472214	442.8152069	876.1962988	9.995255165	279547.1921
0.214874278	12.5041051	0.022006554	26.02689118	26.22470417	0.001728281
0.000979891	9952.926727	31.44350634	0.007755013	1012.918964	52.36532071
2.279519937	21.00594094	0.000779967	6.889552441	0.002520951	4585.452414
0.15580288	413430.0189	68.20551738	0.000347322	1807781.746	2847349.069
7097.862071	21.65632535	0.001125336	8503177.364	23.41964411	197.3457173
486.4054891	550.1680199	52.72873241	2140.935077	3244.630204	0.033449865
108.4214341	200.637552	11.0883451	164538.7808	2019.579319	541641.1111
1147.454244	3174.217162	16.9213751	1098.70442	2526.74145	2599.907783
1.585550628	23.17957111	8.792781472	192.1439536	21.46622391	45894.84745
0.00200755	2555.109525	0.044279049	0.015888065	792840.1904	0.086691829
173.8163838	2312.472214	442.8152069	876.1962988	9.995255165	279547.1921
7097.862071	21.65632535	0.001125336	8503177.364	23.41964411	197.3457173
7097.862071	21.65632535	0.001125336	8503177.364	23.41964411	197.3457173
8877.672958	0.001017116	0.004604691	2.15924121	207.7786831	1924.755597
10.30718391	46.38520534	1269169.494	130.1502729	0.001334923	452.1043041
5.003893849	2.341300122	14.36543789	0.001867833	938.2246245	299.9465697
0.852943194	0.001249056	125.3972904	0.009572251	18.62968355	113423.7866
0.000704862	1.20139047	410.0019847	219.6530538	6.447011111	743.0723488
7.124341844	804.3862342	3430.738433	57.89850054	688.2011452	5.41277124

71724.35614	49.03588381	3.647385731	0.008117099	0.002962521	354.9515936
0.00200755	2555.109525	0.044279049	0.015888065	792840.1904	0.086691829
486.4054891	550.1680199	52.72873241	2140.935077	3244.630204	0.033449865
0.000704862	1.20139047	410.0019847	219.6530538	6.447011111	743.0723488
0.00200755	2555.109525	0.044279049	0.015888065	792840.1904	0.086691829
0.000979891	9952.926727	31.44350634	0.007755013	1012.918964	52.36532071
4116.526367	26379051.1	11.66451751	65376.39617	44939.91948	0.328534252
10.30718391	46.38520534	1269169.494	130.1502729	0.001334923	452.1043041
173.8163838	2312.472214	442.8152069	876.1962988	9.995255165	279547.1921
59211.87468	10052.71621	1850.202975	0.004365964	0.001593459	176.1041742
10.30718391	46.38520534	1269169.494	130.1502729	0.001334923	452.1043041
108.4214341	200.637552	11.0883451	164538.7808	2019.579319	541641.1111
7.124341844	804.3862342	3430.738433	57.89850054	688.2011452	5.41277124
0.000704862	1.20139047	410.0019847	219.6530538	6.447011111	743.0723488
386922.3396	1166.118064	123.3711813	20.97927111	0.000560707	935.5945599
59211.87468	10052.71621	1850.202975	0.004365964	0.001593459	176.1041742
386922.3396	1166.118064	123.3711813	20.97927111	0.000560707	935.5945599
59211.87468	10052.71621	1850.202975	0.004365964	0.001593459	176.1041742
21	22	23	24	25	26
FAM20A-Hs01034070_m1	NEK9-Hs00929602_m1	NEK9-Hs00929594_m1	ABCC1-Hs01561504_m1	SORBS2-Hs01125202_m1	SORBS2-Hs00243432_m1

27	28	29	30	31	32
TMPRSS2-ERG fusion gene	ATP5O-Hs04272738_m1	DSCAM-Hs00242097_m1	ERG-Hs01573964_m1	ICOSLG-Hs00391287_m1	DOP1B-Hs01123288_m1
0.133545135	14.81474838	0.954382623	0.816672026	70.47771596	12.54537914
0.121285934	2.635308865	0.449446015	1.525343572	0.00061551	0.000423844
8.210068665	0.005664664	0.000444056	238552.8977	134967.0567	226.4122406
4.001507594	163.9665142	31.08392772	69.22260712	0.081499539	3368.95795
0.19965409	21.14430497	1.67268882	1.109772089	4.802826745	0.001162649
1.42026838	1220698.639	20.65450137	1.459440622	16053.76239	1980.842914
6.631193984	59.89776951	34.04886711	9.205227192	128.6438378	0.014167549
0.202723942	7.882093712	0.428855375	2.34E-06	0.000980914	0.000675464
0.530192157	12.72510199	1.535877044	0.729556036	14.01848456	42.17860326
0.281548313	22.21188935	3.217311863	0.949568787	54.2959258	9.893139302
1.28258075	252.0409852	22.29581319	2.111386677	0.011828006	0.008144847
4.001507594	163.9665142	31.08392772	69.22260712	0.081499539	3368.95795
3.305125836	54.8000689	7.727447262	254967.11	57.04860346	128.1980387
0.530192157	12.72510199	1.535877044	0.729556036	14.01848456	42.17860326
0.198290212	26927.02609	12.66450708	4551.577587	58.30864619	37.67715002
0.198586345	15.87802831	2.890298745	2.724429219	0.004153862	373.0913766
3.0192923	50.58908994	9.970378374	20267.99012	264.4445336	1333.878117
0.339861639	15.52648114	1.554771039	0.074396185	19.25433638	751.975998
8.210068665	0.005664664	0.000444056	238552.8977	134967.0567	226.4122406
3.083239954	72.4513816	46.03660834	0.012353032	0.052911977	0.036435554
0.198586345	15.87802831	2.890298745	2.724429219	0.004153862	373.0913766
0.55416361	55.59326508	6.367827129	26.00573749	81.36957731	91.41284164
4.586168467	188.9107512	34.5481433	5.559752013	719.0846225	3252.775753
1.28258075	252.0409852	22.29581319	2.111386677	0.011828006	0.008144847
0.19965409	21.14430497	1.67268882	1.109772089	4.802826745	0.001162649
4.586168467	188.9107512	34.5481433	5.559752013	719.0846225	3252.775753
0.281548313	22.21188935	3.217311863	0.949568787	54.2959258	9.893139302
1.28258075	252.0409852	22.29581319	2.111386677	0.011828006	0.008144847
0.198290212	26927.02609	12.66450708	4551.577587	58.30864619	37.67715002
0.530192157	12.72510199	1.535877044	0.729556036	14.01848456	42.17860326
0.133545135	14.81474838	0.954382623	0.816672026	70.47771596	12.54537914
6.407327969	0.041677675	10.15802274	38964.07411	0.026497539	695.2963467
0.198586345	15.87802831	2.890298745	2.724429219	0.004153862	373.0913766
1.42026838	1220698.639	20.65450137	1.459440622	16053.76239	1980.842914
0.365257503	0.000170526	1.497953968	6491.387136	0.003269318	20.14540996
0.281548313	22.21188935	3.217311863	0.949568787	54.2959258	9.893139302
0.55416361	55.59326508	6.367827129	26.00573749	81.36957731	91.41284164

4.586168467	188.9107512	34.5481433	5.559752013	719.0846225	3252.775753
1.42026838	1220698.639	20.65450137	1.459440622	16053.76239	1980.842914
1.706674617	13.9296059	15.57522978	0.0301036	80.19848051	125.9135185
0.198586345	15.87802831	2.890298745	2.724429219	0.004153862	373.0913766
0.281548313	22.21188935	3.217311863	0.949568787	54.2959258	9.893139302
1.42026838	1220698.639	20.65450137	1.459440622	16053.76239	1980.842914
6.407327969	0.041677675	10.15802274	38964.07411	0.026497539	695.2963467
3.083239954	72.4513816	46.03660834	0.012353032	0.052911977	0.036435554
0.19965409	21.14430497	1.67268882	1.109772089	4.802826745	0.001162649
1.706674617	13.9296059	15.57522978	0.0301036	80.19848051	125.9135185
0.19965409	21.14430497	1.67268882	1.109772089	4.802826745	0.001162649
8.210068665	0.005664664	0.000444056	238552.8977	134967.0567	226.4122406
0.55416361	55.59326508	6.367827129	26.00573749	81.36957731	91.41284164
0.148796703	6.230127017	1.518385671	7.463994701	27.70295678	90.50473091
54.42667484	0.005576344	367.0171513	10063105.29	6131.555202	372901.8002
1.707133388	77.06474695	16.04900246	40507.17965	5340475.134	346.9465131
3.124345615	63.10265755	14.79787763	0.010175583	265.0872199	185.2203281
1.334260566	14.20741876	8.479742795	0.434487256	102.6304732	766.7850473
8.464536659	135.1578262	41.57906082	0.021150193	1226.703634	1591.805388
4.114009411	36.50752938	10.49738	1.772448251	1599.451717	416.0128287
3.124345615	63.10265755	14.79787763	0.010175583	265.0872199	185.2203281
0.471954172	5.311692329	2.789233089	0.281020837	235.7729799	146.2289743
1.74678418	39.14156953	7.732681006	0.018863839	647.2189703	1.13207589
1.707133388	77.06474695	16.04900246	40507.17965	5340475.134	346.9465131
1.243063847	60.10687269	3.796971449	1264.884748	392.1088114	0.010231395
8.464536659	135.1578262	41.57906082	0.021150193	1226.703634	1591.805388
8.480593511	98.16995661	71707.18021	15.73872939	705.8751992	5071.954414
1.243063847	60.10687269	3.796971449	1264.884748	392.1088114	0.010231395
3.124345615	63.10265755	14.79787763	0.010175583	265.0872199	185.2203281
8.464536659	135.1578262	41.57906082	0.021150193	1226.703634	1591.805388
0.174482667	11.13005785	4.0172359	1.209150248	17.94286893	0.004377164
1.063885018	15.20224748	9.757105764	493.0510379	0.006138098	474.0337749
0.471954172	5.311692329	2.789233089	0.281020837	235.7729799	146.2289743
1.243063847	60.10687269	3.796971449	1264.884748	392.1088114	0.010231395
0.21894707	0.05667782	1.060917442	0.621035655	17.24141958	54.31318402
3.124345615	63.10265755	14.79787763	0.010175583	265.0872199	185.2203281
4.114009411	36.50752938	10.49738	1.772448251	1599.451717	416.0128287
1.243063847	60.10687269	3.796971449	1264.884748	392.1088114	0.010231395
3.124345615	63.10265755	14.79787763	0.010175583	265.0872199	185.2203281
1.243063847	60.10687269	3.796971449	1264.884748	392.1088114	0.010231395
1.780992145	128.7688743	7.582228822	4.900183346	322.1056636	205.9584277

4.188281104	124.9426824	6.682117485	1591.294328	26.60073769	1125.16697
54.42667484	0.005576344	367.0171513	10063105.29	6131.555202	372901.8002
1.707133388	77.06474695	16.04900246	40507.17965	5340475.134	346.9465131
1.334260566	14.20741876	8.479742795	0.434487256	102.6304732	766.7850473
6.974586009	0.015265453	0.169734463	1946.977745	8223.95308	98.71421058
0.471954172	5.311692329	2.789233089	0.281020837	235.7729799	146.2289743
1.780992145	128.7688743	7.582228822	4.900183346	322.1056636	205.9584277
1.348782543	27.52709678	6090.426505	15.99355277	66.96648553	838210.3701
0.471954172	5.311692329	2.789233089	0.281020837	235.7729799	146.2289743
1.36808232	14.32609198	0.013451001	23048.62822	168.9977706	23.46735703
0.21894707	0.05667782	1.060917442	0.621035655	17.24141958	54.31318402
0.104204786	4.424925002	0.000110169	4481.222881	16310.66312	0.018553817
8.464536659	135.1578262	41.57906082	0.021150193	1226.703634	1591.805388
3.124345615	63.10265755	14.79787763	0.010175583	265.0872199	185.2203281
8.464536659	135.1578262	41.57906082	0.021150193	1226.703634	1591.805388
1.243063847	60.10687269	3.796971449	1264.884748	392.1088114	0.010231395
1.243063847	60.10687269	3.796971449	1264.884748	392.1088114	0.010231395
1.243063847	60.10687269	3.796971449	1264.884748	392.1088114	0.010231395
1.243063847	60.10687269	3.796971449	1264.884748	392.1088114	0.010231395
1.243063847	60.10687269	3.796971449	1264.884748	392.1088114	0.010231395
0.524390548	59.31225537	4.226285114	266.706229	214.1999318	3741.798029
0.471954172	5.311692329	2.789233089	0.281020837	235.7729799	146.2289743
3.124345615	63.10265755	14.79787763	0.010175583	265.0872199	185.2203281
1.707133388	77.06474695	16.04900246	40507.17965	5340475.134	346.9465131
3.124345615	63.10265755	14.79787763	0.010175583	265.0872199	185.2203281
8.464536659	135.1578262	41.57906082	0.021150193	1226.703634	1591.805388
0.471954172	5.311692329	2.789233089	0.281020837	235.7729799	146.2289743
1.84655553	0.001356082	9.635827175	7.041910215	15.32397228	0.017902965
1.243063847	60.10687269	3.796971449	1264.884748	392.1088114	0.010231395
1.243063847	60.10687269	3.796971449	1264.884748	392.1088114	0.010231395
8.462505354	660.766597	17.70371273	20.19544547	0.064445396	0.044377546
0.304684186	22.32231372	17.26502338	0.00158146	2.163388742	4.980732837
1.84655553	0.001356082	9.635827175	7.041910215	15.32397228	0.017902965
8.464536659	135.1578262	41.57906082	0.021150193	1226.703634	1591.805388
1.429694041	0.001440843	11.47926338	6.058372034	35.35957098	284.4223853
1.74678418	39.14156953	7.732681006	0.018863839	647.2189703	1.13207589
0.148796703	6.230127017	1.518385671	7.463994701	27.70295678	90.50473091
1.348782543	27.52709678	6090.426505	15.99355277	66.96648553	838210.3701
1.334260566	14.20741876	8.479742795	0.434487256	102.6304732	766.7850473
8.464536659	135.1578262	41.57906082	0.021150193	1226.703634	1591.805388
1.348782543	27.52709678	6090.426505	15.99355277	66.96648553	838210.3701

1.243063847	60.10687269	3.796971449	1264.884748	392.1088114	0.010231395
2.663404946	26.28506589	14.15335703	2.1596973	0.037302351	0.025686657
8.464536659	135.1578262	41.57906082	0.021150193	1226.703634	1591.805388
1.74678418	39.14156953	7.732681006	0.018863839	647.2189703	1.13207589
3.124345615	63.10265755	14.79787763	0.010175583	265.0872199	185.2203281
8.464536659	135.1578262	41.57906082	0.021150193	1226.703634	1591.805388
1.707133388	77.06474695	16.04900246	40507.17965	5340475.134	346.9465131
2.663404946	26.28506589	14.15335703	2.1596973	0.037302351	0.025686657
8.464536659	135.1578262	41.57906082	0.021150193	1226.703634	1591.805388
3.124345615	63.10265755	14.79787763	0.010175583	265.0872199	185.2203281
1.243063847	60.10687269	3.796971449	1264.884748	392.1088114	0.010231395
4.114009411	36.50752938	10.49738	1.772448251	1599.451717	416.0128287
8.464536659	135.1578262	41.57906082	0.021150193	1226.703634	1591.805388
3.124345615	63.10265755	14.79787763	0.010175583	265.0872199	185.2203281
8.464536659	135.1578262	41.57906082	0.021150193	1226.703634	1591.805388
4.114009411	36.50752938	10.49738	1.772448251	1599.451717	416.0128287
8.464536659	135.1578262	41.57906082	0.021150193	1226.703634	1591.805388
0.931440765	45.3976929	7.476177884	126.2810675	0.009949876	263.6330236
1.84655553	0.001356082	9.635827175	7.041910215	15.32397228	0.017902965
0.21894707	0.05667782	1.060917442	0.621035655	17.24141958	54.31318402
0.170347797	6.407282377	2.216511787	260.8310627	9.115521429	31306.63106
0.524390548	59.31225537	4.226285114	266.706229	214.1999318	3741.798029
3.124345615	63.10265755	14.79787763	0.010175583	265.0872199	185.2203281
1.063885018	15.20224748	9.757105764	493.0510379	0.006138098	474.0337749
1.334260566	14.20741876	8.479742795	0.434487256	102.6304732	766.7850473
0.304684186	22.32231372	17.26502338	0.00158146	2.163388742	4.980732837
2.663404946	26.28506589	14.15335703	2.1596973	0.037302351	0.025686657
4.114009411	36.50752938	10.49738	1.772448251	1599.451717	416.0128287
0.601023963	76.60850563	9.383264999	23.63188596	0.045818851	462.6026003
0.170347797	6.407282377	2.216511787	260.8310627	9.115521429	31306.63106
3.124345615	63.10265755	14.79787763	0.010175583	265.0872199	185.2203281
1.780992145	128.7688743	7.582228822	4.900183346	322.1056636	205.9584277
2.663404946	26.28506589	14.15335703	2.1596973	0.037302351	0.025686657
3.124345615	63.10265755	14.79787763	0.010175583	265.0872199	185.2203281
1.334260566	14.20741876	8.479742795	0.434487256	102.6304732	766.7850473
1.243063847	60.10687269	3.796971449	1264.884748	392.1088114	0.010231395
8.464536659	135.1578262	41.57906082	0.021150193	1226.703634	1591.805388
54.42667484	0.005576344	367.0171513	10063105.29	6131.555202	372901.8002
3.124345615	63.10265755	14.79787763	0.010175583	265.0872199	185.2203281
3.124345615	63.10265755	14.79787763	0.010175583	265.0872199	185.2203281
4.114009411	36.50752938	10.49738	1.772448251	1599.451717	416.0128287

1.348782543	27.52709678	6090.426505	15.99355277	66.96648553	838210.3701
0.524390548	59.31225537	4.226285114	266.706229	214.1999318	3741.798029
18.78601156	2572385.756	490.3926503	56.41015292	447.3507743	842535.2186
4.45166709	30.81716963	20.27063196	34.7506403	0.03257597	0.035194761
1.427332548	126.8583124	6.880385283	0.00271409	0.021507298	2950.544493
0.21894707	0.05667782	1.060917442	0.621035655	17.24141958	54.31318402
2.663404946	26.28506589	14.15335703	2.1596973	0.037302351	0.025686657
0.471954172	5.311692329	2.789233089	0.281020837	235.7729799	146.2289743
1.74678418	39.14156953	7.732681006	0.018863839	647.2189703	1.13207589
1.84655553	0.001356082	9.635827175	7.041910215	15.32397228	0.017902965
0.170347797	6.407282377	2.216511787	260.8310627	9.115521429	31306.63106
1.780992145	128.7688743	7.582228822	4.900183346	322.1056636	205.9584277
1.243063847	60.10687269	3.796971449	1264.884748	392.1088114	0.010231395
1.243063847	60.10687269	3.796971449	1264.884748	392.1088114	0.010231395
0.471954172	5.311692329	2.789233089	0.281020837	235.7729799	146.2289743
1.243063847	60.10687269	3.796971449	1264.884748	392.1088114	0.010231395
1.243063847	60.10687269	3.796971449	1264.884748	392.1088114	0.010231395
1.243063847	60.10687269	3.796971449	1264.884748	392.1088114	0.010231395
1.243063847	60.10687269	3.796971449	1264.884748	392.1088114	0.010231395
0.471954172	5.311692329	2.789233089	0.281020837	235.7729799	146.2289743
8.464536659	135.1578262	41.57906082	0.021150193	1226.703634	1591.805388
1.243063847	60.10687269	3.796971449	1264.884748	392.1088114	0.010231395
3.124345615	63.10265755	14.79787763	0.010175583	265.0872199	185.2203281
1.243063847	60.10687269	3.796971449	1264.884748	392.1088114	0.010231395
0.174482667	11.13005785	4.0172359	1.209150248	17.94286893	0.004377164
0.304684186	22.32231372	17.26502338	0.00158146	2.163388742	4.980732837
1.780992145	128.7688743	7.582228822	4.900183346	322.1056636	205.9584277
1.780992145	128.7688743	7.582228822	4.900183346	322.1056636	205.9584277
54.42667484	0.005576344	367.0171513	10063105.29	6131.555202	372901.8002
0.211055583	7.898454285	0.291779177	0.074994086	0.001343063	0.000924843
1.243063847	60.10687269	3.796971449	1264.884748	392.1088114	0.010231395
3.124345615	63.10265755	14.79787763	0.010175583	265.0872199	185.2203281
0.601023963	76.60850563	9.383264999	23.63188596	0.045818851	462.6026003
0.317958635	8.326596974	0.503682723	0.592008188	17.63561945	12.35939987
3.124345615	63.10265755	14.79787763	0.010175583	265.0872199	185.2203281
0.601023963	76.60850563	9.383264999	23.63188596	0.045818851	462.6026003
1.348782543	27.52709678	6090.426505	15.99355277	66.96648553	838210.3701
1.707133388	77.06474695	16.04900246	40507.17965	5340475.134	346.9465131
3.124345615	63.10265755	14.79787763	0.010175583	265.0872199	185.2203281
3.124345615	63.10265755	14.79787763	0.010175583	265.0872199	185.2203281
1.243063847	60.10687269	3.796971449	1264.884748	392.1088114	0.010231395

0.601023963	76.60850563	9.383264999	23.63188596	0.045818851	462.6026003
0.471954172	5.311692329	2.789233089	0.281020837	235.7729799	146.2289743
1.243063847	60.10687269	3.796971449	1264.884748	392.1088114	0.010231395
0.304684186	22.32231372	17.26502338	0.00158146	2.163388742	4.980732837
3.124345615	63.10265755	14.79787763	0.010175583	265.0872199	185.2203281
4.188281104	124.9426824	6.682117485	1591.294328	26.60073769	1125.16697
1.334260566	14.20741876	8.479742795	0.434487256	102.6304732	766.7850473
4.188281104	124.9426824	6.682117485	1591.294328	26.60073769	1125.16697
1.74678418	39.14156953	7.732681006	0.018863839	647.2189703	1.13207589
0.170347797	6.407282377	2.216511787	260.8310627	9.115521429	31306.63106
4.114009411	36.50752938	10.49738	1.772448251	1599.451717	416.0128287
8.464536659	135.1578262	41.57906082	0.021150193	1226.703634	1591.805388
3.124345615	63.10265755	14.79787763	0.010175583	265.0872199	185.2203281
1.334260566	14.20741876	8.479742795	0.434487256	102.6304732	766.7850473
1.243063847	60.10687269	3.796971449	1264.884748	392.1088114	0.010231395
4.114009411	36.50752938	10.49738	1.772448251	1599.451717	416.0128287
0.524390548	59.31225537	4.226285114	266.706229	214.1999318	3741.798029
0.104204786	4.424925002	0.000110169	4481.222881	16310.66312	0.018553817
54.42667484	0.005576344	367.0171513	10063105.29	6131.555202	372901.8002
0.601023963	76.60850563	9.383264999	23.63188596	0.045818851	462.6026003
0.304684186	22.32231372	17.26502338	0.00158146	2.163388742	4.980732837
8.464536659	135.1578262	41.57906082	0.021150193	1226.703634	1591.805388
1.348782543	27.52709678	6090.426505	15.99355277	66.96648553	838210.3701
1.780992145	128.7688743	7.582228822	4.900183346	322.1056636	205.9584277
0.21894707	0.05667782	1.060917442	0.621035655	17.24141958	54.31318402
8.464536659	135.1578262	41.57906082	0.021150193	1226.703634	1591.805388
1.243063847	60.10687269	3.796971449	1264.884748	392.1088114	0.010231395
1.243063847	60.10687269	3.796971449	1264.884748	392.1088114	0.010231395
1.334260566	14.20741876	8.479742795	0.434487256	102.6304732	766.7850473
1.243063847	60.10687269	3.796971449	1264.884748	392.1088114	0.010231395
4.114009411	36.50752938	10.49738	1.772448251	1599.451717	416.0128287
1.243063847	60.10687269	3.796971449	1264.884748	392.1088114	0.010231395
1.243063847	60.10687269	3.796971449	1264.884748	392.1088114	0.010231395
8.464536659	135.1578262	41.57906082	0.021150193	1226.703634	1591.805388
8.464536659	135.1578262	41.57906082	0.021150193	1226.703634	1591.805388
3.124345615	63.10265755	14.79787763	0.010175583	265.0872199	185.2203281
0.471954172	5.311692329	2.789233089	0.281020837	235.7729799	146.2289743
8.464536659	135.1578262	41.57906082	0.021150193	1226.703634	1591.805388
0.21894707	0.05667782	1.060917442	0.621035655	17.24141958	54.31318402
1.243063847	60.10687269	3.796971449	1264.884748	392.1088114	0.010231395
3.124345615	63.10265755	14.79787763	0.010175583	265.0872199	185.2203281

4.114009411	36.50752938	10.49738	1.772448251	1599.451717	416.0128287
0.601023963	76.60850563	9.383264999	23.63188596	0.045818851	462.6026003
0.471954172	5.311692329	2.789233089	0.281020837	235.7729799	146.2289743
1.063885018	15.20224748	9.757105764	493.0510379	0.006138098	474.0337749
1.780992145	128.7688743	7.582228822	4.900183346	322.1056636	205.9584277
4.188281104	124.9426824	6.682117485	1591.294328	26.60073769	1125.16697
1.334260566	14.20741876	8.479742795	0.434487256	102.6304732	766.7850473
0.304684186	22.32231372	17.26502338	0.00158146	2.163388742	4.980732837
0.170347797	6.407282377	2.216511787	260.8310627	9.115521429	31306.63106
3.124345615	63.10265755	14.79787763	0.010175583	265.0872199	185.2203281
1.334260566	14.20741876	8.479742795	0.434487256	102.6304732	766.7850473
0.170347797	6.407282377	2.216511787	260.8310627	9.115521429	31306.63106
0.304684186	22.32231372	17.26502338	0.00158146	2.163388742	4.980732837
1.348782543	27.52709678	6090.426505	15.99355277	66.96648553	838210.3701
2.663404946	26.28506589	14.15335703	2.1596973	0.037302351	0.025686657
3.124345615	63.10265755	14.79787763	0.010175583	265.0872199	185.2203281
0.21894707	0.05667782	1.060917442	0.621035655	17.24141958	54.31318402
0.524390548	59.31225537	4.226285114	266.706229	214.1999318	3741.798029
1.780992145	128.7688743	7.582228822	4.900183346	322.1056636	205.9584277
1.243063847	60.10687269	3.796971449	1264.884748	392.1088114	0.010231395
1.334260566	14.20741876	8.479742795	0.434487256	102.6304732	766.7850473
1.243063847	60.10687269	3.796971449	1264.884748	392.1088114	0.010231395
3.124345615	63.10265755	14.79787763	0.010175583	265.0872199	185.2203281
0.21894707	0.05667782	1.060917442	0.621035655	17.24141958	54.31318402
1.243063847	60.10687269	3.796971449	1264.884748	392.1088114	0.010231395
8.464536659	135.1578262	41.57906082	0.021150193	1226.703634	1591.805388
0.170347797	6.407282377	2.216511787	260.8310627	9.115521429	31306.63106
0.170347797	6.407282377	2.216511787	260.8310627	9.115521429	31306.63106
8.480593511	98.16995661	71707.18021	15.73872939	705.8751992	5071.954414
3.124345615	63.10265755	14.79787763	0.010175583	265.0872199	185.2203281
0.21894707	0.05667782	1.060917442	0.621035655	17.24141958	54.31318402
1.243063847	60.10687269	3.796971449	1264.884748	392.1088114	0.010231395
2.488958178	6.23181704	0.013504795	6.06E-05	6.090597043	142.7238958
1.243063847	60.10687269	3.796971449	1264.884748	392.1088114	0.010231395
0.170347797	6.407282377	2.216511787	260.8310627	9.115521429	31306.63106
0.304684186	22.32231372	17.26502338	0.00158146	2.163388742	4.980732837
4.114009411	36.50752938	10.49738	1.772448251	1599.451717	416.0128287
2.663404946	26.28506589	14.15335703	2.1596973	0.037302351	0.025686657
3.124345615	63.10265755	14.79787763	0.010175583	265.0872199	185.2203281
1.243063847	60.10687269	3.796971449	1264.884748	392.1088114	0.010231395
8.464536659	135.1578262	41.57906082	0.021150193	1226.703634	1591.805388

8.464536659	135.1578262	41.57906082	0.021150193	1226.703634	1591.805388
3.124345615	63.10265755	14.79787763	0.010175583	265.0872199	185.2203281
1.243063847	60.10687269	3.796971449	1264.884748	392.1088114	0.010231395
2.488958178	6.23181704	0.013504795	6.06E-05	6.090597043	142.7238958
1.243063847	60.10687269	3.796971449	1264.884748	392.1088114	0.010231395
1.243063847	60.10687269	3.796971449	1264.884748	392.1088114	0.010231395
8.464536659	135.1578262	41.57906082	0.021150193	1226.703634	1591.805388
4.114009411	36.50752938	10.49738	1.772448251	1599.451717	416.0128287
3.124345615	63.10265755	14.79787763	0.010175583	265.0872199	185.2203281
0.304684186	22.32231372	17.26502338	0.00158146	2.163388742	4.980732837
0.471954172	5.311692329	2.789233089	0.281020837	235.7729799	146.2289743
4.45166709	30.81716963	20.27063196	34.7506403	0.03257597	0.035194761
1.243063847	60.10687269	3.796971449	1264.884748	392.1088114	0.010231395
8.464536659	135.1578262	41.57906082	0.021150193	1226.703634	1591.805388
0.471954172	5.311692329	2.789233089	0.281020837	235.7729799	146.2289743
41.8663938	0.415159837	0.000223383	4455.372978	0.054632957	2574.103289
0.170347797	6.407282377	2.216511787	260.8310627	9.115521429	31306.63106
6.974586009	0.015265453	0.169734463	1946.977745	8223.95308	98.71421058
4.114009411	36.50752938	10.49738	1.772448251	1599.451717	416.0128287
0.170347797	6.407282377	2.216511787	260.8310627	9.115521429	31306.63106
0.601023963	76.60850563	9.383264999	23.63188596	0.045818851	462.6026003
0.148796703	6.230127017	1.518385671	7.463994701	27.70295678	90.50473091
8.462505354	660.766597	17.70371273	20.19544547	0.064445396	0.044377546
1.348782543	27.52709678	6090.426505	15.99355277	66.96648553	838210.3701
3.124345615	63.10265755	14.79787763	0.010175583	265.0872199	185.2203281
0.524390548	59.31225537	4.226285114	266.706229	214.1999318	3741.798029
0.601023963	76.60850563	9.383264999	23.63188596	0.045818851	462.6026003
1.780992145	128.7688743	7.582228822	4.900183346	322.1056636	205.9584277
0.104204786	4.424925002	0.000110169	4481.222881	16310.66312	0.018553817
2.488958178	6.23181704	0.013504795	6.06E-05	6.090597043	142.7238958
0.304684186	22.32231372	17.26502338	0.00158146	2.163388742	4.980732837
1.780992145	128.7688743	7.582228822	4.900183346	322.1056636	205.9584277
3.124345615	63.10265755	14.79787763	0.010175583	265.0872199	185.2203281
8.462505354	660.766597	17.70371273	20.19544547	0.064445396	0.044377546
8.480593511	98.16995661	71707.18021	15.73872939	705.8751992	5071.954414
3.124345615	63.10265755	14.79787763	0.010175583	265.0872199	185.2203281
2.663404946	26.28506589	14.15335703	2.1596973	0.037302351	0.025686657
0.170347797	6.407282377	2.216511787	260.8310627	9.115521429	31306.63106
3.124345615	63.10265755	14.79787763	0.010175583	265.0872199	185.2203281
3.124345615	63.10265755	14.79787763	0.010175583	265.0872199	185.2203281
41.8663938	0.415159837	0.000223383	4455.372978	0.054632957	2574.103289

1.243063847	60.10687269	3.796971449	1264.884748	392.1088114	0.010231395
0.320470125	5.012689198	0.937141108	3667.00418	0.001461948	36.10869609
3.124345615	63.10265755	14.79787763	0.010175583	265.0872199	185.2203281
8.464536659	135.1578262	41.57906082	0.021150193	1226.703634	1591.805388
0.304684186	22.32231372	17.26502338	0.00158146	2.163388742	4.980732837
1.334260566	14.20741876	8.479742795	0.434487256	102.6304732	766.7850473
0.170347797	6.407282377	2.216511787	260.8310627	9.115521429	31306.63106
3.124345615	63.10265755	14.79787763	0.010175583	265.0872199	185.2203281
8.464536659	135.1578262	41.57906082	0.021150193	1226.703634	1591.805388
0.21894707	0.05667782	1.060917442	0.621035655	17.24141958	54.31318402
3.124345615	63.10265755	14.79787763	0.010175583	265.0872199	185.2203281
1.243063847	60.10687269	3.796971449	1264.884748	392.1088114	0.010231395
0.317958635	8.326596974	0.503682723	0.592008188	17.63561945	12.35939987
8.464536659	135.1578262	41.57906082	0.021150193	1226.703634	1591.805388
3.124345615	63.10265755	14.79787763	0.010175583	265.0872199	185.2203281
3.124345615	63.10265755	14.79787763	0.010175583	265.0872199	185.2203281
0.21894707	0.05667782	1.060917442	0.621035655	17.24141958	54.31318402
8.464536659	135.1578262	41.57906082	0.021150193	1226.703634	1591.805388
6.974586009	0.015265453	0.169734463	1946.977745	8223.95308	98.71421058
0.148796703	6.230127017	1.518385671	7.463994701	27.70295678	90.50473091
8.462505354	660.766597	17.70371273	20.19544547	0.064445396	0.044377546
3.124345615	63.10265755	14.79787763	0.010175583	265.0872199	185.2203281
3.124345615	63.10265755	14.79787763	0.010175583	265.0872199	185.2203281
4.114009411	36.50752938	10.49738	1.772448251	1599.451717	416.0128287
0.601023963	76.60850563	9.383264999	23.63188596	0.045818851	462.6026003
0.211055583	7.898454285	0.291779177	0.074994086	0.001343063	0.000924843
0.471954172	5.311692329	2.789233089	0.281020837	235.7729799	146.2289743
1.84655553	0.001356082	9.635827175	7.041910215	15.32397228	0.017902965
8.464536659	135.1578262	41.57906082	0.021150193	1226.703634	1591.805388
0.170347797	6.407282377	2.216511787	260.8310627	9.115521429	31306.63106
1.780992145	128.7688743	7.582228822	4.900183346	322.1056636	205.9584277
1.243063847	60.10687269	3.796971449	1264.884748	392.1088114	0.010231395
4.188281104	124.9426824	6.682117485	1591.294328	26.60073769	1125.16697
3.124345615	63.10265755	14.79787763	0.010175583	265.0872199	185.2203281
8.464536659	135.1578262	41.57906082	0.021150193	1226.703634	1591.805388
1.243063847	60.10687269	3.796971449	1264.884748	392.1088114	0.010231395
0.471954172	5.311692329	2.789233089	0.281020837	235.7729799	146.2289743
0.695462637	20.35969391	0.961358453	0.003513087	18.12071288	1.864346973
3.124345615	63.10265755	14.79787763	0.010175583	265.0872199	185.2203281
3.124345615	63.10265755	14.79787763	0.010175583	265.0872199	185.2203281
3.124345615	63.10265755	14.79787763	0.010175583	265.0872199	185.2203281

0.304684186	22.32231372	17.26502338	0.00158146	2.163388742	4.980732837
1.74678418	39.14156953	7.732681006	0.018863839	647.2189703	1.13207589
1.243063847	60.10687269	3.796971449	1264.884748	392.1088114	0.010231395
3.124345615	63.10265755	14.79787763	0.010175583	265.0872199	185.2203281
8.480593511	98.16995661	71707.18021	15.73872939	705.8751992	5071.954414
1.243063847	60.10687269	3.796971449	1264.884748	392.1088114	0.010231395
0.471954172	5.311692329	2.789233089	0.281020837	235.7729799	146.2289743
1.780992145	128.7688743	7.582228822	4.900183346	322.1056636	205.9584277
4.188281104	124.9426824	6.682117485	1591.294328	26.60073769	1125.16697
18.78601156	2572385.756	490.3926503	56.41015292	447.3507743	842535.2186
1.74678418	39.14156953	7.732681006	0.018863839	647.2189703	1.13207589
1.74678418	39.14156953	7.732681006	0.018863839	647.2189703	1.13207589
0.471954172	5.311692329	2.789233089	0.281020837	235.7729799	146.2289743
0.304684186	22.32231372	17.26502338	0.00158146	2.163388742	4.980732837
3.124345615	63.10265755	14.79787763	0.010175583	265.0872199	185.2203281
8.464536659	135.1578262	41.57906082	0.021150193	1226.703634	1591.805388
3.124345615	63.10265755	14.79787763	0.010175583	265.0872199	185.2203281
8.464536659	135.1578262	41.57906082	0.021150193	1226.703634	1591.805388
1.243063847	60.10687269	3.796971449	1264.884748	392.1088114	0.010231395
1.334260566	14.20741876	8.479742795	0.434487256	102.6304732	766.7850473
6.974586009	0.015265453	0.169734463	1946.977745	8223.95308	98.71421058
62.99061471	1609.819268	6.395223922	2.36E-05	1408354.653	0.00559945
8.464536659	135.1578262	41.57906082	0.021150193	1226.703634	1591.805388
8.464536659	135.1578262	41.57906082	0.021150193	1226.703634	1591.805388
1.74678418	39.14156953	7.732681006	0.018863839	647.2189703	1.13207589
0.524390548	59.31225537	4.226285114	266.706229	214.1999318	3741.798029
8.464536659	135.1578262	41.57906082	0.021150193	1226.703634	1591.805388
8.464536659	135.1578262	41.57906082	0.021150193	1226.703634	1591.805388
4.114009411	36.50752938	10.49738	1.772448251	1599.451717	416.0128287
1.707133388	77.06474695	16.04900246	40507.17965	5340475.134	346.9465131
4.114009411	36.50752938	10.49738	1.772448251	1599.451717	416.0128287
4.114009411	36.50752938	10.49738	1.772448251	1599.451717	416.0128287
1.243063847	60.10687269	3.796971449	1264.884748	392.1088114	0.010231395
0.601023963	76.60850563	9.383264999	23.63188596	0.045818851	462.6026003
0.471954172	5.311692329	2.789233089	0.281020837	235.7729799	146.2289743
8.464536659	135.1578262	41.57906082	0.021150193	1226.703634	1591.805388
8.462505354	660.766597	17.70371273	20.19544547	0.064445396	0.044377546
8.480593511	98.16995661	71707.18021	15.73872939	705.8751992	5071.954414
1.780992145	128.7688743	7.582228822	4.900183346	322.1056636	205.9584277
8.464536659	135.1578262	41.57906082	0.021150193	1226.703634	1591.805388
3.124345615	63.10265755	14.79787763	0.010175583	265.0872199	185.2203281

1.74678418	39.14156953	7.732681006	0.018863839	647.2189703	1.13207589
8.464536659	135.1578262	41.57906082	0.021150193	1226.703634	1591.805388
0.601023963	76.60850563	9.383264999	23.63188596	0.045818851	462.6026003
0.695462637	20.35969391	0.961358453	0.003513087	18.12071288	1.864346973
1.243063847	60.10687269	3.796971449	1264.884748	392.1088114	0.010231395
0.304684186	22.32231372	17.26502338	0.00158146	2.163388742	4.980732837
1.74678418	39.14156953	7.732681006	0.018863839	647.2189703	1.13207589
8.480593511	98.16995661	71707.18021	15.73872939	705.8751992	5071.954414
0.524390548	59.31225537	4.226285114	266.706229	214.1999318	3741.798029
0.524390548	59.31225537	4.226285114	266.706229	214.1999318	3741.798029
1.334260566	14.20741876	8.479742795	0.434487256	102.6304732	766.7850473
4.114009411	36.50752938	10.49738	1.772448251	1599.451717	416.0128287
1.243063847	60.10687269	3.796971449	1264.884748	392.1088114	0.010231395
1.707133388	77.06474695	16.04900246	40507.17965	5340475.134	346.9465131
0.471954172	5.311692329	2.789233089	0.281020837	235.7729799	146.2289743
2.663404946	26.28506589	14.15335703	2.1596973	0.037302351	0.025686657
0.471954172	5.311692329	2.789233089	0.281020837	235.7729799	146.2289743
1.780992145	128.7688743	7.582228822	4.900183346	322.1056636	205.9584277
3.124345615	63.10265755	14.79787763	0.010175583	265.0872199	185.2203281
8.480593511	98.16995661	71707.18021	15.73872939	705.8751992	5071.954414
1.348782543	27.52709678	6090.426505	15.99355277	66.96648553	838210.3701
1.243063847	60.10687269	3.796971449	1264.884748	392.1088114	0.010231395
8.480593511	98.16995661	71707.18021	15.73872939	705.8751992	5071.954414
0.148796703	6.230127017	1.518385671	7.463994701	27.70295678	90.50473091
3.124345615	63.10265755	14.79787763	0.010175583	265.0872199	185.2203281
0.304684186	22.32231372	17.26502338	0.00158146	2.163388742	4.980732837
0.21894707	0.05667782	1.060917442	0.621035655	17.24141958	54.31318402
8.464536659	135.1578262	41.57906082	0.021150193	1226.703634	1591.805388
1.780992145	128.7688743	7.582228822	4.900183346	322.1056636	205.9584277
54.42667484	0.005576344	367.0171513	10063105.29	6131.555202	372901.8002
4.114009411	36.50752938	10.49738	1.772448251	1599.451717	416.0128287
0.170347797	6.407282377	2.216511787	260.8310627	9.115521429	31306.63106
2.663404946	26.28506589	14.15335703	2.1596973	0.037302351	0.025686657
1.243063847	60.10687269	3.796971449	1264.884748	392.1088114	0.010231395
4.45166709	30.81716963	20.27063196	34.7506403	0.03257597	0.035194761
1.780992145	128.7688743	7.582228822	4.900183346	322.1056636	205.9584277
8.464536659	135.1578262	41.57906082	0.021150193	1226.703634	1591.805388
3.124345615	63.10265755	14.79787763	0.010175583	265.0872199	185.2203281
1.780992145	128.7688743	7.582228822	4.900183346	322.1056636	205.9584277
3.124345615	63.10265755	14.79787763	0.010175583	265.0872199	185.2203281
1.334260566	14.20741876	8.479742795	0.434487256	102.6304732	766.7850473

6.974586009	0.015265453	0.169734463	1946.977745	8223.95308	98.71421058
0.471954172	5.311692329	2.789233089	0.281020837	235.7729799	146.2289743
1.707133388	77.06474695	16.04900246	40507.17965	5340475.134	346.9465131
1.780992145	128.7688743	7.582228822	4.900183346	322.1056636	205.9584277
0.304684186	22.32231372	17.26502338	0.00158146	2.163388742	4.980732837
1.74678418	39.14156953	7.732681006	0.018863839	647.2189703	1.13207589
3.124345615	63.10265755	14.79787763	0.010175583	265.0872199	185.2203281
1.243063847	60.10687269	3.796971449	1264.884748	392.1088114	0.010231395
1.243063847	60.10687269	3.796971449	1264.884748	392.1088114	0.010231395
0.320470125	5.012689198	0.937141108	3667.00418	0.001461948	36.10869609
0.104204786	4.424925002	0.000110169	4481.222881	16310.66312	0.018553817
1.780992145	128.7688743	7.582228822	4.900183346	322.1056636	205.9584277
3.124345615	63.10265755	14.79787763	0.010175583	265.0872199	185.2203281
8.464536659	135.1578262	41.57906082	0.021150193	1226.703634	1591.805388
1.243063847	60.10687269	3.796971449	1264.884748	392.1088114	0.010231395
0.601023963	76.60850563	9.383264999	23.63188596	0.045818851	462.6026003
0.304684186	22.32231372	17.26502338	0.00158146	2.163388742	4.980732837
1.243063847	60.10687269	3.796971449	1264.884748	392.1088114	0.010231395
0.320470125	5.012689198	0.937141108	3667.00418	0.001461948	36.10869609
0.170347797	6.407282377	2.216511787	260.8310627	9.115521429	31306.63106
1.243063847	60.10687269	3.796971449	1264.884748	392.1088114	0.010231395
0.471954172	5.311692329	2.789233089	0.281020837	235.7729799	146.2289743
1.780992145	128.7688743	7.582228822	4.900183346	322.1056636	205.9584277
1.780992145	128.7688743	7.582228822	4.900183346	322.1056636	205.9584277
4.45166709	30.81716963	20.27063196	34.7506403	0.03257597	0.035194761
1.707133388	77.06474695	16.04900246	40507.17965	5340475.134	346.9465131
1.707133388	77.06474695	16.04900246	40507.17965	5340475.134	346.9465131
1.780992145	128.7688743	7.582228822	4.900183346	322.1056636	205.9584277
1.063885018	15.20224748	9.757105764	493.0510379	0.006138098	474.0337749
1.334260566	14.20741876	8.479742795	0.434487256	102.6304732	766.7850473
3.124345615	63.10265755	14.79787763	0.010175583	265.0872199	185.2203281
8.480593511	98.16995661	71707.18021	15.73872939	705.8751992	5071.954414
0.211055583	7.898454285	0.291779177	0.074994086	0.001343063	0.000924843
2.488958178	6.23181704	0.013504795	6.06E-05	6.090597043	142.7238958
0.320470125	5.012689198	0.937141108	3667.00418	0.001461948	36.10869609
0.211055583	7.898454285	0.291779177	0.074994086	0.001343063	0.000924843
1.243063847	60.10687269	3.796971449	1264.884748	392.1088114	0.010231395
1.84655553	0.001356082	9.635827175	7.041910215	15.32397228	0.017902965
3.124345615	63.10265755	14.79787763	0.010175583	265.0872199	185.2203281
1.84655553	0.001356082	9.635827175	7.041910215	15.32397228	0.017902965
3.124345615	63.10265755	14.79787763	0.010175583	265.0872199	185.2203281

1.243063847	60.10687269	3.796971449	1264.884748	392.1088114	0.010231395
1.243063847	60.10687269	3.796971449	1264.884748	392.1088114	0.010231395
3.124345615	63.10265755	14.79787763	0.010175583	265.0872199	185.2203281
1.243063847	60.10687269	3.796971449	1264.884748	392.1088114	0.010231395
2.663404946	26.28506589	14.15335703	2.1596973	0.037302351	0.025686657
3.124345615	63.10265755	14.79787763	0.010175583	265.0872199	185.2203281
3.124345615	63.10265755	14.79787763	0.010175583	265.0872199	185.2203281
62.99061471	1609.819268	6.395223922	2.36E-05	1408354.653	0.00559945
18.78601156	2572385.756	490.3926503	56.41015292	447.3507743	842535.2186
0.317958635	8.326596974	0.503682723	0.592008188	17.63561945	12.35939987
0.104204786	4.424925002	0.000110169	4481.222881	16310.66312	0.018553817
0.170347797	6.407282377	2.216511787	260.8310627	9.115521429	31306.63106
1.36808232	14.32609198	0.013451001	23048.62822	168.9977706	23.46735703
1.348782543	27.52709678	6090.426505	15.99355277	66.96648553	838210.3701
1.063885018	15.20224748	9.757105764	493.0510379	0.006138098	474.0337749
1.780992145	128.7688743	7.582228822	4.900183346	322.1056636	205.9584277
0.471954172	5.311692329	2.789233089	0.281020837	235.7729799	146.2289743
1.243063847	60.10687269	3.796971449	1264.884748	392.1088114	0.010231395
8.480593511	98.16995661	71707.18021	15.73872939	705.8751992	5071.954414
0.524390548	59.31225537	4.226285114	266.706229	214.1999318	3741.798029
1.243063847	60.10687269	3.796971449	1264.884748	392.1088114	0.010231395
2.663404946	26.28506589	14.15335703	2.1596973	0.037302351	0.025686657
4.114009411	36.50752938	10.49738	1.772448251	1599.451717	416.0128287
1.243063847	60.10687269	3.796971449	1264.884748	392.1088114	0.010231395
3.124345615	63.10265755	14.79787763	0.010175583	265.0872199	185.2203281
0.524390548	59.31225537	4.226285114	266.706229	214.1999318	3741.798029
8.464536659	135.1578262	41.57906082	0.021150193	1226.703634	1591.805388
1.063885018	15.20224748	9.757105764	493.0510379	0.006138098	474.0337749
1.36808232	14.32609198	0.013451001	23048.62822	168.9977706	23.46735703
8.480593511	98.16995661	71707.18021	15.73872939	705.8751992	5071.954414
1.780992145	128.7688743	7.582228822	4.900183346	322.1056636	205.9584277
4.114009411	36.50752938	10.49738	1.772448251	1599.451717	416.0128287
0.304684186	22.32231372	17.26502338	0.00158146	2.163388742	4.980732837
2.663404946	26.28506589	14.15335703	2.1596973	0.037302351	0.025686657
0.471954172	5.311692329	2.789233089	0.281020837	235.7729799	146.2289743
1.243063847	60.10687269	3.796971449	1264.884748	392.1088114	0.010231395
1.780992145	128.7688743	7.582228822	4.900183346	322.1056636	205.9584277
0.170347797	6.407282377	2.216511787	260.8310627	9.115521429	31306.63106
4.114009411	36.50752938	10.49738	1.772448251	1599.451717	416.0128287
8.464536659	135.1578262	41.57906082	0.021150193	1226.703634	1591.805388
1.334260566	14.20741876	8.479742795	0.434487256	102.6304732	766.7850473

0.601023963	76.60850563	9.383264999	23.63188596	0.045818851	462.6026003
3.124345615	63.10265755	14.79787763	0.010175583	265.0872199	185.2203281
3.124345615	63.10265755	14.79787763	0.010175583	265.0872199	185.2203281
1.707133388	77.06474695	16.04900246	40507.17965	5340475.134	346.9465131
8.464536659	135.1578262	41.57906082	0.021150193	1226.703634	1591.805388
4.45166709	30.81716963	20.27063196	34.7506403	0.03257597	0.035194761
1.243063847	60.10687269	3.796971449	1264.884748	392.1088114	0.010231395
1.243063847	60.10687269	3.796971449	1264.884748	392.1088114	0.010231395
8.462505354	660.766597	17.70371273	20.19544547	0.064445396	0.044377546
3.124345615	63.10265755	14.79787763	0.010175583	265.0872199	185.2203281
1.243063847	60.10687269	3.796971449	1264.884748	392.1088114	0.010231395
1.780992145	128.7688743	7.582228822	4.900183346	322.1056636	205.9584277
8.480593511	98.16995661	71707.18021	15.73872939	705.8751992	5071.954414
4.114009411	36.50752938	10.49738	1.772448251	1599.451717	416.0128287
1.243063847	60.10687269	3.796971449	1264.884748	392.1088114	0.010231395
8.464536659	135.1578262	41.57906082	0.021150193	1226.703634	1591.805388
1.243063847	60.10687269	3.796971449	1264.884748	392.1088114	0.010231395
4.114009411	36.50752938	10.49738	1.772448251	1599.451717	416.0128287
1.84655553	0.001356082	9.635827175	7.041910215	15.32397228	0.017902965
1.780992145	128.7688743	7.582228822	4.900183346	322.1056636	205.9584277
8.480593511	98.16995661	71707.18021	15.73872939	705.8751992	5071.954414
62.99061471	1609.819268	6.395223922	2.36E-05	1408354.653	0.00559945
0.471954172	5.311692329	2.789233089	0.281020837	235.7729799	146.2289743
8.464536659	135.1578262	41.57906082	0.021150193	1226.703634	1591.805388
4.114009411	36.50752938	10.49738	1.772448251	1599.451717	416.0128287
0.931440765	45.3976929	7.476177884	126.2810675	0.009949876	263.6330236
0.524390548	59.31225537	4.226285114	266.706229	214.1999318	3741.798029
1.243063847	60.10687269	3.796971449	1264.884748	392.1088114	0.010231395
8.464536659	135.1578262	41.57906082	0.021150193	1226.703634	1591.805388
1.74678418	39.14156953	7.732681006	0.018863839	647.2189703	1.13207589
3.124345615	63.10265755	14.79787763	0.010175583	265.0872199	185.2203281
1.243063847	60.10687269	3.796971449	1264.884748	392.1088114	0.010231395
18.78601156	2572385.756	490.3926503	56.41015292	447.3507743	842535.2186
1.243063847	60.10687269	3.796971449	1264.884748	392.1088114	0.010231395
0.304684186	22.32231372	17.26502338	0.00158146	2.163388742	4.980732837
4.114009411	36.50752938	10.49738	1.772448251	1599.451717	416.0128287
0.317958635	8.326596974	0.503682723	0.592008188	17.63561945	12.35939987
1.334260566	14.20741876	8.479742795	0.434487256	102.6304732	766.7850473
1.707133388	77.06474695	16.04900246	40507.17965	5340475.134	346.9465131
1.334260566	14.20741876	8.479742795	0.434487256	102.6304732	766.7850473
0.21894707	0.05667782	1.060917442	0.621035655	17.24141958	54.31318402

8.464536659	135.1578262	41.57906082	0.021150193	1226.703634	1591.805388
4.114009411	36.50752938	10.49738	1.772448251	1599.451717	416.0128287
1.334260566	14.20741876	8.479742795	0.434487256	102.6304732	766.7850473
1.74678418	39.14156953	7.732681006	0.018863839	647.2189703	1.13207589
8.464536659	135.1578262	41.57906082	0.021150193	1226.703634	1591.805388
1.243063847	60.10687269	3.796971449	1264.884748	392.1088114	0.010231395
1.348782543	27.52709678	6090.426505	15.99355277	66.96648553	838210.3701
0.304684186	22.32231372	17.26502338	0.00158146	2.163388742	4.980732837
1.74678418	39.14156953	7.732681006	0.018863839	647.2189703	1.13207589
3.124345615	63.10265755	14.79787763	0.010175583	265.0872199	185.2203281
0.304684186	22.32231372	17.26502338	0.00158146	2.163388742	4.980732837
0.304684186	22.32231372	17.26502338	0.00158146	2.163388742	4.980732837
1.780992145	128.7688743	7.582228822	4.900183346	322.1056636	205.9584277
2.884854564	621281.7415	38.24704442	4.041383129	87.49995699	650.2497189
1.243063847	60.10687269	3.796971449	1264.884748	392.1088114	0.010231395
8.462505354	660.766597	17.70371273	20.19544547	0.064445396	0.044377546
1.243063847	60.10687269	3.796971449	1264.884748	392.1088114	0.010231395
1.243063847	60.10687269	3.796971449	1264.884748	392.1088114	0.010231395
3.124345615	63.10265755	14.79787763	0.010175583	265.0872199	185.2203281
1.243063847	60.10687269	3.796971449	1264.884748	392.1088114	0.010231395
6.974586009	0.015265453	0.169734463	1946.977745	8223.95308	98.71421058
1.348782543	27.52709678	6090.426505	15.99355277	66.96648553	838210.3701
3.124345615	63.10265755	14.79787763	0.010175583	265.0872199	185.2203281
8.480593511	98.16995661	71707.18021	15.73872939	705.8751992	5071.954414
0.148796703	6.230127017	1.518385671	7.463994701	27.70295678	90.50473091
8.464536659	135.1578262	41.57906082	0.021150193	1226.703634	1591.805388
0.471954172	5.311692329	2.789233089	0.281020837	235.7729799	146.2289743
1.243063847	60.10687269	3.796971449	1264.884748	392.1088114	0.010231395
3.124345615	63.10265755	14.79787763	0.010175583	265.0872199	185.2203281
1.74678418	39.14156953	7.732681006	0.018863839	647.2189703	1.13207589
3.124345615	63.10265755	14.79787763	0.010175583	265.0872199	185.2203281
2.884854564	621281.7415	38.24704442	4.041383129	87.49995699	650.2497189
41.8663938	0.415159837	0.000223383	4455.372978	0.054632957	2574.103289
1.243063847	60.10687269	3.796971449	1264.884748	392.1088114	0.010231395
8.464536659	135.1578262	41.57906082	0.021150193	1226.703634	1591.805388
8.464536659	135.1578262	41.57906082	0.021150193	1226.703634	1591.805388
0.471954172	5.311692329	2.789233089	0.281020837	235.7729799	146.2289743
8.480593511	98.16995661	71707.18021	15.73872939	705.8751992	5071.954414
1.243063847	60.10687269	3.796971449	1264.884748	392.1088114	0.010231395
8.462505354	660.766597	17.70371273	20.19544547	0.064445396	0.044377546
3.124345615	63.10265755	14.79787763	0.010175583	265.0872199	185.2203281

8.464536659	135.1578262	41.57906082	0.021150193	1226.703634	1591.805388
4.114009411	36.50752938	10.49738	1.772448251	1599.451717	416.0128287
1.707133388	77.06474695	16.04900246	40507.17965	5340475.134	346.9465131
0.304684186	22.32231372	17.26502338	0.00158146	2.163388742	4.980732837
8.464536659	135.1578262	41.57906082	0.021150193	1226.703634	1591.805388
0.471954172	5.311692329	2.789233089	0.281020837	235.7729799	146.2289743
8.462505354	660.766597	17.70371273	20.19544547	0.064445396	0.044377546
1.243063847	60.10687269	3.796971449	1264.884748	392.1088114	0.010231395
1.74678418	39.14156953	7.732681006	0.018863839	647.2189703	1.13207589
8.464536659	135.1578262	41.57906082	0.021150193	1226.703634	1591.805388
1.243063847	60.10687269	3.796971449	1264.884748	392.1088114	0.010231395
1.243063847	60.10687269	3.796971449	1264.884748	392.1088114	0.010231395
3.124345615	63.10265755	14.79787763	0.010175583	265.0872199	185.2203281
8.464536659	135.1578262	41.57906082	0.021150193	1226.703634	1591.805388
4.114009411	36.50752938	10.49738	1.772448251	1599.451717	416.0128287
1.243063847	60.10687269	3.796971449	1264.884748	392.1088114	0.010231395
4.114009411	36.50752938	10.49738	1.772448251	1599.451717	416.0128287
1.74678418	39.14156953	7.732681006	0.018863839	647.2189703	1.13207589
2.663404946	26.28506589	14.15335703	2.1596973	0.037302351	0.025686657
1.74678418	39.14156953	7.732681006	0.018863839	647.2189703	1.13207589
8.464536659	135.1578262	41.57906082	0.021150193	1226.703634	1591.805388
1.74678418	39.14156953	7.732681006	0.018863839	647.2189703	1.13207589
3.124345615	63.10265755	14.79787763	0.010175583	265.0872199	185.2203281
41.8663938	0.415159837	0.000223383	4455.372978	0.054632957	2574.103289
1.84655553	0.001356082	9.635827175	7.041910215	15.32397228	0.017902965
1.243063847	60.10687269	3.796971449	1264.884748	392.1088114	0.010231395
1.780992145	128.7688743	7.582228822	4.900183346	322.1056636	205.9584277
4.114009411	36.50752938	10.49738	1.772448251	1599.451717	416.0128287
1.707133388	77.06474695	16.04900246	40507.17965	5340475.134	346.9465131
4.114009411	36.50752938	10.49738	1.772448251	1599.451717	416.0128287
8.464536659	135.1578262	41.57906082	0.021150193	1226.703634	1591.805388
0.304684186	22.32231372	17.26502338	0.00158146	2.163388742	4.980732837
41.8663938	0.415159837	0.000223383	4455.372978	0.054632957	2574.103289
1.707133388	77.06474695	16.04900246	40507.17965	5340475.134	346.9465131
0.471954172	5.311692329	2.789233089	0.281020837	235.7729799	146.2289743
1.780992145	128.7688743	7.582228822	4.900183346	322.1056636	205.9584277
0.471954172	5.311692329	2.789233089	0.281020837	235.7729799	146.2289743
8.464536659	135.1578262	41.57906082	0.021150193	1226.703634	1591.805388
0.21894707	0.05667782	1.060917442	0.621035655	17.24141958	54.31318402
1.243063847	60.10687269	3.796971449	1264.884748	392.1088114	0.010231395
8.464536659	135.1578262	41.57906082	0.021150193	1226.703634	1591.805388

1.780992145	128.7688743	7.582228822	4.900183346	322.1056636	205.9584277
1.243063847	60.10687269	3.796971449	1264.884748	392.1088114	0.010231395
3.124345615	63.10265755	14.79787763	0.010175583	265.0872199	185.2203281
0.148796703	6.230127017	1.518385671	7.463994701	27.70295678	90.50473091
3.124345615	63.10265755	14.79787763	0.010175583	265.0872199	185.2203281
3.124345615	63.10265755	14.79787763	0.010175583	265.0872199	185.2203281
1.243063847	60.10687269	3.796971449	1264.884748	392.1088114	0.010231395
1.780992145	128.7688743	7.582228822	4.900183346	322.1056636	205.9584277
0.471954172	5.311692329	2.789233089	0.281020837	235.7729799	146.2289743
8.464536659	135.1578262	41.57906082	0.021150193	1226.703634	1591.805388
1.243063847	60.10687269	3.796971449	1264.884748	392.1088114	0.010231395
8.464536659	135.1578262	41.57906082	0.021150193	1226.703634	1591.805388
2.884854564	621281.7415	38.24704442	4.041383129	87.49995699	650.2497189
3.124345615	63.10265755	14.79787763	0.010175583	265.0872199	185.2203281
3.124345615	63.10265755	14.79787763	0.010175583	265.0872199	185.2203281
1.707133388	77.06474695	16.04900246	40507.17965	5340475.134	346.9465131
0.601023963	76.60850563	9.383264999	23.63188596	0.045818851	462.6026003
0.471954172	5.311692329	2.789233089	0.281020837	235.7729799	146.2289743
4.114009411	36.50752938	10.49738	1.772448251	1599.451717	416.0128287
0.104204786	4.424925002	0.000110169	4481.222881	16310.66312	0.018553817
8.464536659	135.1578262	41.57906082	0.021150193	1226.703634	1591.805388
0.21894707	0.05667782	1.060917442	0.621035655	17.24141958	54.31318402
0.304684186	22.32231372	17.26502338	0.00158146	2.163388742	4.980732837
0.170347797	6.407282377	2.216511787	260.8310627	9.115521429	31306.63106
1.74678418	39.14156953	7.732681006	0.018863839	647.2189703	1.13207589
0.148796703	6.230127017	1.518385671	7.463994701	27.70295678	90.50473091
0.931440765	45.3976929	7.476177884	126.2810675	0.009949876	263.6330236
0.524390548	59.31225537	4.226285114	266.706229	214.1999318	3741.798029
1.36808232	14.32609198	0.013451001	23048.62822	168.9977706	23.46735703
8.464536659	135.1578262	41.57906082	0.021150193	1226.703634	1591.805388
0.471954172	5.311692329	2.789233089	0.281020837	235.7729799	146.2289743
1.780992145	128.7688743	7.582228822	4.900183346	322.1056636	205.9584277
54.42667484	0.005576344	367.0171513	10063105.29	6131.555202	372901.8002
3.124345615	63.10265755	14.79787763	0.010175583	265.0872199	185.2203281
1.243063847	60.10687269	3.796971449	1264.884748	392.1088114	0.010231395
1.36808232	14.32609198	0.013451001	23048.62822	168.9977706	23.46735703
0.170347797	6.407282377	2.216511787	260.8310627	9.115521429	31306.63106
3.124345615	63.10265755	14.79787763	0.010175583	265.0872199	185.2203281
3.124345615	63.10265755	14.79787763	0.010175583	265.0872199	185.2203281
0.104204786	4.424925002	0.000110169	4481.222881	16310.66312	0.018553817
1.334260566	14.20741876	8.479742795	0.434487256	102.6304732	766.7850473

8.462505354	660.766597	17.70371273	20.19544547	0.064445396	0.044377546
3.124345615	63.10265755	14.79787763	0.010175583	265.0872199	185.2203281
1.063885018	15.20224748	9.757105764	493.0510379	0.006138098	474.0337749
3.124345615	63.10265755	14.79787763	0.010175583	265.0872199	185.2203281
1.243063847	60.10687269	3.796971449	1264.884748	392.1088114	0.010231395
1.334260566	14.20741876	8.479742795	0.434487256	102.6304732	766.7850473
1.780992145	128.7688743	7.582228822	4.900183346	322.1056636	205.9584277
3.124345615	63.10265755	14.79787763	0.010175583	265.0872199	185.2203281
1.334260566	14.20741876	8.479742795	0.434487256	102.6304732	766.7850473
0.304684186	22.32231372	17.26502338	0.00158146	2.163388742	4.980732837
3.124345615	63.10265755	14.79787763	0.010175583	265.0872199	185.2203281
3.124345615	63.10265755	14.79787763	0.010175583	265.0872199	185.2203281
1.84655553	0.001356082	9.635827175	7.041910215	15.32397228	0.017902965
8.464536659	135.1578262	41.57906082	0.021150193	1226.703634	1591.805388
8.462505354	660.766597	17.70371273	20.19544547	0.064445396	0.044377546
8.464536659	135.1578262	41.57906082	0.021150193	1226.703634	1591.805388
3.124345615	63.10265755	14.79787763	0.010175583	265.0872199	185.2203281
1.780992145	128.7688743	7.582228822	4.900183346	322.1056636	205.9584277
0.21894707	0.05667782	1.060917442	0.621035655	17.24141958	54.31318402
1.243063847	60.10687269	3.796971449	1264.884748	392.1088114	0.010231395
0.148796703	6.230127017	1.518385671	7.463994701	27.70295678	90.50473091
1.707133388	77.06474695	16.04900246	40507.17965	5340475.134	346.9465131
8.464536659	135.1578262	41.57906082	0.021150193	1226.703634	1591.805388
1.780992145	128.7688743	7.582228822	4.900183346	322.1056636	205.9584277
3.124345615	63.10265755	14.79787763	0.010175583	265.0872199	185.2203281
3.124345615	63.10265755	14.79787763	0.010175583	265.0872199	185.2203281
1.243063847	60.10687269	3.796971449	1264.884748	392.1088114	0.010231395
1.243063847	60.10687269	3.796971449	1264.884748	392.1088114	0.010231395
1.243063847	60.10687269	3.796971449	1264.884748	392.1088114	0.010231395
1.74678418	39.14156953	7.732681006	0.018863839	647.2189703	1.13207589
1.243063847	60.10687269	3.796971449	1264.884748	392.1088114	0.010231395
1.707133388	77.06474695	16.04900246	40507.17965	5340475.134	346.9465131
3.124345615	63.10265755	14.79787763	0.010175583	265.0872199	185.2203281
3.124345615	63.10265755	14.79787763	0.010175583	265.0872199	185.2203281
0.148796703	6.230127017	1.518385671	7.463994701	27.70295678	90.50473091
0.170347797	6.407282377	2.216511787	260.8310627	9.115521429	31306.63106
0.524390548	59.31225537	4.226285114	266.706229	214.1999318	3741.798029
0.211055583	7.898454285	0.291779177	0.074994086	0.001343063	0.000924843
1.348782543	27.52709678	6090.426505	15.99355277	66.96648553	838210.3701
1.334260566	14.20741876	8.479742795	0.434487256	102.6304732	766.7850473
1.243063847	60.10687269	3.796971449	1264.884748	392.1088114	0.010231395

1.707133388	77.06474695	16.04900246	40507.17965	5340475.134	346.9465131
0.304684186	22.32231372	17.26502338	0.00158146	2.163388742	4.980732837
0.304684186	22.32231372	17.26502338	0.00158146	2.163388742	4.980732837
0.304684186	22.32231372	17.26502338	0.00158146	2.163388742	4.980732837
3.124345615	63.10265755	14.79787763	0.010175583	265.0872199	185.2203281
1.243063847	60.10687269	3.796971449	1264.884748	392.1088114	0.010231395
3.124345615	63.10265755	14.79787763	0.010175583	265.0872199	185.2203281
8.464536659	135.1578262	41.57906082	0.021150193	1226.703634	1591.805388
1.334260566	14.20741876	8.479742795	0.434487256	102.6304732	766.7850473
8.464536659	135.1578262	41.57906082	0.021150193	1226.703634	1591.805388
1.348782543	27.52709678	6090.426505	15.99355277	66.96648553	838210.3701
1.243063847	60.10687269	3.796971449	1264.884748	392.1088114	0.010231395
3.124345615	63.10265755	14.79787763	0.010175583	265.0872199	185.2203281
4.114009411	36.50752938	10.49738	1.772448251	1599.451717	416.0128287
8.464536659	135.1578262	41.57906082	0.021150193	1226.703634	1591.805388
0.320470125	5.012689198	0.937141108	3667.00418	0.001461948	36.10869609
1.243063847	60.10687269	3.796971449	1264.884748	392.1088114	0.010231395
1.243063847	60.10687269	3.796971449	1264.884748	392.1088114	0.010231395
4.114009411	36.50752938	10.49738	1.772448251	1599.451717	416.0128287
0.21894707	0.05667782	1.060917442	0.621035655	17.24141958	54.31318402
1.243063847	60.10687269	3.796971449	1264.884748	392.1088114	0.010231395
54.42667484	0.005576344	367.0171513	10063105.29	6131.555202	372901.8002
8.464536659	135.1578262	41.57906082	0.021150193	1226.703634	1591.805388
0.471954172	5.311692329	2.789233089	0.281020837	235.7729799	146.2289743
4.114009411	36.50752938	10.49738	1.772448251	1599.451717	416.0128287
1.84655553	0.001356082	9.635827175	7.041910215	15.32397228	0.017902965
1.243063847	60.10687269	3.796971449	1264.884748	392.1088114	0.010231395
2.663404946	26.28506589	14.15335703	2.1596973	0.037302351	0.025686657
0.601023963	76.60850563	9.383264999	23.63188596	0.045818851	462.6026003
8.464536659	135.1578262	41.57906082	0.021150193	1226.703634	1591.805388
1.334260566	14.20741876	8.479742795	0.434487256	102.6304732	766.7850473
0.304684186	22.32231372	17.26502338	0.00158146	2.163388742	4.980732837
8.464536659	135.1578262	41.57906082	0.021150193	1226.703634	1591.805388
1.243063847	60.10687269	3.796971449	1264.884748	392.1088114	0.010231395
4.45166709	30.81716963	20.27063196	34.7506403	0.03257597	0.035194761
3.124345615	63.10265755	14.79787763	0.010175583	265.0872199	185.2203281
8.462505354	660.766597	17.70371273	20.19544547	0.064445396	0.044377546
0.317958635	8.326596974	0.503682723	0.592008188	17.63561945	12.35939987
1.243063847	60.10687269	3.796971449	1264.884748	392.1088114	0.010231395
3.124345615	63.10265755	14.79787763	0.010175583	265.0872199	185.2203281
1.707133388	77.06474695	16.04900246	40507.17965	5340475.134	346.9465131

1.243063847	60.10687269	3.796971449	1264.884748	392.1088114	0.010231395
2.488958178	6.23181704	0.013504795	6.06E-05	6.090597043	142.7238958
0.471954172	5.311692329	2.789233089	0.281020837	235.7729799	146.2289743
3.124345615	63.10265755	14.79787763	0.010175583	265.0872199	185.2203281
1.243063847	60.10687269	3.796971449	1264.884748	392.1088114	0.010231395
8.464536659	135.1578262	41.57906082	0.021150193	1226.703634	1591.805388
8.464536659	135.1578262	41.57906082	0.021150193	1226.703634	1591.805388
8.464536659	135.1578262	41.57906082	0.021150193	1226.703634	1591.805388
1.243063847	60.10687269	3.796971449	1264.884748	392.1088114	0.010231395
3.124345615	63.10265755	14.79787763	0.010175583	265.0872199	185.2203281
1.243063847	60.10687269	3.796971449	1264.884748	392.1088114	0.010231395
1.243063847	60.10687269	3.796971449	1264.884748	392.1088114	0.010231395
3.124345615	63.10265755	14.79787763	0.010175583	265.0872199	185.2203281
0.320470125	5.012689198	0.937141108	3667.00418	0.001461948	36.10869609
4.45166709	30.81716963	20.27063196	34.7506403	0.03257597	0.035194761
8.464536659	135.1578262	41.57906082	0.021150193	1226.703634	1591.805388
1.243063847	60.10687269	3.796971449	1264.884748	392.1088114	0.010231395
3.124345615	63.10265755	14.79787763	0.010175583	265.0872199	185.2203281
3.124345615	63.10265755	14.79787763	0.010175583	265.0872199	185.2203281
0.21894707	0.05667782	1.060917442	0.621035655	17.24141958	54.31318402
3.124345615	63.10265755	14.79787763	0.010175583	265.0872199	185.2203281
8.480593511	98.16995661	71707.18021	15.73872939	705.8751992	5071.954414
1.348782543	27.52709678	6090.426505	15.99355277	66.96648553	838210.3701
1.243063847	60.10687269	3.796971449	1264.884748	392.1088114	0.010231395
1.334260566	14.20741876	8.479742795	0.434487256	102.6304732	766.7850473
1.243063847	60.10687269	3.796971449	1264.884748	392.1088114	0.010231395
1.243063847	60.10687269	3.796971449	1264.884748	392.1088114	0.010231395
0.931440765	45.3976929	7.476177884	126.2810675	0.009949876	263.6330236
1.707133388	77.06474695	16.04900246	40507.17965	5340475.134	346.9465131
0.304684186	22.32231372	17.26502338	0.00158146	2.163388742	4.980732837
3.124345615	63.10265755	14.79787763	0.010175583	265.0872199	185.2203281
0.601023963	76.60850563	9.383264999	23.63188596	0.045818851	462.6026003
2.663404946	26.28506589	14.15335703	2.1596973	0.037302351	0.025686657
0.170347797	6.407282377	2.216511787	260.8310627	9.115521429	31306.63106
8.464536659	135.1578262	41.57906082	0.021150193	1226.703634	1591.805388
3.124345615	63.10265755	14.79787763	0.010175583	265.0872199	185.2203281
8.464536659	135.1578262	41.57906082	0.021150193	1226.703634	1591.805388
2.488958178	6.23181704	0.013504795	6.06E-05	6.090597043	142.7238958
8.480593511	98.16995661	71707.18021	15.73872939	705.8751992	5071.954414
1.243063847	60.10687269	3.796971449	1264.884748	392.1088114	0.010231395
0.21894707	0.05667782	1.060917442	0.621035655	17.24141958	54.31318402

2.663404946	26.28506589	14.15335703	2.1596973	0.037302351	0.025686657
1.243063847	60.10687269	3.796971449	1264.884748	392.1088114	0.010231395
8.464536659	135.1578262	41.57906082	0.021150193	1226.703634	1591.805388
1.063885018	15.20224748	9.757105764	493.0510379	0.006138098	474.0337749
3.124345615	63.10265755	14.79787763	0.010175583	265.0872199	185.2203281
8.464536659	135.1578262	41.57906082	0.021150193	1226.703634	1591.805388
8.464536659	135.1578262	41.57906082	0.021150193	1226.703634	1591.805388
18.78601156	2572385.756	490.3926503	56.41015292	447.3507743	842535.2186
0.21894707	0.05667782	1.060917442	0.621035655	17.24141958	54.31318402
0.304684186	22.32231372	17.26502338	0.00158146	2.163388742	4.980732837
3.124345615	63.10265755	14.79787763	0.010175583	265.0872199	185.2203281
1.243063847	60.10687269	3.796971449	1264.884748	392.1088114	0.010231395
3.124345615	63.10265755	14.79787763	0.010175583	265.0872199	185.2203281
1.348782543	27.52709678	6090.426505	15.99355277	66.96648553	838210.3701
0.170347797	6.407282377	2.216511787	260.8310627	9.115521429	31306.63106
8.464536659	135.1578262	41.57906082	0.021150193	1226.703634	1591.805388
0.471954172	5.311692329	2.789233089	0.281020837	235.7729799	146.2289743
1.707133388	77.06474695	16.04900246	40507.17965	5340475.134	346.9465131
0.695462637	20.35969391	0.961358453	0.003513087	18.12071288	1.864346973
1.36808232	14.32609198	0.013451001	23048.62822	168.9977706	23.46735703
0.211055583	7.898454285	0.291779177	0.074994086	0.001343063	0.000924843
8.462505354	660.766597	17.70371273	20.19544547	0.064445396	0.044377546
4.114009411	36.50752938	10.49738	1.772448251	1599.451717	416.0128287
1.84655553	0.001356082	9.635827175	7.041910215	15.32397228	0.017902965
1.243063847	60.10687269	3.796971449	1264.884748	392.1088114	0.010231395
3.124345615	63.10265755	14.79787763	0.010175583	265.0872199	185.2203281
0.170347797	6.407282377	2.216511787	260.8310627	9.115521429	31306.63106
1.334260566	14.20741876	8.479742795	0.434487256	102.6304732	766.7850473
0.471954172	5.311692329	2.789233089	0.281020837	235.7729799	146.2289743
8.464536659	135.1578262	41.57906082	0.021150193	1226.703634	1591.805388
8.464536659	135.1578262	41.57906082	0.021150193	1226.703634	1591.805388
1.243063847	60.10687269	3.796971449	1264.884748	392.1088114	0.010231395
3.124345615	63.10265755	14.79787763	0.010175583	265.0872199	185.2203281
1.707133388	77.06474695	16.04900246	40507.17965	5340475.134	346.9465131
1.063885018	15.20224748	9.757105764	493.0510379	0.006138098	474.0337749
0.174482667	11.13005785	4.0172359	1.209150248	17.94286893	0.004377164
1.243063847	60.10687269	3.796971449	1264.884748	392.1088114	0.010231395
3.124345615	63.10265755	14.79787763	0.010175583	265.0872199	185.2203281
1.74678418	39.14156953	7.732681006	0.018863839	647.2189703	1.13207589
3.124345615	63.10265755	14.79787763	0.010175583	265.0872199	185.2203281
1.780992145	128.7688743	7.582228822	4.900183346	322.1056636	205.9584277

4.114009411	36.50752938	10.49738	1.772448251	1599.451717	416.0128287
8.464536659	135.1578262	41.57906082	0.021150193	1226.703634	1591.805388
1.707133388	77.06474695	16.04900246	40507.17965	5340475.134	346.9465131
0.471954172	5.311692329	2.789233089	0.281020837	235.7729799	146.2289743
1.348782543	27.52709678	6090.426505	15.99355277	66.96648553	838210.3701
1.243063847	60.10687269	3.796971449	1264.884748	392.1088114	0.010231395
0.304684186	22.32231372	17.26502338	0.00158146	2.163388742	4.980732837
0.211055583	7.898454285	0.291779177	0.074994086	0.001343063	0.000924843
0.170347797	6.407282377	2.216511787	260.8310627	9.115521429	31306.63106
1.780992145	128.7688743	7.582228822	4.900183346	322.1056636	205.9584277
0.471954172	5.311692329	2.789233089	0.281020837	235.7729799	146.2289743
0.931440765	45.3976929	7.476177884	126.2810675	0.009949876	263.6330236
0.304684186	22.32231372	17.26502338	0.00158146	2.163388742	4.980732837
4.45166709	30.81716963	20.27063196	34.7506403	0.03257597	0.035194761
3.124345615	63.10265755	14.79787763	0.010175583	265.0872199	185.2203281
0.304684186	22.32231372	17.26502338	0.00158146	2.163388742	4.980732837
3.124345615	63.10265755	14.79787763	0.010175583	265.0872199	185.2203281
0.471954172	5.311692329	2.789233089	0.281020837	235.7729799	146.2289743
8.464536659	135.1578262	41.57906082	0.021150193	1226.703634	1591.805388
0.471954172	5.311692329	2.789233089	0.281020837	235.7729799	146.2289743
1.243063847	60.10687269	3.796971449	1264.884748	392.1088114	0.010231395
8.464536659	135.1578262	41.57906082	0.021150193	1226.703634	1591.805388
1.243063847	60.10687269	3.796971449	1264.884748	392.1088114	0.010231395
0.601023963	76.60850563	9.383264999	23.63188596	0.045818851	462.6026003
0.471954172	5.311692329	2.789233089	0.281020837	235.7729799	146.2289743
2.663404946	26.28506589	14.15335703	2.1596973	0.037302351	0.025686657
8.462505354	660.766597	17.70371273	20.19544547	0.064445396	0.044377546
8.464536659	135.1578262	41.57906082	0.021150193	1226.703634	1591.805388
4.114009411	36.50752938	10.49738	1.772448251	1599.451717	416.0128287
8.464536659	135.1578262	41.57906082	0.021150193	1226.703634	1591.805388
0.601023963	76.60850563	9.383264999	23.63188596	0.045818851	462.6026003
0.471954172	5.311692329	2.789233089	0.281020837	235.7729799	146.2289743
0.104204786	4.424925002	0.000110169	4481.222881	16310.66312	0.018553817
0.170347797	6.407282377	2.216511787	260.8310627	9.115521429	31306.63106
0.931440765	45.3976929	7.476177884	126.2810675	0.009949876	263.6330236
0.170347797	6.407282377	2.216511787	260.8310627	9.115521429	31306.63106
1.243063847	60.10687269	3.796971449	1264.884748	392.1088114	0.010231395
0.174482667	11.13005785	4.0172359	1.209150248	17.94286893	0.004377164
0.471954172	5.311692329	2.789233089	0.281020837	235.7729799	146.2289743
1.334260566	14.20741876	8.479742795	0.434487256	102.6304732	766.7850473
8.480593511	98.16995661	71707.18021	15.73872939	705.8751992	5071.954414

0.174482667	11.13005785	4.0172359	1.209150248	17.94286893	0.004377164
1.84655553	0.001356082	9.635827175	7.041910215	15.32397228	0.017902965
3.124345615	63.10265755	14.79787763	0.010175583	265.0872199	185.2203281
3.124345615	63.10265755	14.79787763	0.010175583	265.0872199	185.2203281
1.243063847	60.10687269	3.796971449	1264.884748	392.1088114	0.010231395
0.104204786	4.424925002	0.000110169	4481.222881	16310.66312	0.018553817
1.74678418	39.14156953	7.732681006	0.018863839	647.2189703	1.13207589
1.427332548	126.8583124	6.880385283	0.00271409	0.021507298	2950.544493
4.114009411	36.50752938	10.49738	1.772448251	1599.451717	416.0128287
0.104204786	4.424925002	0.000110169	4481.222881	16310.66312	0.018553817
1.429694041	0.001440843	11.47926338	6.058372034	35.35957098	284.4223853
3.124345615	63.10265755	14.79787763	0.010175583	265.0872199	185.2203281
1.707133388	77.06474695	16.04900246	40507.17965	5340475.134	346.9465131
0.317958635	8.326596974	0.503682723	0.592008188	17.63561945	12.35939987
2.884854564	621281.7415	38.24704442	4.041383129	87.49995699	650.2497189
0.104204786	4.424925002	0.000110169	4481.222881	16310.66312	0.018553817
8.480593511	98.16995661	71707.18021	15.73872939	705.8751992	5071.954414
0.471954172	5.311692329	2.789233089	0.281020837	235.7729799	146.2289743
3.124345615	63.10265755	14.79787763	0.010175583	265.0872199	185.2203281
41.8663938	0.415159837	0.000223383	4455.372978	0.054632957	2574.103289
8.464536659	135.1578262	41.57906082	0.021150193	1226.703634	1591.805388
1.334260566	14.20741876	8.479742795	0.434487256	102.6304732	766.7850473
1.243063847	60.10687269	3.796971449	1264.884748	392.1088114	0.010231395
0.524390548	59.31225537	4.226285114	266.706229	214.1999318	3741.798029
0.304684186	22.32231372	17.26502338	0.00158146	2.163388742	4.980732837
1.780992145	128.7688743	7.582228822	4.900183346	322.1056636	205.9584277
54.42667484	0.005576344	367.0171513	10063105.29	6131.555202	372901.8002
8.464536659	135.1578262	41.57906082	0.021150193	1226.703634	1591.805388
1.334260566	14.20741876	8.479742795	0.434487256	102.6304732	766.7850473
1.780992145	128.7688743	7.582228822	4.900183346	322.1056636	205.9584277
0.471954172	5.311692329	2.789233089	0.281020837	235.7729799	146.2289743
0.104204786	4.424925002	0.000110169	4481.222881	16310.66312	0.018553817
1.334260566	14.20741876	8.479742795	0.434487256	102.6304732	766.7850473
0.174482667	11.13005785	4.0172359	1.209150248	17.94286893	0.004377164
1.427332548	126.8583124	6.880385283	0.00271409	0.021507298	2950.544493
0.104204786	4.424925002	0.000110169	4481.222881	16310.66312	0.018553817
3.124345615	63.10265755	14.79787763	0.010175583	265.0872199	185.2203281
2.663404946	26.28506589	14.15335703	2.1596973	0.037302351	0.025686657
0.104204786	4.424925002	0.000110169	4481.222881	16310.66312	0.018553817
0.304684186	22.32231372	17.26502338	0.00158146	2.163388742	4.980732837
3.124345615	63.10265755	14.79787763	0.010175583	265.0872199	185.2203281

8.464536659	135.1578262	41.57906082	0.021150193	1226.703634	1591.805388
41.8663938	0.415159837	0.000223383	4455.372978	0.054632957	2574.103289
1.780992145	128.7688743	7.582228822	4.900183346	322.1056636	205.9584277
4.45166709	30.81716963	20.27063196	34.7506403	0.03257597	0.035194761
1.348782543	27.52709678	6090.426505	15.99355277	66.96648553	838210.3701
0.524390548	59.31225537	4.226285114	266.706229	214.1999318	3741.798029
0.21894707	0.05667782	1.060917442	0.621035655	17.24141958	54.31318402
0.304684186	22.32231372	17.26502338	0.00158146	2.163388742	4.980732837
0.304684186	22.32231372	17.26502338	0.00158146	2.163388742	4.980732837
1.243063847	60.10687269	3.796971449	1264.884748	392.1088114	0.010231395
1.334260566	14.20741876	8.479742795	0.434487256	102.6304732	766.7850473
1.780992145	128.7688743	7.582228822	4.900183346	322.1056636	205.9584277
8.480593511	98.16995661	71707.18021	15.73872939	705.8751992	5071.954414
8.464536659	135.1578262	41.57906082	0.021150193	1226.703634	1591.805388
1.348782543	27.52709678	6090.426505	15.99355277	66.96648553	838210.3701
1.243063847	60.10687269	3.796971449	1264.884748	392.1088114	0.010231395
8.464536659	135.1578262	41.57906082	0.021150193	1226.703634	1591.805388
4.188281104	124.9426824	6.682117485	1591.294328	26.60073769	1125.16697
18.78601156	2572385.756	490.3926503	56.41015292	447.3507743	842535.2186
1.348782543	27.52709678	6090.426505	15.99355277	66.96648553	838210.3701
1.36808232	14.32609198	0.013451001	23048.62822	168.9977706	23.46735703
8.480593511	98.16995661	71707.18021	15.73872939	705.8751992	5071.954414
0.21894707	0.05667782	1.060917442	0.621035655	17.24141958	54.31318402
4.114009411	36.50752938	10.49738	1.772448251	1599.451717	416.0128287
2.884854564	621281.7415	38.24704442	4.041383129	87.49995699	650.2497189
0.170347797	6.407282377	2.216511787	260.8310627	9.115521429	31306.63106
1.780992145	128.7688743	7.582228822	4.900183346	322.1056636	205.9584277
1.707133388	77.06474695	16.04900246	40507.17965	5340475.134	346.9465131
3.124345615	63.10265755	14.79787763	0.010175583	265.0872199	185.2203281
1.063885018	15.20224748	9.757105764	493.0510379	0.006138098	474.0337749
0.320470125	5.012689198	0.937141108	3667.00418	0.001461948	36.10869609
8.464536659	135.1578262	41.57906082	0.021150193	1226.703634	1591.805388
8.480593511	98.16995661	71707.18021	15.73872939	705.8751992	5071.954414
1.780992145	128.7688743	7.582228822	4.900183346	322.1056636	205.9584277
1.780992145	128.7688743	7.582228822	4.900183346	322.1056636	205.9584277
1.36808232	14.32609198	0.013451001	23048.62822	168.9977706	23.46735703
1.334260566	14.20741876	8.479742795	0.434487256	102.6304732	766.7850473
0.174482667	11.13005785	4.0172359	1.209150248	17.94286893	0.004377164
4.45166709	30.81716963	20.27063196	34.7506403	0.03257597	0.035194761
0.304684186	22.32231372	17.26502338	0.00158146	2.163388742	4.980732837
0.317958635	8.326596974	0.503682723	0.592008188	17.63561945	12.35939987

1.429694041	0.001440843	11.47926338	6.058372034	35.35957098	284.4223853
8.464536659	135.1578262	41.57906082	0.021150193	1226.703634	1591.805388
1.707133388	77.06474695	16.04900246	40507.17965	5340475.134	346.9465131
0.304684186	22.32231372	17.26502338	0.00158146	2.163388742	4.980732837
8.464536659	135.1578262	41.57906082	0.021150193	1226.703634	1591.805388
4.114009411	36.50752938	10.49738	1.772448251	1599.451717	416.0128287
0.104204786	4.424925002	0.000110169	4481.222881	16310.66312	0.018553817
1.334260566	14.20741876	8.479742795	0.434487256	102.6304732	766.7850473
8.480593511	98.16995661	71707.18021	15.73872939	705.8751992	5071.954414
1.243063847	60.10687269	3.796971449	1264.884748	392.1088114	0.010231395
1.334260566	14.20741876	8.479742795	0.434487256	102.6304732	766.7850473
3.124345615	63.10265755	14.79787763	0.010175583	265.0872199	185.2203281
0.317958635	8.326596974	0.503682723	0.592008188	17.63561945	12.35939987
0.304684186	22.32231372	17.26502338	0.00158146	2.163388742	4.980732837
0.524390548	59.31225537	4.226285114	266.706229	214.1999318	3741.798029
1.243063847	60.10687269	3.796971449	1264.884748	392.1088114	0.010231395
0.524390548	59.31225537	4.226285114	266.706229	214.1999318	3741.798029
1.243063847	60.10687269	3.796971449	1264.884748	392.1088114	0.010231395
27	28	29	30	31	32
TMPRSS2-ERG fusion gene	ATP5O-Hs04272738_m1	DSCAM-Hs00242097_m1	ERG-Hs01573964_m1	ICOSLG-Hs00391287_m1	DOP1B-Hs01123288_m1

33	34	35	36	37	38
DOP1B-Hs01123267_g1	C21orf33-Hs01105802_g1	ADAMTS5-Hs04272736_s1	CXADR-Hs04194411_s1	NCAM2-Hs01562292_m1	UBASH3A-Hs00955169_m1
0.913205517	0.465492648	0.121563839	0.224938658	0.295308505	152.482659
1.068634838	0.2604341	0.196945467	0.37714008	46.23566445	8837.157871
0.004024004	368318.5076	3.272305712	2.038237132	15706537.8	20725.87129
240.9357673	2.507445522	3.079828864	6.027526237	1.874037513	0.085540442
6.576777556	0.154979855	0.185938425	0.306368208	0.392868679	17120696.04
0.115838719	1.390917353	0.676136839	0.862098433	125.6434586	1.102056855
5.662102137	7602.241658	1.332423356	2.289245835	8.725877059	12.79693969
13.85852294	2.94365403	0.066007009	0.230065413	0.000359971	112.553689
440884.0624	0.154518703	0.258265278	0.420031974	1078.193289	475397.8458
16.43083435	1.038758451	0.582370226	0.964636268	1.532059487	3.036645003
0.000438256	2796.835423	8.702719388	2.035262887	18.45103414	63.25200782
240.9357673	2.507445522	3.079828864	6.027526237	1.874037513	0.085540442
3.6071682	2.407602882	3.102010539	1.756748893	4.943116878	2198476.462
440884.0624	0.154518703	0.258265278	0.420031974	1078.193289	475397.8458
21.37426037	6.917768134	0.969799877	1.243043994	365018.997	38.10202225
4.865064799	0.291467689	0.873699995	1.405282914	10.19556501	76.53716017
39.42577124	9.034083895	2.93569175	3.009520281	0.213184255	4057.246754
11.41703718	101.8718996	0.230586939	0.511309832	1671.23098	0.003620568
0.004024004	368318.5076	3.272305712	2.038237132	15706537.8	20725.87129
634.291053	3.007188226	1.314365694	2.148444979	66.11309657	1.883185328
4.865064799	0.291467689	0.873699995	1.405282914	10.19556501	76.53716017
46124.02268	0.096217435	1.478132526	2.779263028	4684.831127	47.43587019
1399846.866	120.5310523	1.341649005	7.573108872	1197.111242	877.0790061
0.000438256	2796.835423	8.702719388	2.035262887	18.45103414	63.25200782
6.576777556	0.154979855	0.185938425	0.306368208	0.392868679	17120696.04
1399846.866	120.5310523	1.341649005	7.573108872	1197.111242	877.0790061
16.43083435	1.038758451	0.582370226	0.964636268	1.532059487	3.036645003
0.000438256	2796.835423	8.702719388	2.035262887	18.45103414	63.25200782
21.37426037	6.917768134	0.969799877	1.243043994	365018.997	38.10202225
440884.0624	0.154518703	0.258265278	0.420031974	1078.193289	475397.8458
0.913205517	0.465492648	0.121563839	0.224938658	0.295308505	152.482659
5155.620287	1.249132475	1.205962445	0.378968812	0.007514676	329.5588706
4.865064799	0.291467689	0.873699995	1.405282914	10.19556501	76.53716017
0.115838719	1.390917353	0.676136839	0.862098433	125.6434586	1.102056855
11.25728785	2.819336574	0.092880011	0.251546398	0.031910502	75.73637919
16.43083435	1.038758451	0.582370226	0.964636268	1.532059487	3.036645003
46124.02268	0.096217435	1.478132526	2.779263028	4684.831127	47.43587019

1399846.866	120.5310523	1.341649005	7.573108872	1197.111242	877.0790061
0.115838719	1.390917353	0.676136839	0.862098433	125.6434586	1.102056855
4.18715841	13940.81181	1.175248871	0.566568938	2020.633806	1287.095513
4.865064799	0.291467689	0.873699995	1.405282914	10.19556501	76.53716017
16.43083435	1.038758451	0.582370226	0.964636268	1.532059487	3.036645003
0.115838719	1.390917353	0.676136839	0.862098433	125.6434586	1.102056855
5155.620287	1.249132475	1.205962445	0.378968812	0.007514676	329.5588706
634.291053	3.007188226	1.314365694	2.148444979	66.11309657	1.883185328
6.576777556	0.154979855	0.185938425	0.306368208	0.392868679	17120696.04
4.18715841	13940.81181	1.175248871	0.566568938	2020.633806	1287.095513
6.576777556	0.154979855	0.185938425	0.306368208	0.392868679	17120696.04
0.004024004	368318.5076	3.272305712	2.038237132	15706537.8	20725.87129
46124.02268	0.096217435	1.478132526	2.779263028	4684.831127	47.43587019
2.468255987	0.026418519	0.443674403	0.433507053	2.47E-05	66.9519551
18568.80575	216.7719624	7.314889929	2.984262284	5.303856851	0.112210498
54.3281515	9.505708636	0.981578193	2.424928695	0.525099404	658.4281885
19.76773277	26.15553412	2.164307785	1.613844364	2.124361233	40.20781649
5.879186695	8.83174086	1.265719456	1.526789857	4.590098063	4589304.132
1266.016198	136.166018	4.830015036	5.658542823	0.012961684	8.424915164
21.73776805	153.007792	0.633428826	1.626510537	0.006475321	0.213273606
19.76773277	26.15553412	2.164307785	1.613844364	2.124361233	40.20781649
22.09800435	45129.47088	0.731099825	0.062816755	0.275597132	3.353155271
30.41401997	5.192032106	8.204517682	1.916424455	14.06887752	3668.158746
54.3281515	9.505708636	0.981578193	2.424928695	0.525099404	658.4281885
66.45741888	0.48027586	0.547940367	1.093570903	25.01397453	0.0155948
1266.016198	136.166018	4.830015036	5.658542823	0.012961684	8.424915164
266.2567944	23.73902711	0.246481843	2.57468402	103.108229	0.161420088
66.45741888	0.48027586	0.547940367	1.093570903	25.01397453	0.0155948
19.76773277	26.15553412	2.164307785	1.613844364	2.124361233	40.20781649
1266.016198	136.166018	4.830015036	5.658542823	0.012961684	8.424915164
0.000235526	0.42445576	0.130664491	1.295339448	66.07924721	0.404880467
15.34601505	31813.10151	1.092970564	0.391240411	12.10629879	0.050273659
22.09800435	45129.47088	0.731099825	0.062816755	0.275597132	3.353155271
66.45741888	0.48027586	0.547940367	1.093570903	25.01397453	0.0155948
36.97841801	2.472742827	3.49391889	0.172082191	32.65910515	2.600578752
19.76773277	26.15553412	2.164307785	1.613844364	2.124361233	40.20781649
21.73776805	153.007792	0.633428826	1.626510537	0.006475321	0.213273606
66.45741888	0.48027586	0.547940367	1.093570903	25.01397453	0.0155948
19.76773277	26.15553412	2.164307785	1.613844364	2.124361233	40.20781649
66.45741888	0.48027586	0.547940367	1.093570903	25.01397453	0.0155948
10.07599483	0.765699433	0.315126474	2.826034881	14.14867545	6931.52463

8.313131537	5.385900628	0.935531588	1.380779273	662.0914902	639.2113166
18568.80575	216.7719624	7.314889929	2.984262284	5.303856851	0.112210498
54.3281515	9.505708636	0.981578193	2.424928695	0.525099404	658.4281885
5.879186695	8.83174086	1.265719456	1.526789857	4.590098063	4589304.132
73.26665109	3.671444805	9.867576127	2.459204558	0.654044029	3585950013
22.09800435	45129.47088	0.731099825	0.062816755	0.275597132	3.353155271
10.07599483	0.765699433	0.315126474	2.826034881	14.14867545	6931.52463
169.1928822	3.810743241	1.473957122	2.043511683	87877.79345	1.43004752
22.09800435	45129.47088	0.731099825	0.062816755	0.275597132	3.353155271
194674.4558	0.798978841	0.496572546	1.627149415	83.23698734	20.14299098
36.97841801	2.472742827	3.49391889	0.172082191	32.65910515	2.600578752
107126.8596	0.123368538	1.227067142	0.910983175	0.752820883	0.028279923
1266.016198	136.166018	4.830015036	5.658542823	0.012961684	8.424915164
19.76773277	26.15553412	2.164307785	1.613844364	2.124361233	40.20781649
1266.016198	136.166018	4.830015036	5.658542823	0.012961684	8.424915164
66.45741888	0.48027586	0.547940367	1.093570903	25.01397453	0.0155948
66.45741888	0.48027586	0.547940367	1.093570903	25.01397453	0.0155948
66.45741888	0.48027586	0.547940367	1.093570903	25.01397453	0.0155948
66.45741888	0.48027586	0.547940367	1.093570903	25.01397453	0.0155948
66.45741888	0.48027586	0.547940367	1.093570903	25.01397453	0.0155948
22.54147642	2.490918001	0.428640274	0.742596221	0.004262236	19.27915906
22.09800435	45129.47088	0.731099825	0.062816755	0.275597132	3.353155271
19.76773277	26.15553412	2.164307785	1.613844364	2.124361233	40.20781649
54.3281515	9.505708636	0.981578193	2.424928695	0.525099404	658.4281885
19.76773277	26.15553412	2.164307785	1.613844364	2.124361233	40.20781649
1266.016198	136.166018	4.830015036	5.658542823	0.012961684	8.424915164
22.09800435	45129.47088	0.731099825	0.062816755	0.275597132	3.353155271
0.000963319	0.285624349	0.585966806	0.5481098	4035.3477	0.210294443
66.45741888	0.48027586	0.547940367	1.093570903	25.01397453	0.0155948
66.45741888	0.48027586	0.547940367	1.093570903	25.01397453	0.0155948
23.15224205	12.48785503	7.317679096	11.69104729	1.828916419	490.1890688
53.0253013	0.000448569	1.02977887	2.396825512	135.7678598	0.038128973
0.000963319	0.285624349	0.585966806	0.5481098	4035.3477	0.210294443
1266.016198	136.166018	4.830015036	5.658542823	0.012961684	8.424915164
0.927904551	13.09900442	3.821802563	1.653896965	9827.705967	619348.9686
30.41401997	5.192032106	8.204517682	1.916424455	14.06887752	3668.158746
2.468255987	0.026418519	0.443674403	0.433507053	2.47E-05	66.9519551
169.1928822	3.810743241	1.473957122	2.043511683	87877.79345	1.43004752
5.879186695	8.83174086	1.265719456	1.526789857	4.590098063	4589304.132
1266.016198	136.166018	4.830015036	5.658542823	0.012961684	8.424915164
169.1928822	3.810743241	1.473957122	2.043511683	87877.79345	1.43004752

66.45741888	0.48027586	0.547940367	1.093570903	25.01397453	0.0155948
1.031468273	3.124178523	1.548182107	2.203696827	3.900025721	148.5739097
1266.016198	136.166018	4.830015036	5.658542823	0.012961684	8.424915164
30.41401997	5.192032106	8.204517682	1.916424455	14.06887752	3668.158746
19.76773277	26.15553412	2.164307785	1.613844364	2.124361233	40.20781649
1266.016198	136.166018	4.830015036	5.658542823	0.012961684	8.424915164
54.3281515	9.505708636	0.981578193	2.424928695	0.525099404	658.4281885
1.031468273	3.124178523	1.548182107	2.203696827	3.900025721	148.5739097
1266.016198	136.166018	4.830015036	5.658542823	0.012961684	8.424915164
19.76773277	26.15553412	2.164307785	1.613844364	2.124361233	40.20781649
66.45741888	0.48027586	0.547940367	1.093570903	25.01397453	0.0155948
21.73776805	153.007792	0.633428826	1.626510537	0.006475321	0.213273606
1266.016198	136.166018	4.830015036	5.658542823	0.012961684	8.424915164
19.76773277	26.15553412	2.164307785	1.613844364	2.124361233	40.20781649
1266.016198	136.166018	4.830015036	5.658542823	0.012961684	8.424915164
21.73776805	153.007792	0.633428826	1.626510537	0.006475321	0.213273606
1266.016198	136.166018	4.830015036	5.658542823	0.012961684	8.424915164
3.528750715	100.4526071	0.712168835	1.005018489	0.762780078	25.83373763
0.000963319	0.285624349	0.585966806	0.5481098	4035.3477	0.210294443
36.97841801	2.472742827	3.49391889	0.172082191	32.65910515	2.600578752
313461.5456	2.04668892	0.072055881	0.232985497	1.295160328	265252.0966
22.54147642	2.490918001	0.428640274	0.742596221	0.004262236	19.27915906
19.76773277	26.15553412	2.164307785	1.613844364	2.124361233	40.20781649
15.34601505	31813.10151	1.092970564	0.391240411	12.10629879	0.050273659
5.879186695	8.83174086	1.265719456	1.526789857	4.590098063	4589304.132
53.0253013	0.000448569	1.02977887	2.396825512	135.7678598	0.038128973
1.031468273	3.124178523	1.548182107	2.203696827	3.900025721	148.5739097
21.73776805	153.007792	0.633428826	1.626510537	0.006475321	0.213273606
0.633821239	0.00030662	1.741000906	1.268339685	0.075508671	0.077116824
313461.5456	2.04668892	0.072055881	0.232985497	1.295160328	265252.0966
19.76773277	26.15553412	2.164307785	1.613844364	2.124361233	40.20781649
10.07599483	0.765699433	0.315126474	2.826034881	14.14867545	6931.52463
1.031468273	3.124178523	1.548182107	2.203696827	3.900025721	148.5739097
19.76773277	26.15553412	2.164307785	1.613844364	2.124361233	40.20781649
5.879186695	8.83174086	1.265719456	1.526789857	4.590098063	4589304.132
66.45741888	0.48027586	0.547940367	1.093570903	25.01397453	0.0155948
1266.016198	136.166018	4.830015036	5.658542823	0.012961684	8.424915164
18568.80575	216.7719624	7.314889929	2.984262284	5.303856851	0.112210498
19.76773277	26.15553412	2.164307785	1.613844364	2.124361233	40.20781649
19.76773277	26.15553412	2.164307785	1.613844364	2.124361233	40.20781649
21.73776805	153.007792	0.633428826	1.626510537	0.006475321	0.213273606

169.1928822	3.810743241	1.473957122	2.043511683	87877.79345	1.43004752
22.54147642	2.490918001	0.428640274	0.742596221	0.004262236	19.27915906
36.77273411	14.45513701	0.62100212	0.852578792	1760.282485	0.796748454
1.183699882	1627.295113	2.288510658	2.116976239	0.050920618	0.034191149
157.421691	5.917306608	3.674731809	4.837610608	0.253877466	83.0582072
36.97841801	2.472742827	3.49391889	0.172082191	32.65910515	2.600578752
1.031468273	3.124178523	1.548182107	2.203696827	3.900025721	148.5739097
22.09800435	45129.47088	0.731099825	0.062816755	0.275597132	3.353155271
30.41401997	5.192032106	8.204517682	1.916424455	14.06887752	3668.158746
0.000963319	0.285624349	0.585966806	0.5481098	4035.3477	0.210294443
313461.5456	2.04668892	0.072055881	0.232985497	1.295160328	265252.0966
10.07599483	0.765699433	0.315126474	2.826034881	14.14867545	6931.52463
66.45741888	0.48027586	0.547940367	1.093570903	25.01397453	0.0155948
66.45741888	0.48027586	0.547940367	1.093570903	25.01397453	0.0155948
22.09800435	45129.47088	0.731099825	0.062816755	0.275597132	3.353155271
66.45741888	0.48027586	0.547940367	1.093570903	25.01397453	0.0155948
66.45741888	0.48027586	0.547940367	1.093570903	25.01397453	0.0155948
66.45741888	0.48027586	0.547940367	1.093570903	25.01397453	0.0155948
66.45741888	0.48027586	0.547940367	1.093570903	25.01397453	0.0155948
22.09800435	45129.47088	0.731099825	0.062816755	0.275597132	3.353155271
1266.016198	136.166018	4.830015036	5.658542823	0.012961684	8.424915164
66.45741888	0.48027586	0.547940367	1.093570903	25.01397453	0.0155948
19.76773277	26.15553412	2.164307785	1.613844364	2.124361233	40.20781649
66.45741888	0.48027586	0.547940367	1.093570903	25.01397453	0.0155948
0.000235526	0.42445576	0.130664491	1.295339448	66.07924721	0.404880467
53.0253013	0.000448569	1.02977887	2.396825512	135.7678598	0.038128973
10.07599483	0.765699433	0.315126474	2.826034881	14.14867545	6931.52463
10.07599483	0.765699433	0.315126474	2.826034881	14.14867545	6931.52463
18568.80575	216.7719624	7.314889929	2.984262284	5.303856851	0.112210498
0.607917287	3.601276895	0.79238186	0.808034925	3.162938239	74.03553048
66.45741888	0.48027586	0.547940367	1.093570903	25.01397453	0.0155948
19.76773277	26.15553412	2.164307785	1.613844364	2.124361233	40.20781649
0.633821239	0.00030662	1.741000906	1.268339685	0.075508671	0.077116824
0.622512782	1.377303224	0.223313122	0.267778483	0.01953645	160.5929829
19.76773277	26.15553412	2.164307785	1.613844364	2.124361233	40.20781649
0.633821239	0.00030662	1.741000906	1.268339685	0.075508671	0.077116824
169.1928822	3.810743241	1.473957122	2.043511683	87877.79345	1.43004752
54.3281515	9.505708636	0.981578193	2.424928695	0.525099404	658.4281885
19.76773277	26.15553412	2.164307785	1.613844364	2.124361233	40.20781649
19.76773277	26.15553412	2.164307785	1.613844364	2.124361233	40.20781649
66.45741888	0.48027586	0.547940367	1.093570903	25.01397453	0.0155948

0.633821239	0.00030662	1.741000906	1.268339685	0.075508671	0.077116824
22.09800435	45129.47088	0.731099825	0.062816755	0.275597132	3.353155271
66.45741888	0.48027586	0.547940367	1.093570903	25.01397453	0.0155948
53.0253013	0.000448569	1.02977887	2.396825512	135.7678598	0.038128973
19.76773277	26.15553412	2.164307785	1.613844364	2.124361233	40.20781649
8.313131537	5.385900628	0.935531588	1.380779273	662.0914902	639.2113166
5.879186695	8.83174086	1.265719456	1.526789857	4.590098063	4589304.132
8.313131537	5.385900628	0.935531588	1.380779273	662.0914902	639.2113166
30.41401997	5.192032106	8.204517682	1.916424455	14.06887752	3668.158746
313461.5456	2.04668892	0.072055881	0.232985497	1.295160328	265252.0966
21.73776805	153.007792	0.633428826	1.626510537	0.006475321	0.213273606
1266.016198	136.166018	4.830015036	5.658542823	0.012961684	8.424915164
19.76773277	26.15553412	2.164307785	1.613844364	2.124361233	40.20781649
5.879186695	8.83174086	1.265719456	1.526789857	4.590098063	4589304.132
66.45741888	0.48027586	0.547940367	1.093570903	25.01397453	0.0155948
21.73776805	153.007792	0.633428826	1.626510537	0.006475321	0.213273606
22.54147642	2.490918001	0.428640274	0.742596221	0.004262236	19.27915906
107126.8596	0.123368538	1.227067142	0.910983175	0.752820883	0.028279923
18568.80575	216.7719624	7.314889929	2.984262284	5.303856851	0.112210498
0.633821239	0.00030662	1.741000906	1.268339685	0.075508671	0.077116824
53.0253013	0.000448569	1.02977887	2.396825512	135.7678598	0.038128973
1266.016198	136.166018	4.830015036	5.658542823	0.012961684	8.424915164
169.1928822	3.810743241	1.473957122	2.043511683	87877.79345	1.43004752
10.07599483	0.765699433	0.315126474	2.826034881	14.14867545	6931.52463
36.97841801	2.472742827	3.49391889	0.172082191	32.65910515	2.600578752
1266.016198	136.166018	4.830015036	5.658542823	0.012961684	8.424915164
66.45741888	0.48027586	0.547940367	1.093570903	25.01397453	0.0155948
66.45741888	0.48027586	0.547940367	1.093570903	25.01397453	0.0155948
5.879186695	8.83174086	1.265719456	1.526789857	4.590098063	4589304.132
66.45741888	0.48027586	0.547940367	1.093570903	25.01397453	0.0155948
21.73776805	153.007792	0.633428826	1.626510537	0.006475321	0.213273606
66.45741888	0.48027586	0.547940367	1.093570903	25.01397453	0.0155948
66.45741888	0.48027586	0.547940367	1.093570903	25.01397453	0.0155948
1266.016198	136.166018	4.830015036	5.658542823	0.012961684	8.424915164
1266.016198	136.166018	4.830015036	5.658542823	0.012961684	8.424915164
19.76773277	26.15553412	2.164307785	1.613844364	2.124361233	40.20781649
22.09800435	45129.47088	0.731099825	0.062816755	0.275597132	3.353155271
1266.016198	136.166018	4.830015036	5.658542823	0.012961684	8.424915164
36.97841801	2.472742827	3.49391889	0.172082191	32.65910515	2.600578752
66.45741888	0.48027586	0.547940367	1.093570903	25.01397453	0.0155948
19.76773277	26.15553412	2.164307785	1.613844364	2.124361233	40.20781649

21.73776805	153.007792	0.633428826	1.626510537	0.006475321	0.213273606
0.633821239	0.00030662	1.741000906	1.268339685	0.075508671	0.077116824
22.09800435	45129.47088	0.731099825	0.062816755	0.275597132	3.353155271
15.34601505	31813.10151	1.092970564	0.391240411	12.10629879	0.050273659
10.07599483	0.765699433	0.315126474	2.826034881	14.14867545	6931.52463
8.313131537	5.385900628	0.935531588	1.380779273	662.0914902	639.2113166
5.879186695	8.83174086	1.265719456	1.526789857	4.590098063	4589304.132
53.0253013	0.000448569	1.02977887	2.396825512	135.7678598	0.038128973
313461.5456	2.04668892	0.072055881	0.232985497	1.295160328	265252.0966
19.76773277	26.15553412	2.164307785	1.613844364	2.124361233	40.20781649
5.879186695	8.83174086	1.265719456	1.526789857	4.590098063	4589304.132
313461.5456	2.04668892	0.072055881	0.232985497	1.295160328	265252.0966
53.0253013	0.000448569	1.02977887	2.396825512	135.7678598	0.038128973
169.1928822	3.810743241	1.473957122	2.043511683	87877.79345	1.43004752
1.031468273	3.124178523	1.548182107	2.203696827	3.900025721	148.5739097
19.76773277	26.15553412	2.164307785	1.613844364	2.124361233	40.20781649
36.97841801	2.472742827	3.49391889	0.172082191	32.65910515	2.600578752
22.54147642	2.490918001	0.428640274	0.742596221	0.004262236	19.27915906
10.07599483	0.765699433	0.315126474	2.826034881	14.14867545	6931.52463
66.45741888	0.48027586	0.547940367	1.093570903	25.01397453	0.0155948
5.879186695	8.83174086	1.265719456	1.526789857	4.590098063	4589304.132
66.45741888	0.48027586	0.547940367	1.093570903	25.01397453	0.0155948
19.76773277	26.15553412	2.164307785	1.613844364	2.124361233	40.20781649
36.97841801	2.472742827	3.49391889	0.172082191	32.65910515	2.600578752
66.45741888	0.48027586	0.547940367	1.093570903	25.01397453	0.0155948
1266.016198	136.166018	4.830015036	5.658542823	0.012961684	8.424915164
313461.5456	2.04668892	0.072055881	0.232985497	1.295160328	265252.0966
313461.5456	2.04668892	0.072055881	0.232985497	1.295160328	265252.0966
266.2567944	23.73902711	0.246481843	2.57468402	103.108229	0.161420088
19.76773277	26.15553412	2.164307785	1.613844364	2.124361233	40.20781649
36.97841801	2.472742827	3.49391889	0.172082191	32.65910515	2.600578752
66.45741888	0.48027586	0.547940367	1.093570903	25.01397453	0.0155948
0.075725929	1.294090635	1.012868115	0.36654753	0.585910942	19.77707951
66.45741888	0.48027586	0.547940367	1.093570903	25.01397453	0.0155948
313461.5456	2.04668892	0.072055881	0.232985497	1.295160328	265252.0966
53.0253013	0.000448569	1.02977887	2.396825512	135.7678598	0.038128973
21.73776805	153.007792	0.633428826	1.626510537	0.006475321	0.213273606
1.031468273	3.124178523	1.548182107	2.203696827	3.900025721	148.5739097
19.76773277	26.15553412	2.164307785	1.613844364	2.124361233	40.20781649
66.45741888	0.48027586	0.547940367	1.093570903	25.01397453	0.0155948
1266.016198	136.166018	4.830015036	5.658542823	0.012961684	8.424915164

1266.016198	136.166018	4.830015036	5.658542823	0.012961684	8.424915164
19.76773277	26.15553412	2.164307785	1.613844364	2.124361233	40.20781649
66.45741888	0.48027586	0.547940367	1.093570903	25.01397453	0.0155948
0.075725929	1.294090635	1.012868115	0.36654753	0.585910942	19.77707951
66.45741888	0.48027586	0.547940367	1.093570903	25.01397453	0.0155948
66.45741888	0.48027586	0.547940367	1.093570903	25.01397453	0.0155948
1266.016198	136.166018	4.830015036	5.658542823	0.012961684	8.424915164
21.73776805	153.007792	0.633428826	1.626510537	0.006475321	0.213273606
19.76773277	26.15553412	2.164307785	1.613844364	2.124361233	40.20781649
53.0253013	0.000448569	1.02977887	2.396825512	135.7678598	0.038128973
22.09800435	45129.47088	0.731099825	0.062816755	0.275597132	3.353155271
1.183699882	1627.295113	2.288510658	2.116976239	0.050920618	0.034191149
66.45741888	0.48027586	0.547940367	1.093570903	25.01397453	0.0155948
1266.016198	136.166018	4.830015036	5.658542823	0.012961684	8.424915164
22.09800435	45129.47088	0.731099825	0.062816755	0.275597132	3.353155271
3035.266701	6740.552411	1.012714615	0.600211742	99447.90994	0.057341764
313461.5456	2.04668892	0.072055881	0.232985497	1.295160328	265252.0966
73.26665109	3.671444805	9.867576127	2.459204558	0.654044029	3585950013
21.73776805	153.007792	0.633428826	1.626510537	0.006475321	0.213273606
313461.5456	2.04668892	0.072055881	0.232985497	1.295160328	265252.0966
0.633821239	0.00030662	1.741000906	1.268339685	0.075508671	0.077116824
2.468255987	0.026418519	0.443674403	0.433507053	2.47E-05	66.9519551
23.15224205	12.48785503	7.317679096	11.69104729	1.828916419	490.1890688
169.1928822	3.810743241	1.473957122	2.043511683	87877.79345	1.43004752
19.76773277	26.15553412	2.164307785	1.613844364	2.124361233	40.20781649
22.54147642	2.490918001	0.428640274	0.742596221	0.004262236	19.27915906
0.633821239	0.00030662	1.741000906	1.268339685	0.075508671	0.077116824
10.07599483	0.765699433	0.315126474	2.826034881	14.14867545	6931.52463
107126.8596	0.123368538	1.227067142	0.910983175	0.752820883	0.028279923
0.075725929	1.294090635	1.012868115	0.36654753	0.585910942	19.77707951
53.0253013	0.000448569	1.02977887	2.396825512	135.7678598	0.038128973
10.07599483	0.765699433	0.315126474	2.826034881	14.14867545	6931.52463
19.76773277	26.15553412	2.164307785	1.613844364	2.124361233	40.20781649
23.15224205	12.48785503	7.317679096	11.69104729	1.828916419	490.1890688
266.2567944	23.73902711	0.246481843	2.57468402	103.108229	0.161420088
19.76773277	26.15553412	2.164307785	1.613844364	2.124361233	40.20781649
1.031468273	3.124178523	1.548182107	2.203696827	3.900025721	148.5739097
313461.5456	2.04668892	0.072055881	0.232985497	1.295160328	265252.0966
19.76773277	26.15553412	2.164307785	1.613844364	2.124361233	40.20781649
19.76773277	26.15553412	2.164307785	1.613844364	2.124361233	40.20781649
3035.266701	6740.552411	1.012714615	0.600211742	99447.90994	0.057341764

66.45741888	0.48027586	0.547940367	1.093570903	25.01397453	0.0155948
6.910631055	0.558508444	0.075786067	0.261486938	3.814890351	13.17679122
19.76773277	26.15553412	2.164307785	1.613844364	2.124361233	40.20781649
1266.016198	136.166018	4.830015036	5.658542823	0.012961684	8.424915164
53.0253013	0.000448569	1.02977887	2.396825512	135.7678598	0.038128973
5.879186695	8.83174086	1.265719456	1.526789857	4.590098063	4589304.132
313461.5456	2.04668892	0.072055881	0.232985497	1.295160328	265252.0966
19.76773277	26.15553412	2.164307785	1.613844364	2.124361233	40.20781649
1266.016198	136.166018	4.830015036	5.658542823	0.012961684	8.424915164
36.97841801	2.472742827	3.49391889	0.172082191	32.65910515	2.600578752
19.76773277	26.15553412	2.164307785	1.613844364	2.124361233	40.20781649
66.45741888	0.48027586	0.547940367	1.093570903	25.01397453	0.0155948
0.622512782	1.377303224	0.223313122	0.267778483	0.01953645	160.5929829
1266.016198	136.166018	4.830015036	5.658542823	0.012961684	8.424915164
19.76773277	26.15553412	2.164307785	1.613844364	2.124361233	40.20781649
19.76773277	26.15553412	2.164307785	1.613844364	2.124361233	40.20781649
36.97841801	2.472742827	3.49391889	0.172082191	32.65910515	2.600578752
1266.016198	136.166018	4.830015036	5.658542823	0.012961684	8.424915164
73.26665109	3.671444805	9.867576127	2.459204558	0.654044029	3585950013
2.468255987	0.026418519	0.443674403	0.433507053	2.47E-05	66.9519551
23.15224205	12.48785503	7.317679096	11.69104729	1.828916419	490.1890688
19.76773277	26.15553412	2.164307785	1.613844364	2.124361233	40.20781649
19.76773277	26.15553412	2.164307785	1.613844364	2.124361233	40.20781649
21.73776805	153.007792	0.633428826	1.626510537	0.006475321	0.213273606
0.633821239	0.00030662	1.741000906	1.268339685	0.075508671	0.077116824
0.607917287	3.601276895	0.79238186	0.808034925	3.162938239	74.03553048
22.09800435	45129.47088	0.731099825	0.062816755	0.275597132	3.353155271
0.000963319	0.285624349	0.585966806	0.5481098	4035.3477	0.210294443
1266.016198	136.166018	4.830015036	5.658542823	0.012961684	8.424915164
313461.5456	2.04668892	0.072055881	0.232985497	1.295160328	265252.0966
10.07599483	0.765699433	0.315126474	2.826034881	14.14867545	6931.52463
66.45741888	0.48027586	0.547940367	1.093570903	25.01397453	0.0155948
8.313131537	5.385900628	0.935531588	1.380779273	662.0914902	639.2113166
19.76773277	26.15553412	2.164307785	1.613844364	2.124361233	40.20781649
1266.016198	136.166018	4.830015036	5.658542823	0.012961684	8.424915164
66.45741888	0.48027586	0.547940367	1.093570903	25.01397453	0.0155948
22.09800435	45129.47088	0.731099825	0.062816755	0.275597132	3.353155271
2.367270739	1.636394145	0.199400213	0.266052104	0.059479575	41.8334753
19.76773277	26.15553412	2.164307785	1.613844364	2.124361233	40.20781649
19.76773277	26.15553412	2.164307785	1.613844364	2.124361233	40.20781649
19.76773277	26.15553412	2.164307785	1.613844364	2.124361233	40.20781649

53.0253013	0.000448569	1.02977887	2.396825512	135.7678598	0.038128973
30.41401997	5.192032106	8.204517682	1.916424455	14.06887752	3668.158746
66.45741888	0.48027586	0.547940367	1.093570903	25.01397453	0.0155948
19.76773277	26.15553412	2.164307785	1.613844364	2.124361233	40.20781649
266.2567944	23.73902711	0.246481843	2.57468402	103.108229	0.161420088
66.45741888	0.48027586	0.547940367	1.093570903	25.01397453	0.0155948
22.09800435	45129.47088	0.731099825	0.062816755	0.275597132	3.353155271
10.07599483	0.765699433	0.315126474	2.826034881	14.14867545	6931.52463
8.313131537	5.385900628	0.935531588	1.380779273	662.0914902	639.2113166
36.77273411	14.45513701	0.62100212	0.852578792	1760.282485	0.796748454
30.41401997	5.192032106	8.204517682	1.916424455	14.06887752	3668.158746
30.41401997	5.192032106	8.204517682	1.916424455	14.06887752	3668.158746
22.09800435	45129.47088	0.731099825	0.062816755	0.275597132	3.353155271
53.0253013	0.000448569	1.02977887	2.396825512	135.7678598	0.038128973
19.76773277	26.15553412	2.164307785	1.613844364	2.124361233	40.20781649
1266.016198	136.166018	4.830015036	5.658542823	0.012961684	8.424915164
19.76773277	26.15553412	2.164307785	1.613844364	2.124361233	40.20781649
1266.016198	136.166018	4.830015036	5.658542823	0.012961684	8.424915164
66.45741888	0.48027586	0.547940367	1.093570903	25.01397453	0.0155948
5.879186695	8.83174086	1.265719456	1.526789857	4.590098063	4589304.132
73.26665109	3.671444805	9.867576127	2.459204558	0.654044029	3585950013
0.033684265	0.02797494	0.331728952	0.250960669	8367.241909	0.004278054
1266.016198	136.166018	4.830015036	5.658542823	0.012961684	8.424915164
1266.016198	136.166018	4.830015036	5.658542823	0.012961684	8.424915164
30.41401997	5.192032106	8.204517682	1.916424455	14.06887752	3668.158746
22.54147642	2.490918001	0.428640274	0.742596221	0.004262236	19.27915906
1266.016198	136.166018	4.830015036	5.658542823	0.012961684	8.424915164
1266.016198	136.166018	4.830015036	5.658542823	0.012961684	8.424915164
21.73776805	153.007792	0.633428826	1.626510537	0.006475321	0.213273606
54.3281515	9.505708636	0.981578193	2.424928695	0.525099404	658.4281885
21.73776805	153.007792	0.633428826	1.626510537	0.006475321	0.213273606
21.73776805	153.007792	0.633428826	1.626510537	0.006475321	0.213273606
66.45741888	0.48027586	0.547940367	1.093570903	25.01397453	0.0155948
0.633821239	0.00030662	1.741000906	1.268339685	0.075508671	0.077116824
22.09800435	45129.47088	0.731099825	0.062816755	0.275597132	3.353155271
1266.016198	136.166018	4.830015036	5.658542823	0.012961684	8.424915164
23.15224205	12.48785503	7.317679096	11.69104729	1.828916419	490.1890688
266.2567944	23.73902711	0.246481843	2.57468402	103.108229	0.161420088
10.07599483	0.765699433	0.315126474	2.826034881	14.14867545	6931.52463
1266.016198	136.166018	4.830015036	5.658542823	0.012961684	8.424915164
19.76773277	26.15553412	2.164307785	1.613844364	2.124361233	40.20781649

30.41401997	5.192032106	8.204517682	1.916424455	14.06887752	3668.158746
1266.016198	136.166018	4.830015036	5.658542823	0.012961684	8.424915164
0.633821239	0.00030662	1.741000906	1.268339685	0.075508671	0.077116824
2.367270739	1.636394145	0.199400213	0.266052104	0.059479575	41.8334753
66.45741888	0.48027586	0.547940367	1.093570903	25.01397453	0.0155948
53.0253013	0.000448569	1.02977887	2.396825512	135.7678598	0.038128973
30.41401997	5.192032106	8.204517682	1.916424455	14.06887752	3668.158746
266.2567944	23.73902711	0.246481843	2.57468402	103.108229	0.161420088
22.54147642	2.490918001	0.428640274	0.742596221	0.004262236	19.27915906
22.54147642	2.490918001	0.428640274	0.742596221	0.004262236	19.27915906
5.879186695	8.83174086	1.265719456	1.526789857	4.590098063	4589304.132
21.73776805	153.007792	0.633428826	1.626510537	0.006475321	0.213273606
66.45741888	0.48027586	0.547940367	1.093570903	25.01397453	0.0155948
54.3281515	9.505708636	0.981578193	2.424928695	0.525099404	658.4281885
22.09800435	45129.47088	0.731099825	0.062816755	0.275597132	3.353155271
1.031468273	3.124178523	1.548182107	2.203696827	3.900025721	148.5739097
22.09800435	45129.47088	0.731099825	0.062816755	0.275597132	3.353155271
10.07599483	0.765699433	0.315126474	2.826034881	14.14867545	6931.52463
19.76773277	26.15553412	2.164307785	1.613844364	2.124361233	40.20781649
266.2567944	23.73902711	0.246481843	2.57468402	103.108229	0.161420088
169.1928822	3.810743241	1.473957122	2.043511683	87877.79345	1.43004752
66.45741888	0.48027586	0.547940367	1.093570903	25.01397453	0.0155948
266.2567944	23.73902711	0.246481843	2.57468402	103.108229	0.161420088
2.468255987	0.026418519	0.443674403	0.433507053	2.47E-05	66.9519551
19.76773277	26.15553412	2.164307785	1.613844364	2.124361233	40.20781649
53.0253013	0.000448569	1.02977887	2.396825512	135.7678598	0.038128973
36.97841801	2.472742827	3.49391889	0.172082191	32.65910515	2.600578752
1266.016198	136.166018	4.830015036	5.658542823	0.012961684	8.424915164
10.07599483	0.765699433	0.315126474	2.826034881	14.14867545	6931.52463
18568.80575	216.7719624	7.314889929	2.984262284	5.303856851	0.112210498
21.73776805	153.007792	0.633428826	1.626510537	0.006475321	0.213273606
313461.5456	2.04668892	0.072055881	0.232985497	1.295160328	265252.0966
1.031468273	3.124178523	1.548182107	2.203696827	3.900025721	148.5739097
66.45741888	0.48027586	0.547940367	1.093570903	25.01397453	0.0155948
1.183699882	1627.295113	2.288510658	2.116976239	0.050920618	0.034191149
10.07599483	0.765699433	0.315126474	2.826034881	14.14867545	6931.52463
1266.016198	136.166018	4.830015036	5.658542823	0.012961684	8.424915164
19.76773277	26.15553412	2.164307785	1.613844364	2.124361233	40.20781649
10.07599483	0.765699433	0.315126474	2.826034881	14.14867545	6931.52463
19.76773277	26.15553412	2.164307785	1.613844364	2.124361233	40.20781649
5.879186695	8.83174086	1.265719456	1.526789857	4.590098063	4589304.132

73.26665109	3.671444805	9.867576127	2.459204558	0.654044029	3585950013
22.09800435	45129.47088	0.731099825	0.062816755	0.275597132	3.353155271
54.3281515	9.505708636	0.981578193	2.424928695	0.525099404	658.4281885
10.07599483	0.765699433	0.315126474	2.826034881	14.14867545	6931.52463
53.0253013	0.000448569	1.02977887	2.396825512	135.7678598	0.038128973
30.41401997	5.192032106	8.204517682	1.916424455	14.06887752	3668.158746
19.76773277	26.15553412	2.164307785	1.613844364	2.124361233	40.20781649
66.45741888	0.48027586	0.547940367	1.093570903	25.01397453	0.0155948
66.45741888	0.48027586	0.547940367	1.093570903	25.01397453	0.0155948
6.910631055	0.558508444	0.075786067	0.261486938	3.814890351	13.17679122
107126.8596	0.123368538	1.227067142	0.910983175	0.752820883	0.028279923
10.07599483	0.765699433	0.315126474	2.826034881	14.14867545	6931.52463
19.76773277	26.15553412	2.164307785	1.613844364	2.124361233	40.20781649
1266.016198	136.166018	4.830015036	5.658542823	0.012961684	8.424915164
66.45741888	0.48027586	0.547940367	1.093570903	25.01397453	0.0155948
0.633821239	0.00030662	1.741000906	1.268339685	0.075508671	0.077116824
53.0253013	0.000448569	1.02977887	2.396825512	135.7678598	0.038128973
66.45741888	0.48027586	0.547940367	1.093570903	25.01397453	0.0155948
6.910631055	0.558508444	0.075786067	0.261486938	3.814890351	13.17679122
313461.5456	2.04668892	0.072055881	0.232985497	1.295160328	265252.0966
66.45741888	0.48027586	0.547940367	1.093570903	25.01397453	0.0155948
22.09800435	45129.47088	0.731099825	0.062816755	0.275597132	3.353155271
10.07599483	0.765699433	0.315126474	2.826034881	14.14867545	6931.52463
10.07599483	0.765699433	0.315126474	2.826034881	14.14867545	6931.52463
1.183699882	1627.295113	2.288510658	2.116976239	0.050920618	0.034191149
54.3281515	9.505708636	0.981578193	2.424928695	0.525099404	658.4281885
54.3281515	9.505708636	0.981578193	2.424928695	0.525099404	658.4281885
10.07599483	0.765699433	0.315126474	2.826034881	14.14867545	6931.52463
15.34601505	31813.10151	1.092970564	0.391240411	12.10629879	0.050273659
5.879186695	8.83174086	1.265719456	1.526789857	4.590098063	4589304.132
19.76773277	26.15553412	2.164307785	1.613844364	2.124361233	40.20781649
266.2567944	23.73902711	0.246481843	2.57468402	103.108229	0.161420088
0.607917287	3.601276895	0.79238186	0.808034925	3.162938239	74.03553048
0.075725929	1.294090635	1.012868115	0.36654753	0.585910942	19.77707951
6.910631055	0.558508444	0.075786067	0.261486938	3.814890351	13.17679122
0.607917287	3.601276895	0.79238186	0.808034925	3.162938239	74.03553048
66.45741888	0.48027586	0.547940367	1.093570903	25.01397453	0.0155948
0.000963319	0.285624349	0.585966806	0.5481098	4035.3477	0.210294443
19.76773277	26.15553412	2.164307785	1.613844364	2.124361233	40.20781649
0.000963319	0.285624349	0.585966806	0.5481098	4035.3477	0.210294443
19.76773277	26.15553412	2.164307785	1.613844364	2.124361233	40.20781649

66.45741888	0.48027586	0.547940367	1.093570903	25.01397453	0.0155948
66.45741888	0.48027586	0.547940367	1.093570903	25.01397453	0.0155948
19.76773277	26.15553412	2.164307785	1.613844364	2.124361233	40.20781649
66.45741888	0.48027586	0.547940367	1.093570903	25.01397453	0.0155948
1.031468273	3.124178523	1.548182107	2.203696827	3.900025721	148.5739097
19.76773277	26.15553412	2.164307785	1.613844364	2.124361233	40.20781649
19.76773277	26.15553412	2.164307785	1.613844364	2.124361233	40.20781649
0.033684265	0.02797494	0.331728952	0.250960669	8367.241909	0.004278054
36.77273411	14.45513701	0.62100212	0.852578792	1760.282485	0.796748454
0.622512782	1.377303224	0.223313122	0.267778483	0.01953645	160.5929829
107126.8596	0.123368538	1.227067142	0.910983175	0.752820883	0.028279923
313461.5456	2.04668892	0.072055881	0.232985497	1.295160328	265252.0966
194674.4558	0.798978841	0.496572546	1.627149415	83.23698734	20.14299098
169.1928822	3.810743241	1.473957122	2.043511683	87877.79345	1.43004752
15.34601505	31813.10151	1.092970564	0.391240411	12.10629879	0.050273659
10.07599483	0.765699433	0.315126474	2.826034881	14.14867545	6931.52463
22.09800435	45129.47088	0.731099825	0.062816755	0.275597132	3.353155271
66.45741888	0.48027586	0.547940367	1.093570903	25.01397453	0.0155948
266.2567944	23.73902711	0.246481843	2.57468402	103.108229	0.161420088
22.54147642	2.490918001	0.428640274	0.742596221	0.004262236	19.27915906
66.45741888	0.48027586	0.547940367	1.093570903	25.01397453	0.0155948
1.031468273	3.124178523	1.548182107	2.203696827	3.900025721	148.5739097
21.73776805	153.007792	0.633428826	1.626510537	0.006475321	0.213273606
66.45741888	0.48027586	0.547940367	1.093570903	25.01397453	0.0155948
19.76773277	26.15553412	2.164307785	1.613844364	2.124361233	40.20781649
22.54147642	2.490918001	0.428640274	0.742596221	0.004262236	19.27915906
1266.016198	136.166018	4.830015036	5.658542823	0.012961684	8.424915164
15.34601505	31813.10151	1.092970564	0.391240411	12.10629879	0.050273659
194674.4558	0.798978841	0.496572546	1.627149415	83.23698734	20.14299098
266.2567944	23.73902711	0.246481843	2.57468402	103.108229	0.161420088
10.07599483	0.765699433	0.315126474	2.826034881	14.14867545	6931.52463
21.73776805	153.007792	0.633428826	1.626510537	0.006475321	0.213273606
53.0253013	0.000448569	1.02977887	2.396825512	135.7678598	0.038128973
1.031468273	3.124178523	1.548182107	2.203696827	3.900025721	148.5739097
22.09800435	45129.47088	0.731099825	0.062816755	0.275597132	3.353155271
66.45741888	0.48027586	0.547940367	1.093570903	25.01397453	0.0155948
10.07599483	0.765699433	0.315126474	2.826034881	14.14867545	6931.52463
313461.5456	2.04668892	0.072055881	0.232985497	1.295160328	265252.0966
21.73776805	153.007792	0.633428826	1.626510537	0.006475321	0.213273606
1266.016198	136.166018	4.830015036	5.658542823	0.012961684	8.424915164
5.879186695	8.83174086	1.265719456	1.526789857	4.590098063	4589304.132

0.633821239	0.00030662	1.741000906	1.268339685	0.075508671	0.077116824
19.76773277	26.15553412	2.164307785	1.613844364	2.124361233	40.20781649
19.76773277	26.15553412	2.164307785	1.613844364	2.124361233	40.20781649
54.3281515	9.505708636	0.981578193	2.424928695	0.525099404	658.4281885
1266.016198	136.166018	4.830015036	5.658542823	0.012961684	8.424915164
1.183699882	1627.295113	2.288510658	2.116976239	0.050920618	0.034191149
66.45741888	0.48027586	0.547940367	1.093570903	25.01397453	0.0155948
66.45741888	0.48027586	0.547940367	1.093570903	25.01397453	0.0155948
23.15224205	12.48785503	7.317679096	11.69104729	1.828916419	490.1890688
19.76773277	26.15553412	2.164307785	1.613844364	2.124361233	40.20781649
66.45741888	0.48027586	0.547940367	1.093570903	25.01397453	0.0155948
10.07599483	0.765699433	0.315126474	2.826034881	14.14867545	6931.52463
266.2567944	23.73902711	0.246481843	2.57468402	103.108229	0.161420088
21.73776805	153.007792	0.633428826	1.626510537	0.006475321	0.213273606
66.45741888	0.48027586	0.547940367	1.093570903	25.01397453	0.0155948
1266.016198	136.166018	4.830015036	5.658542823	0.012961684	8.424915164
66.45741888	0.48027586	0.547940367	1.093570903	25.01397453	0.0155948
21.73776805	153.007792	0.633428826	1.626510537	0.006475321	0.213273606
0.000963319	0.285624349	0.585966806	0.5481098	4035.3477	0.210294443
10.07599483	0.765699433	0.315126474	2.826034881	14.14867545	6931.52463
266.2567944	23.73902711	0.246481843	2.57468402	103.108229	0.161420088
0.033684265	0.02797494	0.331728952	0.250960669	8367.241909	0.004278054
22.09800435	45129.47088	0.731099825	0.062816755	0.275597132	3.353155271
1266.016198	136.166018	4.830015036	5.658542823	0.012961684	8.424915164
21.73776805	153.007792	0.633428826	1.626510537	0.006475321	0.213273606
3.528750715	100.4526071	0.712168835	1.005018489	0.762780078	25.83373763
22.54147642	2.490918001	0.428640274	0.742596221	0.004262236	19.27915906
66.45741888	0.48027586	0.547940367	1.093570903	25.01397453	0.0155948
1266.016198	136.166018	4.830015036	5.658542823	0.012961684	8.424915164
30.41401997	5.192032106	8.204517682	1.916424455	14.06887752	3668.158746
19.76773277	26.15553412	2.164307785	1.613844364	2.124361233	40.20781649
66.45741888	0.48027586	0.547940367	1.093570903	25.01397453	0.0155948
36.77273411	14.45513701	0.62100212	0.852578792	1760.282485	0.796748454
66.45741888	0.48027586	0.547940367	1.093570903	25.01397453	0.0155948
53.0253013	0.000448569	1.02977887	2.396825512	135.7678598	0.038128973
21.73776805	153.007792	0.633428826	1.626510537	0.006475321	0.213273606
0.622512782	1.377303224	0.223313122	0.267778483	0.01953645	160.5929829
5.879186695	8.83174086	1.265719456	1.526789857	4.590098063	4589304.132
54.3281515	9.505708636	0.981578193	2.424928695	0.525099404	658.4281885
5.879186695	8.83174086	1.265719456	1.526789857	4.590098063	4589304.132
36.97841801	2.472742827	3.49391889	0.172082191	32.65910515	2.600578752

1266.016198	136.166018	4.830015036	5.658542823	0.012961684	8.424915164
21.73776805	153.007792	0.633428826	1.626510537	0.006475321	0.213273606
5.879186695	8.83174086	1.265719456	1.526789857	4.590098063	4589304.132
30.41401997	5.192032106	8.204517682	1.916424455	14.06887752	3668.158746
1266.016198	136.166018	4.830015036	5.658542823	0.012961684	8.424915164
66.45741888	0.48027586	0.547940367	1.093570903	25.01397453	0.0155948
169.1928822	3.810743241	1.473957122	2.043511683	87877.79345	1.43004752
53.0253013	0.000448569	1.02977887	2.396825512	135.7678598	0.038128973
30.41401997	5.192032106	8.204517682	1.916424455	14.06887752	3668.158746
19.76773277	26.15553412	2.164307785	1.613844364	2.124361233	40.20781649
53.0253013	0.000448569	1.02977887	2.396825512	135.7678598	0.038128973
53.0253013	0.000448569	1.02977887	2.396825512	135.7678598	0.038128973
10.07599483	0.765699433	0.315126474	2.826034881	14.14867545	6931.52463
101.1452473	14.00541805	7.254544628	2.613031417	176092.7785	1936408.462
66.45741888	0.48027586	0.547940367	1.093570903	25.01397453	0.0155948
23.15224205	12.48785503	7.317679096	11.69104729	1.828916419	490.1890688
66.45741888	0.48027586	0.547940367	1.093570903	25.01397453	0.0155948
66.45741888	0.48027586	0.547940367	1.093570903	25.01397453	0.0155948
19.76773277	26.15553412	2.164307785	1.613844364	2.124361233	40.20781649
66.45741888	0.48027586	0.547940367	1.093570903	25.01397453	0.0155948
73.26665109	3.671444805	9.867576127	2.459204558	0.654044029	3585950013
169.1928822	3.810743241	1.473957122	2.043511683	87877.79345	1.43004752
19.76773277	26.15553412	2.164307785	1.613844364	2.124361233	40.20781649
266.2567944	23.73902711	0.246481843	2.57468402	103.108229	0.161420088
2.468255987	0.026418519	0.443674403	0.433507053	2.47E-05	66.9519551
1266.016198	136.166018	4.830015036	5.658542823	0.012961684	8.424915164
22.09800435	45129.47088	0.731099825	0.062816755	0.275597132	3.353155271
66.45741888	0.48027586	0.547940367	1.093570903	25.01397453	0.0155948
19.76773277	26.15553412	2.164307785	1.613844364	2.124361233	40.20781649
30.41401997	5.192032106	8.204517682	1.916424455	14.06887752	3668.158746
19.76773277	26.15553412	2.164307785	1.613844364	2.124361233	40.20781649
101.1452473	14.00541805	7.254544628	2.613031417	176092.7785	1936408.462
3035.266701	6740.552411	1.012714615	0.600211742	99447.90994	0.057341764
66.45741888	0.48027586	0.547940367	1.093570903	25.01397453	0.0155948
1266.016198	136.166018	4.830015036	5.658542823	0.012961684	8.424915164
1266.016198	136.166018	4.830015036	5.658542823	0.012961684	8.424915164
22.09800435	45129.47088	0.731099825	0.062816755	0.275597132	3.353155271
266.2567944	23.73902711	0.246481843	2.57468402	103.108229	0.161420088
66.45741888	0.48027586	0.547940367	1.093570903	25.01397453	0.0155948
23.15224205	12.48785503	7.317679096	11.69104729	1.828916419	490.1890688
19.76773277	26.15553412	2.164307785	1.613844364	2.124361233	40.20781649

1266.016198	136.166018	4.830015036	5.658542823	0.012961684	8.424915164
21.73776805	153.007792	0.633428826	1.626510537	0.006475321	0.213273606
54.3281515	9.505708636	0.981578193	2.424928695	0.525099404	658.4281885
53.0253013	0.000448569	1.02977887	2.396825512	135.7678598	0.038128973
1266.016198	136.166018	4.830015036	5.658542823	0.012961684	8.424915164
22.09800435	45129.47088	0.731099825	0.062816755	0.275597132	3.353155271
23.15224205	12.48785503	7.317679096	11.69104729	1.828916419	490.1890688
66.45741888	0.48027586	0.547940367	1.093570903	25.01397453	0.0155948
30.41401997	5.192032106	8.204517682	1.916424455	14.06887752	3668.158746
1266.016198	136.166018	4.830015036	5.658542823	0.012961684	8.424915164
66.45741888	0.48027586	0.547940367	1.093570903	25.01397453	0.0155948
66.45741888	0.48027586	0.547940367	1.093570903	25.01397453	0.0155948
19.76773277	26.15553412	2.164307785	1.613844364	2.124361233	40.20781649
1266.016198	136.166018	4.830015036	5.658542823	0.012961684	8.424915164
21.73776805	153.007792	0.633428826	1.626510537	0.006475321	0.213273606
66.45741888	0.48027586	0.547940367	1.093570903	25.01397453	0.0155948
21.73776805	153.007792	0.633428826	1.626510537	0.006475321	0.213273606
30.41401997	5.192032106	8.204517682	1.916424455	14.06887752	3668.158746
1.031468273	3.124178523	1.548182107	2.203696827	3.900025721	148.5739097
30.41401997	5.192032106	8.204517682	1.916424455	14.06887752	3668.158746
1266.016198	136.166018	4.830015036	5.658542823	0.012961684	8.424915164
30.41401997	5.192032106	8.204517682	1.916424455	14.06887752	3668.158746
19.76773277	26.15553412	2.164307785	1.613844364	2.124361233	40.20781649
3035.266701	6740.552411	1.012714615	0.600211742	99447.90994	0.057341764
0.000963319	0.285624349	0.585966806	0.5481098	4035.3477	0.210294443
66.45741888	0.48027586	0.547940367	1.093570903	25.01397453	0.0155948
10.07599483	0.765699433	0.315126474	2.826034881	14.14867545	6931.52463
21.73776805	153.007792	0.633428826	1.626510537	0.006475321	0.213273606
54.3281515	9.505708636	0.981578193	2.424928695	0.525099404	658.4281885
21.73776805	153.007792	0.633428826	1.626510537	0.006475321	0.213273606
1266.016198	136.166018	4.830015036	5.658542823	0.012961684	8.424915164
53.0253013	0.000448569	1.02977887	2.396825512	135.7678598	0.038128973
3035.266701	6740.552411	1.012714615	0.600211742	99447.90994	0.057341764
54.3281515	9.505708636	0.981578193	2.424928695	0.525099404	658.4281885
22.09800435	45129.47088	0.731099825	0.062816755	0.275597132	3.353155271
10.07599483	0.765699433	0.315126474	2.826034881	14.14867545	6931.52463
22.09800435	45129.47088	0.731099825	0.062816755	0.275597132	3.353155271
1266.016198	136.166018	4.830015036	5.658542823	0.012961684	8.424915164
36.97841801	2.472742827	3.49391889	0.172082191	32.65910515	2.600578752
66.45741888	0.48027586	0.547940367	1.093570903	25.01397453	0.0155948
1266.016198	136.166018	4.830015036	5.658542823	0.012961684	8.424915164

10.07599483	0.765699433	0.315126474	2.826034881	14.14867545	6931.52463
66.45741888	0.48027586	0.547940367	1.093570903	25.01397453	0.0155948
19.76773277	26.15553412	2.164307785	1.613844364	2.124361233	40.20781649
2.468255987	0.026418519	0.443674403	0.433507053	2.47E-05	66.9519551
19.76773277	26.15553412	2.164307785	1.613844364	2.124361233	40.20781649
19.76773277	26.15553412	2.164307785	1.613844364	2.124361233	40.20781649
66.45741888	0.48027586	0.547940367	1.093570903	25.01397453	0.0155948
10.07599483	0.765699433	0.315126474	2.826034881	14.14867545	6931.52463
22.09800435	45129.47088	0.731099825	0.062816755	0.275597132	3.353155271
1266.016198	136.166018	4.830015036	5.658542823	0.012961684	8.424915164
66.45741888	0.48027586	0.547940367	1.093570903	25.01397453	0.0155948
1266.016198	136.166018	4.830015036	5.658542823	0.012961684	8.424915164
101.1452473	14.00541805	7.254544628	2.613031417	176092.7785	1936408.462
19.76773277	26.15553412	2.164307785	1.613844364	2.124361233	40.20781649
19.76773277	26.15553412	2.164307785	1.613844364	2.124361233	40.20781649
54.3281515	9.505708636	0.981578193	2.424928695	0.525099404	658.4281885
0.633821239	0.00030662	1.741000906	1.268339685	0.075508671	0.077116824
22.09800435	45129.47088	0.731099825	0.062816755	0.275597132	3.353155271
21.73776805	153.007792	0.633428826	1.626510537	0.006475321	0.213273606
107126.8596	0.123368538	1.227067142	0.910983175	0.752820883	0.028279923
1266.016198	136.166018	4.830015036	5.658542823	0.012961684	8.424915164
36.97841801	2.472742827	3.49391889	0.172082191	32.65910515	2.600578752
53.0253013	0.000448569	1.02977887	2.396825512	135.7678598	0.038128973
313461.5456	2.04668892	0.072055881	0.232985497	1.295160328	265252.0966
30.41401997	5.192032106	8.204517682	1.916424455	14.06887752	3668.158746
2.468255987	0.026418519	0.443674403	0.433507053	2.47E-05	66.9519551
3.528750715	100.4526071	0.712168835	1.005018489	0.762780078	25.83373763
22.54147642	2.490918001	0.428640274	0.742596221	0.004262236	19.27915906
194674.4558	0.798978841	0.496572546	1.627149415	83.23698734	20.14299098
1266.016198	136.166018	4.830015036	5.658542823	0.012961684	8.424915164
22.09800435	45129.47088	0.731099825	0.062816755	0.275597132	3.353155271
10.07599483	0.765699433	0.315126474	2.826034881	14.14867545	6931.52463
18568.80575	216.7719624	7.314889929	2.984262284	5.303856851	0.112210498
19.76773277	26.15553412	2.164307785	1.613844364	2.124361233	40.20781649
66.45741888	0.48027586	0.547940367	1.093570903	25.01397453	0.0155948
194674.4558	0.798978841	0.496572546	1.627149415	83.23698734	20.14299098
313461.5456	2.04668892	0.072055881	0.232985497	1.295160328	265252.0966
19.76773277	26.15553412	2.164307785	1.613844364	2.124361233	40.20781649
19.76773277	26.15553412	2.164307785	1.613844364	2.124361233	40.20781649
107126.8596	0.123368538	1.227067142	0.910983175	0.752820883	0.028279923
5.879186695	8.83174086	1.265719456	1.526789857	4.590098063	4589304.132

23.15224205	12.48785503	7.317679096	11.69104729	1.828916419	490.1890688
19.76773277	26.15553412	2.164307785	1.613844364	2.124361233	40.20781649
15.34601505	31813.10151	1.092970564	0.391240411	12.10629879	0.050273659
19.76773277	26.15553412	2.164307785	1.613844364	2.124361233	40.20781649
66.45741888	0.48027586	0.547940367	1.093570903	25.01397453	0.0155948
5.879186695	8.83174086	1.265719456	1.526789857	4.590098063	4589304.132
10.07599483	0.765699433	0.315126474	2.826034881	14.14867545	6931.52463
19.76773277	26.15553412	2.164307785	1.613844364	2.124361233	40.20781649
5.879186695	8.83174086	1.265719456	1.526789857	4.590098063	4589304.132
53.0253013	0.000448569	1.02977887	2.396825512	135.7678598	0.038128973
19.76773277	26.15553412	2.164307785	1.613844364	2.124361233	40.20781649
19.76773277	26.15553412	2.164307785	1.613844364	2.124361233	40.20781649
0.000963319	0.285624349	0.585966806	0.5481098	4035.3477	0.210294443
1266.016198	136.166018	4.830015036	5.658542823	0.012961684	8.424915164
23.15224205	12.48785503	7.317679096	11.69104729	1.828916419	490.1890688
1266.016198	136.166018	4.830015036	5.658542823	0.012961684	8.424915164
19.76773277	26.15553412	2.164307785	1.613844364	2.124361233	40.20781649
10.07599483	0.765699433	0.315126474	2.826034881	14.14867545	6931.52463
36.97841801	2.472742827	3.49391889	0.172082191	32.65910515	2.600578752
66.45741888	0.48027586	0.547940367	1.093570903	25.01397453	0.0155948
2.468255987	0.026418519	0.443674403	0.433507053	2.47E-05	66.9519551
54.3281515	9.505708636	0.981578193	2.424928695	0.525099404	658.4281885
1266.016198	136.166018	4.830015036	5.658542823	0.012961684	8.424915164
10.07599483	0.765699433	0.315126474	2.826034881	14.14867545	6931.52463
19.76773277	26.15553412	2.164307785	1.613844364	2.124361233	40.20781649
19.76773277	26.15553412	2.164307785	1.613844364	2.124361233	40.20781649
66.45741888	0.48027586	0.547940367	1.093570903	25.01397453	0.0155948
66.45741888	0.48027586	0.547940367	1.093570903	25.01397453	0.0155948
66.45741888	0.48027586	0.547940367	1.093570903	25.01397453	0.0155948
30.41401997	5.192032106	8.204517682	1.916424455	14.06887752	3668.158746
66.45741888	0.48027586	0.547940367	1.093570903	25.01397453	0.0155948
54.3281515	9.505708636	0.981578193	2.424928695	0.525099404	658.4281885
19.76773277	26.15553412	2.164307785	1.613844364	2.124361233	40.20781649
19.76773277	26.15553412	2.164307785	1.613844364	2.124361233	40.20781649
2.468255987	0.026418519	0.443674403	0.433507053	2.47E-05	66.9519551
313461.5456	2.04668892	0.072055881	0.232985497	1.295160328	265252.0966
22.54147642	2.490918001	0.428640274	0.742596221	0.004262236	19.27915906
0.607917287	3.601276895	0.79238186	0.808034925	3.162938239	74.03553048
169.1928822	3.810743241	1.473957122	2.043511683	87877.79345	1.43004752
5.879186695	8.83174086	1.265719456	1.526789857	4.590098063	4589304.132
66.45741888	0.48027586	0.547940367	1.093570903	25.01397453	0.0155948

54.3281515	9.505708636	0.981578193	2.424928695	0.525099404	658.4281885
53.0253013	0.000448569	1.02977887	2.396825512	135.7678598	0.038128973
53.0253013	0.000448569	1.02977887	2.396825512	135.7678598	0.038128973
53.0253013	0.000448569	1.02977887	2.396825512	135.7678598	0.038128973
19.76773277	26.15553412	2.164307785	1.613844364	2.124361233	40.20781649
66.45741888	0.48027586	0.547940367	1.093570903	25.01397453	0.0155948
19.76773277	26.15553412	2.164307785	1.613844364	2.124361233	40.20781649
1266.016198	136.166018	4.830015036	5.658542823	0.012961684	8.424915164
5.879186695	8.83174086	1.265719456	1.526789857	4.590098063	4589304.132
1266.016198	136.166018	4.830015036	5.658542823	0.012961684	8.424915164
169.1928822	3.810743241	1.473957122	2.043511683	87877.79345	1.43004752
66.45741888	0.48027586	0.547940367	1.093570903	25.01397453	0.0155948
19.76773277	26.15553412	2.164307785	1.613844364	2.124361233	40.20781649
21.73776805	153.007792	0.633428826	1.626510537	0.006475321	0.213273606
1266.016198	136.166018	4.830015036	5.658542823	0.012961684	8.424915164
6.910631055	0.558508444	0.075786067	0.261486938	3.814890351	13.17679122
66.45741888	0.48027586	0.547940367	1.093570903	25.01397453	0.0155948
66.45741888	0.48027586	0.547940367	1.093570903	25.01397453	0.0155948
21.73776805	153.007792	0.633428826	1.626510537	0.006475321	0.213273606
36.97841801	2.472742827	3.49391889	0.172082191	32.65910515	2.600578752
66.45741888	0.48027586	0.547940367	1.093570903	25.01397453	0.0155948
18568.80575	216.7719624	7.314889929	2.984262284	5.303856851	0.112210498
1266.016198	136.166018	4.830015036	5.658542823	0.012961684	8.424915164
22.09800435	45129.47088	0.731099825	0.062816755	0.275597132	3.353155271
21.73776805	153.007792	0.633428826	1.626510537	0.006475321	0.213273606
0.000963319	0.285624349	0.585966806	0.5481098	4035.3477	0.210294443
66.45741888	0.48027586	0.547940367	1.093570903	25.01397453	0.0155948
1.031468273	3.124178523	1.548182107	2.203696827	3.900025721	148.5739097
0.633821239	0.00030662	1.741000906	1.268339685	0.075508671	0.077116824
1266.016198	136.166018	4.830015036	5.658542823	0.012961684	8.424915164
5.879186695	8.83174086	1.265719456	1.526789857	4.590098063	4589304.132
53.0253013	0.000448569	1.02977887	2.396825512	135.7678598	0.038128973
1266.016198	136.166018	4.830015036	5.658542823	0.012961684	8.424915164
66.45741888	0.48027586	0.547940367	1.093570903	25.01397453	0.0155948
1.183699882	1627.295113	2.288510658	2.116976239	0.050920618	0.034191149
19.76773277	26.15553412	2.164307785	1.613844364	2.124361233	40.20781649
23.15224205	12.48785503	7.317679096	11.69104729	1.828916419	490.1890688
0.622512782	1.377303224	0.223313122	0.267778483	0.01953645	160.5929829
66.45741888	0.48027586	0.547940367	1.093570903	25.01397453	0.0155948
19.76773277	26.15553412	2.164307785	1.613844364	2.124361233	40.20781649
54.3281515	9.505708636	0.981578193	2.424928695	0.525099404	658.4281885

66.45741888	0.48027586	0.547940367	1.093570903	25.01397453	0.0155948
0.075725929	1.294090635	1.012868115	0.36654753	0.585910942	19.77707951
22.09800435	45129.47088	0.731099825	0.062816755	0.275597132	3.353155271
19.76773277	26.15553412	2.164307785	1.613844364	2.124361233	40.20781649
66.45741888	0.48027586	0.547940367	1.093570903	25.01397453	0.0155948
1266.016198	136.166018	4.830015036	5.658542823	0.012961684	8.424915164
1266.016198	136.166018	4.830015036	5.658542823	0.012961684	8.424915164
1266.016198	136.166018	4.830015036	5.658542823	0.012961684	8.424915164
66.45741888	0.48027586	0.547940367	1.093570903	25.01397453	0.0155948
19.76773277	26.15553412	2.164307785	1.613844364	2.124361233	40.20781649
66.45741888	0.48027586	0.547940367	1.093570903	25.01397453	0.0155948
66.45741888	0.48027586	0.547940367	1.093570903	25.01397453	0.0155948
19.76773277	26.15553412	2.164307785	1.613844364	2.124361233	40.20781649
6.910631055	0.558508444	0.075786067	0.261486938	3.814890351	13.17679122
1.183699882	1627.295113	2.288510658	2.116976239	0.050920618	0.034191149
1266.016198	136.166018	4.830015036	5.658542823	0.012961684	8.424915164
66.45741888	0.48027586	0.547940367	1.093570903	25.01397453	0.0155948
19.76773277	26.15553412	2.164307785	1.613844364	2.124361233	40.20781649
19.76773277	26.15553412	2.164307785	1.613844364	2.124361233	40.20781649
36.97841801	2.472742827	3.49391889	0.172082191	32.65910515	2.600578752
19.76773277	26.15553412	2.164307785	1.613844364	2.124361233	40.20781649
266.2567944	23.73902711	0.246481843	2.57468402	103.108229	0.161420088
169.1928822	3.810743241	1.473957122	2.043511683	87877.79345	1.43004752
66.45741888	0.48027586	0.547940367	1.093570903	25.01397453	0.0155948
5.879186695	8.83174086	1.265719456	1.526789857	4.590098063	4589304.132
66.45741888	0.48027586	0.547940367	1.093570903	25.01397453	0.0155948
66.45741888	0.48027586	0.547940367	1.093570903	25.01397453	0.0155948
3.528750715	100.4526071	0.712168835	1.005018489	0.762780078	25.83373763
54.3281515	9.505708636	0.981578193	2.424928695	0.525099404	658.4281885
53.0253013	0.000448569	1.02977887	2.396825512	135.7678598	0.038128973
19.76773277	26.15553412	2.164307785	1.613844364	2.124361233	40.20781649
0.633821239	0.00030662	1.741000906	1.268339685	0.075508671	0.077116824
1.031468273	3.124178523	1.548182107	2.203696827	3.900025721	148.5739097
313461.5456	2.04668892	0.072055881	0.232985497	1.295160328	265252.0966
1266.016198	136.166018	4.830015036	5.658542823	0.012961684	8.424915164
19.76773277	26.15553412	2.164307785	1.613844364	2.124361233	40.20781649
1266.016198	136.166018	4.830015036	5.658542823	0.012961684	8.424915164
0.075725929	1.294090635	1.012868115	0.36654753	0.585910942	19.77707951
266.2567944	23.73902711	0.246481843	2.57468402	103.108229	0.161420088
66.45741888	0.48027586	0.547940367	1.093570903	25.01397453	0.0155948
36.97841801	2.472742827	3.49391889	0.172082191	32.65910515	2.600578752

1.031468273	3.124178523	1.548182107	2.203696827	3.900025721	148.5739097
66.45741888	0.48027586	0.547940367	1.093570903	25.01397453	0.0155948
1266.016198	136.166018	4.830015036	5.658542823	0.012961684	8.424915164
15.34601505	31813.10151	1.092970564	0.391240411	12.10629879	0.050273659
19.76773277	26.15553412	2.164307785	1.613844364	2.124361233	40.20781649
1266.016198	136.166018	4.830015036	5.658542823	0.012961684	8.424915164
1266.016198	136.166018	4.830015036	5.658542823	0.012961684	8.424915164
36.77273411	14.45513701	0.62100212	0.852578792	1760.282485	0.796748454
36.97841801	2.472742827	3.49391889	0.172082191	32.65910515	2.600578752
53.0253013	0.000448569	1.02977887	2.396825512	135.7678598	0.038128973
19.76773277	26.15553412	2.164307785	1.613844364	2.124361233	40.20781649
66.45741888	0.48027586	0.547940367	1.093570903	25.01397453	0.0155948
19.76773277	26.15553412	2.164307785	1.613844364	2.124361233	40.20781649
169.1928822	3.810743241	1.473957122	2.043511683	87877.79345	1.43004752
313461.5456	2.04668892	0.072055881	0.232985497	1.295160328	265252.0966
1266.016198	136.166018	4.830015036	5.658542823	0.012961684	8.424915164
22.09800435	45129.47088	0.731099825	0.062816755	0.275597132	3.353155271
54.3281515	9.505708636	0.981578193	2.424928695	0.525099404	658.4281885
2.367270739	1.636394145	0.199400213	0.266052104	0.059479575	41.8334753
194674.4558	0.798978841	0.496572546	1.627149415	83.23698734	20.14299098
0.607917287	3.601276895	0.79238186	0.808034925	3.162938239	74.03553048
23.15224205	12.48785503	7.317679096	11.69104729	1.828916419	490.1890688
21.73776805	153.007792	0.633428826	1.626510537	0.006475321	0.213273606
0.000963319	0.285624349	0.585966806	0.5481098	4035.3477	0.210294443
66.45741888	0.48027586	0.547940367	1.093570903	25.01397453	0.0155948
19.76773277	26.15553412	2.164307785	1.613844364	2.124361233	40.20781649
313461.5456	2.04668892	0.072055881	0.232985497	1.295160328	265252.0966
5.879186695	8.83174086	1.265719456	1.526789857	4.590098063	4589304.132
22.09800435	45129.47088	0.731099825	0.062816755	0.275597132	3.353155271
1266.016198	136.166018	4.830015036	5.658542823	0.012961684	8.424915164
1266.016198	136.166018	4.830015036	5.658542823	0.012961684	8.424915164
66.45741888	0.48027586	0.547940367	1.093570903	25.01397453	0.0155948
19.76773277	26.15553412	2.164307785	1.613844364	2.124361233	40.20781649
54.3281515	9.505708636	0.981578193	2.424928695	0.525099404	658.4281885
15.34601505	31813.10151	1.092970564	0.391240411	12.10629879	0.050273659
0.000235526	0.42445576	0.130664491	1.295339448	66.07924721	0.404880467
66.45741888	0.48027586	0.547940367	1.093570903	25.01397453	0.0155948
19.76773277	26.15553412	2.164307785	1.613844364	2.124361233	40.20781649
30.41401997	5.192032106	8.204517682	1.916424455	14.06887752	3668.158746
19.76773277	26.15553412	2.164307785	1.613844364	2.124361233	40.20781649
10.07599483	0.765699433	0.315126474	2.826034881	14.14867545	6931.52463

21.73776805	153.007792	0.633428826	1.626510537	0.006475321	0.213273606
1266.016198	136.166018	4.830015036	5.658542823	0.012961684	8.424915164
54.3281515	9.505708636	0.981578193	2.424928695	0.525099404	658.4281885
22.09800435	45129.47088	0.731099825	0.062816755	0.275597132	3.353155271
169.1928822	3.810743241	1.473957122	2.043511683	87877.79345	1.43004752
66.45741888	0.48027586	0.547940367	1.093570903	25.01397453	0.0155948
53.0253013	0.000448569	1.02977887	2.396825512	135.7678598	0.038128973
0.607917287	3.601276895	0.79238186	0.808034925	3.162938239	74.03553048
313461.5456	2.04668892	0.072055881	0.232985497	1.295160328	265252.0966
10.07599483	0.765699433	0.315126474	2.826034881	14.14867545	6931.52463
22.09800435	45129.47088	0.731099825	0.062816755	0.275597132	3.353155271
3.528750715	100.4526071	0.712168835	1.005018489	0.762780078	25.83373763
53.0253013	0.000448569	1.02977887	2.396825512	135.7678598	0.038128973
1.183699882	1627.295113	2.288510658	2.116976239	0.050920618	0.034191149
19.76773277	26.15553412	2.164307785	1.613844364	2.124361233	40.20781649
53.0253013	0.000448569	1.02977887	2.396825512	135.7678598	0.038128973
19.76773277	26.15553412	2.164307785	1.613844364	2.124361233	40.20781649
22.09800435	45129.47088	0.731099825	0.062816755	0.275597132	3.353155271
1266.016198	136.166018	4.830015036	5.658542823	0.012961684	8.424915164
22.09800435	45129.47088	0.731099825	0.062816755	0.275597132	3.353155271
66.45741888	0.48027586	0.547940367	1.093570903	25.01397453	0.0155948
1266.016198	136.166018	4.830015036	5.658542823	0.012961684	8.424915164
66.45741888	0.48027586	0.547940367	1.093570903	25.01397453	0.0155948
0.633821239	0.00030662	1.741000906	1.268339685	0.075508671	0.077116824
22.09800435	45129.47088	0.731099825	0.062816755	0.275597132	3.353155271
1.031468273	3.124178523	1.548182107	2.203696827	3.900025721	148.5739097
23.15224205	12.48785503	7.317679096	11.69104729	1.828916419	490.1890688
1266.016198	136.166018	4.830015036	5.658542823	0.012961684	8.424915164
21.73776805	153.007792	0.633428826	1.626510537	0.006475321	0.213273606
1266.016198	136.166018	4.830015036	5.658542823	0.012961684	8.424915164
0.633821239	0.00030662	1.741000906	1.268339685	0.075508671	0.077116824
22.09800435	45129.47088	0.731099825	0.062816755	0.275597132	3.353155271
107126.8596	0.123368538	1.227067142	0.910983175	0.752820883	0.028279923
313461.5456	2.04668892	0.072055881	0.232985497	1.295160328	265252.0966
3.528750715	100.4526071	0.712168835	1.005018489	0.762780078	25.83373763
313461.5456	2.04668892	0.072055881	0.232985497	1.295160328	265252.0966
66.45741888	0.48027586	0.547940367	1.093570903	25.01397453	0.0155948
0.000235526	0.42445576	0.130664491	1.295339448	66.07924721	0.404880467
22.09800435	45129.47088	0.731099825	0.062816755	0.275597132	3.353155271
5.879186695	8.83174086	1.265719456	1.526789857	4.590098063	4589304.132
266.2567944	23.73902711	0.246481843	2.57468402	103.108229	0.161420088

0.000235526	0.42445576	0.130664491	1.295339448	66.07924721	0.404880467
0.000963319	0.285624349	0.585966806	0.5481098	4035.3477	0.210294443
19.76773277	26.15553412	2.164307785	1.613844364	2.124361233	40.20781649
19.76773277	26.15553412	2.164307785	1.613844364	2.124361233	40.20781649
66.45741888	0.48027586	0.547940367	1.093570903	25.01397453	0.0155948
107126.8596	0.123368538	1.227067142	0.910983175	0.752820883	0.028279923
30.41401997	5.192032106	8.204517682	1.916424455	14.06887752	3668.158746
157.421691	5.917306608	3.674731809	4.837610608	0.253877466	83.0582072
21.73776805	153.007792	0.633428826	1.626510537	0.006475321	0.213273606
107126.8596	0.123368538	1.227067142	0.910983175	0.752820883	0.028279923
0.927904551	13.09900442	3.821802563	1.653896965	9827.705967	619348.9686
19.76773277	26.15553412	2.164307785	1.613844364	2.124361233	40.20781649
54.3281515	9.505708636	0.981578193	2.424928695	0.525099404	658.4281885
0.622512782	1.377303224	0.223313122	0.267778483	0.01953645	160.5929829
101.1452473	14.00541805	7.254544628	2.613031417	176092.7785	1936408.462
107126.8596	0.123368538	1.227067142	0.910983175	0.752820883	0.028279923
266.2567944	23.73902711	0.246481843	2.57468402	103.108229	0.161420088
22.09800435	45129.47088	0.731099825	0.062816755	0.275597132	3.353155271
19.76773277	26.15553412	2.164307785	1.613844364	2.124361233	40.20781649
3035.266701	6740.552411	1.012714615	0.600211742	99447.90994	0.057341764
1266.016198	136.166018	4.830015036	5.658542823	0.012961684	8.424915164
5.879186695	8.83174086	1.265719456	1.526789857	4.590098063	4589304.132
66.45741888	0.48027586	0.547940367	1.093570903	25.01397453	0.0155948
22.54147642	2.490918001	0.428640274	0.742596221	0.004262236	19.27915906
53.0253013	0.000448569	1.02977887	2.396825512	135.7678598	0.038128973
10.07599483	0.765699433	0.315126474	2.826034881	14.14867545	6931.52463
18568.80575	216.7719624	7.314889929	2.984262284	5.303856851	0.112210498
1266.016198	136.166018	4.830015036	5.658542823	0.012961684	8.424915164
5.879186695	8.83174086	1.265719456	1.526789857	4.590098063	4589304.132
10.07599483	0.765699433	0.315126474	2.826034881	14.14867545	6931.52463
22.09800435	45129.47088	0.731099825	0.062816755	0.275597132	3.353155271
107126.8596	0.123368538	1.227067142	0.910983175	0.752820883	0.028279923
5.879186695	8.83174086	1.265719456	1.526789857	4.590098063	4589304.132
0.000235526	0.42445576	0.130664491	1.295339448	66.07924721	0.404880467
157.421691	5.917306608	3.674731809	4.837610608	0.253877466	83.0582072
107126.8596	0.123368538	1.227067142	0.910983175	0.752820883	0.028279923
19.76773277	26.15553412	2.164307785	1.613844364	2.124361233	40.20781649
1.031468273	3.124178523	1.548182107	2.203696827	3.900025721	148.5739097
107126.8596	0.123368538	1.227067142	0.910983175	0.752820883	0.028279923
53.0253013	0.000448569	1.02977887	2.396825512	135.7678598	0.038128973
19.76773277	26.15553412	2.164307785	1.613844364	2.124361233	40.20781649

1266.016198	136.166018	4.830015036	5.658542823	0.012961684	8.424915164
3035.266701	6740.552411	1.012714615	0.600211742	99447.90994	0.057341764
10.07599483	0.765699433	0.315126474	2.826034881	14.14867545	6931.52463
1.183699882	1627.295113	2.288510658	2.116976239	0.050920618	0.034191149
169.1928822	3.810743241	1.473957122	2.043511683	87877.79345	1.43004752
22.54147642	2.490918001	0.428640274	0.742596221	0.004262236	19.27915906
36.97841801	2.472742827	3.49391889	0.172082191	32.65910515	2.600578752
53.0253013	0.000448569	1.02977887	2.396825512	135.7678598	0.038128973
53.0253013	0.000448569	1.02977887	2.396825512	135.7678598	0.038128973
66.45741888	0.48027586	0.547940367	1.093570903	25.01397453	0.0155948
5.879186695	8.83174086	1.265719456	1.526789857	4.590098063	4589304.132
10.07599483	0.765699433	0.315126474	2.826034881	14.14867545	6931.52463
266.2567944	23.73902711	0.246481843	2.57468402	103.108229	0.161420088
1266.016198	136.166018	4.830015036	5.658542823	0.012961684	8.424915164
169.1928822	3.810743241	1.473957122	2.043511683	87877.79345	1.43004752
66.45741888	0.48027586	0.547940367	1.093570903	25.01397453	0.0155948
1266.016198	136.166018	4.830015036	5.658542823	0.012961684	8.424915164
8.313131537	5.385900628	0.935531588	1.380779273	662.0914902	639.2113166
36.77273411	14.45513701	0.62100212	0.852578792	1760.282485	0.796748454
169.1928822	3.810743241	1.473957122	2.043511683	87877.79345	1.43004752
194674.4558	0.798978841	0.496572546	1.627149415	83.23698734	20.14299098
266.2567944	23.73902711	0.246481843	2.57468402	103.108229	0.161420088
36.97841801	2.472742827	3.49391889	0.172082191	32.65910515	2.600578752
21.73776805	153.007792	0.633428826	1.626510537	0.006475321	0.213273606
101.1452473	14.00541805	7.254544628	2.613031417	176092.7785	1936408.462
313461.5456	2.04668892	0.072055881	0.232985497	1.295160328	265252.0966
10.07599483	0.765699433	0.315126474	2.826034881	14.14867545	6931.52463
54.3281515	9.505708636	0.981578193	2.424928695	0.525099404	658.4281885
19.76773277	26.15553412	2.164307785	1.613844364	2.124361233	40.20781649
15.34601505	31813.10151	1.092970564	0.391240411	12.10629879	0.050273659
6.910631055	0.558508444	0.075786067	0.261486938	3.814890351	13.17679122
1266.016198	136.166018	4.830015036	5.658542823	0.012961684	8.424915164
266.2567944	23.73902711	0.246481843	2.57468402	103.108229	0.161420088
10.07599483	0.765699433	0.315126474	2.826034881	14.14867545	6931.52463
10.07599483	0.765699433	0.315126474	2.826034881	14.14867545	6931.52463
194674.4558	0.798978841	0.496572546	1.627149415	83.23698734	20.14299098
5.879186695	8.83174086	1.265719456	1.526789857	4.590098063	4589304.132
0.000235526	0.42445576	0.130664491	1.295339448	66.07924721	0.404880467
1.183699882	1627.295113	2.288510658	2.116976239	0.050920618	0.034191149
53.0253013	0.000448569	1.02977887	2.396825512	135.7678598	0.038128973
0.622512782	1.377303224	0.223313122	0.267778483	0.01953645	160.5929829

0.927904551	13.09900442	3.821802563	1.653896965	9827.705967	619348.9686
1266.016198	136.166018	4.830015036	5.658542823	0.012961684	8.424915164
54.3281515	9.505708636	0.981578193	2.424928695	0.525099404	658.4281885
53.0253013	0.000448569	1.02977887	2.396825512	135.7678598	0.038128973
1266.016198	136.166018	4.830015036	5.658542823	0.012961684	8.424915164
21.73776805	153.007792	0.633428826	1.626510537	0.006475321	0.213273606
107126.8596	0.123368538	1.227067142	0.910983175	0.752820883	0.028279923
5.879186695	8.83174086	1.265719456	1.526789857	4.590098063	4589304.132
266.2567944	23.73902711	0.246481843	2.57468402	103.108229	0.161420088
66.45741888	0.48027586	0.547940367	1.093570903	25.01397453	0.0155948
5.879186695	8.83174086	1.265719456	1.526789857	4.590098063	4589304.132
19.76773277	26.15553412	2.164307785	1.613844364	2.124361233	40.20781649
0.622512782	1.377303224	0.223313122	0.267778483	0.01953645	160.5929829
53.0253013	0.000448569	1.02977887	2.396825512	135.7678598	0.038128973
22.54147642	2.490918001	0.428640274	0.742596221	0.004262236	19.27915906
66.45741888	0.48027586	0.547940367	1.093570903	25.01397453	0.0155948
22.54147642	2.490918001	0.428640274	0.742596221	0.004262236	19.27915906
66.45741888	0.48027586	0.547940367	1.093570903	25.01397453	0.0155948
33	34	35	36	37	38
DOP1B-Hs01123267_g1	C21orf33-Hs01105802_g1	ADAMTS5-Hs04272736_s1	CXADR-Hs04194411_s1	NCAM2-Hs01562292_m1	UBASH3A-Hs00955169_m1

39	40	41	42	43	44
UBASH3A-Hs00955168_m1	PFKL-Hs01040525_m1	CHODL-Hs01070471_m1	PKNOX1-Hs01007098_m1	PKNOX1-Hs01007097_m1	PKNOX1-Hs01007094_m1
1.340246505	0.037727178	22.24294908	54163.1928	2805.536892	522.2411957
10.7410827	0.036806185	51.32945585	9.18E-05	0.000753104	0.03904239
0.049330724	6.494228425	2006.898641	13153.45828	638094.5286	0.001711311
0.037019532	4.873500192	0.125945205	16644.86089	4241.220028	149.1083909
0.000766925	99.84334823	0.002609179	213.9597422	139344.0229	1.067310431
0.010897266	20360.61434	296.4509364	18.93484545	54.36227445	39811.41253
5815.143759	1.23029477	268.6491856	3626.891332	609418.3417	54.45138655
0.000445561	0.058656597	24.10925189	304.7308484	0.001200193	12.16732101
9.495484129	0.150712221	8.360938635	100503.0287	0.003083775	146936.7895
0.005258125	4881.725445	0.017888818	85.29141643	15.26807993	11.60583563
0.005372635	0.707289771	0.018278394	8.183222756	21.8821969	0.116524173
0.037019532	4.873500192	0.125945205	16644.86089	4241.220028	149.1083909
78695.91365	222.9287439	747.4071534	39377322.27	88.30447304	37.38085013
9.495484129	0.150712221	8.360938635	100503.0287	0.003083775	146936.7895
1.215805511	732575809.3	5542.944676	37.54586964	0.016690416	67.58598473
1338171.278	0.24839217	0.006419165	13.71075529	4.180106984	6.544240634
17404079.42	13489.67892	281.8369522	864.215896	60.95897116	0.000228903
30083.40983	0.206274802	1.834688746	0.000514505	41.98164808	11.44786483
0.049330724	6.494228425	2006.898641	13153.45828	638094.5286	0.001711311
34.05858338	3.164024444	59.62864248	1320931.182	0.064740194	0.513731545
1338171.278	0.24839217	0.006419165	13.71075529	4.180106984	6.544240634
1041539.983	1.153413002	0.029807496	0.002876925	99.50464022	0.000303939
89.48930252	300.6529558	856.1172773	11558.44177	413.5091331	366.3331542
0.005372635	0.707289771	0.018278394	8.183222756	21.8821969	0.116524173
0.000766925	99.84334823	0.002609179	213.9597422	139344.0229	1.067310431
89.48930252	300.6529558	856.1172773	11558.44177	413.5091331	366.3331542
0.005258125	4881.725445	0.017888818	85.29141643	15.26807993	11.60583563
0.005372635	0.707289771	0.018278394	8.183222756	21.8821969	0.116524173
1.215805511	732575809.3	5542.944676	37.54586964	0.016690416	67.58598473
9.495484129	0.150712221	8.360938635	100503.0287	0.003083775	146936.7895
1.340246505	0.037727178	22.24294908	54163.1928	2805.536892	522.2411957
23.51452227	1.584496825	793.3575673	10465.1586	1159.149343	158.7182674
1338171.278	0.24839217	0.006419165	13.71075529	4.180106984	6.544240634
0.010897266	20360.61434	296.4509364	18.93484545	54.36227445	39811.41253
4.856124492	165.8452381	8.00499103	1.064946928	36.17748322	2.695573556
0.005258125	4881.725445	0.017888818	85.29141643	15.26807993	11.60583563
1041539.983	1.153413002	0.029807496	0.002876925	99.50464022	0.000303939

89.48930252	300.6529558	856.1172773	11558.44177	413.5091331	366.3331542
0.010897266	20360.61434	296.4509364	18.93484545	54.36227445	39811.41253
863.6250147	0.788985657	0.020389649	7742796.032	499.8602977	0.000207908
1338171.278	0.24839217	0.006419165	13.71075529	4.180106984	6.544240634
0.005258125	4881.725445	0.017888818	85.29141643	15.26807993	11.60583563
0.010897266	20360.61434	296.4509364	18.93484545	54.36227445	39811.41253
23.51452227	1.584496825	793.3575673	10465.1586	1159.149343	158.7182674
34.05858338	3.164024444	59.62864248	1320931.182	0.064740194	0.513731545
0.000766925	99.84334823	0.002609179	213.9597422	139344.0229	1.067310431
863.6250147	0.788985657	0.020389649	7742796.032	499.8602977	0.000207908
0.000766925	99.84334823	0.002609179	213.9597422	139344.0229	1.067310431
0.049330724	6.494228425	2006.898641	13153.45828	638094.5286	0.001711311
1041539.983	1.153413002	0.029807496	0.002876925	99.50464022	0.000303939
0.002287645	3421.127911	161133.5523	16.23189513	2645.570264	771.3547188
105.9732816	6.3929747	0.165212779	122600.7605	0.458528487	3813.563699
0.009476463	1.247545308	418.6383503	3.994100994	237.5002741	20.63147071
0.014948631	1.967938295	321.1088231	1242.03174	6.88952025	3.107924374
526445.3132	0.744328439	45.66244823	82879.57285	18.39041237	4838.439694
626.1466263	3.233256262	0.083556604	402.4668722	701.5811738	0.571536667
0.011987858	1.578162213	0.040784232	852.8692415	0.032291321	0.000415866
0.014948631	1.967938295	321.1088231	1242.03174	6.88952025	3.107924374
0.000597272	0.07862887	0.002031995	0.000852615	0.001608852	10.3649392
0.103811401	10752.00032	930.4253655	1.796525825	92.40164725	4397.009315
0.009476463	1.247545308	418.6383503	3.994100994	237.5002741	20.63147071
77.61239172	0.888483377	266.5892352	2151924.152	6161.391268	16600.1317
626.1466263	3.233256262	0.083556604	402.4668722	701.5811738	0.571536667
21287.98288	55979.56656	1185.365727	746.7222688	6673.504885	13367.94956
77.61239172	0.888483377	266.5892352	2151924.152	6161.391268	16600.1317
0.014948631	1.967938295	321.1088231	1242.03174	6.88952025	3.107924374
626.1466263	3.233256262	0.083556604	402.4668722	701.5811738	0.571536667
150365.2287	45.43559849	0.009823085	1.943123938	122.4287388	25.08318135
0.002788108	0.367045261	0.009485501	1111.987943	0.00751024	12.48542726
0.000597272	0.07862887	0.002031995	0.000852615	0.001608852	10.3649392
77.61239172	0.888483377	266.5892352	2151924.152	6161.391268	16600.1317
19.06650031	0.064457932	868.8052699	0.000160776	0.001318896	9.314874595
0.014948631	1.967938295	321.1088231	1242.03174	6.88952025	3.107924374
0.011987858	1.578162213	0.040784232	852.8692415	0.032291321	0.000415866
77.61239172	0.888483377	266.5892352	2151924.152	6161.391268	16600.1317
0.014948631	1.967938295	321.1088231	1242.03174	6.88952025	3.107924374
77.61239172	0.888483377	266.5892352	2151924.152	6161.391268	16600.1317
0.015405246	109.6570687	274.3303944	369.1194492	989.1050501	353.0399182

6.433193314	0.663480649	203.6340103	205.3135512	3631.52014	960828.0776
105.9732816	6.3929747	0.165212779	122600.7605	0.458528487	3813.563699
0.009476463	1.247545308	418.6383503	3.994100994	237.5002741	20.63147071
526445.3132	0.744328439	45.66244823	82879.57285	18.39041237	4838.439694
1867.779178	17.5010107	0.452276249	4.797533388	16.56754947	5075.746238
0.000597272	0.07862887	0.002031995	0.000852615	0.001608852	10.3649392
0.015405246	109.6570687	274.3303944	369.1194492	989.1050501	353.0399182
72.98093717	19683.22675	182.9091078	157.9680679	294.508805	29.33683247
0.000597272	0.07862887	0.002031995	0.000852615	0.001608852	10.3649392
45.71571223	19434.15265	29351.49885	7091.325138	0.03245681	0.004846509
19.06650031	0.064457932	868.8052699	0.000160776	0.001318896	9.314874595
101392.068	1.611193581	0.041637857	11015.75437	204.3346638	48490.28502
626.1466263	3.233256262	0.083556604	402.4668722	701.5811738	0.571536667
0.014948631	1.967938295	321.1088231	1242.03174	6.88952025	3.107924374
626.1466263	3.233256262	0.083556604	402.4668722	701.5811738	0.571536667
77.61239172	0.888483377	266.5892352	2151924.152	6161.391268	16600.1317
77.61239172	0.888483377	266.5892352	2151924.152	6161.391268	16600.1317
77.61239172	0.888483377	266.5892352	2151924.152	6161.391268	16600.1317
77.61239172	0.888483377	266.5892352	2151924.152	6161.391268	16600.1317
77.61239172	0.888483377	266.5892352	2151924.152	6161.391268	16600.1317
576.8221635	2475.022232	53.96825277	31.81190518	581.1972185	2486.626226
0.000597272	0.07862887	0.002031995	0.000852615	0.001608852	10.3649392
0.014948631	1.967938295	321.1088231	1242.03174	6.88952025	3.107924374
0.009476463	1.247545308	418.6383503	3.994100994	237.5002741	20.63147071
0.014948631	1.967938295	321.1088231	1242.03174	6.88952025	3.107924374
626.1466263	3.233256262	0.083556604	402.4668722	701.5811738	0.571536667
0.000597272	0.07862887	0.002031995	0.000852615	0.001608852	10.3649392
15.22447571	1.554674281	0.040177237	70.10941609	0.031810726	0.000409676
77.61239172	0.888483377	266.5892352	2151924.152	6161.391268	16600.1317
77.61239172	0.888483377	266.5892352	2151924.152	6161.391268	16600.1317
2474713.365	3.853698492	0.099590608	0.009612171	10563.88602	4.911887668
0.008623181	1.135213607	297.4278164	160.0650048	0.023227997	98.50054455
15.22447571	1.554674281	0.040177237	70.10941609	0.031810726	0.000409676
626.1466263	3.233256262	0.083556604	402.4668722	701.5811738	0.571536667
0.012547577	3.348872813	0.042688471	0.004120156	0.033799021	0.000435283
0.103811401	10752.00032	930.4253655	1.796525825	92.40164725	4397.009315
0.002287645	3421.127911	161133.5523	16.23189513	2645.570264	771.3547188
72.98093717	19683.22675	182.9091078	157.9680679	294.508805	29.33683247
526445.3132	0.744328439	45.66244823	82879.57285	18.39041237	4838.439694
626.1466263	3.233256262	0.083556604	402.4668722	701.5811738	0.571536667
72.98093717	19683.22675	182.9091078	157.9680679	294.508805	29.33683247

77.61239172	0.888483377	266.5892352	2151924.152	6161.391268	16600.1317
632.7980203	2.23060177	340.6038937	118.861307	2352.821802	0.007152493
626.1466263	3.233256262	0.083556604	402.4668722	701.5811738	0.571536667
0.103811401	10752.00032	930.4253655	1.796525825	92.40164725	4397.009315
0.014948631	1.967938295	321.1088231	1242.03174	6.88952025	3.107924374
626.1466263	3.233256262	0.083556604	402.4668722	701.5811738	0.571536667
0.009476463	1.247545308	418.6383503	3.994100994	237.5002741	20.63147071
632.7980203	2.23060177	340.6038937	118.861307	2352.821802	0.007152493
626.1466263	3.233256262	0.083556604	402.4668722	701.5811738	0.571536667
0.014948631	1.967938295	321.1088231	1242.03174	6.88952025	3.107924374
77.61239172	0.888483377	266.5892352	2151924.152	6161.391268	16600.1317
0.011987858	1.578162213	0.040784232	852.8692415	0.032291321	0.000415866
626.1466263	3.233256262	0.083556604	402.4668722	701.5811738	0.571536667
0.014948631	1.967938295	321.1088231	1242.03174	6.88952025	3.107924374
626.1466263	3.233256262	0.083556604	402.4668722	701.5811738	0.571536667
0.011987858	1.578162213	0.040784232	852.8692415	0.032291321	0.000415866
626.1466263	3.233256262	0.083556604	402.4668722	701.5811738	0.571536667
39.55229442	774.2950174	0.015376028	312536.0489	124.6097818	636.2423463
15.22447571	1.554674281	0.040177237	70.10941609	0.031810726	0.000409676
19.06650031	0.064457932	868.8052699	0.000160776	0.001318896	9.314874595
0.000536897	0.070680793	48.28889118	25976.69676	194.3603045	0.044758063
576.8221635	2475.022232	53.96825277	31.81190518	581.1972185	2486.626226
0.014948631	1.967938295	321.1088231	1242.03174	6.88952025	3.107924374
0.002788108	0.367045261	0.009485501	1111.987943	0.00751024	12.48542726
526445.3132	0.744328439	45.66244823	82879.57285	18.39041237	4838.439694
0.008623181	1.135213607	297.4278164	160.0650048	0.023227997	98.50054455
632.7980203	2.23060177	340.6038937	118.861307	2352.821802	0.007152493
0.011987858	1.578162213	0.040784232	852.8692415	0.032291321	0.000415866
0.020812295	2.739870489	0.070806102	0.391927783	49.12034862	0.126119339
0.000536897	0.070680793	48.28889118	25976.69676	194.3603045	0.044758063
0.014948631	1.967938295	321.1088231	1242.03174	6.88952025	3.107924374
0.015405246	109.6570687	274.3303944	369.1194492	989.1050501	353.0399182
632.7980203	2.23060177	340.6038937	118.861307	2352.821802	0.007152493
0.014948631	1.967938295	321.1088231	1242.03174	6.88952025	3.107924374
526445.3132	0.744328439	45.66244823	82879.57285	18.39041237	4838.439694
77.61239172	0.888483377	266.5892352	2151924.152	6161.391268	16600.1317
626.1466263	3.233256262	0.083556604	402.4668722	701.5811738	0.571536667
105.9732816	6.3929747	0.165212779	122600.7605	0.458528487	3813.563699
0.014948631	1.967938295	321.1088231	1242.03174	6.88952025	3.107924374
0.014948631	1.967938295	321.1088231	1242.03174	6.88952025	3.107924374
0.011987858	1.578162213	0.040784232	852.8692415	0.032291321	0.000415866

72.98093717	19683.22675	182.9091078	157.9680679	294.508805	29.33683247
576.8221635	2475.022232	53.96825277	31.81190518	581.1972185	2486.626226
66.82931254	34792.47757	2740.636219	173.8610139	2539.861059	10.43059205
0.871323227	18.42367298	0.050341233	0.037150284	0.039858171	0.879961062
4823711.483	3284.337376	642.3911258	8.613380148	0.026315151	67.81256821
19.06650031	0.064457932	868.8052699	0.000160776	0.001318896	9.314874595
632.7980203	2.23060177	340.6038937	118.861307	2352.821802	0.007152493
0.000597272	0.07862887	0.002031995	0.000852615	0.001608852	10.3649392
0.103811401	10752.00032	930.4253655	1.796525825	92.40164725	4397.009315
15.22447571	1.554674281	0.040177237	70.10941609	0.031810726	0.000409676
0.000536897	0.070680793	48.28889118	25976.69676	194.3603045	0.044758063
0.015405246	109.6570687	274.3303944	369.1194492	989.1050501	353.0399182
77.61239172	0.888483377	266.5892352	2151924.152	6161.391268	16600.1317
77.61239172	0.888483377	266.5892352	2151924.152	6161.391268	16600.1317
0.000597272	0.07862887	0.002031995	0.000852615	0.001608852	10.3649392
77.61239172	0.888483377	266.5892352	2151924.152	6161.391268	16600.1317
77.61239172	0.888483377	266.5892352	2151924.152	6161.391268	16600.1317
77.61239172	0.888483377	266.5892352	2151924.152	6161.391268	16600.1317
77.61239172	0.888483377	266.5892352	2151924.152	6161.391268	16600.1317
0.000597272	0.07862887	0.002031995	0.000852615	0.001608852	10.3649392
626.1466263	3.233256262	0.083556604	402.4668722	701.5811738	0.571536667
77.61239172	0.888483377	266.5892352	2151924.152	6161.391268	16600.1317
0.014948631	1.967938295	321.1088231	1242.03174	6.88952025	3.107924374
77.61239172	0.888483377	266.5892352	2151924.152	6161.391268	16600.1317
150365.2287	45.43559849	0.009823085	1.943123938	122.4287388	25.08318135
0.008623181	1.135213607	297.4278164	160.0650048	0.023227997	98.50054455
0.015405246	109.6570687	274.3303944	369.1194492	989.1050501	353.0399182
0.015405246	109.6570687	274.3303944	369.1194492	989.1050501	353.0399182
105.9732816	6.3929747	0.165212779	122600.7605	0.458528487	3813.563699
0.000610059	0.080312331	0.002075501	0.000200321	0.001643298	0.001569903
77.61239172	0.888483377	266.5892352	2151924.152	6161.391268	16600.1317
0.014948631	1.967938295	321.1088231	1242.03174	6.88952025	3.107924374
0.020812295	2.739870489	0.070806102	0.391927783	49.12034862	0.126119339
0.394088162	998.2521398	16.05577584	0.603887652	25.39822402	245337.7573
0.014948631	1.967938295	321.1088231	1242.03174	6.88952025	3.107924374
0.020812295	2.739870489	0.070806102	0.391927783	49.12034862	0.126119339
72.98093717	19683.22675	182.9091078	157.9680679	294.508805	29.33683247
0.009476463	1.247545308	418.6383503	3.994100994	237.5002741	20.63147071
0.014948631	1.967938295	321.1088231	1242.03174	6.88952025	3.107924374
0.014948631	1.967938295	321.1088231	1242.03174	6.88952025	3.107924374
77.61239172	0.888483377	266.5892352	2151924.152	6161.391268	16600.1317

0.020812295	2.739870489	0.070806102	0.391927783	49.12034862	0.126119339
0.000597272	0.07862887	0.002031995	0.000852615	0.001608852	10.3649392
77.61239172	0.888483377	266.5892352	2151924.152	6161.391268	16600.1317
0.008623181	1.135213607	297.4278164	160.0650048	0.023227997	98.50054455
0.014948631	1.967938295	321.1088231	1242.03174	6.88952025	3.107924374
6.433193314	0.663480649	203.6340103	205.3135512	3631.52014	960828.0776
526445.3132	0.744328439	45.66244823	82879.57285	18.39041237	4838.439694
6.433193314	0.663480649	203.6340103	205.3135512	3631.52014	960828.0776
0.103811401	10752.00032	930.4253655	1.796525825	92.40164725	4397.009315
0.000536897	0.070680793	48.28889118	25976.69676	194.3603045	0.044758063
0.011987858	1.578162213	0.040784232	852.8692415	0.032291321	0.000415866
626.1466263	3.233256262	0.083556604	402.4668722	701.5811738	0.571536667
0.014948631	1.967938295	321.1088231	1242.03174	6.88952025	3.107924374
526445.3132	0.744328439	45.66244823	82879.57285	18.39041237	4838.439694
77.61239172	0.888483377	266.5892352	2151924.152	6161.391268	16600.1317
0.011987858	1.578162213	0.040784232	852.8692415	0.032291321	0.000415866
576.8221635	2475.022232	53.96825277	31.81190518	581.1972185	2486.626226
101392.068	1.611193581	0.041637857	11015.75437	204.3346638	48490.28502
105.9732816	6.3929747	0.165212779	122600.7605	0.458528487	3813.563699
0.020812295	2.739870489	0.070806102	0.391927783	49.12034862	0.126119339
0.008623181	1.135213607	297.4278164	160.0650048	0.023227997	98.50054455
626.1466263	3.233256262	0.083556604	402.4668722	701.5811738	0.571536667
72.98093717	19683.22675	182.9091078	157.9680679	294.508805	29.33683247
0.015405246	109.6570687	274.3303944	369.1194492	989.1050501	353.0399182
19.06650031	0.064457932	868.8052699	0.000160776	0.001318896	9.314874595
626.1466263	3.233256262	0.083556604	402.4668722	701.5811738	0.571536667
77.61239172	0.888483377	266.5892352	2151924.152	6161.391268	16600.1317
77.61239172	0.888483377	266.5892352	2151924.152	6161.391268	16600.1317
526445.3132	0.744328439	45.66244823	82879.57285	18.39041237	4838.439694
77.61239172	0.888483377	266.5892352	2151924.152	6161.391268	16600.1317
0.011987858	1.578162213	0.040784232	852.8692415	0.032291321	0.000415866
77.61239172	0.888483377	266.5892352	2151924.152	6161.391268	16600.1317
77.61239172	0.888483377	266.5892352	2151924.152	6161.391268	16600.1317
626.1466263	3.233256262	0.083556604	402.4668722	701.5811738	0.571536667
626.1466263	3.233256262	0.083556604	402.4668722	701.5811738	0.571536667
0.014948631	1.967938295	321.1088231	1242.03174	6.88952025	3.107924374
0.000597272	0.07862887	0.002031995	0.000852615	0.001608852	10.3649392
626.1466263	3.233256262	0.083556604	402.4668722	701.5811738	0.571536667
19.06650031	0.064457932	868.8052699	0.000160776	0.001318896	9.314874595
77.61239172	0.888483377	266.5892352	2151924.152	6161.391268	16600.1317
0.014948631	1.967938295	321.1088231	1242.03174	6.88952025	3.107924374

0.011987858	1.578162213	0.040784232	852.8692415	0.032291321	0.000415866
0.020812295	2.739870489	0.070806102	0.391927783	49.12034862	0.126119339
0.000597272	0.07862887	0.002031995	0.000852615	0.001608852	10.3649392
0.002788108	0.367045261	0.009485501	1111.987943	0.00751024	12.48542726
0.015405246	109.6570687	274.3303944	369.1194492	989.1050501	353.0399182
6.433193314	0.663480649	203.6340103	205.3135512	3631.52014	960828.0776
526445.3132	0.744328439	45.66244823	82879.57285	18.39041237	4838.439694
0.008623181	1.135213607	297.4278164	160.0650048	0.023227997	98.50054455
0.000536897	0.070680793	48.28889118	25976.69676	194.3603045	0.044758063
0.014948631	1.967938295	321.1088231	1242.03174	6.88952025	3.107924374
526445.3132	0.744328439	45.66244823	82879.57285	18.39041237	4838.439694
0.000536897	0.070680793	48.28889118	25976.69676	194.3603045	0.044758063
0.008623181	1.135213607	297.4278164	160.0650048	0.023227997	98.50054455
72.98093717	19683.22675	182.9091078	157.9680679	294.508805	29.33683247
632.7980203	2.23060177	340.6038937	118.861307	2352.821802	0.007152493
0.014948631	1.967938295	321.1088231	1242.03174	6.88952025	3.107924374
19.06650031	0.064457932	868.8052699	0.000160776	0.001318896	9.314874595
576.8221635	2475.022232	53.96825277	31.81190518	581.1972185	2486.626226
0.015405246	109.6570687	274.3303944	369.1194492	989.1050501	353.0399182
77.61239172	0.888483377	266.5892352	2151924.152	6161.391268	16600.1317
526445.3132	0.744328439	45.66244823	82879.57285	18.39041237	4838.439694
77.61239172	0.888483377	266.5892352	2151924.152	6161.391268	16600.1317
0.014948631	1.967938295	321.1088231	1242.03174	6.88952025	3.107924374
19.06650031	0.064457932	868.8052699	0.000160776	0.001318896	9.314874595
77.61239172	0.888483377	266.5892352	2151924.152	6161.391268	16600.1317
626.1466263	3.233256262	0.083556604	402.4668722	701.5811738	0.571536667
0.000536897	0.070680793	48.28889118	25976.69676	194.3603045	0.044758063
0.000536897	0.070680793	48.28889118	25976.69676	194.3603045	0.044758063
21287.98288	55979.56656	1185.365727	746.7222688	6673.504885	13367.94956
0.014948631	1.967938295	321.1088231	1242.03174	6.88952025	3.107924374
19.06650031	0.064457932	868.8052699	0.000160776	0.001318896	9.314874595
77.61239172	0.888483377	266.5892352	2151924.152	6161.391268	16600.1317
35.43013637	2156.189912	0.039241621	23.75132877	694.0906976	8.569733235
77.61239172	0.888483377	266.5892352	2151924.152	6161.391268	16600.1317
0.000536897	0.070680793	48.28889118	25976.69676	194.3603045	0.044758063
0.008623181	1.135213607	297.4278164	160.0650048	0.023227997	98.50054455
0.011987858	1.578162213	0.040784232	852.8692415	0.032291321	0.000415866
632.7980203	2.23060177	340.6038937	118.861307	2352.821802	0.007152493
0.014948631	1.967938295	321.1088231	1242.03174	6.88952025	3.107924374
77.61239172	0.888483377	266.5892352	2151924.152	6161.391268	16600.1317
626.1466263	3.233256262	0.083556604	402.4668722	701.5811738	0.571536667

626.1466263	3.233256262	0.083556604	402.4668722	701.5811738	0.571536667
0.014948631	1.967938295	321.1088231	1242.03174	6.88952025	3.107924374
77.61239172	0.888483377	266.5892352	2151924.152	6161.391268	16600.1317
35.43013637	2156.189912	0.039241621	23.75132877	694.0906976	8.569733235
77.61239172	0.888483377	266.5892352	2151924.152	6161.391268	16600.1317
77.61239172	0.888483377	266.5892352	2151924.152	6161.391268	16600.1317
626.1466263	3.233256262	0.083556604	402.4668722	701.5811738	0.571536667
0.011987858	1.578162213	0.040784232	852.8692415	0.032291321	0.000415866
0.014948631	1.967938295	321.1088231	1242.03174	6.88952025	3.107924374
0.008623181	1.135213607	297.4278164	160.0650048	0.023227997	98.50054455
0.000597272	0.07862887	0.002031995	0.000852615	0.001608852	10.3649392
0.871323227	18.42367298	0.050341233	0.037150284	0.039858171	0.879961062
77.61239172	0.888483377	266.5892352	2151924.152	6161.391268	16600.1317
626.1466263	3.233256262	0.083556604	402.4668722	701.5811738	0.571536667
0.000597272	0.07862887	0.002031995	0.000852615	0.001608852	10.3649392
41332.26009	3.266935407	10146.53011	0.008148624	6281017.939	0.000860879
0.000536897	0.070680793	48.28889118	25976.69676	194.3603045	0.044758063
1867.779178	17.5010107	0.452276249	4.797533388	16.56754947	5075.746238
0.011987858	1.578162213	0.040784232	852.8692415	0.032291321	0.000415866
0.000536897	0.070680793	48.28889118	25976.69676	194.3603045	0.044758063
0.020812295	2.739870489	0.070806102	0.391927783	49.12034862	0.126119339
0.002287645	3421.127911	161133.5523	16.23189513	2645.570264	771.3547188
2474713.365	3.853698492	0.099590608	0.009612171	10563.88602	4.911887668
72.98093717	19683.22675	182.9091078	157.9680679	294.508805	29.33683247
0.014948631	1.967938295	321.1088231	1242.03174	6.88952025	3.107924374
576.8221635	2475.022232	53.96825277	31.81190518	581.1972185	2486.626226
0.020812295	2.739870489	0.070806102	0.391927783	49.12034862	0.126119339
0.015405246	109.6570687	274.3303944	369.1194492	989.1050501	353.0399182
101392.068	1.611193581	0.041637857	11015.75437	204.3346638	48490.28502
35.43013637	2156.189912	0.039241621	23.75132877	694.0906976	8.569733235
0.008623181	1.135213607	297.4278164	160.0650048	0.023227997	98.50054455
0.015405246	109.6570687	274.3303944	369.1194492	989.1050501	353.0399182
0.014948631	1.967938295	321.1088231	1242.03174	6.88952025	3.107924374
2474713.365	3.853698492	0.099590608	0.009612171	10563.88602	4.911887668
21287.98288	55979.56656	1185.365727	746.7222688	6673.504885	13367.94956
0.014948631	1.967938295	321.1088231	1242.03174	6.88952025	3.107924374
632.7980203	2.23060177	340.6038937	118.861307	2352.821802	0.007152493
0.000536897	0.070680793	48.28889118	25976.69676	194.3603045	0.044758063
0.014948631	1.967938295	321.1088231	1242.03174	6.88952025	3.107924374
0.014948631	1.967938295	321.1088231	1242.03174	6.88952025	3.107924374
41332.26009	3.266935407	10146.53011	0.008148624	6281017.939	0.000860879

77.61239172	0.888483377	266.5892352	2151924.152	6161.391268	16600.1317
4.07342159	0.087421402	11.31481594	0.000218053	0.001788759	0.039418547
0.014948631	1.967938295	321.1088231	1242.03174	6.88952025	3.107924374
626.1466263	3.233256262	0.083556604	402.4668722	701.5811738	0.571536667
0.008623181	1.135213607	297.4278164	160.0650048	0.023227997	98.50054455
526445.3132	0.744328439	45.66244823	82879.57285	18.39041237	4838.439694
0.000536897	0.070680793	48.28889118	25976.69676	194.3603045	0.044758063
0.014948631	1.967938295	321.1088231	1242.03174	6.88952025	3.107924374
626.1466263	3.233256262	0.083556604	402.4668722	701.5811738	0.571536667
19.06650031	0.064457932	868.8052699	0.000160776	0.001318896	9.314874595
0.014948631	1.967938295	321.1088231	1242.03174	6.88952025	3.107924374
77.61239172	0.888483377	266.5892352	2151924.152	6161.391268	16600.1317
0.394088162	998.2521398	16.05577584	0.603887652	25.39822402	245337.7573
626.1466263	3.233256262	0.083556604	402.4668722	701.5811738	0.571536667
0.014948631	1.967938295	321.1088231	1242.03174	6.88952025	3.107924374
0.014948631	1.967938295	321.1088231	1242.03174	6.88952025	3.107924374
19.06650031	0.064457932	868.8052699	0.000160776	0.001318896	9.314874595
626.1466263	3.233256262	0.083556604	402.4668722	701.5811738	0.571536667
1867.779178	17.5010107	0.452276249	4.797533388	16.56754947	5075.746238
0.002287645	3421.127911	161133.5523	16.23189513	2645.570264	771.3547188
2474713.365	3.853698492	0.099590608	0.009612171	10563.88602	4.911887668
0.014948631	1.967938295	321.1088231	1242.03174	6.88952025	3.107924374
0.014948631	1.967938295	321.1088231	1242.03174	6.88952025	3.107924374
0.011987858	1.578162213	0.040784232	852.8692415	0.032291321	0.000415866
0.020812295	2.739870489	0.070806102	0.391927783	49.12034862	0.126119339
0.000610059	0.080312331	0.002075501	0.000200321	0.001643298	0.001569903
0.000597272	0.07862887	0.002031995	0.000852615	0.001608852	10.3649392
15.22447571	1.554674281	0.040177237	70.10941609	0.031810726	0.000409676
626.1466263	3.233256262	0.083556604	402.4668722	701.5811738	0.571536667
0.000536897	0.070680793	48.28889118	25976.69676	194.3603045	0.044758063
0.015405246	109.6570687	274.3303944	369.1194492	989.1050501	353.0399182
77.61239172	0.888483377	266.5892352	2151924.152	6161.391268	16600.1317
6.433193314	0.663480649	203.6340103	205.3135512	3631.52014	960828.0776
0.014948631	1.967938295	321.1088231	1242.03174	6.88952025	3.107924374
626.1466263	3.233256262	0.083556604	402.4668722	701.5811738	0.571536667
77.61239172	0.888483377	266.5892352	2151924.152	6161.391268	16600.1317
0.000597272	0.07862887	0.002031995	0.000852615	0.001608852	10.3649392
953.7982127	84.08315452	17.62468986	8240.827346	0.078049331	0.004693407
0.014948631	1.967938295	321.1088231	1242.03174	6.88952025	3.107924374
0.014948631	1.967938295	321.1088231	1242.03174	6.88952025	3.107924374
0.014948631	1.967938295	321.1088231	1242.03174	6.88952025	3.107924374

0.008623181	1.135213607	297.4278164	160.0650048	0.023227997	98.50054455
0.103811401	10752.00032	930.4253655	1.796525825	92.40164725	4397.009315
77.61239172	0.888483377	266.5892352	2151924.152	6161.391268	16600.1317
0.014948631	1.967938295	321.1088231	1242.03174	6.88952025	3.107924374
21287.98288	55979.56656	1185.365727	746.7222688	6673.504885	13367.94956
77.61239172	0.888483377	266.5892352	2151924.152	6161.391268	16600.1317
0.000597272	0.07862887	0.002031995	0.000852615	0.001608852	10.3649392
0.015405246	109.6570687	274.3303944	369.1194492	989.1050501	353.0399182
6.433193314	0.663480649	203.6340103	205.3135512	3631.52014	960828.0776
66.82931254	34792.47757	2740.636219	173.8610139	2539.861059	10.43059205
0.103811401	10752.00032	930.4253655	1.796525825	92.40164725	4397.009315
0.103811401	10752.00032	930.4253655	1.796525825	92.40164725	4397.009315
0.000597272	0.07862887	0.002031995	0.000852615	0.001608852	10.3649392
0.008623181	1.135213607	297.4278164	160.0650048	0.023227997	98.50054455
0.014948631	1.967938295	321.1088231	1242.03174	6.88952025	3.107924374
626.1466263	3.233256262	0.083556604	402.4668722	701.5811738	0.571536667
0.014948631	1.967938295	321.1088231	1242.03174	6.88952025	3.107924374
626.1466263	3.233256262	0.083556604	402.4668722	701.5811738	0.571536667
77.61239172	0.888483377	266.5892352	2151924.152	6161.391268	16600.1317
526445.3132	0.744328439	45.66244823	82879.57285	18.39041237	4838.439694
1867.779178	17.5010107	0.452276249	4.797533388	16.56754947	5075.746238
9975251.794	0.701985511	646668.04	931498.876	152527.9596	2937822.355
626.1466263	3.233256262	0.083556604	402.4668722	701.5811738	0.571536667
626.1466263	3.233256262	0.083556604	402.4668722	701.5811738	0.571536667
0.103811401	10752.00032	930.4253655	1.796525825	92.40164725	4397.009315
576.8221635	2475.022232	53.96825277	31.81190518	581.1972185	2486.626226
626.1466263	3.233256262	0.083556604	402.4668722	701.5811738	0.571536667
626.1466263	3.233256262	0.083556604	402.4668722	701.5811738	0.571536667
0.011987858	1.578162213	0.040784232	852.8692415	0.032291321	0.000415866
0.009476463	1.247545308	418.6383503	3.994100994	237.5002741	20.63147071
0.011987858	1.578162213	0.040784232	852.8692415	0.032291321	0.000415866
0.011987858	1.578162213	0.040784232	852.8692415	0.032291321	0.000415866
77.61239172	0.888483377	266.5892352	2151924.152	6161.391268	16600.1317
0.020812295	2.739870489	0.070806102	0.391927783	49.12034862	0.126119339
0.000597272	0.07862887	0.002031995	0.000852615	0.001608852	10.3649392
626.1466263	3.233256262	0.083556604	402.4668722	701.5811738	0.571536667
2474713.365	3.853698492	0.099590608	0.009612171	10563.88602	4.911887668
21287.98288	55979.56656	1185.365727	746.7222688	6673.504885	13367.94956
0.015405246	109.6570687	274.3303944	369.1194492	989.1050501	353.0399182
626.1466263	3.233256262	0.083556604	402.4668722	701.5811738	0.571536667
0.014948631	1.967938295	321.1088231	1242.03174	6.88952025	3.107924374

0.103811401	10752.00032	930.4253655	1.796525825	92.40164725	4397.009315
626.1466263	3.233256262	0.083556604	402.4668722	701.5811738	0.571536667
0.020812295	2.739870489	0.070806102	0.391927783	49.12034862	0.126119339
953.7982127	84.08315452	17.62468986	8240.827346	0.078049331	0.004693407
77.61239172	0.888483377	266.5892352	2151924.152	6161.391268	16600.1317
0.008623181	1.135213607	297.4278164	160.0650048	0.023227997	98.50054455
0.103811401	10752.00032	930.4253655	1.796525825	92.40164725	4397.009315
21287.98288	55979.56656	1185.365727	746.7222688	6673.504885	13367.94956
576.8221635	2475.022232	53.96825277	31.81190518	581.1972185	2486.626226
576.8221635	2475.022232	53.96825277	31.81190518	581.1972185	2486.626226
526445.3132	0.744328439	45.66244823	82879.57285	18.39041237	4838.439694
0.011987858	1.578162213	0.040784232	852.8692415	0.032291321	0.000415866
77.61239172	0.888483377	266.5892352	2151924.152	6161.391268	16600.1317
0.009476463	1.247545308	418.6383503	3.994100994	237.5002741	20.63147071
0.000597272	0.07862887	0.002031995	0.000852615	0.001608852	10.3649392
632.7980203	2.23060177	340.6038937	118.861307	2352.821802	0.007152493
0.000597272	0.07862887	0.002031995	0.000852615	0.001608852	10.3649392
0.015405246	109.6570687	274.3303944	369.1194492	989.1050501	353.0399182
0.014948631	1.967938295	321.1088231	1242.03174	6.88952025	3.107924374
21287.98288	55979.56656	1185.365727	746.7222688	6673.504885	13367.94956
72.98093717	19683.22675	182.9091078	157.9680679	294.508805	29.33683247
77.61239172	0.888483377	266.5892352	2151924.152	6161.391268	16600.1317
21287.98288	55979.56656	1185.365727	746.7222688	6673.504885	13367.94956
0.002287645	3421.127911	161133.5523	16.23189513	2645.570264	771.3547188
0.014948631	1.967938295	321.1088231	1242.03174	6.88952025	3.107924374
0.008623181	1.135213607	297.4278164	160.0650048	0.023227997	98.50054455
19.06650031	0.064457932	868.8052699	0.000160776	0.001318896	9.314874595
626.1466263	3.233256262	0.083556604	402.4668722	701.5811738	0.571536667
0.015405246	109.6570687	274.3303944	369.1194492	989.1050501	353.0399182
105.9732816	6.3929747	0.165212779	122600.7605	0.458528487	3813.563699
0.011987858	1.578162213	0.040784232	852.8692415	0.032291321	0.000415866
0.000536897	0.070680793	48.28889118	25976.69676	194.3603045	0.044758063
632.7980203	2.23060177	340.6038937	118.861307	2352.821802	0.007152493
77.61239172	0.888483377	266.5892352	2151924.152	6161.391268	16600.1317
0.871323227	18.42367298	0.050341233	0.037150284	0.039858171	0.879961062
0.015405246	109.6570687	274.3303944	369.1194492	989.1050501	353.0399182
626.1466263	3.233256262	0.083556604	402.4668722	701.5811738	0.571536667
0.014948631	1.967938295	321.1088231	1242.03174	6.88952025	3.107924374
0.015405246	109.6570687	274.3303944	369.1194492	989.1050501	353.0399182
0.014948631	1.967938295	321.1088231	1242.03174	6.88952025	3.107924374
526445.3132	0.744328439	45.66244823	82879.57285	18.39041237	4838.439694

1867.779178	17.5010107	0.452276249	4.797533388	16.56754947	5075.746238
0.000597272	0.07862887	0.002031995	0.000852615	0.001608852	10.3649392
0.009476463	1.247545308	418.6383503	3.994100994	237.5002741	20.63147071
0.015405246	109.6570687	274.3303944	369.1194492	989.1050501	353.0399182
0.008623181	1.135213607	297.4278164	160.0650048	0.023227997	98.50054455
0.103811401	10752.00032	930.4253655	1.796525825	92.40164725	4397.009315
0.014948631	1.967938295	321.1088231	1242.03174	6.88952025	3.107924374
77.61239172	0.888483377	266.5892352	2151924.152	6161.391268	16600.1317
77.61239172	0.888483377	266.5892352	2151924.152	6161.391268	16600.1317
4.07342159	0.087421402	11.31481594	0.000218053	0.001788759	0.039418547
101392.068	1.611193581	0.041637857	11015.75437	204.3346638	48490.28502
0.015405246	109.6570687	274.3303944	369.1194492	989.1050501	353.0399182
0.014948631	1.967938295	321.1088231	1242.03174	6.88952025	3.107924374
626.1466263	3.233256262	0.083556604	402.4668722	701.5811738	0.571536667
77.61239172	0.888483377	266.5892352	2151924.152	6161.391268	16600.1317
0.020812295	2.739870489	0.070806102	0.391927783	49.12034862	0.126119339
0.008623181	1.135213607	297.4278164	160.0650048	0.023227997	98.50054455
77.61239172	0.888483377	266.5892352	2151924.152	6161.391268	16600.1317
4.07342159	0.087421402	11.31481594	0.000218053	0.001788759	0.039418547
0.000536897	0.070680793	48.28889118	25976.69676	194.3603045	0.044758063
77.61239172	0.888483377	266.5892352	2151924.152	6161.391268	16600.1317
0.000597272	0.07862887	0.002031995	0.000852615	0.001608852	10.3649392
0.015405246	109.6570687	274.3303944	369.1194492	989.1050501	353.0399182
0.015405246	109.6570687	274.3303944	369.1194492	989.1050501	353.0399182
0.871323227	18.42367298	0.050341233	0.037150284	0.039858171	0.879961062
0.009476463	1.247545308	418.6383503	3.994100994	237.5002741	20.63147071
0.009476463	1.247545308	418.6383503	3.994100994	237.5002741	20.63147071
0.015405246	109.6570687	274.3303944	369.1194492	989.1050501	353.0399182
0.002788108	0.367045261	0.009485501	1111.987943	0.00751024	12.48542726
526445.3132	0.744328439	45.66244823	82879.57285	18.39041237	4838.439694
0.014948631	1.967938295	321.1088231	1242.03174	6.88952025	3.107924374
21287.98288	55979.56656	1185.365727	746.7222688	6673.504885	13367.94956
0.000610059	0.080312331	0.002075501	0.000200321	0.001643298	0.001569903
35.43013637	2156.189912	0.039241621	23.75132877	694.0906976	8.569733235
4.07342159	0.087421402	11.31481594	0.000218053	0.001788759	0.039418547
0.000610059	0.080312331	0.002075501	0.000200321	0.001643298	0.001569903
77.61239172	0.888483377	266.5892352	2151924.152	6161.391268	16600.1317
15.22447571	1.554674281	0.040177237	70.10941609	0.031810726	0.000409676
0.014948631	1.967938295	321.1088231	1242.03174	6.88952025	3.107924374
15.22447571	1.554674281	0.040177237	70.10941609	0.031810726	0.000409676
0.014948631	1.967938295	321.1088231	1242.03174	6.88952025	3.107924374

77.61239172	0.888483377	266.5892352	2151924.152	6161.391268	16600.1317
77.61239172	0.888483377	266.5892352	2151924.152	6161.391268	16600.1317
0.014948631	1.967938295	321.1088231	1242.03174	6.88952025	3.107924374
77.61239172	0.888483377	266.5892352	2151924.152	6161.391268	16600.1317
632.7980203	2.23060177	340.6038937	118.861307	2352.821802	0.007152493
0.014948631	1.967938295	321.1088231	1242.03174	6.88952025	3.107924374
0.014948631	1.967938295	321.1088231	1242.03174	6.88952025	3.107924374
9975251.794	0.701985511	646668.04	931498.876	152527.9596	2937822.355
66.82931254	34792.47757	2740.636219	173.8610139	2539.861059	10.43059205
0.394088162	998.2521398	16.05577584	0.603887652	25.39822402	245337.7573
101392.068	1.611193581	0.041637857	11015.75437	204.3346638	48490.28502
0.000536897	0.070680793	48.28889118	25976.69676	194.3603045	0.044758063
45.71571223	19434.15265	29351.49885	7091.325138	0.03245681	0.004846509
72.98093717	19683.22675	182.9091078	157.9680679	294.508805	29.33683247
0.002788108	0.367045261	0.009485501	1111.987943	0.00751024	12.48542726
0.015405246	109.6570687	274.3303944	369.1194492	989.1050501	353.0399182
0.000597272	0.07862887	0.002031995	0.000852615	0.001608852	10.3649392
77.61239172	0.888483377	266.5892352	2151924.152	6161.391268	16600.1317
21287.98288	55979.56656	1185.365727	746.7222688	6673.504885	13367.94956
576.8221635	2475.022232	53.96825277	31.81190518	581.1972185	2486.626226
77.61239172	0.888483377	266.5892352	2151924.152	6161.391268	16600.1317
632.7980203	2.23060177	340.6038937	118.861307	2352.821802	0.007152493
0.011987858	1.578162213	0.040784232	852.8692415	0.032291321	0.000415866
77.61239172	0.888483377	266.5892352	2151924.152	6161.391268	16600.1317
0.014948631	1.967938295	321.1088231	1242.03174	6.88952025	3.107924374
576.8221635	2475.022232	53.96825277	31.81190518	581.1972185	2486.626226
626.1466263	3.233256262	0.083556604	402.4668722	701.5811738	0.571536667
0.002788108	0.367045261	0.009485501	1111.987943	0.00751024	12.48542726
45.71571223	19434.15265	29351.49885	7091.325138	0.03245681	0.004846509
21287.98288	55979.56656	1185.365727	746.7222688	6673.504885	13367.94956
0.015405246	109.6570687	274.3303944	369.1194492	989.1050501	353.0399182
0.011987858	1.578162213	0.040784232	852.8692415	0.032291321	0.000415866
0.008623181	1.135213607	297.4278164	160.0650048	0.023227997	98.50054455
632.7980203	2.23060177	340.6038937	118.861307	2352.821802	0.007152493
0.000597272	0.07862887	0.002031995	0.000852615	0.001608852	10.3649392
77.61239172	0.888483377	266.5892352	2151924.152	6161.391268	16600.1317
0.015405246	109.6570687	274.3303944	369.1194492	989.1050501	353.0399182
0.000536897	0.070680793	48.28889118	25976.69676	194.3603045	0.044758063
0.011987858	1.578162213	0.040784232	852.8692415	0.032291321	0.000415866
626.1466263	3.233256262	0.083556604	402.4668722	701.5811738	0.571536667
526445.3132	0.744328439	45.66244823	82879.57285	18.39041237	4838.439694

0.020812295	2.739870489	0.070806102	0.391927783	49.12034862	0.126119339
0.014948631	1.967938295	321.1088231	1242.03174	6.88952025	3.107924374
0.014948631	1.967938295	321.1088231	1242.03174	6.88952025	3.107924374
0.009476463	1.247545308	418.6383503	3.994100994	237.5002741	20.63147071
626.1466263	3.233256262	0.083556604	402.4668722	701.5811738	0.571536667
0.871323227	18.42367298	0.050341233	0.037150284	0.039858171	0.879961062
77.61239172	0.888483377	266.5892352	2151924.152	6161.391268	16600.1317
77.61239172	0.888483377	266.5892352	2151924.152	6161.391268	16600.1317
2474713.365	3.853698492	0.099590608	0.009612171	10563.88602	4.911887668
0.014948631	1.967938295	321.1088231	1242.03174	6.88952025	3.107924374
77.61239172	0.888483377	266.5892352	2151924.152	6161.391268	16600.1317
0.015405246	109.6570687	274.3303944	369.1194492	989.1050501	353.0399182
21287.98288	55979.56656	1185.365727	746.7222688	6673.504885	13367.94956
0.011987858	1.578162213	0.040784232	852.8692415	0.032291321	0.000415866
77.61239172	0.888483377	266.5892352	2151924.152	6161.391268	16600.1317
626.1466263	3.233256262	0.083556604	402.4668722	701.5811738	0.571536667
77.61239172	0.888483377	266.5892352	2151924.152	6161.391268	16600.1317
0.011987858	1.578162213	0.040784232	852.8692415	0.032291321	0.000415866
15.22447571	1.554674281	0.040177237	70.10941609	0.031810726	0.000409676
0.015405246	109.6570687	274.3303944	369.1194492	989.1050501	353.0399182
21287.98288	55979.56656	1185.365727	746.7222688	6673.504885	13367.94956
9975251.794	0.701985511	646668.04	931498.876	152527.9596	2937822.355
0.000597272	0.07862887	0.002031995	0.000852615	0.001608852	10.3649392
626.1466263	3.233256262	0.083556604	402.4668722	701.5811738	0.571536667
0.011987858	1.578162213	0.040784232	852.8692415	0.032291321	0.000415866
39.55229442	774.2950174	0.015376028	312536.0489	124.6097818	636.2423463
576.8221635	2475.022232	53.96825277	31.81190518	581.1972185	2486.626226
77.61239172	0.888483377	266.5892352	2151924.152	6161.391268	16600.1317
626.1466263	3.233256262	0.083556604	402.4668722	701.5811738	0.571536667
0.103811401	10752.00032	930.4253655	1.796525825	92.40164725	4397.009315
0.014948631	1.967938295	321.1088231	1242.03174	6.88952025	3.107924374
77.61239172	0.888483377	266.5892352	2151924.152	6161.391268	16600.1317
66.82931254	34792.47757	2740.636219	173.8610139	2539.861059	10.43059205
77.61239172	0.888483377	266.5892352	2151924.152	6161.391268	16600.1317
0.008623181	1.135213607	297.4278164	160.0650048	0.023227997	98.50054455
0.011987858	1.578162213	0.040784232	852.8692415	0.032291321	0.000415866
0.394088162	998.2521398	16.05577584	0.603887652	25.39822402	245337.7573
526445.3132	0.744328439	45.66244823	82879.57285	18.39041237	4838.439694
0.009476463	1.247545308	418.6383503	3.994100994	237.5002741	20.63147071
526445.3132	0.744328439	45.66244823	82879.57285	18.39041237	4838.439694
19.06650031	0.064457932	868.8052699	0.000160776	0.001318896	9.314874595

626.1466263	3.233256262	0.083556604	402.4668722	701.5811738	0.571536667
0.011987858	1.578162213	0.040784232	852.8692415	0.032291321	0.000415866
526445.3132	0.744328439	45.66244823	82879.57285	18.39041237	4838.439694
0.103811401	10752.00032	930.4253655	1.796525825	92.40164725	4397.009315
626.1466263	3.233256262	0.083556604	402.4668722	701.5811738	0.571536667
77.61239172	0.888483377	266.5892352	2151924.152	6161.391268	16600.1317
72.98093717	19683.22675	182.9091078	157.9680679	294.508805	29.33683247
0.008623181	1.135213607	297.4278164	160.0650048	0.023227997	98.50054455
0.103811401	10752.00032	930.4253655	1.796525825	92.40164725	4397.009315
0.014948631	1.967938295	321.1088231	1242.03174	6.88952025	3.107924374
0.008623181	1.135213607	297.4278164	160.0650048	0.023227997	98.50054455
0.008623181	1.135213607	297.4278164	160.0650048	0.023227997	98.50054455
0.015405246	109.6570687	274.3303944	369.1194492	989.1050501	353.0399182
0.010677332	10306.90517	1001.58427	117.5306001	241.659629	0.000370403
77.61239172	0.888483377	266.5892352	2151924.152	6161.391268	16600.1317
2474713.365	3.853698492	0.099590608	0.009612171	10563.88602	4.911887668
77.61239172	0.888483377	266.5892352	2151924.152	6161.391268	16600.1317
77.61239172	0.888483377	266.5892352	2151924.152	6161.391268	16600.1317
0.014948631	1.967938295	321.1088231	1242.03174	6.88952025	3.107924374
77.61239172	0.888483377	266.5892352	2151924.152	6161.391268	16600.1317
1867.779178	17.5010107	0.452276249	4.797533388	16.56754947	5075.746238
72.98093717	19683.22675	182.9091078	157.9680679	294.508805	29.33683247
0.014948631	1.967938295	321.1088231	1242.03174	6.88952025	3.107924374
21287.98288	55979.56656	1185.365727	746.7222688	6673.504885	13367.94956
0.002287645	3421.127911	161133.5523	16.23189513	2645.570264	771.3547188
626.1466263	3.233256262	0.083556604	402.4668722	701.5811738	0.571536667
0.000597272	0.07862887	0.002031995	0.000852615	0.001608852	10.3649392
77.61239172	0.888483377	266.5892352	2151924.152	6161.391268	16600.1317
0.014948631	1.967938295	321.1088231	1242.03174	6.88952025	3.107924374
0.103811401	10752.00032	930.4253655	1.796525825	92.40164725	4397.009315
0.014948631	1.967938295	321.1088231	1242.03174	6.88952025	3.107924374
0.010677332	10306.90517	1001.58427	117.5306001	241.659629	0.000370403
41332.26009	3.266935407	10146.53011	0.008148624	6281017.939	0.000860879
77.61239172	0.888483377	266.5892352	2151924.152	6161.391268	16600.1317
626.1466263	3.233256262	0.083556604	402.4668722	701.5811738	0.571536667
626.1466263	3.233256262	0.083556604	402.4668722	701.5811738	0.571536667
0.000597272	0.07862887	0.002031995	0.000852615	0.001608852	10.3649392
21287.98288	55979.56656	1185.365727	746.7222688	6673.504885	13367.94956
77.61239172	0.888483377	266.5892352	2151924.152	6161.391268	16600.1317
2474713.365	3.853698492	0.099590608	0.009612171	10563.88602	4.911887668
0.014948631	1.967938295	321.1088231	1242.03174	6.88952025	3.107924374

626.1466263	3.233256262	0.083556604	402.4668722	701.5811738	0.571536667
0.011987858	1.578162213	0.040784232	852.8692415	0.032291321	0.000415866
0.009476463	1.247545308	418.6383503	3.994100994	237.5002741	20.63147071
0.008623181	1.135213607	297.4278164	160.0650048	0.023227997	98.50054455
626.1466263	3.233256262	0.083556604	402.4668722	701.5811738	0.571536667
0.000597272	0.07862887	0.002031995	0.000852615	0.001608852	10.3649392
2474713.365	3.853698492	0.099590608	0.009612171	10563.88602	4.911887668
77.61239172	0.888483377	266.5892352	2151924.152	6161.391268	16600.1317
0.103811401	10752.00032	930.4253655	1.796525825	92.40164725	4397.009315
626.1466263	3.233256262	0.083556604	402.4668722	701.5811738	0.571536667
77.61239172	0.888483377	266.5892352	2151924.152	6161.391268	16600.1317
77.61239172	0.888483377	266.5892352	2151924.152	6161.391268	16600.1317
0.014948631	1.967938295	321.1088231	1242.03174	6.88952025	3.107924374
626.1466263	3.233256262	0.083556604	402.4668722	701.5811738	0.571536667
0.011987858	1.578162213	0.040784232	852.8692415	0.032291321	0.000415866
77.61239172	0.888483377	266.5892352	2151924.152	6161.391268	16600.1317
0.011987858	1.578162213	0.040784232	852.8692415	0.032291321	0.000415866
0.103811401	10752.00032	930.4253655	1.796525825	92.40164725	4397.009315
632.7980203	2.23060177	340.6038937	118.861307	2352.821802	0.007152493
0.103811401	10752.00032	930.4253655	1.796525825	92.40164725	4397.009315
626.1466263	3.233256262	0.083556604	402.4668722	701.5811738	0.571536667
0.103811401	10752.00032	930.4253655	1.796525825	92.40164725	4397.009315
0.014948631	1.967938295	321.1088231	1242.03174	6.88952025	3.107924374
41332.26009	3.266935407	10146.53011	0.008148624	6281017.939	0.000860879
15.22447571	1.554674281	0.040177237	70.10941609	0.031810726	0.000409676
77.61239172	0.888483377	266.5892352	2151924.152	6161.391268	16600.1317
0.015405246	109.6570687	274.3303944	369.1194492	989.1050501	353.0399182
0.011987858	1.578162213	0.040784232	852.8692415	0.032291321	0.000415866
0.009476463	1.247545308	418.6383503	3.994100994	237.5002741	20.63147071
0.011987858	1.578162213	0.040784232	852.8692415	0.032291321	0.000415866
626.1466263	3.233256262	0.083556604	402.4668722	701.5811738	0.571536667
0.008623181	1.135213607	297.4278164	160.0650048	0.023227997	98.50054455
41332.26009	3.266935407	10146.53011	0.008148624	6281017.939	0.000860879
0.009476463	1.247545308	418.6383503	3.994100994	237.5002741	20.63147071
0.000597272	0.07862887	0.002031995	0.000852615	0.001608852	10.3649392
0.015405246	109.6570687	274.3303944	369.1194492	989.1050501	353.0399182
0.000597272	0.07862887	0.002031995	0.000852615	0.001608852	10.3649392
626.1466263	3.233256262	0.083556604	402.4668722	701.5811738	0.571536667
19.06650031	0.064457932	868.8052699	0.000160776	0.001318896	9.314874595
77.61239172	0.888483377	266.5892352	2151924.152	6161.391268	16600.1317
626.1466263	3.233256262	0.083556604	402.4668722	701.5811738	0.571536667

0.015405246	109.6570687	274.3303944	369.1194492	989.1050501	353.0399182
77.61239172	0.888483377	266.5892352	2151924.152	6161.391268	16600.1317
0.014948631	1.967938295	321.1088231	1242.03174	6.88952025	3.107924374
0.002287645	3421.127911	161133.5523	16.23189513	2645.570264	771.3547188
0.014948631	1.967938295	321.1088231	1242.03174	6.88952025	3.107924374
0.014948631	1.967938295	321.1088231	1242.03174	6.88952025	3.107924374
77.61239172	0.888483377	266.5892352	2151924.152	6161.391268	16600.1317
0.015405246	109.6570687	274.3303944	369.1194492	989.1050501	353.0399182
0.000597272	0.07862887	0.002031995	0.000852615	0.001608852	10.3649392
626.1466263	3.233256262	0.083556604	402.4668722	701.5811738	0.571536667
77.61239172	0.888483377	266.5892352	2151924.152	6161.391268	16600.1317
626.1466263	3.233256262	0.083556604	402.4668722	701.5811738	0.571536667
0.010677332	10306.90517	1001.58427	117.5306001	241.659629	0.000370403
0.014948631	1.967938295	321.1088231	1242.03174	6.88952025	3.107924374
0.014948631	1.967938295	321.1088231	1242.03174	6.88952025	3.107924374
0.009476463	1.247545308	418.6383503	3.994100994	237.5002741	20.63147071
0.020812295	2.739870489	0.070806102	0.391927783	49.12034862	0.126119339
0.000597272	0.07862887	0.002031995	0.000852615	0.001608852	10.3649392
0.011987858	1.578162213	0.040784232	852.8692415	0.032291321	0.000415866
101392.068	1.611193581	0.041637857	11015.75437	204.3346638	48490.28502
626.1466263	3.233256262	0.083556604	402.4668722	701.5811738	0.571536667
19.06650031	0.064457932	868.8052699	0.000160776	0.001318896	9.314874595
0.008623181	1.135213607	297.4278164	160.0650048	0.023227997	98.50054455
0.000536897	0.070680793	48.28889118	25976.69676	194.3603045	0.044758063
0.103811401	10752.00032	930.4253655	1.796525825	92.40164725	4397.009315
0.002287645	3421.127911	161133.5523	16.23189513	2645.570264	771.3547188
39.55229442	774.2950174	0.015376028	312536.0489	124.6097818	636.2423463
576.8221635	2475.022232	53.96825277	31.81190518	581.1972185	2486.626226
45.71571223	19434.15265	29351.49885	7091.325138	0.03245681	0.004846509
626.1466263	3.233256262	0.083556604	402.4668722	701.5811738	0.571536667
0.000597272	0.07862887	0.002031995	0.000852615	0.001608852	10.3649392
0.015405246	109.6570687	274.3303944	369.1194492	989.1050501	353.0399182
105.9732816	6.3929747	0.165212779	122600.7605	0.458528487	3813.563699
0.014948631	1.967938295	321.1088231	1242.03174	6.88952025	3.107924374
77.61239172	0.888483377	266.5892352	2151924.152	6161.391268	16600.1317
45.71571223	19434.15265	29351.49885	7091.325138	0.03245681	0.004846509
0.000536897	0.070680793	48.28889118	25976.69676	194.3603045	0.044758063
0.014948631	1.967938295	321.1088231	1242.03174	6.88952025	3.107924374
0.014948631	1.967938295	321.1088231	1242.03174	6.88952025	3.107924374
101392.068	1.611193581	0.041637857	11015.75437	204.3346638	48490.28502
526445.3132	0.744328439	45.66244823	82879.57285	18.39041237	4838.439694

2474713.365	3.853698492	0.099590608	0.009612171	10563.88602	4.911887668
0.014948631	1.967938295	321.1088231	1242.03174	6.88952025	3.107924374
0.002788108	0.367045261	0.009485501	1111.987943	0.00751024	12.48542726
0.014948631	1.967938295	321.1088231	1242.03174	6.88952025	3.107924374
77.61239172	0.888483377	266.5892352	2151924.152	6161.391268	16600.1317
526445.3132	0.744328439	45.66244823	82879.57285	18.39041237	4838.439694
0.015405246	109.6570687	274.3303944	369.1194492	989.1050501	353.0399182
0.014948631	1.967938295	321.1088231	1242.03174	6.88952025	3.107924374
526445.3132	0.744328439	45.66244823	82879.57285	18.39041237	4838.439694
0.008623181	1.135213607	297.4278164	160.0650048	0.023227997	98.50054455
0.014948631	1.967938295	321.1088231	1242.03174	6.88952025	3.107924374
0.014948631	1.967938295	321.1088231	1242.03174	6.88952025	3.107924374
15.22447571	1.554674281	0.040177237	70.10941609	0.031810726	0.000409676
626.1466263	3.233256262	0.083556604	402.4668722	701.5811738	0.571536667
2474713.365	3.853698492	0.099590608	0.009612171	10563.88602	4.911887668
626.1466263	3.233256262	0.083556604	402.4668722	701.5811738	0.571536667
0.014948631	1.967938295	321.1088231	1242.03174	6.88952025	3.107924374
0.015405246	109.6570687	274.3303944	369.1194492	989.1050501	353.0399182
19.06650031	0.064457932	868.8052699	0.000160776	0.001318896	9.314874595
77.61239172	0.888483377	266.5892352	2151924.152	6161.391268	16600.1317
0.002287645	3421.127911	161133.5523	16.23189513	2645.570264	771.3547188
0.009476463	1.247545308	418.6383503	3.994100994	237.5002741	20.63147071
626.1466263	3.233256262	0.083556604	402.4668722	701.5811738	0.571536667
0.015405246	109.6570687	274.3303944	369.1194492	989.1050501	353.0399182
0.014948631	1.967938295	321.1088231	1242.03174	6.88952025	3.107924374
0.014948631	1.967938295	321.1088231	1242.03174	6.88952025	3.107924374
77.61239172	0.888483377	266.5892352	2151924.152	6161.391268	16600.1317
77.61239172	0.888483377	266.5892352	2151924.152	6161.391268	16600.1317
77.61239172	0.888483377	266.5892352	2151924.152	6161.391268	16600.1317
0.103811401	10752.00032	930.4253655	1.796525825	92.40164725	4397.009315
77.61239172	0.888483377	266.5892352	2151924.152	6161.391268	16600.1317
0.009476463	1.247545308	418.6383503	3.994100994	237.5002741	20.63147071
0.014948631	1.967938295	321.1088231	1242.03174	6.88952025	3.107924374
0.014948631	1.967938295	321.1088231	1242.03174	6.88952025	3.107924374
0.002287645	3421.127911	161133.5523	16.23189513	2645.570264	771.3547188
0.000536897	0.070680793	48.28889118	25976.69676	194.3603045	0.044758063
576.8221635	2475.022232	53.96825277	31.81190518	581.1972185	2486.626226
0.000610059	0.080312331	0.002075501	0.000200321	0.001643298	0.001569903
72.98093717	19683.22675	182.9091078	157.9680679	294.508805	29.33683247
526445.3132	0.744328439	45.66244823	82879.57285	18.39041237	4838.439694
77.61239172	0.888483377	266.5892352	2151924.152	6161.391268	16600.1317

0.009476463	1.247545308	418.6383503	3.994100994	237.5002741	20.63147071
0.008623181	1.135213607	297.4278164	160.0650048	0.023227997	98.50054455
0.008623181	1.135213607	297.4278164	160.0650048	0.023227997	98.50054455
0.008623181	1.135213607	297.4278164	160.0650048	0.023227997	98.50054455
0.014948631	1.967938295	321.1088231	1242.03174	6.88952025	3.107924374
77.61239172	0.888483377	266.5892352	2151924.152	6161.391268	16600.1317
0.014948631	1.967938295	321.1088231	1242.03174	6.88952025	3.107924374
626.1466263	3.233256262	0.083556604	402.4668722	701.5811738	0.571536667
526445.3132	0.744328439	45.66244823	82879.57285	18.39041237	4838.439694
626.1466263	3.233256262	0.083556604	402.4668722	701.5811738	0.571536667
72.98093717	19683.22675	182.9091078	157.9680679	294.508805	29.33683247
77.61239172	0.888483377	266.5892352	2151924.152	6161.391268	16600.1317
0.014948631	1.967938295	321.1088231	1242.03174	6.88952025	3.107924374
0.011987858	1.578162213	0.040784232	852.8692415	0.032291321	0.000415866
626.1466263	3.233256262	0.083556604	402.4668722	701.5811738	0.571536667
4.07342159	0.087421402	11.31481594	0.000218053	0.001788759	0.039418547
77.61239172	0.888483377	266.5892352	2151924.152	6161.391268	16600.1317
77.61239172	0.888483377	266.5892352	2151924.152	6161.391268	16600.1317
0.011987858	1.578162213	0.040784232	852.8692415	0.032291321	0.000415866
19.06650031	0.064457932	868.8052699	0.000160776	0.001318896	9.314874595
77.61239172	0.888483377	266.5892352	2151924.152	6161.391268	16600.1317
105.9732816	6.3929747	0.165212779	122600.7605	0.458528487	3813.563699
626.1466263	3.233256262	0.083556604	402.4668722	701.5811738	0.571536667
0.000597272	0.07862887	0.002031995	0.000852615	0.001608852	10.3649392
0.011987858	1.578162213	0.040784232	852.8692415	0.032291321	0.000415866
15.22447571	1.554674281	0.040177237	70.10941609	0.031810726	0.000409676
77.61239172	0.888483377	266.5892352	2151924.152	6161.391268	16600.1317
632.7980203	2.23060177	340.6038937	118.861307	2352.821802	0.007152493
0.020812295	2.739870489	0.070806102	0.391927783	49.12034862	0.126119339
626.1466263	3.233256262	0.083556604	402.4668722	701.5811738	0.571536667
526445.3132	0.744328439	45.66244823	82879.57285	18.39041237	4838.439694
0.008623181	1.135213607	297.4278164	160.0650048	0.023227997	98.50054455
626.1466263	3.233256262	0.083556604	402.4668722	701.5811738	0.571536667
77.61239172	0.888483377	266.5892352	2151924.152	6161.391268	16600.1317
0.871323227	18.42367298	0.050341233	0.037150284	0.039858171	0.879961062
0.014948631	1.967938295	321.1088231	1242.03174	6.88952025	3.107924374
2474713.365	3.853698492	0.099590608	0.009612171	10563.88602	4.911887668
0.394088162	998.2521398	16.05577584	0.603887652	25.39822402	245337.7573
77.61239172	0.888483377	266.5892352	2151924.152	6161.391268	16600.1317
0.014948631	1.967938295	321.1088231	1242.03174	6.88952025	3.107924374
0.009476463	1.247545308	418.6383503	3.994100994	237.5002741	20.63147071

77.61239172	0.888483377	266.5892352	2151924.152	6161.391268	16600.1317
35.43013637	2156.189912	0.039241621	23.75132877	694.0906976	8.569733235
0.000597272	0.07862887	0.002031995	0.000852615	0.001608852	10.3649392
0.014948631	1.967938295	321.1088231	1242.03174	6.88952025	3.107924374
77.61239172	0.888483377	266.5892352	2151924.152	6161.391268	16600.1317
626.1466263	3.233256262	0.083556604	402.4668722	701.5811738	0.571536667
626.1466263	3.233256262	0.083556604	402.4668722	701.5811738	0.571536667
626.1466263	3.233256262	0.083556604	402.4668722	701.5811738	0.571536667
77.61239172	0.888483377	266.5892352	2151924.152	6161.391268	16600.1317
0.014948631	1.967938295	321.1088231	1242.03174	6.88952025	3.107924374
77.61239172	0.888483377	266.5892352	2151924.152	6161.391268	16600.1317
77.61239172	0.888483377	266.5892352	2151924.152	6161.391268	16600.1317
0.014948631	1.967938295	321.1088231	1242.03174	6.88952025	3.107924374
4.07342159	0.087421402	11.31481594	0.000218053	0.001788759	0.039418547
0.871323227	18.42367298	0.050341233	0.037150284	0.039858171	0.879961062
626.1466263	3.233256262	0.083556604	402.4668722	701.5811738	0.571536667
77.61239172	0.888483377	266.5892352	2151924.152	6161.391268	16600.1317
0.014948631	1.967938295	321.1088231	1242.03174	6.88952025	3.107924374
0.014948631	1.967938295	321.1088231	1242.03174	6.88952025	3.107924374
19.06650031	0.064457932	868.8052699	0.000160776	0.001318896	9.314874595
0.014948631	1.967938295	321.1088231	1242.03174	6.88952025	3.107924374
21287.98288	55979.56656	1185.365727	746.7222688	6673.504885	13367.94956
72.98093717	19683.22675	182.9091078	157.9680679	294.508805	29.33683247
77.61239172	0.888483377	266.5892352	2151924.152	6161.391268	16600.1317
526445.3132	0.744328439	45.66244823	82879.57285	18.39041237	4838.439694
77.61239172	0.888483377	266.5892352	2151924.152	6161.391268	16600.1317
77.61239172	0.888483377	266.5892352	2151924.152	6161.391268	16600.1317
39.55229442	774.2950174	0.015376028	312536.0489	124.6097818	636.2423463
0.009476463	1.247545308	418.6383503	3.994100994	237.5002741	20.63147071
0.008623181	1.135213607	297.4278164	160.0650048	0.023227997	98.50054455
0.014948631	1.967938295	321.1088231	1242.03174	6.88952025	3.107924374
0.020812295	2.739870489	0.070806102	0.391927783	49.12034862	0.126119339
632.7980203	2.23060177	340.6038937	118.861307	2352.821802	0.007152493
0.000536897	0.070680793	48.28889118	25976.69676	194.3603045	0.044758063
626.1466263	3.233256262	0.083556604	402.4668722	701.5811738	0.571536667
0.014948631	1.967938295	321.1088231	1242.03174	6.88952025	3.107924374
626.1466263	3.233256262	0.083556604	402.4668722	701.5811738	0.571536667
35.43013637	2156.189912	0.039241621	23.75132877	694.0906976	8.569733235
21287.98288	55979.56656	1185.365727	746.7222688	6673.504885	13367.94956
77.61239172	0.888483377	266.5892352	2151924.152	6161.391268	16600.1317
19.06650031	0.064457932	868.8052699	0.000160776	0.001318896	9.314874595

632.7980203	2.23060177	340.6038937	118.861307	2352.821802	0.007152493
77.61239172	0.888483377	266.5892352	2151924.152	6161.391268	16600.1317
626.1466263	3.233256262	0.083556604	402.4668722	701.5811738	0.571536667
0.002788108	0.367045261	0.009485501	1111.987943	0.00751024	12.48542726
0.014948631	1.967938295	321.1088231	1242.03174	6.88952025	3.107924374
626.1466263	3.233256262	0.083556604	402.4668722	701.5811738	0.571536667
626.1466263	3.233256262	0.083556604	402.4668722	701.5811738	0.571536667
66.82931254	34792.47757	2740.636219	173.8610139	2539.861059	10.43059205
19.06650031	0.064457932	868.8052699	0.000160776	0.001318896	9.314874595
0.008623181	1.135213607	297.4278164	160.0650048	0.023227997	98.50054455
0.014948631	1.967938295	321.1088231	1242.03174	6.88952025	3.107924374
77.61239172	0.888483377	266.5892352	2151924.152	6161.391268	16600.1317
0.014948631	1.967938295	321.1088231	1242.03174	6.88952025	3.107924374
72.98093717	19683.22675	182.9091078	157.9680679	294.508805	29.33683247
0.000536897	0.070680793	48.28889118	25976.69676	194.3603045	0.044758063
626.1466263	3.233256262	0.083556604	402.4668722	701.5811738	0.571536667
0.000597272	0.07862887	0.002031995	0.000852615	0.001608852	10.3649392
0.009476463	1.247545308	418.6383503	3.994100994	237.5002741	20.63147071
953.7982127	84.08315452	17.62468986	8240.827346	0.078049331	0.004693407
45.71571223	19434.15265	29351.49885	7091.325138	0.03245681	0.004846509
0.000610059	0.080312331	0.002075501	0.000200321	0.001643298	0.001569903
2474713.365	3.853698492	0.099590608	0.009612171	10563.88602	4.911887668
0.011987858	1.578162213	0.040784232	852.8692415	0.032291321	0.000415866
15.22447571	1.554674281	0.040177237	70.10941609	0.031810726	0.000409676
77.61239172	0.888483377	266.5892352	2151924.152	6161.391268	16600.1317
0.014948631	1.967938295	321.1088231	1242.03174	6.88952025	3.107924374
0.000536897	0.070680793	48.28889118	25976.69676	194.3603045	0.044758063
526445.3132	0.744328439	45.66244823	82879.57285	18.39041237	4838.439694
0.000597272	0.07862887	0.002031995	0.000852615	0.001608852	10.3649392
626.1466263	3.233256262	0.083556604	402.4668722	701.5811738	0.571536667
626.1466263	3.233256262	0.083556604	402.4668722	701.5811738	0.571536667
77.61239172	0.888483377	266.5892352	2151924.152	6161.391268	16600.1317
0.014948631	1.967938295	321.1088231	1242.03174	6.88952025	3.107924374
0.009476463	1.247545308	418.6383503	3.994100994	237.5002741	20.63147071
0.002788108	0.367045261	0.009485501	1111.987943	0.00751024	12.48542726
150365.2287	45.43559849	0.009823085	1.943123938	122.4287388	25.08318135
77.61239172	0.888483377	266.5892352	2151924.152	6161.391268	16600.1317
0.014948631	1.967938295	321.1088231	1242.03174	6.88952025	3.107924374
0.103811401	10752.00032	930.4253655	1.796525825	92.40164725	4397.009315
0.014948631	1.967938295	321.1088231	1242.03174	6.88952025	3.107924374
0.015405246	109.6570687	274.3303944	369.1194492	989.1050501	353.0399182

0.011987858	1.578162213	0.040784232	852.8692415	0.032291321	0.000415866
626.1466263	3.233256262	0.083556604	402.4668722	701.5811738	0.571536667
0.009476463	1.247545308	418.6383503	3.994100994	237.5002741	20.63147071
0.000597272	0.07862887	0.002031995	0.000852615	0.001608852	10.3649392
72.98093717	19683.22675	182.9091078	157.9680679	294.508805	29.33683247
77.61239172	0.888483377	266.5892352	2151924.152	6161.391268	16600.1317
0.008623181	1.135213607	297.4278164	160.0650048	0.023227997	98.50054455
0.000610059	0.080312331	0.002075501	0.000200321	0.001643298	0.001569903
0.000536897	0.070680793	48.28889118	25976.69676	194.3603045	0.044758063
0.015405246	109.6570687	274.3303944	369.1194492	989.1050501	353.0399182
0.000597272	0.07862887	0.002031995	0.000852615	0.001608852	10.3649392
39.55229442	774.2950174	0.015376028	312536.0489	124.6097818	636.2423463
0.008623181	1.135213607	297.4278164	160.0650048	0.023227997	98.50054455
0.871323227	18.42367298	0.050341233	0.037150284	0.039858171	0.879961062
0.014948631	1.967938295	321.1088231	1242.03174	6.88952025	3.107924374
0.008623181	1.135213607	297.4278164	160.0650048	0.023227997	98.50054455
0.014948631	1.967938295	321.1088231	1242.03174	6.88952025	3.107924374
0.000597272	0.07862887	0.002031995	0.000852615	0.001608852	10.3649392
626.1466263	3.233256262	0.083556604	402.4668722	701.5811738	0.571536667
0.000597272	0.07862887	0.002031995	0.000852615	0.001608852	10.3649392
77.61239172	0.888483377	266.5892352	2151924.152	6161.391268	16600.1317
626.1466263	3.233256262	0.083556604	402.4668722	701.5811738	0.571536667
77.61239172	0.888483377	266.5892352	2151924.152	6161.391268	16600.1317
0.020812295	2.739870489	0.070806102	0.391927783	49.12034862	0.126119339
0.000597272	0.07862887	0.002031995	0.000852615	0.001608852	10.3649392
632.7980203	2.23060177	340.6038937	118.861307	2352.821802	0.007152493
2474713.365	3.853698492	0.099590608	0.009612171	10563.88602	4.911887668
626.1466263	3.233256262	0.083556604	402.4668722	701.5811738	0.571536667
0.011987858	1.578162213	0.040784232	852.8692415	0.032291321	0.000415866
626.1466263	3.233256262	0.083556604	402.4668722	701.5811738	0.571536667
0.020812295	2.739870489	0.070806102	0.391927783	49.12034862	0.126119339
0.000597272	0.07862887	0.002031995	0.000852615	0.001608852	10.3649392
101392.068	1.611193581	0.041637857	11015.75437	204.3346638	48490.28502
0.000536897	0.070680793	48.28889118	25976.69676	194.3603045	0.044758063
39.55229442	774.2950174	0.015376028	312536.0489	124.6097818	636.2423463
0.000536897	0.070680793	48.28889118	25976.69676	194.3603045	0.044758063
77.61239172	0.888483377	266.5892352	2151924.152	6161.391268	16600.1317
150365.2287	45.43559849	0.009823085	1.943123938	122.4287388	25.08318135
0.000597272	0.07862887	0.002031995	0.000852615	0.001608852	10.3649392
526445.3132	0.744328439	45.66244823	82879.57285	18.39041237	4838.439694
21287.98288	55979.56656	1185.365727	746.7222688	6673.504885	13367.94956

150365.2287	45.43559849	0.009823085	1.943123938	122.4287388	25.08318135
15.22447571	1.554674281	0.040177237	70.10941609	0.031810726	0.000409676
0.014948631	1.967938295	321.1088231	1242.03174	6.88952025	3.107924374
0.014948631	1.967938295	321.1088231	1242.03174	6.88952025	3.107924374
77.61239172	0.888483377	266.5892352	2151924.152	6161.391268	16600.1317
101392.068	1.611193581	0.041637857	11015.75437	204.3346638	48490.28502
0.103811401	10752.00032	930.4253655	1.796525825	92.40164725	4397.009315
4823711.483	3284.337376	642.3911258	8.613380148	0.026315151	67.81256821
0.011987858	1.578162213	0.040784232	852.8692415	0.032291321	0.000415866
101392.068	1.611193581	0.041637857	11015.75437	204.3346638	48490.28502
0.012547577	3.348872813	0.042688471	0.004120156	0.033799021	0.000435283
0.014948631	1.967938295	321.1088231	1242.03174	6.88952025	3.107924374
0.009476463	1.247545308	418.6383503	3.994100994	237.5002741	20.63147071
0.394088162	998.2521398	16.05577584	0.603887652	25.39822402	245337.7573
0.010677332	10306.90517	1001.58427	117.5306001	241.659629	0.000370403
101392.068	1.611193581	0.041637857	11015.75437	204.3346638	48490.28502
21287.98288	55979.56656	1185.365727	746.7222688	6673.504885	13367.94956
0.000597272	0.07862887	0.002031995	0.000852615	0.001608852	10.3649392
0.014948631	1.967938295	321.1088231	1242.03174	6.88952025	3.107924374
41332.26009	3.266935407	10146.53011	0.008148624	6281017.939	0.000860879
626.1466263	3.233256262	0.083556604	402.4668722	701.5811738	0.571536667
526445.3132	0.744328439	45.66244823	82879.57285	18.39041237	4838.439694
77.61239172	0.888483377	266.5892352	2151924.152	6161.391268	16600.1317
576.8221635	2475.022232	53.96825277	31.81190518	581.1972185	2486.626226
0.008623181	1.135213607	297.4278164	160.0650048	0.023227997	98.50054455
0.015405246	109.6570687	274.3303944	369.1194492	989.1050501	353.0399182
105.9732816	6.3929747	0.165212779	122600.7605	0.458528487	3813.563699
626.1466263	3.233256262	0.083556604	402.4668722	701.5811738	0.571536667
526445.3132	0.744328439	45.66244823	82879.57285	18.39041237	4838.439694
0.015405246	109.6570687	274.3303944	369.1194492	989.1050501	353.0399182
0.000597272	0.07862887	0.002031995	0.000852615	0.001608852	10.3649392
101392.068	1.611193581	0.041637857	11015.75437	204.3346638	48490.28502
526445.3132	0.744328439	45.66244823	82879.57285	18.39041237	4838.439694
150365.2287	45.43559849	0.009823085	1.943123938	122.4287388	25.08318135
4823711.483	3284.337376	642.3911258	8.613380148	0.026315151	67.81256821
101392.068	1.611193581	0.041637857	11015.75437	204.3346638	48490.28502
0.014948631	1.967938295	321.1088231	1242.03174	6.88952025	3.107924374
632.7980203	2.23060177	340.6038937	118.861307	2352.821802	0.007152493
101392.068	1.611193581	0.041637857	11015.75437	204.3346638	48490.28502
0.008623181	1.135213607	297.4278164	160.0650048	0.023227997	98.50054455
0.014948631	1.967938295	321.1088231	1242.03174	6.88952025	3.107924374

626.1466263	3.233256262	0.083556604	402.4668722	701.5811738	0.571536667
41332.26009	3.266935407	10146.53011	0.008148624	6281017.939	0.000860879
0.015405246	109.6570687	274.3303944	369.1194492	989.1050501	353.0399182
0.871323227	18.42367298	0.050341233	0.037150284	0.039858171	0.879961062
72.98093717	19683.22675	182.9091078	157.9680679	294.508805	29.33683247
576.8221635	2475.022232	53.96825277	31.81190518	581.1972185	2486.626226
19.06650031	0.064457932	868.8052699	0.000160776	0.001318896	9.314874595
0.008623181	1.135213607	297.4278164	160.0650048	0.023227997	98.50054455
0.008623181	1.135213607	297.4278164	160.0650048	0.023227997	98.50054455
77.61239172	0.888483377	266.5892352	2151924.152	6161.391268	16600.1317
526445.3132	0.744328439	45.66244823	82879.57285	18.39041237	4838.439694
0.015405246	109.6570687	274.3303944	369.1194492	989.1050501	353.0399182
21287.98288	55979.56656	1185.365727	746.7222688	6673.504885	13367.94956
626.1466263	3.233256262	0.083556604	402.4668722	701.5811738	0.571536667
72.98093717	19683.22675	182.9091078	157.9680679	294.508805	29.33683247
77.61239172	0.888483377	266.5892352	2151924.152	6161.391268	16600.1317
626.1466263	3.233256262	0.083556604	402.4668722	701.5811738	0.571536667
6.433193314	0.663480649	203.6340103	205.3135512	3631.52014	960828.0776
66.82931254	34792.47757	2740.636219	173.8610139	2539.861059	10.43059205
72.98093717	19683.22675	182.9091078	157.9680679	294.508805	29.33683247
45.71571223	19434.15265	29351.49885	7091.325138	0.03245681	0.004846509
21287.98288	55979.56656	1185.365727	746.7222688	6673.504885	13367.94956
19.06650031	0.064457932	868.8052699	0.000160776	0.001318896	9.314874595
0.011987858	1.578162213	0.040784232	852.8692415	0.032291321	0.000415866
0.010677332	10306.90517	1001.58427	117.5306001	241.659629	0.000370403
0.000536897	0.070680793	48.28889118	25976.69676	194.3603045	0.044758063
0.015405246	109.6570687	274.3303944	369.1194492	989.1050501	353.0399182
0.009476463	1.247545308	418.6383503	3.994100994	237.5002741	20.63147071
0.014948631	1.967938295	321.1088231	1242.03174	6.88952025	3.107924374
0.002788108	0.367045261	0.009485501	1111.987943	0.00751024	12.48542726
4.07342159	0.087421402	11.31481594	0.000218053	0.001788759	0.039418547
626.1466263	3.233256262	0.083556604	402.4668722	701.5811738	0.571536667
21287.98288	55979.56656	1185.365727	746.7222688	6673.504885	13367.94956
0.015405246	109.6570687	274.3303944	369.1194492	989.1050501	353.0399182
0.015405246	109.6570687	274.3303944	369.1194492	989.1050501	353.0399182
45.71571223	19434.15265	29351.49885	7091.325138	0.03245681	0.004846509
526445.3132	0.744328439	45.66244823	82879.57285	18.39041237	4838.439694
150365.2287	45.43559849	0.009823085	1.943123938	122.4287388	25.08318135
0.871323227	18.42367298	0.050341233	0.037150284	0.039858171	0.879961062
0.008623181	1.135213607	297.4278164	160.0650048	0.023227997	98.50054455
0.394088162	998.2521398	16.05577584	0.603887652	25.39822402	245337.7573

0.012547577	3.348872813	0.042688471	0.004120156	0.033799021	0.000435283
626.1466263	3.233256262	0.083556604	402.4668722	701.5811738	0.571536667
0.009476463	1.247545308	418.6383503	3.994100994	237.5002741	20.63147071
0.008623181	1.135213607	297.4278164	160.0650048	0.023227997	98.50054455
626.1466263	3.233256262	0.083556604	402.4668722	701.5811738	0.571536667
0.011987858	1.578162213	0.040784232	852.8692415	0.032291321	0.000415866
101392.068	1.611193581	0.041637857	11015.75437	204.3346638	48490.28502
526445.3132	0.744328439	45.66244823	82879.57285	18.39041237	4838.439694
21287.98288	55979.56656	1185.365727	746.7222688	6673.504885	13367.94956
77.61239172	0.888483377	266.5892352	2151924.152	6161.391268	16600.1317
526445.3132	0.744328439	45.66244823	82879.57285	18.39041237	4838.439694
0.014948631	1.967938295	321.1088231	1242.03174	6.88952025	3.107924374
0.394088162	998.2521398	16.05577584	0.603887652	25.39822402	245337.7573
0.008623181	1.135213607	297.4278164	160.0650048	0.023227997	98.50054455
576.8221635	2475.022232	53.96825277	31.81190518	581.1972185	2486.626226
77.61239172	0.888483377	266.5892352	2151924.152	6161.391268	16600.1317
576.8221635	2475.022232	53.96825277	31.81190518	581.1972185	2486.626226
77.61239172	0.888483377	266.5892352	2151924.152	6161.391268	16600.1317
39	40	41	42	43	44
UBASH3A-Hs00955168_m1	PFKL-Hs01040525_m1	CHODL-Hs01070471_m1	PKNOX1-Hs01007098_m1	PKNOX1-Hs01007097_m1	PKNOX1-Hs01007094_m1

45	46	47	48	49	50
PKNOX1-Hs01007093_m1	PKNOX1-Hs01007092_m1	PKNOX1-Hs00231814_m1	CYYR1-Hs00951849_m1	SLC19A1-Hs00953342_m1	SLC19A1-Hs00953341_m1
48.41056772	101232.0971	1.94E-06	37.36817424	1.320387317	0.000902681
0.031033768	0.000308131	0.519206227	2.252078542	90.19285362	0.000880645
59841.21985	241.6059154	1291123.069	2.084242854	825922.5084	1427.862261
40.3670238	19.34889771	3.570475575	2591.849001	0.017116939	405.3750403
0.000161642	0.251672652	9.624883464	20916.67392	1.821079194	3.832538343
308.6230019	306.2303081	64.04525386	63.75477531	153.2062338	0.166051589
30.49306552	15211529.41	13.45644866	42.5397454	83.9410448	183.49573
48.04882597	710752.5842	0.77664186	0.000187569	252.7585725	8.321384451
2.887814914	0.629515385	0.957460813	12.22046995	26.28153688	1127023.074
0.45957143	56681761.57	1.074941502	0.002213533	0.00243123	0.016562319
5153.715785	0.005921236	1367.703162	0.002261739	12.50438035	0.537816724
40.3670238	19.34889771	3.570475575	2591.849001	0.017116939	405.3750403
40.02663594	395.2312814	0.372324771	1565.239501	245.7934325	107508.4816
2.887814914	0.629515385	0.957460813	12.22046995	26.28153688	1127023.074
86.58517476	21.2023281	50.86800811	18.83992988	15.12741699	0.019517004
0.001381711	0.061193653	0.010959458	15624.79993	981.8668457	86.45489977
74.95065829	22033.06748	0.14280576	24.67190412	366.076064	649.4828957
1398.546865	165.0671432	0.423838598	0.000659616	96628.37594	0.004935445
59841.21985	241.6059154	1291123.069	2.084242854	825922.5084	1427.862261
7834.061059	0.026488345	29.49424129	0.010117772	71.8798407	0.075704201
0.001381711	0.061193653	0.010959458	15624.79993	981.8668457	86.45489977
19561908.1	0.009656057	5.272012808	506.1903674	7.125366856	0.027597198
422.4633154	1153.53267	1.04284868	2563117.399	688.7736212	1658.976205
5153.715785	0.005921236	1367.703162	0.002261739	12.50438035	0.537816724
0.000161642	0.251672652	9.624883464	20916.67392	1.821079194	3.832538343
422.4633154	1153.53267	1.04284868	2563117.399	688.7736212	1658.976205
0.45957143	56681761.57	1.074941502	0.002213533	0.00243123	0.016562319
5153.715785	0.005921236	1367.703162	0.002261739	12.50438035	0.537816724
86.58517476	21.2023281	50.86800811	18.83992988	15.12741699	0.019517004
2.887814914	0.629515385	0.957460813	12.22046995	26.28153688	1127023.074
48.41056772	101232.0971	1.94E-06	37.36817424	1.320387317	0.000902681
204.0576898	12079.88214	11.43961314	32.42699195	25.66420716	256991.0612
0.001381711	0.061193653	0.010959458	15624.79993	981.8668457	86.45489977
308.6230019	306.2303081	64.04525386	63.75477531	153.2062338	0.166051589
4.409381321	210.7227323	0.045666454	38802711.04	12.08494279	609.8674319
0.45957143	56681761.57	1.074941502	0.002213533	0.00243123	0.016562319
19561908.1	0.009656057	5.272012808	506.1903674	7.125366856	0.027597198

422.4633154	1153.53267	1.04284868	2563117.399	688.7736212	1658.976205
308.6230019	306.2303081	64.04525386	63.75477531	153.2062338	0.166051589
0.047413054	2644.257632	91908.47278	68105.57087	83.91115134	201.2591821
0.001381711	0.061193653	0.010959458	15624.79993	981.8668457	86.45489977
0.45957143	56681761.57	1.074941502	0.002213533	0.00243123	0.016562319
308.6230019	306.2303081	64.04525386	63.75477531	153.2062338	0.166051589
204.0576898	12079.88214	11.43961314	32.42699195	25.66420716	256991.0612
7834.061059	0.026488345	29.49424129	0.010117772	71.8798407	0.075704201
0.000161642	0.251672652	9.624883464	20916.67392	1.821079194	3.832538343
0.047413054	2644.257632	91908.47278	68105.57087	83.91115134	201.2591821
0.000161642	0.251672652	9.624883464	20916.67392	1.821079194	3.832538343
59841.21985	241.6059154	1291123.069	2.084242854	825922.5084	1427.862261
19561908.1	0.009656057	5.272012808	506.1903674	7.125366856	0.027597198
0.000482159	0.002521237	1.55E-05	0.005506384	0.001057752	0.007205744
793236.9018	19681.0082	12741.86914	140.6064572	2.851419285	1.100140828
4119.001973	12.96182847	97978.19598	3026.535321	0.509946024	266.4579467
507.1011936	1478.891687	1511.869022	2939.496313	32.29524662	0.873728374
0.00210413	0.120368286	41.98575599	332885.5239	0.002614266	169.8590328
0.881985989	0.027067935	39.49331828	429342.378	0.011355996	0.077360679
2492617.667	0.432218488	0.519781662	0.005046575	48444.92742	0.037759983
507.1011936	1478.891687	1511.869022	2939.496313	32.29524662	0.873728374
53.92016893	54780.47857	2.398800153	0.000251436	1134.11636	7.274232584
5737.994112	2783120.43	5.128574608	513876.0139	1303.951778	1759960.698
4119.001973	12.96182847	97978.19598	3026.535321	0.509946024	266.4579467
0.001422464	0.00941797	0.204926973	0.721276114	12.3232199	3478.146114
0.881985989	0.027067935	39.49331828	429342.378	0.011355996	0.077360679
68457.92115	2505.973416	28.92950345	64.45951709	1388.585013	0.042603568
0.001422464	0.00941797	0.204926973	0.721276114	12.3232199	3478.146114
507.1011936	1478.891687	1511.869022	2939.496313	32.29524662	0.873728374
0.881985989	0.027067935	39.49331828	429342.378	0.011355996	0.077360679
1407.3203	17.21923482	3.133003664	0.001215493	3.484959996	13.52883657
246.9539207	3128.825214	3320.500866	1022.604151	660.0289615	3.439133266
53.92016893	54780.47857	2.398800153	0.000251436	1134.11636	7.274232584
0.001422464	0.00941797	0.204926973	0.721276114	12.3232199	3478.146114
46.88689043	0.391622165	531.2950075	23.94964113	22.18992424	0.001542256
507.1011936	1478.891687	1511.869022	2939.496313	32.29524662	0.873728374
2492617.667	0.432218488	0.519781662	0.005046575	48444.92742	0.037759983
0.001422464	0.00941797	0.204926973	0.721276114	12.3232199	3478.146114
507.1011936	1478.891687	1511.869022	2939.496313	32.29524662	0.873728374
0.001422464	0.00941797	0.204926973	0.721276114	12.3232199	3478.146114
122.6515324	19.5047941	0.94053901	0.006485206	5.827767614	142.178948

391.5511783	119356784.6	280778.3653	69.86173262	201.6378553	0.129436783
793236.9018	19681.0082	12741.86914	140.6064572	2.851419285	1.100140828
4119.001973	12.96182847	97978.19598	3026.535321	0.509946024	266.4579467
0.00210413	0.120368286	41.98575599	332885.5239	0.002614266	169.8590328
265.8638962	2.574642456	0.000899196	35.21442232	222.1223505	569.0275115
53.92016893	54780.47857	2.398800153	0.000251436	1134.11636	7.274232584
122.6515324	19.5047941	0.94053901	0.006485206	5.827767614	142.178948
83.20846796	78551.74488	2.927555361	6.945833152	119.7214744	656.7244025
53.92016893	54780.47857	2.398800153	0.000251436	1134.11636	7.274232584
0.00253959	292.6377976	5956.773245	0.105152004	10617393.69	1.849463033
46.88689043	0.391622165	531.2950075	23.94964113	22.18992424	0.001542256
2.357992593	0.013488471	0.163257793	13.4799025	2.499363915	26.17785932
0.881985989	0.027067935	39.49331828	429342.378	0.011355996	0.077360679
507.1011936	1478.891687	1511.869022	2939.496313	32.29524662	0.873728374
0.881985989	0.027067935	39.49331828	429342.378	0.011355996	0.077360679
0.001422464	0.00941797	0.204926973	0.721276114	12.3232199	3478.146114
0.001422464	0.00941797	0.204926973	0.721276114	12.3232199	3478.146114
0.001422464	0.00941797	0.204926973	0.721276114	12.3232199	3478.146114
0.001422464	0.00941797	0.204926973	0.721276114	12.3232199	3478.146114
0.001422464	0.00941797	0.204926973	0.721276114	12.3232199	3478.146114
42.05187837	28157.9058	5.60105163	14.51839423	31.08904201	345.0495564
53.92016893	54780.47857	2.398800153	0.000251436	1134.11636	7.274232584
507.1011936	1478.891687	1511.869022	2939.496313	32.29524662	0.873728374
4119.001973	12.96182847	97978.19598	3026.535321	0.509946024	266.4579467
507.1011936	1478.891687	1511.869022	2939.496313	32.29524662	0.873728374
0.881985989	0.027067935	39.49331828	429342.378	0.011355996	0.077360679
53.92016893	54780.47857	2.398800153	0.000251436	1134.11636	7.274232584
0.002489037	0.013015307	0.490936354	2.791297441	1002.558859	0.037197998
0.001422464	0.00941797	0.204926973	0.721276114	12.3232199	3478.146114
0.001422464	0.00941797	0.204926973	0.721276114	12.3232199	3478.146114
751.4658587	6.515515762	58.76152474	0.01232318	643.3277344	6658.145091
25.36093266	0.030713913	5059.993294	0.003630134	333.5791205	24245.75888
0.002489037	0.013015307	0.490936354	2.791297441	1002.558859	0.037197998
0.881985989	0.027067935	39.49331828	429342.378	0.011355996	0.077360679
0.002644611	0.013828814	2012.047599	0.005282202	16148.89966	9.589666162
5737.994112	2783120.43	5.128574608	513876.0139	1303.951778	1759960.698
0.000482159	0.002521237	1.55E-05	0.005506384	0.001057752	0.007205744
83.20846796	78551.74488	2.927555361	6.945833152	119.7214744	656.7244025
0.00210413	0.120368286	41.98575599	332885.5239	0.002614266	169.8590328
0.881985989	0.027067935	39.49331828	429342.378	0.011355996	0.077360679
83.20846796	78551.74488	2.927555361	6.945833152	119.7214744	656.7244025

0.001422464	0.00941797	0.204926973	0.721276114	12.3232199	3478.146114
3820.009616	7.957270648	1.582118024	34.54294407	0.007834425	98.70656889
0.881985989	0.027067935	39.49331828	429342.378	0.011355996	0.077360679
5737.994112	2783120.43	5.128574608	513876.0139	1303.951778	1759960.698
507.1011936	1478.891687	1511.869022	2939.496313	32.29524662	0.873728374
0.881985989	0.027067935	39.49331828	429342.378	0.011355996	0.077360679
4119.001973	12.96182847	97978.19598	3026.535321	0.509946024	266.4579467
3820.009616	7.957270648	1.582118024	34.54294407	0.007834425	98.70656889
0.881985989	0.027067935	39.49331828	429342.378	0.011355996	0.077360679
507.1011936	1478.891687	1511.869022	2939.496313	32.29524662	0.873728374
0.001422464	0.00941797	0.204926973	0.721276114	12.3232199	3478.146114
2492617.667	0.432218488	0.519781662	0.005046575	48444.92742	0.037759983
0.881985989	0.027067935	39.49331828	429342.378	0.011355996	0.077360679
507.1011936	1478.891687	1511.869022	2939.496313	32.29524662	0.873728374
0.881985989	0.027067935	39.49331828	429342.378	0.011355996	0.077360679
2492617.667	0.432218488	0.519781662	0.005046575	48444.92742	0.037759983
0.881985989	0.027067935	39.49331828	429342.378	0.011355996	0.077360679
303.7647121	0.040367691	88825.40181	31.83876004	65.96527827	23.08359483
0.002489037	0.013015307	0.490936354	2.791297441	1002.558859	0.037197998
46.88689043	0.391622165	531.2950075	23.94964113	22.18992424	0.001542256
10.83050708	42477.74287	13.76429903	68103.17337	10984.57956	0.001691148
42.05187837	28157.9058	5.60105163	14.51839423	31.08904201	345.0495564
507.1011936	1478.891687	1511.869022	2939.496313	32.29524662	0.873728374
246.9539207	3128.825214	3320.500866	1022.604151	660.0289615	3.439133266
0.00210413	0.120368286	41.98575599	332885.5239	0.002614266	169.8590328
25.36093266	0.030713913	5059.993294	0.003630134	333.5791205	24245.75888
3820.009616	7.957270648	1.582118024	34.54294407	0.007834425	98.70656889
2492617.667	0.432218488	0.519781662	0.005046575	48444.92742	0.037759983
0.004386538	612.351195	3834.828045	27.88750645	0.673629365	25.66378743
10.83050708	42477.74287	13.76429903	68103.17337	10984.57956	0.001691148
507.1011936	1478.891687	1511.869022	2939.496313	32.29524662	0.873728374
122.6515324	19.5047941	0.94053901	0.006485206	5.827767614	142.178948
3820.009616	7.957270648	1.582118024	34.54294407	0.007834425	98.70656889
507.1011936	1478.891687	1511.869022	2939.496313	32.29524662	0.873728374
0.00210413	0.120368286	41.98575599	332885.5239	0.002614266	169.8590328
0.001422464	0.00941797	0.204926973	0.721276114	12.3232199	3478.146114
0.881985989	0.027067935	39.49331828	429342.378	0.011355996	0.077360679
793236.9018	19681.0082	12741.86914	140.6064572	2.851419285	1.100140828
507.1011936	1478.891687	1511.869022	2939.496313	32.29524662	0.873728374
507.1011936	1478.891687	1511.869022	2939.496313	32.29524662	0.873728374
2492617.667	0.432218488	0.519781662	0.005046575	48444.92742	0.037759983

83.20846796	78551.74488	2.927555361	6.945833152	119.7214744	656.7244025
42.05187837	28157.9058	5.60105163	14.51839423	31.08904201	345.0495564
234.174525	19.21560603	0.019944373	167.453655	157.6983767	276.7276739
0.025054755	62060.76279	108.4091933	1.659943972	0.006841767	0.046608309
16.85488392	145222.0361	393.9165404	54.44420646	63.29111729	0.030771725
46.88689043	0.391622165	531.2950075	23.94964113	22.18992424	0.001542256
3820.009616	7.957270648	1.582118024	34.54294407	0.007834425	98.70656889
53.92016893	54780.47857	2.398800153	0.000251436	1134.11636	7.274232584
5737.994112	2783120.43	5.128574608	513876.0139	1303.951778	1759960.698
0.002489037	0.013015307	0.490936354	2.791297441	1002.558859	0.037197998
10.83050708	42477.74287	13.76429903	68103.17337	10984.57956	0.001691148
122.6515324	19.5047941	0.94053901	0.006485206	5.827767614	142.178948
0.001422464	0.00941797	0.204926973	0.721276114	12.3232199	3478.146114
0.001422464	0.00941797	0.204926973	0.721276114	12.3232199	3478.146114
53.92016893	54780.47857	2.398800153	0.000251436	1134.11636	7.274232584
0.001422464	0.00941797	0.204926973	0.721276114	12.3232199	3478.146114
0.001422464	0.00941797	0.204926973	0.721276114	12.3232199	3478.146114
0.001422464	0.00941797	0.204926973	0.721276114	12.3232199	3478.146114
0.001422464	0.00941797	0.204926973	0.721276114	12.3232199	3478.146114
53.92016893	54780.47857	2.398800153	0.000251436	1134.11636	7.274232584
0.881985989	0.027067935	39.49331828	429342.378	0.011355996	0.077360679
0.001422464	0.00941797	0.204926973	0.721276114	12.3232199	3478.146114
507.1011936	1478.891687	1511.869022	2939.496313	32.29524662	0.873728374
0.001422464	0.00941797	0.204926973	0.721276114	12.3232199	3478.146114
1407.3203	17.21923482	3.133003664	0.001215493	3.484959996	13.52883657
25.36093266	0.030713913	5059.993294	0.003630134	333.5791205	24245.75888
122.6515324	19.5047941	0.94053901	0.006485206	5.827767614	142.178948
122.6515324	19.5047941	0.94053901	0.006485206	5.827767614	142.178948
793236.9018	19681.0082	12741.86914	140.6064572	2.851419285	1.100140828
26.73621697	0.00719895	0.195071634	5952.751845	2.366244668	5.51508649
0.001422464	0.00941797	0.204926973	0.721276114	12.3232199	3478.146114
507.1011936	1478.891687	1511.869022	2939.496313	32.29524662	0.873728374
0.004386538	612.351195	3834.828045	27.88750645	0.673629365	25.66378743
0.007277636	1.909846368	0.943378642	2.382431085	628707.3608	22620.20466
507.1011936	1478.891687	1511.869022	2939.496313	32.29524662	0.873728374
0.004386538	612.351195	3834.828045	27.88750645	0.673629365	25.66378743
83.20846796	78551.74488	2.927555361	6.945833152	119.7214744	656.7244025
4119.001973	12.96182847	97978.19598	3026.535321	0.509946024	266.4579467
507.1011936	1478.891687	1511.869022	2939.496313	32.29524662	0.873728374
507.1011936	1478.891687	1511.869022	2939.496313	32.29524662	0.873728374
0.001422464	0.00941797	0.204926973	0.721276114	12.3232199	3478.146114

0.004386538	612.351195	3834.828045	27.88750645	0.673629365	25.66378743
53.92016893	54780.47857	2.398800153	0.000251436	1134.11636	7.274232584
0.001422464	0.00941797	0.204926973	0.721276114	12.3232199	3478.146114
25.36093266	0.030713913	5059.993294	0.003630134	333.5791205	24245.75888
507.1011936	1478.891687	1511.869022	2939.496313	32.29524662	0.873728374
391.5511783	119356784.6	280778.3653	69.86173262	201.6378553	0.129436783
0.00210413	0.120368286	41.98575599	332885.5239	0.002614266	169.8590328
391.5511783	119356784.6	280778.3653	69.86173262	201.6378553	0.129436783
5737.994112	2783120.43	5.128574608	513876.0139	1303.951778	1759960.698
10.83050708	42477.74287	13.76429903	68103.17337	10984.57956	0.001691148
2492617.667	0.432218488	0.519781662	0.005046575	48444.92742	0.037759983
0.881985989	0.027067935	39.49331828	429342.378	0.011355996	0.077360679
507.1011936	1478.891687	1511.869022	2939.496313	32.29524662	0.873728374
0.00210413	0.120368286	41.98575599	332885.5239	0.002614266	169.8590328
0.001422464	0.00941797	0.204926973	0.721276114	12.3232199	3478.146114
2492617.667	0.432218488	0.519781662	0.005046575	48444.92742	0.037759983
42.05187837	28157.9058	5.60105163	14.51839423	31.08904201	345.0495564
2.357992593	0.013488471	0.163257793	13.4799025	2.499363915	26.17785932
793236.9018	19681.0082	12741.86914	140.6064572	2.851419285	1.100140828
0.004386538	612.351195	3834.828045	27.88750645	0.673629365	25.66378743
25.36093266	0.030713913	5059.993294	0.003630134	333.5791205	24245.75888
0.881985989	0.027067935	39.49331828	429342.378	0.011355996	0.077360679
83.20846796	78551.74488	2.927555361	6.945833152	119.7214744	656.7244025
122.6515324	19.5047941	0.94053901	0.006485206	5.827767614	142.178948
46.88689043	0.391622165	531.2950075	23.94964113	22.18992424	0.001542256
0.881985989	0.027067935	39.49331828	429342.378	0.011355996	0.077360679
0.001422464	0.00941797	0.204926973	0.721276114	12.3232199	3478.146114
0.001422464	0.00941797	0.204926973	0.721276114	12.3232199	3478.146114
0.00210413	0.120368286	41.98575599	332885.5239	0.002614266	169.8590328
0.001422464	0.00941797	0.204926973	0.721276114	12.3232199	3478.146114
2492617.667	0.432218488	0.519781662	0.005046575	48444.92742	0.037759983
0.001422464	0.00941797	0.204926973	0.721276114	12.3232199	3478.146114
0.001422464	0.00941797	0.204926973	0.721276114	12.3232199	3478.146114
0.881985989	0.027067935	39.49331828	429342.378	0.011355996	0.077360679
0.881985989	0.027067935	39.49331828	429342.378	0.011355996	0.077360679
507.1011936	1478.891687	1511.869022	2939.496313	32.29524662	0.873728374
53.92016893	54780.47857	2.398800153	0.000251436	1134.11636	7.274232584
0.881985989	0.027067935	39.49331828	429342.378	0.011355996	0.077360679
46.88689043	0.391622165	531.2950075	23.94964113	22.18992424	0.001542256
0.001422464	0.00941797	0.204926973	0.721276114	12.3232199	3478.146114
507.1011936	1478.891687	1511.869022	2939.496313	32.29524662	0.873728374

2492617.667	0.432218488	0.519781662	0.005046575	48444.92742	0.037759983
0.004386538	612.351195	3834.828045	27.88750645	0.673629365	25.66378743
53.92016893	54780.47857	2.398800153	0.000251436	1134.11636	7.274232584
246.9539207	3128.825214	3320.500866	1022.604151	660.0289615	3.439133266
122.6515324	19.5047941	0.94053901	0.006485206	5.827767614	142.178948
391.5511783	119356784.6	280778.3653	69.86173262	201.6378553	0.129436783
0.00210413	0.120368286	41.98575599	332885.5239	0.002614266	169.8590328
25.36093266	0.030713913	5059.993294	0.003630134	333.5791205	24245.75888
10.83050708	42477.74287	13.76429903	68103.17337	10984.57956	0.001691148
507.1011936	1478.891687	1511.869022	2939.496313	32.29524662	0.873728374
0.00210413	0.120368286	41.98575599	332885.5239	0.002614266	169.8590328
10.83050708	42477.74287	13.76429903	68103.17337	10984.57956	0.001691148
25.36093266	0.030713913	5059.993294	0.003630134	333.5791205	24245.75888
83.20846796	78551.74488	2.927555361	6.945833152	119.7214744	656.7244025
3820.009616	7.957270648	1.582118024	34.54294407	0.007834425	98.70656889
507.1011936	1478.891687	1511.869022	2939.496313	32.29524662	0.873728374
46.88689043	0.391622165	531.2950075	23.94964113	22.18992424	0.001542256
42.05187837	28157.9058	5.60105163	14.51839423	31.08904201	345.0495564
122.6515324	19.5047941	0.94053901	0.006485206	5.827767614	142.178948
0.001422464	0.00941797	0.204926973	0.721276114	12.3232199	3478.146114
0.00210413	0.120368286	41.98575599	332885.5239	0.002614266	169.8590328
0.001422464	0.00941797	0.204926973	0.721276114	12.3232199	3478.146114
507.1011936	1478.891687	1511.869022	2939.496313	32.29524662	0.873728374
46.88689043	0.391622165	531.2950075	23.94964113	22.18992424	0.001542256
0.001422464	0.00941797	0.204926973	0.721276114	12.3232199	3478.146114
0.881985989	0.027067935	39.49331828	429342.378	0.011355996	0.077360679
10.83050708	42477.74287	13.76429903	68103.17337	10984.57956	0.001691148
10.83050708	42477.74287	13.76429903	68103.17337	10984.57956	0.001691148
68457.92115	2505.973416	28.92950345	64.45951709	1388.585013	0.042603568
507.1011936	1478.891687	1511.869022	2939.496313	32.29524662	0.873728374
46.88689043	0.391622165	531.2950075	23.94964113	22.18992424	0.001542256
0.001422464	0.00941797	0.204926973	0.721276114	12.3232199	3478.146114
0.06370059	42594452.55	2.282674268	0.004855694	8.929688044	81.43019797
0.001422464	0.00941797	0.204926973	0.721276114	12.3232199	3478.146114
10.83050708	42477.74287	13.76429903	68103.17337	10984.57956	0.001691148
25.36093266	0.030713913	5059.993294	0.003630134	333.5791205	24245.75888
2492617.667	0.432218488	0.519781662	0.005046575	48444.92742	0.037759983
3820.009616	7.957270648	1.582118024	34.54294407	0.007834425	98.70656889
507.1011936	1478.891687	1511.869022	2939.496313	32.29524662	0.873728374
0.001422464	0.00941797	0.204926973	0.721276114	12.3232199	3478.146114
0.881985989	0.027067935	39.49331828	429342.378	0.011355996	0.077360679

0.881985989	0.027067935	39.49331828	429342.378	0.011355996	0.077360679
507.1011936	1478.891687	1511.869022	2939.496313	32.29524662	0.873728374
0.001422464	0.00941797	0.204926973	0.721276114	12.3232199	3478.146114
0.06370059	42594452.55	2.282674268	0.004855694	8.929688044	81.43019797
0.001422464	0.00941797	0.204926973	0.721276114	12.3232199	3478.146114
0.001422464	0.00941797	0.204926973	0.721276114	12.3232199	3478.146114
0.881985989	0.027067935	39.49331828	429342.378	0.011355996	0.077360679
2492617.667	0.432218488	0.519781662	0.005046575	48444.92742	0.037759983
507.1011936	1478.891687	1511.869022	2939.496313	32.29524662	0.873728374
25.36093266	0.030713913	5059.993294	0.003630134	333.5791205	24245.75888
53.92016893	54780.47857	2.398800153	0.000251436	1134.11636	7.274232584
0.025054755	62060.76279	108.4091933	1.659943972	0.006841767	0.046608309
0.001422464	0.00941797	0.204926973	0.721276114	12.3232199	3478.146114
0.881985989	0.027067935	39.49331828	429342.378	0.011355996	0.077360679
53.92016893	54780.47857	2.398800153	0.000251436	1134.11636	7.274232584
53684.21014	13.10238807	118785.3992	37718.2922	53.04765393	761212318.6
10.83050708	42477.74287	13.76429903	68103.17337	10984.57956	0.001691148
265.8638962	2.574642456	0.000899196	35.21442232	222.1223505	569.0275115
2492617.667	0.432218488	0.519781662	0.005046575	48444.92742	0.037759983
10.83050708	42477.74287	13.76429903	68103.17337	10984.57956	0.001691148
0.004386538	612.351195	3834.828045	27.88750645	0.673629365	25.66378743
0.000482159	0.002521237	1.55E-05	0.005506384	0.001057752	0.007205744
751.4658587	6.515515762	58.76152474	0.01232318	643.3277344	6658.145091
83.20846796	78551.74488	2.927555361	6.945833152	119.7214744	656.7244025
507.1011936	1478.891687	1511.869022	2939.496313	32.29524662	0.873728374
42.05187837	28157.9058	5.60105163	14.51839423	31.08904201	345.0495564
0.004386538	612.351195	3834.828045	27.88750645	0.673629365	25.66378743
122.6515324	19.5047941	0.94053901	0.006485206	5.827767614	142.178948
2.357992593	0.013488471	0.163257793	13.4799025	2.499363915	26.17785932
0.06370059	42594452.55	2.282674268	0.004855694	8.929688044	81.43019797
25.36093266	0.030713913	5059.993294	0.003630134	333.5791205	24245.75888
122.6515324	19.5047941	0.94053901	0.006485206	5.827767614	142.178948
507.1011936	1478.891687	1511.869022	2939.496313	32.29524662	0.873728374
751.4658587	6.515515762	58.76152474	0.01232318	643.3277344	6658.145091
68457.92115	2505.973416	28.92950345	64.45951709	1388.585013	0.042603568
507.1011936	1478.891687	1511.869022	2939.496313	32.29524662	0.873728374
3820.009616	7.957270648	1.582118024	34.54294407	0.007834425	98.70656889
10.83050708	42477.74287	13.76429903	68103.17337	10984.57956	0.001691148
507.1011936	1478.891687	1511.869022	2939.496313	32.29524662	0.873728374
507.1011936	1478.891687	1511.869022	2939.496313	32.29524662	0.873728374
53684.21014	13.10238807	118785.3992	37718.2922	53.04765393	761212318.6

0.001422464	0.00941797	0.204926973	0.721276114	12.3232199	3478.146114
11.30252243	12861.48096	4.49E-06	1152886.39	14149523.55	1.462553101
507.1011936	1478.891687	1511.869022	2939.496313	32.29524662	0.873728374
0.881985989	0.027067935	39.49331828	429342.378	0.011355996	0.077360679
25.36093266	0.030713913	5059.993294	0.003630134	333.5791205	24245.75888
0.00210413	0.120368286	41.98575599	332885.5239	0.002614266	169.8590328
10.83050708	42477.74287	13.76429903	68103.17337	10984.57956	0.001691148
507.1011936	1478.891687	1511.869022	2939.496313	32.29524662	0.873728374
0.881985989	0.027067935	39.49331828	429342.378	0.011355996	0.077360679
46.88689043	0.391622165	531.2950075	23.94964113	22.18992424	0.001542256
507.1011936	1478.891687	1511.869022	2939.496313	32.29524662	0.873728374
0.001422464	0.00941797	0.204926973	0.721276114	12.3232199	3478.146114
0.007277636	1.909846368	0.943378642	2.382431085	628707.3608	22620.20466
0.881985989	0.027067935	39.49331828	429342.378	0.011355996	0.077360679
507.1011936	1478.891687	1511.869022	2939.496313	32.29524662	0.873728374
507.1011936	1478.891687	1511.869022	2939.496313	32.29524662	0.873728374
46.88689043	0.391622165	531.2950075	23.94964113	22.18992424	0.001542256
0.881985989	0.027067935	39.49331828	429342.378	0.011355996	0.077360679
265.8638962	2.574642456	0.000899196	35.21442232	222.1223505	569.0275115
0.000482159	0.002521237	1.55E-05	0.005506384	0.001057752	0.007205744
751.4658587	6.515515762	58.76152474	0.01232318	643.3277344	6658.145091
507.1011936	1478.891687	1511.869022	2939.496313	32.29524662	0.873728374
507.1011936	1478.891687	1511.869022	2939.496313	32.29524662	0.873728374
2492617.667	0.432218488	0.519781662	0.005046575	48444.92742	0.037759983
0.004386538	612.351195	3834.828045	27.88750645	0.673629365	25.66378743
26.73621697	0.00719895	0.195071634	5952.751845	2.366244668	5.51508649
53.92016893	54780.47857	2.398800153	0.000251436	1134.11636	7.274232584
0.002489037	0.013015307	0.490936354	2.791297441	1002.558859	0.037197998
0.881985989	0.027067935	39.49331828	429342.378	0.011355996	0.077360679
10.83050708	42477.74287	13.76429903	68103.17337	10984.57956	0.001691148
122.6515324	19.5047941	0.94053901	0.006485206	5.827767614	142.178948
0.001422464	0.00941797	0.204926973	0.721276114	12.3232199	3478.146114
391.5511783	119356784.6	280778.3653	69.86173262	201.6378553	0.129436783
507.1011936	1478.891687	1511.869022	2939.496313	32.29524662	0.873728374
0.881985989	0.027067935	39.49331828	429342.378	0.011355996	0.077360679
0.001422464	0.00941797	0.204926973	0.721276114	12.3232199	3478.146114
53.92016893	54780.47857	2.398800153	0.000251436	1134.11636	7.274232584
4.582263427	0.161047566	1.856342814	4.589482063	1.324464207	0.001090377
507.1011936	1478.891687	1511.869022	2939.496313	32.29524662	0.873728374
507.1011936	1478.891687	1511.869022	2939.496313	32.29524662	0.873728374
507.1011936	1478.891687	1511.869022	2939.496313	32.29524662	0.873728374

25.36093266	0.030713913	5059.993294	0.003630134	333.5791205	24245.75888
5737.994112	2783120.43	5.128574608	513876.0139	1303.951778	1759960.698
0.001422464	0.00941797	0.204926973	0.721276114	12.3232199	3478.146114
507.1011936	1478.891687	1511.869022	2939.496313	32.29524662	0.873728374
68457.92115	2505.973416	28.92950345	64.45951709	1388.585013	0.042603568
0.001422464	0.00941797	0.204926973	0.721276114	12.3232199	3478.146114
53.92016893	54780.47857	2.398800153	0.000251436	1134.11636	7.274232584
122.6515324	19.5047941	0.94053901	0.006485206	5.827767614	142.178948
391.5511783	119356784.6	280778.3653	69.86173262	201.6378553	0.129436783
234.174525	19.21560603	0.019944373	167.453655	157.6983767	276.7276739
5737.994112	2783120.43	5.128574608	513876.0139	1303.951778	1759960.698
5737.994112	2783120.43	5.128574608	513876.0139	1303.951778	1759960.698
53.92016893	54780.47857	2.398800153	0.000251436	1134.11636	7.274232584
25.36093266	0.030713913	5059.993294	0.003630134	333.5791205	24245.75888
507.1011936	1478.891687	1511.869022	2939.496313	32.29524662	0.873728374
0.881985989	0.027067935	39.49331828	429342.378	0.011355996	0.077360679
507.1011936	1478.891687	1511.869022	2939.496313	32.29524662	0.873728374
0.881985989	0.027067935	39.49331828	429342.378	0.011355996	0.077360679
0.001422464	0.00941797	0.204926973	0.721276114	12.3232199	3478.146114
0.00210413	0.120368286	41.98575599	332885.5239	0.002614266	169.8590328
265.8638962	2.574642456	0.000899196	35.21442232	222.1223505	569.0275115
116.8592582	8047.583317	8929.399901	8.1119557	0.000856053	0.00583171
0.881985989	0.027067935	39.49331828	429342.378	0.011355996	0.077360679
0.881985989	0.027067935	39.49331828	429342.378	0.011355996	0.077360679
5737.994112	2783120.43	5.128574608	513876.0139	1303.951778	1759960.698
42.05187837	28157.9058	5.60105163	14.51839423	31.08904201	345.0495564
0.881985989	0.027067935	39.49331828	429342.378	0.011355996	0.077360679
0.881985989	0.027067935	39.49331828	429342.378	0.011355996	0.077360679
2492617.667	0.432218488	0.519781662	0.005046575	48444.92742	0.037759983
4119.001973	12.96182847	97978.19598	3026.535321	0.509946024	266.4579467
2492617.667	0.432218488	0.519781662	0.005046575	48444.92742	0.037759983
2492617.667	0.432218488	0.519781662	0.005046575	48444.92742	0.037759983
0.001422464	0.00941797	0.204926973	0.721276114	12.3232199	3478.146114
0.004386538	612.351195	3834.828045	27.88750645	0.673629365	25.66378743
53.92016893	54780.47857	2.398800153	0.000251436	1134.11636	7.274232584
0.881985989	0.027067935	39.49331828	429342.378	0.011355996	0.077360679
751.4658587	6.515515762	58.76152474	0.01232318	643.3277344	6658.145091
68457.92115	2505.973416	28.92950345	64.45951709	1388.585013	0.042603568
122.6515324	19.5047941	0.94053901	0.006485206	5.827767614	142.178948
0.881985989	0.027067935	39.49331828	429342.378	0.011355996	0.077360679
507.1011936	1478.891687	1511.869022	2939.496313	32.29524662	0.873728374

5737.994112	2783120.43	5.128574608	513876.0139	1303.951778	1759960.698
0.881985989	0.027067935	39.49331828	429342.378	0.011355996	0.077360679
0.004386538	612.351195	3834.828045	27.88750645	0.673629365	25.66378743
4.582263427	0.161047566	1.856342814	4.589482063	1.324464207	0.001090377
0.001422464	0.00941797	0.204926973	0.721276114	12.3232199	3478.146114
25.36093266	0.030713913	5059.993294	0.003630134	333.5791205	24245.75888
5737.994112	2783120.43	5.128574608	513876.0139	1303.951778	1759960.698
68457.92115	2505.973416	28.92950345	64.45951709	1388.585013	0.042603568
42.05187837	28157.9058	5.60105163	14.51839423	31.08904201	345.0495564
42.05187837	28157.9058	5.60105163	14.51839423	31.08904201	345.0495564
0.00210413	0.120368286	41.98575599	332885.5239	0.002614266	169.8590328
2492617.667	0.432218488	0.519781662	0.005046575	48444.92742	0.037759983
0.001422464	0.00941797	0.204926973	0.721276114	12.3232199	3478.146114
4119.001973	12.96182847	97978.19598	3026.535321	0.509946024	266.4579467
53.92016893	54780.47857	2.398800153	0.000251436	1134.11636	7.274232584
3820.009616	7.957270648	1.582118024	34.54294407	0.007834425	98.70656889
53.92016893	54780.47857	2.398800153	0.000251436	1134.11636	7.274232584
122.6515324	19.5047941	0.94053901	0.006485206	5.827767614	142.178948
507.1011936	1478.891687	1511.869022	2939.496313	32.29524662	0.873728374
68457.92115	2505.973416	28.92950345	64.45951709	1388.585013	0.042603568
83.20846796	78551.74488	2.927555361	6.945833152	119.7214744	656.7244025
0.001422464	0.00941797	0.204926973	0.721276114	12.3232199	3478.146114
68457.92115	2505.973416	28.92950345	64.45951709	1388.585013	0.042603568
0.000482159	0.002521237	1.55E-05	0.005506384	0.001057752	0.007205744
507.1011936	1478.891687	1511.869022	2939.496313	32.29524662	0.873728374
25.36093266	0.030713913	5059.993294	0.003630134	333.5791205	24245.75888
46.88689043	0.391622165	531.2950075	23.94964113	22.18992424	0.001542256
0.881985989	0.027067935	39.49331828	429342.378	0.011355996	0.077360679
122.6515324	19.5047941	0.94053901	0.006485206	5.827767614	142.178948
793236.9018	19681.0082	12741.86914	140.6064572	2.851419285	1.100140828
2492617.667	0.432218488	0.519781662	0.005046575	48444.92742	0.037759983
10.83050708	42477.74287	13.76429903	68103.17337	10984.57956	0.001691148
3820.009616	7.957270648	1.582118024	34.54294407	0.007834425	98.70656889
0.001422464	0.00941797	0.204926973	0.721276114	12.3232199	3478.146114
0.025054755	62060.76279	108.4091933	1.659943972	0.006841767	0.046608309
122.6515324	19.5047941	0.94053901	0.006485206	5.827767614	142.178948
0.881985989	0.027067935	39.49331828	429342.378	0.011355996	0.077360679
507.1011936	1478.891687	1511.869022	2939.496313	32.29524662	0.873728374
122.6515324	19.5047941	0.94053901	0.006485206	5.827767614	142.178948
507.1011936	1478.891687	1511.869022	2939.496313	32.29524662	0.873728374
0.00210413	0.120368286	41.98575599	332885.5239	0.002614266	169.8590328

265.8638962	2.574642456	0.000899196	35.21442232	222.1223505	569.0275115
53.92016893	54780.47857	2.398800153	0.000251436	1134.11636	7.274232584
4119.001973	12.96182847	97978.19598	3026.535321	0.509946024	266.4579467
122.6515324	19.5047941	0.94053901	0.006485206	5.827767614	142.178948
25.36093266	0.030713913	5059.993294	0.003630134	333.5791205	24245.75888
5737.994112	2783120.43	5.128574608	513876.0139	1303.951778	1759960.698
507.1011936	1478.891687	1511.869022	2939.496313	32.29524662	0.873728374
0.001422464	0.00941797	0.204926973	0.721276114	12.3232199	3478.146114
0.001422464	0.00941797	0.204926973	0.721276114	12.3232199	3478.146114
11.30252243	12861.48096	4.49E-06	1152886.39	14149523.55	1.462553101
2.357992593	0.013488471	0.163257793	13.4799025	2.499363915	26.17785932
122.6515324	19.5047941	0.94053901	0.006485206	5.827767614	142.178948
507.1011936	1478.891687	1511.869022	2939.496313	32.29524662	0.873728374
0.881985989	0.027067935	39.49331828	429342.378	0.011355996	0.077360679
0.001422464	0.00941797	0.204926973	0.721276114	12.3232199	3478.146114
0.004386538	612.351195	3834.828045	27.88750645	0.673629365	25.66378743
25.36093266	0.030713913	5059.993294	0.003630134	333.5791205	24245.75888
0.001422464	0.00941797	0.204926973	0.721276114	12.3232199	3478.146114
11.30252243	12861.48096	4.49E-06	1152886.39	14149523.55	1.462553101
10.83050708	42477.74287	13.76429903	68103.17337	10984.57956	0.001691148
0.001422464	0.00941797	0.204926973	0.721276114	12.3232199	3478.146114
53.92016893	54780.47857	2.398800153	0.000251436	1134.11636	7.274232584
122.6515324	19.5047941	0.94053901	0.006485206	5.827767614	142.178948
122.6515324	19.5047941	0.94053901	0.006485206	5.827767614	142.178948
0.025054755	62060.76279	108.4091933	1.659943972	0.006841767	0.046608309
4119.001973	12.96182847	97978.19598	3026.535321	0.509946024	266.4579467
4119.001973	12.96182847	97978.19598	3026.535321	0.509946024	266.4579467
122.6515324	19.5047941	0.94053901	0.006485206	5.827767614	142.178948
246.9539207	3128.825214	3320.500866	1022.604151	660.0289615	3.439133266
0.00210413	0.120368286	41.98575599	332885.5239	0.002614266	169.8590328
507.1011936	1478.891687	1511.869022	2939.496313	32.29524662	0.873728374
68457.92115	2505.973416	28.92950345	64.45951709	1388.585013	0.042603568
26.73621697	0.00719895	0.195071634	5952.751845	2.366244668	5.51508649
0.06370059	42594452.55	2.282674268	0.004855694	8.929688044	81.43019797
11.30252243	12861.48096	4.49E-06	1152886.39	14149523.55	1.462553101
26.73621697	0.00719895	0.195071634	5952.751845	2.366244668	5.51508649
0.001422464	0.00941797	0.204926973	0.721276114	12.3232199	3478.146114
0.002489037	0.013015307	0.490936354	2.791297441	1002.558859	0.037197998
507.1011936	1478.891687	1511.869022	2939.496313	32.29524662	0.873728374
0.002489037	0.013015307	0.490936354	2.791297441	1002.558859	0.037197998
507.1011936	1478.891687	1511.869022	2939.496313	32.29524662	0.873728374

0.001422464	0.00941797	0.204926973	0.721276114	12.3232199	3478.146114
0.001422464	0.00941797	0.204926973	0.721276114	12.3232199	3478.146114
507.1011936	1478.891687	1511.869022	2939.496313	32.29524662	0.873728374
0.001422464	0.00941797	0.204926973	0.721276114	12.3232199	3478.146114
3820.009616	7.957270648	1.582118024	34.54294407	0.007834425	98.70656889
507.1011936	1478.891687	1511.869022	2939.496313	32.29524662	0.873728374
507.1011936	1478.891687	1511.869022	2939.496313	32.29524662	0.873728374
116.8592582	8047.583317	8929.399901	8.1119557	0.000856053	0.00583171
234.174525	19.21560603	0.019944373	167.453655	157.6983767	276.7276739
0.007277636	1.909846368	0.943378642	2.382431085	628707.3608	22620.20466
2.357992593	0.013488471	0.163257793	13.4799025	2.499363915	26.17785932
10.83050708	42477.74287	13.76429903	68103.17337	10984.57956	0.001691148
0.00253959	292.6377976	5956.773245	0.105152004	10617393.69	1.849463033
83.20846796	78551.74488	2.927555361	6.945833152	119.7214744	656.7244025
246.9539207	3128.825214	3320.500866	1022.604151	660.0289615	3.439133266
122.6515324	19.5047941	0.94053901	0.006485206	5.827767614	142.178948
53.92016893	54780.47857	2.398800153	0.000251436	1134.11636	7.274232584
0.001422464	0.00941797	0.204926973	0.721276114	12.3232199	3478.146114
68457.92115	2505.973416	28.92950345	64.45951709	1388.585013	0.042603568
42.05187837	28157.9058	5.60105163	14.51839423	31.08904201	345.0495564
0.001422464	0.00941797	0.204926973	0.721276114	12.3232199	3478.146114
3820.009616	7.957270648	1.582118024	34.54294407	0.007834425	98.70656889
2492617.667	0.432218488	0.519781662	0.005046575	48444.92742	0.037759983
0.001422464	0.00941797	0.204926973	0.721276114	12.3232199	3478.146114
507.1011936	1478.891687	1511.869022	2939.496313	32.29524662	0.873728374
42.05187837	28157.9058	5.60105163	14.51839423	31.08904201	345.0495564
0.881985989	0.027067935	39.49331828	429342.378	0.011355996	0.077360679
246.9539207	3128.825214	3320.500866	1022.604151	660.0289615	3.439133266
0.00253959	292.6377976	5956.773245	0.105152004	10617393.69	1.849463033
68457.92115	2505.973416	28.92950345	64.45951709	1388.585013	0.042603568
122.6515324	19.5047941	0.94053901	0.006485206	5.827767614	142.178948
2492617.667	0.432218488	0.519781662	0.005046575	48444.92742	0.037759983
25.36093266	0.030713913	5059.993294	0.003630134	333.5791205	24245.75888
3820.009616	7.957270648	1.582118024	34.54294407	0.007834425	98.70656889
53.92016893	54780.47857	2.398800153	0.000251436	1134.11636	7.274232584
0.001422464	0.00941797	0.204926973	0.721276114	12.3232199	3478.146114
122.6515324	19.5047941	0.94053901	0.006485206	5.827767614	142.178948
10.83050708	42477.74287	13.76429903	68103.17337	10984.57956	0.001691148
2492617.667	0.432218488	0.519781662	0.005046575	48444.92742	0.037759983
0.881985989	0.027067935	39.49331828	429342.378	0.011355996	0.077360679
0.00210413	0.120368286	41.98575599	332885.5239	0.002614266	169.8590328

0.004386538	612.351195	3834.828045	27.88750645	0.673629365	25.66378743
507.1011936	1478.891687	1511.869022	2939.496313	32.29524662	0.873728374
507.1011936	1478.891687	1511.869022	2939.496313	32.29524662	0.873728374
4119.001973	12.96182847	97978.19598	3026.535321	0.509946024	266.4579467
0.881985989	0.027067935	39.49331828	429342.378	0.011355996	0.077360679
0.025054755	62060.76279	108.4091933	1.659943972	0.006841767	0.046608309
0.001422464	0.00941797	0.204926973	0.721276114	12.3232199	3478.146114
0.001422464	0.00941797	0.204926973	0.721276114	12.3232199	3478.146114
751.4658587	6.515515762	58.76152474	0.01232318	643.3277344	6658.145091
507.1011936	1478.891687	1511.869022	2939.496313	32.29524662	0.873728374
0.001422464	0.00941797	0.204926973	0.721276114	12.3232199	3478.146114
122.6515324	19.5047941	0.94053901	0.006485206	5.827767614	142.178948
68457.92115	2505.973416	28.92950345	64.45951709	1388.585013	0.042603568
2492617.667	0.432218488	0.519781662	0.005046575	48444.92742	0.037759983
0.001422464	0.00941797	0.204926973	0.721276114	12.3232199	3478.146114
0.881985989	0.027067935	39.49331828	429342.378	0.011355996	0.077360679
0.001422464	0.00941797	0.204926973	0.721276114	12.3232199	3478.146114
2492617.667	0.432218488	0.519781662	0.005046575	48444.92742	0.037759983
0.002489037	0.013015307	0.490936354	2.791297441	1002.558859	0.037197998
122.6515324	19.5047941	0.94053901	0.006485206	5.827767614	142.178948
68457.92115	2505.973416	28.92950345	64.45951709	1388.585013	0.042603568
116.8592582	8047.583317	8929.399901	8.1119557	0.000856053	0.00583171
53.92016893	54780.47857	2.398800153	0.000251436	1134.11636	7.274232584
0.881985989	0.027067935	39.49331828	429342.378	0.011355996	0.077360679
2492617.667	0.432218488	0.519781662	0.005046575	48444.92742	0.037759983
303.7647121	0.040367691	88825.40181	31.83876004	65.96527827	23.08359483
42.05187837	28157.9058	5.60105163	14.51839423	31.08904201	345.0495564
0.001422464	0.00941797	0.204926973	0.721276114	12.3232199	3478.146114
0.881985989	0.027067935	39.49331828	429342.378	0.011355996	0.077360679
5737.994112	2783120.43	5.128574608	513876.0139	1303.951778	1759960.698
507.1011936	1478.891687	1511.869022	2939.496313	32.29524662	0.873728374
0.001422464	0.00941797	0.204926973	0.721276114	12.3232199	3478.146114
234.174525	19.21560603	0.019944373	167.453655	157.6983767	276.7276739
0.001422464	0.00941797	0.204926973	0.721276114	12.3232199	3478.146114
25.36093266	0.030713913	5059.993294	0.003630134	333.5791205	24245.75888
2492617.667	0.432218488	0.519781662	0.005046575	48444.92742	0.037759983
0.007277636	1.909846368	0.943378642	2.382431085	628707.3608	22620.20466
0.00210413	0.120368286	41.98575599	332885.5239	0.002614266	169.8590328
4119.001973	12.96182847	97978.19598	3026.535321	0.509946024	266.4579467
0.00210413	0.120368286	41.98575599	332885.5239	0.002614266	169.8590328
46.88689043	0.391622165	531.2950075	23.94964113	22.18992424	0.001542256

0.881985989	0.027067935	39.49331828	429342.378	0.011355996	0.077360679
2492617.667	0.432218488	0.519781662	0.005046575	48444.92742	0.037759983
0.00210413	0.120368286	41.98575599	332885.5239	0.002614266	169.8590328
5737.994112	2783120.43	5.128574608	513876.0139	1303.951778	1759960.698
0.881985989	0.027067935	39.49331828	429342.378	0.011355996	0.077360679
0.001422464	0.00941797	0.204926973	0.721276114	12.3232199	3478.146114
83.20846796	78551.74488	2.927555361	6.945833152	119.7214744	656.7244025
25.36093266	0.030713913	5059.993294	0.003630134	333.5791205	24245.75888
5737.994112	2783120.43	5.128574608	513876.0139	1303.951778	1759960.698
507.1011936	1478.891687	1511.869022	2939.496313	32.29524662	0.873728374
25.36093266	0.030713913	5059.993294	0.003630134	333.5791205	24245.75888
25.36093266	0.030713913	5059.993294	0.003630134	333.5791205	24245.75888
122.6515324	19.5047941	0.94053901	0.006485206	5.827767614	142.178948
39.78483772	345311.1709	7.712725298	0.004494878	354.6221056	360.4606496
0.001422464	0.00941797	0.204926973	0.721276114	12.3232199	3478.146114
751.4658587	6.515515762	58.76152474	0.01232318	643.3277344	6658.145091
0.001422464	0.00941797	0.204926973	0.721276114	12.3232199	3478.146114
0.001422464	0.00941797	0.204926973	0.721276114	12.3232199	3478.146114
507.1011936	1478.891687	1511.869022	2939.496313	32.29524662	0.873728374
0.001422464	0.00941797	0.204926973	0.721276114	12.3232199	3478.146114
265.8638962	2.574642456	0.000899196	35.21442232	222.1223505	569.0275115
83.20846796	78551.74488	2.927555361	6.945833152	119.7214744	656.7244025
507.1011936	1478.891687	1511.869022	2939.496313	32.29524662	0.873728374
68457.92115	2505.973416	28.92950345	64.45951709	1388.585013	0.042603568
0.000482159	0.002521237	1.55E-05	0.005506384	0.001057752	0.007205744
0.881985989	0.027067935	39.49331828	429342.378	0.011355996	0.077360679
53.92016893	54780.47857	2.398800153	0.000251436	1134.11636	7.274232584
0.001422464	0.00941797	0.204926973	0.721276114	12.3232199	3478.146114
507.1011936	1478.891687	1511.869022	2939.496313	32.29524662	0.873728374
5737.994112	2783120.43	5.128574608	513876.0139	1303.951778	1759960.698
507.1011936	1478.891687	1511.869022	2939.496313	32.29524662	0.873728374
39.78483772	345311.1709	7.712725298	0.004494878	354.6221056	360.4606496
53684.21014	13.10238807	118785.3992	37718.2922	53.04765393	761212318.6
0.001422464	0.00941797	0.204926973	0.721276114	12.3232199	3478.146114
0.881985989	0.027067935	39.49331828	429342.378	0.011355996	0.077360679
0.881985989	0.027067935	39.49331828	429342.378	0.011355996	0.077360679
53.92016893	54780.47857	2.398800153	0.000251436	1134.11636	7.274232584
68457.92115	2505.973416	28.92950345	64.45951709	1388.585013	0.042603568
0.001422464	0.00941797	0.204926973	0.721276114	12.3232199	3478.146114
751.4658587	6.515515762	58.76152474	0.01232318	643.3277344	6658.145091
507.1011936	1478.891687	1511.869022	2939.496313	32.29524662	0.873728374

0.881985989	0.027067935	39.49331828	429342.378	0.011355996	0.077360679
2492617.667	0.432218488	0.519781662	0.005046575	48444.92742	0.037759983
4119.001973	12.96182847	97978.19598	3026.535321	0.509946024	266.4579467
25.36093266	0.030713913	5059.993294	0.003630134	333.5791205	24245.75888
0.881985989	0.027067935	39.49331828	429342.378	0.011355996	0.077360679
53.92016893	54780.47857	2.398800153	0.000251436	1134.11636	7.274232584
751.4658587	6.515515762	58.76152474	0.01232318	643.3277344	6658.145091
0.001422464	0.00941797	0.204926973	0.721276114	12.3232199	3478.146114
5737.994112	2783120.43	5.128574608	513876.0139	1303.951778	1759960.698
0.881985989	0.027067935	39.49331828	429342.378	0.011355996	0.077360679
0.001422464	0.00941797	0.204926973	0.721276114	12.3232199	3478.146114
0.001422464	0.00941797	0.204926973	0.721276114	12.3232199	3478.146114
507.1011936	1478.891687	1511.869022	2939.496313	32.29524662	0.873728374
0.881985989	0.027067935	39.49331828	429342.378	0.011355996	0.077360679
2492617.667	0.432218488	0.519781662	0.005046575	48444.92742	0.037759983
0.001422464	0.00941797	0.204926973	0.721276114	12.3232199	3478.146114
2492617.667	0.432218488	0.519781662	0.005046575	48444.92742	0.037759983
5737.994112	2783120.43	5.128574608	513876.0139	1303.951778	1759960.698
3820.009616	7.957270648	1.582118024	34.54294407	0.007834425	98.70656889
5737.994112	2783120.43	5.128574608	513876.0139	1303.951778	1759960.698
0.881985989	0.027067935	39.49331828	429342.378	0.011355996	0.077360679
5737.994112	2783120.43	5.128574608	513876.0139	1303.951778	1759960.698
507.1011936	1478.891687	1511.869022	2939.496313	32.29524662	0.873728374
53684.21014	13.10238807	118785.3992	37718.2922	53.04765393	761212318.6
0.002489037	0.013015307	0.490936354	2.791297441	1002.558859	0.037197998
0.001422464	0.00941797	0.204926973	0.721276114	12.3232199	3478.146114
122.6515324	19.5047941	0.94053901	0.006485206	5.827767614	142.178948
2492617.667	0.432218488	0.519781662	0.005046575	48444.92742	0.037759983
4119.001973	12.96182847	97978.19598	3026.535321	0.509946024	266.4579467
2492617.667	0.432218488	0.519781662	0.005046575	48444.92742	0.037759983
0.881985989	0.027067935	39.49331828	429342.378	0.011355996	0.077360679
25.36093266	0.030713913	5059.993294	0.003630134	333.5791205	24245.75888
53684.21014	13.10238807	118785.3992	37718.2922	53.04765393	761212318.6
4119.001973	12.96182847	97978.19598	3026.535321	0.509946024	266.4579467
53.92016893	54780.47857	2.398800153	0.000251436	1134.11636	7.274232584
122.6515324	19.5047941	0.94053901	0.006485206	5.827767614	142.178948
53.92016893	54780.47857	2.398800153	0.000251436	1134.11636	7.274232584
0.881985989	0.027067935	39.49331828	429342.378	0.011355996	0.077360679
46.88689043	0.391622165	531.2950075	23.94964113	22.18992424	0.001542256
0.001422464	0.00941797	0.204926973	0.721276114	12.3232199	3478.146114
0.881985989	0.027067935	39.49331828	429342.378	0.011355996	0.077360679

122.6515324	19.5047941	0.94053901	0.006485206	5.827767614	142.178948
0.001422464	0.00941797	0.204926973	0.721276114	12.3232199	3478.146114
507.1011936	1478.891687	1511.869022	2939.496313	32.29524662	0.873728374
0.000482159	0.002521237	1.55E-05	0.005506384	0.001057752	0.007205744
507.1011936	1478.891687	1511.869022	2939.496313	32.29524662	0.873728374
507.1011936	1478.891687	1511.869022	2939.496313	32.29524662	0.873728374
0.001422464	0.00941797	0.204926973	0.721276114	12.3232199	3478.146114
122.6515324	19.5047941	0.94053901	0.006485206	5.827767614	142.178948
53.92016893	54780.47857	2.398800153	0.000251436	1134.11636	7.274232584
0.881985989	0.027067935	39.49331828	429342.378	0.011355996	0.077360679
0.001422464	0.00941797	0.204926973	0.721276114	12.3232199	3478.146114
0.881985989	0.027067935	39.49331828	429342.378	0.011355996	0.077360679
39.78483772	345311.1709	7.712725298	0.004494878	354.6221056	360.4606496
507.1011936	1478.891687	1511.869022	2939.496313	32.29524662	0.873728374
507.1011936	1478.891687	1511.869022	2939.496313	32.29524662	0.873728374
4119.001973	12.96182847	97978.19598	3026.535321	0.509946024	266.4579467
0.004386538	612.351195	3834.828045	27.88750645	0.673629365	25.66378743
53.92016893	54780.47857	2.398800153	0.000251436	1134.11636	7.274232584
2492617.667	0.432218488	0.519781662	0.005046575	48444.92742	0.037759983
2.357992593	0.013488471	0.163257793	13.4799025	2.499363915	26.17785932
0.881985989	0.027067935	39.49331828	429342.378	0.011355996	0.077360679
46.88689043	0.391622165	531.2950075	23.94964113	22.18992424	0.001542256
25.36093266	0.030713913	5059.993294	0.003630134	333.5791205	24245.75888
10.83050708	42477.74287	13.76429903	68103.17337	10984.57956	0.001691148
5737.994112	2783120.43	5.128574608	513876.0139	1303.951778	1759960.698
0.000482159	0.002521237	1.55E-05	0.005506384	0.001057752	0.007205744
303.7647121	0.040367691	88825.40181	31.83876004	65.96527827	23.08359483
42.05187837	28157.9058	5.60105163	14.51839423	31.08904201	345.0495564
0.00253959	292.6377976	5956.773245	0.105152004	10617393.69	1.849463033
0.881985989	0.027067935	39.49331828	429342.378	0.011355996	0.077360679
53.92016893	54780.47857	2.398800153	0.000251436	1134.11636	7.274232584
122.6515324	19.5047941	0.94053901	0.006485206	5.827767614	142.178948
793236.9018	19681.0082	12741.86914	140.6064572	2.851419285	1.100140828
507.1011936	1478.891687	1511.869022	2939.496313	32.29524662	0.873728374
0.001422464	0.00941797	0.204926973	0.721276114	12.3232199	3478.146114
0.00253959	292.6377976	5956.773245	0.105152004	10617393.69	1.849463033
10.83050708	42477.74287	13.76429903	68103.17337	10984.57956	0.001691148
507.1011936	1478.891687	1511.869022	2939.496313	32.29524662	0.873728374
507.1011936	1478.891687	1511.869022	2939.496313	32.29524662	0.873728374
2.357992593	0.013488471	0.163257793	13.4799025	2.499363915	26.17785932
0.00210413	0.120368286	41.98575599	332885.5239	0.002614266	169.8590328

751.4658587	6.515515762	58.76152474	0.01232318	643.3277344	6658.145091
507.1011936	1478.891687	1511.869022	2939.496313	32.29524662	0.873728374
246.9539207	3128.825214	3320.500866	1022.604151	660.0289615	3.439133266
507.1011936	1478.891687	1511.869022	2939.496313	32.29524662	0.873728374
0.001422464	0.00941797	0.204926973	0.721276114	12.3232199	3478.146114
0.00210413	0.120368286	41.98575599	332885.5239	0.002614266	169.8590328
122.6515324	19.5047941	0.94053901	0.006485206	5.827767614	142.178948
507.1011936	1478.891687	1511.869022	2939.496313	32.29524662	0.873728374
0.00210413	0.120368286	41.98575599	332885.5239	0.002614266	169.8590328
25.36093266	0.030713913	5059.993294	0.003630134	333.5791205	24245.75888
507.1011936	1478.891687	1511.869022	2939.496313	32.29524662	0.873728374
507.1011936	1478.891687	1511.869022	2939.496313	32.29524662	0.873728374
0.002489037	0.013015307	0.490936354	2.791297441	1002.558859	0.037197998
0.881985989	0.027067935	39.49331828	429342.378	0.011355996	0.077360679
751.4658587	6.515515762	58.76152474	0.01232318	643.3277344	6658.145091
0.881985989	0.027067935	39.49331828	429342.378	0.011355996	0.077360679
507.1011936	1478.891687	1511.869022	2939.496313	32.29524662	0.873728374
122.6515324	19.5047941	0.94053901	0.006485206	5.827767614	142.178948
46.88689043	0.391622165	531.2950075	23.94964113	22.18992424	0.001542256
0.001422464	0.00941797	0.204926973	0.721276114	12.3232199	3478.146114
0.000482159	0.002521237	1.55E-05	0.005506384	0.001057752	0.007205744
4119.001973	12.96182847	97978.19598	3026.535321	0.509946024	266.4579467
0.881985989	0.027067935	39.49331828	429342.378	0.011355996	0.077360679
122.6515324	19.5047941	0.94053901	0.006485206	5.827767614	142.178948
507.1011936	1478.891687	1511.869022	2939.496313	32.29524662	0.873728374
507.1011936	1478.891687	1511.869022	2939.496313	32.29524662	0.873728374
0.001422464	0.00941797	0.204926973	0.721276114	12.3232199	3478.146114
0.001422464	0.00941797	0.204926973	0.721276114	12.3232199	3478.146114
0.001422464	0.00941797	0.204926973	0.721276114	12.3232199	3478.146114
5737.994112	2783120.43	5.128574608	513876.0139	1303.951778	1759960.698
0.001422464	0.00941797	0.204926973	0.721276114	12.3232199	3478.146114
4119.001973	12.96182847	97978.19598	3026.535321	0.509946024	266.4579467
507.1011936	1478.891687	1511.869022	2939.496313	32.29524662	0.873728374
507.1011936	1478.891687	1511.869022	2939.496313	32.29524662	0.873728374
0.000482159	0.002521237	1.55E-05	0.005506384	0.001057752	0.007205744
10.83050708	42477.74287	13.76429903	68103.17337	10984.57956	0.001691148
42.05187837	28157.9058	5.60105163	14.51839423	31.08904201	345.0495564
26.73621697	0.00719895	0.195071634	5952.751845	2.366244668	5.51508649
83.20846796	78551.74488	2.927555361	6.945833152	119.7214744	656.7244025
0.00210413	0.120368286	41.98575599	332885.5239	0.002614266	169.8590328
0.001422464	0.00941797	0.204926973	0.721276114	12.3232199	3478.146114

4119.001973	12.96182847	97978.19598	3026.535321	0.509946024	266.4579467
25.36093266	0.030713913	5059.993294	0.003630134	333.5791205	24245.75888
25.36093266	0.030713913	5059.993294	0.003630134	333.5791205	24245.75888
25.36093266	0.030713913	5059.993294	0.003630134	333.5791205	24245.75888
507.1011936	1478.891687	1511.869022	2939.496313	32.29524662	0.873728374
0.001422464	0.00941797	0.204926973	0.721276114	12.3232199	3478.146114
507.1011936	1478.891687	1511.869022	2939.496313	32.29524662	0.873728374
0.881985989	0.027067935	39.49331828	429342.378	0.011355996	0.077360679
0.00210413	0.120368286	41.98575599	332885.5239	0.002614266	169.8590328
0.881985989	0.027067935	39.49331828	429342.378	0.011355996	0.077360679
83.20846796	78551.74488	2.927555361	6.945833152	119.7214744	656.7244025
0.001422464	0.00941797	0.204926973	0.721276114	12.3232199	3478.146114
507.1011936	1478.891687	1511.869022	2939.496313	32.29524662	0.873728374
2492617.667	0.432218488	0.519781662	0.005046575	48444.92742	0.037759983
0.881985989	0.027067935	39.49331828	429342.378	0.011355996	0.077360679
11.30252243	12861.48096	4.49E-06	1152886.39	14149523.55	1.462553101
0.001422464	0.00941797	0.204926973	0.721276114	12.3232199	3478.146114
0.001422464	0.00941797	0.204926973	0.721276114	12.3232199	3478.146114
2492617.667	0.432218488	0.519781662	0.005046575	48444.92742	0.037759983
46.88689043	0.391622165	531.2950075	23.94964113	22.18992424	0.001542256
0.001422464	0.00941797	0.204926973	0.721276114	12.3232199	3478.146114
793236.9018	19681.0082	12741.86914	140.6064572	2.851419285	1.100140828
0.881985989	0.027067935	39.49331828	429342.378	0.011355996	0.077360679
53.92016893	54780.47857	2.398800153	0.000251436	1134.11636	7.274232584
2492617.667	0.432218488	0.519781662	0.005046575	48444.92742	0.037759983
0.002489037	0.013015307	0.490936354	2.791297441	1002.558859	0.037197998
0.001422464	0.00941797	0.204926973	0.721276114	12.3232199	3478.146114
3820.009616	7.957270648	1.582118024	34.54294407	0.007834425	98.70656889
0.004386538	612.351195	3834.828045	27.88750645	0.673629365	25.66378743
0.881985989	0.027067935	39.49331828	429342.378	0.011355996	0.077360679
0.00210413	0.120368286	41.98575599	332885.5239	0.002614266	169.8590328
25.36093266	0.030713913	5059.993294	0.003630134	333.5791205	24245.75888
0.881985989	0.027067935	39.49331828	429342.378	0.011355996	0.077360679
0.001422464	0.00941797	0.204926973	0.721276114	12.3232199	3478.146114
0.025054755	62060.76279	108.4091933	1.659943972	0.006841767	0.046608309
507.1011936	1478.891687	1511.869022	2939.496313	32.29524662	0.873728374
751.4658587	6.515515762	58.76152474	0.01232318	643.3277344	6658.145091
0.007277636	1.909846368	0.943378642	2.382431085	628707.3608	22620.20466
0.001422464	0.00941797	0.204926973	0.721276114	12.3232199	3478.146114
507.1011936	1478.891687	1511.869022	2939.496313	32.29524662	0.873728374
4119.001973	12.96182847	97978.19598	3026.535321	0.509946024	266.4579467

0.001422464	0.00941797	0.204926973	0.721276114	12.3232199	3478.146114
0.06370059	42594452.55	2.282674268	0.004855694	8.929688044	81.43019797
53.92016893	54780.47857	2.398800153	0.000251436	1134.11636	7.274232584
507.1011936	1478.891687	1511.869022	2939.496313	32.29524662	0.873728374
0.001422464	0.00941797	0.204926973	0.721276114	12.3232199	3478.146114
0.881985989	0.027067935	39.49331828	429342.378	0.011355996	0.077360679
0.881985989	0.027067935	39.49331828	429342.378	0.011355996	0.077360679
0.881985989	0.027067935	39.49331828	429342.378	0.011355996	0.077360679
0.001422464	0.00941797	0.204926973	0.721276114	12.3232199	3478.146114
507.1011936	1478.891687	1511.869022	2939.496313	32.29524662	0.873728374
0.001422464	0.00941797	0.204926973	0.721276114	12.3232199	3478.146114
0.001422464	0.00941797	0.204926973	0.721276114	12.3232199	3478.146114
507.1011936	1478.891687	1511.869022	2939.496313	32.29524662	0.873728374
11.30252243	12861.48096	4.49E-06	1152886.39	14149523.55	1.462553101
0.025054755	62060.76279	108.4091933	1.659943972	0.006841767	0.046608309
0.881985989	0.027067935	39.49331828	429342.378	0.011355996	0.077360679
0.001422464	0.00941797	0.204926973	0.721276114	12.3232199	3478.146114
507.1011936	1478.891687	1511.869022	2939.496313	32.29524662	0.873728374
507.1011936	1478.891687	1511.869022	2939.496313	32.29524662	0.873728374
46.88689043	0.391622165	531.2950075	23.94964113	22.18992424	0.001542256
507.1011936	1478.891687	1511.869022	2939.496313	32.29524662	0.873728374
68457.92115	2505.973416	28.92950345	64.45951709	1388.585013	0.042603568
83.20846796	78551.74488	2.927555361	6.945833152	119.7214744	656.7244025
0.001422464	0.00941797	0.204926973	0.721276114	12.3232199	3478.146114
0.00210413	0.120368286	41.98575599	332885.5239	0.002614266	169.8590328
0.001422464	0.00941797	0.204926973	0.721276114	12.3232199	3478.146114
0.001422464	0.00941797	0.204926973	0.721276114	12.3232199	3478.146114
303.7647121	0.040367691	88825.40181	31.83876004	65.96527827	23.08359483
4119.001973	12.96182847	97978.19598	3026.535321	0.509946024	266.4579467
25.36093266	0.030713913	5059.993294	0.003630134	333.5791205	24245.75888
507.1011936	1478.891687	1511.869022	2939.496313	32.29524662	0.873728374
0.004386538	612.351195	3834.828045	27.88750645	0.673629365	25.66378743
3820.009616	7.957270648	1.582118024	34.54294407	0.007834425	98.70656889
10.83050708	42477.74287	13.76429903	68103.17337	10984.57956	0.001691148
0.881985989	0.027067935	39.49331828	429342.378	0.011355996	0.077360679
507.1011936	1478.891687	1511.869022	2939.496313	32.29524662	0.873728374
0.881985989	0.027067935	39.49331828	429342.378	0.011355996	0.077360679
0.06370059	42594452.55	2.282674268	0.004855694	8.929688044	81.43019797
68457.92115	2505.973416	28.92950345	64.45951709	1388.585013	0.042603568
0.001422464	0.00941797	0.204926973	0.721276114	12.3232199	3478.146114
46.88689043	0.391622165	531.2950075	23.94964113	22.18992424	0.001542256

3820.009616	7.957270648	1.582118024	34.54294407	0.007834425	98.70656889
0.001422464	0.00941797	0.204926973	0.721276114	12.3232199	3478.146114
0.881985989	0.027067935	39.49331828	429342.378	0.011355996	0.077360679
246.9539207	3128.825214	3320.500866	1022.604151	660.0289615	3.439133266
507.1011936	1478.891687	1511.869022	2939.496313	32.29524662	0.873728374
0.881985989	0.027067935	39.49331828	429342.378	0.011355996	0.077360679
0.881985989	0.027067935	39.49331828	429342.378	0.011355996	0.077360679
234.174525	19.21560603	0.019944373	167.453655	157.6983767	276.7276739
46.88689043	0.391622165	531.2950075	23.94964113	22.18992424	0.001542256
25.36093266	0.030713913	5059.993294	0.003630134	333.5791205	24245.75888
507.1011936	1478.891687	1511.869022	2939.496313	32.29524662	0.873728374
0.001422464	0.00941797	0.204926973	0.721276114	12.3232199	3478.146114
507.1011936	1478.891687	1511.869022	2939.496313	32.29524662	0.873728374
83.20846796	78551.74488	2.927555361	6.945833152	119.7214744	656.7244025
10.83050708	42477.74287	13.76429903	68103.17337	10984.57956	0.001691148
0.881985989	0.027067935	39.49331828	429342.378	0.011355996	0.077360679
53.92016893	54780.47857	2.398800153	0.000251436	1134.11636	7.274232584
4119.001973	12.96182847	97978.19598	3026.535321	0.509946024	266.4579467
4.582263427	0.161047566	1.856342814	4.589482063	1.324464207	0.001090377
0.00253959	292.6377976	5956.773245	0.105152004	10617393.69	1.849463033
26.73621697	0.00719895	0.195071634	5952.751845	2.366244668	5.51508649
751.4658587	6.515515762	58.76152474	0.01232318	643.3277344	6658.145091
2492617.667	0.432218488	0.519781662	0.005046575	48444.92742	0.037759983
0.002489037	0.013015307	0.490936354	2.791297441	1002.558859	0.037197998
0.001422464	0.00941797	0.204926973	0.721276114	12.3232199	3478.146114
507.1011936	1478.891687	1511.869022	2939.496313	32.29524662	0.873728374
10.83050708	42477.74287	13.76429903	68103.17337	10984.57956	0.001691148
0.00210413	0.120368286	41.98575599	332885.5239	0.002614266	169.8590328
53.92016893	54780.47857	2.398800153	0.000251436	1134.11636	7.274232584
0.881985989	0.027067935	39.49331828	429342.378	0.011355996	0.077360679
0.881985989	0.027067935	39.49331828	429342.378	0.011355996	0.077360679
0.001422464	0.00941797	0.204926973	0.721276114	12.3232199	3478.146114
507.1011936	1478.891687	1511.869022	2939.496313	32.29524662	0.873728374
4119.001973	12.96182847	97978.19598	3026.535321	0.509946024	266.4579467
246.9539207	3128.825214	3320.500866	1022.604151	660.0289615	3.439133266
1407.3203	17.21923482	3.133003664	0.001215493	3.484959996	13.52883657
0.001422464	0.00941797	0.204926973	0.721276114	12.3232199	3478.146114
507.1011936	1478.891687	1511.869022	2939.496313	32.29524662	0.873728374
5737.994112	2783120.43	5.128574608	513876.0139	1303.951778	1759960.698
507.1011936	1478.891687	1511.869022	2939.496313	32.29524662	0.873728374
122.6515324	19.5047941	0.94053901	0.006485206	5.827767614	142.178948

2492617.667	0.432218488	0.519781662	0.005046575	48444.92742	0.037759983
0.881985989	0.027067935	39.49331828	429342.378	0.011355996	0.077360679
4119.001973	12.96182847	97978.19598	3026.535321	0.509946024	266.4579467
53.92016893	54780.47857	2.398800153	0.000251436	1134.11636	7.274232584
83.20846796	78551.74488	2.927555361	6.945833152	119.7214744	656.7244025
0.001422464	0.00941797	0.204926973	0.721276114	12.3232199	3478.146114
25.36093266	0.030713913	5059.993294	0.003630134	333.5791205	24245.75888
26.73621697	0.00719895	0.195071634	5952.751845	2.366244668	5.51508649
10.83050708	42477.74287	13.76429903	68103.17337	10984.57956	0.001691148
122.6515324	19.5047941	0.94053901	0.006485206	5.827767614	142.178948
53.92016893	54780.47857	2.398800153	0.000251436	1134.11636	7.274232584
303.7647121	0.040367691	88825.40181	31.83876004	65.96527827	23.08359483
25.36093266	0.030713913	5059.993294	0.003630134	333.5791205	24245.75888
0.025054755	62060.76279	108.4091933	1.659943972	0.006841767	0.046608309
507.1011936	1478.891687	1511.869022	2939.496313	32.29524662	0.873728374
25.36093266	0.030713913	5059.993294	0.003630134	333.5791205	24245.75888
507.1011936	1478.891687	1511.869022	2939.496313	32.29524662	0.873728374
53.92016893	54780.47857	2.398800153	0.000251436	1134.11636	7.274232584
0.881985989	0.027067935	39.49331828	429342.378	0.011355996	0.077360679
53.92016893	54780.47857	2.398800153	0.000251436	1134.11636	7.274232584
0.001422464	0.00941797	0.204926973	0.721276114	12.3232199	3478.146114
0.881985989	0.027067935	39.49331828	429342.378	0.011355996	0.077360679
0.001422464	0.00941797	0.204926973	0.721276114	12.3232199	3478.146114
0.004386538	612.351195	3834.828045	27.88750645	0.673629365	25.66378743
53.92016893	54780.47857	2.398800153	0.000251436	1134.11636	7.274232584
3820.009616	7.957270648	1.582118024	34.54294407	0.007834425	98.70656889
751.4658587	6.515515762	58.76152474	0.01232318	643.3277344	6658.145091
0.881985989	0.027067935	39.49331828	429342.378	0.011355996	0.077360679
2492617.667	0.432218488	0.519781662	0.005046575	48444.92742	0.037759983
0.881985989	0.027067935	39.49331828	429342.378	0.011355996	0.077360679
0.004386538	612.351195	3834.828045	27.88750645	0.673629365	25.66378743
53.92016893	54780.47857	2.398800153	0.000251436	1134.11636	7.274232584
2.357992593	0.013488471	0.163257793	13.4799025	2.499363915	26.17785932
10.83050708	42477.74287	13.76429903	68103.17337	10984.57956	0.001691148
303.7647121	0.040367691	88825.40181	31.83876004	65.96527827	23.08359483
10.83050708	42477.74287	13.76429903	68103.17337	10984.57956	0.001691148
0.001422464	0.00941797	0.204926973	0.721276114	12.3232199	3478.146114
1407.3203	17.21923482	3.133003664	0.001215493	3.484959996	13.52883657
53.92016893	54780.47857	2.398800153	0.000251436	1134.11636	7.274232584
0.00210413	0.120368286	41.98575599	332885.5239	0.002614266	169.8590328
68457.92115	2505.973416	28.92950345	64.45951709	1388.585013	0.042603568

1407.3203	17.21923482	3.133003664	0.001215493	3.484959996	13.52883657
0.002489037	0.013015307	0.490936354	2.791297441	1002.558859	0.037197998
507.1011936	1478.891687	1511.869022	2939.496313	32.29524662	0.873728374
507.1011936	1478.891687	1511.869022	2939.496313	32.29524662	0.873728374
0.001422464	0.00941797	0.204926973	0.721276114	12.3232199	3478.146114
2.357992593	0.013488471	0.163257793	13.4799025	2.499363915	26.17785932
5737.994112	2783120.43	5.128574608	513876.0139	1303.951778	1759960.698
16.85488392	145222.0361	393.9165404	54.44420646	63.29111729	0.030771725
2492617.667	0.432218488	0.519781662	0.005046575	48444.92742	0.037759983
2.357992593	0.013488471	0.163257793	13.4799025	2.499363915	26.17785932
0.002644611	0.013828814	2012.047599	0.005282202	16148.89966	9.589666162
507.1011936	1478.891687	1511.869022	2939.496313	32.29524662	0.873728374
4119.001973	12.96182847	97978.19598	3026.535321	0.509946024	266.4579467
0.007277636	1.909846368	0.943378642	2.382431085	628707.3608	22620.20466
39.78483772	345311.1709	7.712725298	0.004494878	354.6221056	360.4606496
2.357992593	0.013488471	0.163257793	13.4799025	2.499363915	26.17785932
68457.92115	2505.973416	28.92950345	64.45951709	1388.585013	0.042603568
53.92016893	54780.47857	2.398800153	0.000251436	1134.11636	7.274232584
507.1011936	1478.891687	1511.869022	2939.496313	32.29524662	0.873728374
53684.21014	13.10238807	118785.3992	37718.2922	53.04765393	761212318.6
0.881985989	0.027067935	39.49331828	429342.378	0.011355996	0.077360679
0.00210413	0.120368286	41.98575599	332885.5239	0.002614266	169.8590328
0.001422464	0.00941797	0.204926973	0.721276114	12.3232199	3478.146114
42.05187837	28157.9058	5.60105163	14.51839423	31.08904201	345.0495564
25.36093266	0.030713913	5059.993294	0.003630134	333.5791205	24245.75888
122.6515324	19.5047941	0.94053901	0.006485206	5.827767614	142.178948
793236.9018	19681.0082	12741.86914	140.6064572	2.851419285	1.100140828
0.881985989	0.027067935	39.49331828	429342.378	0.011355996	0.077360679
0.00210413	0.120368286	41.98575599	332885.5239	0.002614266	169.8590328
122.6515324	19.5047941	0.94053901	0.006485206	5.827767614	142.178948
53.92016893	54780.47857	2.398800153	0.000251436	1134.11636	7.274232584
2.357992593	0.013488471	0.163257793	13.4799025	2.499363915	26.17785932
0.00210413	0.120368286	41.98575599	332885.5239	0.002614266	169.8590328
1407.3203	17.21923482	3.133003664	0.001215493	3.484959996	13.52883657
16.85488392	145222.0361	393.9165404	54.44420646	63.29111729	0.030771725
2.357992593	0.013488471	0.163257793	13.4799025	2.499363915	26.17785932
507.1011936	1478.891687	1511.869022	2939.496313	32.29524662	0.873728374
3820.009616	7.957270648	1.582118024	34.54294407	0.007834425	98.70656889
2.357992593	0.013488471	0.163257793	13.4799025	2.499363915	26.17785932
25.36093266	0.030713913	5059.993294	0.003630134	333.5791205	24245.75888
507.1011936	1478.891687	1511.869022	2939.496313	32.29524662	0.873728374

0.881985989	0.027067935	39.49331828	429342.378	0.011355996	0.077360679
53684.21014	13.10238807	118785.3992	37718.2922	53.04765393	761212318.6
122.6515324	19.5047941	0.94053901	0.006485206	5.827767614	142.178948
0.025054755	62060.76279	108.4091933	1.659943972	0.006841767	0.046608309
83.20846796	78551.74488	2.927555361	6.945833152	119.7214744	656.7244025
42.05187837	28157.9058	5.60105163	14.51839423	31.08904201	345.0495564
46.88689043	0.391622165	531.2950075	23.94964113	22.18992424	0.001542256
25.36093266	0.030713913	5059.993294	0.003630134	333.5791205	24245.75888
25.36093266	0.030713913	5059.993294	0.003630134	333.5791205	24245.75888
0.001422464	0.00941797	0.204926973	0.721276114	12.3232199	3478.146114
0.00210413	0.120368286	41.98575599	332885.5239	0.002614266	169.8590328
122.6515324	19.5047941	0.94053901	0.006485206	5.827767614	142.178948
68457.92115	2505.973416	28.92950345	64.45951709	1388.585013	0.042603568
0.881985989	0.027067935	39.49331828	429342.378	0.011355996	0.077360679
83.20846796	78551.74488	2.927555361	6.945833152	119.7214744	656.7244025
0.001422464	0.00941797	0.204926973	0.721276114	12.3232199	3478.146114
0.881985989	0.027067935	39.49331828	429342.378	0.011355996	0.077360679
391.5511783	119356784.6	280778.3653	69.86173262	201.6378553	0.129436783
234.174525	19.21560603	0.019944373	167.453655	157.6983767	276.7276739
83.20846796	78551.74488	2.927555361	6.945833152	119.7214744	656.7244025
0.00253959	292.6377976	5956.773245	0.105152004	10617393.69	1.849463033
68457.92115	2505.973416	28.92950345	64.45951709	1388.585013	0.042603568
46.88689043	0.391622165	531.2950075	23.94964113	22.18992424	0.001542256
2492617.667	0.432218488	0.519781662	0.005046575	48444.92742	0.037759983
39.78483772	345311.1709	7.712725298	0.004494878	354.6221056	360.4606496
10.83050708	42477.74287	13.76429903	68103.17337	10984.57956	0.001691148
122.6515324	19.5047941	0.94053901	0.006485206	5.827767614	142.178948
4119.001973	12.96182847	97978.19598	3026.535321	0.509946024	266.4579467
507.1011936	1478.891687	1511.869022	2939.496313	32.29524662	0.873728374
246.9539207	3128.825214	3320.500866	1022.604151	660.0289615	3.439133266
11.30252243	12861.48096	4.49E-06	1152886.39	14149523.55	1.462553101
0.881985989	0.027067935	39.49331828	429342.378	0.011355996	0.077360679
68457.92115	2505.973416	28.92950345	64.45951709	1388.585013	0.042603568
122.6515324	19.5047941	0.94053901	0.006485206	5.827767614	142.178948
122.6515324	19.5047941	0.94053901	0.006485206	5.827767614	142.178948
0.00253959	292.6377976	5956.773245	0.105152004	10617393.69	1.849463033
0.00210413	0.120368286	41.98575599	332885.5239	0.002614266	169.8590328
1407.3203	17.21923482	3.133003664	0.001215493	3.484959996	13.52883657
0.025054755	62060.76279	108.4091933	1.659943972	0.006841767	0.046608309
25.36093266	0.030713913	5059.993294	0.003630134	333.5791205	24245.75888
0.007277636	1.909846368	0.943378642	2.382431085	628707.3608	22620.20466

0.002644611	0.013828814	2012.047599	0.005282202	16148.89966	9.589666162
0.881985989	0.027067935	39.49331828	429342.378	0.011355996	0.077360679
4119.001973	12.96182847	97978.19598	3026.535321	0.509946024	266.4579467
25.36093266	0.030713913	5059.993294	0.003630134	333.5791205	24245.75888
0.881985989	0.027067935	39.49331828	429342.378	0.011355996	0.077360679
2492617.667	0.432218488	0.519781662	0.005046575	48444.92742	0.037759983
2.357992593	0.013488471	0.163257793	13.4799025	2.499363915	26.17785932
0.00210413	0.120368286	41.98575599	332885.5239	0.002614266	169.8590328
68457.92115	2505.973416	28.92950345	64.45951709	1388.585013	0.042603568
0.001422464	0.00941797	0.204926973	0.721276114	12.3232199	3478.146114
0.00210413	0.120368286	41.98575599	332885.5239	0.002614266	169.8590328
507.1011936	1478.891687	1511.869022	2939.496313	32.29524662	0.873728374
0.007277636	1.909846368	0.943378642	2.382431085	628707.3608	22620.20466
25.36093266	0.030713913	5059.993294	0.003630134	333.5791205	24245.75888
42.05187837	28157.9058	5.60105163	14.51839423	31.08904201	345.0495564
0.001422464	0.00941797	0.204926973	0.721276114	12.3232199	3478.146114
42.05187837	28157.9058	5.60105163	14.51839423	31.08904201	345.0495564
0.001422464	0.00941797	0.204926973	0.721276114	12.3232199	3478.146114
45	46	47	48	49	50
PKNOX1-Hs01007093_m1	PKNOX1-Hs01007092_m1	PKNOX1-Hs00231814_m1	CYYR1-Hs00951849_m1	SLC19A1-Hs00953342_m1	SLC19A1-Hs00953341_m1

51	52	53	54
PRDM15-Hs00411318_m1	COL6A1-Hs01095585_m1	ABCG1-Hs01555191_m1	GART-Hs00531926_m1
7.483255928	2.401556547	2.94E-05	29.67086411
255171.4227	5.435260621	3.373976106	13.27128917
906943.9958	0.022675079	2902984.457	294272.3268
887.7015739	268.867245	537869.0348	1575.978134
3.826846881	0.000352521	1.314669839	0.752089129
0.002959193	0.974699136	360.3031033	0.560732943
38.81606051	2870.445911	0.000960285	0.307850823
59.43646253	18.01032158	4.58E-05	0.583008225
29.07905701	13.63044419	355.468137	0.018604776
0.001427863	46.1977074	0.000540296	2.087265415
0.001458959	0.024653542	0.000552062	21.24301193
887.7015739	268.867245	537869.0348	1575.978134
4603.875712	7.034692477	230.0451047	10797.36059
29.07905701	13.63044419	355.468137	0.018604776
0.001682591	23.33704731	0.459601366	79.88596913
0.65549061	20.7802547	21843.65722	2.506670205
23174138.56	250.9911268	109.2217014	2.856995989
173.7434427	4.063382558	2.284860439	10.71924058
906943.9958	0.022675079	2902984.457	294272.3268
2864.891549	2781.350945	10128.79592	4177.736305
0.65549061	20.7802547	21843.65722	2.506670205
7.974558474	43.61972245	0.075218624	2312.861929
65.20437042	0.00668205	372.4579484	1013.890678
0.001458959	0.024653542	0.000552062	21.24301193
3.826846881	0.000352521	1.314669839	0.752089129
65.20437042	0.00668205	372.4579484	1013.890678
0.001427863	46.1977074	0.000540296	2.087265415
0.001458959	0.024653542	0.000552062	21.24301193
0.001682591	23.33704731	0.459601366	79.88596913
29.07905701	13.63044419	355.468137	0.018604776
7.483255928	2.401556547	2.94E-05	29.67086411
444.5768852	368.2917494	10.99167495	11.65881358
0.65549061	20.7802547	21843.65722	2.506670205
0.002959193	0.974699136	360.3031033	0.560732943
0.000403263	2.102814336	15.49445659	0.221416388
0.001427863	46.1977074	0.000540296	2.087265415
7.974558474	43.61972245	0.075218624	2312.861929

65.20437042	0.00668205	372.4579484	1013.890678
0.002959193	0.974699136	360.3031033	0.560732943
29.88245829	128.4724746	258736.5839	259.3966273
0.65549061	20.7802547	21843.65722	2.506670205
0.001427863	46.1977074	0.000540296	2.087265415
0.002959193	0.974699136	360.3031033	0.560732943
444.5768852	368.2917494	10.99167495	11.65881358
2864.891549	2781.350945	10128.79592	4177.736305
3.826846881	0.000352521	1.314669839	0.752089129
29.88245829	128.4724746	258736.5839	259.3966273
3.826846881	0.000352521	1.314669839	0.752089129
906943.9958	0.022675079	2902984.457	294272.3268
7.974558474	43.61972245	0.075218624	2312.861929
0.000621218	0.001051526	0.000235066	0.776173373
17.97047524	3137.655643	5107264.194	0.110313418
238.5822635	1254.567176	0.011078274	17.44998909
26.39163536	6073.744781	96.4708329	4.900801524
16.79057795	54.01345836	215.3946583	90.95807077
444.9439191	493.0399195	220.5042306	111.6163211
0.003255347	46.61255529	0.001231806	24.23669999
26.39163536	6073.744781	96.4708329	4.900801524
92.71200379	85.77269248	6.14E-05	0.055037026
44.04480964	396.4183114	16.81160169	22.21980926
238.5822635	1254.567176	0.011078274	17.44998909
18487653.09	0.003102205	24182.7109	14.50127004
444.9439191	493.0399195	220.5042306	111.6163211
0.00367292	205.3538926	107.7500234	8.656298264
18487653.09	0.003102205	24182.7109	14.50127004
26.39163536	6073.744781	96.4708329	4.900801524
444.9439191	493.0399195	220.5042306	111.6163211
325.3362474	0.953297937	0.82421472	1.817875669
326298.382	1511.906117	32422.88517	47.19092299
92.71200379	85.77269248	6.14E-05	0.055037026
18487653.09	0.003102205	24182.7109	14.50127004
0.00013296	48.99647608	0.000829599	1.426632815
26.39163536	6073.744781	96.4708329	4.900801524
0.003255347	46.61255529	0.001231806	24.23669999
18487653.09	0.003102205	24182.7109	14.50127004
26.39163536	6073.744781	96.4708329	4.900801524
18487653.09	0.003102205	24182.7109	14.50127004
36472.41117	138.9546577	2.804091203	29.94239575

6.618400234	618982.885	8812.10418	10.11805667
17.97047524	3137.655643	5107264.194	0.110313418
238.5822635	1254.567176	0.011078274	17.44998909
16.79057795	54.01345836	215.3946583	90.95807077
3521.376477	0.061106073	51.99743357	0.084609634
92.71200379	85.77269248	6.14E-05	0.055037026
36472.41117	138.9546577	2.804091203	29.94239575
0.002079684	24.86448114	2.542027574	1.103747429
92.71200379	85.77269248	6.14E-05	0.055037026
0.00327203	8.814760671	0.003052285	0.067559455
0.00013296	48.99647608	0.000829599	1.426632815
6.543337611	0.005625601	73.90578451	8.944156771
444.9439191	493.0399195	220.5042306	111.6163211
26.39163536	6073.744781	96.4708329	4.900801524
444.9439191	493.0399195	220.5042306	111.6163211
18487653.09	0.003102205	24182.7109	14.50127004
18487653.09	0.003102205	24182.7109	14.50127004
18487653.09	0.003102205	24182.7109	14.50127004
18487653.09	0.003102205	24182.7109	14.50127004
18487653.09	0.003102205	24182.7109	14.50127004
4699.689732	16.5445412	56.996003	3.920494479
92.71200379	85.77269248	6.14E-05	0.055037026
26.39163536	6073.744781	96.4708329	4.900801524
238.5822635	1254.567176	0.011078274	17.44998909
26.39163536	6073.744781	96.4708329	4.900801524
444.9439191	493.0399195	220.5042306	111.6163211
92.71200379	85.77269248	6.14E-05	0.055037026
443.3872409	0.00542826	8.971843259	34.66796735
18487653.09	0.003102205	24182.7109	14.50127004
18487653.09	0.003102205	24182.7109	14.50127004
2.921087263	291.1689411	0.003007935	161.5382025
0.002341657	0.003963682	2545.630641	2.259819368
443.3872409	0.00542826	8.971843259	34.66796735
444.9439191	493.0399195	220.5042306	111.6163211
0.003407341	0.005767548	220.7173297	0.000234915
44.04480964	396.4183114	16.81160169	22.21980926
0.000621218	0.001051526	0.000235066	0.776173373
0.002079684	24.86448114	2.542027574	1.103747429
16.79057795	54.01345836	215.3946583	90.95807077
444.9439191	493.0399195	220.5042306	111.6163211
0.002079684	24.86448114	2.542027574	1.103747429

18487653.09	0.003102205	24182.7109	14.50127004
26.10673933	0.007788311	0.065280254	2.335333077
444.9439191	493.0399195	220.5042306	111.6163211
44.04480964	396.4183114	16.81160169	22.21980926
26.39163536	6073.744781	96.4708329	4.900801524
444.9439191	493.0399195	220.5042306	111.6163211
238.5822635	1254.567176	0.011078274	17.44998909
26.10673933	0.007788311	0.065280254	2.335333077
444.9439191	493.0399195	220.5042306	111.6163211
26.39163536	6073.744781	96.4708329	4.900801524
18487653.09	0.003102205	24182.7109	14.50127004
0.003255347	46.61255529	0.001231806	24.23669999
444.9439191	493.0399195	220.5042306	111.6163211
26.39163536	6073.744781	96.4708329	4.900801524
444.9439191	493.0399195	220.5042306	111.6163211
0.003255347	46.61255529	0.001231806	24.23669999
444.9439191	493.0399195	220.5042306	111.6163211
64302.06533	19.67707718	8.016328145	11.7087833
443.3872409	0.00542826	8.971843259	34.66796735
0.00013296	48.99647608	0.000829599	1.426632815
541278.185	83.3727028	1.350728114	42.74555378
4699.689732	16.5445412	56.996003	3.920494479
26.39163536	6073.744781	96.4708329	4.900801524
326298.382	1511.906117	32422.88517	47.19092299
16.79057795	54.01345836	215.3946583	90.95807077
0.002341657	0.003963682	2545.630641	2.259819368
26.10673933	0.007788311	0.065280254	2.335333077
0.003255347	46.61255529	0.001231806	24.23669999
4289681.134	390326.2634	16963.31967	212.054701
541278.185	83.3727028	1.350728114	42.74555378
26.39163536	6073.744781	96.4708329	4.900801524
36472.41117	138.9546577	2.804091203	29.94239575
26.10673933	0.007788311	0.065280254	2.335333077
26.39163536	6073.744781	96.4708329	4.900801524
16.79057795	54.01345836	215.3946583	90.95807077
18487653.09	0.003102205	24182.7109	14.50127004
444.9439191	493.0399195	220.5042306	111.6163211
17.97047524	3137.655643	5107264.194	0.110313418
26.39163536	6073.744781	96.4708329	4.900801524
26.39163536	6073.744781	96.4708329	4.900801524
0.003255347	46.61255529	0.001231806	24.23669999

0.002079684	24.86448114	2.542027574	1.103747429
4699.689732	16.5445412	56.996003	3.920494479
258.8753146	447.4771016	676287.9066	6.143344583
23454.39012	186404.0517	0.673319243	88.35793893
0.002652878	52.58453061	249.5887533	4.956175605
0.00013296	48.99647608	0.000829599	1.426632815
26.10673933	0.007788311	0.065280254	2.335333077
92.71200379	85.77269248	6.14E-05	0.055037026
44.04480964	396.4183114	16.81160169	22.21980926
443.3872409	0.00542826	8.971843259	34.66796735
541278.185	83.3727028	1.350728114	42.74555378
36472.41117	138.9546577	2.804091203	29.94239575
18487653.09	0.003102205	24182.7109	14.50127004
18487653.09	0.003102205	24182.7109	14.50127004
92.71200379	85.77269248	6.14E-05	0.055037026
18487653.09	0.003102205	24182.7109	14.50127004
18487653.09	0.003102205	24182.7109	14.50127004
18487653.09	0.003102205	24182.7109	14.50127004
18487653.09	0.003102205	24182.7109	14.50127004
92.71200379	85.77269248	6.14E-05	0.055037026
444.9439191	493.0399195	220.5042306	111.6163211
18487653.09	0.003102205	24182.7109	14.50127004
26.39163536	6073.744781	96.4708329	4.900801524
18487653.09	0.003102205	24182.7109	14.50127004
325.3362474	0.953297937	0.82421472	1.817875669
0.002341657	0.003963682	2545.630641	2.259819368
36472.41117	138.9546577	2.804091203	29.94239575
36472.41117	138.9546577	2.804091203	29.94239575
17.97047524	3137.655643	5107264.194	0.110313418
0.270067477	0.377395125	24.08311636	2.057954211
18487653.09	0.003102205	24182.7109	14.50127004
26.39163536	6073.744781	96.4708329	4.900801524
4289681.134	390326.2634	16963.31967	212.054701
2.306166232	2.799578684	0.000113672	0.425449008
26.39163536	6073.744781	96.4708329	4.900801524
4289681.134	390326.2634	16963.31967	212.054701
0.002079684	24.86448114	2.542027574	1.103747429
238.5822635	1254.567176	0.011078274	17.44998909
26.39163536	6073.744781	96.4708329	4.900801524
26.39163536	6073.744781	96.4708329	4.900801524
18487653.09	0.003102205	24182.7109	14.50127004

4289681.134	390326.2634	16963.31967	212.054701
92.71200379	85.77269248	6.14E-05	0.055037026
18487653.09	0.003102205	24182.7109	14.50127004
0.002341657	0.003963682	2545.630641	2.259819368
26.39163536	6073.744781	96.4708329	4.900801524
6.618400234	618982.885	8812.10418	10.11805667
16.79057795	54.01345836	215.3946583	90.95807077
6.618400234	618982.885	8812.10418	10.11805667
44.04480964	396.4183114	16.81160169	22.21980926
541278.185	83.3727028	1.350728114	42.74555378
0.003255347	46.61255529	0.001231806	24.23669999
444.9439191	493.0399195	220.5042306	111.6163211
26.39163536	6073.744781	96.4708329	4.900801524
16.79057795	54.01345836	215.3946583	90.95807077
18487653.09	0.003102205	24182.7109	14.50127004
0.003255347	46.61255529	0.001231806	24.23669999
4699.689732	16.5445412	56.996003	3.920494479
6.543337611	0.005625601	73.90578451	8.944156771
17.97047524	3137.655643	5107264.194	0.110313418
4289681.134	390326.2634	16963.31967	212.054701
0.002341657	0.003963682	2545.630641	2.259819368
444.9439191	493.0399195	220.5042306	111.6163211
0.002079684	24.86448114	2.542027574	1.103747429
36472.41117	138.9546577	2.804091203	29.94239575
0.00013296	48.99647608	0.000829599	1.426632815
444.9439191	493.0399195	220.5042306	111.6163211
18487653.09	0.003102205	24182.7109	14.50127004
18487653.09	0.003102205	24182.7109	14.50127004
16.79057795	54.01345836	215.3946583	90.95807077
18487653.09	0.003102205	24182.7109	14.50127004
0.003255347	46.61255529	0.001231806	24.23669999
18487653.09	0.003102205	24182.7109	14.50127004
18487653.09	0.003102205	24182.7109	14.50127004
444.9439191	493.0399195	220.5042306	111.6163211
444.9439191	493.0399195	220.5042306	111.6163211
26.39163536	6073.744781	96.4708329	4.900801524
92.71200379	85.77269248	6.14E-05	0.055037026
444.9439191	493.0399195	220.5042306	111.6163211
0.00013296	48.99647608	0.000829599	1.426632815
18487653.09	0.003102205	24182.7109	14.50127004
26.39163536	6073.744781	96.4708329	4.900801524

0.003255347	46.61255529	0.001231806	24.23669999
4289681.134	390326.2634	16963.31967	212.054701
92.71200379	85.77269248	6.14E-05	0.055037026
326298.382	1511.906117	32422.88517	47.19092299
36472.41117	138.9546577	2.804091203	29.94239575
6.618400234	618982.885	8812.10418	10.11805667
16.79057795	54.01345836	215.3946583	90.95807077
0.002341657	0.003963682	2545.630641	2.259819368
541278.185	83.3727028	1.350728114	42.74555378
26.39163536	6073.744781	96.4708329	4.900801524
16.79057795	54.01345836	215.3946583	90.95807077
541278.185	83.3727028	1.350728114	42.74555378
0.002341657	0.003963682	2545.630641	2.259819368
0.002079684	24.86448114	2.542027574	1.103747429
26.10673933	0.007788311	0.065280254	2.335333077
26.39163536	6073.744781	96.4708329	4.900801524
0.00013296	48.99647608	0.000829599	1.426632815
4699.689732	16.5445412	56.996003	3.920494479
36472.41117	138.9546577	2.804091203	29.94239575
18487653.09	0.003102205	24182.7109	14.50127004
16.79057795	54.01345836	215.3946583	90.95807077
18487653.09	0.003102205	24182.7109	14.50127004
26.39163536	6073.744781	96.4708329	4.900801524
0.00013296	48.99647608	0.000829599	1.426632815
18487653.09	0.003102205	24182.7109	14.50127004
444.9439191	493.0399195	220.5042306	111.6163211
541278.185	83.3727028	1.350728114	42.74555378
541278.185	83.3727028	1.350728114	42.74555378
0.00367292	205.3538926	107.7500234	8.656298264
26.39163536	6073.744781	96.4708329	4.900801524
0.00013296	48.99647608	0.000829599	1.426632815
18487653.09	0.003102205	24182.7109	14.50127004
2.210941183	806.7370046	0.001185215	0.396914612
18487653.09	0.003102205	24182.7109	14.50127004
541278.185	83.3727028	1.350728114	42.74555378
0.002341657	0.003963682	2545.630641	2.259819368
0.003255347	46.61255529	0.001231806	24.23669999
26.10673933	0.007788311	0.065280254	2.335333077
26.39163536	6073.744781	96.4708329	4.900801524
18487653.09	0.003102205	24182.7109	14.50127004
444.9439191	493.0399195	220.5042306	111.6163211

444.9439191	493.0399195	220.5042306	111.6163211
26.39163536	6073.744781	96.4708329	4.900801524
18487653.09	0.003102205	24182.7109	14.50127004
2.210941183	806.7370046	0.001185215	0.396914612
18487653.09	0.003102205	24182.7109	14.50127004
18487653.09	0.003102205	24182.7109	14.50127004
444.9439191	493.0399195	220.5042306	111.6163211
0.003255347	46.61255529	0.001231806	24.23669999
26.39163536	6073.744781	96.4708329	4.900801524
0.002341657	0.003963682	2545.630641	2.259819368
92.71200379	85.77269248	6.14E-05	0.055037026
23454.39012	186404.0517	0.673319243	88.35793893
18487653.09	0.003102205	24182.7109	14.50127004
444.9439191	493.0399195	220.5042306	111.6163211
92.71200379	85.77269248	6.14E-05	0.055037026
1004933.283	1.201854084	0.002549948	151.0739372
541278.185	83.3727028	1.350728114	42.74555378
3521.376477	0.061106073	51.99743357	0.084609634
0.003255347	46.61255529	0.001231806	24.23669999
541278.185	83.3727028	1.350728114	42.74555378
4289681.134	390326.2634	16963.31967	212.054701
0.000621218	0.001051526	0.000235066	0.776173373
2.921087263	291.1689411	0.003007935	161.5382025
0.002079684	24.86448114	2.542027574	1.103747429
26.39163536	6073.744781	96.4708329	4.900801524
4699.689732	16.5445412	56.996003	3.920494479
4289681.134	390326.2634	16963.31967	212.054701
36472.41117	138.9546577	2.804091203	29.94239575
6.543337611	0.005625601	73.90578451	8.944156771
2.210941183	806.7370046	0.001185215	0.396914612
0.002341657	0.003963682	2545.630641	2.259819368
36472.41117	138.9546577	2.804091203	29.94239575
26.39163536	6073.744781	96.4708329	4.900801524
2.921087263	291.1689411	0.003007935	161.5382025
0.00367292	205.3538926	107.7500234	8.656298264
26.39163536	6073.744781	96.4708329	4.900801524
26.10673933	0.007788311	0.065280254	2.335333077
541278.185	83.3727028	1.350728114	42.74555378
26.39163536	6073.744781	96.4708329	4.900801524
26.39163536	6073.744781	96.4708329	4.900801524
1004933.283	1.201854084	0.002549948	151.0739372

18487653.09	0.003102205	24182.7109	14.50127004
654.7408322	0.000833079	3195.258006	2.52234018
26.39163536	6073.744781	96.4708329	4.900801524
444.9439191	493.0399195	220.5042306	111.6163211
0.002341657	0.003963682	2545.630641	2.259819368
16.79057795	54.01345836	215.3946583	90.95807077
541278.185	83.3727028	1.350728114	42.74555378
26.39163536	6073.744781	96.4708329	4.900801524
444.9439191	493.0399195	220.5042306	111.6163211
0.00013296	48.99647608	0.000829599	1.426632815
26.39163536	6073.744781	96.4708329	4.900801524
18487653.09	0.003102205	24182.7109	14.50127004
2.306166232	2.799578684	0.000113672	0.425449008
444.9439191	493.0399195	220.5042306	111.6163211
26.39163536	6073.744781	96.4708329	4.900801524
26.39163536	6073.744781	96.4708329	4.900801524
0.00013296	48.99647608	0.000829599	1.426632815
444.9439191	493.0399195	220.5042306	111.6163211
3521.376477	0.061106073	51.99743357	0.084609634
0.000621218	0.001051526	0.000235066	0.776173373
2.921087263	291.1689411	0.003007935	161.5382025
26.39163536	6073.744781	96.4708329	4.900801524
26.39163536	6073.744781	96.4708329	4.900801524
0.003255347	46.61255529	0.001231806	24.23669999
4289681.134	390326.2634	16963.31967	212.054701
0.270067477	0.377395125	24.08311636	2.057954211
92.71200379	85.77269248	6.14E-05	0.055037026
443.3872409	0.00542826	8.971843259	34.66796735
444.9439191	493.0399195	220.5042306	111.6163211
541278.185	83.3727028	1.350728114	42.74555378
36472.41117	138.9546577	2.804091203	29.94239575
18487653.09	0.003102205	24182.7109	14.50127004
6.618400234	618982.885	8812.10418	10.11805667
26.39163536	6073.744781	96.4708329	4.900801524
444.9439191	493.0399195	220.5042306	111.6163211
18487653.09	0.003102205	24182.7109	14.50127004
92.71200379	85.77269248	6.14E-05	0.055037026
9.40E-05	1427.057408	23.31089059	0.107337433
26.39163536	6073.744781	96.4708329	4.900801524
26.39163536	6073.744781	96.4708329	4.900801524
26.39163536	6073.744781	96.4708329	4.900801524

0.002341657	0.003963682	2545.630641	2.259819368
44.04480964	396.4183114	16.81160169	22.21980926
18487653.09	0.003102205	24182.7109	14.50127004
26.39163536	6073.744781	96.4708329	4.900801524
0.00367292	205.3538926	107.7500234	8.656298264
18487653.09	0.003102205	24182.7109	14.50127004
92.71200379	85.77269248	6.14E-05	0.055037026
36472.41117	138.9546577	2.804091203	29.94239575
6.618400234	618982.885	8812.10418	10.11805667
258.8753146	447.4771016	676287.9066	6.143344583
44.04480964	396.4183114	16.81160169	22.21980926
44.04480964	396.4183114	16.81160169	22.21980926
92.71200379	85.77269248	6.14E-05	0.055037026
0.002341657	0.003963682	2545.630641	2.259819368
26.39163536	6073.744781	96.4708329	4.900801524
444.9439191	493.0399195	220.5042306	111.6163211
26.39163536	6073.744781	96.4708329	4.900801524
444.9439191	493.0399195	220.5042306	111.6163211
18487653.09	0.003102205	24182.7109	14.50127004
16.79057795	54.01345836	215.3946583	90.95807077
3521.376477	0.061106073	51.99743357	0.084609634
0.03112723	25783.58565	687745.5549	0.247096243
444.9439191	493.0399195	220.5042306	111.6163211
444.9439191	493.0399195	220.5042306	111.6163211
44.04480964	396.4183114	16.81160169	22.21980926
4699.689732	16.5445412	56.996003	3.920494479
444.9439191	493.0399195	220.5042306	111.6163211
444.9439191	493.0399195	220.5042306	111.6163211
0.003255347	46.61255529	0.001231806	24.23669999
238.5822635	1254.567176	0.011078274	17.44998909
0.003255347	46.61255529	0.001231806	24.23669999
0.003255347	46.61255529	0.001231806	24.23669999
18487653.09	0.003102205	24182.7109	14.50127004
4289681.134	390326.2634	16963.31967	212.054701
92.71200379	85.77269248	6.14E-05	0.055037026
444.9439191	493.0399195	220.5042306	111.6163211
2.921087263	291.1689411	0.003007935	161.5382025
0.00367292	205.3538926	107.7500234	8.656298264
36472.41117	138.9546577	2.804091203	29.94239575
444.9439191	493.0399195	220.5042306	111.6163211
26.39163536	6073.744781	96.4708329	4.900801524

44.04480964	396.4183114	16.81160169	22.21980926
444.9439191	493.0399195	220.5042306	111.6163211
4289681.134	390326.2634	16963.31967	212.054701
9.40E-05	1427.057408	23.31089059	0.107337433
18487653.09	0.003102205	24182.7109	14.50127004
0.002341657	0.003963682	2545.630641	2.259819368
44.04480964	396.4183114	16.81160169	22.21980926
0.00367292	205.3538926	107.7500234	8.656298264
4699.689732	16.5445412	56.996003	3.920494479
4699.689732	16.5445412	56.996003	3.920494479
16.79057795	54.01345836	215.3946583	90.95807077
0.003255347	46.61255529	0.001231806	24.23669999
18487653.09	0.003102205	24182.7109	14.50127004
238.5822635	1254.567176	0.011078274	17.44998909
92.71200379	85.77269248	6.14E-05	0.055037026
26.10673933	0.007788311	0.065280254	2.335333077
92.71200379	85.77269248	6.14E-05	0.055037026
36472.41117	138.9546577	2.804091203	29.94239575
26.39163536	6073.744781	96.4708329	4.900801524
0.00367292	205.3538926	107.7500234	8.656298264
0.002079684	24.86448114	2.542027574	1.103747429
18487653.09	0.003102205	24182.7109	14.50127004
0.00367292	205.3538926	107.7500234	8.656298264
0.000621218	0.001051526	0.000235066	0.776173373
26.39163536	6073.744781	96.4708329	4.900801524
0.002341657	0.003963682	2545.630641	2.259819368
0.00013296	48.99647608	0.000829599	1.426632815
444.9439191	493.0399195	220.5042306	111.6163211
36472.41117	138.9546577	2.804091203	29.94239575
17.97047524	3137.655643	5107264.194	0.110313418
0.003255347	46.61255529	0.001231806	24.23669999
541278.185	83.3727028	1.350728114	42.74555378
26.10673933	0.007788311	0.065280254	2.335333077
18487653.09	0.003102205	24182.7109	14.50127004
23454.39012	186404.0517	0.673319243	88.35793893
36472.41117	138.9546577	2.804091203	29.94239575
444.9439191	493.0399195	220.5042306	111.6163211
26.39163536	6073.744781	96.4708329	4.900801524
36472.41117	138.9546577	2.804091203	29.94239575
26.39163536	6073.744781	96.4708329	4.900801524
16.79057795	54.01345836	215.3946583	90.95807077

3521.376477	0.061106073	51.99743357	0.084609634
92.71200379	85.77269248	6.14E-05	0.055037026
238.5822635	1254.567176	0.011078274	17.44998909
36472.41117	138.9546577	2.804091203	29.94239575
0.002341657	0.003963682	2545.630641	2.259819368
44.04480964	396.4183114	16.81160169	22.21980926
26.39163536	6073.744781	96.4708329	4.900801524
18487653.09	0.003102205	24182.7109	14.50127004
18487653.09	0.003102205	24182.7109	14.50127004
654.7408322	0.000833079	3195.258006	2.52234018
6.543337611	0.005625601	73.90578451	8.944156771
36472.41117	138.9546577	2.804091203	29.94239575
26.39163536	6073.744781	96.4708329	4.900801524
444.9439191	493.0399195	220.5042306	111.6163211
18487653.09	0.003102205	24182.7109	14.50127004
4289681.134	390326.2634	16963.31967	212.054701
0.002341657	0.003963682	2545.630641	2.259819368
18487653.09	0.003102205	24182.7109	14.50127004
654.7408322	0.000833079	3195.258006	2.52234018
541278.185	83.3727028	1.350728114	42.74555378
18487653.09	0.003102205	24182.7109	14.50127004
92.71200379	85.77269248	6.14E-05	0.055037026
36472.41117	138.9546577	2.804091203	29.94239575
36472.41117	138.9546577	2.804091203	29.94239575
23454.39012	186404.0517	0.673319243	88.35793893
238.5822635	1254.567176	0.011078274	17.44998909
238.5822635	1254.567176	0.011078274	17.44998909
36472.41117	138.9546577	2.804091203	29.94239575
326298.382	1511.906117	32422.88517	47.19092299
16.79057795	54.01345836	215.3946583	90.95807077
26.39163536	6073.744781	96.4708329	4.900801524
0.00367292	205.3538926	107.7500234	8.656298264
0.270067477	0.377395125	24.08311636	2.057954211
2.210941183	806.7370046	0.001185215	0.396914612
654.7408322	0.000833079	3195.258006	2.52234018
0.270067477	0.377395125	24.08311636	2.057954211
18487653.09	0.003102205	24182.7109	14.50127004
443.3872409	0.00542826	8.971843259	34.66796735
26.39163536	6073.744781	96.4708329	4.900801524
443.3872409	0.00542826	8.971843259	34.66796735
26.39163536	6073.744781	96.4708329	4.900801524

18487653.09	0.003102205	24182.7109	14.50127004
18487653.09	0.003102205	24182.7109	14.50127004
26.39163536	6073.744781	96.4708329	4.900801524
18487653.09	0.003102205	24182.7109	14.50127004
26.10673933	0.007788311	0.065280254	2.335333077
26.39163536	6073.744781	96.4708329	4.900801524
26.39163536	6073.744781	96.4708329	4.900801524
0.03112723	25783.58565	687745.5549	0.247096243
258.8753146	447.4771016	676287.9066	6.143344583
2.306166232	2.799578684	0.000113672	0.425449008
6.543337611	0.005625601	73.90578451	8.944156771
541278.185	83.3727028	1.350728114	42.74555378
0.00327203	8.814760671	0.003052285	0.067559455
0.002079684	24.86448114	2.542027574	1.103747429
326298.382	1511.906117	32422.88517	47.19092299
36472.41117	138.9546577	2.804091203	29.94239575
92.71200379	85.77269248	6.14E-05	0.055037026
18487653.09	0.003102205	24182.7109	14.50127004
0.00367292	205.3538926	107.7500234	8.656298264
4699.689732	16.5445412	56.996003	3.920494479
18487653.09	0.003102205	24182.7109	14.50127004
26.10673933	0.007788311	0.065280254	2.335333077
0.003255347	46.61255529	0.001231806	24.23669999
18487653.09	0.003102205	24182.7109	14.50127004
26.39163536	6073.744781	96.4708329	4.900801524
4699.689732	16.5445412	56.996003	3.920494479
444.9439191	493.0399195	220.5042306	111.6163211
326298.382	1511.906117	32422.88517	47.19092299
0.00327203	8.814760671	0.003052285	0.067559455
0.00367292	205.3538926	107.7500234	8.656298264
36472.41117	138.9546577	2.804091203	29.94239575
0.003255347	46.61255529	0.001231806	24.23669999
0.002341657	0.003963682	2545.630641	2.259819368
26.10673933	0.007788311	0.065280254	2.335333077
92.71200379	85.77269248	6.14E-05	0.055037026
18487653.09	0.003102205	24182.7109	14.50127004
36472.41117	138.9546577	2.804091203	29.94239575
541278.185	83.3727028	1.350728114	42.74555378
0.003255347	46.61255529	0.001231806	24.23669999
444.9439191	493.0399195	220.5042306	111.6163211
16.79057795	54.01345836	215.3946583	90.95807077

4289681.134	390326.2634	16963.31967	212.054701
26.39163536	6073.744781	96.4708329	4.900801524
26.39163536	6073.744781	96.4708329	4.900801524
238.5822635	1254.567176	0.011078274	17.44998909
444.9439191	493.0399195	220.5042306	111.6163211
23454.39012	186404.0517	0.673319243	88.35793893
18487653.09	0.003102205	24182.7109	14.50127004
18487653.09	0.003102205	24182.7109	14.50127004
2.921087263	291.1689411	0.003007935	161.5382025
26.39163536	6073.744781	96.4708329	4.900801524
18487653.09	0.003102205	24182.7109	14.50127004
36472.41117	138.9546577	2.804091203	29.94239575
0.00367292	205.3538926	107.7500234	8.656298264
0.003255347	46.61255529	0.001231806	24.23669999
18487653.09	0.003102205	24182.7109	14.50127004
444.9439191	493.0399195	220.5042306	111.6163211
18487653.09	0.003102205	24182.7109	14.50127004
0.003255347	46.61255529	0.001231806	24.23669999
443.3872409	0.00542826	8.971843259	34.66796735
36472.41117	138.9546577	2.804091203	29.94239575
0.00367292	205.3538926	107.7500234	8.656298264
0.03112723	25783.58565	687745.5549	0.247096243
92.71200379	85.77269248	6.14E-05	0.055037026
444.9439191	493.0399195	220.5042306	111.6163211
0.003255347	46.61255529	0.001231806	24.23669999
64302.06533	19.67707718	8.016328145	11.7087833
4699.689732	16.5445412	56.996003	3.920494479
18487653.09	0.003102205	24182.7109	14.50127004
444.9439191	493.0399195	220.5042306	111.6163211
44.04480964	396.4183114	16.81160169	22.21980926
26.39163536	6073.744781	96.4708329	4.900801524
18487653.09	0.003102205	24182.7109	14.50127004
258.8753146	447.4771016	676287.9066	6.143344583
18487653.09	0.003102205	24182.7109	14.50127004
0.002341657	0.003963682	2545.630641	2.259819368
0.003255347	46.61255529	0.001231806	24.23669999
2.306166232	2.799578684	0.000113672	0.425449008
16.79057795	54.01345836	215.3946583	90.95807077
238.5822635	1254.567176	0.011078274	17.44998909
16.79057795	54.01345836	215.3946583	90.95807077
0.00013296	48.99647608	0.000829599	1.426632815

444.9439191	493.0399195	220.5042306	111.6163211
0.003255347	46.61255529	0.001231806	24.23669999
16.79057795	54.01345836	215.3946583	90.95807077
44.04480964	396.4183114	16.81160169	22.21980926
444.9439191	493.0399195	220.5042306	111.6163211
18487653.09	0.003102205	24182.7109	14.50127004
0.002079684	24.86448114	2.542027574	1.103747429
0.002341657	0.003963682	2545.630641	2.259819368
44.04480964	396.4183114	16.81160169	22.21980926
26.39163536	6073.744781	96.4708329	4.900801524
0.002341657	0.003963682	2545.630641	2.259819368
0.002341657	0.003963682	2545.630641	2.259819368
36472.41117	138.9546577	2.804091203	29.94239575
0.002899469	44.20196441	82.72672859	1.433566977
18487653.09	0.003102205	24182.7109	14.50127004
2.921087263	291.1689411	0.003007935	161.5382025
18487653.09	0.003102205	24182.7109	14.50127004
18487653.09	0.003102205	24182.7109	14.50127004
26.39163536	6073.744781	96.4708329	4.900801524
18487653.09	0.003102205	24182.7109	14.50127004
3521.376477	0.061106073	51.99743357	0.084609634
0.002079684	24.86448114	2.542027574	1.103747429
26.39163536	6073.744781	96.4708329	4.900801524
0.00367292	205.3538926	107.7500234	8.656298264
0.000621218	0.001051526	0.000235066	0.776173373
444.9439191	493.0399195	220.5042306	111.6163211
92.71200379	85.77269248	6.14E-05	0.055037026
18487653.09	0.003102205	24182.7109	14.50127004
26.39163536	6073.744781	96.4708329	4.900801524
44.04480964	396.4183114	16.81160169	22.21980926
26.39163536	6073.744781	96.4708329	4.900801524
0.002899469	44.20196441	82.72672859	1.433566977
1004933.283	1.201854084	0.002549948	151.0739372
18487653.09	0.003102205	24182.7109	14.50127004
444.9439191	493.0399195	220.5042306	111.6163211
444.9439191	493.0399195	220.5042306	111.6163211
92.71200379	85.77269248	6.14E-05	0.055037026
0.00367292	205.3538926	107.7500234	8.656298264
18487653.09	0.003102205	24182.7109	14.50127004
2.921087263	291.1689411	0.003007935	161.5382025
26.39163536	6073.744781	96.4708329	4.900801524

444.9439191	493.0399195	220.5042306	111.6163211
0.003255347	46.61255529	0.001231806	24.23669999
238.5822635	1254.567176	0.011078274	17.44998909
0.002341657	0.003963682	2545.630641	2.259819368
444.9439191	493.0399195	220.5042306	111.6163211
92.71200379	85.77269248	6.14E-05	0.055037026
2.921087263	291.1689411	0.003007935	161.5382025
18487653.09	0.003102205	24182.7109	14.50127004
44.04480964	396.4183114	16.81160169	22.21980926
444.9439191	493.0399195	220.5042306	111.6163211
18487653.09	0.003102205	24182.7109	14.50127004
18487653.09	0.003102205	24182.7109	14.50127004
26.39163536	6073.744781	96.4708329	4.900801524
444.9439191	493.0399195	220.5042306	111.6163211
0.003255347	46.61255529	0.001231806	24.23669999
18487653.09	0.003102205	24182.7109	14.50127004
0.003255347	46.61255529	0.001231806	24.23669999
44.04480964	396.4183114	16.81160169	22.21980926
26.10673933	0.007788311	0.065280254	2.335333077
44.04480964	396.4183114	16.81160169	22.21980926
444.9439191	493.0399195	220.5042306	111.6163211
44.04480964	396.4183114	16.81160169	22.21980926
26.39163536	6073.744781	96.4708329	4.900801524
1004933.283	1.201854084	0.002549948	151.0739372
443.3872409	0.00542826	8.971843259	34.66796735
18487653.09	0.003102205	24182.7109	14.50127004
36472.41117	138.9546577	2.804091203	29.94239575
0.003255347	46.61255529	0.001231806	24.23669999
238.5822635	1254.567176	0.011078274	17.44998909
0.003255347	46.61255529	0.001231806	24.23669999
444.9439191	493.0399195	220.5042306	111.6163211
0.002341657	0.003963682	2545.630641	2.259819368
1004933.283	1.201854084	0.002549948	151.0739372
238.5822635	1254.567176	0.011078274	17.44998909
92.71200379	85.77269248	6.14E-05	0.055037026
36472.41117	138.9546577	2.804091203	29.94239575
92.71200379	85.77269248	6.14E-05	0.055037026
444.9439191	493.0399195	220.5042306	111.6163211
0.00013296	48.99647608	0.000829599	1.426632815
18487653.09	0.003102205	24182.7109	14.50127004
444.9439191	493.0399195	220.5042306	111.6163211

36472.41117	138.9546577	2.804091203	29.94239575
18487653.09	0.003102205	24182.7109	14.50127004
26.39163536	6073.744781	96.4708329	4.900801524
0.000621218	0.001051526	0.000235066	0.776173373
26.39163536	6073.744781	96.4708329	4.900801524
26.39163536	6073.744781	96.4708329	4.900801524
18487653.09	0.003102205	24182.7109	14.50127004
36472.41117	138.9546577	2.804091203	29.94239575
92.71200379	85.77269248	6.14E-05	0.055037026
444.9439191	493.0399195	220.5042306	111.6163211
18487653.09	0.003102205	24182.7109	14.50127004
444.9439191	493.0399195	220.5042306	111.6163211
0.002899469	44.20196441	82.72672859	1.433566977
26.39163536	6073.744781	96.4708329	4.900801524
26.39163536	6073.744781	96.4708329	4.900801524
238.5822635	1254.567176	0.011078274	17.44998909
4289681.134	390326.2634	16963.31967	212.054701
92.71200379	85.77269248	6.14E-05	0.055037026
0.003255347	46.61255529	0.001231806	24.23669999
6.543337611	0.005625601	73.90578451	8.944156771
444.9439191	493.0399195	220.5042306	111.6163211
0.00013296	48.99647608	0.000829599	1.426632815
0.002341657	0.003963682	2545.630641	2.259819368
541278.185	83.3727028	1.350728114	42.74555378
44.04480964	396.4183114	16.81160169	22.21980926
0.000621218	0.001051526	0.000235066	0.776173373
64302.06533	19.67707718	8.016328145	11.7087833
4699.689732	16.5445412	56.996003	3.920494479
0.00327203	8.814760671	0.003052285	0.067559455
444.9439191	493.0399195	220.5042306	111.6163211
92.71200379	85.77269248	6.14E-05	0.055037026
36472.41117	138.9546577	2.804091203	29.94239575
17.97047524	3137.655643	5107264.194	0.110313418
26.39163536	6073.744781	96.4708329	4.900801524
18487653.09	0.003102205	24182.7109	14.50127004
0.00327203	8.814760671	0.003052285	0.067559455
541278.185	83.3727028	1.350728114	42.74555378
26.39163536	6073.744781	96.4708329	4.900801524
26.39163536	6073.744781	96.4708329	4.900801524
6.543337611	0.005625601	73.90578451	8.944156771
16.79057795	54.01345836	215.3946583	90.95807077

2.921087263	291.1689411	0.003007935	161.5382025
26.39163536	6073.744781	96.4708329	4.900801524
326298.382	1511.906117	32422.88517	47.19092299
26.39163536	6073.744781	96.4708329	4.900801524
18487653.09	0.003102205	24182.7109	14.50127004
16.79057795	54.01345836	215.3946583	90.95807077
36472.41117	138.9546577	2.804091203	29.94239575
26.39163536	6073.744781	96.4708329	4.900801524
16.79057795	54.01345836	215.3946583	90.95807077
0.002341657	0.003963682	2545.630641	2.259819368
26.39163536	6073.744781	96.4708329	4.900801524
26.39163536	6073.744781	96.4708329	4.900801524
443.3872409	0.00542826	8.971843259	34.66796735
444.9439191	493.0399195	220.5042306	111.6163211
2.921087263	291.1689411	0.003007935	161.5382025
444.9439191	493.0399195	220.5042306	111.6163211
26.39163536	6073.744781	96.4708329	4.900801524
36472.41117	138.9546577	2.804091203	29.94239575
0.00013296	48.99647608	0.000829599	1.426632815
18487653.09	0.003102205	24182.7109	14.50127004
0.000621218	0.001051526	0.000235066	0.776173373
238.5822635	1254.567176	0.011078274	17.44998909
444.9439191	493.0399195	220.5042306	111.6163211
36472.41117	138.9546577	2.804091203	29.94239575
26.39163536	6073.744781	96.4708329	4.900801524
26.39163536	6073.744781	96.4708329	4.900801524
18487653.09	0.003102205	24182.7109	14.50127004
18487653.09	0.003102205	24182.7109	14.50127004
18487653.09	0.003102205	24182.7109	14.50127004
44.04480964	396.4183114	16.81160169	22.21980926
18487653.09	0.003102205	24182.7109	14.50127004
238.5822635	1254.567176	0.011078274	17.44998909
26.39163536	6073.744781	96.4708329	4.900801524
26.39163536	6073.744781	96.4708329	4.900801524
0.000621218	0.001051526	0.000235066	0.776173373
541278.185	83.3727028	1.350728114	42.74555378
4699.689732	16.5445412	56.996003	3.920494479
0.270067477	0.377395125	24.08311636	2.057954211
0.002079684	24.86448114	2.542027574	1.103747429
16.79057795	54.01345836	215.3946583	90.95807077
18487653.09	0.003102205	24182.7109	14.50127004

238.5822635	1254.567176	0.011078274	17.44998909
0.002341657	0.003963682	2545.630641	2.259819368
0.002341657	0.003963682	2545.630641	2.259819368
0.002341657	0.003963682	2545.630641	2.259819368
26.39163536	6073.744781	96.4708329	4.900801524
18487653.09	0.003102205	24182.7109	14.50127004
26.39163536	6073.744781	96.4708329	4.900801524
444.9439191	493.0399195	220.5042306	111.6163211
16.79057795	54.01345836	215.3946583	90.95807077
444.9439191	493.0399195	220.5042306	111.6163211
0.002079684	24.86448114	2.542027574	1.103747429
18487653.09	0.003102205	24182.7109	14.50127004
26.39163536	6073.744781	96.4708329	4.900801524
0.003255347	46.61255529	0.001231806	24.23669999
444.9439191	493.0399195	220.5042306	111.6163211
654.7408322	0.000833079	3195.258006	2.52234018
18487653.09	0.003102205	24182.7109	14.50127004
18487653.09	0.003102205	24182.7109	14.50127004
0.003255347	46.61255529	0.001231806	24.23669999
0.00013296	48.99647608	0.000829599	1.426632815
18487653.09	0.003102205	24182.7109	14.50127004
17.97047524	3137.655643	5107264.194	0.110313418
444.9439191	493.0399195	220.5042306	111.6163211
92.71200379	85.77269248	6.14E-05	0.055037026
0.003255347	46.61255529	0.001231806	24.23669999
443.3872409	0.00542826	8.971843259	34.66796735
18487653.09	0.003102205	24182.7109	14.50127004
26.10673933	0.007788311	0.065280254	2.335333077
4289681.134	390326.2634	16963.31967	212.054701
444.9439191	493.0399195	220.5042306	111.6163211
16.79057795	54.01345836	215.3946583	90.95807077
0.002341657	0.003963682	2545.630641	2.259819368
444.9439191	493.0399195	220.5042306	111.6163211
18487653.09	0.003102205	24182.7109	14.50127004
23454.39012	186404.0517	0.673319243	88.35793893
26.39163536	6073.744781	96.4708329	4.900801524
2.921087263	291.1689411	0.003007935	161.5382025
2.306166232	2.799578684	0.000113672	0.425449008
18487653.09	0.003102205	24182.7109	14.50127004
26.39163536	6073.744781	96.4708329	4.900801524
238.5822635	1254.567176	0.011078274	17.44998909

18487653.09	0.003102205	24182.7109	14.50127004
2.210941183	806.7370046	0.001185215	0.396914612
92.71200379	85.77269248	6.14E-05	0.055037026
26.39163536	6073.744781	96.4708329	4.900801524
18487653.09	0.003102205	24182.7109	14.50127004
444.9439191	493.0399195	220.5042306	111.6163211
444.9439191	493.0399195	220.5042306	111.6163211
444.9439191	493.0399195	220.5042306	111.6163211
18487653.09	0.003102205	24182.7109	14.50127004
26.39163536	6073.744781	96.4708329	4.900801524
18487653.09	0.003102205	24182.7109	14.50127004
18487653.09	0.003102205	24182.7109	14.50127004
26.39163536	6073.744781	96.4708329	4.900801524
654.7408322	0.000833079	3195.258006	2.52234018
23454.39012	186404.0517	0.673319243	88.35793893
444.9439191	493.0399195	220.5042306	111.6163211
18487653.09	0.003102205	24182.7109	14.50127004
26.39163536	6073.744781	96.4708329	4.900801524
26.39163536	6073.744781	96.4708329	4.900801524
0.00013296	48.99647608	0.000829599	1.426632815
26.39163536	6073.744781	96.4708329	4.900801524
0.00367292	205.3538926	107.7500234	8.656298264
0.002079684	24.86448114	2.542027574	1.103747429
18487653.09	0.003102205	24182.7109	14.50127004
16.79057795	54.01345836	215.3946583	90.95807077
18487653.09	0.003102205	24182.7109	14.50127004
18487653.09	0.003102205	24182.7109	14.50127004
64302.06533	19.67707718	8.016328145	11.7087833
238.5822635	1254.567176	0.011078274	17.44998909
0.002341657	0.003963682	2545.630641	2.259819368
26.39163536	6073.744781	96.4708329	4.900801524
4289681.134	390326.2634	16963.31967	212.054701
26.10673933	0.007788311	0.065280254	2.335333077
541278.185	83.3727028	1.350728114	42.74555378
444.9439191	493.0399195	220.5042306	111.6163211
26.39163536	6073.744781	96.4708329	4.900801524
444.9439191	493.0399195	220.5042306	111.6163211
2.210941183	806.7370046	0.001185215	0.396914612
0.00367292	205.3538926	107.7500234	8.656298264
18487653.09	0.003102205	24182.7109	14.50127004
0.00013296	48.99647608	0.000829599	1.426632815

26.10673933	0.007788311	0.065280254	2.335333077
18487653.09	0.003102205	24182.7109	14.50127004
444.9439191	493.0399195	220.5042306	111.6163211
326298.382	1511.906117	32422.88517	47.19092299
26.39163536	6073.744781	96.4708329	4.900801524
444.9439191	493.0399195	220.5042306	111.6163211
444.9439191	493.0399195	220.5042306	111.6163211
258.8753146	447.4771016	676287.9066	6.143344583
0.00013296	48.99647608	0.000829599	1.426632815
0.002341657	0.003963682	2545.630641	2.259819368
26.39163536	6073.744781	96.4708329	4.900801524
18487653.09	0.003102205	24182.7109	14.50127004
26.39163536	6073.744781	96.4708329	4.900801524
0.002079684	24.86448114	2.542027574	1.103747429
541278.185	83.3727028	1.350728114	42.74555378
444.9439191	493.0399195	220.5042306	111.6163211
92.71200379	85.77269248	6.14E-05	0.055037026
238.5822635	1254.567176	0.011078274	17.44998909
9.40E-05	1427.057408	23.31089059	0.107337433
0.00327203	8.814760671	0.003052285	0.067559455
0.270067477	0.377395125	24.08311636	2.057954211
2.921087263	291.1689411	0.003007935	161.5382025
0.003255347	46.61255529	0.001231806	24.23669999
443.3872409	0.00542826	8.971843259	34.66796735
18487653.09	0.003102205	24182.7109	14.50127004
26.39163536	6073.744781	96.4708329	4.900801524
541278.185	83.3727028	1.350728114	42.74555378
16.79057795	54.01345836	215.3946583	90.95807077
92.71200379	85.77269248	6.14E-05	0.055037026
444.9439191	493.0399195	220.5042306	111.6163211
444.9439191	493.0399195	220.5042306	111.6163211
18487653.09	0.003102205	24182.7109	14.50127004
26.39163536	6073.744781	96.4708329	4.900801524
238.5822635	1254.567176	0.011078274	17.44998909
326298.382	1511.906117	32422.88517	47.19092299
325.3362474	0.953297937	0.82421472	1.817875669
18487653.09	0.003102205	24182.7109	14.50127004
26.39163536	6073.744781	96.4708329	4.900801524
44.04480964	396.4183114	16.81160169	22.21980926
26.39163536	6073.744781	96.4708329	4.900801524
36472.41117	138.9546577	2.804091203	29.94239575

0.003255347	46.61255529	0.001231806	24.23669999
444.9439191	493.0399195	220.5042306	111.6163211
238.5822635	1254.567176	0.011078274	17.44998909
92.71200379	85.77269248	6.14E-05	0.055037026
0.002079684	24.86448114	2.542027574	1.103747429
18487653.09	0.003102205	24182.7109	14.50127004
0.002341657	0.003963682	2545.630641	2.259819368
0.270067477	0.377395125	24.08311636	2.057954211
541278.185	83.3727028	1.350728114	42.74555378
36472.41117	138.9546577	2.804091203	29.94239575
92.71200379	85.77269248	6.14E-05	0.055037026
64302.06533	19.67707718	8.016328145	11.7087833
0.002341657	0.003963682	2545.630641	2.259819368
23454.39012	186404.0517	0.673319243	88.35793893
26.39163536	6073.744781	96.4708329	4.900801524
0.002341657	0.003963682	2545.630641	2.259819368
26.39163536	6073.744781	96.4708329	4.900801524
92.71200379	85.77269248	6.14E-05	0.055037026
444.9439191	493.0399195	220.5042306	111.6163211
92.71200379	85.77269248	6.14E-05	0.055037026
18487653.09	0.003102205	24182.7109	14.50127004
444.9439191	493.0399195	220.5042306	111.6163211
18487653.09	0.003102205	24182.7109	14.50127004
4289681.134	390326.2634	16963.31967	212.054701
92.71200379	85.77269248	6.14E-05	0.055037026
26.10673933	0.007788311	0.065280254	2.335333077
2.921087263	291.1689411	0.003007935	161.5382025
444.9439191	493.0399195	220.5042306	111.6163211
0.003255347	46.61255529	0.001231806	24.23669999
444.9439191	493.0399195	220.5042306	111.6163211
4289681.134	390326.2634	16963.31967	212.054701
92.71200379	85.77269248	6.14E-05	0.055037026
6.543337611	0.005625601	73.90578451	8.944156771
541278.185	83.3727028	1.350728114	42.74555378
64302.06533	19.67707718	8.016328145	11.7087833
541278.185	83.3727028	1.350728114	42.74555378
18487653.09	0.003102205	24182.7109	14.50127004
325.3362474	0.953297937	0.82421472	1.817875669
92.71200379	85.77269248	6.14E-05	0.055037026
16.79057795	54.01345836	215.3946583	90.95807077
0.00367292	205.3538926	107.7500234	8.656298264

325.3362474	0.953297937	0.82421472	1.817875669
443.3872409	0.00542826	8.971843259	34.66796735
26.39163536	6073.744781	96.4708329	4.900801524
26.39163536	6073.744781	96.4708329	4.900801524
18487653.09	0.003102205	24182.7109	14.50127004
6.543337611	0.005625601	73.90578451	8.944156771
44.04480964	396.4183114	16.81160169	22.21980926
0.002652878	52.58453061	249.5887533	4.956175605
0.003255347	46.61255529	0.001231806	24.23669999
6.543337611	0.005625601	73.90578451	8.944156771
0.003407341	0.005767548	220.7173297	0.000234915
26.39163536	6073.744781	96.4708329	4.900801524
238.5822635	1254.567176	0.011078274	17.44998909
2.306166232	2.799578684	0.000113672	0.425449008
0.002899469	44.20196441	82.72672859	1.433566977
6.543337611	0.005625601	73.90578451	8.944156771
0.00367292	205.3538926	107.7500234	8.656298264
92.71200379	85.77269248	6.14E-05	0.055037026
26.39163536	6073.744781	96.4708329	4.900801524
1004933.283	1.201854084	0.002549948	151.0739372
444.9439191	493.0399195	220.5042306	111.6163211
16.79057795	54.01345836	215.3946583	90.95807077
18487653.09	0.003102205	24182.7109	14.50127004
4699.689732	16.5445412	56.996003	3.920494479
0.002341657	0.003963682	2545.630641	2.259819368
36472.41117	138.9546577	2.804091203	29.94239575
17.97047524	3137.655643	5107264.194	0.110313418
444.9439191	493.0399195	220.5042306	111.6163211
16.79057795	54.01345836	215.3946583	90.95807077
36472.41117	138.9546577	2.804091203	29.94239575
92.71200379	85.77269248	6.14E-05	0.055037026
6.543337611	0.005625601	73.90578451	8.944156771
16.79057795	54.01345836	215.3946583	90.95807077
325.3362474	0.953297937	0.82421472	1.817875669
0.002652878	52.58453061	249.5887533	4.956175605
6.543337611	0.005625601	73.90578451	8.944156771
26.39163536	6073.744781	96.4708329	4.900801524
26.10673933	0.007788311	0.065280254	2.335333077
6.543337611	0.005625601	73.90578451	8.944156771
0.002341657	0.003963682	2545.630641	2.259819368
26.39163536	6073.744781	96.4708329	4.900801524

444.9439191	493.0399195	220.5042306	111.6163211
1004933.283	1.201854084	0.002549948	151.0739372
36472.41117	138.9546577	2.804091203	29.94239575
23454.39012	186404.0517	0.673319243	88.35793893
0.002079684	24.86448114	2.542027574	1.103747429
4699.689732	16.5445412	56.996003	3.920494479
0.00013296	48.99647608	0.000829599	1.426632815
0.002341657	0.003963682	2545.630641	2.259819368
0.002341657	0.003963682	2545.630641	2.259819368
18487653.09	0.003102205	24182.7109	14.50127004
16.79057795	54.01345836	215.3946583	90.95807077
36472.41117	138.9546577	2.804091203	29.94239575
0.00367292	205.3538926	107.7500234	8.656298264
444.9439191	493.0399195	220.5042306	111.6163211
0.002079684	24.86448114	2.542027574	1.103747429
18487653.09	0.003102205	24182.7109	14.50127004
444.9439191	493.0399195	220.5042306	111.6163211
6.618400234	618982.885	8812.10418	10.11805667
258.8753146	447.4771016	676287.9066	6.143344583
0.002079684	24.86448114	2.542027574	1.103747429
0.00327203	8.814760671	0.003052285	0.067559455
0.00367292	205.3538926	107.7500234	8.656298264
0.00013296	48.99647608	0.000829599	1.426632815
0.003255347	46.61255529	0.001231806	24.23669999
0.002899469	44.20196441	82.72672859	1.433566977
541278.185	83.3727028	1.350728114	42.74555378
36472.41117	138.9546577	2.804091203	29.94239575
238.5822635	1254.567176	0.011078274	17.44998909
26.39163536	6073.744781	96.4708329	4.900801524
326298.382	1511.906117	32422.88517	47.19092299
654.7408322	0.000833079	3195.258006	2.52234018
444.9439191	493.0399195	220.5042306	111.6163211
0.00367292	205.3538926	107.7500234	8.656298264
36472.41117	138.9546577	2.804091203	29.94239575
36472.41117	138.9546577	2.804091203	29.94239575
0.00327203	8.814760671	0.003052285	0.067559455
16.79057795	54.01345836	215.3946583	90.95807077
325.3362474	0.953297937	0.82421472	1.817875669
23454.39012	186404.0517	0.673319243	88.35793893
0.002341657	0.003963682	2545.630641	2.259819368
2.306166232	2.799578684	0.000113672	0.425449008

0.003407341	0.005767548	220.7173297	0.000234915
444.9439191	493.0399195	220.5042306	111.6163211
238.5822635	1254.567176	0.011078274	17.44998909
0.002341657	0.003963682	2545.630641	2.259819368
444.9439191	493.0399195	220.5042306	111.6163211
0.003255347	46.61255529	0.001231806	24.23669999
6.543337611	0.005625601	73.90578451	8.944156771
16.79057795	54.01345836	215.3946583	90.95807077
0.00367292	205.3538926	107.7500234	8.656298264
18487653.09	0.003102205	24182.7109	14.50127004
16.79057795	54.01345836	215.3946583	90.95807077
26.39163536	6073.744781	96.4708329	4.900801524
2.306166232	2.799578684	0.000113672	0.425449008
0.002341657	0.003963682	2545.630641	2.259819368
4699.689732	16.5445412	56.996003	3.920494479
18487653.09	0.003102205	24182.7109	14.50127004
4699.689732	16.5445412	56.996003	3.920494479
18487653.09	0.003102205	24182.7109	14.50127004
51	52	53	54
PRDM15-Hs00411318_m1	COL6A1-Hs01095585_m1	ABCG1-Hs01555191_m1	GART-Hs00531926_m1
